# Regiodivergent Nucleophilic Fluorination under Hydrogen Bonding Catalysis: A Computational and Experimental Study

**DOI:** 10.1021/jacs.3c01303

**Published:** 2023-04-20

**Authors:** Matthew A. Horwitz, Alexander B. Dürr, Konstantinos Afratis, Zijun Chen, Julia Soika, Kirsten E. Christensen, Makoto Fushimi, Robert S. Paton, Véronique Gouverneur

**Affiliations:** † Chemistry Research Laboratory, University of Oxford, 12 Mansfield Road, Oxford OX1 3TA, U.K; ‡ Takeda Pharmaceutical Company Limited, 26-1, Muraoka-Higashi 2-Chome, Fujisawa, Kanagawa 251-8555, Japan; § Department of Chemistry, Colorado State University, Fort Collins, Colorado 80528, United States

## Abstract

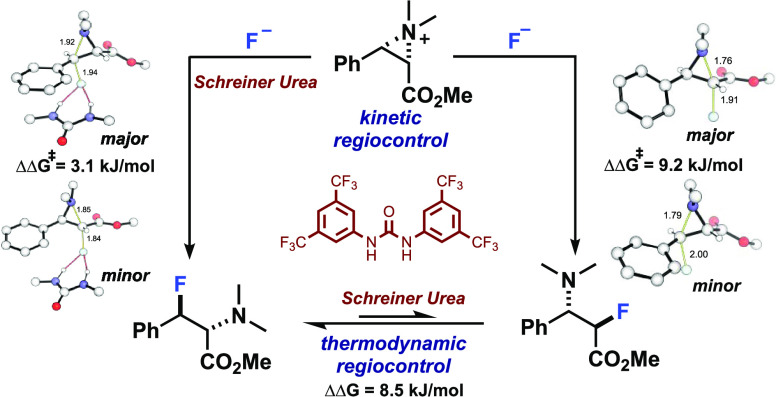

The controlled programming of regiochemical outcomes in nucleophilic fluorination reactions with alkali metal fluoride is a problem yet to be solved. Herein, two synergistic approaches exploiting hydrogen bonding catalysis are presented. First, we demonstrate that modulating the charge density of fluoride with a hydrogen-bond donor urea catalyst directly influences the kinetic regioselectivity in the fluorination of dissymmetric aziridinium salts with aryl and ester substituents. Moreover, we report a urea-catalyzed formal dyotropic rearrangement, a thermodynamically controlled regiochemical editing process consisting of C–F bond scission followed by fluoride rebound. These findings offer a route to access enantioenriched fluoroamine regioisomers from a single chloroamine precursor, and more generally, new opportunities in regiodivergent asymmetric (*bis*)urea-based organocatalysis.

## Introduction

The development and widespread application of asymmetric organocatalysis was recognized with the Nobel Prize in Chemistry awarded in 2021 to List and MacMillan.^1^ Organocatalysts were also deployed to control regioselectivity including examples of asymmetric regiodivergent reactions.^2^ Standout examples are the development of the regiocontrolled *N*-alkylation of triazoles with an amidinium hydrogen-bond donor (HBD) catalyst^3^ and an asymmetric regiodivergent cycloaddition catalyzed by an *N*-heterocyclic carbene leading to regioisomeric dihydroisoquinolines.^4^

**Scheme 1:**
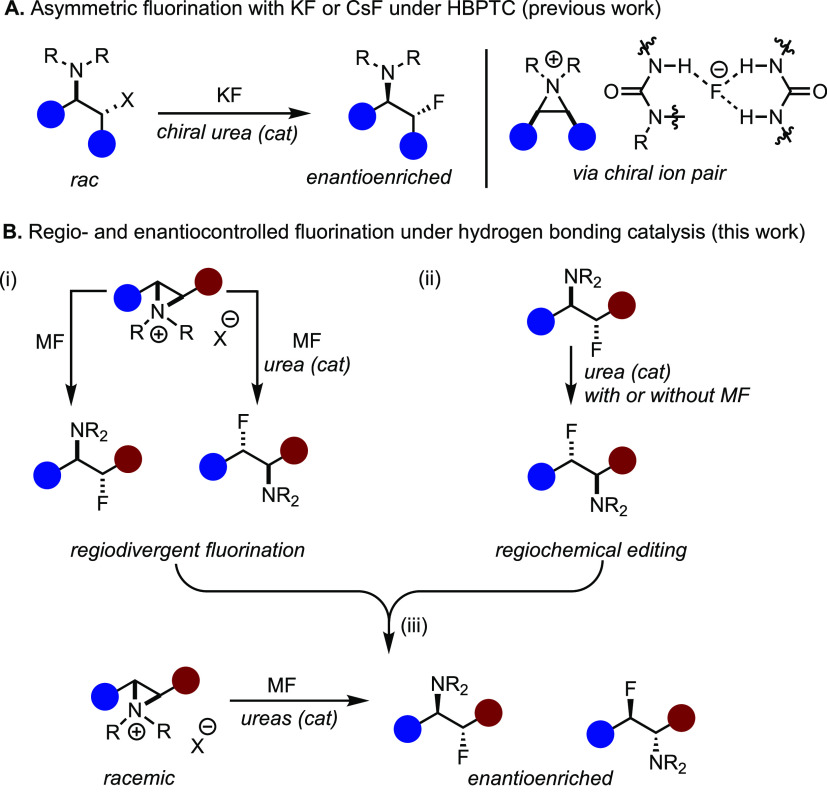
Regiocontrolled Ring Opening of Aziridinium ions with Alkali Metal Fluoride (A) Enantioselective fluorination of *meso*-aziridinium salts with KF under HBPTC; (B) this work: (i) kinetic regiodivergence in nucleophilic substitution with alkali metal fluoride under hydrogen bonding catalysis, (ii) thermodynamic regiochemical editing under hydrogen bonding catalysis, and (iii) regiodivergent kinetic resolution of dissymmetric aziridinium ions with alkali metal fluoride.

As part of our ongoing studies into nucleophilic fluorination under hydrogen bonding phase-transfer catalysis (HBPTC),^5^ the issue of regiocontrolled fluorination arose. In 2019, we reported the enantioselective desymmetrization of *meso*-aziridinium ions with alkali metal fluoride using BINAM-derived *N*-alkylated *bis*-urea catalysts (Scheme 1A).^5d^ The resulting β-fluoroamines are formed in high yields and enantiomeric excesses. In this process, the insoluble fluoride salt is solubilized in the presence of a chiral HBD *bis*-urea phase transfer catalyst. The resulting hydrogen-bonded fluoride is a competent nucleophile in the enantiocontrolled reaction with the cationic electrophilic aziridinium partner via the formation of a chiral ion pair. Building on these precedents, hydrogen bonding catalysis applied to the regiocontrolled fluorination of dissymmetric aziridinium salts would represent a significant advance, with the prospect of offering new opportunities in regiodivergent asymmetric organocatalysis. The most attractive scenario would employ a chiral hydrogen-bond donor catalyst to accelerate the fluorination of both enantiomers of a racemic mixture along regiodivergent pathways.

We noted two examples of regiodivergent parallel kinetic resolutions of aziridines with nitrogen and carbon nucleophiles carried out under transition-metal catalysis. In 2009, Parquette and RajanBabu reported that a dimeric yttrium-salen complex can induce divergent regioselectivities in the ring-opening reaction of racemic terminal aziridines with trimethylsilylazide.^6^ More recently, Shibasaki and Matsunaga broadened the synthetic utility of the process to internal aziridines and nucleophilic malonates applying combined Lewis acid [Y(OTf)_3_] and Brønsted base [La(O*i*Pr)_3_] catalysis.^7^ To date, no solution is available to program the regiochemical outcome of the ring opening of dissymmetric aziridinium salts using an inexpensive alkali metal fluoride as the fluorinating reagent.

Herein, we report new approaches for regiocontrolled fluorination using HBD catalysis.^8^ First, we demonstrate that the kinetic regiopreference in the fluorination of dissymmetric β-chloroamines with an alkali metal fluoride can be inverted in the presence of a hydrogen bonding urea catalyst that attenuates the charge of fluoride (Scheme 1B, (i)). Second, the regiochemical editing of vicinal fluoroamines, consisting of C–F bond scission followed by fluoride rebound under hydrogen bonding catalysis, is unveiled (Scheme 1B, (ii)). Third, we disclose a protocol to access enantioenriched regioisomeric α- and β-fluoroamines using KF, a chiral BINAM-derived *bis*-urea catalyst, and the Schreiner’s urea catalyst (Scheme 1B, (iii)).

## Results and Discussion

### Regiocontrolled Ring Opening of *rac*-Aziridinium Ions with Alkali Metal Fluoride with and without Hydrogen-Bond Donor Catalyst: A Computational and Experimental Analysis

**Figure 1:**
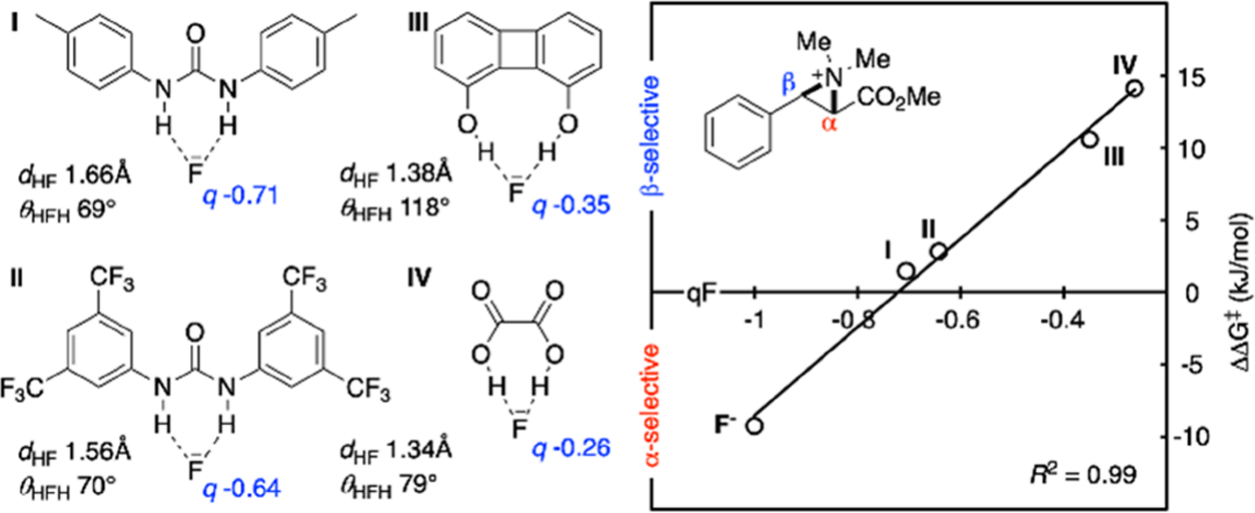
Computed levels of regioselectivity in nucleophilic fluorination with and without hydrogen-bond donor coordination (ωB97X-D3/(ma)-def2-TZVPP)//M06-2X/def2-SVP(TZVPPD).^13^ Löwdin charges *qF* are shown.

Sharpless and co-workers reported that the regiochemical preference for the ring opening of aziridinium salts with phenyl and ester substitution depends on the nucleophile.^9^ Thiolate nucleophiles react α to the carboxylic ester, while opening at the benzylic position was preferred for amine nucleophiles. Controlling access to one or the other regioisomer upon opening of a dissymmetric aziridinium salt with an alkali metal fluoride nucleophile is, therefore, not a trivial problem.^10^ We envisioned that attenuation of the charge density of fluoride through hydrogen bonding may be a viable approach to reroute nucleophilic substitution toward the otherwise disfavored regioisomer. Preliminary computational studies were performed to explore this hypothesis. We considered the addition of a fluoride nucleophile to a dissymmetric aziridinium ion with phenyl and methyl ester substituents (Figure 1).

Quantum chemical calculations were used to compute the regioisomeric transition structures (TSs) and to predict the regioselectivity (ΔΔ*G*^‡^) for a free fluoride anion and fluoride bound to four bidentate HBD moieties.^11,12^ The predicted regioselectivity is highly dependent upon the strength of coordination to the fluoride anion: while a free fluoride favors addition to the α-position by 9 kJ/mol, coordination to oxalic acid (IV) results in a preference for the β-position by 14 kJ/mol. Indeed, upon collecting structural and electronic parameters for the fluoride species I–IV, we obtained a linear correlation between ΔΔ*G*^‡^ for nucleophilic attack and the charge on F. As the HBD acidity increases, more negative charge is transferred from F in the complex and tighter complexes (with shorter H-F distances) are formed. These studies indicate that the electronic environment on fluoride, and hence control over regioselectivity in nucleophilic fluorination, can be rationally tuned through bidentate coordination with HBD catalysts.

We then turned to the experimental validation of this hypothesis with dissymmetric nonterminal aziridinium chlorides. The ring opening of *rac*-aziridinium chloride derived from the model *rac*-2-amino-3-chloro-3-aryl propanoic ester **1a** served to investigate the ability of 1,3-*bis*[3,5-*bis*(trifluoromethyl)phenyl]urea (SU, Schreiner’s urea) to influence regioselectivity.

Fluorination with CsF in DCM at ambient temperature afforded preferentially β-amino-α-fluoroester **2a** (r.r. > 20:1, α:β) (Table 1, entry 1). The low conversion of 34% was expected as the reaction was carried out in the absence of an exogenous phase transfer agent for CsF solubilization. In the presence of 20 mol % of SU under otherwise identical conditions, fluorination is quantitative due to the ability of the SU catalyst to bring CsF into solution. Significantly, the kinetic regiopreference of fluoride is reversed, now favoring the formation of the β-fluoro-α-aminoester **3a** (r.r. = 1:2.6, α:β) (Table 1, entry 2). The urea-catalyzed reaction also led predominantly to **3a** with KF (r.r. = 1:2.8, α:β), a fluoride source that does not react in the absence of SU catalyst (Table 1, entries 3 and 4). Similar trends were obtained in 1,2-difluorobenzene, a solvent of choice for fluorination reactions carried out under HBPTC^6^ (Table 1, entries 5–8). The soluble fluoride source NEt_3_·3HF displayed preferential β-fluorination, but **2a** and **3a** were formed in low yields due to the decomposition of the starting material (Table 1, entry 9). A poor yield was also observed with TBAF, a reagent displaying overall poor regiocontrol (Table 1, entry 10). Slow release of soluble fluoride, which is achieved with alkali metal fluoride brought into solution by the urea phase transfer catalyst, enhances conversion to the desired fluorinated products.

**Table 1:**
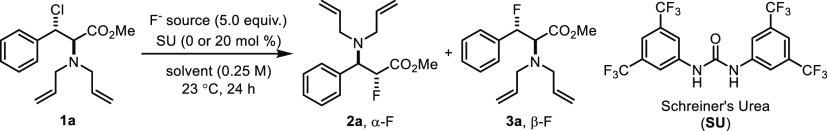
Regiodivergence in Nucleophilic Fluorination of **1a** with and without Schreiner’s Urea Catalyst^a^

entry	fluoride source	solvent	SU (mol %)	yield^b^	r.r.^c^**2a:3a**
1	CsF	DCM	0	34%	>20:1
2	CsF	DCM	20	>95%	1:2.6
3	KF	DCM	0	0%	
4	KF	DCM	20	>95%	1:2.8
5	CsF	1,2-DFB	0	4%	**α** only
6	CsF	1,2-DFB	20	70%	1:2.4
7	KF	1,2-DFB	0	0%	
8	KF	1,2-DFB	20	>95%	1:4
9	NEt_3_·3HF	DCM	0	<10%	1:15
10	TBAF^d^	DCM	0	20%	1.3:1

a Reaction conditions: 0.1 mmol of **1a**, SU (0 or 20 mol %), and KF, CsF, or NEt_3_·3HF (5.0 equiv) were stirred in dichloromethane (0.25 M) at 1200 rpm for 24 h.

b Determined by ^1^H NMR using 1,3,5-trimethoxybenzene as an internal standard.

c r.r. = regioisomeric ratio, determined by ^1^H NMR of crude mixture.

d 6.0 equiv tetrabutylammonium fluoride. SU = Schreiner’s urea.

Considering that hydrogen-bond donors are capable of C–F bond polarization,^14^ the possibility of reversible fluorination was probed experimentally. These experiments were carried out in 1,2-difluorobenzene (b.p. 94 °C). We subjected the α-fluorinated product **2a** to the reaction conditions at increasing temperatures. After 24 h at room temperature, the β-regioisomer was detectable (Table 2, entry 1), and as the temperature increased, the defluorination-fluorination pathway became more efficient (Table 2, entries 2–3).^15^ A control experiment demonstrated that the α-regioisomer **2a** is fully recovered in the absence of Schreiner’s urea catalyst at 60 °C (Table 2, entry 4). Moreover, β-**3a** was formed in 59% yield in the absence of KF under hydrogen bonding catalysis, a unique example of regiochemical editing through organocatalytic C–F bond scission and fluoride rebound (Table 2, entry 5).^16^ The conversion of **2a** to **3a** constitutes a formal organocatalyzed dyotropic rearrangement with no erosion of stereochemistry observed upon fluoride transfer.

**Table 2:**
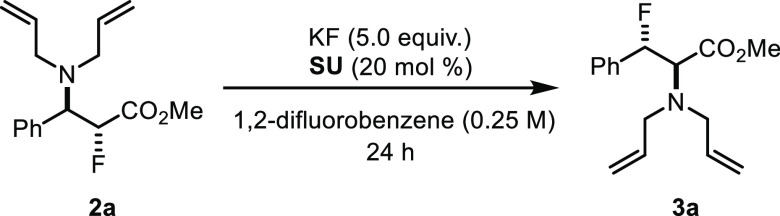
Regiochemical Editing through a Urea-Catalyzed Defluorination-Fluorination Pathway^a^

entry	*T* (°C)	yield^b^ (**2a** + **3a**)	r.r.^c^ (**2a:3a**)
1	23	>95%	13.4:1
2	40	>95%	2.9:1
3	60	>95%	1:20
4^d^	60	>95%	**α** only
5^e^	60	59%	1:6.8

a Reaction conditions: 0.1 mmol of **2a**, SU (20 mol %), and KF (5 equiv) were stirred in 1,2-difluorobenzene at 1200 rpm for 24 h.

b Determined by ^1^H NMR using 1,3,5-trimethoxybenzene as an internal standard.

c r.r. = regioisomeric ratio, determined by ^1^H NMR of crude mixture.

d Reaction performed without Schreiner’s urea (SU).

e Reaction performed without KF. SU = Schreiner’s urea.

**Scheme 2:**
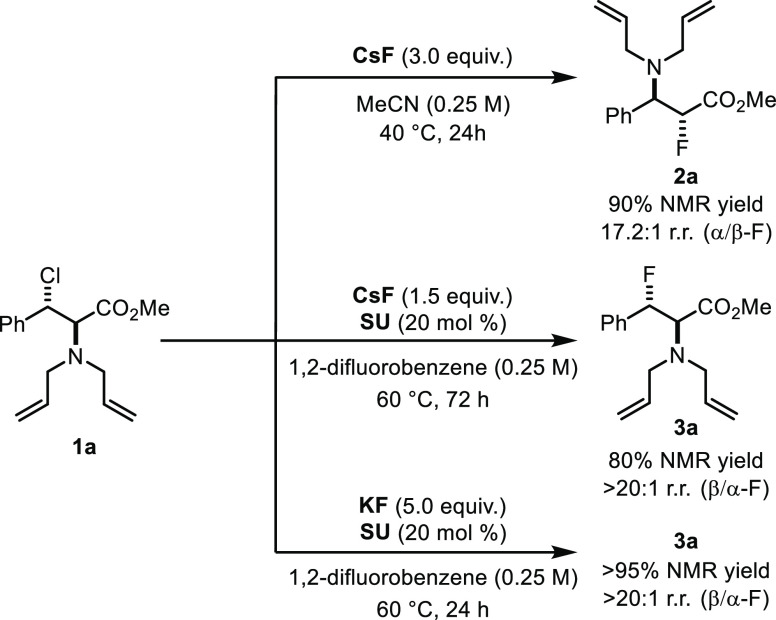
Regiodivergence in Nucleophilic Fluorination with Alkali Metal Fluoride SU = Schreiner’s urea.

With these data in hand, further optimization varying the temperature, solvent, and reaction time provided reaction conditions to access the regioisomeric α- and β-fluoroamines **2a** and **3a** from **1a** in high regioselectivity and high yield from CsF. The β-fluoroamine **3a** was also within reach with KF under HBPTC with catalytic Schreiner’s urea (SU) (Scheme 2).^17^

**Figure 2:**
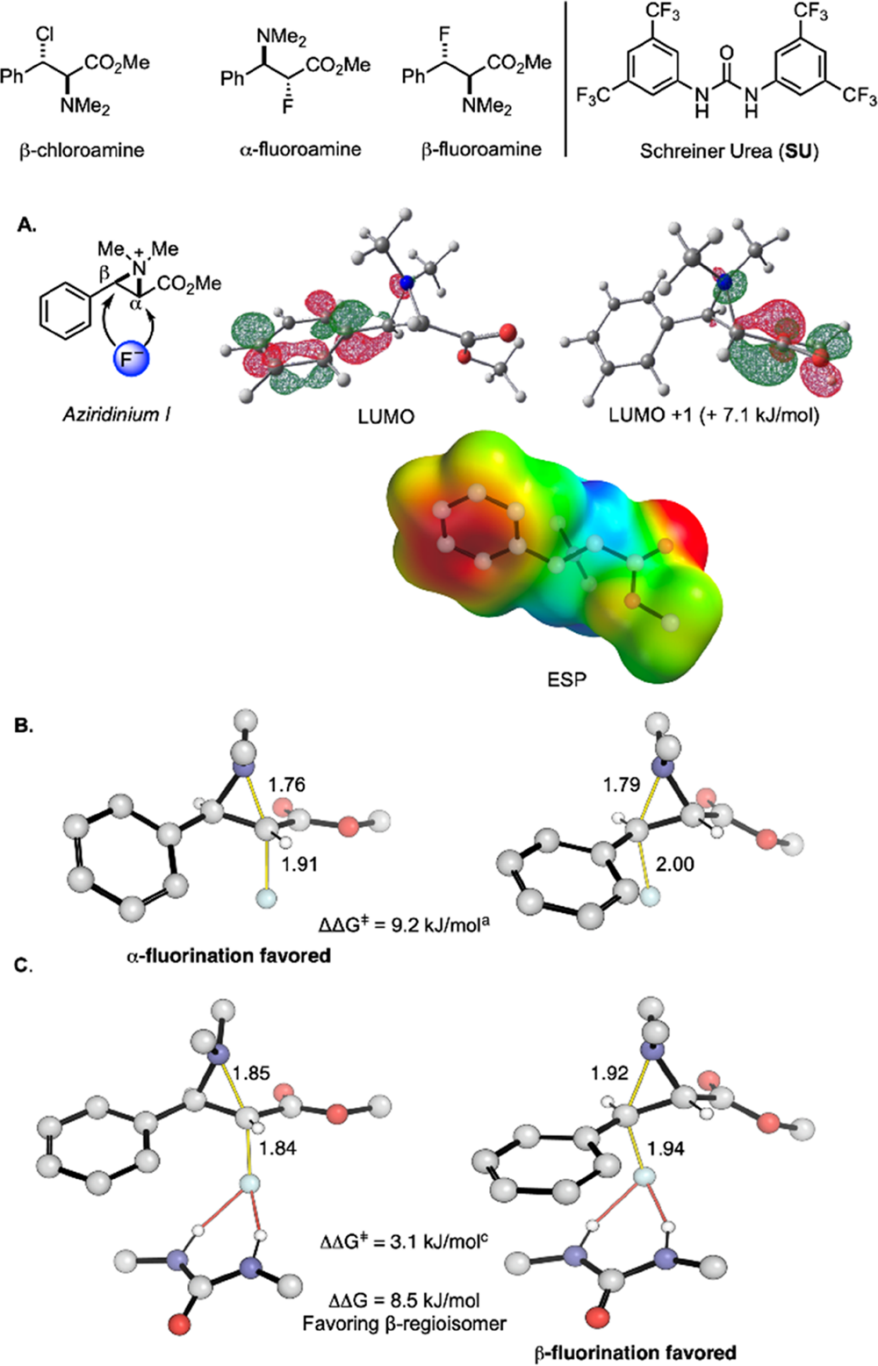
Computed ground and TS structures for aziridinium fluorination (B97X-D3/(ma)-def2-TZVPP//M06-2X/def2-SVP(TZVPPD) with CPCM DCM solvation).^13^ (A) Aziridinium LUMO, LUMO + 1 and ESP plots. (B) TSs with free fluoride. (C) TSs with SU-bound fluoride (SU aryl rings abbreviated for clarity).

**Figure 3:**
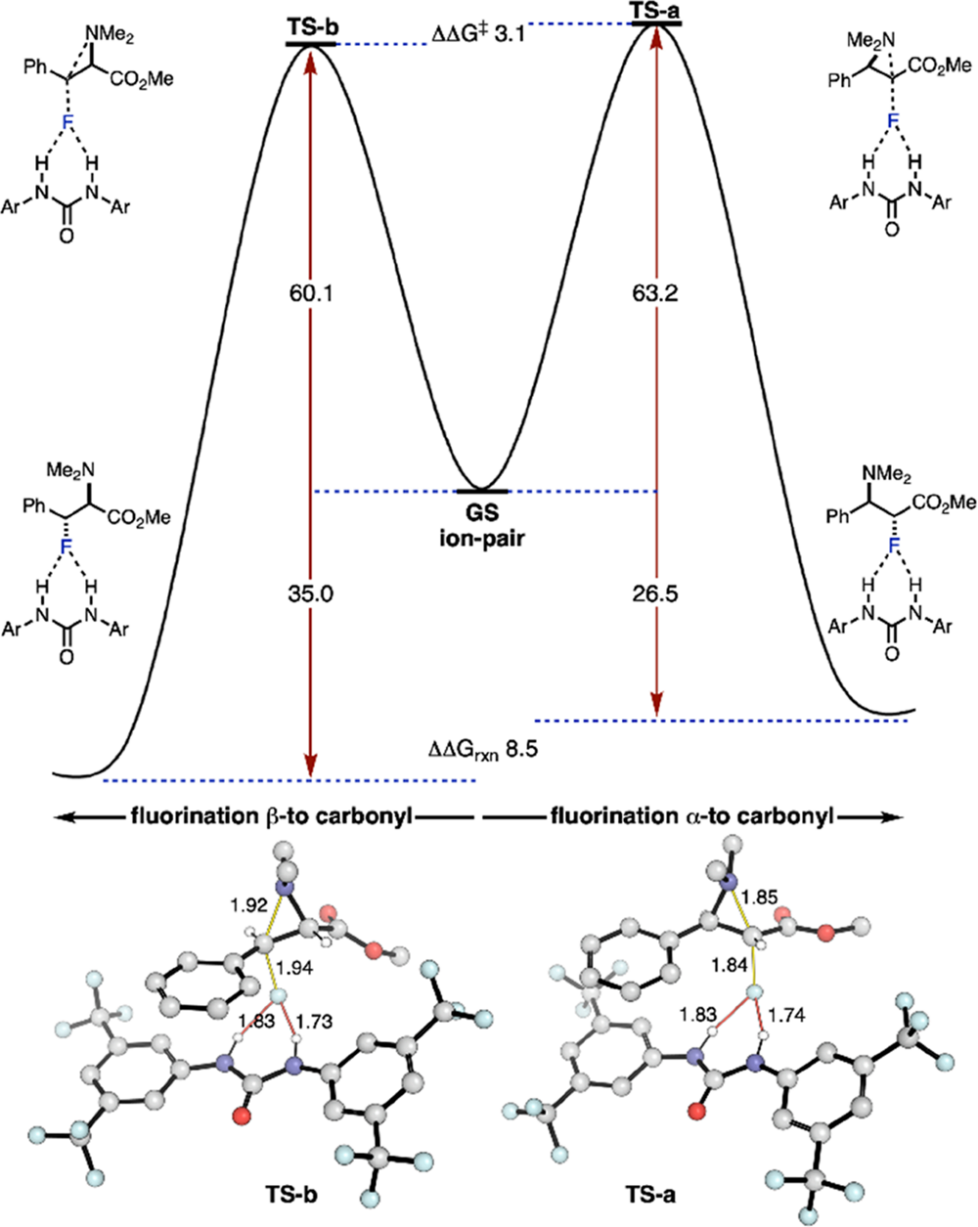
Computed PES for defluorination/fluorination sequence enabling the regiochemical editing of fluoroamines. (ωB97X-D3/(ma)-def2-TZVPP)//M06-2X/def2-SVP(TZVPPD).^13^ Ar = 3,5-bis(trifluoromethyl)phenyl.

Computational studies gave further insight (Figure 2). Data secured with *N*-allyl modeled by *N*-methyl groups indicated that the α-position, whose attack is favored in the absence of urea, is characterized by a more positive electrostatic potential (Figure 2A). In contrast, at the β-position, which is the preferred site of attack with catalytic SU, there is greater contribution of the antibonding σ_C-N_* orbital to the LUMO (the LUMO + 1 has contribution from the σ_C-N_* to the α-position). LUMO and LUMO + 1 energies are very similar, rendering the application of FMO theory to understand regioselectivity challenging. The TS geometries for free fluoride are relatively early, with longer C–F/shorter C–N distances compared to the fluoride:HBD complex (Figure 2B). Greater electrostatic attraction at the α-carbon steers the regiochemical preference for this position. With fluoride bound to the SU catalyst, its negative charge and nucleophilicity are attenuated, leading to later TS geometries with shorter C–F/longer C–N distances (Figure 2C). Electrostatic interactions are less significant, and substrate distortion energies become more critical (vide infra). The β-fluoroamine is favored thermodynamically by 8.5 kJ/mol over the α-regioisomer. The potential energy surface (PES) for SU-catalyzed conversion of **2a** into **3a** was also studied computationally (Figure 3). At higher temperatures, **3a** is the major regioisomer formed, which is expected as it is thermodynamically more stable than **2a**. The fluorination TS leading to this product is also more favorable than that leading to the α-regioisomer by 3.1 kJ/mol, consistent with kinetically controlled regioselectivity for the β-regioisomer in the presence of SU catalyst.

**Figure 4:**
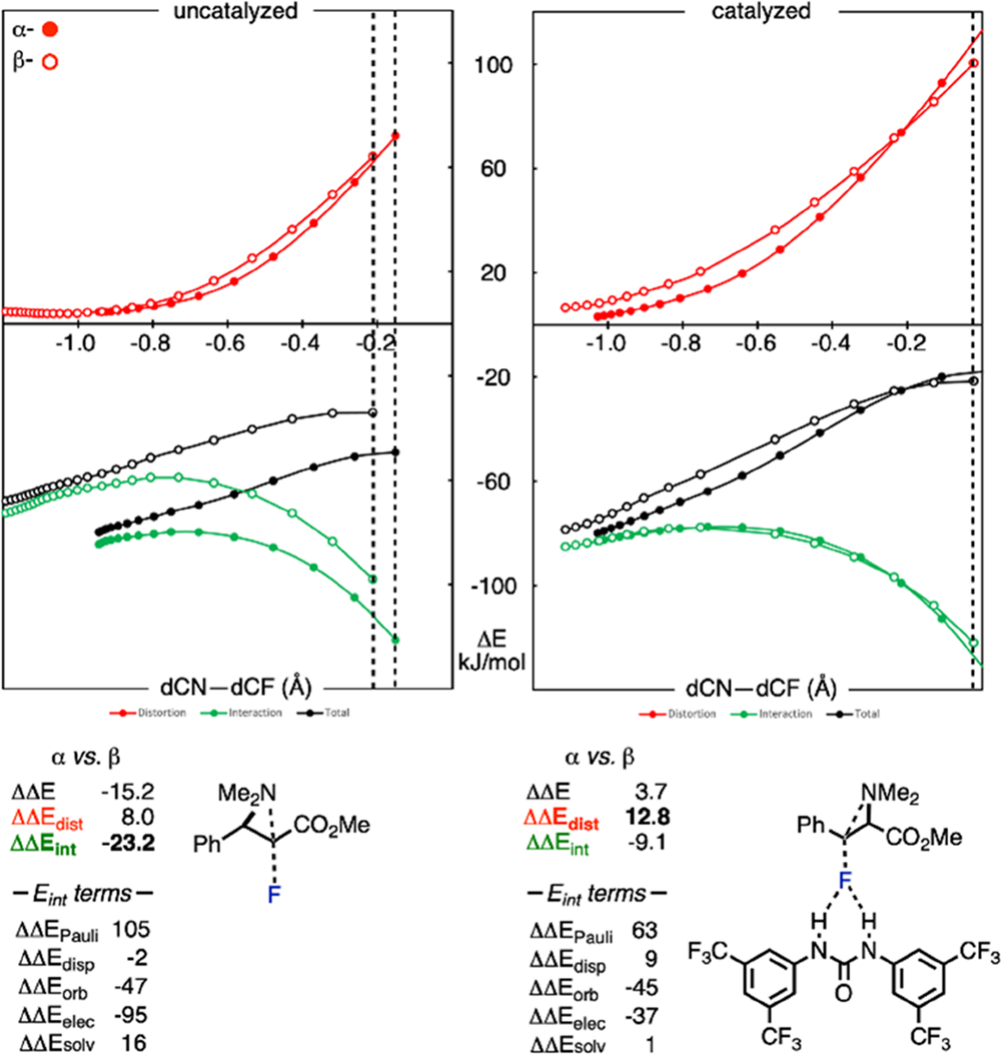
Distortion-interaction activation-strain analysis for aziridinium fluorination with and without Schreiner’s urea (ωB97X-D3/def2-TZVPP with CPCM solvation).^13^ Filled circles = α-pathway; empty circles = β-pathway. TS positions are shown with dashed lines.

To gain further quantitative insight into the origins of the switch in regioselectivity in the presence of the Schreiner’s urea, a distortion-interaction activation-strain analysis was performed along the fluorination intrinsic reaction coordinate (IRC) with and without catalyst (Figure 4).^18^ The difference between breaking (C–N) and forming (C–F) bonds was used as the reduced reaction coordinate. Further decomposition of the interaction energies into Pauli repulsion, orbital (polarization and charge transfer), electrostatic, and dispersion interactions was performed using the absolutely localized molecular orbital-energy decomposition analysis (ALMO-EDA).^19^ Selectivity in the uncatalyzed fluorination reaction is dictated by the difference in interaction energies (green curves). The approach of free fluoride at the α-position has a more favorable interaction energy by 23.2 kJ/mol, the result of a large stabilizing electrostatic attraction between nucleophile and electrophile for the attack at this position. This is consistent with the ESP map (Figure 2A), showing a more positive value around the α-carbon. Since the TS geometries are relatively early, substrate distortion terms are small. Overall, these results are consistent with electrostatically controlled selectivity with free fluoride. In contrast, when fluoride is bound to urea, distortion energy terms become more important: the TS is later, and so substrate distortion is larger. Interestingly, the distortion energy of the α-TS grows more sharply than the β-TS as the TS is approached; we ascribe this to the destabilizing accumulation of positive charge in the substrate α- to an electron-withdrawing ester group. At the same time, the electrostatic preference for the α-position is reduced (from 95 to 37 kJ/mol), presumably since F now bears less negative charge (−0.64, Figure 1) compared to unbound fluoride. As a result, the difference in distortion energies dictates the regioselectivity. This term is mainly derived from the aziridinium electrophile, where lengthening of the β-C–N bond is less costly than the α-C–N bond, presumably due to the ability of the adjacent phenyl group to stabilize the developing positive charge in the distorted geometry.

### Generalization and Asymmetric Regiodivergent Fluorination with an (*S*)-BINAM-Derived Urea Catalyst

**Scheme 3:**
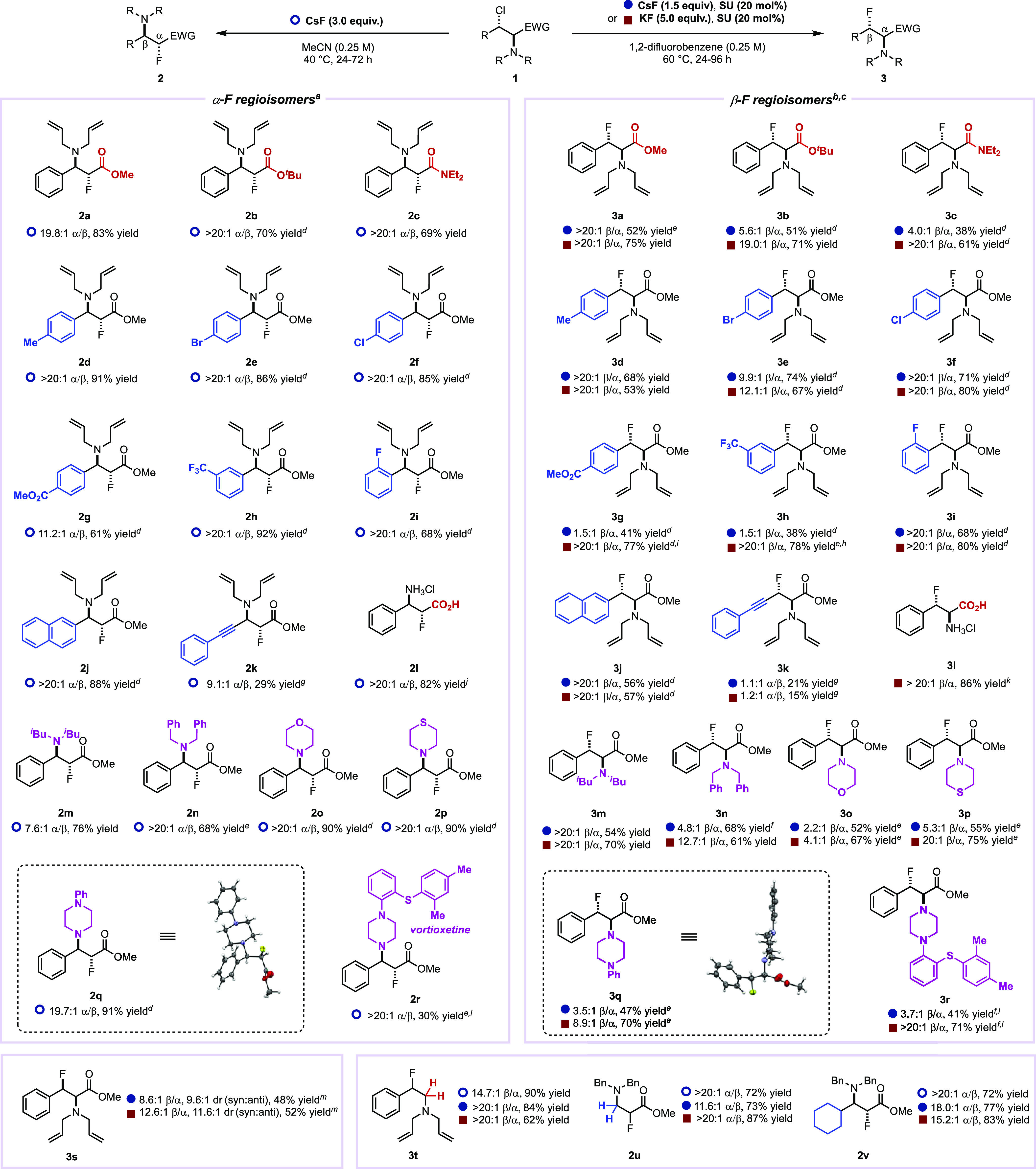
Scope of α- and β-Fluorination of β-Chloroamines,,,,,,,,,,, Reaction conditions for α-F regioisomers: 0.25 mmol of β-chloroamine and CsF (3.0 equiv) were stirred in MeCN (1.0 mL, 0.25 M) at 1200 rpm for 24 h at 40 °C. Reaction conditions for β-F regioisomers: (b) 0.25 mmol of β-chloroamine, SU (20 mol %), and KF (5.0 equiv) or (c) 0.25 mmol of β-chloroamine, SU (20 mol %), and CsF (1.5 equiv) were stirred in 1,2-DFB (1.0 mL, 0.25 M) at 1200 rpm for 24 h at 60 °C. Reaction was run for 48 h. Reaction was run for 72 h. Reaction was run for 96 h. Reaction at 23 °C. Reaction at 70 °C. Reaction at 80 °C. Obtained from **2b**.^17^ Obtained from **3b**.^17^ Reaction at 0.15 mmol scale. Yield determined by quantitative ^1^H NMR, using 1,3,5-trimethoxybenzene as an internal standard.

With the optimized experimental protocols in hand, we sought to evaluate the scope of the regiodivergent fluorination reaction without and with SU catalyst (Scheme 3).^20^

β-Chloroamines bearing various aryl, amino, ester, and amide substituents were first subjected to α-fluorination with CsF in MeCN at 40 °C. The reaction tolerates *tert*-butyl ester instead of methyl ester, providing **2b**, which was transformed into the α-fluoro-β-amino acid **2l**. Most aryl groups investigated afforded the α-regioisomers in a high α:β ratio (>20:1), but we noted that the regiopreference observed with **1g** featuring the electron-withdrawing methyl ester group at the para position was less pronounced (r.r. = 11.2:1, α:β). The aryl group can be replaced by propargyl albeit affording **2k** in lower yield and α/β ratio (9.1:1). Gratifyingly, the reaction is compatible with several amino groups including motifs frequently seen in medicinal chemistry such as, for example, piperidine and (thio)morpholines. Next, we subjected the same library of β-chloroamines to β-fluorination using either KF or CsF in the presence of the Schreiner’s urea (SU) catalyst. Higher β:α ratios were obtained with KF compared to CsF. This result was expected due to the lack of background reactivity of KF in the absence of SU catalyst under otherwise similar reaction conditions. Higher yields of isolated products were also often obtained with KF versus CsF. The reaction tolerates all substrates **1a–r** but underperformed for the propargylic fluoride **3k**, which was obtained as a mixture of regioisomers. When the electron-withdrawing ability of the aromatic substituent was increased, a higher temperature was required to achieve high regioselectivity (for **3g**, for instance). This catalytic reaction gives high regioselectivity with mildly electron-donating (**3d**) and halogen (**3e–3f**) substituents, although a longer reaction time was also required in the latter cases. Tertiary amines, including the bulkier diisobutylamine (**3m**), saturated heterocycles (**3o–3q**), and a biologically active motif (**3r**), were well tolerated. The *cis*-aziridinium precursor gave **3s** in good regioselectivity (r.r. = 12.6:1, β:α) along with a detectable amount of the *anti*-diastereomer. A substrate lacking the ester motif resulted in regioselective benzylic fluorination (**3t**) under all reaction conditions. Conversely, when a serine-derived starting material lacking the aryl group was employed (**1u**), α-fluorination was invariably observed. A similar α-regiopreference was observed with a cyclohexyl substituent (**3v**). This change in regioselectivity is consistent with the LUMO coefficient localized on the carbon α to the ester for these substrates.^17^

**Scheme 4:**
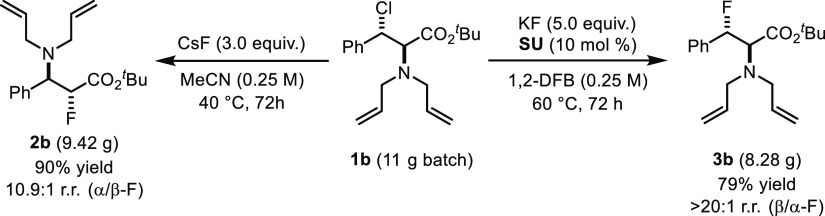
Multigram Synthesis of **2b** and **3b**

Multigram quantities of both regioisomers **2b** and **3b** were obtained from β-chloroamine **1b** (Scheme 4). Starting with 11 g of **1b**, 8.28 g of β-fluoroamine **3b** (r.r. > 20:1) was isolated when the reaction was performed with KF (5 equiv) and SU catalyst (10 mol %) at 60 °C. A reaction time of 72 h was necessary to reach 79% yield. The fluorination of 11 g of **1b** with CsF (3 equiv) in MeCN at 40 °C for 72 h afforded 9.42 g of α-fluoroamine **2b** (r.r. > 10:1).

**Scheme 5:**
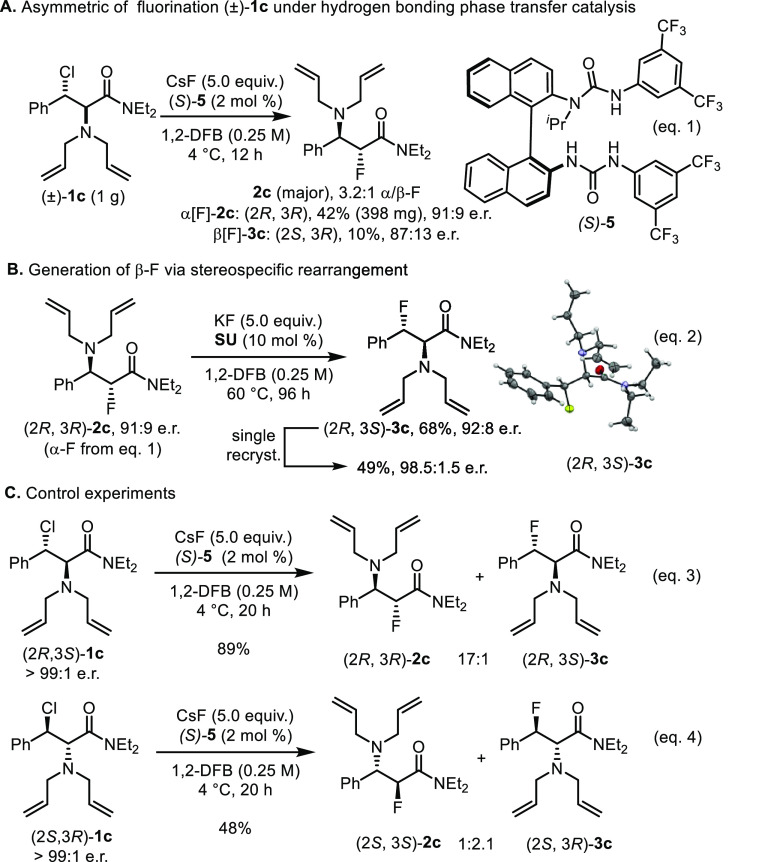
Regiocontrolled Fluorination under Asymmetric Hydrogen Bonding Phase Transfer Catalysis

Having demonstrated regiodivergence under hydrogen bonding catalysis, we considered the BINAM-derived (*S*)-*bis*-urea catalyst **5** to prepare enantioenriched fluoroamines. Early experimentations identified the β-chloro-α-amino amide (±)-**1c** as a promising substrate for this study.^17^ The reaction of (±)-**1c** with CsF in 1,2-difluorobenzene at 4 °C in the presence of 2 mol % of (*S*)-**5** gave α[F]-**2c** as the preferential regioisomer (r.r. = 3.2:1, α:β) in enantioenriched form (91:9 e.r.). The minor regioisomer was also formed with significant enantioenrichment (87:13 e.r.). (Scheme 5, eq 1). Furthermore, when enantioenriched α[F]-**2c** was subjected to stereospecific fluoride migration in the presence of KF and the Schreiner’s urea (Scheme 5, eq 2), the β-fluorinated product β[F]-**3c** was formed with no erosion of enantiomeric ratio; to proceed, this rearrangement required elevated temperature (60 °C). Control experiments with enantiopure (2*R*,3*S*)-**1c** and (2*S*,3*R*)-**1c** gave useful insight.^21^ The reaction of (2*R*,3*S*)-**1c** (>99:1 e.r.) with CsF in the presence of 2 mol % of the BINAM-derived (*S*)-*bis*-urea catalyst **5** led to regioisomers **2c** and **3c** with **2c** being largely predominant (r.r. = 17:1, α:β) (Scheme 5, eq 3). Enantiomer (2*R*,3*S*)-**1c** under the same reaction conditions was consumed less rapidly and led to two regioisomers with a ratio now favoring β-fluorination (r.r. = 1:2.1, α:β) (Scheme 5, eq 4). These results indicate that the regiopreference of the catalyst allows divergent reaction pathways for each enantiomer with fluorination proceeding with unequal rates. The exact origin of the regiopreference of this catalyst remains to be established. It is conceivable that the chiral urea–fluoride complex binds the two enantiomers of the aziridinium ion in distinct orientations during the activation process, and each of the diastereomeric complexes has a different electrophilic carbon favorably exposed for attack by fluoride.

## Conclusions

This work has unveiled a novel approach to invert the sense of regiocontrol for fluorination with alkali metal fluoride through a modulation of charge density on fluoride with a hydrogen-bond donor phase transfer catalyst. Combined with a novel HBD-enabled regiochemical editing process consisting of an equilibration mechanism based on urea-catalyzed C–F activation followed by fluoride rebound, high regioisomeric ratios in favor of either regioisomer are within reach using an alkali metal fluoride as fluorination reagent. Moreover, the synthesis of regio- and enantioenriched α- and β-fluoroamines under asymmetric hydrogen bonding catalysis with a BINAM-derived *bis*-urea catalyst offers new opportunities to expand the scope of synthetic strategies available to access fluorinated molecules. More generally, this catalyst-controlled approach to alter regiochemical preference may be applicable to many electrophiles and charged nucleophiles other than alkali metal fluoride.

## Supporting Information Available

Experimental details, characterization data, NMR spectra, and full computational details (PDF)
Computational data_xyz_coordinates (ZIP)
Computational data_MP2_alpha_TS.xyz (XYZ)
Computational data_MP2_beta_TS.xyz (XYZ)

## Supplementary Material

ja3c01303_si_002.zip

ja3c01303_si_003.xyz

ja3c01303_si_004.xyz
