# Supplementary material for: Regiodivergent Nucleophilic Fluorination under Hydrogen Bonding Catalysis: A Computational and Experimental Study
Source: J Am Chem Soc. 2023 Apr 20;145(17):9708–17. doi: 10.1021/jacs.3c01303 (PMC10161234; doi:10.1021/jacs.3c01303)
Supplement: Supplementary file 1 — ja3c01303_si_001.pdf [file ja3c01303_si_001.pdf]

Regiodivergent Nucleophilic Fluorination under Hydrogen Bonding Catalysis: A Computational and Experimental Study

Matthew A. Horwitz,[1#*] Alexander B. Dürr,[1#*] Konstantinos Afratis,[1#] Zijun Chen,[1#] Julia Soika,[1] Kirsten E. Christensen,[1] Makoto Fushimi,[2] Robert S. Paton,[3*] Véronique Gouverneur[1*]

[1]Chemistry Research Laboratory, University of Oxford, 12 Mansfield Road, Oxford, OX1 3TA, U.K. [2]Takeda Pharmaceutical Company Limited, 26-1, Muraoka-Higashi 2-Chome, Fujisawa, Kanagawa 251-8555, Japan. [3]Department of Chemistry, Colorado State University, Fort Collins, CO 80528, USA

**Table of Contents**	**Page**
General information	S2
Optimization Data for α-Fluorination with CsF	S3
Optimization Data for β-Fluorination under HBPTC	S4-S7
Catalyst Synthesis	S8
Synthesis of Epoxides	S8
General Procedure for Opening of Epoxides with Secondary Amines	S8-S9
Characterization Data for Amino Alcohols	S9-S16
General Procedure for Chlorination (or Mesylation) of Amino Alcohols	S16
Characterization Data for Amino Chlorides and Mesylates	S17-S23
General Procedure for α-Fluorination with CsF	S24
Characterization Data for α–Fluoro Amines	S24-S30
General Procedure for β-Fluorination under HBTPC	S31
Characterization Data for β–Fluoro Amines	S32-S42
Synthesis of Amino Acid HCl Salts	S42-43
Enantiospecific Synthesis of α- and β–Fluoro Esters	S44-S47
Overview of the Synthesis of Enantioenriched Epoxy Amides via Shibasaki Epoxidation	S47-S48
Catalytic Asymmetric Epoxidation of β-Aryl Substituted α,β-Unsaturated Amides	S48
Compounds Derived from Enantioenriched Epoxy Amides	S48-S49
Asymmetric a-Fluorination of Racemic Amino Chloride Substrates	S50-S52
Investigation of Regiochemical Preference of Each Enantiomer of Substrate	S52-S53
Computational Methods	S54
Method Validation	S54-55
The relation of charge and kinetic selectivity	S55-S56
The relative energy differences of the unoccupied molecular orbitals on the α and β-position of the aziridinium intermediate	S56-S57
The Potential Energy Surface	S57-S58
Distortion-Interaction / Activation-Strain analysis	S58-S59
Tabulated energies of computed structures	S59-S61
Copies of HPLC Traces	S62-S75
X-ray analysis	S76-S78
NMR spectra of new compounds	S79-S182
References	S183-S185

General Information

Unless otherwise stated, all reagents were purchased from commercial suppliers (Sigma-Aldrich, Alfa Aesar, Fluorochem and Apollo Scientific) and used without further purification. Unless otherwise stated, solvents were used without prior drying/degassing. KF (99.9% trace metal basis from Alfa Aesar) was used as provided by the supplier (fine powder) and used without pre-drying. CsF (99.9% trace metal basis from Sigma-Aldrich) was ground prior to the reaction and used without pre-drying. Reactions were monitored by thin layer chromatography (TLC) on silica gel pre-coated aluminium sheets (Merck Kieselgel 60 F254 plates). Visualization was accomplished by irradiation with UV light at 254 nm, and/or phosphomolybdic acid (PMA) stain, and/or cerium ammonium molybdate (CAM) stain, and/or permanganate stain. Flash column chromatography (FCC) was performed on Merck silica gel (60, particle size 0.040-0.063 mm). All NMR spectra were recorded on Bruker AVIIIHD 400, AVIIIHD 500 or VII 500. ^{1}H and ^{13}C NMR spectral data are reported as chemical shifts (δ) in parts per million (ppm) relative to the solvent peak using the Bruker internal referencing procedure (edlock). ^{19}F NMR spectra are referenced relative to $CFCl_3$. Data are reported as follows: chemical shift, multiplicity (s = singlet, d = doublet, t = triplet, q = quartet, br = broad, m = multiplet), coupling constants (Hz) and integration. NMR spectra were processed with Topspin 3.6. Yields refer to isolated yields of compounds unless otherwise specified; compounds considered isolated are >95% pure. Yields and regioselectivities reported in Supporting Information are single trials, not averages of multiple trials, so they may not exactly match the values reported in the main text. High resolution mass spectra (HRMS, m/z) were recorded on a Thermo Exactive mass spectrometer equipped with Waters Acquity liquid chromatography system using either the heated electrospray (HESI-II) probe for positive electrospray ionization (ESI+) or the atmospheric pressure chemical ionization (APCI) probe. Infrared spectra were recorded as the neat compound or in solution using a Bruker tensor 27 FT-IR spectrometer. Absorptions are reported in wavenumber (cm^{-1}). Melting points of solids were measured on a Griffin apparatus and are uncorrected.

Optimization Data for α-Fluorination with CsF

Table S1: Solvent and Concentration Screening

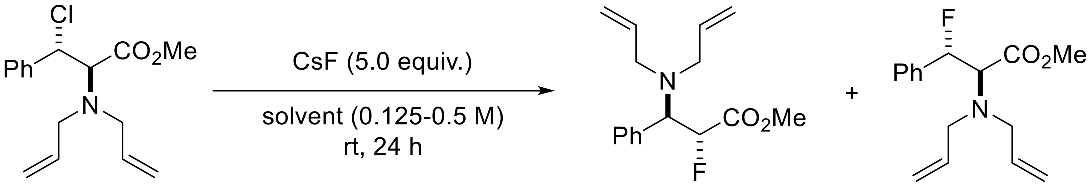

1a **2a** **3a**

Entry	Solvent	Concentration (M)	NMR Yield[a]	r.r.[b] (2a:3a)
1	1,2-difluorobenzene	0.25	4%	-
**2**	**CH_3CN**	**0.25**	**79%**	**18.2:1**
3	Tetrahydrofuran	0.25	0%	-
4	CH_2Cl_2	0.25	34%	>20:1
5	EtOAc	0.25	0%	-
6	Toluene	0.25	0%	-
7	Acetone	0.25	8%	>10:1
8	Dimethylformamide	0.25	28%	>20:1
9	CH_3CN	0.125	84%	18.9:1
10	CH_3CN	0.50	77%	11.4:1

Reaction conditions: 0.1 mmol of amino chloride and CsF (5.0 equiv.) were stirred in commercial solvent listed above at 1200 rpm for 24 h at a distance of 2 cm from a stirring plate. Worked up by passing through a short plug of SiO_2 with diethyl ether, followed by concentration *in vacuo*. [a]Determined by ^1H NMR using 1,3,5-trimethoxybenzene as an internal standard. [b]r.r. = regioisomeric ratio, determined by ^1H NMR of crude mixture.

Table S2: Temperature, Nucleophile Equivalency, and Reaction Time Screening

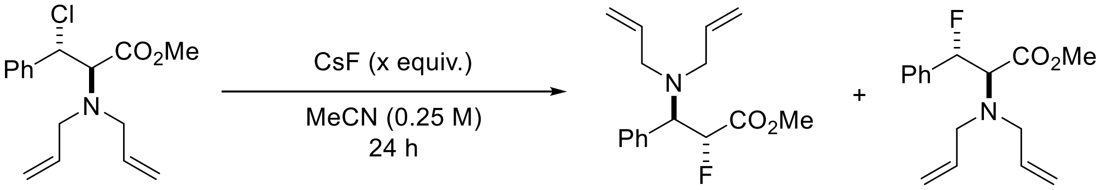

1a **2a** **3a**

Entry	Time (h)	CsF (x equiv.)	Temperature (°C)	NMR Yield[a]	r.r.[b] (2a:3a)
1	24	5.0	23	79%	18.2:1
2	24	5.0	40	>95%	17.5:1
3	24	5.0	50	>95%	14.8:1
**4**	**24**	**3.0**	**40**	**90%**	**17.2:1**
5	24	1.5	40	85%	5.2:1

Reaction conditions: 0.1 mmol of amino chloride and CsF (5.0 equiv.) were stirred in commercial solvent listed above at 1200 rpm for 24 h at a distance of 2 cm from a stirring plate. Worked up by passing through a short plug of SiO_2 with diethyl ether, followed by concentration *in vacuo*. [a]Determined by ^1H NMR using 1,3,5-trimethoxybenzene as an internal standard. [b]r.r. = regioisomeric ratio, determined by ^1H NMR of crude mixture.

Optimization Data for β-Fluorination under HBPTC

Table S3: Solvent and Concentration Screening

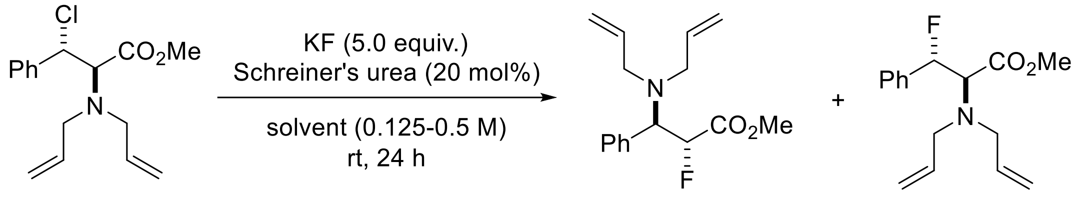

1a **2a** **3a**

Entry	Solvent	Concentration (M)	NMR Yield[a]	r.r.[b] (2a:3a)
**1**	**1,2-difluorobenzene**	**0.25**	**>95%**	**1:4.0**
2	CH_3CN	0.25	>95%	1:3.1
3	CH_3CN/H_2O (1:1)[c]	0.25	21%	1:4.7
4	α,α,α-trifluorotoluene	0.25	>95%	1:2.9
5	CH_2Cl_2	0.25	>95%	1:2.8
6	$CHCl_3$	0.25	>95%	1:3.8
7	1,2,3-trifluorobenzene	0.25	>95%	1:4.4
8	Toluene	0.25	73%	1:1.5
9	Tetrahydrofuran	0.25	70%	1:1.9
10	1,2-difluorobenzene	0.125	>95%	1:3.4
11	1,2-difluorobenzene	0.50	>95%	1:4.1

Reaction conditions: 0.1 mmol of amino chloride, Schreiner's urea (20 mol %), and KF (5.0 equiv.) were stirred in commercial solvent listed above at 1200 rpm for 24 h at a distance of 2 cm from a stirring plate. Worked up by passing through a short plug of SiO_2 with diethyl ether, followed by concentration *in vacuo*. [a]Determined by ^1H NMR using 1,3,5-trimethoxybenzene as an internal standard. [b]r.r. = regioisomeric ratio, determined by ^1H NMR of crude mixture. [c] No catalyst used in this trial.

Table S4: Catalyst Loading Screening

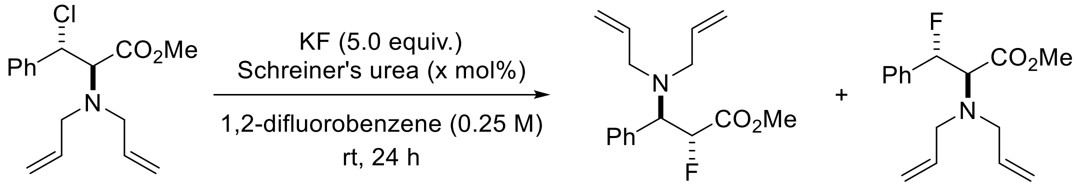

1a **2a** **3a**

Entry	Catalyst (mol %)	NMR Yield[a]	r.r.[b] (2a:3a)
1	0	0%	-
2	5	>95%	1:2.4
3	10	>95%	1:3.1
**4**	**20**	**>95%**	**1:4.0**
5	50	>95%	1:3.9
6	100	>95%	1:4.0

Reaction conditions: 0.1 mmol of amino chloride, Schreiner's urea (x mol %), and KF (5.0 equiv.) were stirred in 1,2-difluorobenzene at 1200 rpm for 24 h at a distance of 2 cm from a stirring plate. Worked up by passing through a short plug of SiO_2 with diethyl ether, followed by concentration *in vacuo*. [a]Determined by ^1H NMR using 1,3,5-trimethoxybenzene as an internal standard. [b]r.r. = regioisomeric ratio, determined by ^1H NMR of crude mixture.

Table S5: Fluoride Salt (Identity and Equivalents) Screening

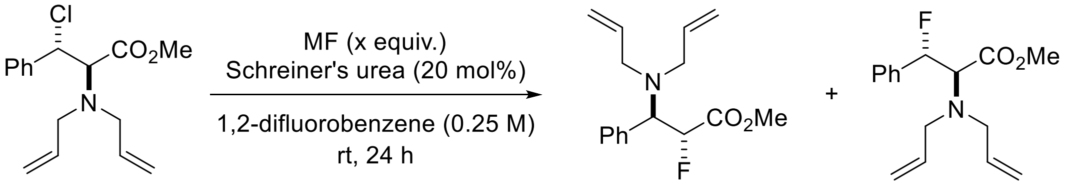

1a 2a 3a

Entry	MF (x equiv.)	Catalyst (mol %)	NMR Yield[a]	r.r.[b] (2a:3a)
1	CsF (5.0 equiv.)	20	70%	1:2.4
2	CsF (5.0 equiv.)	0	4%	>10:1
3	LiF (5.0 equiv.)	20	0%	-
4	AgF (5.0 equiv.)	20	>95%	1:3.5
5	AgF (5.0 equiv.)	0	93%	1:5.3
6	NaF (5.0 equiv.)	20	0%	-
7	NH_4F (5.0 equiv.)	20	26%	1:4.5
8	NH_4F (5.0 equiv.)	0	0%	-
9	NBu_4F (6.0 equiv.)[c]	0	20%	1.3:1

Reaction conditions: 0.1 mmol of amino chloride, Schreiner's urea (20 mol %, if applicable), and fluoride salt listed above were stirred in 1,2-difluorobenzene (0.25M) at 1200 rpm for 24 h at a distance of 2 cm from a stirring plate. Worked up by passing through a short plug of SiO_2 with diethyl ether, followed by concentration *in vacuo*. [a]Determined by ^1H NMR using 1,3,5-trimethoxybenzene as an internal standard. [b]r.r. = regioisomeric ratio, determined by ^1H NMR of crude mixture. [c] 6.0 equiv. was calculated on an anhydrous basis. Most starting material was destroyed in reaction (only 5% remained after the reaction).

Table S6: Effect of Temperature

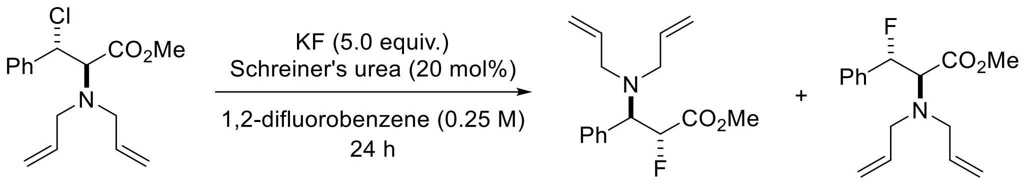

1a 2a 3a

Entry	Temperature (°C)	NMR Yield[a]	r.r.[b] (2a:3a)
1	4	>95%	1:3.2
2	23	>95%	1:4.0
3	40	>95%	1:5.4
4	50	>95%	1:8.1
**5**	**60**	**>95%**	**<1:20**
6	70	28%	<1:20
7[c]	60	>95%	1:6.9
8[d]	60	0%	-

Reaction conditions: 0.1 mmol of amino chloride, Schreiner's urea (20 mol %), and KF (1.5 equiv.) were stirred in 1,2-difluorobenzene at 1200 rpm for 24 h at a distance of 2 cm from a stirring plate. An oil bath was using to achieve temperatures above room temperature. A cold room at 4 °C was used to run a low temperature trial. Worked up by passing through a short plug of SiO_2 with diethyl ether, followed by concentration *in vacuo*. [a]Determined by ^1H NMR using 1,3,5-trimethoxybenzene as an internal standard. [b]r.r. = regioisomeric ratio, determined by ^1H NMR of crude mixture. [c]Reaction was run for 6 h instead of 24 h. [d]Reaction was performed without Schreiner's urea.

Table S7: Varying Cation Identity at Elevated Temperature

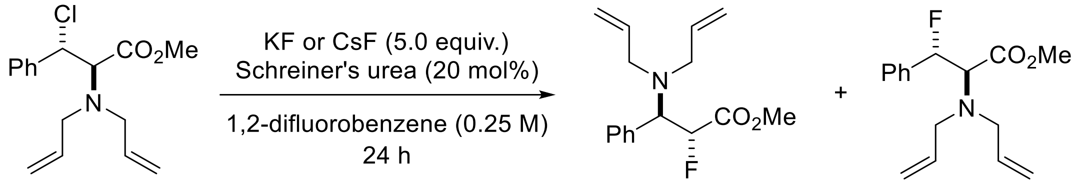

1a **2a** **3a**

Entry	MF (x equiv.)	Temperature (°C)	NMR Yield[a]	r.r.[b] (2a:3a)
1	KF (5.0 equiv.)	23	>95%	1:4.0
2	KF (5.0 equiv.)	60	>95%	<1:20
3	CsF (5.0 equiv.)	23	70%	1:2.4
4	CsF (5.0 equiv.)	60	>95%	1:4.8
5	CsF (1.5 equiv.)	60	83%	1:7.4
6[c]	CsF (1.5 equiv.)	60	80%	<1:20

Reaction conditions: 0.1 mmol of amino chloride, Schreiner's urea (20 mol %), and KF or CsF (1.5 equiv.) were stirred in 1,2-difluorobenzene at 1200 rpm for 24 h at a distance of 2 cm from a stirring plate. An oil bath was using to achieve temperatures above room temperature. Worked up by passing through a short plug of SiO_2 with diethyl ether, followed by concentration *in vacuo*. [a]Determined by ^1H NMR using 1,3,5-trimethoxybenzene as an internal standard. [b]r.r. = regioisomeric ratio, determined by ^1H NMR of crude mixture. [c]Reaction was allowed to proceed for 72 h.

Table S8: Rearrangement of α-Fluoro-β-amine Ester to β-Fluoro-α-amino Ester

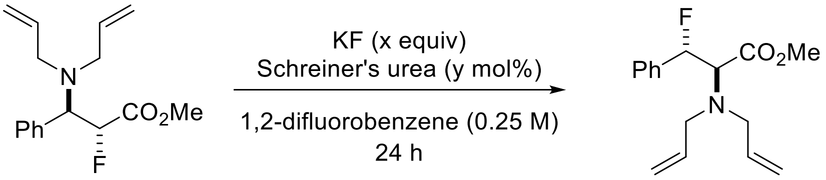

2a **3a**

Entry	Schreiner's urea (y mol %)	KF (x equiv.)	Temperature (°C)	NMR Yield[a] (of α-F + β-F)	r.r.[b] (2a:3a)
1	20	5.0	60	>95%	1:14.6
2	0	5.0	60	>95%	only **2a**
3	20	0	60	59%	1:6.8
4	20	5.0	50	>95%	1:1.4
5	20	5.0	40	>95%	2.9:1
6	20	5.0	23	>95%	13.4:1

Reaction conditions: 0.1 mmol of amino chloride, Schreiner's urea (y mol %), and KF (x equiv.) were stirred in 1,2-difluorobenzene at 1200 rpm for 24 h at a distance of 2 cm from a stirring plate. An oil bath was using to achieve temperatures above room temperature. Worked up by passing through a short plug of SiO_2 with diethyl ether, followed by concentration *in vacuo*. [a]Determined by ^1H NMR using 1,3,5-trimethoxybenzene as an internal standard. [b]r.r. = regioisomeric ratio, determined by ^1H NMR of crude mixture.

Table S9: Effect of HFIP and Stoichiometric amount of Schreiner's urea

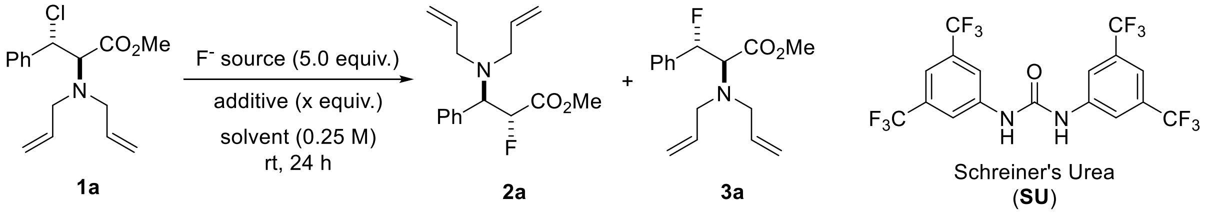

Entry	Fluoride source	Solvent	Additive (x equiv.)	NMR Yield[a]	r.r.[b] (2a:3a)
1	CsF	DCM	-	34%	> 20:1
2	CsF	DCM	SU (0.2)	>95%	1:2.6
3	CsF	DCM	HFIP (0.2)	17%	7.5:1
4	KF	DCM	-	0%	-
5	KF	DCM	SU (0.2)	>95%	1:2.8
6	KF	DCM	HFIP (0.2)	<2%	-
7	TBAF[c]	DCM	SU (1.5)	48%	1:1.7
8	TBAF[c]	DCM	SU (3.0)	90%	1:2.9
9	CsF	1,2-DFB	-	4%	**α** only
10	CsF	1,2-DFB	SU (0.2)	70%	1:2.4
11	CsF	1,2-DFB	HFIP (0.2)	0%	-
12	KF	1,2-DFB	-	0%	-
13	KF	1,2-DFB	SU (0.2)	>95%	1:4
14	KF	1,2-DFB	HFIP (0.2)	0%	-
15	TBAF[c]	1,2-DFB	SU (1.5)	22%	1:2.1
16	TBAF[c]	1,2-DFB	SU (3.0)	78%	1:2.1
17	CsF	HFIP	-	<1%	-
18	CsF	HFIP	SU (0.2)	<1%	-
19	KF	HFIP	-	<1%	-
20	KF	HFIP	SU (0.2)	<1%	-
21	TBAF	HFIP	-	0%	-
22	$NEt_3 \cdot 3HF$	DCM	-	<10%	1:15
23	TBAF	DCM	-	20%	1.3:1

Reaction conditions: 0.1 mmol of amino chloride, fluoride sources (5.0 equiv.) and additives (0 or 0.2 equiv.) were stirred in commercial solvent listed above at 1200 rpm for 24 h at a distance of 2 cm from a stirring plate. Worked up by passing through a short plug of SiO_2 with diethyl ether, followed by concentration *in vacuo*. [a]Determined by ^{1}H NMR using 1,3,5-trimethoxybenzene as an internal standard or ^{19}F NMR using 4-fluoroanisole as an internal standard. [b]r.r. = regioisomeric ratio, determined by ^{1}H and ^{19}F NMR of crude mixture. SU = Schreiner's urea [c]1.5 equiv. of TBAF.

Catalyst Synthesis

(*S*)-BINAM bis-urea (see main text) was synthesized according to literature procedures.[1,2] 1,3-bis(3,5-bis(trifluoromethyl)phenyl)urea ("Schreiner's urea") is commercially available, but was synthesized on large scale at low cost.[3]

Synthesis of Epoxides

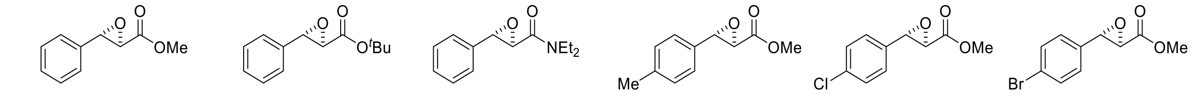

E1 **E2** **E3** **E4** **E5** **E6**

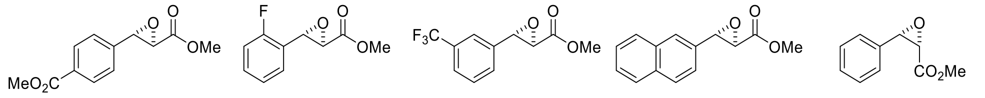

E7 **E8** **E9** **E10** **E11**

Epoxides **E1**[4], **E2**[5], **E3**[6], **E4**[7], **E5**[8], **E6**[8], **E7**[9], **E8**[10], **E10**[9], and **E11**[11] have previously been reported and were made according to literature procedures. **E9** was not previously reported.

(±)-*trans*-methyl 3-(3-(trifluoromethyl)phenyl)oxirane-2-carboxylate (E9)

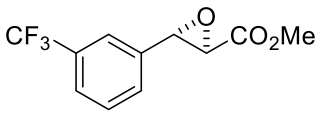

A 100 mL round-bottomed flask was sequentially charged with NaOMe (5.30 g, 98.1 mmol, 1.51 equiv.) and MeOH (35 mL, 1.9 M), then cooled in an ice bath. Once cooled, 3-(Trifluoromethyl)benzaldehyde (8.70 mL, 65.0 mmol, 1.0 equiv.) and methyl chloroacetate (8.50 mL, 97.0 mmol, 1.49 equiv.) were added dropwise and simultaneously. The reaction was then allowed to proceed for 30 h, warming naturally to ambient temperature. Upon completion, the reaction was concentrated *in vacuo*. The residue was diluted with water and extracted three times with diethyl ether. The combined layers were dried over $MgSO_4$, filtered, and concentrated *in vacuo*. The crude mixture was purified by silica gel chromatography using a gradient of 5–10% Et_2O/pentane. Colorless oil (10.5 g, 42.7 mmol, 66%); **^{1}H NMR** (500 MHz, CDCl3) δ 7.59−7.48 (m, 2H), 7.47−7.39 (m, 2H), 4.13 (app s, 1H), 3.77 (s, 3H), 3.47 (app s, 1H); **^{13}C NMR** (126 MHz, CDCl3) δ 168.1, 136.3, 131.1 (q, *J* = 32.6 Hz), 129.3, 129.2, 125.7 (q, *J* = 3.6 Hz), 123.8 (q, *J* = 272.9 Hz), 122.6 (q, *J* = 3.6 Hz), 57.1, 56.6, 52.6; **^{19}F NMR** (471 MHz, $CDCl_3$) δ -62.9; **IR** (thin layer film) ν (cm^{-1}) = 1749, 1441, 1327, 1294, 1210, 1164, 1121, 1071, 1023, 886, 699, 661; **HRMS** (ESI^+) *m/z* calculated for $C_{11}H_{10}O_3F_3^+$ $[M+H]^+$ 247.0577, found 247.0579.

General Procedure I for Opening of Epoxides with Secondary Amines

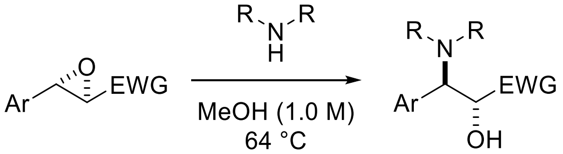

A round-bottomed flask was sequentially charged with epoxide (1.0 equiv.) and MeOH (1.0 M), followed by secondary amine (1.05 equiv.). The reaction was then heated at 64 °C with an exit line overnight. The crude mixture was then concentrated *in vacuo* and purified by silica gel chromatography using the gradient listed below to afford the amino alcohol in the stated regiopurity. Although we have found that a regioisomeric mixture can be used in the next step and obtain similar

results, we sought to isolate the major regioisomer from the epoxide opening where possible (even at the expense of a lower isolated yield).

(±)-methyl 3-(diallylamino)-2-hydroxy-3-phenylpropanoate (*trans* diastereomer)

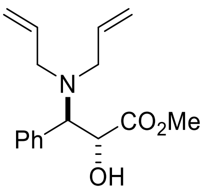

The title compound was prepared according to the general procedure **I**; the reaction was performed on 25.0 mmol scale and was allowed to proceed for 24 h. The crude mixture was purified by silica gel chromatography using a gradient of 2−14% EtOAc/pentane. No minor regioisomer was present in the isolated product. Regioisomer assignment supported by ^{1}H-^{13}C HSQC and HMBC. White solid (5.7 g, 20.7 mmol, 62%); **^{1}H NMR** (400 MHz, CD_3CN) δ 7.40−7.28 (m, 5H), 5.80−5.68 (m, 2H), 5.20−5.09 (m, 4H), 4.75 (dd, *J* = 8.4, 6.9 Hz, 1H), 4.03 (d, *J* = 8.7 Hz, 1H), 3.69 (s, 3H), 3.35 (d, *J* = 6.9 Hz, 1H), 3.33−3.26 (m, 2H), 2.64 (dd, *J* = 14.4, 8.0 Hz, 2H); **^{13}C NMR** (101 MHz, CD_3CN) δ 174.7, 137.7, 136.2, 130.6, 128.9, 128.5, 117.7, 72.7, 66.8, 53.9, 52.2; **IR** (thin layer film) ν (cm^{-1}) = 3456, 2981, 2888, 1717, 1641, 1450, 1416, 1382, 1215, 1082; **mp** = 85−87 °C; **HRMS** (ESI$^+$) *m/z* calculated for $C_{16}H_{22}NO_3^+$ [M+H]$^+$ 276.1594, found 276.1593.

(±)-methyl 2-hydroxy-3-morpholino-3-phenylpropanoate

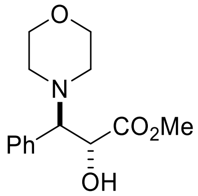

The title compound was prepared according to the general procedure **I**; the reaction was performed on 20.2 mmol scale and was allowed to proceed for 20 h. The crude mixture was purified by silica gel chromatography using a gradient of 8−20% EtOAc/pentane. The compound was isolated as a >20:1 regioisomeric mixture, favoring the shown regioisomer. The regioisomeric ratio of the isolated product was determined by ^{1}H NMR spectroscopic analysis by comparison of the resonances at δ 5.03 (minor regioisomer) and δ 4.77 (major regioisomer). Regioisomer assignment supported by ^{1}H-^{13}C HSQC and HMBC. White solid (3.3 g, 12.4 mmol, 57%); **^{1}H NMR** (500 MHz, $CDCl_3$) δ 7.33−7.26 (m, 5H), 4.77 (d, *J* = 4.4 Hz, 1H), 3.71 (t, *J* = 4.5 Hz, 4H), 3.61 (s, 3H), 3.57 (d, *J* = 4.4 Hz, 1H), 3.02 (br s, 1H), 2.55 (br s, 2H), 2.48−2.44 (m, 2H); **^{13}C NMR** (125 MHz, $CDCl_3$) δ 173.3, 135.7, 129.2, 128.4, 128.4, 72.4, 70.3, 67.1, 52.3, 51.6; **IR** (thin layer film) ν (cm^{-1}) = 3401, 2981, 2889, 1742, 1452, 1382, 1251, 1115, 1072, 956; **mp** = 95−97 °C; **HRMS** (ESI$^+$) *m/z* calculated for $C_{14}H_{20}NO_4^+$ [M+H]$^+$ 266.1387, found 266.1387.

(±)-methyl 2-hydroxy-3-phenyl-3-thiomorpholinopropanoate

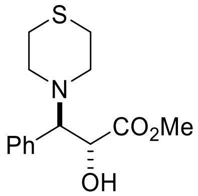

The title compound was prepared according to the general procedure **I**; the reaction was performed on 16.8 mmol scale and was allowed to proceed for 24 h. The crude mixture was purified by silica gel chromatography using a gradient of 10−25% EtOAc/pentane. The compound was isolated as a >20:1 regioisomeric mixture, favoring the shown regioisomer. The regioisomeric ratio of the isolated product was determined by ^{1}H NMR spectroscopic analysis by comparison of the resonances at δ 4.94 (minor regioisomer) and δ 4.74 (major regioisomer). Regioisomer assignment supported by ^{1}H-^{13}C HSQC and HMBC. White solid (3.9 g, 13.8 mmol, 82%); **^{1}H NMR** (500 MHz, $CDCl_3$) δ 7.33−7.19 (m, 5H), 4.74 (d, *J* = 6.1 Hz, 1H), 3.70 (d, *J* = 6.1 Hz, 1H), 3.64 (s, 3H), 3.03−2.84 (br m, 1H), 2.81−2.67 (m, 4H), 2.65−2.53 (m, 4H); **^{13}C NMR** (125 MHz, $CDCl_3$) δ 173.7, 135.2, 129.0, 128.3, 128.1, 72.5, 70.9, 52.7, 52.2, 28.01; **IR** (thin layer film) ν (cm^{-1}) = 3450, 2981, 2908, 1730, 1494, 1453, 1417,

1384, 1280, 1082; **mp** = 119−121 °C; **HRMS** (ESI+) *m/z* calculated for $C_{14}H_{20}NO_3S^+$ [M+H]+ 282.1158, found 282.1158.

(±)-methyl 3-(diisobutylamino)-2-hydroxy-3-phenylpropanoate

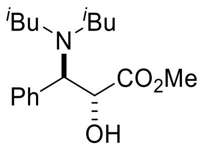

The title compound was prepared according to the general procedure **I**; the reaction was performed on 10.0 mmol scale and was allowed to proceed for 19 h. The crude mixture was purified by silica gel chromatography using a gradient mobile phase of 0−7% EtOAc/pentane. The compound was isolated as a >20:1 regioisomeric mixture, favoring the shown regioisomer. The regioisomeric ratio of the isolated product was determined by ^{1}H NMR spectroscopic analysis by comparison of the resonances at δ 4.91 (minor regioisomer) and δ 4.71 (major regioisomer). Regioisomer assignment supported by ^{1}H-^{13}C HSQC and HMBC. White solid (2.23 g, 7.25 mmol, 73%); **^{1}H NMR** (500 MHz, $CDCl_3$) δ 7.37−7.23 (m, 5H), 4.71 (d, *J* = 6.9 Hz, 1H), 4.07 (d, *J* = 7.2 Hz, 1H), 3.72 (s, 3H), 2.46 (s, 1H), 2.22 (dd, *J* = 12.9, 9.4 Hz, 2H), 2.09 (dd, *J* = 12.9, 4.9 Hz, 2H), 1.81-1.73 (m, 2H), 0.90 (d, *J* = 6.5 Hz, 6H), 0.82 (d, *J* = 6.7 Hz, 6H); **^{13}C NMR** (125 MHz, $CDCl_3$) δ 174.3, 135.3, 130.1, 128.1, 127.8, 72.1, 67.8, 59.9, 52.3, 26.4, 21.2, 20.8; **IR** (thin layer film) ν (cm^{-1}) = 3457, 2955, 1741, 1459, 1387, 1267, 1200, 1176, 1059, 702; **mp** = 93−95 °C; **HRMS** (ESI+) *m/z* calculated for $C_{18}H_{30}NO_3^+$ [M+H]+ 308.2220, found 308.2218.

(±)-methyl 2-hydroxy-3-phenyl-3-(4-phenylpiperazin-1-yl)propanoate

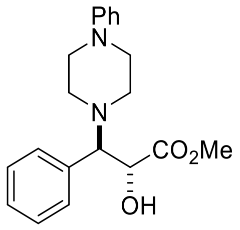

The title compound was prepared according to the general procedure **I**; the reaction was performed on 20.0 mmol scale and was allowed to proceed for 24 h. The crude mixture was purified by silica gel chromatography using a gradient from 20% Et_2O/pentane to pure Et_2O. The compound was isolated as a 12.0:1 regioisomeric mixture, favoring the shown regioisomer. The regioisomeric ratio of the isolated product was determined by ^{1}H NMR spectroscopic analysis by comparison of the resonances at δ 5.10 (minor regioisomer) and δ 4.87 (major regioisomer). Regioisomer assignment supported by ^{1}H-^{13}C HSQC and HMBC. White solid (5.13 g, 15.1 mmol, 75%); **^{1}H NMR** (500 MHz, $CDCl_3$) δ 7.42−7.32 (m, 5H), 7.31−7.25 (m, 2H) 6.93 (d, *J* = 8.0 Hz, 2H), 6.88 (t, *J* = 7.3 Hz, 1H), 4.87 (br s, 1H), 3.69 (d, *J* = 4.8 Hz, 1H), 3.65 (s, 3H), 3.23 (t, *J* = 5.0 Hz, 4H), 3.14−3.08 (m, 1H), 2.79−2.72 (m, 2H), 2.71−2.64 (m, 2H); **^{13}C NMR** (126 MHz, $CDCl_3$) δ 173.3, 151.2, 135.8, 129.2, 129.2, 128.4, 128.4, 119.9, 116.1, 72.1, 70.7, 52.3, 51.0, 49.4; **IR** (thin layer film) ν (cm^{-1}) = 2825, 1739, 1599, 1496, 1452, 1386, 1234, 1140, 1007, 760; **mp** = 128−130 °C; **HRMS** (ESI+) *m/z* calculated for $C_{20}H_{25}N_2O_3^+$ [M+H]+ 341.1860, found 341.1857.

(±)-methyl 2-(4-(2-((2,4-dimethylphenyl)thio)phenyl)piperazin-1-yl)-3-hydroxy-3-phenylpropanoate

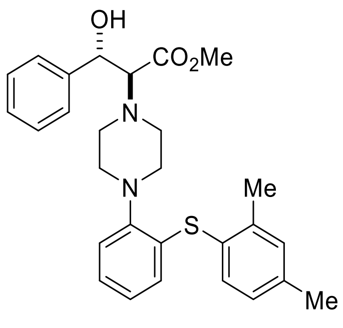

The title compound was prepared according to the general procedure **I** using equal molar equivalents of epoxide and secondary amine (vortioxetine); in this case, we isolated the minor regiosomer formed during the epoxide opening (the α-amino ester). The reaction was performed on 6.0 mmol scale and was allowed to proceed for 24 h. The crude mixture was purified by silica gel chromatography using a gradient from 20−40% Et_2O/pentane. The compound was isolated as a 7.1:1 regioisomeric mixture, favoring the shown regioisomer. The

regioisomeric ratio of the isolated product was determined by ^{1}H NMR spectroscopic analysis by comparison of the resonances at δ 5.11 (major regioisomer) and δ 4.61 (minor regioisomer). Regioisomer assignment supported by ^{1}H-^{13}C HSQC and HMBC. White solid (343 mg, 0.7 mmol, 12%); **^{1}H NMR** (500 MHz, $CDCl_3$) δ 7.41−7.33 (m, 5H), 7.32−7.26 (m, 1H), 7.14 (s, 1H), 7.09−6.95 (m, 3H), 6.87−6.82 (m, 1H), 6.51−6.46 (m, 1H), 5.11 (dd, *J* = 7.0, 4.0 Hz, 1H), 3.66 (s, 3H), 3.39 (d, *J* = 7.2 Hz, 1H), 3.27 (d, *J* = 4.2 Hz, 1H), 3.04−2.95 (m, 4H), 2.93-2.86 (m, 2H), 2.78−2.71 (m, 2H), 2.35 (s, 3H), 2.30 (s, 3H); **^{13}C NMR** (126 MHz, $CDCl_3$) δ 171.7, 149.2, 142.5, 141.1, 139.3, 136.3, 134.6, 131.8, 128.3, 128.1, 127.9, 127.9, 126.6, 126.3, 125.6, 124.5, 119.9, 73.9, 71.5, 51.9, 51.6, 51.1, 21.3, 20.7; **IR** (thin layer film) ν (cm^{-1}) = 3657, 2981, 2889, 1703, 1381, 1250, 1155, 1072, 954; **mp** = 68−70 °C; **HRMS** (ESI^+) *m/z* calculated for $C_{28}H_{33}N_2O_3S^+$ $[M+H]^+$ 477.2206, found 477.2205.

(±)-methyl 2-(dibenzylamino)-3-hydroxy-3-phenylpropanoate

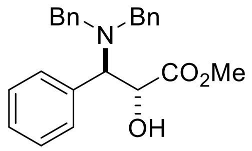

The title compound was prepared according to the general procedure **I**; the reaction was performed on 16.8 mmol scale and was allowed to proceed for 24 h. The crude mixture was purified by silica gel chromatography using a gradient of 10−20% EtOAc/pentane. The compound was isolated as a 16.7:1 regioisomeric mixture, favoring the shown regioisomer. The regioisomeric ratio of the isolated product was determined by ^{1}H NMR spectroscopic analysis by comparison of the resonances at δ 2.51 (minor regioisomer) and δ 2.31 (major regioisomer). Regioisomer assignment supported by ^{1}H-^{13}C HSQC and HMBC. White solid (4.6 g, 12.3 mmol, 73%); **^{1}H NMR** (400 MHz, $CDCl_3$) δ 7.37−7.30 (m, 2H), 7.30−7.19 (m, 11H), 7.19−7.12 (m, 2H), 4.80 (t, *J* = 7.3 Hz, 1H), 3.96 (d, *J* = 7.7 Hz, 1H), 3.83 (d, *J* = 13.7 Hz, 2H), 3.65 (s, 3H), 3.14 (d, *J* = 13.8 Hz, 2H), 2.31 (d, *J* = 7.0 Hz, 1H); **^{13}C NMR** (101 MHz, $CDCl_3$) δ 173.7, 139.3, 134.4, 129.9, 128.9, 128.4, 128.4, 128.1, 127.2, 72.1, 65.8, 54.6, 52.4; **IR** (thin layer film) ν (cm^{-1}) = 3481, 1738, 1494, 1453, 1282, 1205, 1069, 1028, 912, 699; **mp** = 110−112 °C; **HRMS** (ESI^+) *m/z* calculated for $C_{24}H_{26}NO_3^+$ $[M+H]^+$ 376.1907, found 376.1904.

(±)-methyl 3-(4-bromophenyl)-3-(diallylamino)-2-hydroxypropanoate

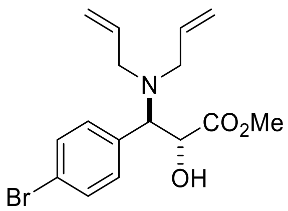

The title compound was prepared according to the general procedure **I**; the reaction was performed on 25.0 mmol scale and was allowed to proceed for 24 h. The crude mixture was purified by silica gel chromatography using a gradient mobile phase of 2−14% EtOAc/pentane. The compound was isolated as a 13.4:1 regioisomeric mixture, favoring the shown regioisomer. The regioisomeric ratio of the isolated product was determined by ^{1}H NMR spectroscopic analysis by comparison of the resonances at δ 4.15 (minor regioisomer) and δ 4.02 (major regioisomer). Regioisomer assignment supported by ^{1}H-^{13}C HSQC and HMBC. Yellow oil (4.42 g, 12.5 mmol, 50%); **^{1}H NMR** (500 MHz, $CDCl_3$) δ 7.45 (d, *J* = 8.4 Hz, 2H), 7.18 (d, *J* = 8.4 Hz, 2H), 5.89−5.70 (m, 2H), 5.19−5.08 (m, 4H), 4.75 (d, *J* = 5.6 Hz, 1H), 4.02 (d, *J* = 5.7 Hz, 1H), 3.68 (s, 3H), 3.31 (dd, *J* = 14.5, 5.3 Hz, 2H), 2.94 (dd, *J* = 14.5, 7.4 Hz, 2H), 2.81 (s, 1H); **^{13}C NMR** (126 MHz, $CDCl_3$) δ 173.6, 135.3, 135.0, 131.5, 131.0, 122.1, 118.0, 71.4, 66.1, 53.0, 52.4; **IR** (thin layer film) ν (cm^{-1}) = 3457, 2817, 1739, 1642, 1590, 1488, 1438, 1277, 1204, 921; **HRMS** (ESI^+) *m/z* calculated for $C_{16}H_{21}BrNO_3^+$ $[M+H]^+$ 354.0699, found 354.0701.

(±)-methyl 3-(4-chlorophenyl)-3-(diallylamino)-2-hydroxypropanoate

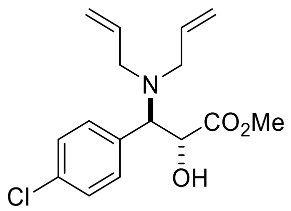

The title compound was prepared according to the general procedure **I**; the reaction was performed on 18.8 mmol scale and was allowed to proceed for 24 h. The crude mixture was purified by silica gel chromatography using a gradient mobile phase of 5−20% EtOAc/pentane. The compound was isolated as a >20:1 regioisomeric mixture, favoring the shown regioisomer. The regioisomeric ratio of the isolated product was determined by ^{1}H NMR spectroscopic analysis by comparison of the resonances at δ 4.16 (minor regioisomer) and δ 4.03 (major regioisomer). Regioisomer assignment supported by ^{1}H-^{13}C HSQC and HMBC. Yellow oil (3.50 g, 11.3 mmol, 60%); **^{1}H NMR** (500 MHz, $CDCl_3$) δ 7.30−7.28 (m, 2H), 7.25−7.22 (m, 2H), 5.83−5.75 (m, 2H), 5.17−5.12 (m, 4H), 4.75 (d, *J* = 4.4 Hz, 1H), 4.03 (d, *J* = 5.8 Hz, 1H), 3.68 (s, 3H), 3.31 (dd, *J* = 14.5, 5.2 Hz, 2H), 2.94 (dd, *J* = 14.5, 7.5 Hz, 2H) , 2.83 (br s, 1H); **^{13}C NMR** (126 MHz, $CDCl_3$) δ 173.6, 135.4, 134.4, 133.9, 130.6, 128.5, 118.0, 71.4, 66.1, 53.0, 52.4; **IR** (thin layer film) ν (cm^{-1}) = 3470, 2817, 1730, 1642, 1491, 1439, 1417, 1276, 1204, 1092; **HRMS** (ESI$^+$) *m/z* calculated for $C_{16}H_{21}ClNO_3^+$ [M+H]$^+$ 310.1204, found 310.1203.

(±)-methyl 3-(diallylamino)-2-hydroxy-3-(*p*-tolyl)propanoate

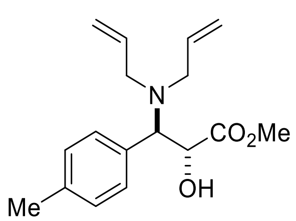

The title compound was prepared according to the general procedure **I**; the reaction was performed on 26 mmol scale and was allowed to proceed for 3 h. The crude mixture was purified by silica gel chromatography using a gradient of 10−20% EtOAc/pentane. The compound was isolated as a >20:1 regioisomeric mixture, favoring the shown regioisomer. The regioisomeric ratio of the isolated product was determined by ^{1}H NMR spectroscopic analysis by comparison of the resonances at δ 4.95 (minor regioisomer) and δ 4.75 (major regioisomer). Regioisomer assignment supported by ^{1}H-^{13}C HSQC and HMBC. Colorless oil (7.0 g, 24.2 mmol, 93%); **^{1}H NMR** (500 MHz, $CDCl_3$) δ 7.17 (d, *J* = 8.1 Hz, 2H), 7.13 (d, *J* = 8.1 Hz, 2H), 5.84−5.76 (m, 2H), 5.16−5.13 (m, 4H), 4.75 (d, *J* = 6.3 Hz, 1H), 4.01 (d, *J* = 6.3 Hz, 1H), 3.67 (s, 3H), 3.32 (dd, *J* = 14.5, 5.1 Hz, 2H), 2.92 (dd, *J* = 14.5, 7.6 Hz, 2H), 2.74 (br s, 1H), 2.33 (s, 3H); **^{13}C NMR** (126 MHz, $CDCl_3$) δ 173.8, 137.8, 135.7, 132.4, 129.2, 129.1, 117.7, 71.7, 66.5, 53.1, 52.2, 21.3; **IR** (thin layer film) ν (cm^{-1}) =2980, 2817, 1738, 1642, 1439, 1418, 1263, 1217, 1198, 918; **HRMS** (ESI$^+$) *m/z* calculated for $C_{17}H_{24}NO_3^+$ [M+H]$^+$ 290.1751, found 290.1748.

(±)-methyl 4-(1-(diallylamino)-2-hydroxy-3-methoxy-3-oxopropyl)benzoate

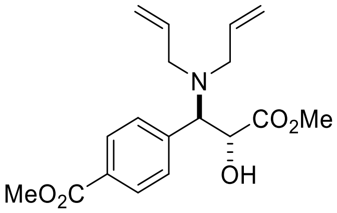

The title compound was prepared according to the general procedure **I**; the reaction was performed on 25 mmol scale and was allowed to proceed for 24 h. The crude mixture was purified by silica gel chromatography using a gradient of 8−20% EtOAc/pentane. The compound was isolated as a >20:1 regioisomeric mixture, favoring the shown regioisomer. The regioisomeric ratio of the isolated product was determined by ^{1}H NMR spectroscopic analysis by comparison of the resonances at δ 5.85-5.74 (major regioisomer) and δ 5.41-5.31 (minor regioisomer). Regioisomer assignment supported by ^{1}H-^{13}C HSQC and HMBC. White solid (2.95 g, 8.8 mmol, 35%); **^{1}H NMR** (500 MHz, $CDCl_3$) δ 7.99 (d, *J* = 8.3 Hz, 2H), 7.38 (d, *J* = 8.3 Hz, 2H), 5.85−5.74 (m, 2H), 5.20−5.09 (m, 4H), 4.79 (d, *J* = 5.8 Hz, 1H), 4.11 (d, *J* = 5.8 Hz, 1H), 3.90 (s, 3H), 3.67 (s, 3H), 3.31 (dd, *J* = 14.5, 5.2 Hz, 2H), 2.94 (dd, *J* = 14.6, 7.5 Hz, 2H), 2.85 (br

s, 1H); **^{13}C NMR** (126 MHz, $CDCl_3$) δ 173.6, 167.0, 141.3, 135.3, 129.8, 129.6, 129.3, 118.1, 71.3, 66.5, 53.1, 52.4, 52.2; **IR** (thin layer film) ν (cm^{-1}) = 3456, 1723, 1611, 1437, 1201, 1193, 1109, 1020, 921, 870; **mp** = 104−106 °C; **HRMS** (ESI^+) *m/z* calculated for $C_{18}H_{24}NO_5^+$ $[M+H]^+$ 334.1649, found 334.1648.

(±)-methyl 3-(diallylamino)-3-(2-fluorophenyl)-2-hydroxypropanoate

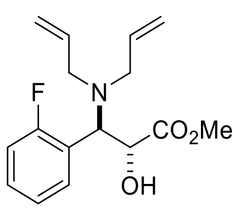

The title compound was prepared according to the general procedure **I**; the reaction was performed on 15.3 mmol scale and was allowed to proceed for 24 h. The crude mixture was purified by silica gel chromatography using a gradient of 5−10% EtOAc/pentane. The compound was isolated as a 6.8:1 regioisomeric mixture, favoring the shown regioisomer. The regioisomeric ratio of the isolated product was determined by ^{1}H NMR spectroscopic analysis by comparison of the resonances at δ 7.43 (major regioisomer) and δ 7.33 (minor regioisomer). Regioisomer assignment supported by ^{1}H-^{13}C HSQC and HMBC. White solid (1.4 g, 4.84 mmol, 32%); **^{1}H NMR** (400 MHz, $CDCl_3$) δ 7.43 (td, *J* = 7.4, 1.5 Hz, 1H), 7.25−7.15 (m, 1H), 7.07 (td, *J* = 7.6, 1.1 Hz, 1H), 7.01−6.94 (m, 1H), 5.79−5.65 (m, 2H), 5.14−5.03 (m, 4H), 4.73 (d, *J* = 6.6 Hz, 1H), 4.37 (d, *J* = 6.6 Hz, 1H), 3.64 (s, 3H), 3.28 (dd, *J* = 14.3, 5.3 Hz, 2H), 2.86 (dd, *J* = 14.5, 7.5 Hz, 2H); **^{13}C NMR** (101 MHz, $CDCl_3$) δ 173.9, 161.5 (d, *J* = 246.8 Hz), 135.6, 131.1 (d, *J* = 4.4 Hz), 129.6 (d, *J* = 8.6 Hz), 124.0 (d, *J* = 3.5 Hz), 122.8 (d, *J* = 14.5 Hz), 117.8, 115.7 (d, *J* = 23.9 Hz), 71.5 (d, *J* = 1.9 Hz), 60.0, 53.4, 52.3; **^{19}F NMR** (376 MHz, $CDCl_3$) δ -115.8; **IR** (thin layer film) ν (cm^{-1}) = 3460, 2821, 1721, 1642, 1613, 1490, 1450, 1330, 1213, 1089; **mp** = 67−69 °C**;** **HRMS** (ESI^+) *m/z* calculated for $C_{16}H_{21}NO_3F^+$ $[M+H]^+$ 294.1500, found 294.1500.

(±)-methyl 3-(diallylamino)-2-hydroxy-3-(3-(trifluoromethyl)phenyl)propanoate

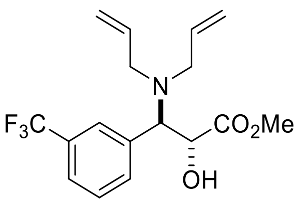

The title compound was prepared according to the general procedure **I**; the reaction was performed on 20.3 mmol scale and was allowed to proceed for 24 h. The crude mixture was purified by silica gel chromatography using a gradient of 5−10% EtOAc/pentane. The compound was isolated as a 5.0:1 regioisomeric mixture, favoring the shown regioisomer. The regioisomeric ratio of the isolated product was determined by ^{1}H NMR spectroscopic analysis by comparison of the resonances at δ 3.79 (minor regioisomer) and δ 3.69 (major regioisomer). Regioisomer assignment supported by ^{1}H-^{13}C HSQC and HMBC. Low-melting white solid (3.3 g, 9.6 mmol, 48%); **^{1}H NMR** (500 MHz, $CDCl_3$) δ 7.59−7.50 (m, 3H), 7.45 (t, *J* = 7.6 Hz, 1H), 5.88−5.75 (m, 2H), 5.22−5.09 (m, 4H), 4.80 (br s, 1H), 4.13 (d, *J* = 5.5 Hz, 1H), 3.69 (s, 3H), 3.33 (dd, *J* = 14.6, 5.3 Hz, 2H), 2.99 (dd, *J* = 14.4, 7.4 Hz, 2H), 2.79 (br s, 1H); **^{13}C NMR** (126 MHz, $CDCl_3$) δ 173.6, 137.3, 135.1, 132.5, 130.6 (q, *J* = 32.3 Hz), 128.8, 126.1 (q, *J* = 3.9 Hz), 124.9 (q, *J* = 3.6 Hz), 124.2 (q, *J* = 272.4 Hz), 118.1, 71.2, 66.5, 53.1, 52.5; **^{19}F NMR** (471 MHz, $CDCl_3$) δ -62.6; **IR** (thin layer film) ν (cm^{-1}) = 3490, 1738, 1447, 1320, 1281, 1167, 1124, 1075, 997, 922; **mp** = 58−60 °C; **HRMS** (ESI^+) *m/z* calculated for $C_{17}H_{21}NO_3F_3^+$ $[M+H]^+$ 344.1468, found 344.1468.

(±)-*tert*-butyl 3-(diallylamino)-2-hydroxy-3-phenylpropanoate

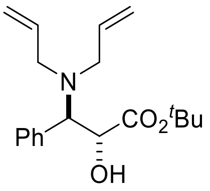

The title compound was prepared according to the general procedure **I**; the reaction was performed on 25.0 mmol scale and was allowed to proceed for 15 h. The crude mixture was purified by silica gel chromatography using a gradient mobile phase of 2−10% EtOAc/pentane. The compound was isolated as a >20:1 regioisomeric mixture, favoring the shown regioisomer. The regioisomeric ratio of

the isolated product was determined by ^{1}H NMR spectroscopic analysis by comparison of the resonances at δ 4.03 (major regioisomer) and δ 3.57 (minor regioisomer). Regioisomer assignment supported by ^{1}H-^{13}C HSQC and HMBC. Colorless oil (3.93 g,12.4 mmol, 49.5%); **^{1}H NMR** (500 MHz, $CDCl_3$) δ 7.34−7.26 (m, 5H), 5.89−5.81 (m, 2H), 5.18−5.13 (m, 4H), 4.63 (br s, 1H), 4.03 (d, *J* = 5.5 Hz, 1H), 3.35 (dd, *J* = 14.4, 5.3 Hz, 2H), 2.97 (dd, J = 14.3, 7.5 Hz, 2H), 2.85 (br s, 1H), 1.37 (s, 9H). **^{13}C NMR** (126 MHz, $CDCl_3$) δ 172.6, 136.1, 135.9, 129.8, 128.2, 127.9, 117.6, 82.5, 71.7, 66.4, 53.3, 28.0; **IR** (thin layer film) ν (cm^{-1}) = 3481, 3075, 2815, 1725, 1642, 1495, 1454, 1418, 1159, 918; **HRMS** (ESI^+) *m/z* calculated for C19H28NO_3^+ $[M+H]^+$ 318.2064, found 318.2062.

(±)-3-(diallylamino)-*N*,*N*-diethyl-2-hydroxy-3-phenylpropanamide

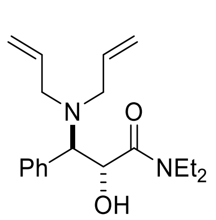

The title compound was prepared according to the general procedure **I**; the reaction was performed on 10.0 mmol scale and was allowed to proceed for 24 h. The crude mixture was purified by silica gel chromatography using a gradient mobile phase of 10—30% EtOAc/pentane. No minor regioisomer was present in the isolated product. Regioisomer assignment supported by ^{1}H-^{13}C HSQC and HMBC. Yellow solid (2.1 g, 6.6 mmol, 69%); **^{1}H NMR** (400 MHz, $CDCl_3$) δ 7.26—7.15 (m, 5H), 5.85—5.73 (m, 2H), 5.15—5.04 (m, 4H), 4.79 (d, *J* = 4.6 Hz, 1H), 3.84 (d, *J* = 4.6 Hz, 1H), 3.61–3.46 (m, 2H), 3.42 (dd, *J* = 14.6, 5.2 Hz, 2H), 3.25—3.13 (m, 1H), 3.03—2.93 (m, 1H), 2.90 (dd, *J* = 14.6, 7.5 Hz, 2H), 1.17 (t, *J* = 7.1 Hz, 3H), 0.99 (t, *J* = 7.1 Hz, 3H); **^{13}C NMR** (101 MHz, $CDCl_3$) δ 172.1, 136.2, 135.9, 129.8, 127.9, 127.7, 117.4, 68.9, 66.5, 53.6, 41.3, 40.5, 14.3, 12.8; **IR** (thin layer film) ν (cm^{-1}) = 3045, 2815, 1630, 1453, 1403, 1359, 1280, 1219, 1146, 917; **mp** = 64—66 °C; **HRMS** (ESI^+) *m/z* calculated for $C_{19}H_{29}N_2O_2^+$ $[M+H]^+$ 317.2224, found 317.2221.

(±)-methyl 3-(diallylamino)-2-hydroxy-3-(naphthalen-2-yl)propanoate

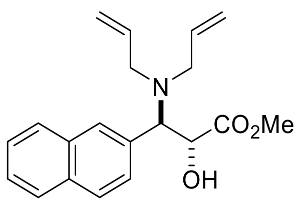

The title compound was prepared according to the general procedure **I**; the reaction was performed on 11.0 mmol scale and was allowed to proceed for 24 h. The crude mixture was purified by silica gel chromatography using a gradient of 5−15% EtOAc/pentane. The compound was isolated as a >20:1 regioisomeric mixture, favoring the shown regioisomer. The regioisomeric ratio of the isolated product was determined by ^{1}H NMR spectroscopic analysis by comparison of the resonances at δ 4.89 (major regioisomer) and δ 4.70 (minor regioisomer). Regioisomer assignment supported by ^{1}H-^{13}C HSQC and HMBC. Yellow oil (2.74 g, 8.4 mmol, 76%); **^{1}H NMR** (500 MHz, $CDCl_3$) δ 7.87−7.79 (m, 3H), 7.75 (s, 1H), 7.52−7.44 (m, 3H), 5.92−5.80 (m, 2H), 5.21−5.13 (m, 4H), 4.89 (d, *J* = 5.9 Hz, 1H), 4.24 (d, *J* = 5.8 Hz, 1H), 3.66 (s, 3H), 3.39 (ddd, *J* = 14.5, 4.5, 2.6 Hz, 2H), 3.03 (dd, *J* = 14.5, 7.4 Hz, 2H), 2.88 (s, 1H); **^{13}C NMR** (126 MHz, $CDCl_3$) δ 173.7, 135.6, 133.5, 133.3, 133.2, 128.6, 128.1, 128.0, 127.8, 127.0, 126.2, 126.2, 117.9, 71.7, 66.9, 53.1, 52.3. **IR** (thin layer film) ν (cm^{-1}) =3478, 3060, 2817, 1739, 1641, 1508, 1438, 1274, 920, 709; **HRMS** (ESI^+) *m/z* calculated for $C_{20}H_{24}NO_3^+$ $[M+H]^+$ 326.1751, found 326.1747.

(±)-methyl 3-(diallylamino)-2-hydroxy-3-phenylpropanoate (*cis* diastereomer)

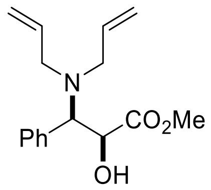

The title compound was prepared according to the general procedure **I**; the reaction was performed on 15.0 mmol scale and was allowed to proceed for 24 h. The crude mixture was purified by silica gel chromatography using a gradient of 6−18% Et_2O/pentane. The compound was isolated as a >20:1 regioisomeric mixture, favoring the shown regioisomer. The regioisomeric ratio of the isolated product was determined by ^{1}H NMR spectroscopic analysis by comparison of the resonances at δ 4.63 (major regioisomer) and δ 4.25 (minor regioisomer). Regioisomer assignment supported by ^{1}H-^{13}C HSQC and HMBC. Yellow oil (1.68 g, 6.1 mmol, 41%); **^{1}H NMR** (500 MHz, $CDCl_3$) δ 7.41−7.32 (m, 3H), 7.30−7.25 (m, 2H),5.87−5.75 (m, 2H), 5.26−5.16 (m, 4H), 4.63 (d, *J* = 9.0 Hz, 1H), 4.11 (d, *J* = 9.0 Hz, 1H), 3.58 (s, 3H), 3.46−3.38 (m, 2H), 2.71 (dd, *J* = 14.2, 8.4 Hz, 2H); **^{13}C NMR** (126 MHz, $CDCl_3$) δ 172.9, 136.0, 133.8, 129.8, 128.3, 128.3, 118.4, 70.4, 65.1, 53.3, 52.2; **IR** (thin layer film) ν (cm^{-1}) = 3075, 2823, 1744, 1642, 1494, 1451, 1418, 1259, 1207, 1142; **HRMS** (ESI$^+$) *m/z* calculated for $C_{16}H_{22}NO_3^+$ [M+H]$^+$ 276.1594, found 276.1593.

(±)-methyl 3-(diallylamino)-2-hydroxy-5-phenylpent-4-ynoate

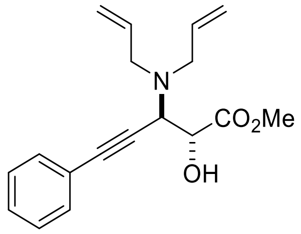

The title compound was prepared by a two-step procedure **I**. To a solution of NaOMe (2.92 g, 54.0 mmol, 1.5 equiv.) in MeOH (15.6 mL, 2.3 M) in a 100 mL round-bottomed flask in an ice bath was added phenylpropargyl aldehyde (4.40 mL, 36.0 mmol, 1.0 equiv.) and methyl chloroacetate (4.73 mL, 54.0 mmol, 1.5 equiv.) dropwise and simultaneously. After 24 h, the reaction was concentrated *in vacuo* and partitioned between water and diethyl ether. The layers were separated and the aqueous layer was extracted twice with diethyl ether. The combined organic layers were dried with magnesium sulfate, filtered, and concentrated to yield crude *trans*-methyl 3-(phenylethynyl)oxirane-2-carboxylate, which was purified by silica gel chromatography using a gradient of 0−4% Et_2O/pentane. The epoxide was obtained as a 4.1:1 *trans*/*cis* mixture favoring the *trans* isomer (1.5 g, 7.4 mmol, 21% yield; *trans* and *cis* epoxides could not be separated) which was used in the next reaction directly. The major diastereomer was assigned as *trans* using the methine C-H coupling constants (1.8 Hz for *trans*, 3.1 Hz for *cis*). The diastereomeric mixture of epoxides thusly obtained was dissolved in MeOH (7.4 mL, 1.0 M) in a 14 mL vial before adding diallylamine (1.83 mL, 14.8 mmol, 2.0 equiv.). The reaction was heated in an oil bath at 64 °C with an exit line for 24 h before concentrating *in vacuo*. The crude reaction mixture was purified by silica gel chromatography using a gradient from 6−20% Et_2O/pentane (flushing with pure Et_2O at the end) to obtain the title compound as an orange oil (963 mg, 3.2 mmol, 43% yield; 9% yield over two steps). Regioisomer assignment supported by ^{1}H-^{13}C HSQC and HMBC. Relative stereochemistry inferred from the fact that a yield of 43% in the epoxide opening reaction could only arise from opening the major (*trans*) epoxide of the first step. **^{1}H NMR** (500 MHz, $CDCl_3$) δ 7.47−7.42 (m, 2H), 7.34−7.28 (m, 3H), 5.88−5.78 (m, 2H), 5.26−5.20 (m, 2H), 5.19−5.14 (m, 2H), 4.42 (dd, *J* = 8.2, 5.5 Hz, 1H), 4.13 (d, *J* = 5.4 Hz, 1H), 3.80 (s, 3H), 3.47−3.39 (m, 2H), 3.14 (d, *J* = 8.3 Hz, 1H), 3.08 (dd, *J* = 14.3, 7.9 Hz, 2H); **^{13}C NMR** (126 MHz, $CDCl_3$) δ 173.0, 136.0, 132.0, 128.6, 128.4, 122.7, 117.9, 88.0, 83.0, 73.2, 56.9. 55.0, 52.6; **IR** (thin layer film) ν (cm^{-1}) = 3466, 3079, 1742, 1643, 1598, 1490, 1443, 1419, 1277, 757; **HRMS** (ESI$^+$) *m/z* calculated for $C_{18}H_{22}NO_3^+$ [M+H]$^+$ 300.1594, found 300.1592.

(±)-2-(diallylamino)-1-phenylethan-1-ol

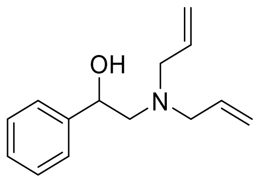

The title compound was prepared according to a previously published procedure[12]; To a solution of water (3.4 mL, 0.4 M) and styrene oxide (1.0 g, 8.4 mmol, 1 equiv.) in a 25 mL round-bottomed flask in an ice-water bath was added diallylamine (1.24 mL, 10.0 mmol, 1.2 equiv.) The ice-water bath was then removed and the reaction was allowed to proceed at room temperature. After 22 h, 3.4 mL of water was added and the reaction partitioned between water and ethyl acetate. The aqueous layer was extracted twice with ethyl acetate. The combined organic layers were dried with magnesium sulfate, filtered, and concentrated *in vacuo*. The crude was purified by silica gel chromatography using 10% EtOAc/pentane to obtain the title compound as an yellow liquid (0.86 g, 4.0 mmol, 47% yield). The compound was isolated as a 2.2:1 regioisomeric mixture, favoring the shown regioisomer The regioisomeric ratio of the isolated product was determined by ^{1}H NMR spectroscopic analysis by comparison of the resonances at δ 4.72 (major regioisomer) and δ 3.65 (minor regioisomer). **^{1}H NMR** (500 MHz, $CDCl_3$) δ 7.44−7.33 (m, 4H), 7.32−7.26 (m, 1H), 5.94-5.85 (m, 2H), 5.26−5.17 (m, 4H), 4.72 (dd, *J* = 10.2, 3.9 Hz, 1H), 3.39 (dd, *J* = 14.0, 5.7 Hz, 2H), 3.12 (dd, *J* = 14.1, 7.6 Hz, 2H), 2.64 (dd, *J* = 12.9, 3.9 Hz, 1H), 2.59 (dd, *J* = 12.9, 10.2 Hz, 1H); **^{13}C NMR** (126 MHz, $CDCl_3$) δ 142.4, 135.0, 128.4, 127.6, 126.0, 118.4, 69.5, 61.5, 56.9; **IR** (thin layer film) ν (cm^{-1}) = 3423, 2818, 1643, 1451, 1417, 1060, 1027, 994, 317, 755, 699; **HRMS** (ESI^+) *m/z* calculated for $C14H20NO^+$ $[M+H]^+$ 218.1539, found 218.1540.

General Procedure II for Chlorination of Amino Alcohols

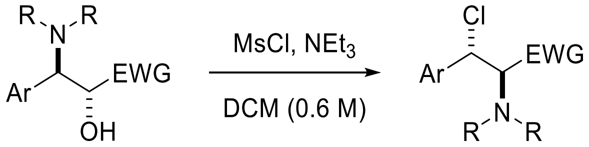

A round-bottom flask was charged with the amino alcohol (1.0 equiv.) and dichloromethane (0.6 M), followed by triethylamine (1.1 equiv.). Then, at 0 °C and under an atmosphere of nitrogen, methanesulfonyl chloride (1.1 equiv.) was added dropwise. The reaction was allowed to warm to room temperature and was stirred until completion. The reaction was then forced through a short plug of SiO_2 with diethyl ether and concentrated *in vacuo* to afford the chloro amine product in the stated regiopurity. Although products were sometimes obtained in initially poor regioselectivity, they were found to equilibrate to the benzylic chloride after heating at 50 °C (oil bath) under high vacuum. In some cases (**(±)-methyl 3-cyclohexyl-2-(dibenzylamino)-3-((methylsulfonyl)oxy)propanoate** and **(±)-methyl 3-(diallylamino)-2-((methylsulfonyl)oxy)-5-phenylpent-4-ynoate**), the mesylate was formed, rather than the chloride – in those cases, thermodynamic equilibration was not performed and the mesylate was used directly in the fluorination reaction.

Characterization Data for Amino Chlorides

(±)-methyl 3-chloro-2-(diallylamino)-3-phenylpropanoate (*trans* diastereomer)

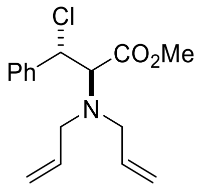

The title compound was prepared according to the general procedure **II**; the reaction was performed on 10.5 mmol scale and was allowed to proceed for 2 h. The compound was isolated as a >20:1 regioisomeric mixture, favoring the shown regioisomer. The regioisomeric ratio of the isolated product was determined by ^{1}H NMR spectroscopic analysis by comparison of the resonances at δ 4.38 (minor regioisomer) and δ 3.99 (major regioisomer). Regioisomer assignment supported by ^{1}H-^{13}C HSQC and HMBC. Low-melting white solid (3.02 g, 10.3 mmol, 98%); **^{1}H NMR** (400 MHz, $CDCl_3$) δ 7.37−7.28 (m, 5H), 5.40−5.29 (m, 2H), 5.14 (d, *J* = 11.0 Hz, 1H), 5.03−4.98 (m, 4H), 3.99 (d, *J* = 10.9 Hz, 1H), 3.82 (s, 3H), 3.27−3.21 (m, 2H), 2.82 (dd, *J* = 14.4, 8.0 Hz, 2H); **^{13}C NMR** (101 MHz, $CDCl_3$) δ 170.5, 138.7, 135.5, 128.6, 128.4, 128.3, 117.9, 67.8, 60.1, 53.4, 51.6; **IR** (thin layer film) ν (cm^{-1}) = 2822, 1735, 1643, 1497, 1454, 1434, 1357, 1267, 1192, 923; **mp** = 44−46 °C; **HRMS** (ESI^+) *m/z* calculated for $C_{16}H_{21}ClNO_2^+$ $[M+H]^+$ 294.1255, found 294.1254.

(±)-methyl 3-chloro-2-morpholino-3-phenylpropanoate

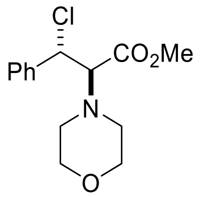

The title compound was prepared according to the general procedure **II**; the reaction was performed on 5.7 mmol scale and was allowed to proceed for 2.5 h. The compound was isolated as a 12:1 regioisomeric mixture, favoring the shown regioisomer. The regioisomeric ratio of the isolated product was determined by ^{1}H NMR spectroscopic analysis by comparison of the resonances at δ 4.77 (minor regioisomer) and δ 5.15 (major regioisomer). Regioisomer assignment supported by ^{1}H-^{13}C HSQC and HMBC. White solid (1.3 g, 4.6 mmol, 81%); **^{1}H NMR** (500 MHz, $CDCl_3$) δ 7.41−7.28 (m, 5H), 5.15 (d, *J* = 10.8 Hz, 1H), 3.81 (s, 3H), 3.73 (d, *J* = 10.8 Hz, 1H), 3.42−3.28 (m, 4H), 2.64−2.55 (m, 2H), 2.42−2.34 (m, 2H); **^{13}C NMR** (126 MHz, $CDCl_3$) δ 169.2, 138.5, 128.7, 128.5, 127.9, 73.7, 67.1, 59.2, 51.7, 50.2; **IR** (thin layer film) ν (cm^{-1}) = 2953, 2854, 1733, 1453, 1435, 1348, 1295, 1192, 1115, 868; **mp** = 115−117 °C; **HRMS** (ESI^+) *m/z* calculated for $C_{14}H_{19}ClNO_3^+$ $[M+H]^+$ 284.1048, found 284.1047.

(±)-methyl 3-chloro-3-phenyl-2-thiomorpholinopropanoate

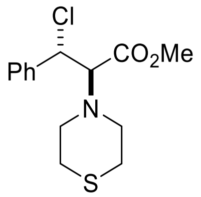

The title compound was prepared according to the general procedure **II**; the reaction was performed on 7.1 mmol scale and was allowed to proceed for 4 h. The compound was isolated as a 12.5:1 regioisomeric mixture, favoring the shown regioisomer. The regioisomeric ratio of the isolated product was determined by ^{1}H NMR spectroscopic analysis by comparison of the resonances at δ 5.15 (major regioisomer) and δ 4.75 (minor regioisomer). Regioisomer assignment supported by ^{1}H-^{13}C HSQC and HMBC. White solid (1.69 g, 5.6 mmol, 80%); **^{1}H NMR** (500 MHz, $CDCl_3$) δ 7.44−7.29 (m, 5H), 5.15 (d, *J* = 10.8 Hz, 1H), 3.83 (s, 3H), 3.68 (d, *J* = 10.8 Hz, 1H), 2.92−2.85 (m, 2H), 2.66−2.59 (m, 2H), 2.36−2.22 (m, 4H); **^{13}C NMR** (126 MHz, $CDCl_3$) δ 169.6, 138.6, 128.8, 128.6, 128.0, 74.9, 59.4, 52.5, 51.8, 28.3; **IR** (thin layer film) ν (cm^{-1}) = 2827, 1733, 1497, 1454, 1435, 1359, 1334, 1281, 1166, 700; **mp** = 120−122 °C; **HRMS** (ESI^+) *m/z* calculated for $C_{14}H_{19}ClNO_2S^+$ $[M+H]^+$ 300.0820, found 300.0820.

(±)-methyl 3-chloro-2-(diisobutylamino)-3-phenylpropanoate

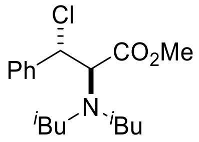

The title compound was prepared according to the general procedure **II**; the reaction was performed on 6.0 mmol scale and was allowed to proceed for 2.5 h. The compound was isolated as a >20:1 regioisomeric mixture, favoring the shown regioisomer. The regioisomeric ratio of the isolated product was determined by ^{1}H NMR spectroscopic analysis by comparison of the resonances at δ 4.33 (minor regioisomer) and δ 4.02 (major regioisomer). Regioisomer assignment supported by ^{1}H-^{13}C HSQC and HMBC. White solid (1.87 g, 5.8 mmol, 96%); **^{1}H NMR** (400 MHz, $CDCl_3$) δ 7.42−7.27 (m, 5H), 5.14 (d, *J* = 11.0 Hz, 1H), 4.02 (d, *J* = 11.0 Hz, 1H), 3.80 (s, 3H), 2.17 (dd, *J* = 12.9, 5.3 Hz, 1H), 2.06 (dd, *J* = 12.9, 9.1 Hz, 1H), 1.65−1.49 (m, 2H), 0.75 (d, *J* = 6.6 Hz, 6H), 0.49 (d, *J* = 6.6 Hz, 6H); **^{13}C NMR** (101 MHz, $CDCl_3$) δ 170.4, 138.8, 128.8, 128.7, 128.5, 69.7, 60.2, 59.8, 51.4, 26.3, 20.8, 20.5; **IR** (thin layer film) ν (cm^{-1}) = 2954, 2869, 1737, 1456, 1434, 1390, 1192, 1162, 1087, 1052; **mp** = 65−67 °C; **HRMS** (ESI^+) *m/z* calculated for $C_{18}H_{29}ClNO_2^+$ $[M+H]^+$ 326.1881, found 326.1881.

(±)-methyl 2-(dibenzylamino)-3-chloro-3-phenylpropanoate

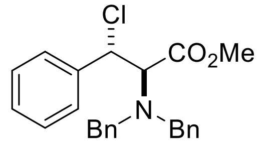

The title compound was prepared according to the general procedure **II**; the reaction was performed on 8.0 mmol scale and was allowed to proceed for 3 h. The crude mixture was purified by silica gel chromatography using a gradient of 20% EtOAc/pentane. The compound was isolated as a >20:1 regioisomeric mixture, favoring the shown regioisomer. The regioisomeric ratio of the isolated product was determined by ^{1}H NMR spectroscopic analysis by comparison of the resonances at δ 5.29 (major regioisomer) and δ 4.93 (minor regioisomer). Regioisomer assignment supported by ^{1}H-^{13}C HSQC and HMBC. White solid (2.79 g, 7.1 mmol, 89%); **^{1}H NMR** (500 MHz, $CDCl_3$) δ 7.43−7.39 (m, 1H), 7.32 (t, *J* = 7.7 Hz, 2H), 7.25−7.18 (m, 6H), 7.07−7.02 (m, 2H), 6.91 (dd, *J* = 6.8, 2.8 Hz, 4H), 5.29 (d, *J* = 11.0 Hz, 1H), 3.96 (d, *J* = 10.5 Hz, 1H, overlapping with signal at δ 3.95), 3.95 (s, 3H, overlapping with signal at δ 3.96), 3.81 (d, *J* = 13.6 Hz, 2H), 3.27 (d, *J* = 13.7 Hz, 2H); **^{13}C NMR** (126 MHz, $CDCl_3$) δ 170.1, 138.1, 138.0, 129.3, 128.9, 128.8, 128.5, 128.2, 127.4, 66.8, 60.1, 54.7, 51.7; **IR** (thin layer film) ν (cm^{-1}) = 3030, 1733, 1495, 1454, 1433, 1373, 1264, 1230, 1192, 607; **mp** = 131−133 °C; **HRMS** (ESI^+) *m/z* calculated for $C_{24}H_{25}ClNO_2^+$ $[M+H]^+$ 394.1568, found 394.1567.

(±)-methyl 3-chloro-3-phenyl-2-(4-phenylpiperazin-1-yl)propanoate

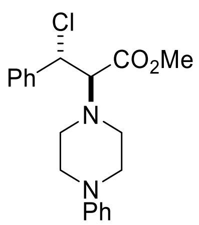

The title compound was prepared according to the general procedure **II**; the reaction was performed on 8.0 mmol scale and was allowed to proceed for 2.5 h. The compound was isolated as a 12.6:1 regioisomeric mixture, favoring the shown regioisomer. The regioisomeric ratio of the isolated product was determined by ^{1}H NMR spectroscopic analysis by comparison of the resonances at δ 5.21 (major regioisomer) and δ 4.82 (minor regioisomer). Regioisomer assignment supported by ^{1}H-^{13}C HSQC and HMBC. White solid (1.89 g, 5.3 mmol, 66%); **^{1}H NMR** (400 MHz, $CDCl_3$) δ 7.42−7.28 (m, 5H), 7.24−7.17 (m, 2H), 6.86−6.77 (m, 3H), 5.21 (d, *J* = 10.8 Hz, 1H), 3.85 (s, 3H, overlapping with signal at δ 3.83), 3.83 (d, *J* = 10.3 Hz, 1H, overlapping with signal at δ 3.85), 2.94−2.74 (m, 6H), 2.59−2.51 (m, 2H); **^{13}C NMR** (101 MHz, $CDCl_3$) δ 169.4, 151.3, 138.6, 129.1, 128.7, 128.5, 128.0, 120.0, 116.2, 73.6, 59.5, 51.7, 49.9, 49.6; **IR** (thin layer film) ν (cm^{-1}) = 2827, 1734, 1599, 1496, 1452, 1384,

1342, 1235, 1153, 1010; **mp** = 107−109 °C; **HRMS** (ESI$^+$) *m/z* calculated for $C_{20}H_{24}ClN_2O_2^+$ [M+H]$^+$ 359.1521, found 359.1515.

(±)-methyl 3-chloro-2-(4-(2-((2,4-dimethylphenyl)thio)phenyl)piperazin-1-yl)-3-phenylpropanoate

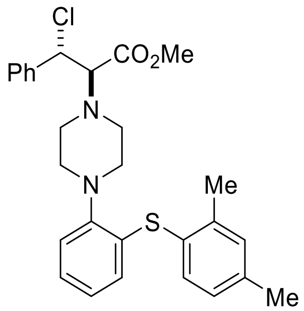

The title compound was prepared according to the general procedure **II**; the reaction was performed on 0.7 mmol scale and was allowed to proceed for 2.5 h. The compound was isolated as a 13.6:1 regioisomeric mixture, favoring the shown regioisomer. The regioisomeric ratio of the isolated product was determined by ^{1}H NMR spectroscopic analysis by comparison of the resonances at δ 5.26 (major regioisomer) and δ 4.86 (minor regioisomer). Regioisomer assignment supported by ^{1}H-^{13}C HSQC and HMBC. White solid (228.0 mg, 0.46 mmol, 66%); **^{1}H NMR** (500 MHz, $CDCl_3$) δ 7.47−7.33 (m, 6H), 7.16 (s, 1H), 7.06−6.98 (m, 2H), 6.89 (d, *J* = 7.8 Hz, 1H), 6.84 (t, *J* = 7.8 Hz, 1H), 6.50 (dd, *J* = 7.8, 1.1 Hz, 1H), 5.26 (d, *J* = 10.8 Hz, 1H), 3.90 (s, 3H, overlapping with signal at δ 3.88), 3.88 (dd, *J* = 14.7, 10.9 Hz, 1H, overlapping with signal at δ 3.90), 2.90−2.79 (m, 4H), 2.79−2.71 (m, 2H), 2.67-2.56 (m, 2H), 2.38 (s, 3H), 2.31 (s, 3H); **^{13}C NMR** (126 MHz, $CDCl_3$) δ 169.5, 149.1, 142.4, 139.2, 138.8, 136.2, 134.4, 131.7, 128.6, 128.5, 128.1, 128.0, 127.9, 126.4, 125.4, 124.4, 119.8, 73.7, 59.6, 51.9, 51.7, 50.2, 21.3, 20.7; **mp** = 68−70 °C; **HRMS** (ESI$^+$) *m/z* calculated for $C_{28}H_{32}ClN_2O_2S^+$ [M+H]$^+$ 495.1868, found 495.1865.

(±)-methyl 3-(4-bromophenyl)-3-chloro-2-(diallylamino)propanoate

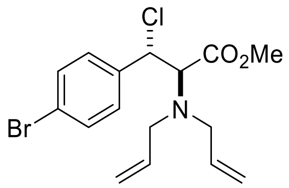

The title compound was prepared according to the general procedure **II**; the reaction was performed on 12.2 mmol scale and was allowed to proceed for 4 h. After the initial silica plug, the compound was purified via flash column chromatography using a mobile phase of 5% EtOAc/pentane. The compound was isolated as a >20:1 regioisomeric mixture, favoring the shown regioisomer. The regioisomeric ratio of the isolated product was determined by ^{1}H NMR spectroscopic analysis by comparison of the resonances at δ 4.35 (minor regioisomer) and δ 3.93 (major regioisomer). Regioisomer assignment supported by ^{1}H-^{13}C HSQC and HMBC. Low-melting white solid (4.25 g, 11.4 mmol, 93%); **^{1}H NMR** (400 MHz, $CDCl_3$) δ 7.49−7.44 (m, 2H), 7.21−7.16 (m, 2H), 5.41−5.28 (m, 2H), 5.10 (d, *J* = 10.9 Hz, 1H), 5.07−4.98 (m, 4H), 3.93 (d, *J* = 10.9 Hz, 1H), 3.81 (s, 3H), 3.28−3.19 (m, 2H), 2.80 (dd, *J* = 14.4, 8.1 Hz, 2H); **^{13}C NMR** (101 MHz, $CDCl_3$) δ 170.2, 137.9, 135.2, 131.4, 130.0, 122.5, 118.2, 67.6, 59.2, 53.5, 51.7; **IR** (thin layer film) ν (cm^{-1}) = 2824, 1734, 1643, 1594, 1489, 1434, 1419, 1165, 1072, 924; **HRMS** (ESI$^+$) *m/z* calculated for $C_{16}H_{20}BrClNO_2^+$ [M+H]$^+$ 372.0361, found 372.0360.

(±)-methyl 3-(4-chlorophenyl)-3-(diallylamino)-2-hydroxypropanoate

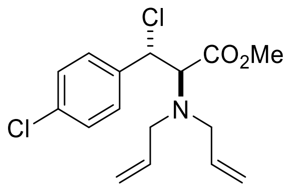

The title compound was prepared according to the general procedure **II**; the reaction was performed on 6.5 mmol scale and was allowed to proceed for 5 h. After the initial silica plug, the compound was purified via flash column chromatography using a mobile phase of 5% Et_2O/pentane. The compound was isolated as a >20:1 regioisomeric mixture, favoring the shown regioisomer. The regioisomeric ratio of the isolated product was determined by ^{1}H NMR spectroscopic analysis by comparison of the resonances at δ 4.29 (minor regioisomer) and δ 3.86 (major

regioisomer). Regioisomer assignment supported by ^{1}H-^{13}C HSQC and HMBC. White solid (1.48 g, 4.5 mmol, 70%); **^{1}H NMR** (400 MHz, $CDCl_3$) δ 7.27−7.23 (m, 2H), 7.20−7.15 (m, 2H), 5.34−5.21 (m, 2H), 5.04 (d, *J* = 10.9 Hz, 1H), 5.01−4.91 (m, 2H), 3.86 (d, *J* = 10.9 Hz, 1H), 3.75 (s, 3H), 3.21−3.13 (m, 2H), 2.73 (dd, *J* = 14.4, 8.1 Hz, 2H); **^{13}C NMR** (101 MHz, $CDCl_3$) δ 170.2, 137.4, 135.3, 134.3, 129.8, 128.5, 118.2, 67.7, 59.2, 53.5, 51.7; **IR** (thin layer film) ν (cm^{-1}) = 2952, 2823, 1734, 1643, 1598, 1493, 1434, 1418, 1357, 1166; **mp** = 43−45 °C; **HRMS** (ESI^+) *m/z* calculated for $C_{16}H_{20}Cl_2NO_2^+$ $[M+H]^+$ 328.0866, found 328.0864.

(±)-methyl 3-chloro-2-(diallylamino)-3-(*p*-tolyl)propanoate

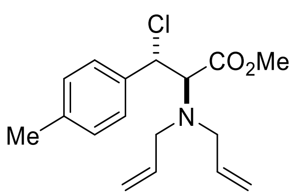

The title compound was prepared according to the general procedure **II**; the reaction was performed on 17.3 mmol scale and was allowed to proceed for 3 h. The compound was isolated as a >20:1 regioisomeric mixture, favoring the shown regioisomer. The regioisomeric ratio of the isolated product was determined by ^{1}H NMR spectroscopic analysis by comparison of the resonances at δ 4.34 (minor regioisomer) and δ 3.99 (major regioisomer). Regioisomer assignment supported by ^{1}H-^{13}C HSQC and HMBC. Orange oil (4.21 g, 13.7 mmol, 79%); **^{1}H NMR** (500 MHz, $CDCl_3$) δ 7.21 (d, *J* = 8.1 Hz, 2H), 7.15 (d, *J* = 8.0 Hz, 2H), 5.43−5.33 (m, 2H), 5.14 (d, *J* = 11.0 Hz, 1H), 5.07−4.97 (m, 4H), 3.99 (d, *J* = 11.0 Hz, 1H), 3.82 (s, 3H), 3.28-3.21 (m, 2H), 2.83 (dd, *J* = 14.4, 8.0 Hz, 2H), 2.36 (s, 3H); **^{13}C NMR** (126 MHz, $CDCl_3$) δ 170.6, 138.4, 135.7, 135.6, 129.0, 128.3, 117.9, 67.7, 60.2, 53.5, 51.6, 21.4; **IR** (thin layer film) ν (cm^{-1}) = 2981, 1735, 1643, 1516, 1434, 1419, 1357, 1266, 1163, 923; **HRMS** (ESI^+) *m/z* calculated for $C_{17}H_{23}ClNO_2^+$ $[M+H]^+$ 308.1412, found 308.1411.

(±)-methyl 4-(1-chloro-2-(diallylamino)-3-methoxy-3-oxopropyl)benzoate

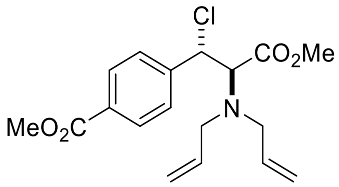

The title compound was prepared according to the general procedure **II**; the reaction was performed on 7.5 mmol scale and was allowed to proceed for 4 h. The compound was isolated as a 19.5:1 regioisomeric mixture, favoring the shown regioisomer. The regioisomeric ratio of the isolated product was determined by ^{1}H NMR spectroscopic analysis by comparison of the resonances at δ 4.43 (minor regioisomer) and δ 3.97 (major regioisomer). Regioisomer assignment supported by ^{1}H-^{13}C HSQC and HMBC. Low-melting yellow solid (2.62 g, 7.5 mmol, 99%); **^{1}H NMR** (500 MHz, $CDCl_3$) δ 8.01 (d, *J* = 8.4 Hz, 2H), 7.39 (d, *J* = 8.3 Hz, 2H), 5.35−5.24 (m, 2H), 5.16 (d, *J* = 10.9 Hz, 1H), 5.05−4.96 (m, 4H), 3.97 (d, *J* = 10.9 Hz, 1H), 3.92 (s, 3H), 3.82 (s, 3H), 3.27-3.20 (m, 2H), 2.79 (dd, *J* = 14.4, 8.2 Hz, 2H); **^{13}C NMR** (126 MHz, $CDCl_3$) δ 170.1, 166.8, 143.8, 135.2, 130.3, 129.6, 128.5, 118.2, 67.7, 59.1, 53.5, 52.3, 51.7; **IR** (thin layer film) ν (cm^{-1}) = 2981, 1726, 1643, 1614, 1435, 1418, 1359, 1280, 1167, 1112; **mp** = 55−57 °C; **HRMS** (ESI^+) *m/z* calculated for $C_{18}H_{23}ClNO_4^+$ $[M+H]^+$ 352.1310, found 352.1312.

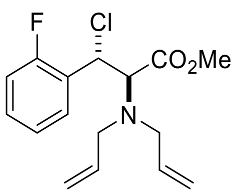

(±)-methyl 3-chloro-2-(diallylamino)-3-(2-fluorophenyl)propanoate

The title compound was prepared according to the general procedure **II**; the reaction was performed on 4.1 mmol scale and was allowed to proceed for 4 h. The compound was isolated as a >20:1 regioisomeric mixture, favoring the shown regioisomer. The regioisomeric ratio of the isolated product was determined by ^{1}H NMR spectroscopic analysis by comparison of the resonances at δ 4.63 (minor regioisomer) and δ 5.52 (major regioisomer). Regioisomer assignment supported by ^{1}H-^{13}C HSQC and

HMBC. Low melting white solid (0.99 g, 3.7 mmol, 77%); **^{1}H NMR** (500 MHz, $CDCl_3$) δ 7.38 (td, *J* = 7.5, 1.8 Hz, 1H), 7.32−7.25 (m, 1H), 7.15 (td, *J* = 7.6, 1.2 Hz, 1H), 7.07−7.00 (m, 1H), 5.52 (d, *J* = 11.0 Hz, 1H), 5.39−5.28 (m, 2H), 5.05−4.94 (m, 4H), 4.01 (d, J = 11.1 Hz, 1H), 3.83 (s, 3H), 3.31−3.25 (m, 2H), 2.79 (dd, *J* = 14.3, 8.3 Hz, 2H); **^{13}C NMR** (126 MHz, $CDCl_3$) δ 170.2, 160.6 (d, *J* = 248.5 Hz), 135.5, 130.1 (d, *J* = 8.4 Hz), 129.8 (d, J = 3.4 Hz), 126.2 (d, *J* = 13.2 Hz), 124.2 (d, *J* = 3.6 Hz), 117.9, 115.4 (d, *J* = 22.6 Hz), 67.5, 53.6, 52.8, 51.6; **^{19}F NMR** 471 MHz, $CDCl_3$) δ -117.3; **IR** (thin layer film) ν (cm^{-1}) = 2982, 2823, 1736, 1643, 1617, 1590, 1493, 1267, 1168, 755; **mp** = 42−44 °C; **HRMS** (ESI^+) *m/z* calculated for $C_{16}H_{20}ClNO_2F^+$ $[M+H]^+$ 312.1161, found 312.1159.

(±)-methyl 3-chloro-2-(diallylamino)-3-(3-(trifluoromethyl)phenyl)propanoate

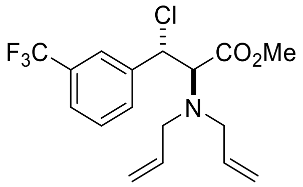

The title compound was prepared according to the general procedure **II**; the reaction was performed on 7.3 mmol scale and was allowed to proceed for 4 h. The compound was isolated as a >20:1 regioisomeric mixture, favoring the shown regioisomer. The regioisomeric ratio of the isolated product was determined by ^{1}H NMR spectroscopic analysis by comparison of the resonances at δ 4.47 (minor regioisomer) and δ 3.96 (major regioisomer). Regioisomer assignment supported by ^{1}H-^{13}C HSQC and HMBC. Low-melting white solid (25.1 g, 6.9 mmol, 95%); **^{1}H NMR** (400 MHz, $CDCl_3$) δ 7.62−7.54 (m, 2H), 7.54−7.42 (m, 2H), 5.37−5.22 (m, 2H), 5.17 (d, *J* = 11.0 Hz, 1H), 5.09−4.94 (m, 4H), 3.96 (d, *J* = 10.9 Hz, 1H), 3.83 (s, 3H), 3.27-3.22 (m, 2H), 2.80 (dd, *J* = 14.4, 8.1 Hz, 2H); **^{13}C NMR** (101 MHz, $CDCl_3$) δ 170.1, 139.9, 135.0, 131.8, 130.8 (q, *J* = 32.4 Hz), 128.8, 125.4 (q, *J* = 3.8 Hz, overlapping with signal at δ 124.1), 125.3 (q, *J* = 3.7 Hz, overlapping with signal at δ 124.1), 124.1 (q, *J* = 273.2 Hz, overlapping with signals at δ 125.4 and δ 125.3), 118.3, 67.6, 59.0, 53.5, 51.7; **^{19}F NMR** (376 MHz, $CDCl_3$) δ -62.8; **IR** (thin layer film) ν (cm^{-1}) = 2981, 1734, 1644, 1451, 1332, 1266, 1166, 1128, 1073, 926; **mp** = 49−51 °C; **HRMS** (ESI^+) *m/z* calculated for $C_{17}H_{20}ClNO_2F_3^+$ $[M+H]^+$ 362.1129, found 362.1129.

(±)-*tert*-butyl 3-chloro-2-(diallylamino)-3-phenylpropanoate

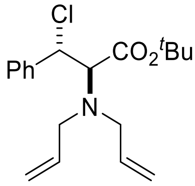

The title compound was prepared according to the general procedure **II**; the reaction was performed on 10.5 mmol scale and was allowed to proceed for 2 h. The compound was isolated as a 13.9:1 regioisomeric mixture, favoring the shown regioisomer. After the initial silica plug, the compound was purified via flash column chromatography using a mobile phase of 5% EtOAc/pentane. The regioisomeric ratio of the isolated product was determined by ^{1}H NMR spectroscopic analysis by comparison of the resonances at δ 4.34 (minor regioisomer) and δ 3.88 (major regioisomer). Regioisomer assignment supported by ^{1}H-^{13}C HSQC and HMBC. White solid (2.82 g, 8.4 mmol, 80%); **^{1}H NMR** (500 MHz, $CDCl_3$) δ 7.36−7.28 (m, 5H), 5.41−5.31 (m, 2H), 5.10 (d, *J* = 11.0 Hz, 1H), 5.05−4.97 (m, 4H), 3.88 (d, *J* = 11.0 Hz, 1H), 3.29−3.22 (m, 2H), 2.86 (dd, *J* = 14.4, 8.2 Hz, 2H), 1.56 (s, 9H); **^{13}C NMR** (126 MHz, $CDCl_3$) δ 169.1, 139.0, 135.8, 128.5, 128.4, 128.3, 117.7, 82.0, 68.2, 60.4, 53.4, 28.5; **IR** (thin layer film) ν (cm^{-1}) = 2980, 1725, 1643, 1455, 1369, 1252, 1149, 1081, 996, 955; **mp** = 77−79 °C; **HRMS** (ESI^+) *m/z* calculated for $C_{19}H_{27}ClNO_2^+$ $[M+H]^+$ 336.1725, found 336.1726.

(±)-3-chloro-2-(diallylamino)-*N*,*N*-diethyl-3-phenylpropanamide

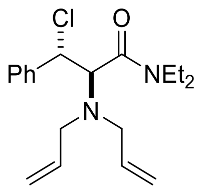

The title compound was prepared according to the general procedure **II**; the reaction was performed on 6.2 mmol scale and was allowed to proceed for 4 h; 1.3 equiv. triethylamine and methanesulfonyl chloride were used. The compound was isolated as a 13.2:1 regioisomeric mixture, favoring the shown regioisomer. The regioisomeric ratio of the isolated product was determined by ^{1}H NMR spectroscopic analysis by comparison of the resonances at δ 4.71 (minor regioisomer) and δ 4.16 (major regioisomer). Regioisomer assignment supported by ^{1}H-^{13}C HSQC and HMBC. Yellow solid (1.80 g, 5.4 mmol, 86%); **^{1}H NMR** (500 MHz, $CDCl_3$) δ 7.44−7.39 (m, 2H), 7.39−7.30 (m, 3H), 5.44−5.33 (m, 3H), 4.99−4.88 (m, 4H), 4.16 (d, *J* = 10.4 Hz, 1H), 3.72−3.61 (m, 2H), 3.32−3.22 (m, 2H), 3.14 (dd, *J* = 15.1, 5.2 Hz, 2H), 3.00 (dd, *J* = 15.1, 6.8 Hz, 2H), 1.30 (t, *J* = 7.2 Hz, 3H), 1.17 (t, *J* = 7.1 Hz, 3H); **^{13}C NMR** (126 MHz, $CDCl_3$) δ 169.2, 139.8, 136.9, 128.7, 128.6, 128.3, 116.7, 63.5, 61.4, 53.1, 42.0, 40.5, 14.8, 13.2; **IR** (thin layer film) ν (cm^{-1}) = 1634, 1433, 1357, 1273, 1142, 1118, 999, 918, 804, 722; **mp** = 64−66 °C; **HRMS** (ESI$^+$) *m/z* calculated for $C_{19}H_{28}ClN_2O^+$ [M+H]$^+$ 335.1885, found 335.1884.

(±)-methyl 3-chloro-2-(diallylamino)-3-(naphthalen-2-yl)propanoate

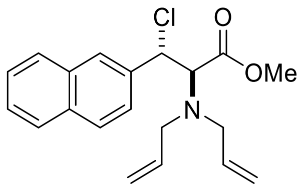

The title compound was prepared according to the general procedure **II**; the reaction was performed on 6.8 mmol scale and was allowed to proceed for 3 h. The compound was isolated as a >20:1 regioisomeric mixture, favoring the shown regioisomer. The regioisomeric ratio of the isolated product was determined by ^{1}H NMR spectroscopic analysis by comparison of the resonances at δ 4.58 (minor regioisomer) and δ 4.15 (major regioisomer). Regioisomer assignment supported by ^{1}H-^{13}C HSQC and HMBC. White solid (2.1 g, 6.1 mmol, 90%); **^{1}H NMR** (500 MHz, $CDCl_3$) δ 7.89−7.80 (m, 3H), 7.76 (s, 1H), 7.55−7.46 (m, 3H), 5.41−5.28 (m, 3H), 5.06−4.97 (m, 4H), 4.15 (d, *J* = 10.9 Hz, 1H), 3.87 (s, 3H), 3.32−3.25 (m, 2H), 2.86 (dd, *J* = 14.3, 8.1 Hz, 2H); **^{13}C NMR** (126 MHz, $CDCl_3$) δ 170.5, 136.0, 135.5, 133.4, 133.0, 128.3, 128.1, 128.0, 127.8, 126.6, 126.4, 125.4, 118.0, 67.5, 60.5, 53.5, 51.7; **IR** (thin layer film) ν (cm^{-1}) = 3060, 2951, 2822, 1734, 1643, 1510, 1434, 1347, 1191, 924; **mp** = 59−61 °C; **HRMS** (ESI$^+$) *m/z* calculated for $C_{20}H_{23}ClNO_2^+$ [M+H]$^+$ 344.1412, found 344.1410.

(±)-methyl 3-chloro-2-(diallylamino)-3-phenylpropanoate (*cis* diastereomer)

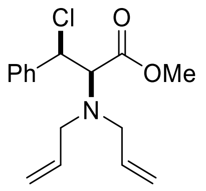

The title compound was prepared according to the general procedure **II**; the reaction was performed on 4.4 mmol scale and was allowed to proceed for 3.5 h. The compound was isolated as a 13.6:1 regioisomeric mixture, favoring the shown regioisomer. The regioisomeric ratio of the isolated product was determined by ^{1}H NMR spectroscopic analysis by comparison of the resonances at δ 4.30 (minor regioisomer) and δ 3.94 (major regioisomer). Regioisomer assignment supported by ^{1}H-^{13}C HSQC and HMBC. Yellow oil (433.8 mg, 1.5 mmol, 34%); **^{1}H NMR** (500 MHz, $CDCl_3$) δ 7.39−7.26 (m, 5H), 5.92-5.80 (m, 2H), 5.31−5.23 (m, 2H), 5.20−5.13 (m, 3H), 3.94 (d, *J* = 10.6 Hz, 1H), 3.60−3.52 (m, 2H), 3.44 (s, 3H), 3.06 (dd, *J* = 14.5, 7.8 Hz, 2H); **^{13}C NMR** (126 MHz, $CDCl_3$) δ 170.1, 138.7, 136.2, 128.8, 128.7, 128.2, 117.8, 68.3, 61.2, 53.7, 51.3; **IR** (thin layer film) ν (cm^{-1}) = 3077, 2821, 1731, 1643, 1495, 1434, 1419, 1322, 1157, 922; **HRMS** (ESI$^+$) *m/z* calculated for $C_{16}H_{21}ClNO_2^+$ [M+H]$^+$ 294.1255, found 294.1256.

(±)-methyl 3-cyclohexyl-2-(dibenzylamino)-3-((methylsulfonyl)oxy)propanoate

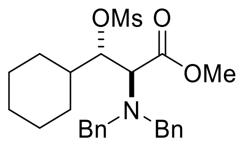

The title compound was prepared according to the general procedure from the corresponding amino alcohol (which has been previously reported[13]); the reaction was performed on 1.57 mmol scale and was allowed to proceed for 4.5 h. The compound was isolated as a single regioisomer. Regioisomer assignment supported by ^{1}H-^{13}C HSQC and HMBC. White solid (300 mg, 0.64 mmol, 41%); **^{1}H NMR** (400 MHz, $CDCl_3$) δ 7.40–7.24 (m, 10H), 5.00 (d, *J* = 10.6, 1H), 3.93 (d, *J* = 13.5 Hz, 2H), 3.86 (s, 3H), 3.59 (d, *J* = 10.6 Hz, 1H), 3.36 (d, *J* = 13.6 Hz, 2H), 2.92 (s, 3H), 2.10–1.96 (m, 1H), 1.84–1.72 (m, 1H), 1.73–1.48 (m, 4H), 1.42–1.23 (m, 2H), 1.16–0.99 (m, 2H), 0.90–0.78 (m, 1H); **^{13}C NMR** (101 MHz, $CDCl_3$) δ 170.5, 138.4, 129.4, 128.6, 127.6, 85.3, 61.1, 55.5, 51.6, 39.0, 38.0, 31.1, 26.9, 26.3, 26.2, 24.6; **IR** (thin layer film) ν (cm^{-1}) = 3659, 2981, 1732, 1453, 1381, 1348, 1252, 1200, 1173, 932; **mp** = 133–135 °C; **HRMS** (ESI$^+$) *m/z* calculated for $C_{25}H_{34}NO_5S^+$ [M+H]$^+$ 460.2152, found 460.2148.

(±)-methyl 3-(diallylamino)-2-((methylsulfonyl)oxy)-5-phenylpent-4-ynoate

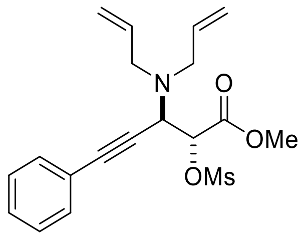

The title compound was prepared according to the general procedure **II**; the reaction was performed on 2.97 mmol scale and was allowed to proceed for 5.5 h. The compound was isolated as a 3.8:1 regioisomer mixture, favoring the shown regioisomer. The regioisomeric ratio of the isolated product was determined by ^{1}H NMR spectroscopic analysis by comparison of the resonances at δ 4.29 (major regioisomer) and δ 3.93 (minor regioisomer). Regioisomer assignment supported by ^{1}H-^{13}C HSQC and HMBC. Dark orange oil (461 mg, 1.2 mmol, 41%); **^{1}H NMR** (500 MHz, $CDCl_3$) δ 7.48–7.42 (m, 2H), 7.41–7.29 (m, 3H), 5.87–5.69 (m, 2H), 5.25–5.13 (m, 4H), 5.09 (d, *J* = 8.9 Hz, 1H), 4.29 (d, *J* = 8.9 Hz, 1H), 3.83 (s, 3H), 3.41–3.33 (m, 2H), 3.11 (s, 3H), 3.04 (dd, *J* = 14.1, 8.2 Hz, 2H); **^{13}C NMR** (126 MHz, $CDCl_3$) δ 167.7, 135.5, 132.0, 128.8, 128.5, 122.3, 118.4, 87.8, 82.5, 78.6, 55.0, 54.7, 52.8, 38.9; **IR** (thin layer film) ν (cm^{-1}) = 3660, 2981, 1756, 1643, 1491, 1443, 1365, 1179, 1029, 965; **HRMS** (ESI$^+$) *m/z* calculated for $C_{19}H_{24}NO_5S^+$ [M+H]$^+$ 378.1370, found 378.1367.

(±)-N-allyl-N-(2-chloro-2-phenylethyl)prop-2-en-1-amine

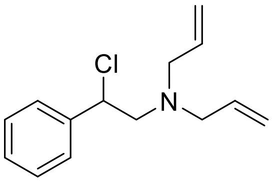

The title compound was prepared according to the general procedure **II**; the reaction was performed on 2.6 mmol scale and was allowed to proceed for 1.5 h. The compound was isolated as a >20:1 regioisomeric mixture, favoring the shown regioisomer. The regioisomeric ratio of the isolated product was determined by ^{1}H NMR spectroscopic analysis by comparison of the resonances at δ 4.91 (major regioisomer) and δ 4.10 (minor regioisomer). Regioisomer assignment supported by ^{1}H-^{13}C HSQC and HMBC. Yellow oil (447 mg, 1.9 mmol, 72%); **^{1}H NMR** (400 MHz, $CDCl_3$) δ 7.39–7.27 (m, 5H), 5.83–5.71 (m, 2H), 5.19–5.08 (m, 4H), 4.91 (t, *J* = 7.1 Hz, 1H), 3.14 (app d, *J* = 6.4 Hz, 4H), 3.06 (dd, *J* = 13.9, 7.4 Hz, 1H), 2.97 (dd, *J* = 13.8, 6.9 Hz, 1H); **^{13}C NMR** (101 MHz, $CDCl_3$) δ 140.7, 135.6, 128.6, 128.4, 127.6, 117.8, 61.6, 61.4, 57.8; **IR** (thin layer film) ν (cm^{-1}) = 3076, 2808, 1643, 1453, 1418, 1040, 919, 718, 696; **HRMS** (ESI$^+$) *m/z* calculated for $C_{14}H_{19}ClN^+$ [M+H]$^+$ 236.1201, found 236.1202.

General Procedure III for α-Fluorination with CsF

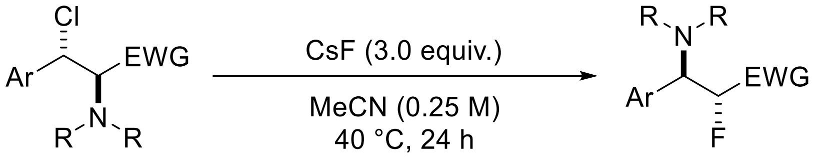

A 3.5 mL vial equipped with a stir bar was sequentially charged with the amino chloride substrate (0.25 mmol, 1.0 equiv.) and CsF (0.75 mmol, 3.0 equiv.) before adding MeCN (1.0 mL, 0.25 M). The reaction was placed in the center of a stir plate at a height of 2 cm and was stirred at 1200 rpm at 40 °C (in an oil bath) for 24 h. Et_2O was used to flush the reaction through a short plug of silica gel and the filtrate was concentrated *in vacuo*. The crude material thusly obtained was purified using flash column chromatography.

Characterization Data for α–Fluoro Amines:

(±)-methyl 3-(diallylamino)-2-fluoro-3-phenylpropanoate (2a)

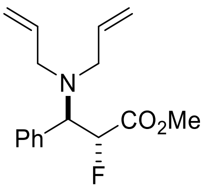

The title compound was prepared according to the general procedure **III**. The compound was formed in a 19.8:1 regioisomeric mixture, favoring the shown regioisomer. The crude product was purified using flash column chromatography, with a gradient from 100:0 to 94:6 pentane/Et_2O to obtain the product as a colorless oil (37.2 mg, 83% yield). **^{1}H NMR** (400 MHz, $CDCl_3$) δ 7.39–7.29 (m, 5H), 5.81–5.71 (m, 2H), 5.45 (dd, *J* = 49.4, 6.7 Hz, 1H), 5.20–5.14 (m, 4H), 4.32 (dd, *J* = 22.3, 6.7 Hz, 1H), 3.73 (s, 3H), 3.33–3.27 (m, 2H), 2.84 (dd, *J* = 14.3, 7.7 Hz, 2H); **^{13}C NMR** (101 MHz, $CDCl_3$) δ 169.3 (d, *J* = 22.9 Hz), 136.1, 134.2, 129.3, 128.3, 128.0, 117.7, 89.4 (d, *J* = 189.6 Hz), 63.9 (d, *J* = 22.8 Hz), 53.5, 52.2; **^{19}F NMR** (376 MHz, $CDCl_3$) δ -197.1; **IR** (thin layer film) ν (cm^{-1}) = 2981, 2819, 1760, 1643, 1496, 1439, 1419, 1293, 1204, 922; **HRMS** (ESI^+) *m/z* calculated for $C_{16}H_{21}NO_2F^+$ $[M+H]^+$ 278.1551, found 278.1549.

(±)-*tert*-butyl 3-(diallylamino)-2-fluoro-3-phenylpropanoate (2b)

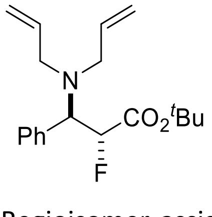

The title compound was prepared according to the general procedure **III** but was allowed to proceed under 48 h. The compound was formed in a >20:1 regioisomeric mixture, favoring the shown regioisomer. The crude product was purified using flash column chromatography, with a gradient from 100:00 to 94:6 pentane/Et_2O to obtain the product as a white solid (55.6 mg, 70% yield). Regioisomer assignment supported by ^{1}H-^{13}C HSQC and HMBC. *On 11.0 g scale*: A 250 mL round-bottom flask was sequentially charged with amino chloride (11.0 g, 32.8 mmol, 1.0 equiv.), CsF (14.92 g, 98.3 mmol, 3.0 equiv.), and MeCN (131 mL, 0.25 M). The reaction was stirred at 40 °C under an atmosphere of N_2 for 72 h. Once per day on subsequent days, the solids which accumulated on the sides of the flask were scraped into solution using a metal spatula, which was then rinsed into the reaction with 2 mL MeCN. Upon reaching completion, the reaction mixture was passed through a pad of silica gel with diethyl ether and concentrated *in vacuo*. The crude product was purified using flash column chromatography, with a gradient from 100:0 to 94:6 pentane/Et_2O to obtain the product as a

white solid (9.42 g, 90% yield). **^{1}H NMR** (500 MHz, $CDCl_3$) δ 7.37–7.27 (m, 5H), 5.87–5.75 (m, 2H), 5.30 (dd, *J* = 49.7, 6.1 Hz, 1H), 5.21–5.11 (m, 4H), 4.30 (dd, *J* = 24.2, 6.1 Hz, 1H), 3.32 (dd, *J* = 14.3, 5.1 Hz, 2H), 2.88 (dd, *J* = 14.3, 7.5 Hz, 2H), 1.39 (s, 9H); **^{13}C NMR** (126 MHz, $CDCl_3$) δ 168.0 (d, *J* = 22.7 Hz), 136.4, 134.6, 129.8 (d, *J* = 1.3 Hz), 128.2, 127.9, 117.6, 89.7 (d, *J* = 190.2 Hz), 82.5, 64.0 (d, *J* = 21.8 Hz), 53.7, 28.0; **^{19}F NMR** (470 MHz, $CDCl_3$) δ -196.4; **IR** (thin layer film) ν (cm^{-1}) = 2820, 1750, 1643, 1455, 1369, 1313, 1259, 1162, 995, 920; **mp** = 40–42 °C; **HRMS** (ESI^+) *m/z* calculated for $C_{19}H_{27}NO_2F^+$ $[M+H]^+$ 320.2020, found 320.2021.

(±)-3-(diallylamino)-*N*,*N*-diethyl-2-fluoro-3-phenylpropanamide (2c)

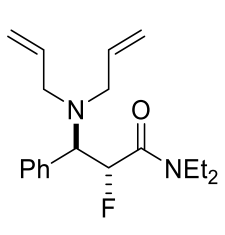

The title compound was prepared according to the general **III**. The compound was formed in a >20:1 regioisomeric mixture, favoring the shown regioisomer. The regioisomeric ratio of the crude product was determined by ^{1}H NMR spectroscopic analysis by comparison of the resonances at δ 4.34 (major regioisomer) and δ 5.77 (minor regioisomer). Regioisomer assignment supported by ^{1}H-^{13}C HSQC and HMBC. The crude product was purified using flash column chromatography, with a gradient from 90:10 to 75:25 pentane/Et_2O to obtain the product as a clear oil (54.9 mg, 69% yield). **^{1}H NMR** (500 MHz, $CDCl_3$) δ 7.37–7.27 (m, 5H), 5.91–5.71 (m, 2H), 5.57 (dd, *J* = 49.6, 7.2 Hz, 1H), 5.22–5.07 (m, 4H), 4.47 (dd, *J* = 16.9, 7.2 Hz, 1H), 3.45–3.00 (m, 6H), 2.83 (dd, *J* = 14.5, 7.8 Hz, 2H), 1.13 (t, *J* = 7.0 Hz, 3H), 1.09 (t, *J* = 7.1 Hz, 3H); **^{13}C NMR** (126 MHz, $CDCl_3$) δ 167.1 (d, *J* = 19.5 Hz), 136.2, 135.0, 129.7, 128.1, 127.8, 117.4, 88.7 (d, *J* = 188.5 Hz), 64.3 (d, *J* = 22.3 Hz), 53.8, 41.3 (d, *J* = 6.4 Hz), 41.1, 14.7, 12.6; **^{19}F NMR** (471 MHz, $CDCl_3$) δ -190.3; **IR** (thin layer film) ν (cm^{-1}) = 2978, 1652, 1488, 1454, 1362, 1362, 1271, 1219, 1086, 1033, 998, 919, 922, 809, 704; **HRMS** (ESI^+) *m/z* calculated for $C_{19}H_{27}FN_2ONa^+$ $[M+Na]^+$ 341.2000., found 341.2000.

(±)-methyl 3-(diallylamino)-2-fluoro-3-(*p*-tolyl)propanoate (2d)

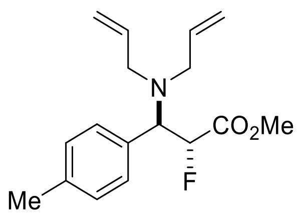

The title compound was prepared according to the general procedure **III** but was allowed to proceed for 48 h. The compound was formed in a >20:1 regioisomeric mixture, favoring the shown regioisomer. The regioisomeric ratio of the crude product was determined by ^{1}H NMR spectroscopic analysis by comparison of the resonances at δ 4.21 (major regioisomer) and δ 5.60 (minor regioisomer). Regioisomer assignment supported by ^{1}H-^{13}C HSQC and HMBC. The crude product was purified using flash column chromatography, with a gradient from 100:0 to 95:5 pentane/Et_2O to obtain the product as a colorless oil (56.3 mg, 91% yield). **^{1}H NMR** (500 MHz, $CDCl_3$) δ 7.22 (d, *J* = 8.2 Hz, 2H), 7.18 (d, *J* = 8.1 Hz, 2H), 5.83–5.72 (m, 2H), 5.44 (dd, *J* = 49.4, 6.8 Hz, 1H), 5.22–5.13 (m, 4H), 4.31 (dd, *J* = 22.0, 6.8 Hz, 1H), 3.74 (s, 3H), 3.35–3.27 (m, 2H), 2.84 (dd, *J* = 14.3, 7.8 Hz, 2H), 2.35 (s, 3H); **^{13}C NMR** (126 MHz, $CDCl_3$) δ 169.4 (d, *J* = 23.2 Hz), 137.8, 136.3, 131.1, 129.3, 129.1, 117.6, 89.5 (d, *J* = 188.0 Hz), 63.7 (d, *J* = 23.2 Hz), 53.6, 52.2, 21.2; **^{19}F NMR** (470 MHz, $CDCl_3$) δ -196.7; **IR** (thin layer film) ν (cm^{-1}) = 2821, 1762, 1439, 1294, 1202, 1114, 1064, 1022, 978, 810, 731; **HRMS** (ESI^+) *m/z* calculated for $C_{17}H_{22}FNO_2Na^+$ $[M+Na]^+$ 314.1527, found 314.1519.

(±)-methyl 3-(4-bromophenyl)-3-(diallylamino)-2-fluoropropanoate (2e)

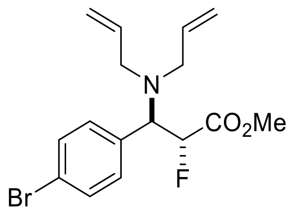

The title compound was prepared according to the general procedure **III** but was allowed to proceed for 48 h. The compound was formed in a >20:1 regioisomeric mixture, favoring the shown regioisomer. The regioisomeric ratio of the crude product was determined by ^{1}H NMR spectroscopic analysis by comparison of the resonances at δ 4.21 (major regioisomer) and δ 5.58 (minor regioisomer). Regioisomer assignment supported by ^{1}H-^{13}C HSQC and HMBC. The crude product was purified using flash column chromatography, with a gradient from 100:0 to 96:4 pentane/Et_2O to obtain the product as a clear oil (77.0 mg, 86% yield). **^{1}H NMR** (500 MHz, $CDCl_3$)) δ 7.48 (d, *J* = 8.5 Hz, 2H), 7.20 (d, *J* = 8.5 Hz 2H), 5.85–5.68 (m, 2H), 5.41 (dd, *J* = 49.4, 6.0 Hz, 1H), 5.23–5.11 (m, 4H), 4.29 (dd, *J* = 23.7, 6.0 Hz, 1H), 3.73 (s, 3H), 3.61–3.09 (m, 2H), 2.87 (dd, *J* = 14.4, 7.5 Hz 2H).); **^{13}C NMR** (101 MHz, $CDCl_3$) δ 169.1 (d, *J* = 22.7 Hz), 135.9, 133.7, 131.6, 131.1 (d, *J* = 1.8 Hz), 122.28, 118.0, 89.2 (d, *J* = 190.3 Hz), 63.6 (d, *J* = 21.8 Hz), 53.6, 52.5; **^{19}F NMR** (470 MHz, $CDCl_3$) δ -198.0; **IR** (thin layer film) ν (cm^{-1}) = 2954, 1761, 1489, 1439, 1292, 1207, 1105, 1075, 1012, 923, 809; **HRMS** (ESI^+) *m/z* calculated for $C_{16}H_{19}BrFNO_2Na^+$ $[M+Na]^+$ 378.0475, found 378.0491.

(±)-methyl 3-(4-chlorophenyl)-3-(diallylamino)-2-fluoropropanoate (2f)

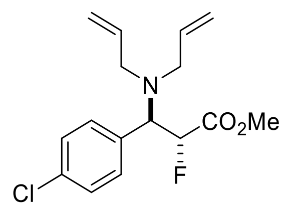

The title compound was prepared according to the general procedure **III** but was allowed to proceed for 48 h. The compound was formed in a >20:1 regioisomeric mixture, favoring the shown regioisomer. The regioisomeric ratio of the crude product was determined by ^{1}H NMR spectroscopic analysis by comparison of the resonances at δ 4.20 (major regioisomer) and δ 5.60 (minor regioisomer). Regioisomer assignment supported by ^{1}H-^{13}C HSQC and HMBC. The crude product was purified using flash column chromatography, with a gradient from 100:0 to 96:4 pentane/Et_2O to obtain the product as a clear oil (66.3 mg, 85% yield). **^{1}H NMR** (500 MHz, $CDCl_3$) δ 7.33 (d, *J* = 8.6 Hz, 2H), 7.26 (d, *J* = 8.6 Hz, 2H), 5.90–5.61 (m, 2H), 5.42 (dd, *J* = 49.4, 6.0 Hz, 1H), 5.22–5.12 (m, 4H), 4.30 (dd, *J* = 23.7, 6.0 Hz, 1H), 3.72 (s, 3H), 3.33–3.23 (m, 2H), 2.88 (dd, *J* = 14.3, 7.3 Hz, 2H); **^{13}C NMR** (101 MHz, $CDCl_3$) δ 169.2 (d, *J* = 22.7 Hz), 135.9, 134.1, 133.2, 130.8 (d, *J* = 1.8 Hz), 128.7, 118.0, 89.3 (d, *J* = 190.3 Hz), 63.6 (d, *J* = 21.8 Hz), 53.6, 52.5; **^{19}F NMR** (470 MHz, $CDCl_3$) δ -198.0; **IR** (thin layer film) ν (cm^{-1}) = 2954, 2822, 1761, 1493, 1439, 1292, 1207, 1095, 1016, 924, 854, 754; **HRMS** (ESI^+) *m/z* calculated for $C_{16}H_{19}ClFNO_2Na^+$ $[M+Na]^+$ 334.0981, found 334.0978.

(±)-methyl 4-(1-(diallylamino)-2-fluoro-3-methoxy-3-oxopropyl)benzoate (2g)

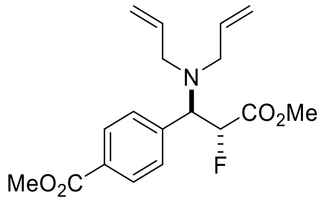

The title compound was prepared according to the general procedure **III** but was allowed to proceed for 48 h. The compound was formed in a 11.2:1 regioisomeric mixture, favoring the shown regioisomer. The regioisomeric ratio of the crude product was determined by ^{1}H NMR spectroscopic analysis by comparison of the resonances at δ 4.31 (major regioisomer) and δ 5.67 (minor regioisomer). Regioisomer assignment supported by ^{1}H-^{13}C HSQC and HMBC. The crude product was purified using flash column chromatography, with a gradient from 95:5 to 90:100 pentane/Et_2O to obtain the product as a white solid (50.8 mg, 61% yield). **^{1}H NMR** (400 MHz, $CDCl_3$) δ 8.09–8.00 (m, 2H), 7.49–7.40 (m, 2H), 5.89–5.65 (m, 2H), 5.48 (dd, *J* = 49.3, 6.3 Hz, 1H), 5.22–5.12 (m, 4H), 4.40 (dd, *J* = 23.3, 6.3 Hz, 1H), 3.93 (s, 3H), 3.74 (s, 3H), 3.41–3.24 (m, 2H), 2.90 (dd, *J* = 14.3, 7.5 Hz, 2H); **^{13}C NMR** (101 MHz, $CDCl_3$) δ 169.0

(d, J = 22.7 Hz), 166.8, 139.8, 135.7, 129.9, 129.5, 129.3 (d, J = 1.8 Hz), 117.9, 88.9 (d, J = 190.3 Hz), 63.8 (d, J = 21.8 Hz), 53.5, 52.3, 52.2; **^{19}F NMR** (376 MHz, $CDCl_3$) δ -196.7; **IR** (thin layer film) ν (cm^{-1}) = 3082, 3010, 2848, 1751, 1717, 1592, 1437, 1289, 1209, 1113, 1019, 933, 714; **mp** = 47–48 °C; **HRMS** (ESI^+) *m/z* calculated for $C_{18}H_{22}FNO_4Na^+$ $[M+Na]^+$ 358.1425, found 358.1433.

(±)-methyl 3-(diallylamino)-2-fluoro-3-(3-(trifluoromethyl)phenyl)propanoate (2h)

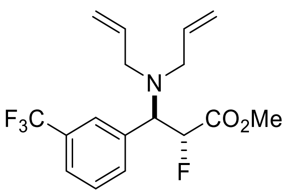

The title compound was prepared according to the general procedure **III** but was allowed to proceed for 48 h. The compound was formed in a >20:1 regioisomeric mixture, favoring the shown regioisomer. The regioisomeric ratio of the crude product was determined by ^{1}H NMR spectroscopic analysis by comparison of the resonances at δ 2.40 (major regioisomer) and δ 2.85 (minor regioisomer). Regioisomer assignment supported by ^{1}H-^{13}C HSQC and HMBC. The crude product was purified using flash column chromatography, with a gradient from 98:2 to 95:5 pentane/Et_2O to obtain the product as a clear oil (79.1 mg, 92% yield). **^{1}H NMR** (500 MHz, $CDCl_3$ δ 7.63–7.53 (m, 3H), 7.52–7.45 (m, 1H), 5.86–5.70 (m, 2H), 5.47 (dd, J = 49.3, 5.9 Hz, 1H), 5.23–5.13 (m, 4H), 4.40 (dd, J = 23.7, 5.8 Hz, 1H), 3.73 (s, 3H), 3.34–3.24 (m, 2H), 2.91 (dd, J = 14.4, 7.3 Hz, 2H); **^{13}C NMR** (126 MHz, $CDCl_3$) δ 169.0 (d, J = 19.5 Hz), 135.9, 135.6, 132.6, 131.8 (q, J = 31.5 Hz), 129.0 126.2, 125.0, 124.2 (q, J = 272.8 Hz), 118.2, 88.9 (d, J = 191.2 Hz), 64.0 (d, J = 21.8 Hz), 53.6, 52.5; **^{19}F NMR** (376 MHz, $CDCl_3$) δ -62.7, -198.1; **IR** (thin layer film) ν (cm^{-1}) = 2957, 1762, 1449, 1343, 1167, 1128, 1075, 998, 924, 806, 707; **HRMS** (ESI^+) *m/z* calculated for $C_{17}H_{19}F_4NO_2Na^+$ $[M+H]^+$ 368.1244, found 368.1251.

(±)-methyl 3-(diallylamino)-2-fluoro-3-(2-fluorophenyl)propanoate (2i)

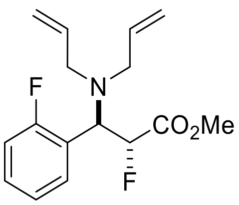

The title compound was prepared according to the general procedure **III** but was allowed to proceed for 48 h. The compound was formed in a >20:1 regioisomeric mixture, favoring the shown regioisomer. The regioisomeric ratio of the crude product was determined by ^{1}H NMR spectroscopic analysis by comparison of the resonances at δ 4.59 (major regioisomer) and δ 6.00 (minor regioisomer). Regioisomer assignment supported by ^{1}H-^{13}C HSQC and HMBC. The crude product was purified using flash column chromatography, with a gradient from 100:0 to 96:4 pentane/Et_2O to obtain the product as a colorless oil (49.8 mg, 68% yield). **^{1}H NMR** (500 MHz, $CDCl_3$)) δ 7.47–7.38 (m, 1H), 7.31 (app dddd, J = 8.2, 7.1, 5.3, 1.8 Hz, 1H), 7.16 (app td, J = 7.5, 1.3 Hz, 1H), 7.08 (app ddd, J = 10.6, 8.3, 1.3 Hz, 1H), 5.81–5.67 (m, 2H), 5.47 (dd, J = 49.2, 7.4 Hz, 1H), 5.23–5.10 (m, 4H), 4.67 (dd, J = 19.9, 7.5 Hz, 1H), 3.76 (s, 3H), 3.37–3.26 (m, 2H), 2.86 (dd, J = 14.3, 7.6 Hz 2H); **^{13}C NMR** (126 MHz, $CDCl_3$) δ 169.2 (d, J = 23.2 Hz), 161.7 (d, J = 246.6 Hz), 136.1, 131.3 (dd, J = 4.5, 2.3 Hz), 129.9 (d, J = 8.6 Hz), 124.1 (d, J = 3.2 Hz), 121.6 (d, J = 15.0 Hz), 117.7, 115.9 (d, J = 23.6 Hz), 89.4 (dd, J = 187.6, 2.7 Hz), 58.3 (d, J = 23.6 Hz), 53.9, 52.4; **^{19}F NMR** (470MHz, $CDCl_3$) δ -114.7, -195.7; **IR** (thin layer film) ν (cm^{-1}) = 2836, 1761, 1490, 1455, 1295, 1220, 1103, 1065, 924, 760; **HRMS** (ESI^+) *m/z* calculated for $C_{16}H_{19}F_2NO_2Na^+$ $[M+Na]^+$ 218.1276, found 318.1269.

(±)-methyl 3-(diallylamino)-2-fluoro-3-(naphthalen-2-yl)propanoate (2j)

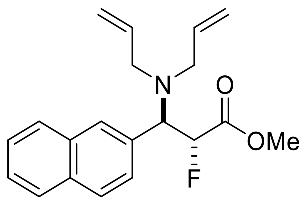

The title compound was prepared according to the general procedure **III** but was allowed to proceed for 48 h. The compound was formed in a >20:1 regioisomeric mixture, favoring the shown regioisomer. The regioisomeric ratio of the crude product was determined by quantitative ^{19}F NMR spectroscopic analysis by comparison of the resonances at δ -197.3 (major regioisomer) and δ -178.1 (minor regioisomer). Regioisomer assignment supported by ^{1}H-^{13}C HSQC and HMBC. The crude product was purified using flash column chromatography, with a gradient from 98:2 to 95:5 pentane/Et_2O to obtain the product as a colorless oil (72.2 mg, 88% yield). **^{1}H NMR** (500 MHz, $CDCl_3$) δ 7.90–7.80 (m, 3H), 7.78 (s, 1H), 7.53–7.45 (m, 3H), 5.88–5.73 (m, 2H), 5.56 (dd, *J* = 49.4, 6.2 Hz, 1H), 5.24–5.14 (m, 4H), 4.50 (dd, *J* = 23.5, 6.2 Hz, 1H), 3.72 (s, 3H), 3.41–3.30 (m, 2H), 2.95 (dd, *J* = 14.3, 7.3 Hz, 2H); **^{13}C NMR** (126 MHz, $CDCl_3$) δ 169.4 (d, *J* = 22.7 Hz), 136.2, 133.2, 133.2, 132.3, 128.7, 128.2, 128.1, 127.7, 127.2, 126.3, 126.3, 117.8, 89.5 (d, *J* = 189.8 Hz), 64.3 (d, *J* = 21.8 Hz), 53.7, 52.4; **^{19}F NMR** (376 MHz, $CDCl_3$) δ -197.3; **IR** (thin layer film) ν (cm^{-1}) = 2954, 1759, 1439, 1292, 1217, 1065, 1021, 922, 822, 751; **HRMS** (ESI$^+$) *m/z* calculated for $C_{20}H_{22}FNO_2Na^+$ $[M+Na]^+$ 350.1527, found 350.1538.

(±)-methyl 3-(diallylamino)-2-fluoro-5-phenylpent-4-ynoate (2k)

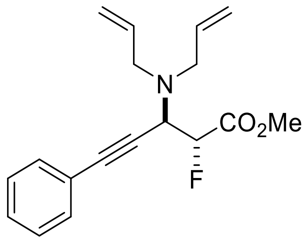

The title compound was prepared according to the general procedure **III** but was carried out at 23 °C. The compound was formed in a 9.1:1 regioisomeric mixture, favoring the shown regioisomer.The regioisomeric ratio of the crude product was determined by quantitative ^{19}F NMR spectroscopic analysis by comparison of the resonances at δ -193.7 (α-fluoro, major regioisomer) and δ -177.8 (β-fluoro, minor regioisomer). Regioisomer assignment supported by ^{1}H-^{13}C HSQC and HMBC. The crude product was purified using flash column chromatography, with a gradient from 100:0 to 94:6 pentane/Et_2O to obtain the product as a colorless oil (22.5 mg, 29% yield). **^{1}H NMR** (500 MHz, $CDCl_3$) δ 7.51–7.42 (m, 2H), 7.37–7.27 (m, 3H), 5.92–5.63 (m, 2H), 5.30–5.14 (m, 4H), 5.06 (dd, *J* = 49.2, 6.3 Hz, 1H), 4.30 (dd, *J* = 20.1, 6.3 Hz, 1H), 3.83 (s, 3H), 3.42–3.32 (m, 2H), 3.07 (dd, *J* = 14.2, 7.9 Hz, 2H); **^{13}C NMR** (126 MHz, $CDCl_3$) 168.3 (d, *J* = 23.6 Hz), 135.8, 132.1, 128.6, 128.4, 122.7, 118.1, 90.9 (d, *J* = 190.4 Hz), 87.6, 82.1, 55.2 (d, *J* = 2.2 Hz), 54.9 (d, *J* = 23.3 Hz), 52.6; **^{19}F NMR** (471 MHz, $CDCl_3$) δ -194.0; **IR** (thin layer film) ν (cm^{-1}) = 3081, 1761, 1491, 1443, 1289, 1205 1070, 924, 758, 692; **HRMS** (ESI$^+$) *m/z* calculated for $C_{18}H_{20}FNO_2Na^+$ $[M+Na]^+$ 324.1370, found 324.1358.

(±)-methyl 3-(diisobutylamino)-2-fluoro-3-phenylpropanoate (2m)

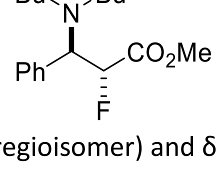

The title compound was prepared according to the general procedure **III**. The compound was formed in a 7.6:1 regioisomeric mixture, favoring the shown regioisomer. The regioisomeric ratio of the crude product was determined by ^{1}H NMR spectroscopic analysis by comparison of the resonances at δ 4.22 (major regioisomer) and δ 5.57 (minor regioisomer). Regioisomer assignment supported by ^{1}H-^{13}C HSQC and HMBC. The crude product was purified using flash column chromatography, with a gradient from 100:0 to 98:2 pentane/Et_2O to obtain the product as a low-melting white solid (58.9 mg, 76% yield). The spectra contain 3% of **3m** (β-F regioisomer) due to challenge in isolation on flash column chromatography. **^{1}H NMR** (500 MHz, $CDCl_3$^{1}H NMR (500 MHz, $CDCl_3$) δ 7.81–6.89 (m, 5H), 5.42 (dd, *J*

= 49.0, 6.4 Hz, 1H), 4.32 (dd, *J* = 22.6, 6.6 Hz, 1H), 3.73 (s, 3H), 2.21 (dd, *J* = 13.0, 8.9 Hz, 2H), 2.12 (dd, *J* = 12.9, 5.6 Hz, 2H), 1.77 (m, 2H), 0.91 (d, *J* = 6.6 Hz, 6H), 0.82 (d, *J* = 6.7 Hz, 6H); **^{13}C NMR** (126 MHz, $CDCl_3$) δ 169.6 (d, *J* = 22.7 Hz), 134.6, 129.9 (d, *J* = 2.3 Hz), 128.1, 127.8, 89.6 (d, *J* = 188.5 Hz), 64.7, 60.0 (d, *J* = 1.4 Hz), 52.4, 26.4, 21.0, 20.8; **^{19}F NMR** (470 MHz, $CDCl_3$) δ -194.9; **IR** (thin layer film) ν (cm^{-1}) = 2961, 1748, 1458, 1383, 1209, 1090, 1052, 978, 763, 705; **HRMS** (ESI^+) *m/z* calculated for $C_{18}H_{28}FNO_2Na^+$ $[M+Na]^+$ 332.1996, found 332.2010.

(±)-methyl 3-(dibenzylamino)-2-fluoro-3-phenylpropanoate (2n)

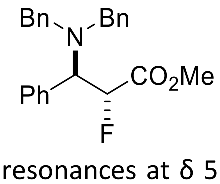

The title compound was prepared according to the general procedure **III** but was allowed to proceed for 72 h. The compound was formed in a> 20:1 regioisomeric mixture, favoring the shown regioisomer. The regioisomeric ratio of the crude product was determined by ^{1}H NMR spectroscopic analysis by comparison of the resonances at δ 5.57 (major regioisomer) and δ 5.81 (minor regioisomer). Regioisomer assignment supported by ^{1}H-^{13}C HSQC and HMBC. The crude product was purified using flash column chromatography, with pure toluene followed by a separate column with a gradient from 98:2 to 92:8 Pentane/Et_2O to obtain the product as a white solid (63.7 mg, 68% yield). **^{1}H NMR** (400 MHz, $CDCl_3$) 7.45–7.20 (m, 15H), 5.57 (dd, *J* = 49.0, 7.0 Hz, 1H), 4.28 (dd, *J* = 21.8, 7.0 Hz, 1H), 3.84 (d, *J* = 13.7 Hz, 2H), 3.72 (s, 3H), 3.32 (d, *J* = 13.7 Hz, 2H); **^{13}C NMR** (101 MHz, $CDCl_3$) δ 169.2 (d, *J* = 22.9 Hz), 139.2, 133.8, 129.8 (d, *J* = 1.3 Hz), 129.0, 128.5, 128.5, 128.2, 127.3, 89.5 (d, *J* = 188.9 Hz), 63.1 (d, *J* = 22.9 Hz), 54.8, 52.4; **^{19}F NMR** (470 MHz, $CDCl_3$) δ -195.4; **IR** (thin layer film) ν (cm^{-1}) = 3030, 2360, 2342, 1751, 1495, 1455, 1295, 1218, 1097, 1028, 751, 700; **mp** = 70–72 °C; **HRMS** (ESI^+) *m/z* calculated for $C_{24}H_{25}FNO_2^+$ $[M+H]^+$ 378.1864, found 378.1875.

(±)-methyl 2-fluoro-3-morpholino-3-phenylpropanoate (2o)

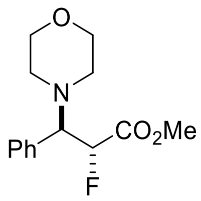

The title compound was prepared according to the general procedure **III** but was allowed to proceed for 48 h. The compound was formed in a >20:1 regioisomeric mixture, favoring the shown regioisomer. The regioisomeric ratio of the crude product was determined by ^{1}H NMR spectroscopic analysis by comparison of the resonances at δ 5.38 (major regioisomer) and δ 5.66 (minor regioisomer). Regioisomer assignment supported by ^{1}H-^{13}C HSQC and HMBC. The crude product was purified using flash column chromatography, with a gradient from 90:10 to 75:25 pentane/Et_2O, to obtain the product as a colorless oil (60.1 mg, 90% yield). **^{1}H NMR** (400 MHz, $CDCl_3$) δ 7.39–7.27 (m, 5H), 5.44 (dd, *J* = 49.8, 4.6 Hz, 1H), 3.80 (dd, *J* = 26.0, 4.6 Hz, 1H), 3.73–3.66 (m, 4H), 3.66 (s, 3H), 2.54–2.48 (m, 4H); **^{13}C NMR** (126 MHz, $CDCl_3$) δ 169.1 (d, *J* = 22.7 Hz), 134.3, 129.5, 128.6, 128.5, 88.3 (d, *J* = 193.9 Hz), 70.9 (d, *J* = 20.0 Hz), 67.2, 52.4, 51.3; **^{19}F NMR** (376MHz, $CDCl_3$) δ -200.6. **IR** (thin layer film) ν (cm^{-1}) = 2960, 2856, 2361, 1763, 1453, 1293, 1153, 1118, 1070, 1007, 808, 758; **HRMS** (ESI^+) *m/z* calculated for $C_{14}H_{18}FNO_3Na^+$ $[M+Na]^+$ 290.1163, found 290.1172.

(±)-methyl 2-fluoro-3-phenyl-3-thiomorpholinopropanoate (2p)

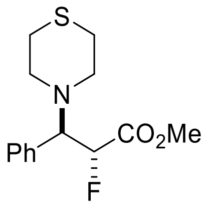

The title compound was prepared according to the general procedure **III** but was allowed to proceed for 48 h. The compound was formed in a >20:1 regioisomeric mixture, favoring the shown regioisomer. The regioisomeric ratio of the crude product was determined by ^{1}H NMR spectroscopic analysis by comparison of the resonances at δ 5.45 (major regioisomer) and δ 5.72 (minor regioisomer).

Regioisomer assignment supported by ^1H-^{13}C HSQC and HMBC. The crude product was purified using flash column chromatography, with a gradient from 95:5 to 88:12 pentane/Et_2O to obtain the product as a white solid (64.3 mg, 90% yield). **^{1}H NMR** (500 MHz, $CDCl_3$) δ 7.38–7.27 (m, 5H), 5.44 (dd, *J* = 49.4, 6.4 Hz, 1H), 4.00 (dd, *J* = 22.6, 6.4 Hz, 1H), 3.74 (s, 3H), 2.90–2.80 (m, 2H), 2.77–2.68 (m, 2H), 2.67–2.55 (m, 4H); **^{13}C NMR** (126 MHz, $CDCl_3$) δ 169.4 (d, *J* = 22.7 Hz), 133.9, 129.2, 128.5, 128.4 (d, *J* = 5.9 Hz), 88.9 (d, *J* = 190.3 Hz), 71.0 (d, *J* = 22.3 Hz), 52.9, 52.5, 28.5; **^{19}F NMR** (376MHz, $CDCl_3$) δ -199.5; **IR** (thin layer film) ν (cm^{-1}) = 2957, 1742, 1452, 1375, 1282, 1258, 1223, 1095, 975, 811, 728, 706; **mp** = 77–79 °C; **HRMS** (ESI^+) *m/z* calculated for $C_{14}H_{18}FNO_2SNa^+$ $[M+Na]^+$ 206.0935, found 306.0932.

(±)-methyl 2-fluoro-3-phenyl-3-(4-phenylpiperazin-1-yl)propanoate (2q)

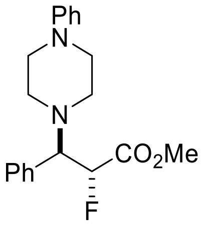

The title compound was prepared according to the general procedure **III** but was allowed to proceed for 48 h. The compound was formed in a 19.7:1 regioisomeric mixture, favoring the shown regioisomer. The regioisomeric ratio of the crude product was determined by ^1H NMR spectroscopic analysis by comparison of the resonances at δ 5.41 (major regioisomer) and δ 5.69 (minor regioisomer). Regioisomer assignment supported by ^1H-^{13}C HSQC and HMBC. The crude product was purified using flash column chromatography, with a gradient from 95:5 to 85:15 pentane/Et_2O to obtain the product as a white solid (77.7 mg, 91% yield). **^{1}H NMR** (500 MHz, $CDCl_3$) δ 7.39–7.30 (m, 5H), 7.29–7.20 (m, 2H), 6.93–6.81 (m, 3H), 5.50 (dd, *J* = 49.6, 5.3 Hz, 1H), 3.93 (dd, *J* = 25.1, 5.1 Hz, 1H), 3.68 (s, 3H), 3.22–3.11 (m, 4H), 2.75–2.61 (m, 4H); **^{13}C NMR** (126 MHz, $CDCl_3$) 169.2 (d, *J* = 22.7 Hz), 151.3, 134.3, 129.5, 129.5, 129.2, 128.5, 120.0, 116.2, 88.7 (d, *J* = 192.6 Hz), 70.4, 52.4, 50.8, 49.6; **^{19}F NMR** (470 MHz, $CDCl_3$) δ -199.5; **IR** (thin layer film) ν (cm^{-1}) = 2956, 2927, 1762, 1601, 1451, 1260, 1223, 1108, 1005, 761, 722; **mp** = 94–96 °C; **HRMS** (ESI^+) *m/z* calculated for $C_{20}H_{23}FN_2O_2Na^+$ $[M+Na]^+$ 365.1636, found 365.1636.

(±)-methyl 3-(4-(2-((2,4-dimethylphenyl)thio)phenyl)piperazin-1-yl)-2-fluoro-3-phenylpropanoate (2r)

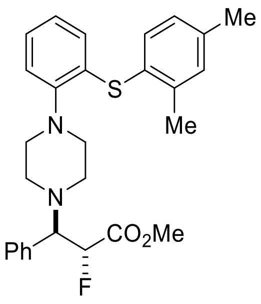

The title compound was prepared according to the general procedure **III** but was allowed to proceed for 72 h and was performed on 0.15 mmol scale (scaling all components by 60%). The compound was formed in a >20:1 regioisomeric mixture, favoring the shown regioisomer. The regioisomeric ratio of the crude product was determined by ^1H NMR spectroscopic analysis by comparison of the resonances at δ 5.46 (major regioisomer) and δ 5.72 (minor regioisomer). Regioisomer assignment supported by ^1H-^{13}C HSQC and HMBC. The crude product was purified using flash column chromatography, with a gradient from 95:5 to 85:15 pentane/Et_2O to obtain the product as a white solid (21.6 mg, 30% yield). **^{1}H NMR** (500 MHz, $CDCl_3$) δ 7.39–7.29 (m, 5H), 7.21–6.75 (m, 6H), 6.49 (d, *J* = 7.5 Hz 1H), 5.57 (dd, *J* = 49.5, 5.1 Hz, 1H), 3.93 (dd, *J* = 25.5, 5.1 Hz, 1H), 3.68 (s, 3H), 3.07 (br, 4H), 2.72 (br, 4H), 2.34 (s, 3H), 2.27 (s, 3H); **^{13}C NMR** (126 MHz, $CDCl_3$) δ 169.3 (d, *J* = 22.2 Hz), 149.3, 142.5, 139.3, 136.3, 134.8, 134.6, 131.8, 129.5, 128.5, 128.4, 128.1, 127.9, 126.4, 125.6, 124.5, 119.8, 88.7 (d, *J* = 193.3 Hz), 70.8 (d, *J* = 20.7 Hz), 52.4, 52.0, 51.3, 21.3, 20.7; **^{19}F NMR** (471 MHz, $CDCl_3$) δ -200.1; **IR** (thin layer film) ν (cm^{-1}) = 2953, 2825, 1765, 1581, 1473, 1452, 1300, 1226, 1104, 1044, 912, 818, 710; **HRMS** (ESI^+) *m/z* calculated for $C_{28}H_{32}FN_2O_2S^+$ $[M+H]^+$ 479.2163, found 479.2166.

General Procedure IV (a) for β-Fluorination with CsF under HBPTC

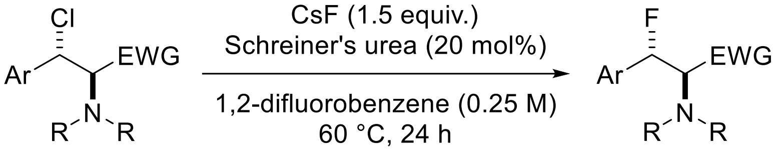

A 3.5 mL vial equipped with a stir bar was sequentially charged with the amino chloride substrate (0.25 mmol, 1.0 equiv.), Schreiner's urea (0.05 mmol, 20 mol %), and CsF (1.5 equiv.) before adding 1,2-difluorobenzene (1.0 mL, 0.25M). The reaction was placed in the center of a stir plate at a height of 2 cm and was stirred at 1200 rpm at the temperature listed below in an oil bath for 24 h. Et_2O was used to flush the reaction through a short plug of silica gel and the filtrate was concentrated *in vacuo*. The crude material thusly obtained was purified using flash column chromatography.

General Procedure IV (b) for β-Fluorination with KF under HBPTC

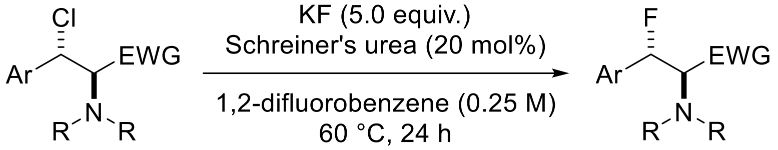

A 3.5 mL vial equipped with a stir bar was sequentially charged with the amino chloride substrate (0.25 mmol, 1.0 equiv.), Schreiner's urea (0.05 mmol, 20 mol %), and KF (5.0 equiv.) before adding 1,2-difluorobenzene (1.0 mL, 0.25M). The reaction was placed in the center of a stir plate at a height of 2 cm and was stirred at 1200 rpm at the temperature listed below in an oil bath for 24 h. Et_2O was used to flush the reaction through a short plug of silica gel and the filtrate was concentrated *in vacuo*. The crude material thusly obtained was purified using flash column chromatography.

Note: In some cases (noted below), removal of the catalyst from the desired β-fluorinated product proved challenging. In these instances, the following procedure was applied. By complexing the residual catalyst with the acetate anion, chromatographic separation between the residual catalyst and the desired product improved.

Catalyst removal procedure (optional): After flushing the reaction through a silica plug, the crude reaction mixture was concentrated as above. The crude residue was dissolved in a small amount of DCM (~2 mL) and tetrabutylammonium acetate (0.5 equiv., 37.7 mg) was added. After stirring for 30 minutes at room temperature, the crude reaction mixture was concentrated once again and purified by flash column chromatography.

Characterization Data for β-Fluoro Amines:

(±)-methyl 2-(diallylamino)-3-fluoro-3-phenylpropanoate (*trans* diastereomer) (3a)

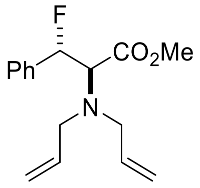

With CsF, the title compound was prepared according to the general procedure **IV (a)**, but was allowed to proceed for 72 hours. The compound was formed in a >20:1 regioisomeric mixture, favoring the shown regioisomer. The regioisomeric ratio of the crude product was determined by ^{1}H NMR spectroscopic analysis by comparison of the resonances at δ 5.63 (major regioisomer) and δ 4.25 (minor regioisomer). Regioisomer assignment supported by ^{1}H-^{13}C HSQC and HMBC. The crude product was purified using flash column chromatography, with a gradient from 100:0 to 96:4 pentane/Et_2O to obtain the product as a colorless oil (36.0 mg, 52% yield).
With KF, the title compound was prepared according to the general procedure **IV (b)**. The compound was formed in a >20:1 regioisomeric mixture, favoring the shown regioisomer. The crude product was purified using flash column chromatography, with a gradient from 100:0 to 96:4 pentane/Et_2O to obtain the product as a yellow oil (52.2 mg, 75% yield). **^{1}H NMR** (500 MHz, $CDCl_3$) δ 7.40−7.30 (m, 5H), 5.70 (dd, *J* = 45.4, 9.2 Hz, 1H), 5.48−5.36 (m, 2H), 5.07−4.99 (m, 4H), 3.84 (dd, *J* = 10.4, 9.3 Hz, 1H), 3.80 (s, 3H), 3.34−3.26 (m, 2H), 2.95 (dd, *J* = 14.5, 7.9 Hz, 2H); **^{13}C NMR** (126 MHz, $CDCl_3$) δ 170.8, 137.5 (d, *J* = 19.1 Hz), 135.6, 128.9 (d, *J* = 2.4 Hz), 128.2, 127.2 (d, *J* = 6.4 Hz), 117.8, 92.4 (d, *J* = 171.9 Hz), 65.7 (d, *J* = 32.1 Hz), 54.0, 51.5; **^{19}F NMR** (470 MHz, $CDCl_3$) δ -178.9; **IR** (thin layer film) ν (cm^{-1}) = 3071, 2824, 1736, 1643, 1435, 1342, 1273, 1193, 1169, 981; **HRMS** (ESI$^+$) *m/z* calculated for $C_{16}H_{21}NO_2F^+$ [M+H]$^+$ 278.1551, found 278.1552.

(±)-*tert*-butyl 2-(diallylamino)-3-fluoro-3-phenylpropanoate (3b)

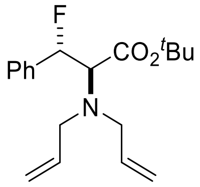

With CsF, the title compound was prepared according to the general procedure **IV (a)** but was allowed to proceed for 48 h. The compound was formed in a 5.6:1 regioisomeric mixture, favoring the shown regioisomer. The regioisomeric ratio of the crude product was determined by ^{1}H NMR spectroscopic analysis by comparison of the resonances at δ 5.60 (major regioisomer) and δ 4.21 (minor regioisomer). Regioisomer assignment supported by ^{1}H-^{13}C HSQC and HMBC. The crude product was purified using flash column chromatography, with a gradient from 100:0 to 97:3 pentane/Et_2O to obtain the product as a white solid (40.8 mg, 51% yield).
With KF, the title compound was prepared according to the general procedure **IV (b)**. The compound was formed in a 19.0:1 regioisomeric mixture, favoring the shown regioisome. The crude product was purified using flash column chromatography, with a gradient from 100:0 to 97:3 pentane/Et_2O to obtain the product as a white solid (56.7 mg, 71% yield). *On 11.0 g scale*: A 250 mL round-bottom flask was sequentially charged with amino chloride (11.0 g, 32.8 mmol, 1.0 equiv.), Schreiner's urea (1.59 g, 3.28 mmol, 10 mol %), KF (9.51 g, 163.8 mmol, 5.0 equiv.), and 1,2-difluorobenzene (131 mL, 0.25 M). The reaction was stirred at 60 °C under an atmosphere of N_2 for 72 h, then passed through a pad of silica gel with diethyl ether and concentrated *in vacuo*. The crude product was purified using flash column chromatography, with a gradient from 100:0 to 97.5:2.5 pentane/Et_2O to obtain the product as a white solid (8.28 g, 79% yield). **^{1}H NMR** (500 MHz, $CDCl_3$) δ 7.39−7.29 (m, 5H), 5.66 (dd, *J* = 45.5, 9.0 Hz, 1H), 5.50−5.38 (m, 2H), 5.09−4.98 (m, 4H), 3.73 (dd, *J* = 11.3 Hz, 9.1 Hz, 1H), 3.36−3.28 (m, 2H), 2.99 (dd, *J* = 14.4, 8.0 Hz, 2H), 1.53 (s, 9H); **^{13}C NMR** (126 MHz, $CDCl_3$) δ 169.5, 137.9 (d, *J* = 19.1 Hz), 136.0, 128.7 (d, *J* = 1.8 Hz), 128.1, 127.2 (d, *J* = 6.8 Hz), 117.6, 92.6 (d, *J* = 171.2 Hz), 81.8, 66.2 (d, *J* =

32.1 Hz), 54.0, 28.5; **^{19}F NMR** (471 MHz, $CDCl_3$) δ -179.3; **IR** (thin layer film) ν (cm^{-1}) = 2980, 1718, 1455, 1346, 1302, 1147, 977, 961, 921, 760, 694; **mp** = 51−53 °C; **HRMS** (ESI^+) *m/z* calculated for $C_{15}H_{19}FNO_2^+$ $[M-{}^tBu+2H]^+$ 264.1394, found 264.1395.

(±)-2-(diallylamino)-*N*,*N*-diethyl-3-fluoro-3-phenylpropanamide (3c)

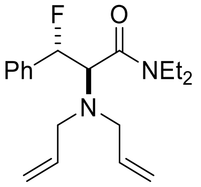

With CsF, the title compound was prepared according to the general procedure **IV (a)** but was allowed to proceed for 48 h. The compound was formed in a 4.0:1 regioisomeric mixture, favoring the shown regioisomer. The regioisomeric ratio of the crude product was determined by ^{1}H NMR spectroscopic analysis by comparison of the resonances at δ 5.77 (major regioisomer) and δ 4.34 (minor regioisomer). Regioisomer assignment supported by ^{1}H-^{13}C HSQC and HMBC. The crude product was purified using flash column chromatography, with a gradient from 90:10 to 80:20 pentane/Et_2O to obtain the product as a colorless oil (30.2 mg, 38% yield).

With KF, the title compound was prepared according to the general procedure **IV (b)** but was allowed to proceed for 48 h. The compound was formed in a >20:1 regioisomeric mixture, favoring the shown regioisomer. The crude product was purified using flash column chromatography, with a gradient from 88:12 to 77.5:22.5 pentane/Et_2O to obtain the product as an off-white solid (48.7 mg, 61% yield). **^{1}H NMR** (500 MHz, $CDCl_3$) δ 7.42−7.33 (m, 5H), 5.82 (dd, *J* = 44.6, 9.3 Hz, 1H), 5.50−5.38 (m, 2H), 5.00−4.86 (m, 4H), 3.98 (app t, *J* = 8.5 Hz, 1H), 3.76−3.66 (m, 1H), 3.62−3.52 (m, 1H), 3.30−3.24 (m, 2H), 3.24−3.15 (m, 2H), 3.06 (dd, *J* = 15.2, 6.9 Hz, 2H), 1.22 (t, *J* = 7.2 Hz, 3H), 1.16 (t, *J* = 7.2 Hz, 3H); **^{13}C NMR** (126 MHz, $CDCl_3$) δ 169.7, 138.4 (d, *J* = 19.2 Hz), 136.8, 128.9 (d, *J* = 2.2 Hz), 128.2, 127.3 (d, *J* = 6.5 Hz), 116.7, 92.9 (d, *J* = 171.2 Hz), 61.3 (d, *J* = 32.1 Hz), 53.5, 42.0, 40.6, 14.8, 13.4; **^{19}F NMR** (471 MHz, $CDCl_3$) δ -177.5; **IR** (thin layer film) ν (cm^{-1}) = 2981, 1632, 1448, 1432, 1382, 1360, 1240, 1215, 1117, 990, 922, 815, 761, 700; **mp** = 50−52 °C; **HRMS** (ESI^+) *m/z* calculated for $C_{19}H_{28}N_2O^+$ $[M+H]^+$ 319.2180, found 319.2191.

(±)-methyl 2-(diallylamino)-3-fluoro-3-(*p*-tolyl)propanoate (3d)

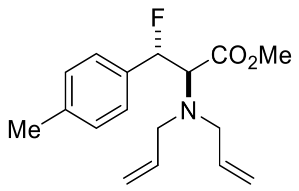

With CsF, the title compound was prepared according to the general procedure **IV (a)**. The compound was formed in a >20:1 regioisomeric mixture, favoring the shown regioisomer. The regioisomeric ratio of the crude product was determined by ^{1}H NMR spectroscopic analysis by comparison of the resonances at δ 5.60 (major regioisomer) and δ 4.21 (minor regioisomer). Regioisomer assignment supported by ^{1}H-^{13}C HSQC and HMBC. The crude product was purified using flash column chromatography, with a gradient from 100:0 to 97:3 pentane/Et_2O to obtain the product as a colorless oil (49.5 mg, 68% yield).

With KF, the title compound was prepared according to the general procedure **IV (b)**. The compound was formed in a >20:1 regioisomeric mixture, favoring the shown regioisomer. The crude product was purified using flash column chromatography, with a gradient from 100:0 to 96:4 pentane/Et_2O to obtain the product as a low-melting white solid (38.8 mg, 53% yield). **^{1}H NMR** (500 MHz, $CDCl_3$) δ 7.22 (d, *J* = 8.1 Hz, 2H), 7.18 (d, *J* = 8.0 Hz, 2H), 5.67 (dd, *J* = 45.3, 9.2 Hz, 1H), 5.50−5.39 (m, 2H), 5.08−5.00 (m, 4H), 3.85 (dd, *J* = 10.1, 9.4 Hz, 1H), 3.79 (s, 3H), 3.34−3.26 (m, 2H), 2.95 (dd, *J* = 14.5, 7.9 Hz, 2H), 2.37 (s, 3H); **^{13}C NMR** (126 MHz, $CDCl_3$) δ 171.0, 138.7 (d, *J* = 2.4 Hz), 135.7, 134.5 (d, *J* = 19.2 Hz), 128.9, 127.2 (d, *J* = 6.3 Hz), 117.7, 92.4 (d, *J* = 171.0 Hz), 65.6 (d, *J* = 32.4 Hz), 54.1, 51.5, 21.4; **^{19}F NMR**

(470 MHz, $CDCl_3$) δ -177.0. **IR** (thin layer film) ν (cm^{-1}) = 2838, 1729, 1187, 1167, 995, 976, 869, 818; **HRMS** (ESI^+) *m/z* calculated for $C_{17}H_{23}NO_2F^+$ $[M+H]^+$ 292.1707, found 292.1706.

(±)-methyl 3-(4-bromophenyl)-2-(diallylamino)-3-fluoropropanoate (3e)

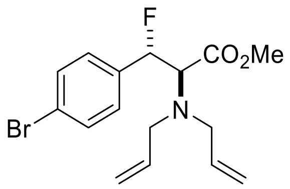

With CsF, the title compound was prepared according to the general procedure **IV (a)** but was allowed to proceed for 48 h. The compound was formed in a 9.9:1 regioisomeric mixture, favoring the shown regioisomer. The regioisomeric ratio of the crude product was determined by ^{1}H NMR spectroscopic analysis by comparison of the resonances at δ 5.58 (major regioisomer) and δ 4.21 (minor regioisomer). Regioisomer assignment supported by ^{1}H-^{13}C HSQC and HMBC. The crude product was purified using flash column chromatography, with a gradient from 100:0 to 97:3 pentane/Et_2O to obtain the product as a clear oil (65.8 mg, 74% yield).

With KF, the title compound was prepared according to the general procedure **IV (b)** but was allowed to proceed for 48 h. The compound was formed in a 12.1:1 regioisomeric mixture, favoring the shown regioisomer. The crude product was purified using flash column chromatography, with a gradient from 100:0 to 96:4 pentane/Et_2O to obtain the product as a colorless oil (60.0 mg, 67% yield). **^{1}H NMR** (500 MHz, $CDCl_3$) δ 7.50 (d, *J* = 8.1 Hz, 2H), 7.20 (d, *J* = 7.8 Hz, 2H), 5.66 (dd, *J* = 45.1, 9.2 Hz, 1H), 5.47–5.35 (m, 2H), 5.09–5.00 (m, 4H), 3.79 (s, 3H, overlapping with signal at δ 3.78), 3.78 (dd, *J* = 10.2, 9.3 Hz, 1H, overlapping with signal at δ 3.79), 3.34–3.25 (m, 2H), 2.93 (dd, *J* = 14.5, 8.1 Hz, 2H); **^{13}C NMR** (101 MHz, $CDCl_3$) δ 170.5, 136.6 (d, *J* = 19.6 Hz), 135.4, 131.4, 128.9 (d, *J* = 6.4 Hz), 122.9 (d, *J* = 2.7 Hz), 118.0, 91.7 (d, *J* = 173.1 Hz), 65.5 (d, *J* = 31.7 Hz), 54.1, 51.6; **^{19}F NMR** (470 MHz, $CDCl_3$) δ -179.8. **IR** (thin layer film) ν (cm^{-1}) = 2952, 1734, 1489, 1434, 1268, 1193, 1169, 1089, 1172, 1107, 925, 774; **HRMS** (ESI^+) *m/z* calculated for $C_{16}H_{20}NO_2BrF^+$ $[M+H]^+$ 356.0656, found 356.0656.

(±)-methyl 3-(4-chlorophenyl)-2-(diallylamino)-3-fluoropropanoate (3f)

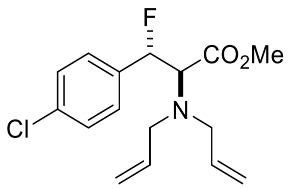

With CsF, the title compound was prepared according to the general procedure **IV (a)** but was allowed to proceed for 48 h. The compound was formed in a >20:1 regioisomeric mixture, favoring the shown regioisomer. The regioisomeric ratio of the crude product was determined by ^{1}H NMR spectroscopic analysis by comparison of the resonances at δ 5.60 (major regioisomer) and δ 4.22 (minor regioisomer). Regioisomer assignment supported by ^{1}H-^{13}C HSQC and HMBC. The crude product was purified using flash column chromatography, with a gradient from 100:0 to 97:3 pentane/Et_2O to obtain the product as a clear oil (55.3 mg, 71% yield).

With KF, the title compound was prepared according to the general procedure **IV (b)** but was allowed to proceed for 48 h. The compound was formed in a >20:1 regioisomeric mixture, favoring the shown regioisomer. The crude product was purified using flash column chromatography, with a gradient from 100:0 to 96:4 pentane/Et_2O to obtain the product as a colorless oil (62.5 mg, 80% yield). **^{1}H NMR** (500 MHz, $CDCl_3$) δ 7.35 (d, *J* = 8.2 Hz, 2H), 7.26 (d, *J* = 7.8 Hz, 2H), 5.67 (dd, *J* = 45.1, 9.3 Hz, 1H), 5.46–5.36 (m, 2H), 5.09–5.00 (m, 4H), 3.79 (s, 3H, overlapping with signal at δ 3.78), 3.78 (dd, *J* = 10.2, 9.3 Hz, 1H, overlapping with signal at δ 3.79), 3.33–3.26 (m, 2H), 2.93 (dd, *J* = 14.5, 8.0 Hz, 2H); **^{13}C NMR** (101 MHz, $CDCl_3$) δ 170.6, 136.1 (d, *J* = 19.7 Hz), 135.4, 134.7 (d, *J* = 2.6 Hz), 128.6 (d, *J* = 6.5 Hz), 128.4, 118.0, 91.6 (d, *J* = 172.9 Hz), 65.6 (d, *J* = 31.8 Hz), 54.1, 51.6; **^{19}F NMR** (470 MHz, $CDCl_3$) δ -179.4; **IR**

(thin layer film) ν (cm^{-1}) = 2953, 2840, 1735, 1494, 1268, 1193, 1169, 1089, 979, 923, 824; **HRMS** (ESI^+) *m/z* calculated for $C_{16}H_{20}NO_2ClF^+$ $[M+H]^+$ 312.1161, found 312.1160.

(±)-methyl 4-(2-(diallylamino)-1-fluoro-3-methoxy-3-oxopropyl)benzoate (3g)

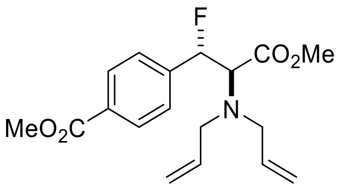

With CsF, the title compound was prepared according to the general procedure **IV (a)** but was allowed to proceed for 48 h. The compound was formed in a 1.5:1 regioisomeric mixture, favoring the shown regioisomer. The regioisomeric ratio of the crude product was determined by ^{1}H NMR spectroscopic analysis by comparison of the resonances at δ 5.67 (major regioisomer) and δ 4.31 (minor regioisomer). Regioisomer assignment supported by ^{1}H-^{13}C HSQC and HMBC. The crude product was purified using flash column chromatography, with a gradient from 100:0 to 92:8 pentane/Et_2O to obtain the product as an off-white solid (34.2 mg, 41% yield).

With KF, the title compound was prepared according to the general procedure **IV (b)** but was carried out at 80 °C and was allowed to proceed for 48 h. The compound was formed in a >20:1 regioisomeric mixture, favoring the shown regioisomer. The crude product was purified using flash column chromatography, with a gradient from 100:0 to 92:8 pentane/Et_2O to obtain the product as an off-white solid (64.7 mg, 77% yield). **^{1}H NMR** (400 MHz, $CDCl_3$) δ 8.03 (d, *J* = 8.0 Hz, 2H), 7.39 (d, *J* = 8.1 Hz, 2H), 5.74 (dd, *J* = 45.4, 9.1 Hz, 1H), 5.44–5.32 (m, 2H), 5.07–4.98 (m, 4H), 3.92 (s, 3H), 3.80 (dd, *J* = 10.7, 9.1 Hz, 1H, overlapping with signal at δ 3.78), 3.78 (s, 3H, overlapping with signal at δ 3.80), 3.33–3.25 (m, 2H), 2.92 (dd, *J* = 14.5, 8.0 Hz, 2H); **^{13}C NMR** (101 MHz, $CDCl_3$) δ 170.4, 166.9, 142.5 (d, *J* = 19.1 Hz), 135.3, 130.5 (d, *J* = 1.7 Hz), 129.4, 127.1 (d, *J* = 6.7 Hz), 118.0, 91.7 (d, *J* = 173.9 Hz), 65.6 (d, *J* = 31.1 Hz), 54.1, 52.3, 51.6; **^{19}F NMR** (376 MHz, $CDCl_3$) δ -182.0; **IR** (thin layer film) ν (cm^{-1}) = 3080, 3004, 2852, 1731, 1710, 1437, 1286, 1172, 1107, 925, 774; **mp** = 76–78 °C**;** **HRMS** (ESI^+) *m/z* calculated for $C_{18}H_{23}NO_4F^+$ $[M+H]^+$ 336.1606, found 336.1605.

(±)-methyl 2-(diallylamino)-3-fluoro-3-(3-(trifluoromethyl)phenyl)propanoate (3h)

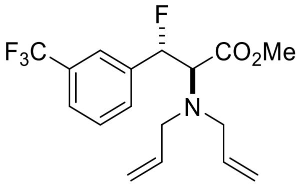

With CsF, the title compound was prepared according to the general procedure **IV (a)** but was allowed to proceed for 48 h. The compound was formed in a 1.5:1 regioisomeric mixture, favoring the shown regioisomer. The regioisomeric ratio of the crude product was determined by ^{1}H NMR spectroscopic analysis by comparison of the resonances at δ 2.85 (major regioisomer) and δ 2.40 (minor regioisomer). Regioisomer assignment supported by ^{1}H-^{13}C HSQC and HMBC. The crude product was purified using flash column chromatography, with pure toluene to obtain the product as a clear oil (32.6 mg, 38% yield).

With KF, the title compound was prepared according to the general procedure **IV (b)** but was carried out at 70 °C and allowed to proceed for 72 h. The compound was formed in a >20:1 regioisomeric mixture, favoring the shown regioisomer. The crude product was purified using flash column chromatography, with pure toluene to obtain the product as a colorless oil (67.1 mg, 78% yield). **^{1}H NMR** (500 MHz, $CDCl_3$) δ 7.67–7.59 (m, 2H), 7.57–7.48 (m, 2H), 5.77 (dd, *J* = 45.1, 9.2 Hz, 1H), 5.47–5.28 (m, 2H), 5.09–5.01 (m, 4H), 3.85–3.79 (m, 4H, overlapping methyl ester and α-H), 3.39–3.22 (m, 2H), 2.95 (dd, *J* = 14.5, 7.9 Hz, 2H); **^{13}C NMR** (126 MHz, $CDCl_3$) δ 170.4, 138.6 (d, *J* = 19.5 Hz), 135.2, 130.6 (q, *J* = 32.4 Hz, overlapping with signal at δ 130.5), 130.5 (d, *J* = 6.3 Hz, overlapping with signal at δ 130.6), 128.6, 125.7–125.5 (m), 124.3–124.1 (m), 124.2 (q, *J* = 272.4 Hz), 118.1, 91.4 (d, *J* = 173.5

Hz), 65.5 (d, J = 30.9 Hz), 54.1, 51.7; **^{19}F NMR** (376 MHz, $CDCl_3$) δ -62.8, -181.3; **IR** (thin layer film) ν (cm^{-1}) = 2959, 1725, 1327, 1161, 1120, 1072, 977, 923, 802, 698; **HRMS** (ESI^+) *m/z* calculated for $C_{17}H_{20}NO_2F_4^+$ $[M+H]^+$ 346.1425, found 346.1423.

(±)-methyl 2-(diallylamino)-3-fluoro-3-(2-fluorophenyl)propanoate (3i)

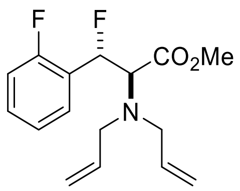

With CsF, the title compound was prepared according to the general procedure **IV (a)** but was allowed to proceed for 48 h. The compound was formed in a >20:1 regioisomeric mixture, favoring the shown regioisomer. The regioisomeric ratio of the crude product was determined by ^{1}H NMR spectroscopic analysis by comparison of the resonances at δ 6.00 (major regioisomer) and δ 4.59 (minor regioisomer). Regioisomer assignment supported by ^{1}H-^{13}C HSQC and HMBC. The crude product was purified using flash column chromatography, with a gradient from 100:0 to 97:3 pentane/Et_2O to obtain the product as a yellow oil (49.9 mg, 68% yield).

With KF, the title compound was prepared according to the general procedure **IV (b)** but was allowed to proceed for 48 h. The compound was formed in a >20:1 regioisomeric mixture, favoring the shown regioisomer. The crude product was purified using flash column chromatography, with a gradient from 100:0 to 96:4 pentane/Et_2O to obtain the product as a yellow oil (59.1 mg, 80% yield). **^{1}H NMR** (500 MHz, $CDCl_3$) δ 7.39−7.29 (m, 2H), 7.17 (app t, *J* = 7.5 Hz, 1H), 7.05 (app t, *J* = 8.9 Hz, 1H), 6.08 (dd, *J* = 45.0, 9.4 Hz, 1H), 5.44−5.33 (m, 2H), 5.07−4.99 (m, 4H), 3.88 (dd, *J* = 10.9, 9.6 Hz, 1H), 3.80 (s, 3H), 3.35−3.29 (m, 2H), 2.90 (dd, *J* = 14.2, 8.3 Hz, 2H); **^{13}C NMR** (126 MHz, $CDCl_3$) δ 170.6, 160.7 (dd, *J* = 248.1, 5.2 Hz), 135.7, 130.5 (dd, *J* = 8.5, 1.8 Hz), 128.3 (dd, *J* = 5.8, 3.8 Hz), 125.2 (dd, *J* = 20.5, 12.9 Hz), 124.1 (d, *J* = 3.4 Hz), 117.9, 115.3 (d, *J* = 21.9 Hz), 86.6 (dd, *J* = 172.0, 2.2 Hz), 65.0 (d, *J* = 31.5 Hz), 54.2, 51.6; **^{19}F NMR** (470MHz, $CDCl_3$) δ -117.9, -184.9; **IR** (thin layer film) ν (cm^{-1}) = 2818, 1724, 1198, 1178, 996, 977, 921, 771; **HRMS** (ESI^+) *m/z* calculated for $C_{16}H_{20}NO_2F_2^+$ $[M+H]^+$ 296.1457, found 296.1456.

(±)-methyl 2-(diallylamino)-3-fluoro-3-(naphthalen-2-yl)propanoate (3j)

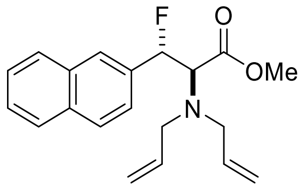

With CsF, the title compound was prepared according to the general procedure **IV (a)** but was allowed to proceed for 48 h. The compound was formed in a >20:1 regioisomeric mixture, favoring the shown regioisomer. The regioisomeric ratio of the crude product was determined by quantitative ^{19}F NMR spectroscopic analysis by comparison of the resonances at δ -178.1 (major regioisomer) and δ -197.2 (minor regioisomer). Regioisomer assignment supported by ^{1}H-^{13}C HSQC and HMBC. The crude product was purified using flash column chromatography, with a gradient from 100:0 to 96:4 pentane/Et_2O to obtain the product as a clear oil (45.8 mg, 56% yield).

With KF, the title compound was prepared according to the general procedure **IV (b)** but was allowed to proceed for 48 h. The compound was formed in a >20:1 regioisomeric mixture, favoring the shown regioisome. The crude product was purified using flash column chromatography, with a gradient from 100:0 to 96:4 pentane/Et_2O to obtain the product as a colorless oil (46.6 mg, 57% yield). **^{1}H NMR** (500 MHz, $CDCl_3$) δ 7.90−7.84 (m, 3H), 7.80 (s, 1H), 7.55−7.49 (m, 2H), 7.49−7.45 (m, 1H), 5.89 (dd, *J* = 45.3, 9.2 Hz, 1H), 5.46−5.35 (m, 2H), 5.09−4.98 (m, 4H), 3.98 (dd, *J* = 10.3, 9.4 Hz, 1H), 3.83 (s, 3H), 3.38−3.31 (m, 2H), 2.97 (dd, *J* = 14.5, 8.0 Hz, 2H); **^{13}C NMR** (126 MHz, $CDCl_3$) δ 170.9, 135.6, 134.9 (d, *J* = 18.9 Hz), 133.6, 132.9, 128.3, 128.0, 127.9, 127.2 (d, *J* = 7.5 Hz), 126.5, 126.4, 124.3 (d, *J* = 5.3 Hz), 117.9, 92.6 (d, *J* = 171.9 Hz), 65.6 (d, *J* = 31.9 Hz), 54.1, 51.6; **^{19}F NMR** (376 MHz, $CDCl_3$) δ -178.2; **IR** (thin layer film)

ν (cm^{-1}) = 2947, 1728, 1173, 1156,983, 933, 914, 820, 781, 752; **HRMS** (ESI$^+$) *m/z* calculated for $C_{20}H_{23}NO_2F^+$ [M+H]$^+$ 328.1707, found 328.1706.

(±)-methyl 2-(diallylamino)-3-fluoro-5-phenylpent-4-ynoate (3k)

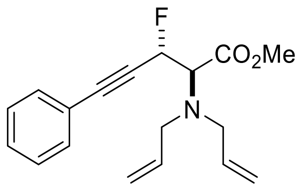

With CsF, the title compound was prepared according to the general procedure **IV (a)** but was carried out at 23 °C. Elevating the temperature was found to favor the α-fluorinated regioisomer in this case. The compound was formed in a 1.1:1 α-fluoro/β-fluoro regioisomeric mixture. The regioisomeric ratio of the crude product was determined by quantitative ^{19}F NMR spectroscopic analysis by comparison of the resonances at δ -177.8 (β-fluoro, minor regioisomer) and δ -193.7 (α-fluoro, major regioisomer). Regioisomer assignment supported by ^{1}H-^{13}C HSQC and HMBC. The crude product was purified using flash column chromatography, with a gradient from 100:0 to 96:4 pentane/Et_2O to obtain the product as a clear oil (15.7 mg, 21% yield).

With KF, the title compound was prepared according to the general procedure **IV (b)** but was carried out at 23 °C. Elevating the temperature was found to favor the α-fluorinated regioisomer in this case. The compound was formed in a 1.2:1 α-fluoro/β-fluoro regioisomeric mixture. The crude product was purified using flash column chromatography, with a gradient from 100:0 to 96:4 pentane/Et_2O to obtain the product as a low-melting white solid (11.4 mg, 15% yield). **^{1}H NMR** (500 MHz, $CDCl_3$) δ 7.49–7.45 (m, 2H), 7.37–7.30 (m, 3H), 5.87–5.77 (m, 2H), 5.66 (dd, *J* = 46.9, 8.4 Hz, 1H), 5.31–5.23 (m, 2H), 5.18–5.13 (m, 2H), 3.90 (dd, *J* = 11.3, 8.5 Hz, 1H), 3.79 (s, 3H), 3.48–3.40 (m, 2H), 3.14 (dd, *J* = 14.5, 7.5 Hz, 2H); **^{13}C NMR** (126 MHz, $CDCl_3$) δ 170.1, 136.0, 132.0 (d, *J* = 2.7 Hz), 129.2, 128.5, 122.1 (d, *J* = 3.7 Hz), 117.9, 89.8 (d, *J* = 10.6 Hz), 84.0 (d, *J* = 23.7 Hz), 81.9 (d, *J* = 171.1 Hz), 64.9 (d, *J* = 28.0 Hz), 54.6, 51.8; **^{19}F NMR** (471 MHz, $CDCl_3$) δ -177.8; **IR** (thin layer film) ν (cm^{-1}) = 2981, 2235, 1736, 1491, 1443, 1359, 1170, 995, 967, 922, 789, 690. **HRMS** (ESI$^+$) *m/z* calculated for $C_{18}H_{21}NO_2F^+$ [M+H]$^+$ 302.1551, found 302.1549.

(±)-methyl 2-(diisobutylamino)-3-fluoro-3-phenylpropanoate (3m)

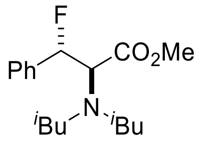

With CsF, the title compound was prepared according to the general procedure **IV (a)**. The compound was formed in a >20:1 regioisomeric mixture, favoring the shown regioisomer. The regioisomeric ratio of the crude product was determined by ^{1}H NMR spectroscopic analysis by comparison of the resonances at δ 5.57 (major regioisomer) and δ 4.22 (minor regioisomer). Regioisomer assignment supported by ^{1}H-^{13}C HSQC and HMBC. The crude product was purified using flash column chromatography, with a gradient from 100:0 to 98:2 pentane/Et_2O to obtain the product as a white solid (41.7 mg, 54% yield).

With KF, the title compound was prepared according to the general procedure **IV (b)**. The compound was formed in a >20:1 regioisomeric mixture, favoring the shown regioisomer. The crude product was purified using flash column chromatography, with a gradient from 100:0 to 98:2 pentane/Et_2O to obtain the product as a white solid (54.3 mg, 70% yield). **^{1}H NMR** (500 MHz, $CDCl_3$) δ 7.42–7.31 (m, 5H), 5.64 (dd, *J* = 45.3, 9.3 Hz, 1H), 3.84 (app t, *J* = 9.4 Hz, 1H), 3.78 (s, 3H), 2.27 (dd, *J* = 12.9, 4.9 Hz, 2H), 2.15 (dd, *J* = 12.9, 9.6 Hz, 2H), 1.60–1.49 (m, 2H), 0.76 (d, *J* = 6.7 Hz, 6H), 0.55 (d, *J* = 6.5 Hz, 6H); **^{13}C NMR** (126 MHz, $CDCl_3$) δ 170.6, 137.5 (d, *J* = 19.0 Hz), 129.2 (d, *J* = 2.3 Hz), 128.3, 127.9 (d, *J* = 5.7 Hz), 92.5 (d, *J* = 170.8 Hz), 67.7 (d, *J* = 30.8 Hz), 60.4, 51.3, 26.5, 20.9, 20.5; **^{19}F NMR** (470 MHz, $CDCl_3$)

δ -173.1; **IR** (thin layer film) ν (cm^{-1}) = 2954, 1727, 1701, 1388, 1346, 1190, 1160, 974, 764, 699; **mp** = 56–58 °C; **HRMS** (ESI^+) *m/z* calculated for $C_{18}H_{29}NO_2F^+$ $[M+H]^+$ 310.2177, found 310.2176.

(±)-methyl (2R,3R)-2-(dibenzylamino)-3-fluoro-3-phenylpropanoate (3n)

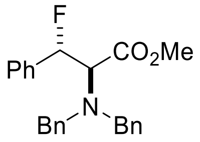

With CsF, the title compound was prepared according to the general procedure **IV (a)** but was allowed to proceed for 96 h. The compound was formed in a 4.8:1 regioisomeric mixture, favoring the shown regioisomer. The regioisomeric ratio of the crude product was determined by ^1H NMR spectroscopic analysis by comparison of the resonances at δ 5.81 (major regioisomer) and δ 5.57 (minor regioisomer). Regioisomer assignment supported by ^1H-^{13}C HSQC and HMBC. The crude product was purified using flash column chromatography, with pure toluene followed by a separate column with a gradient from 95:5 Pentane/Et_2O to obtain the product as a white solid (64.4 mg, 68% yield).

With KF, the title compound was prepared according to the general procedure **IV (b)** but was allowed to proceed for 72 h. The compound was formed in a 12.7:1 regioisomeric mixture, favoring the shown regioisomer. The crude product was purified using flash column chromatography, with pure toluene followed by a separate column with a gradient from 95:5 Pentane/Et_2O to obtain the product as a white solid (57.8 mg, 61% yield). **^{1}H NMR** (400 MHz, $CDCl_3$) δ 7.47–7.39 (m, 1H), 7.39–7.30 (m, 2H), 7.24–7.15 (m, 6H), 7.14–7.06 (m, 2H), 7.01–6.93 (m, 4H), 5.81 (dd, *J* = 45.6, 9.4 Hz, 1H), 3.90 (s, 3H), 3.86 (d, *J* = 13.8 Hz, 2H), 3.81 (dd, *J* = 10.8, 9.4 Hz, 1H), 3.40 (d, *J* = 13.8 Hz, 2H); **^{13}C NMR** (101 MHz, $CDCl_3$) δ 170.6, 138.3, 136.6 (d, *J* = 18.5 Hz), 129.4 (d, *J* = 2.7 Hz), 129.0, 128.4, 128.3, 128.0 (d, *J* = 5.5 Hz), 127.3, 92.8 (d, *J* = 169.3 Hz), 64.6 (d, *J* = 33.2 Hz), 55.3, 51.7; **^{19}F NMR** (471 MHz, $CDCl_3$) δ -174.8; **IR** (thin layer film) ν (cm^{-1}) = 3032, 2360, 2341, 1734, 145, 1171, 983, 697; **mp** = 104–106 °C; **HRMS** (ESI^+) *m/z* calculated for $C_{24}H_{25}NO_2F^+$ $[M+H]^+$ 378.1864, found 378.1863.

(±)-methyl 3-fluoro-2-morpholino-3-phenylpropanoate (3o)

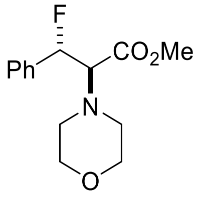

With CsF, the title compound was prepared according to the general procedure **IV (a)** but was allowed to Px1roceed for 72 h. The compound was formed in a 2.2:1 regioisomeric mixture, favoring the shown regioisomer. The regioisomeric ratio of the crude product was determined by ^1H NMR spectroscopic analysis by comparison of the resonances at δ 5.66 (major regioisomer) and δ 5.38 (minor regioisomer). Regioisomer assignment supported by ^1H-^{13}C HSQC and HMBC. The crude product was purified using flash column chromatography, with a gradient from 90:10 to 70:30 pentane/Et_2O, to obtain the product as a white solid (34.5 mg, 52% yield).

With KF, the title compound was prepared according to the general procedure **IV (b)** but was allowed to proceed for 72 h. The compound was formed in a 4.1:1 regioisomeric mixture, favoring the shown regioisomer. The crude product was purified using flash column chromatography, with a gradient from 85:15 to 70:30 pentane/Et_2O, followed by a separate column with a gradient from 99:1 CH_2Cl_2/EtOAc to pure EtOAc to obtain the product as a yellow oil (45.6 mg, 67% yield). **^{1}H NMR** (400 MHz, $CDCl_3$) δ 7.43–7.33 (m, 5H), 5.73 (dd, *J* = 45.1, 8.8 Hz, 1H), 3.79 (s, 3H), 3.57–3.45 (m, 5H), 2.74–2.65 (m, 2H), 2.52–2.42 (m, 2H); **^{13}C NMR** (101 MHz, $CDCl_3$) δ 169.5, 137.3 (d, *J* = 19.4 Hz), 129.0 (d, *J* = 2.2 Hz), 128.4, 126.8 (d, *J* = 6.8 Hz), 91.4 (d, *J* = 172.7 Hz), 71.9 (d, *J* = 30.7 Hz), 67.2, 51.6, 50.9; **^{19}F NMR** (376MHz, $CDCl_3$) δ -180.6; **IR** (thin layer film) ν (cm^{-1}) = 2955, 2858, 2360, 1731, 1153, 1117, 985, 968, 762, 697; **HRMS** (ESI^+) *m/z* calculated for $C_{14}H_{19}NO_3F^+$ $[M+H]^+$ 268.1344, found 268.1344.

(±)-methyl 3-fluoro-3-phenyl-2-thiomorpholinopropanoate (3p)

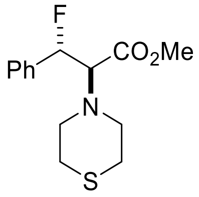

With CsF, the title compound was prepared according to the general procedure **IV (a)** but was allowed to proceed for 72 h. The compound was formed in a 5.3:1 regioisomeric mixture, favoring the shown regioisomer. The regioisomeric ratio of the crude product was determined by ^{1}H NMR spectroscopic analysis by comparison of the resonances at δ 5.72 (major regioisomer) and δ 5.45 (minor regioisomer). Regioisomer assignment supported by ^{1}H-^{13}C HSQC and HMBC. The crude product was purified using flash column chromatography, with a gradient from 97:3 to 90:10 pentane/Et_2O to obtain the product as a white solid (38.9 mg, 55% yield).

With KF, the title compound was prepared according to the general procedure **IV (b)** but was allowed to proceed for 72 h. The compound was formed in a 20:1 regioisomeric mixture, favoring the shown regioisomer. The crude product was purified using flash column chromatography, with a gradient from 95:5 to 90:10 pentane/Et_2O to obtain the product as a yellow oil (53.0 mg, 75% yield). **^{1}H NMR** (500 MHz, $CDCl_3$) δ 7.42–7.31 (m, 5H), 5.71 (dd, *J* = 45.2, 9.0 Hz, 1H), 3.80 (s, 3H), 3.48 (dd, *J* = 10.6, 9.0 Hz, 1H), 3.00–2.92 (m, 2H), 2.72–2.64 (m, 2H), 2.48–2.36 (m, 4H); **^{13}C NMR** (126 MHz, $CDCl_3$) δ 169.7, 137.3 (d, *J* = 19.1 Hz), 129.0 (d, *J* = 2.3 Hz), 128.3, 126.9 (d, *J* = 6.4 Hz), 91.6 (d, *J* = 171.7 Hz), 72.8 (d, *J* = 31.5 Hz), 53.1, 51.7, 28.3; **^{19}F NMR** (376MHz, $CDCl_3$) δ -179.8; **IR** (thin layer film) ν (cm^{-1}) = 2980, 1732, 1454, 1386, 1278, 1167, 1131, 994, 956, 699; **HRMS** (ESI$^+$) *m/z* calculated for $C_{14}H_{19}NO_2SF^+$ [M+H]$^+$ 284.1115, found 284.1115.

(±)-methyl 3-fluoro-3-phenyl-2-(4-phenylpiperazin-1-yl)propanoate (3q)

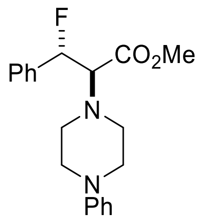

With CsF, the title compound was prepared according to the general procedure **IV (a)** but was allowed to proceed for 72 h. The compound was formed in a 3.5:1 regioisomeric mixture, favoring the shown regioisomer. The regioisomeric ratio of the crude product was determined by ^{1}H NMR spectroscopic analysis by comparison of the resonances at δ 5.69 (major regioisomer) and δ 5.41 (minor regioisomer). Regioisomer assignment supported by ^{1}H-^{13}C HSQC and HMBC. The crude product was purified using flash column chromatography, with a gradient from 98:2 to 85:15 pentane/Et_2O to obtain the product as a white solid (40.4 mg, 47% yield).

With KF, the title compound was prepared according to the general procedure **IV (b)** but was allowed to proceed for 72 h. The compound was formed in a 8.9:1 regioisomeric mixture, favoring the shown regioisomer. The crude product was purified using flash column chromatography, with a gradient from 96:4 to 86:14 pentane/Et_2O to obtain the product as a white solid (60.0 mg, 70% yield). **^{1}H NMR** (500 MHz, $CDCl_3$) δ 7.43−7.32 (m, 5H), 7.28−7.19 (m, 2H), 6.89−6.80 (m, 3H), 5.78 (dd, *J* = 45.1, 9.0 Hz, 1H), 3.81 (s, 3H), 3.62 (dd, *J* = 10.2, 9.2 Hz, 1H), 3.06−2.93 (m, 4H), 2.91−2.83 (m, 2H), 2.66−2.58 (m, 2H); **^{13}C NMR** (126 MHz, $CDCl_3$) δ 169.6, 151.3, 137.4 (d, *J* = 19.2 Hz), 129.2, 129.0 (d, *J* = 1.8 Hz), 128.3, 126.8 (d, *J* = 6.5 Hz), 120.0, 116.3, 91.5 (d, *J* = 172.2 Hz), 71.6 (d, *J* = 30.8 Hz), 51.7, 50.5, 49.6; **^{19}F NMR** (470 MHz, $CDCl_3$) δ -180.7; **IR** (thin layer film) ν (cm^{-1}) = 2958, 2827, 1730, 1599, 1503, 1238, 1155, 1145, 1000, 755; **mp** = 78−80 °C; **HRMS** (ESI$^+$) *m/z* calculated for $C_{20}H_{24}N_2O_2F^+$ [M+H]$^+$ 343.1816, found 343.1815.

(±)-methyl 2-(4-(2-((2,4-dimethylphenyl)thio)phenyl)piperazin-1-yl)-3-fluoro-3-phenylpropanoate (3r)

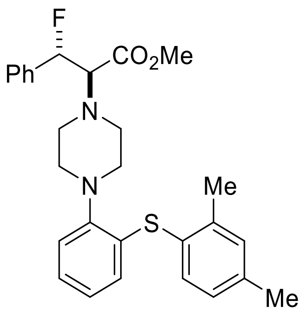

With CsF, the title compound was prepared according to the general procedure **IV (a)** but was performed on 0.15 mmol scale (scaling all components by 60%) and was allowed to proceed for 96 h. The compound was formed in a 3.7:1 regioisomeric mixture, favoring the shown regioisomer. The regioisomeric ratio of the crude product was determined by ^{1}H NMR spectroscopic analysis by comparison of the resonances at δ 5.72 (major regioisomer) and δ 5.46 (minor regioisomer). Regioisomer assignment supported by ^{1}H-^{13}C HSQC and HMBC. The crude product was purified using flash column chromatography, with a gradient from 98:2 to 90:10 pentane/Et_2O to obtain the product as a colorless oil (29.2 mg, 41% yield).

With KF, the title compound was prepared according to the general procedure **IV (b)** but was performed on 0.15 mmol scale (scaling all components by 60%) and was allowed to proceed for 96 h. The compound was formed in a >20:1 regioisomeric mixture, favoring the shown regioisomer. The crude product was purified using flash column chromatography, with a gradient from 100:0 to 90:10 pentane/Et_2O to obtain the product as a colorless oil (60.1 mg, 71% yield). **^{1}H NMR** (500 MHz, $CDCl_3$) δ 7.43−7.37 (m, 5H), 7.35 (d, *J* = 7.8 Hz, 1H), 7.14 (s, 1H), 7.05−6.99 (m, 2H), 6.97−6.93 (m, 1H), 6.86−6.80 (m, 1H), 6.49 (dd, *J* = 7.9, 1.2 Hz, 1H), 5.79 (dd, *J* = 45.2, 9.0 Hz, 1H), 3.82 (s, 3H), 3.62 (dd, *J* = 10.4, 9.1 Hz, 1H), 2.98−2.83 (m, 6H), 2.71−2.63 (m, 2H), 2.36 (s, 3H), 2.30 (s, 3H); **^{13}C NMR** (126 MHz, $CDCl_3$) δ 169.7, 149.2, 142.5, 139.3, 137.6 (d, *J* = 19.1 Hz), 136.2, 134.5, 131.8, 128.9 (d, *J* = 1.7 Hz), 128.3, 128.1, 127.9, 126.9 (d, *J* = 6.5 Hz), 126.4, 125.5, 124.4, 119.8, 91.5 (d, *J* = 172.4), 71.8 (d, *J* = 30.7 Hz), 52.0, 51.7, 50.9, 21.3, 20.7; **^{19}F NMR** (471 MHz, $CDCl_3$) δ -180.8; **HRMS** (ESI^+) *m/z* calculated for $C_{28}H_{32}FN_2O_2S^+$ $[M+H]^+$ 479.2163, found 479.2161.

(±)-methyl 2-(diallylamino)-3-fluoro-3-phenylpropanoate (*cis* diastereomer) (3s)

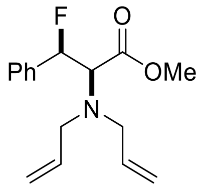

With CsF, the title compound was prepared according to the general procedure **IV (a)**. The compound was formed in a 8.6:1 regioisomeric mixture, favoring the shown regioisomer, with 9.6:1 $dr_{β\text{-}F}$. The regioisomeric ratio of the crude product was determined by quantitative ^{19}F NMR spectroscopic analysis by comparison of the resonances at δ -178.8 (major regioisomer, *trans* diastereomer), δ -185.6 (major regioisomer, *cis* diastereomer) and δ -197.8 (minor regioisomer, *tran*s diastereomer). Regioisomer assignment supported by ^{1}H-^{13}C HSQC and HMBC. As there was poor chromatographic separation of the *cis* and *trans* diastereomers of the β–fluoro regioisomer, only an analytical amount of the compound shown could be isolated and an NMR yield is reported using 1,3,5-trimethoxybenzene as an internal standard. The crude product was purified using flash column chromatography, with a gradient from 100:0 to 96:4 pentane/Et_2O. NMR yield: 48%.

With KF, the title compound was prepared according to the general procedure **IV (b)**. The compound was formed in a 12.6:1 regioisomeric mixture, favoring the shown regioisomer, with 11.6:1 $dr_{β\text{-}F}$. As there was poor chromatographic separation of the *cis* and *trans* diastereomers of the β–fluoro regioisomer, only an analytical amount of the compound shown could be isolated and an NMR yield is reported using 1,3,5-trimethoxybenzene as an internal standard. The crude product was purified using flash column chromatography, with a gradient from 100:0 to 96:4 pentane/Et_2O. NMR yield: 52%. **^{1}H NMR** (500 MHz, $CDCl_3$) δ 7.39−7.28 (m, 5H), 5.88 (dd, *J* = 47.0, 7.1 Hz, 1H), 5.72−5.63 (m, 2H),

5.19−5.12 (m, 2H), 5.11−5.06 (m, 2H), 3.88 (dd, *J* = 18.9, 7.1 Hz, 1H), 3.60 (s, 3H), 3.52−3.45 (m, 2H), 3.25 (dd, *J* = 14.5, 7.4 Hz, 2H); **^{13}C NMR** (126 MHz, $CDCl_3$) δ 170.7, 137.4 (d, *J* = 20.1 Hz), 136.5, 128.7 (d, *J* = 1.8 Hz), 128.3, 126.6 (d, *J* = 7.1 Hz), 117.4, 93.0 (d, *J* = 178.9 Hz), 66.6 (d, *J* = 22.9 Hz), 54.6 (d, *J* = 1.6 Hz), 51.5; **^{19}F NMR** (376 MHz, $CDCl_3$) δ -185.6; **HRMS** (ESI^+) *m/z* calculated for $C_{16}H_{20}NO_2^+$ $[M-F]^+$ 258.1489, found 258.1490.

(±)-N-allyl-N-(2-chloro-2-phenylethyl)prop-2-en-1-amine (3t)

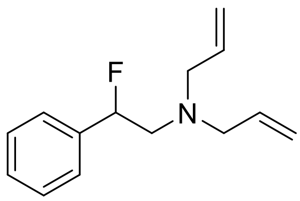

With CsF, the title compound was prepared according to the general procedure **III**. The compound was formed in a 14.7:1 regioisomeric mixture, favoring the shown regioisomer. The regioisomeric ratio of the crude product was determined by quantitative ^{19}F NMR spectroscopic analysis by comparison of the resonances at δ -179.4 (major regioisomer) and δ -219.8 (minor regioisomer). Regioisomer assignment supported by ^{1}H-^{13}C HSQC and HMBC. The crude product was purified using flash column chromatography, with a gradient from 100:0 to 94:6 pentane/Et_2O to obtain the product as a colorless oil (49.4 mg, 90% yield).

With CsF, the title compound was prepared according to the general procedure **IV (a)**. The compound was formed in a >20:1 regioisomeric mixture, favoring the shown regioisomer. The crude product was purified using flash column chromatography, with a gradient from 100:0 to 94:6 pentane/Et_2O to obtain the product as a colorless oil (46.0 mg, 84% yield).

With KF, the title compound was prepared according to the general procedure **IV (b)**. The compound was formed as a single regioisomer, favoring the shown regioisomer. The crude product was purified using flash column chromatography, with a gradient from 95:5 to 90:10 pentane/Et_2O to obtain the product as a colorless oil (33.9 mg, 62% yield). **^{1}H NMR** (400 MHz, $CDCl_3$) δ 7.41–7.28 (m, 5H), 5.94–5.79 (m, 2H), 5.60 (ddd, *J* = 48.6, 8.5, 3.0 Hz, 1H), 5.24–5.11 (m, 4H), 3.25 (d, *J* = 6.5 Hz, 4H), 2.99 (ddd, *J* = 16.8, 14.6, 8.5 Hz, 1H), 2.77 (ddd, *J* = 32.8, 14.6, 3.1 Hz, 1H); **^{13}C NMR** (126 MHz, $CDCl_3$) δ 139.2 (d, *J* = 19.9 Hz), 135.7, 128.5, 128.4 (d, *J* = 1.8 Hz), 125.6 (d, *J* = 7.2 Hz), 117.9, 93.4 (d, *J* = 173.5 Hz), 59.2 (d, *J* = 23.6 Hz), 57.8; **^{19}F NMR** (471 MHz, $CDCl_3$) δ -179.4; **IR** (thin layer film) ν (cm^{-1}) = 3076, 1643, 1453, 1261, 1206, 996, 918, 755, 698; **HRMS** (ESI^+) *m/z* calculated for $C_{14}H_{19}NF^+$ $[M+H]^+$ 220.1496, found 220.1497.

(±)-methyl 3-(dibenzylamino)-2-fluoropropanoate (2u)

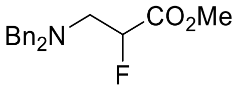

With CsF, the title compound was prepared according to the general procedure **III**. The compound was formed in a >20:1 regioisomeric mixture, favoring the shown regioisomer. The regioisomeric ratio of the crude product was determined by quantitative ^{19}F NMR spectroscopic analysis by comparison of the resonances at δ -190.4 (major regioisomer) and δ -225.1 (minor regioisomer). The crude product was purified using flash column chromatography, with a gradient from 100:0 to 98:2 pentane/Et_2O to obtain the product as a colorless oil (54.2 mg, 72% yield).

With CsF, the title compound was prepared according to the general procedure **IV (a)**. The compound was formed in a 11.6:1 regioisomeric mixture, favoring the shown regioisomer. The crude product was purified using flash column chromatography, with a gradient from 100:0 to 98:2 pentane/Et_2O to obtain the product as a colorless oil (55.0 mg, 73% yield).

With KF, the title compound was prepared according to the general procedure **IV (b)**. The compound was formed in a >20:1 regioisomeric mixture, favoring the shown regioisomer. The crude product was

purified using flash column chromatography, with a gradient from 100:0 to 96:4 pentane/Et_2O to obtain the product as a colorless oil (65.6 mg, 87% yield). This compound has been previously synthesized and closely matched literature values.[14] **^{1}H NMR** (500 MHz, $CDCl_3$) δ 7.38−7.31 (m, 8H), 7.30−7.25 (m, 2H), 5.08 (ddd, *J* = 49.4, 6.0, 3.0 Hz, 1H), 3.86 (d, *J* = 13.7 Hz, 2H), 3.72 (s, 3H), 3.56 (d, *J* = 13.6 Hz, 2H), 3.13−2.95 (m, 2H); **^{13}C NMR** (101 MHz, $CDCl_3$) δ 169.3 (d, *J* = 24.4 Hz), 138.9, 129.1, 128.4, 127.3, 89.5 (d, *J* = 187.0 Hz), 58.9 (d, *J* = 1.3 Hz), 54.4 (d, *J* = 20.2 Hz), 52.3; **^{19}F NMR** (376 MHz, $CDCl_3$) δ -190.5.

(±)-methyl 3-cyclohexyl-3-(dibenzylamino)-2-fluoropropanoate (2v)

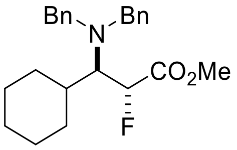

With CsF, the title compound was prepared according to the general procedure **III**. The compound was formed in a >20:1 regioisomeric mixture, favoring the shown regioisomer. The regioisomeric ratio of the crude product was determined by quantitative ^{19}F NMR spectroscopic analysis by comparison of the resonances at δ -201.0 (minor regioisomer) and δ -202.5 (major regioisomer). Regioisomer assignment supported by ^{1}H-^{13}C HSQC and HMBC. The crude product was purified using flash column chromatography, with a gradient from 100:0 to 94:6 pentane/Et_2O to obtain the product as a white solid (69.0 mg, 72% yield).

With CsF, the title compound was prepared according to the general procedure **IV (a)**. The compound was formed in a 18.0:1 regioisomeric mixture, favoring the shown regioisomer. The crude product was purified using flash column chromatography, with a gradient from 100:0 to 94:6 pentane/Et_2O to obtain the product as a white soild (73.9 mg, 77% yield).

With KF, the title compound was prepared according to the general procedure **IV (b)**. The compound was formed in a 15.2:1 regioisomeric mixture, favoring the shown regioisomer. The crude product was purified using flash column chromatography, with a gradient from 100:0 to 94:6 pentane/Et_2O to obtain the product as a white solid (79.8 mg, 83% yield). **^{1}H NMR** (400 MHz, $CDCl_3$) δ 7.31−7.20 (m, 8H), 7.19−7.13 (m, 2H), 5.29 (d, *J* = 48.2 Hz, 1H), 3.88 (d, *J* = 13.8 Hz, 2H), 3.67 (s, 3H), 3.25 (dd, *J* = 13.8, 1.3 Hz, 2H), 2.91 (dd, *J* = 29.0, 9.8 Hz, 1H), 2.20 (d, *J* = 13.2 Hz, 1H), 1.88−1.75 (m, 1H), 1.64−1.55 (m, 2H), 1.55−1.46 (m, 2H), 1.21−1.03 (m, 2H), 1.02−0.88 (m, 1H), 0.71−0.54 (m, 2H); **^{13}C NMR** (101 MHz, $CDCl_3$) δ 171.4 (d, *J* = 23.1 Hz), 139.4, 129.3, 128.3, 127.2, 86.3 (d, *J* = 196.0 Hz), 64.8 (d, *J* = 18.9 Hz), 54.7, 52.4, 35.7 (d, *J* = 4.1 Hz), 30.9, 29.5, 26.5, 26.3 (d, *J* = 3.7 Hz); **^{19}F NMR** (376 MHz, $CDCl_3$) δ -202.5; **IR** (thin layer film) ν (cm^{-1}) = 2949, 2849, 1762, 1214, 1108, 1076, 754, 742, 698; **mp** = 129−131 °C; **HRMS** (ESI$^+$) *m/z* calculated for $C_{24}H_{31}NO_2F^+$ $[M+H]^+$ 384.2333, found 384.2336.

Synthesis of α-Fluorinated Amino Acid HCl Salt

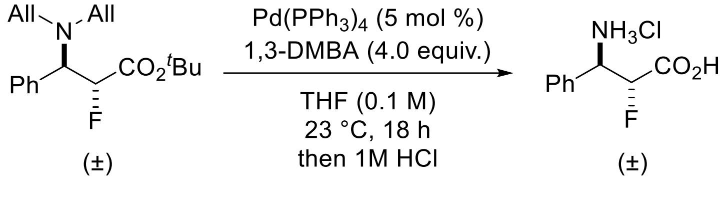

A 100 mL round-bottomed flask with a stir bar was sequentially charged with β-fluorinated amino ester, degassed THF (15.7 mL, 0.1 M), $Pd(PPh_3)_4$ (90.7 mg, 78.5 μmol, 5 mol %), and 1,3-dimethylbarbituric acid (1,3-DMBA, 980.6 mg, 6.3 mmol, 4.0 equiv.). The reaction was stirred at room

temperature under an N_2 atmosphere for 18 h, then concentrated *in vacuo*. The residue was partitioned between diethyl ether and 1M HCl. The layers were separated and the organic layer was extracted with 1M HCl once more. The combined aqueous layers were washed with diethyl ether four times before concentrating *in vacuo*. The solid was collected from the flask by transferring it to a fritted funnel with diethyl ether, and then dried under vacuum to obtain the product.

(±)-3-amino-2-fluoro-3-phenylpropanoic acid hydrochloride (2l)

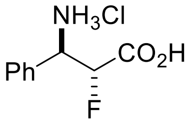

Using the procedure above, the product was obtained as a white solid (282.0 mg, 82% yield). **^{1}H NMR** (500 MHz, D_2O) δ 7.56–7.43 (m, 5H), 5.57 (dd, *J* = 50.1, 3.0 Hz, 1H), 4.99 (dd, *J* = 29.0, 3.0 Hz, 1H), 3.23 (s, 2H).); **^{13}C NMR** (126 MHz, D_2O) δ 170.01 (d, *J* = 21.8 Hz), 130.45, 130.15, 129.22, 128.16 (d, *J* = 2.2 Hz), 88.56 (d, *J* = 190.4 Hz), 55.49 (d, *J* = 18.5 Hz); **^{19}F NMR** (471 MHz, D_2O) δ -199.1; **IR** (thin layer film) ν (cm^{-1}) = 2885, 1751, 1593, 1506, 1222, 1119, 1013, 821, 732, 697; **mp** = 172–173 °C; **HRMS** (ESI^+) *m/z* calculated for $C_9H_{11}FNO_2^+$ [free amine + H]$^+$ 184.0768, found 184.0762.

Synthesis of β-Fluorinated Amino Acid HCl Salt

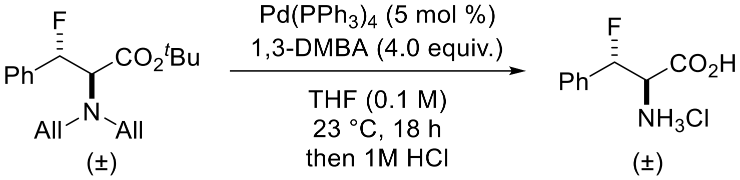

A 100 mL round-bottomed flask with a stir bar was sequentially charged with β-fluorinated amino ester, degassed THF (15.7 mL, 0.1 M), $Pd(PPh_3)_4$ (90.7 mg, 78.5 µmol, 5 mol %), and 1,3-dimethylbarbituric acid (1,3-DMBA, 980.6 mg, 6.3 mmol, 4.0 equiv.). The reaction was stirred at room temperature under an N_2 atmosphere for 18 h, then concentrated *in vacuo*. The residue was partitioned between diethyl ether and 1M HCl. The layers were separated and the organic layer was extracted with 1M HCl once more. The combined aqueous layers were washed with diethyl ether four times before concentrating *in vacuo*. The solid was collected from the flask by transferring it to a fritted funnel with diethyl ether, and then dried under vacuum to obtain the product.

(±)-2-amino-3-fluoro-3-phenylpropanoic acid hydrochloride (3l)

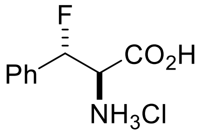

Using the procedure above, the product was obtained as a white solid (295.7 mg, 86% yield). **^{1}H NMR** (500 MHz, CD_3OD) δ 7.51-7.36 (m, 5H), 6.18 (dd, *J* = 44.0, 3.2 Hz, 1H), 4.70 (dd, *J* = 17.5, 3.4 Hz, 1H); **^{13}C NMR** (126 MHz, CD_3OD) δ 167.5 (d, *J* = 9.1 Hz), 134.7 (d, *J* = 20.9 Hz), 130.6, 129.9, 126.6 (d, *J* = 8.4 Hz), 92.0 (d, *J* = 180.6 Hz), 58.5 (d, *J* = 24.7 Hz); **^{19}F NMR** (471 MHz, CD_3OD) δ -191.1; **IR** (thin layer film) ν (cm^{-1}) = 2892, 2630, 2341, 1742, 1593, 1498, 1224, 1016, 742, 698; **mp** = 194–196 °C; **HRMS** (ESI^+) *m/z* calculated for $C_9H_{11}FNO_2^+$ [free amine + H]$^+$ 184.0768, found 184.0769.

Enantiospecific Synthesis of α- and β–Fluoro Esters

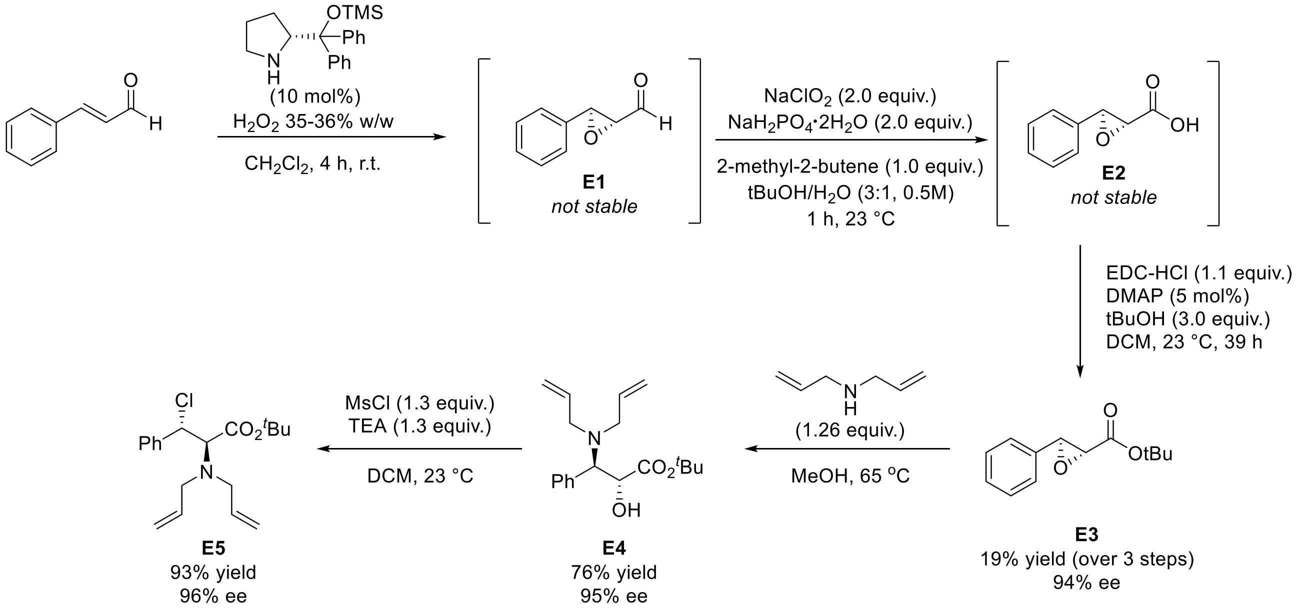

(2*R*,3*S*)-3-phenyloxirane-2-carbaldehyde (E1)

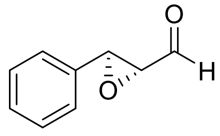

A 500 mL round-bottom flask was sequentially charged with (*R*)-(+)-α,α-diphenyl-2-pyrrolidinemethanol trimethylsilyl ether (1.0 g, 3.1 mmole, 10 mol %; CAS: 943757-71-9), CH_2Cl_2 (31 mL, 1.0 M) and *trans*-cinnamaldehyde (3.9 mL, 31.3 mmol, 1 equiv.). Then at 0 °C, H_2O_2 35 % w/w (4.68 mL, 54.4 mmol, 1.7 equiv.) was added dropwise. The reaction was stirred at room temperature for 4 h, then quenched with saturated aqueous $Na_2S_2O_3$ and extracted three times with dichloromethane. The combined organic layers were washed once with brine, dried over Na_2SO_4 and concentrated *in vacuo* to obtain the crude epoxy aldehyde **E1**, which was used directly in the next step.

(2*R*,3*S*)-3-phenyloxirane-2-carboxylic acid (E2)

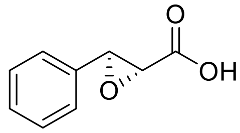

The crude aldehyde **E1**, was dissolved in 3:1 mixture of ^{t}BuOH:H_2O (62 mL, 0.5 M) and $Na_2H_2PO_4 \cdot 2H_2O$ (9.8g, 65.6 mmol, 2 equiv.) was added. After dissolution, the reaction was cooled to 0 °C and 2-methyl-2-butene (3.3 mL, 31.3 mmol, 1 equiv.) and $NaClO_2$ (7.1 g, 62.6 mmol, 2 equiv.) were added sequentially. The reaction was allowed to warm to room temperature and was stirred for 1 h. After this period, the mixture was cooled to 0 °C, quenched with saturated aqueous $NaHCO_3$, diluted with ethyl acetate and then stirred for 10 min. Additional water was added and the mixture was washed twice with ethyl acetate. The aqueous phase was adjusted to pH 3-4 with 2M HCl and extracted with dichloromethane four times. The combined organic layers were dried over $MgSO_4$ and concentrated *in vacuo* to obtain the crude epoxy carboxylic acid **E2**, which was used directly in the next step.

tert-butyl (2_R_,3_S_)-3-phenyloxirane-2-carboxylate (E3)

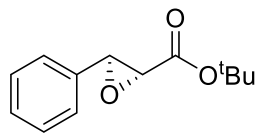

The crude carboxylic acid (**E2**) was dissolved in anhydrous dichloromethane (31 mL, 1.0 M), and treated with 4-dimethylaminopyridine (191 mg, 1.6 mmol, 5 mol %) and ^{t}BuOH (8.9 mL, 94.0 mmol, 3 equiv.). The reaction was cooled to 0 °C and *N*-(3-dimethylaminopropyl)-*N'*-ethylcarbodiimide hydrochloride (6.6 g, 34.5 mmol, 1.1 equiv.) was added in small portions. The reaction was allowed to warm to room temperature and was stirred for 39 h. The mixture was washed twice with 0.1 M HCl, saturated aqueous $NaHCO_3$, brine, and then dried over Na_2SO_4. The solvent was evaporated *in vacuo* and the residue was purified by flash chromatography with a gradient from 98:2 to 92:8 pentane/Et_2O to obtain the product **E3** as pale yellow solid (1.3 g, 19% yield over three steps). **$[\alpha]_D^{25\,°C}$ =** -154.4° (*c* = 0.004 g/mL, MeOH, 94% ee); **HPLC**: DAICEL CHIRALPAK® IB-3, Heptane:^{i}PrOH = 99:1, 1 mL/min; t_1 = 4.28 min (minor), t_2 = 4.89 min (major).

tert-butyl-(2_R_,3_R_)-3-(diallylamino)-2-hydroxy-3-phenylpropanoate (E4)

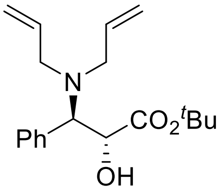

Prepared according to the general procedure for opening of epoxides with secondary amines; the reaction was allowed to proceed for 29 h, and then a further portion of diallylamine (135 µL, 1.1 mmol, 0.21 equiv.) was added and the reaction was allowed to proceed for a further 10 h. The reaction was performed on 5.2 mmol scale. All spectra were in agreement with the racemic compound. The product was isolated as colorless oil (1.25 g, 3.9 mmol, 76%). **$[\alpha]_D^{25\,°C}$ =** -53.2° (*c* = 0.003 g/mL, MeOH, 95% ee); **HPLC**: DAICEL CHIRALPAK® IB-3, Heptane:^{i}PrOH = 99:1, 1 mL/min; t_1 = 5.56 min (minor), t_2 = 7.15 min (major).

tert-butyl (2_R_,3_S_)-3-chloro-2-(diallylamino)-3-phenylpropanoate (E5)

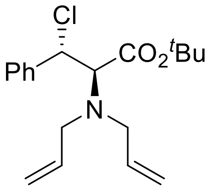

Prepared according to the general procedure for chlorination of amino alcohols, but using 1.3 equiv. MsCl and 1.3 equiv. triethylamine. The reaction was performed on 3.7 mmol scale. All spectra were in agreement with the racemic compound. The product was isolated as colorless oil (1.17 g, 3.5 mmol, 93%). **$[\alpha]_D^{25\,°C}$** = +60.9° (*c* = 0.005 g/mL, MeOH, 96% ee); **HPLC**: DAICEL CHIRALPAK® IC-3, Heptane:^{i}PrOH = 99.5:0.5, 1 mL/min; t_1 = 4.53 min (minor), t_2 = 5.80 min (major).

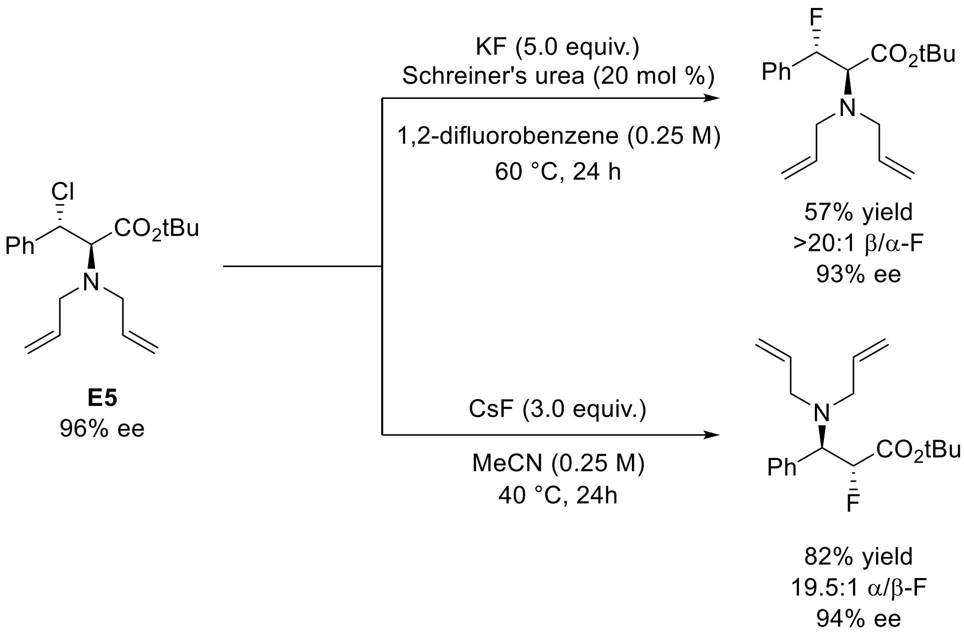

***tert*-butyl (2*R*,3*S*)-2-(diallylamino)-3-fluoro-3-phenylpropanoate**

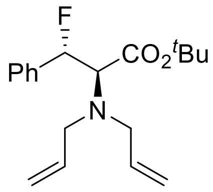

Prepared according to the general procedure for β-fluorination of amino chlorides; the reaction was carried out on 0.25 mmol scale. NMR data matched the racemic compound. The product was isolated as a colorless oil (45 mg, 0.14 mmol, 56%). **[α]$_D$$^{25\,°C}$ =** -10.3° (*c* = 0.003 g/mL, MeOH, 93% ee); **HPLC**: DAICEL CHIRALPAK® IC-3, Heptane:EtOH= 99.5:0.5, 1 mL/min; t_1 = 3.52 min (minor), t_2 = 4.63 min (major).

***tert*-butyl (2*R*,3*R*)-3-(diallylamino)-2-fluoro-3-phenylpropanoate**

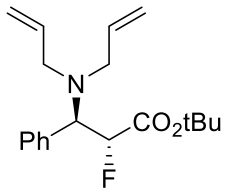

Prepared according to the general procedure for α-fluorination of amino chlorides; the reaction was carried out on 0.25 mmol scale. NMR data matched the racemic compound. The product was isolated as a colorless oil (64 mg, 0.20 mmol, 80%). **[α]$_D$$^{25\,°C}$ =** -43.4° (*c* = 0.021 g/mL, MeOH, 94% ee); **HPLC**: DAICEL CHIRALPAK® IB-3, Heptane:^{i}PrOH = 99:1, 1 mL/min; t_1 = 2.74 min (minor), t_2 = 2.97 min (major).

(1)

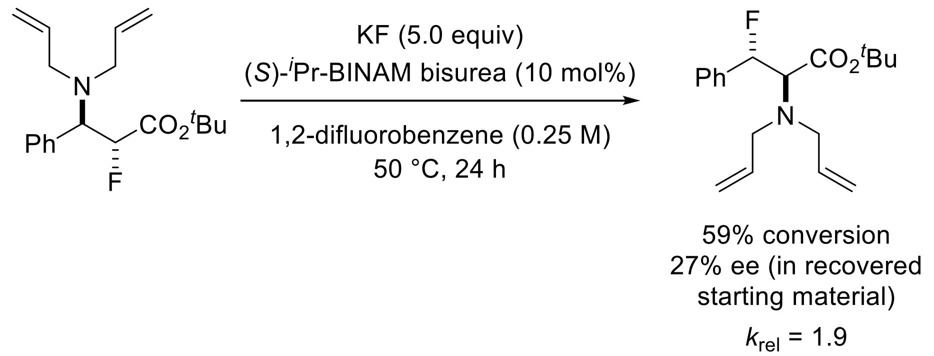

(2)

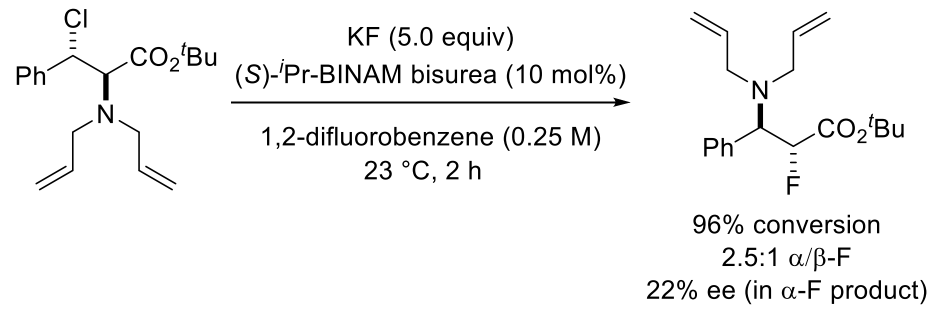

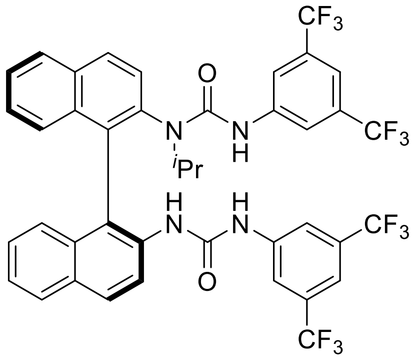

(*S*)-^{i}Pr-BINAM bisurea
CAS: 2227157-06-2

Synthesis of Enantioenriched Epoxy Amides via Shibasaki Epoxidation

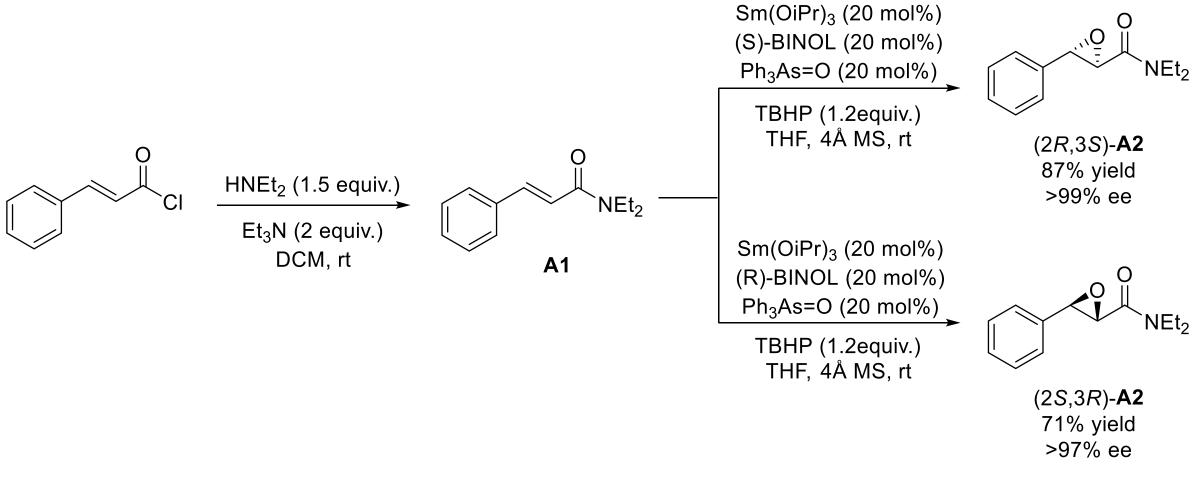

Enamide **A1** was synthesized through a known protocol from cinnamoyl chloride.[15] Subsequently, Shibasaki's asymmetric epoxidation was utilized to access both enantiomers of **A2** in highly enantioenriched form.[16]

Catalytic Asymmetric Epoxidation of β-Aryl Substituted α,β-Unsaturated Amides

To a stirred mixture of MS 4A [1 g/mmol of starting material; MS 4A was dried for 3 h at 180 °C under reduced pressure] was added (*S*)- or (*R*)-BINOL (4.9 mmol, 20 mol %) and triphenylarsine oxide (4.9 mmol, 20 mol %) as a THF solution (0.01 M), followed by $Sm(O^iPr)_3$ (0.1 M solution in THF, 4.9 mmol, 20 mol %) at room temperature. After being stirred for 45 min at the same temperature, *tert*-butyl hydroperoxide (59.0 mmol, 5.0 M solution in decane, 2.4 equiv.) was added. After being stirred for 10 min, the enamide substrate (49.0 mmol) was added directly and the mixture was stirred at room temperature. After 24 h, ^1H NMR indicated quantitative conversion and the reaction mixture was diluted with ethyl acetate then quenched with 2% citric acid (300 ml). The aqueous layer was extracted with EtOAc three times and the combined organic layers were washed with brine and dried over $MgSO_4$. After concentration *in vacuo*, the residue was purified by flash chromatography using a gradient from 80:20 to 70:30 pentane/EtOAc to obtain the enantioenriched epoxy amide.

(2*R*,3*S*)-*N*,*N*-diethyl-3-phenyloxirane-2-carboxamide

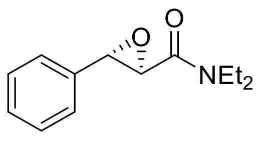

Prepared according to the general procedure using (*S*)-BINOL; the reaction was carried out on 24.6 mmol scale. NMR data was in agreement with the racemic compound. The product was isolated as a pale yellow solid (4.68 g, 87% yield). **$[\alpha]_D^{25\,°C}$ =** -131.0° (*c* = 0.010 g/mL, DCM). **HPLC**: DAICEL CHIRALPAK® IF-3, Heptane:IPA 90:10, flow rate 1 mL/min, t_1 = 16.3 min (major isomer) and t_2 = 19.3 min (minor isomer); >99% ee.

(2*S*,3*R*)-*N*,*N*-diethyl-3-phenyloxirane-2-carboxamide

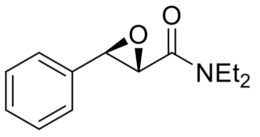

Prepared according to the general procedure using (*R*)-BINOL; the reaction was carried out on 15.7 mmol scale. NMR data was in agreement with the racemic compound.The product was isolated as a pale yellow solid (2.44 g, 71% yield). **$[\alpha]_D^{25\,°C}$** = +132.0° (*c* = 0.010 g/mL, DCM). **HPLC**: DAICEL CHIRALPAK® IF-3, Heptane:IPA 90:10, flow rate 1 mL/min, t_1 = 16.3 min (minor isomer) and t_2 = 19.3 min (major isomer); >99% ee.

Compounds Derived from Enantioenriched Epoxy Amides

(2*R*,3*R*)-3-(diallylamino)-*N*,*N*-diethyl-2-hydroxy-3-phenylpropanamide

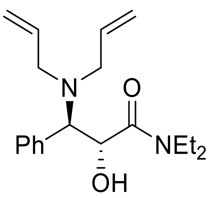

The title compound was prepared according to the general procedure for the opening of epoxides with secondary amines using 1.15 equiv. of diallylamine; the reaction was performed on 13.7 mmol scale and was allowed to proceed for 24 h. The crude mixture was purified by silica gel chromatography using a gradient mobile phase of 10-30% EtOAc/pentane. No minor regioisomer was present in the isolated product. NMR data was in agreement with the racemic compound. The product was isolated as a white solid (3.35 g, 77% yield). **$[\alpha]_D^{25\,°C}$ =** -35.0° (*c* = 0.0033 g/mL, DCM).

HPLC: DAICEL CHIRALPAK® IF-3, Heptane:IPA 97:3, flow rate 1 mL/min, t_1 = 23.4 min (major isomer) and t_2 = 25.3 min (minor isomer); >99% ee.

(2*S*,3*S*)-3-(diallylamino)-*N*,*N*-diethyl-2-hydroxy-3-phenylpropanamide

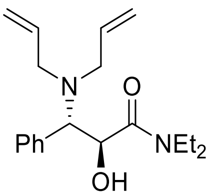

The title compound was prepared according to the general procedure for the opening of epoxides with secondary amines using 1.05 equiv. of diallylamine; the reaction was performed on 9.1 mmol scale and was allowed to proceed for 24 h. The crude mixture was purified by silica gel chromatography using a gradient mobile phase of 10-30% EtOAc/pentane. No minor regioisomer was present in the isolated product. NMR data was in agreement with the racemic compound. The product was isolated as a white solid (1.84 g, 64% yield). **$[\alpha]_D^{25\,°C}$ =** +32.0° (*c* = 0.0043 g/mL, DCM). **HPLC**: DAICEL CHIRALPAK® IF-3, Heptane:IPA 97:3, flow rate 1 mL/min, t_1 = 23.6 min (minor isomer) and t_2 = 24.6 min (major isomer); 98% ee.

(2*R*,3*S*)-3-chloro-2-(diallylamino)-*N*,*N*-diethyl-3-phenylpropanamide

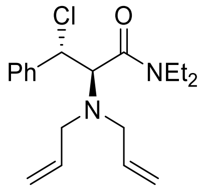

The title compound was prepared according to the general procedure for chlorination of amino alcohols; the reaction was performed on 9.5 mmol scale and was allowed to proceed for 3.5 h; 1.3 equiv. triethylamine and methanesulfonyl chloride were used. The compound was isolated as a 12.2:1 regioisomeric mixture, favoring the shown regioisomer. The regioisomeric ratio of the isolated product was determined by ^{1}H NMR spectroscopic analysis by comparison of the resonances at δ 4.71 (minor regioisomer) and δ 4.16 (major regioisomer). NMR data was in agreement with the racemic compound. **$[\alpha]_D^{25\,°C}$ =** +30.3° (*c* = 0.0064 g/mL, DCM). **HPLC**: DAICEL CHIRALPAK® OJ-3, Heptane:IPA 99:1, flow rate 1 mL/min, t_1 = 4.9 min (major isomer) and t_2 = 6.3 min (minor isomer); >99% ee.

(2*S*,3*R*)-3-chloro-2-(diallylamino)-*N*,*N*-diethyl-3-phenylpropanamide

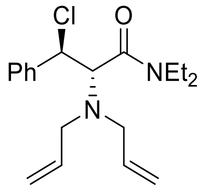

The title compound was prepared according to the general procedure for chlorination of amino alcohols; the reaction was performed on 5.2 mmol scale and was allowed to proceed for 4 h; 1.3 equiv. triethylamine and methanesulfonyl chloride were used. The compound was isolated as a 15.7:1 regioisomeric mixture, favoring the shown regioisomer. The regioisomeric ratio of the isolated product was determined by ^{1}H NMR spectroscopic analysis by comparison of the resonances at δ 4.71 (minor regioisomer) and δ 4.16 (major regioisomer). NMR data was in agreement with the racemic compound. **$[\alpha]_D^{25\,°C}$ =** -31.0° (*c* = 0.0060 g/mL, DCM). **HPLC**: DAICEL CHIRALPAK® OJ-3, Heptane:IPA 99:1, flow rate 1 mL/min, t_1 = 5.0 min (minor isomer) and t_2 = 6.3 min (major isomer); >99% ee.

Asymmetric α-Fluorination of Racemic Amino Chloride Substrates

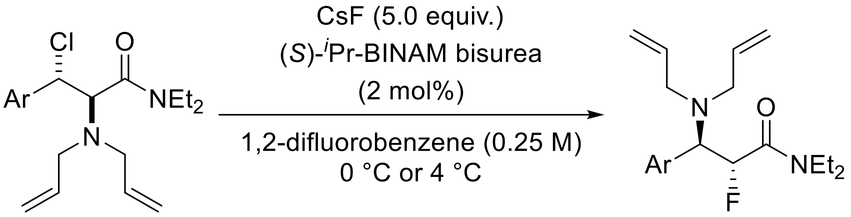

A 7 mL vial equipped with a stir bar was sequentially charged with the amino chloride substrate (1.1 mmol, 1.0 equiv.), (*S*)-^{i}Pr-BINAM bisurea (18.2 mg, 0.02 mmol, 2 mol %), and CsF (827 mg, 3.9 mmol, 5.0 equiv.) before adding 1,2-difluorobenzene (4.4 mL, 0.25M). The reaction was placed in the center of a stir plate at a height of 2 cm and was stirred at 1200 rpm at 4 °C or 0 °C. Once the desired level of conversion was reached (50-60% was generally optimal), Et_2O was used to flush the reaction through a short plug of silica gel and the filtrate was concentrated *in vacuo*. The crude material thusly obtained was purified using flash column chromatography to obtain ted product. To obtain larger amounts of product, the reaction as carried out on gram scale, as in the example below. The crude material thusly obtained was purified using flash column chromatography to obtain pure α-fluorinated product.

Catalyst removal procedure (optional): After flushing the reaction through a silica plug, the crude reaction mixture was concentrated as above. The crude residue was dissolved in a small amount of DCM (~2 mL) and tetrabutylammonium acetate (0.2 equiv.) was added. After stirring for 15 minutes at room temperature, the crude reaction mixture was concentrated once again and purified by flash column chromatography.

(2*R*,3*R*)-2-(diallylamino)-*N*,*N*-diethyl-3-fluoro-3-phenylpropanamide

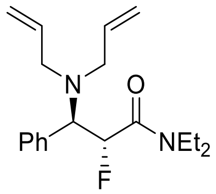

The title compound was prepared according to the general procedure for asymmetric α-fluorination of racemic amino-chloride amide substrates on 1 g scale (3.0 mmol). A 50 mL round bottom flask was charged with amino chloride substrate (1 g, 3.0 mmol), (*S*)-^{i}Pr-BINAM bisurea catalyst (0.06 mmol, 50.2 mg), and CsF (2.28 g, 15.0 mmol). The flask, as well as a container of the solvent (1,2-difluorobenzene), was placed in a cold room at 4 °C for 30 min. Then, 1,2-difluorobenzene (12 mL, 0.25 M) was added at the same temperature. The reaction was allowed to stir for 12 h at 1200 rpm. Et_2O was used to flush the reaction through a short plug of silica gel and the filtrate was concentrated *in vacuo*. The crude material was then analyzed by quantitative ^{1}H NMR and ^{19}F NMR to record its conversion (60%), and regiomeric ratio (3.2:1 α-/β-F). The α-fluoride was then isolated using flash column chromatography, with a gradient from 90:10 to 84:16 pentane/Et_2O to obtain the product as a colorless viscose oil (398.0 mg, 42% yield). NMR data was in agreement with the racemic compound. **$[\alpha]_D^{25\,°C}$** = -66.0° (*c* = 0.0080 g/mL, DCM). **HPLC**: DAICEL CHIRALPAK® IA-3, Heptane:IPA 99:1, flow rate 1 mL/min, t_1 = 9.2 min (major isomer) and t_2 = 12.4 min (minor isomer); 81.3% ee.

(*2R*,*3R*)-3-(diallylamino)-*N*,*N*-diethyl-2-fluoro-3-(naphthalen-2-yl)propanamide

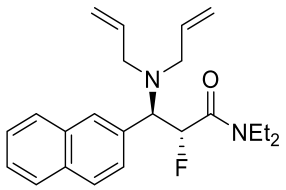

The title compound was prepared according to the general procedure for asymmetric α-fluorination of racemic amino-chloride amide substrates, but on 0.5 mmol scale. A 7 mL vial was charged with amino chloride substrate (192 mg, 0.5 mmol), (*S*)-^{i}Pr-BINAM bisurea catalyst (10 µmol, 8.4 mg), and CsF (380 mg, 2.5 mmol). The vial, as well as a container of the solvent (1,2-difluorobenzene), was placed in a ice bath at 0 °C for 30 min. Then, 1,2-difluorobenzene (2 mL, 0.25 M) was added at the same temperature. The reaction was allowed to stir for 16 h at 1200 rpm. Et_2O was used to flush the reaction through a short plug of silica gel and the filtrate was concentrated *in vacuo*. The crude material was then analyzed by quantitative ^{1}H NMR and ^{19}F NMR to record its conversion (62%), and regiomeric ratio (1.9:1 α-/β-F). The α-fluoride was then isolated using flash column chromatography, with a gradient from 90:10 to 88:12 pentane/Et_2O to obtain the product as a colorless oil (72.0 mg, 39% yield). **^{1}H NMR** (500 MHz, $CDCl_3$ δ 7.87 – 7.80 (m, 3H), 7.76 (d, *J* = 1.8 Hz, 1H), 7.54 (d, *J* = 8.5 Hz, 1H), 7.51 – 7.45 (m, 2H), 5.84 (m, 2H), 5.69 (dd, *J* = 49.6, 6.7 Hz, 1H), 5.23 – 5.13 (m, 4H), 4.64 (dd, *J* = 18.3, 6.6 Hz, 1H), 3.43 – 3.36 (m, 3H), 3.32 – 3.17 (m, 2H), 3.07 – 2.85 (m, 3H), 1.12 (t, *J* = 7.1 Hz, 3H), 1.07 (t, *J* = 7.1 Hz, 3H); **^{13}C NMR** (126 MHz, $CDCl_3$) δ 167.1 (d, *J* = 19.5 Hz), 136.2, 133.2, 133.1, 133.0, 129.0, 128.1, 127.8, 127.7, 127.6, 126.2, 126.1, 117.6, 89.2 (d, *J* = 189.8 Hz), 64.8 (d, *J* = 21.8 Hz), 53.9, 41.3 (d, *J* = 6.8 Hz), 41.2, 14.8, 12.7; **^{19}F NMR** (376 MHz, $CDCl_3$) δ -190.4; **IR** (thin layer film) ν (cm^{-1}) = 3004, 2970, 2159, 1739, 1645, 1300, 1216, 1169, 872, 720; **HRMS** (ESI^{+}) *m/z* calculated for $C_{23}H_{30}FN_2O^{+}$ $[M+H]^{+}$ 369.2337, found 369.2334; **$[\alpha]_D^{25\,°C}$ =** -81.2° (*c* = 0.0091 g/mL, DCM). **HPLC**: DAICEL CHIRALPAK® ID-3, Heptane:IPA 98:2, flow rate 1 mL/min, t_1 = 14.6 min (major isomer) and t_2 = 20.4 min (minor isomer); 82.4% ee.

Thermodynamic equilibration of α-fluoride to form β-fluoride

(*2R*,*3S*)-2-(diallylamino)-*N*,*N*-diethyl-3-fluoro-3-phenylpropanamide

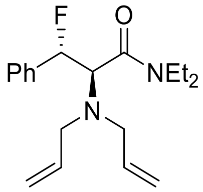

A 25 mL round bottom flask equipped with a stir bar was sequentially charged with the amino chloride substrate (0.94 mmol, 1.0 equiv.), Schreiner's urea (0.09 mmol, 10 mol %), and KF (4.71 mmol, 5.0 equiv.) before adding 1,2-difluorobenzene (3.8 mL, 0.25 M). The reaction was placed in the center of a stir plate and was stirred at 1200 rpm at 60 °C (in an oil bath) for 96 h. Et_2O was used to flush the reaction through a short plug of silica gel and the filtrate was concentrated *in vacuo*. The crude material was then analyzed by quantitative ^{1}H NMR and ^{19}F NMR to record the conversion (>95%) and regiomeric ratio (6.8:1 β-/α-F; favoring the shown regioisomer). The β-fluoride was then isolated using flash column chromatography, with a gradient from 95:10 to 90:10 pentane/Et_2O to obtain the product as a white solid (205.0 mg, 68% yield). All spectra were in agreement with the racemic compound. The isolated β-fluoride product was recrystallized from pure hexane: 148 mg, 49% yield, 97.2% ee. **$[\alpha]_D^{25\,°C}$ =** +7.1° (*c* = 0.0050 g/mL, DCM). **HPLC**: DAICEL CHIRALPAK® OJ-3, Heptane:IPA 99:1, flow rate 1 mL/min, t_1 = 4.7 min (major isomer) and t_2 = 5.9 min (minor isomer), detection at 220 nm.

(2*R*,3*S*)-2-(diallylamino)-*N*,*N*-diethyl-3-fluoro-3-(naphthalen-2-yl)propanamide

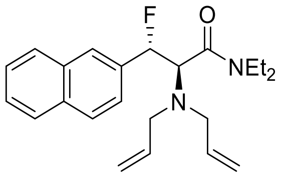

A 7 mL vial equipped with a stir bar was sequentially charged with the amino chloride substrate (36 mg, 0.1 mmol), Schreiner's urea (9.6 mg, 0.02 mmol), and KF (29 mg, 0.5 mmol) before adding 1,2-difluorobenzene (0.4 mL, 0.25 M). The reaction was placed in the center of a stir plate and was stirred at 1200 rpm at 60 °C (in an oil bath) for 48 h. Et_2O was used to flush the reaction through a short plug of silica gel and the filtrate was concentrated *in vacuo*. The β-fluoride was then isolated using flash column chromatography, with a gradient from 90:10 to 85:15 pentane/Et_2O to obtain the product as a white solid (25 mg, 69% yield). All spectra were in agreement with the racemic compound.). **^{1}H NMR** (500 MHz, $CDCl_3$) δ 7.92 – 7.82 (m, 4H), 7.52 (m, 3H), 6.01 (dd, *J* = 44.6, 9.4 Hz, 1H), 5.53 – 5.35 (m, 2H), 4.99 – 4.89 (m, 4H), 4.12 (dd, *J* = 9.4, 8.0 Hz, 1H), 3.79 – 3.53 (m, 2H), 3.36 – 3.03 (m, 6H), 1.27 (t, *J* = 7.2 Hz, 3H), 1.19 (t, *J* = 7.2 Hz, 3H);**^{13}C NMR** (126 MHz, $CDCl_3$) δ 170.0, 136.8, 135.7 (d, *J* = 18.7 Hz), 133.7, 133.0, 128.3, 128.1, 127.9, 127.3 (d, *J* = 7.7 Hz), 126.5, 126.4, 124.5 (d, *J* = 5.0 Hz), 116.8, 93.2 (d, *J* = 171.1 Hz), 61.2 (d, *J* = 31.9 Hz), 53.6, 42.1, 40.7, 14.9, 13.4; **^{19}F NMR** (376 MHz, $CDCl_3$) δ -177.0; **IR** (thin layer film) ν (cm^{-1}) = 2978, 1643, 1449, 1264, 1128, 1096, 1019, 1075, 975, 818, 747; **HRMS** (ESI^+) *m/z* calculated for $C_{23}H_{30}FN_2O^+$ $[M+H]^+$ 369.2337, found 369.2350; **$[\alpha]_D^{25\ °C}$ =** +32.0° (*c* = 0.0100 g/mL, DCM). **HPLC**: DAICEL CHIRALPAK® ID-3, Heptane:IPA 98:2, flow rate 1 mL/min, t_1 = 21.6 min (major isomer) and t_2 = 30.4 min (minor isomer), detection at 254 nm, 79.3% ee.

Investigation of Regiochemical Preference of Each Enantiomer of Substrate

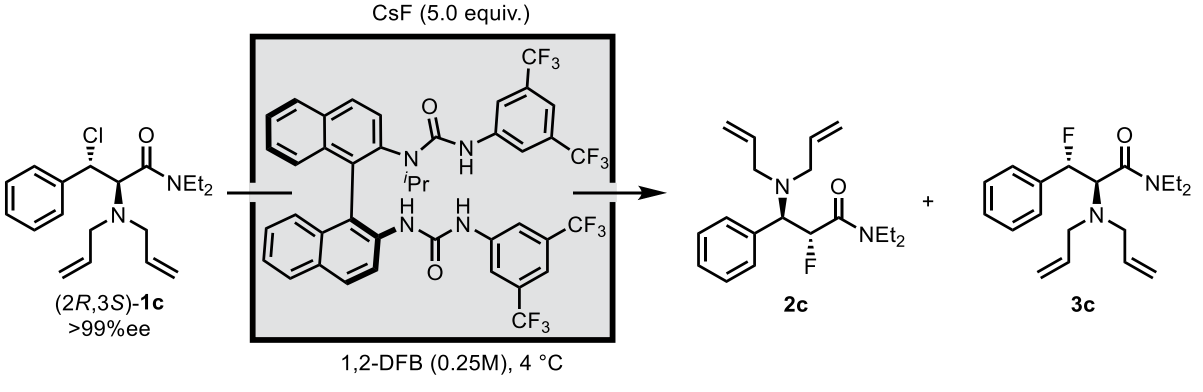

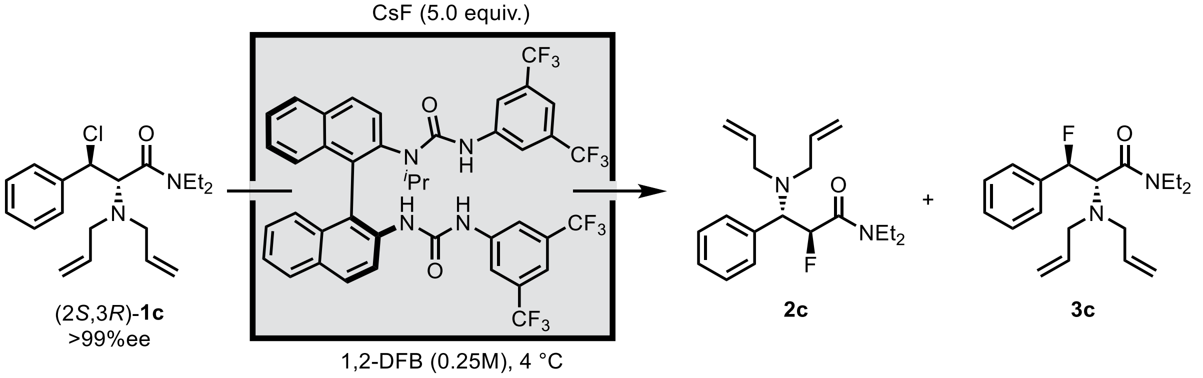

Entry	Enantiomer	Reaction Time (h)	NMR Conversion (%)[a]	r.r. (α-/β-F)[b]
1	(2*R*, 3*S*)	1	23	16.8:1
2	(2*R*, 3*S*)	4	70	17.8:1
3	(2*R*, 3*S*)	8	81	18.6:1
4[c]	(2*R*, 3*S*)	20	89	17.0:1
5	(2*S*, 3*R*)	1	9	1:2.5
6	(2*S*, 3*R*)	4	38	1:2.4
7	(2*S*, 3*R*)	8	36	1:2.3
8	(2*S*, 3*R*)	20	48	1:2.1

Reaction conditions: 0.1 mmol of amino chloride, (*S*)-^{i}Pr-BINAM bisurea (2 mol %), and fluoride salt listed above were stirred in 1,2-difluorobenzene (0.25 M) at 1200 rpm at 4 °C, at a distance of 2 cm from a stirring plate for the time listed above. Reactions were worked up by passing through a short plug of SiO_2 with diethyl ether, followed by concentration *in vacuo*. [a] Determined by quantitative ^{1}H NMR. [b] r.r. = regioisomeric ratio, determined by quantitative ^{19}F NMR of crude mixture. [c] Product was isolated from this trial the enantiomeric excess was measured (product was >99% ee) to confirm that there was no erosion of enantiopurity.

Computational Methods

General procedures: All geometry optimizations and frequency calculations were performed utilizing Gaussian 09, revision D.01.[17] The M06-2X[18] meta-hybrid functional was used in combination with Ahlrich's basis sets: we used the triple-zeta def2-TZVPPD basis set with a diffuse function for all heteroatoms and the double-zeta def2-SVP basis set without diffuse functions for C and H atoms.[19,20] We included solvent effects into our calculations via implementation of the CPCM model for dichloromethane.[21,22] All geometry optimizations were run with an ultrafine grid for numerical integrations. Single point energies were obtained using the ORCA 4.2.0 software package,[23] employing the range-separated ωB97X-D3 functional[24] including Grimme's D3 dispersion correction.[25] The ma-def2-TZVPP[26] basis set was used for heteroatoms and the def2-TZVPP basis set was employed for C and H atoms. Solvation was taken into account by the CPCM model for DCM. This computational protocol is consistent with earlier computational work and benchmarking studies of nucleophilic additions involving fluoride ions.[1]

Stationary points on the potential energy hypersurface were characterized according to their frequencies: computed structures with exactly one imaginary frequency were classified as transition states, structures with all positive frequencies as minima. We introduced thermochemical corrections using the *GoodVibes* package,[27] considering Grimme's recommendation[28] of a free-rotor cutoff value of 100 cm^{-1} for vibrational entropy terms. Unless otherwise stated, Gibbs Free energies were obtained at 298.15 K and 1 M concentration. Electrostatic Potential (ESP) isosurfaces were generated from the M06-2X densities and are shown for density values of 0.002 au. Intrinsic reaction coordinate (IRC) calculations were carried out to ensure that the intermediates (Int) of the different pathways connected to their corresponding transition structure (TS). The distortion-interaction/activation-strain model[29] was applied along the IRC and the intermolecular interaction energies were further decomposed into Pauli repulsion, electrostatic, orbital (polarization and charge-transfer), and dispersion contributions using the second-generation ALMO-EDA method implemented in Q-Chem 5.2.16.[30]

Method Validation

Computational methods employed for obtaining geometries were based on previous studies performed in our group on related systems involving nucleophilic additions of the fluoride anion. Single point energies were validated as shown below.[1,2] We studied two regioisomeric TSs in detail (Figure S1) to compare DFT-computed electronic energies against a wavefunction theory benchmark. Validation of our method was performed against the DLPNO-CCSD(T)[31] / aug-cc-pVTZ[32,33] / aug-cc-pVTZ/C / RIJCOSX[34] / def2/J[35] / TIGHTSCF standard as implemented in ORCA 4.2.0, obtaining good agreement with a difference in electronic energies of the two regiosisomeric TSs, $\Delta\Delta E^{\ddagger}$ = 1.8 kJ/mol (Table S10). We have noted in previously[36] that the default solvent excluded surface-type solute cavity for CPCM in ORCA 4.2.0 can exhibit problematic smearing of point charges, resulting in instabilities in solvation energies, even for closely-related structures. In order to avoid these potential issues, we used the van-der-Waals-type cavity instead.

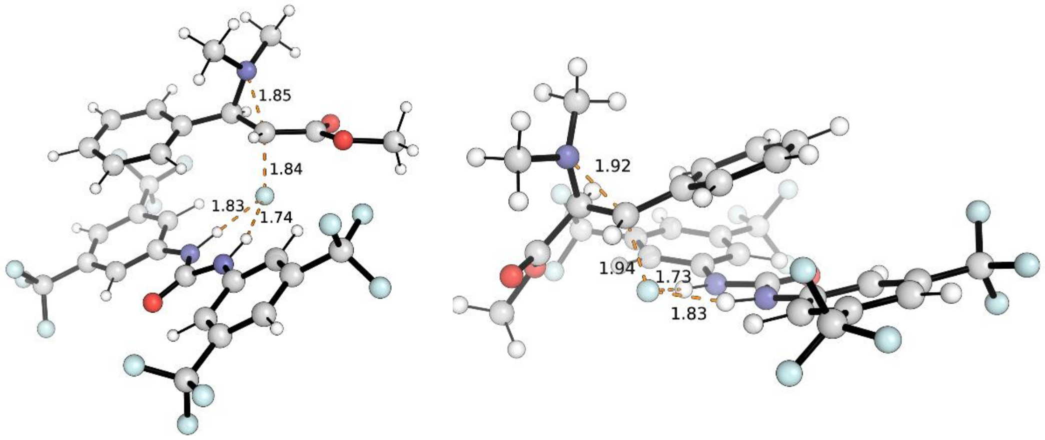

Figure S1: Transition state structures for α–(left) and β–fluorination, mediated by Schreiner's Urea

Table S10: Electronic energy differences of transition state structures for SU mediated a and b –fluorination depending on the method used. wB97X-D3/ma-def2-TZVPP (heteroatoms), def2-TZVPP (C,H); CPCM (DCM, surfacetype=vdW_Gaussian); DLPNO-CCSD(T) / aug-cc-pVTZ / aug-cc-pVTZ/C / RIJCOSX / def2/J / TIGHTSCF; CPCM (DCM, surfacetype=vdW_Gaussian)

method	$\Delta\Delta E^{\ddagger}$
ωB97X-D3	3.3 kJ/mol
DLPNO-CCSD(T)	1.5 kJ/mol

The relation of charge and kinetic selectivity

Hydrogen bond donor catalysts are able to influence a variety of physical and chemical properties of a coordinated anion. We investigated the specific effect of charge modulation in the catalyst:fluoride complex in the ground state upon the kinetic reactivity and selectivity predicted for fluoride addition to an unsymmetrical aziridinium electrophile. Pleasingly, we discovered a Linear Free Energy Relationship (LFER) between this electronic descriptor and regioselectivity: there is a linear dependence on the difference in predicted activation barriers for the two regioisomeric pathways on fluoride's residual charge. We found this to be consistent among Mulliken, Lowdin or Hirshfeld charges (Table S11).

Table S11: Different types of charges assigned to the complexes / free fluoride described in the main manuscript and the Free Energy difference of the regioisomeric transition states (in kJ/mol)

complex	Lowdin	Mulliken	Hirshfeld	$\Delta\Delta G^{\ddagger}$
**I**	-0.71	-0.85	-0.61	1.5

**II**	-0.64	-0.82	-0.57	3.1
**III**	-0.35	-0.71	-0.40	10.5
**IV**	-0.26	-0.62	-0.37	14.0
free fluoride	-1	-1	-1	-8.8

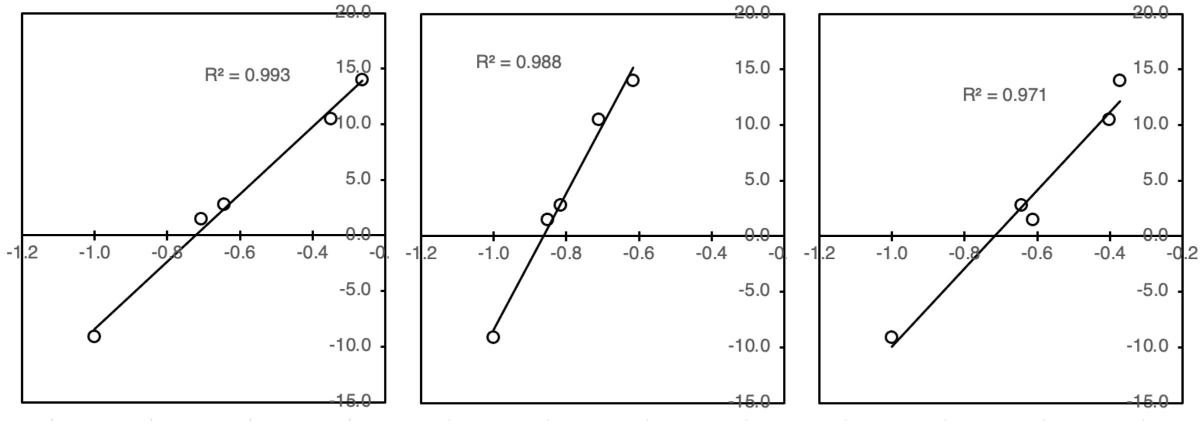

Computed regioselectivity (kJ/mol) vs. Lowdin, Mulliken and Hirshfeld charge on fluoride (au).

The relative energy differences of the unoccupied molecular orbitals on the α and β – position of the aziridinium intermediate: In order for the interaction between an electrophile and a nucleophile to be maximised, the energy differences of the LUMO and HOMO, respectively, should ideally be minimized (among other factors). Having two electrophilic sites within the same molecule, and desiring reactivity of both of these sites to the same nucleophile, small energy differences of the corresponding unoccupied orbitals can therefore be considered feasible.

Based on this, we can understand why our methodology, that is, the tuning of the residual charge on fluoride by HBD catalysts is successful for the intermediate aziridinium ion A, but not for B (Table 12 and Figure S2): the β-position in B does not feature an unoccupied orbital of similar energy to the corresponding orbital at the alpha position (which is now the LUMO). A modulation of charge alone is not successful in turning this intrinsic preference over for B, the overlap of the C-N σ^* orbital at the β-position with an extended π system is suggested to be crucial for energetic feasibility of our method.

Table S12: Energetic differences of unoccupied orbitals with significantly sized lobes at the alpha and beta positions depending on the nature of the substituent R.

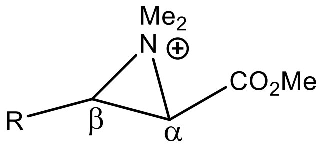

compound A: R = Ph
compound B: R = 1-cyclopentyl

	aziridinium **A**	aziridinium **B**
α position	LUMO+1 (MO57); 0.02122 H	LUMO (MO55); 0.02627 H
β position	LUMO (MO56); 0.02394 H	LUMO+5 (MO60); 0.11109 H
ΔE (kJ/mol)	7.1	222.7

For visualization of the corresponding orbitals for compound A see main manuscript, for compound B see below (Figure S2)

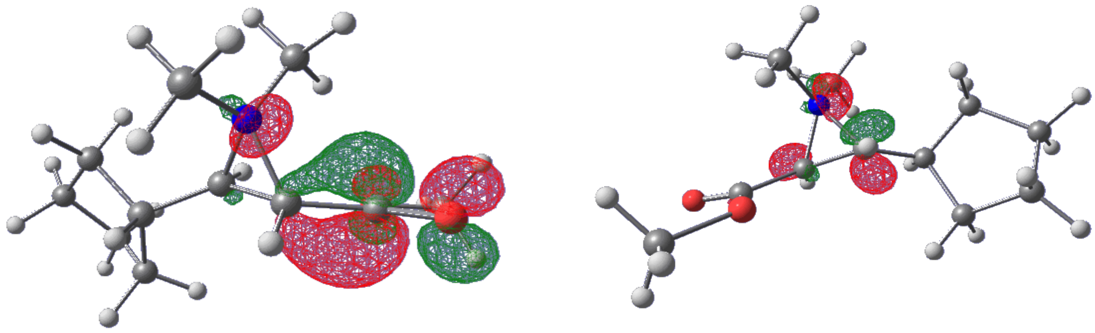

Figure S2: Isosurfaces of lowest unoccupied molecular orbital (compound B) shown for the alpha position (left) and beta position (right). Contour value 0.074651 (left); 0.062208 (right); visualized via Chemcraft version 1.8.

The Potential Energy Surface:

In order to find energetically low-lying conformers of the close-contact ion pair consisting of the Schreiner's Urea Fluoride complex and the aziridinium cation we embarked on a systematic search of the conformational space employing the CREST program version 2.6.[37,38] We obtained eight conformers from this approach[36] and augmented this by adding four structures obtained from a manual search (IP-conf1-4). All structures were further optimized, and single point energies were evaluated and thermodynamically corrected as described above. Eight unique structures were obtained at the DFT level of theory (Table S13).

Table S13: Energetically low-lying conformers of the ion pair described in the PES shown in the main manuscript.

Conformer	ΔG (kJ/mol)
IP-conf-1	12.7
IP-conf-2	16.1
IP-conf-3	7.5
IP-conf-4	1.9
IP-conf-5	7.4
IP-conf-6	9.8
IP-conf-7	7.4
IP-conf-8	7.3
IP-conf-9	8.5
IP-conf-10	7.2
IP-conf-11	8.4
IP-conf-12	0.0

Apart from the highest energy conformer (IP-conf-2) and the lowest energy conformer (IP-conf-12), a common structural feature in all structures involves π–π stacking of the phenyl ring of the substrate and one of the aryl rings of the catalyst's backbone: the distance between said ring systems is typically less than 3.5 Å. In the most stable of the structures, where aryl-aryl π–π stacking is geometrically feasible, IP-conf-4, the fluoride is close to the positively polarized ester carbon, presumably benefiting from electrostatic stabilization (Figure S3). IP-conf-12, however, seems to compensate for the lack of intermolecular π–π stabilization of the respective aromatic systems by an additional H bonding interaction of fluoride with one of the H atoms of the aziridinium ring system. In IP-conf-2, the geometry seems to allow for weak stabilizations of fluoride's negative charge by interactions with the methyl protons and the positively charged nitrogen as well as the electron-poor ester carbon.

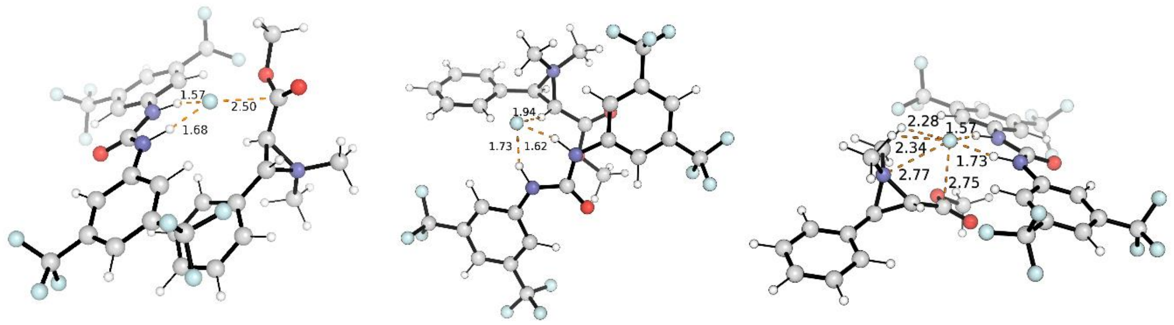

IP-conf-4: 1.9 kJ/mol   IP-conf-12: 0.0 kJ/mol   IP-conf-2: 16.2 kJ/mol

Figure S3: Structural comparison of three ion pair conformers

Distortion-Interaction / Activation-Strain analysis:

The overall distortion along the reaction pathway for the Schreiner Urea mediated fluorination is dominated by the distortion of the aziridinium substrate (Figure S4). The energetic zero is represented

by the infinitely separated aziridinium ion and the SU-fluoride complex. For comparability reasons with the non-catalyzed fluorination, we projected the IRC on a new coordinate, defined by the difference of the breaking C-N bond of the aziridinium minus the forming C-F bond.

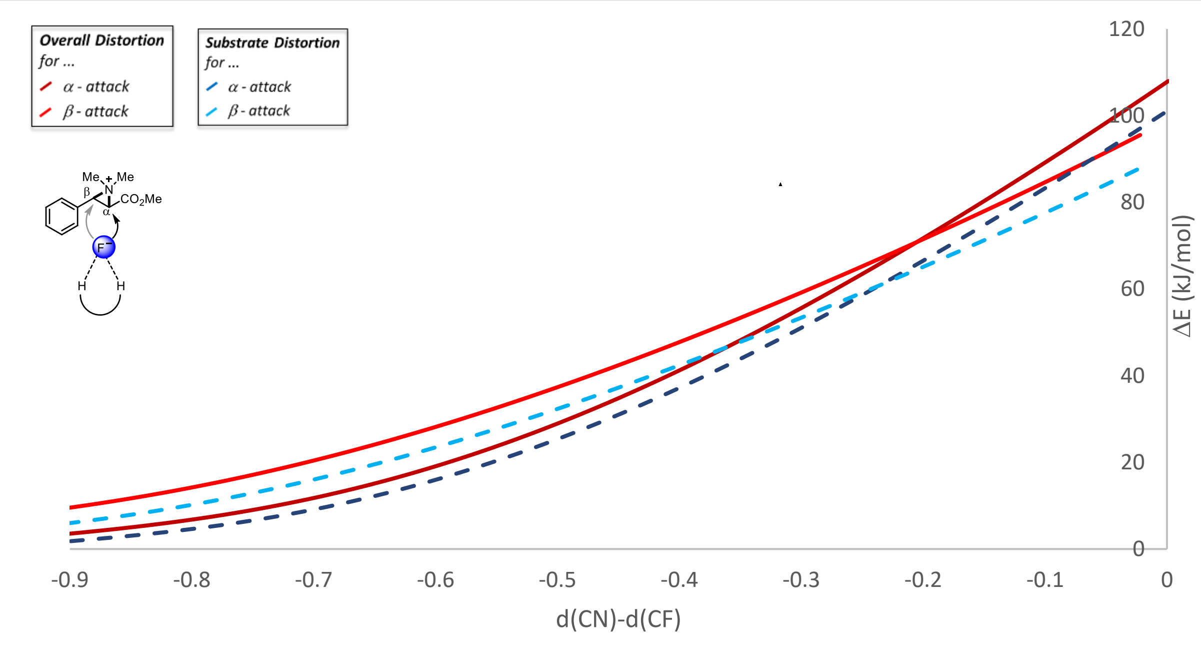

Figure S4: Distortion-Interaction/activation-strain analysis for the regioisomeric fluorination of the aziridinium cation model with a $[SU\text{-}F]^-$ complex.

Tabulated energies of computed structures

Table S14: Method validation; electronic energies

	Energies / Ha	
	DLPNO-CCSD(T)	ωB97x-D3
SU_F-TS-alpha	-2803.477513	-2807.941986
SU_F-TS-beta	-2803.478084	-2807.943257

Table S15: The relationship between charge and selectivity, only lowest energy conformers located are shown below

		Energies / Ha			
		G-qh (opt)	E (opt)	E(sp)	corrected G
SM	aziridinium cation	-671.14175	-671.370407	-671.995577	-671.7669197
	complex I	-865.111836	-865.349758	-866.1067829	-865.8688609
	complex II	-2134.86383	-2135.05318	-2135.942498	-2135.753148
	complex III	-711.872858	-712.007004	-712.579966	-712.4458198
	complex IV	-478.292565	-478.3118	-478.4458462	-478.4266112

TS	TS-complex-I-alpha	-1536.239336	-1536.730194	-1538.107847	-1537.616989
	TS-complex-I-beta	-1536.239259	-1536.730319	-1538.108602	-1537.617542
	TS-complex-II-alpha	-2805.989261	-2806.431867	-2807.941986	-2807.49938
	TS-complex-II-beta	-2805.988001	-2806.4307	-2807.943257	-2807.500558
	TS-complex-III-alpha	-1382.991466	-1383.377849	-1384.572156	-1384.185773
	TS-complex-III-beta	-1382.993189	-1383.377717	-1384.574292	-1384.189764
	TS-complex-IV-alpha	-1149.398205	-1149.668186	-1150.425138	-1150.155157
	TS-complex-IV-beta	-1149.400792	-1149.669071	-1150.428766	-1150.160487
	TS-F-anion-alpha	-771.138478	-771.362566	-771.997194	-771.773106
	TS-F-anion-beta	-771.133066	-771.355957	-771.9925096	-771.7696186

Table S16: phenyl and cyclopentyl substituted aziridinium cation

		Energies / Ha			
		G-qh (opt)	E (opt)	E(sp)	corrected G
aziridinium-cation	Phenyl	-671.14175	-671.370407	-671.995577	-671.7669197
	Cyclopentyl	-635.433719	-635.702592	-636.314065	-636.045192

Table S17: Potential Energy Surface: ion pair

	Energies / Ha			
	G-qh (opt)	E (opt)	E(sp)	corrected G
IP-conf-1	-2806.011326	-2806.454294	-2807.962281	-2807.5193
IP-conf-2	-2806.010034	-2806.453273	-2807.961256	-2807.518
IP-conf-3	-2806.012721	-2806.456592	-2807.965183	-2807.5213
IP-conf-4	-2806.015114	-2806.459437	-2807.967759	-2807.5234
IP-conf-5	-2806.012741	-2806.456591	-2807.965192	-2807.5213
IP-conf-6	-2806.011837	-2806.456209	-2807.964778	-2807.5204
IP-conf-7	-2806.012743	-2806.456592	-2807.965187	-2807.5213
IP-conf-8	-2806.01276	-2806.456592	-2807.96519	-2807.5214
IP-conf-9	-2806.012417	-2806.456201	-2807.964701	-2807.5209
IP-conf-10	-2806.012791	-2806.456591	-2807.965197	-2807.5214
IP-conf-11	-2806.014069	-2806.458663	-2807.96554	-2807.5209
IP-conf-12	-2806.01402	-2806.457261	-2807.967394	-2807.5242

Table S18: Potential Energy Surface: TS

		Energies / Ha			
		G-qh (opt)	E (opt)	E(sp)	corrected G
uncatalysed	TS-alpha-1	-771.133888	-771.360816	-771.9953749	-771.7684469
	TS-alpha-2	-771.13546	-771.362566	-771.997194	-771.770088
	TS-beta-1	-771.130047	-771.355957	-771.9924877	-771.7665777
	TS-beta-2	-771.130046	-771.354225	-771.9909014	-771.7667224

	TS-beta-3	-771.130047	-771.355957	-771.9925096	-771.7665996
SU mediated	TS-alpha-1	-2805.987327	-2806.42979	-2807.940033	-2807.49757
	TS-alpha-2	-2805.989261	-2806.431867	-2807.941986	-2807.49938
	TS-beta-1	-2805.9879	-2806.430764	-2807.942789	-2807.499925
	TS-beta-2	-2805.986897	-2806.428881	-2807.940885	-2807.498901
	TS-beta-3	-2805.988001	-2806.4307	-2807.943257	-2807.500558

Table S19: Potential Energy Surface: products and product complexes.

		Energies / Ha			
		G-qh (opt)	E (opt)	E(sp)	corrected G
final products	alpha-conf1	-771.189712	-771.418895	-772.048071	-771.8188883
	alpha-conf2	-771.190391	-771.419578	-772.048739	-771.8195517
	beta-conf1	-771.192489	-771.421496	-772.050353	-771.8213463
	beta-conf2	-771.191033	-771.420026	-772.048956	-771.819963
products complexed to SU	alpha-conf1	-2806.0261	-2806.471687	-2807.978007	-2807.532419
	alpha-conf2	-2806.02753	-2806.473307	-2807.97935	-2807.533575
	beta-conf1	-2806.02845	-2806.474584	-2807.980823	-2807.534689
	beta-conf2	-2806.02694	-2806.472436	-2807.978696	-2807.533198
	beta-conf3	-2806.03264	-2806.478747	-2807.982931	-2807.536821

Copies of HPLC Traces: Enantiospecific Synthesis of α- and β-Fluoride Products using Jørgensen Epoxidation Route

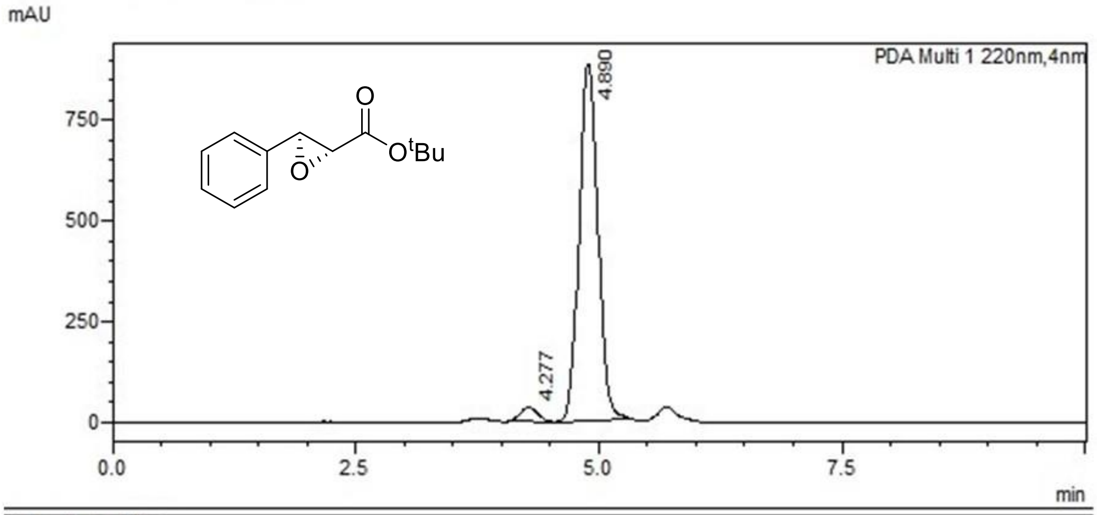

<Peak Table>

Peak#	Ret. Time	Area%
1	4.277	3.241
2	4.890	96.759

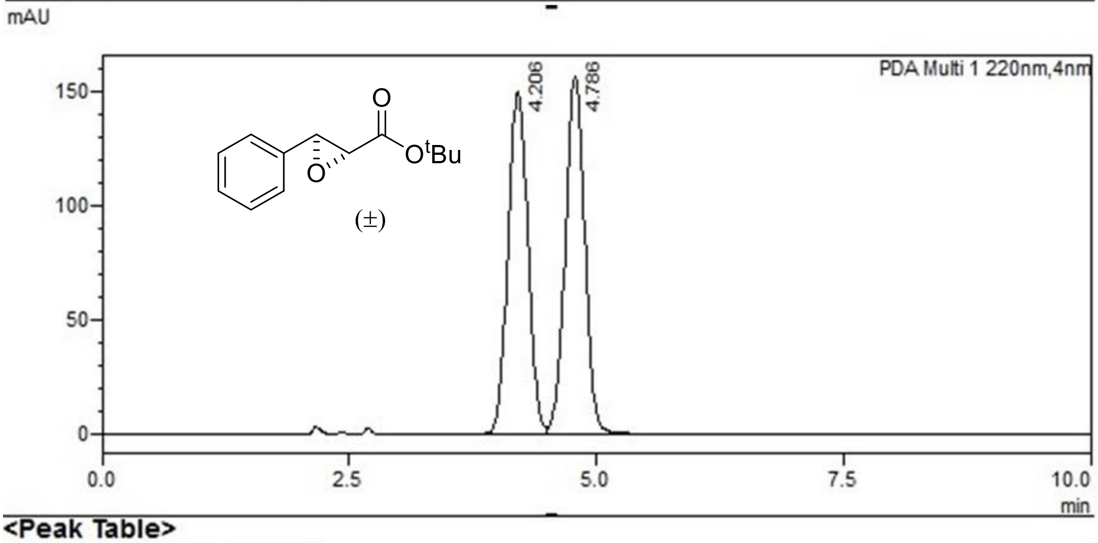

<Peak Table>

Peak#	Ret. Time	Area%
1	4.206	49.568
2	4.786	50.432

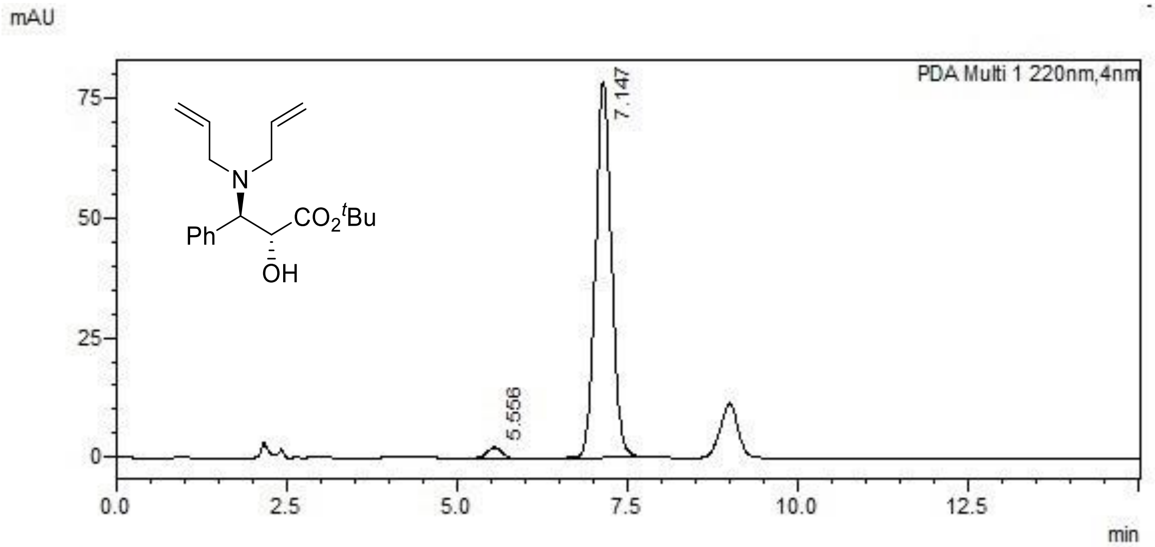

<Peak Table>

Peak#	Ret. Time	Area%
1	5.556	2.751
2	7.147	97.249

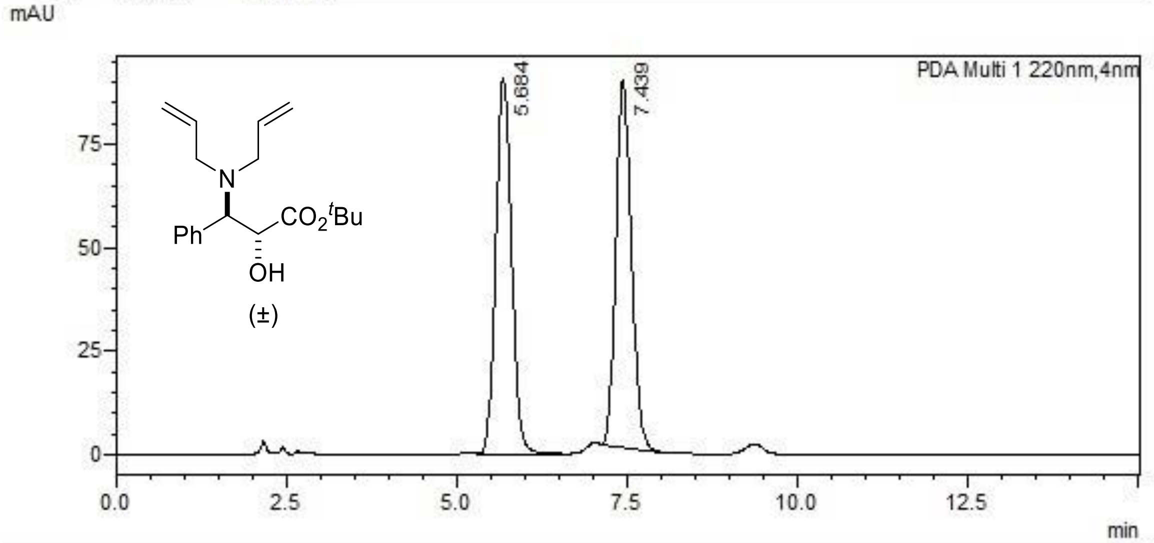

<Peak Table>

Peak#	Ret. Time	Area%
1	5.684	51.084
2	7.439	48.916

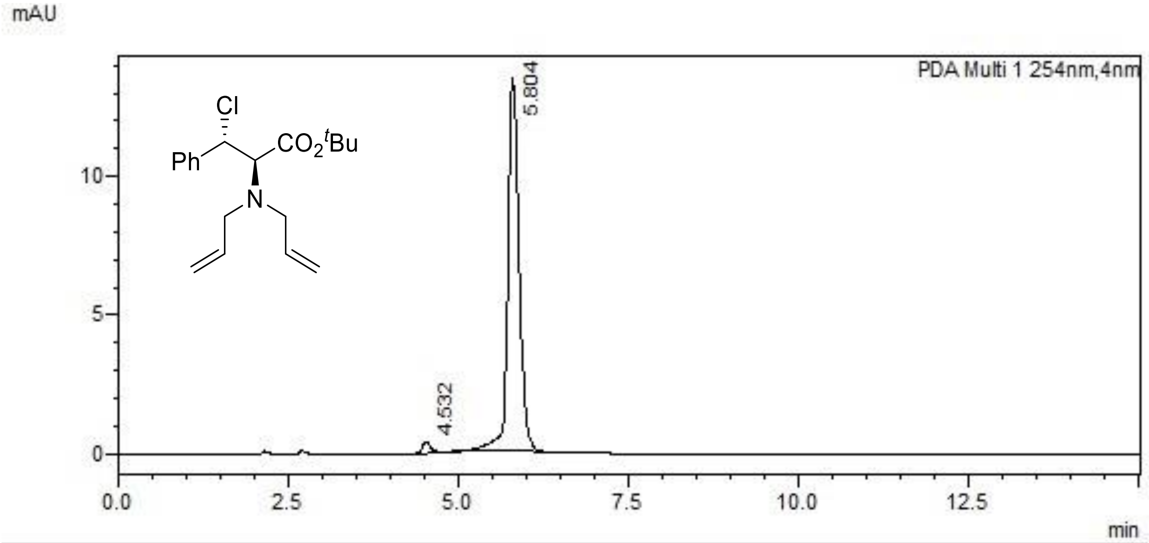

<Peak Table>

Peak#	Ret. Time	Area%
1	4.532	2.076
2	5.804	97.924

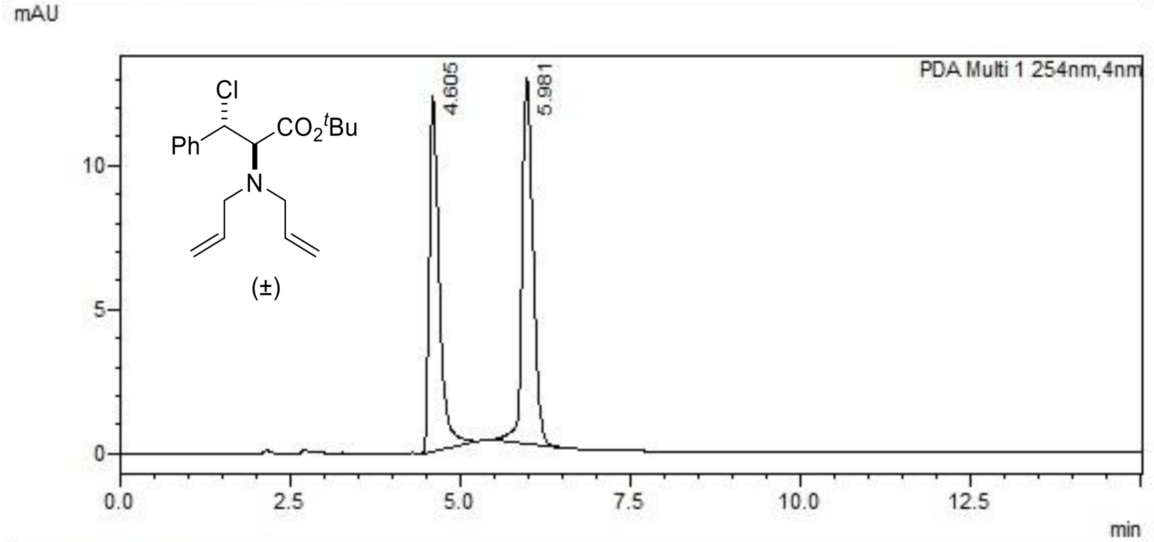

<Peak Table>

Peak#	Ret. Time	Area%
1	4.605	46.843
2	5.981	53.157

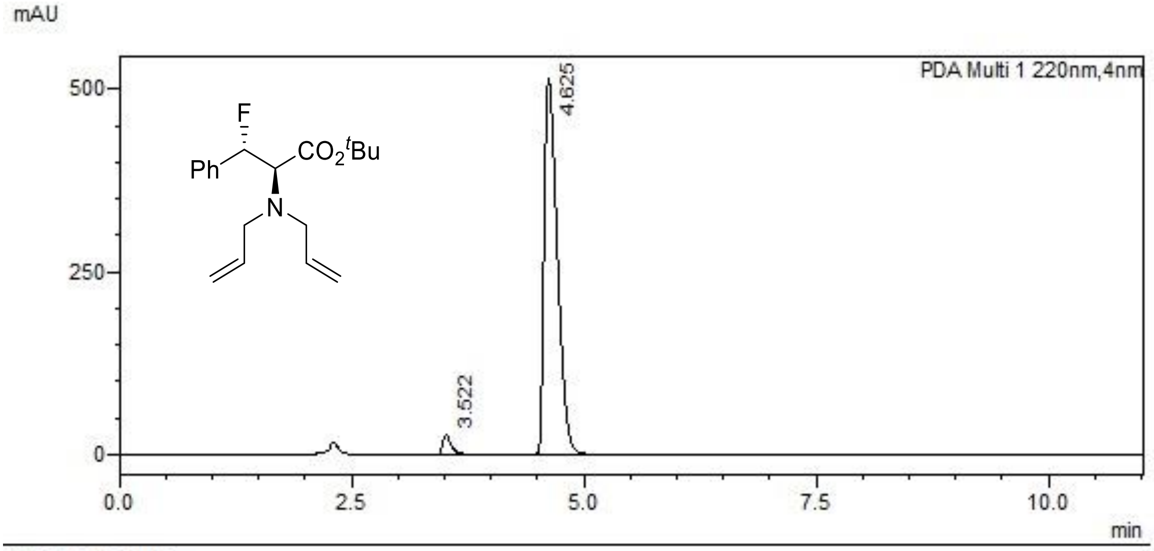

<Peak Table>

Peak#	Ret. Time	Area%
1	3.522	3.348
2	4.625	96.652

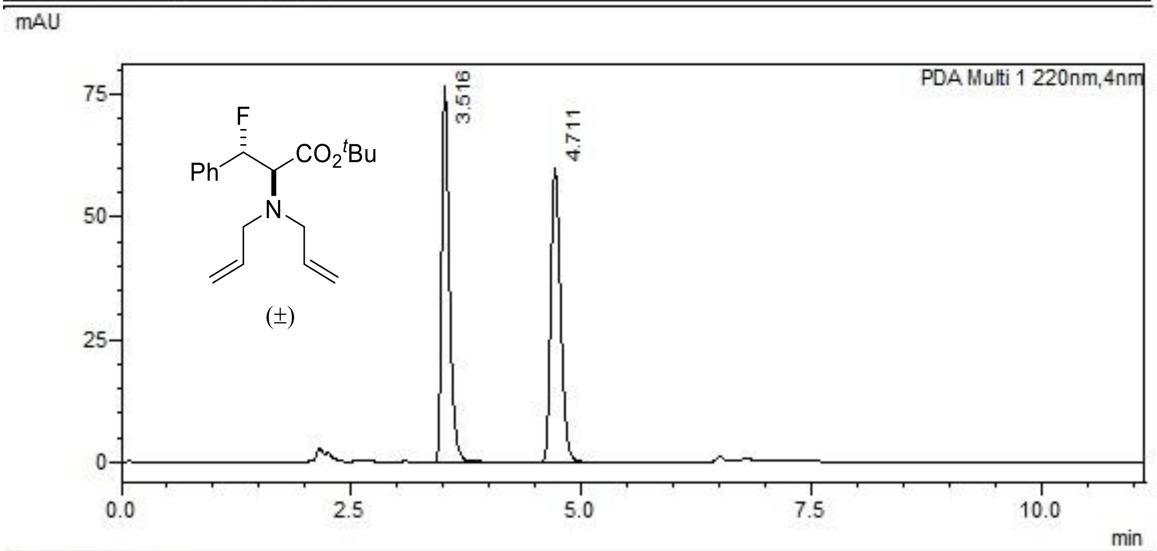

<Peak Table>

Peak#	Ret. Time	Area%
1	3.516	50.109
2	4.711	49.891

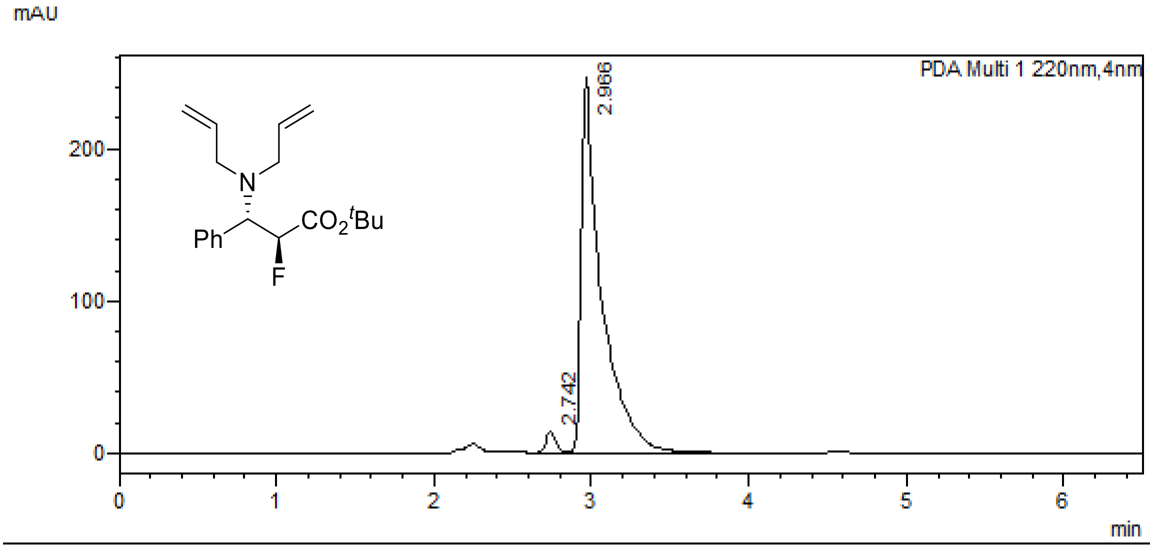

<Peak Table>

Peak#	Ret. Time	Area%
1	2.742	2.757
2	2.966	97.243

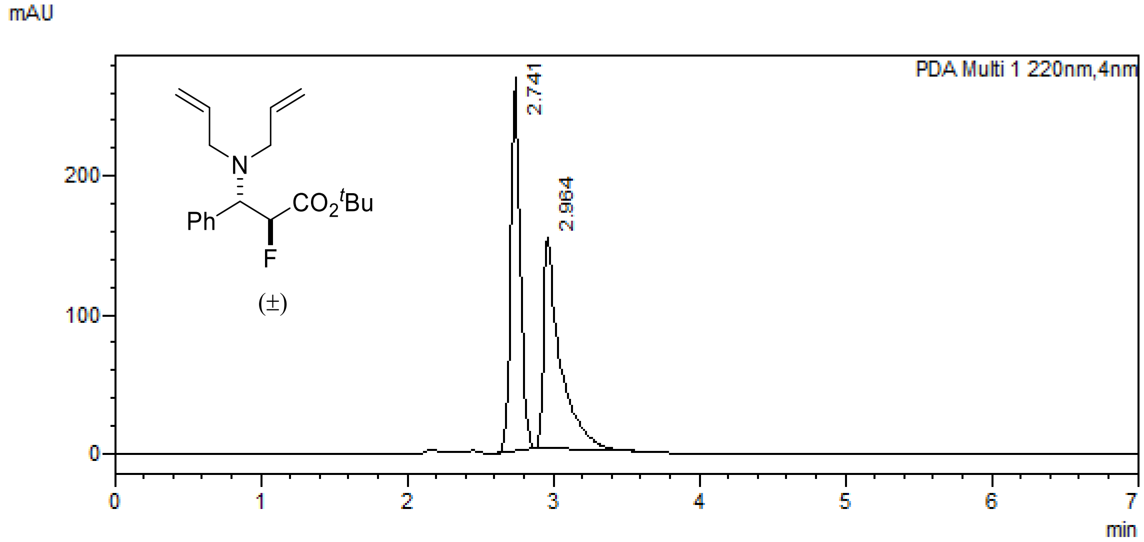

<Peak Table>

Peak#	Ret. Time	Area%
1	2.741	50.033
2	2.964	49.967

Copies of HPLC Traces: Synthesis of Enantioenriched Amino Chloride Amide Substrates via Shibasaki Epoxidation Route

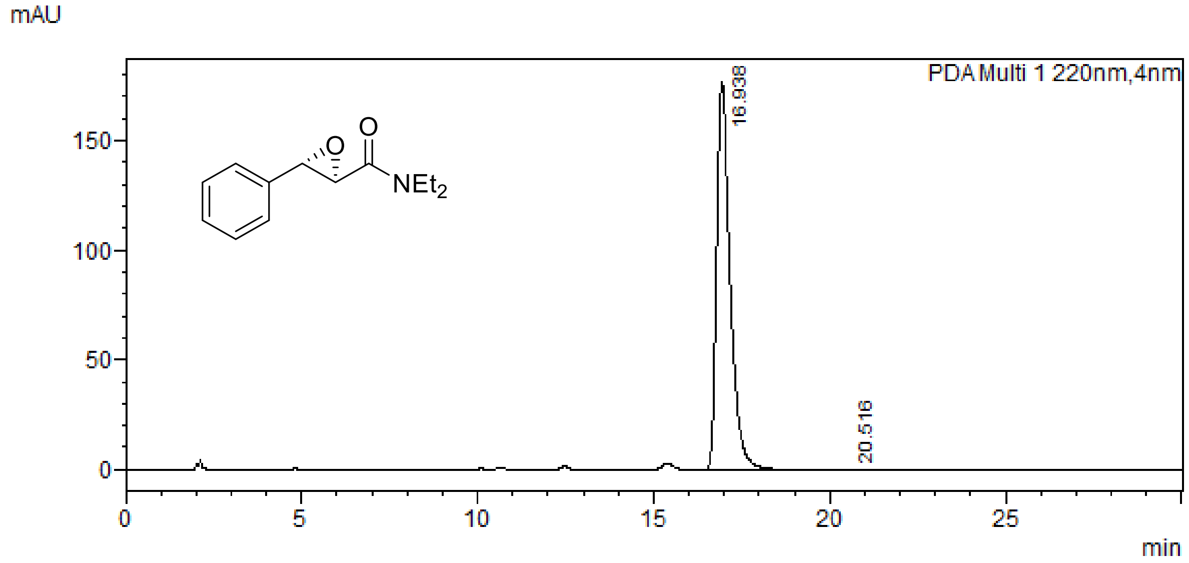

<Peak Table>

PDA Ch1 220nm

Peak#	Ret. Time	Area%
1	16.938	99.733
2	20.516	0.267
Total		100.000

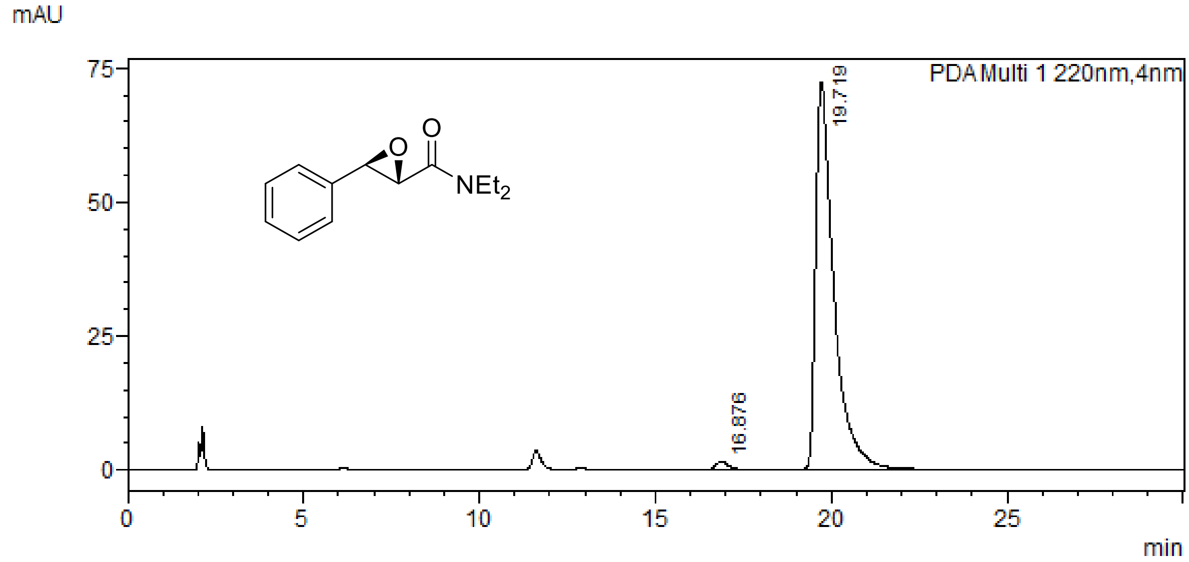

<Peak Table>

PDA Ch1 220nm

Peak#	Ret. Time	Area%
1	16.876	1.417
2	19.719	98.583
Total		100.000

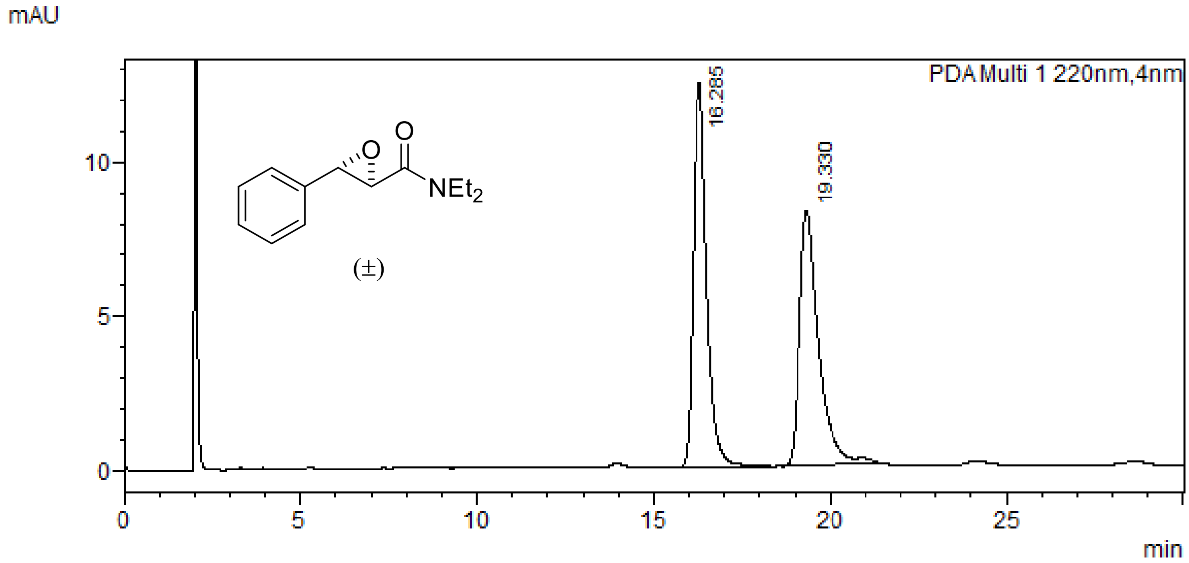

<Peak Table>

PDA Ch1 220nm

Peak#	Ret. Time	Area%
1	16.285	50.541
2	19.330	49.459
Total		100.000

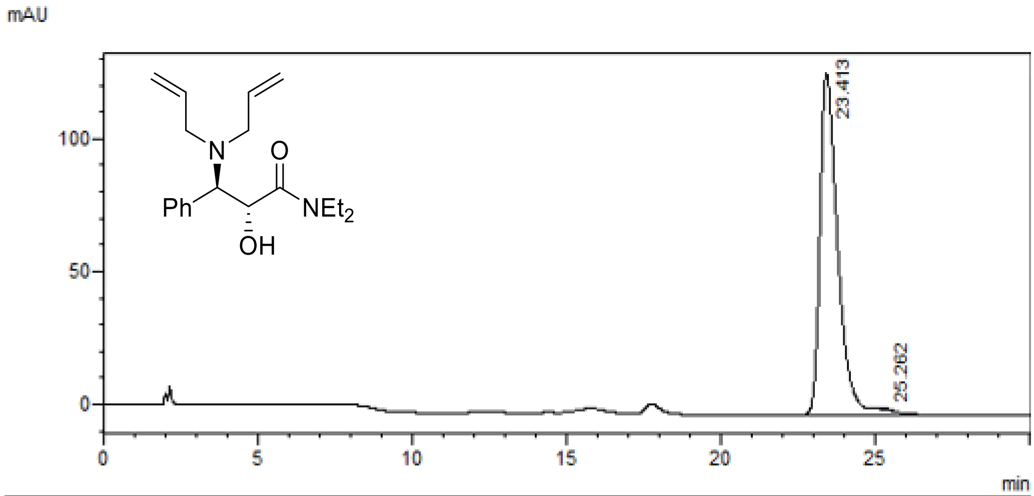

<Peak Table>

Peak#	Ret. Time	Area%
1	23.413	99.618
2	25.262	0.382

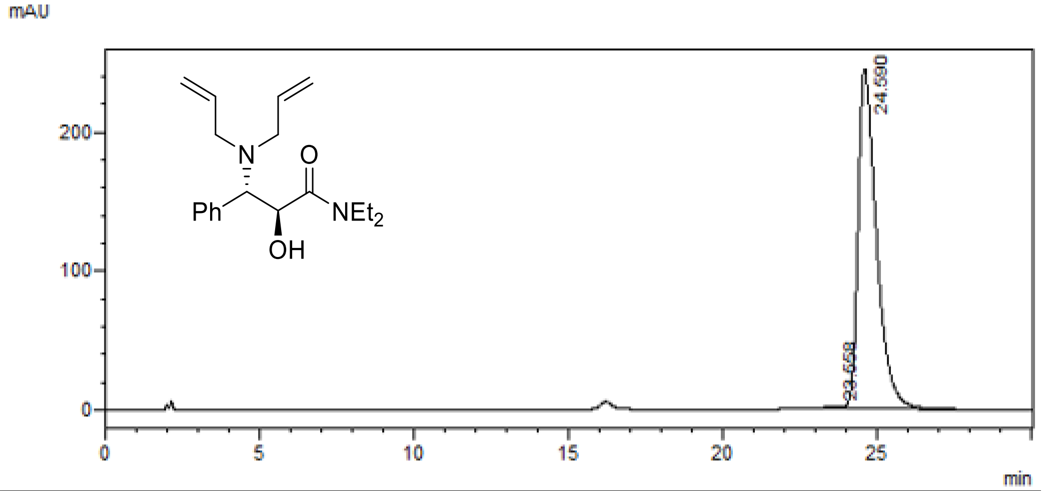

<Peak Table>

Peak#	Ret. Time	Area%
1	23.558	0.847
2	24.590	99.153

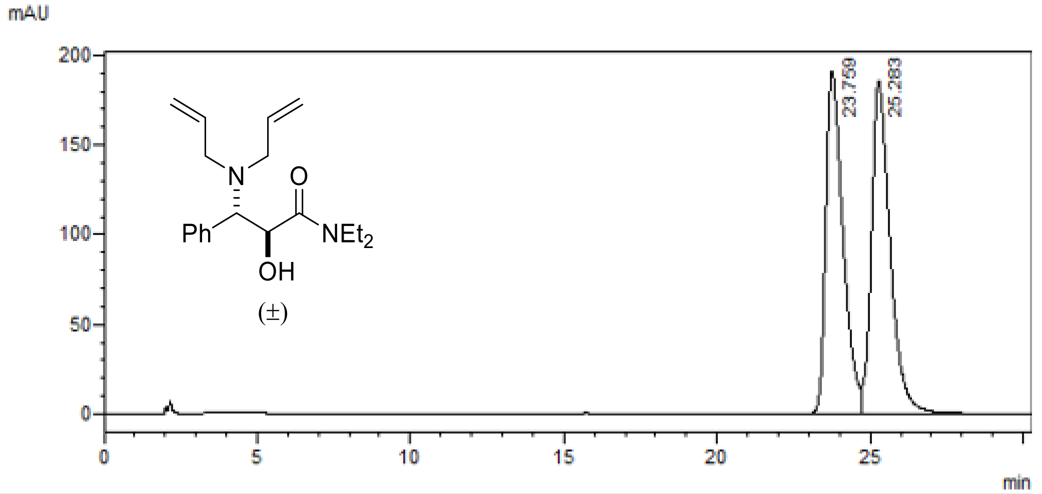

<Peak Table>

Peak#	Ret. Time	Area%
1	23.759	48.578
2	25.283	51.422

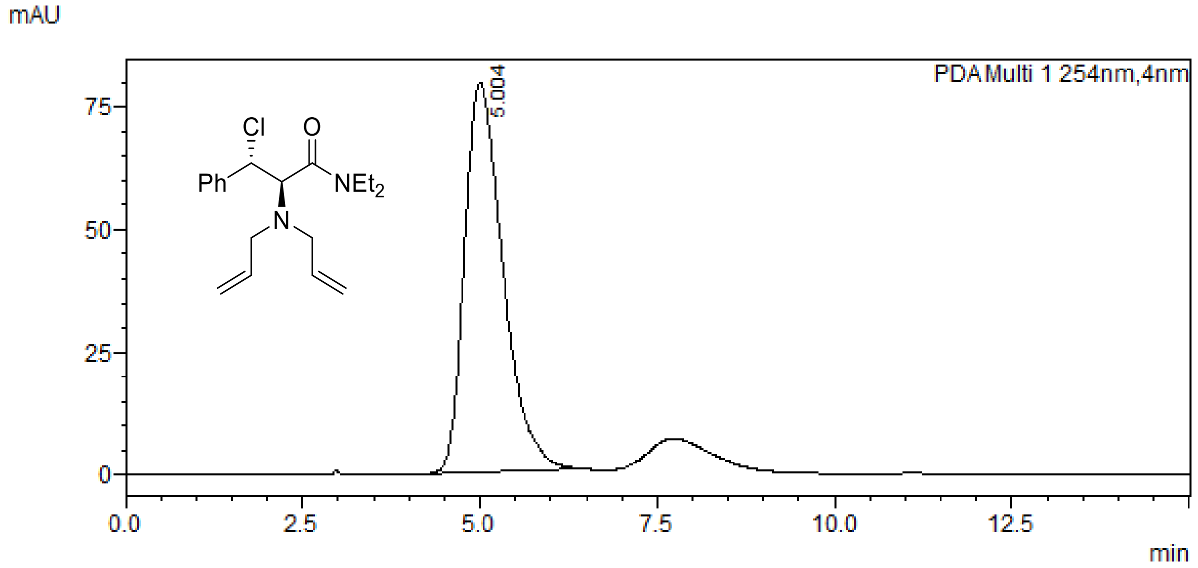

<Peak Table>

PDA Ch1 254nm

Peak#	Ret. Time	Area%
1	5.004	100.000
Total		100.000

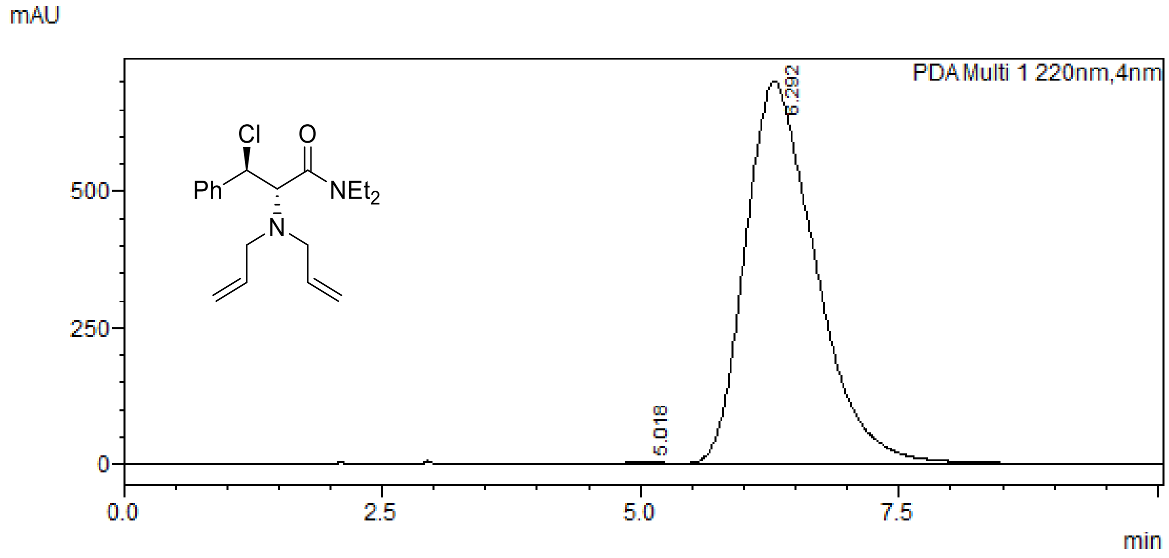

<Peak Table>

PDA Ch1 220nm

Peak#	Ret. Time	Area%
1	5.018	0.207
2	6.292	99.793
Total		100.000

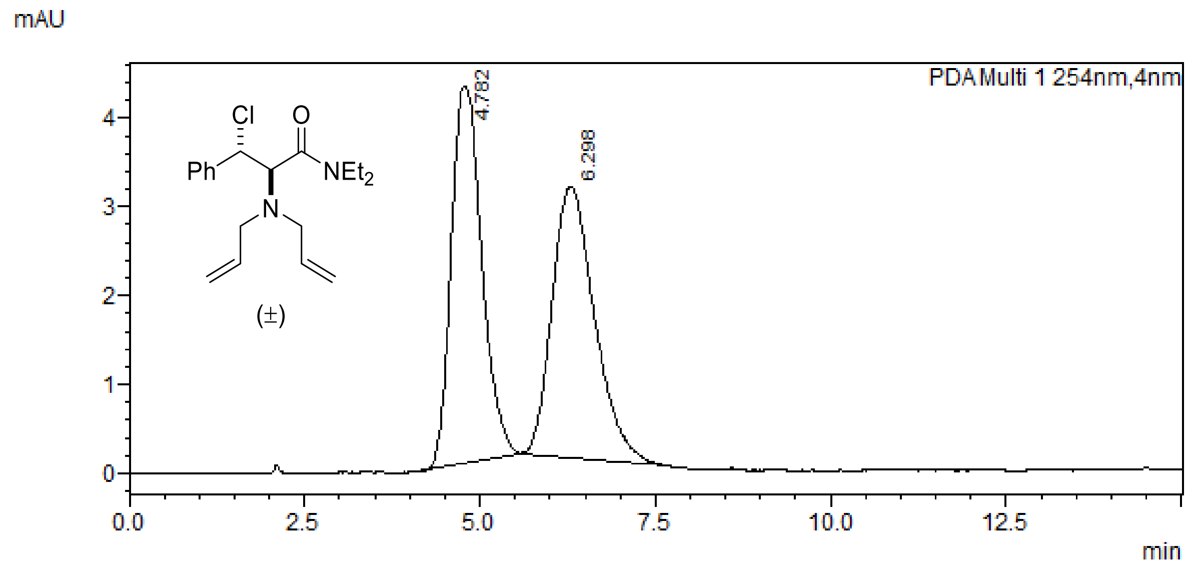

<Peak Table>

PDA Ch1 254nm

Peak#	Ret. Time	Area%
1	4.782	49.946
2	6.298	50.054
Total		100.000

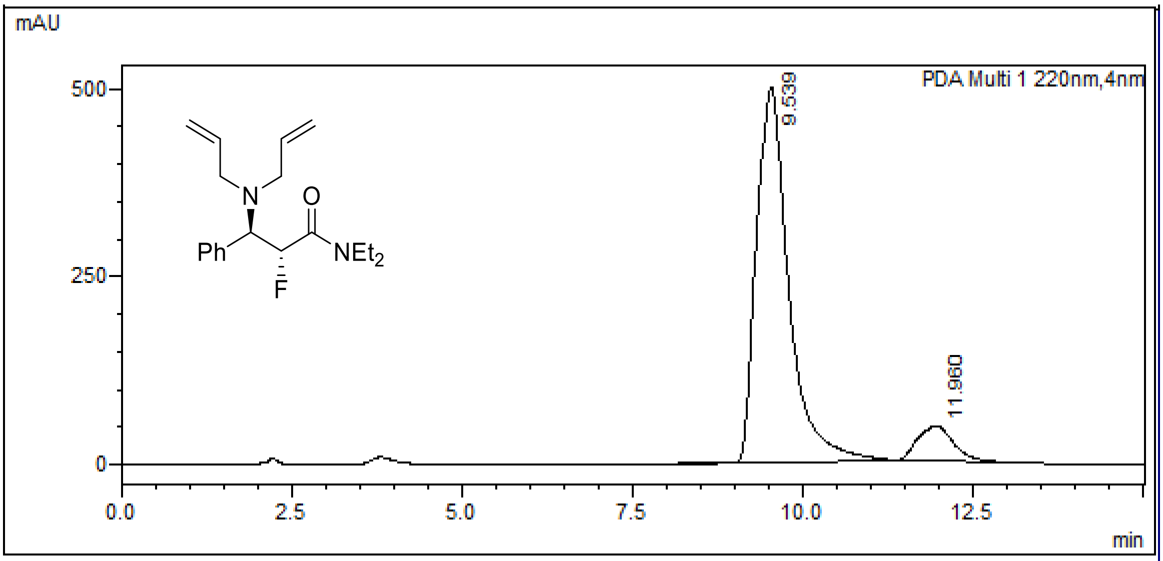

<Peak Table>

PDA Ch1 220nm

Peak#	Ret. Time	Area%
1	9.539	90.638
2	11.960	9.362

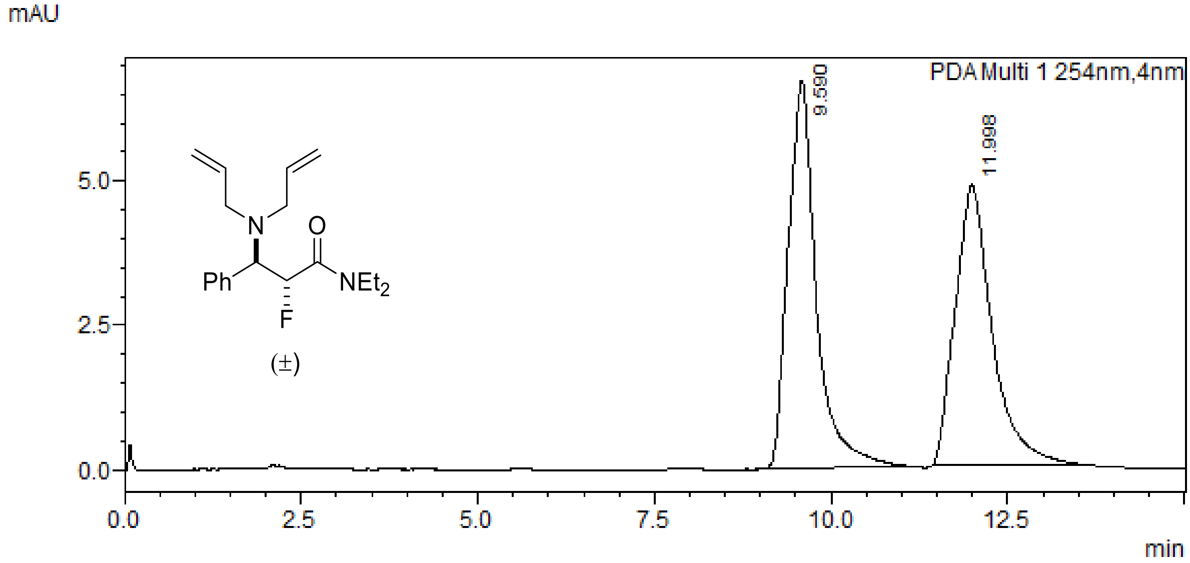

<Peak Table>

PDA Ch1 254nm

Peak#	Ret. Time	Area%
1	9.590	51.191
2	11.998	48.809
Total		100.000

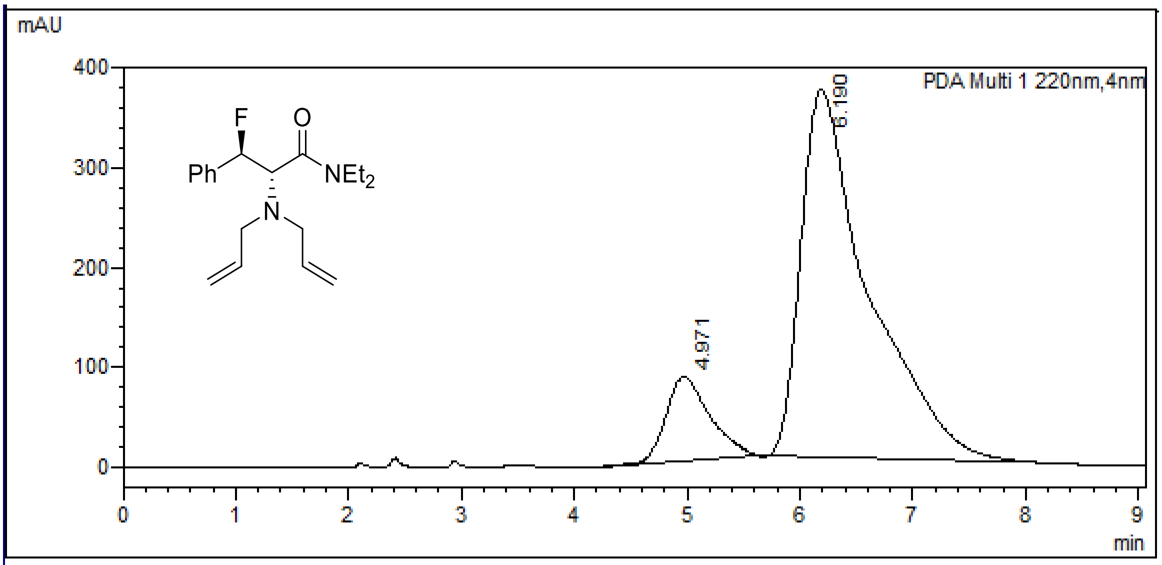

<Peak Table>

PDA Ch1 220nm

Peak#	Ret. Time	Area%
1	4.971	12.843
2	6.190	87.157

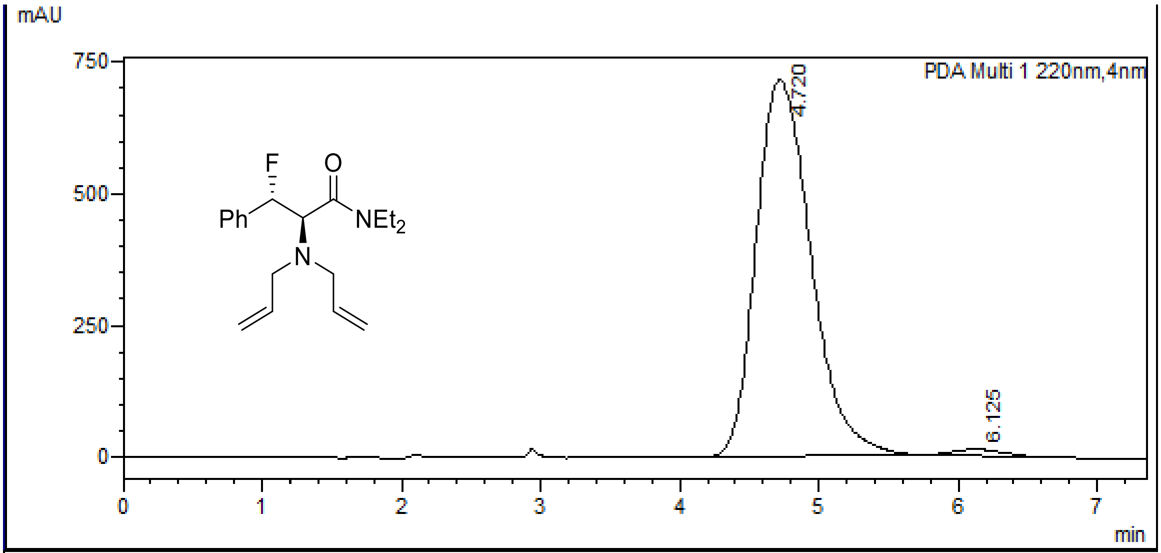

<Peak Table>

PDA Ch1 220nm

Peak#	Ret. Time	Area%
1	4.720	98.567
2	6.125	1.433

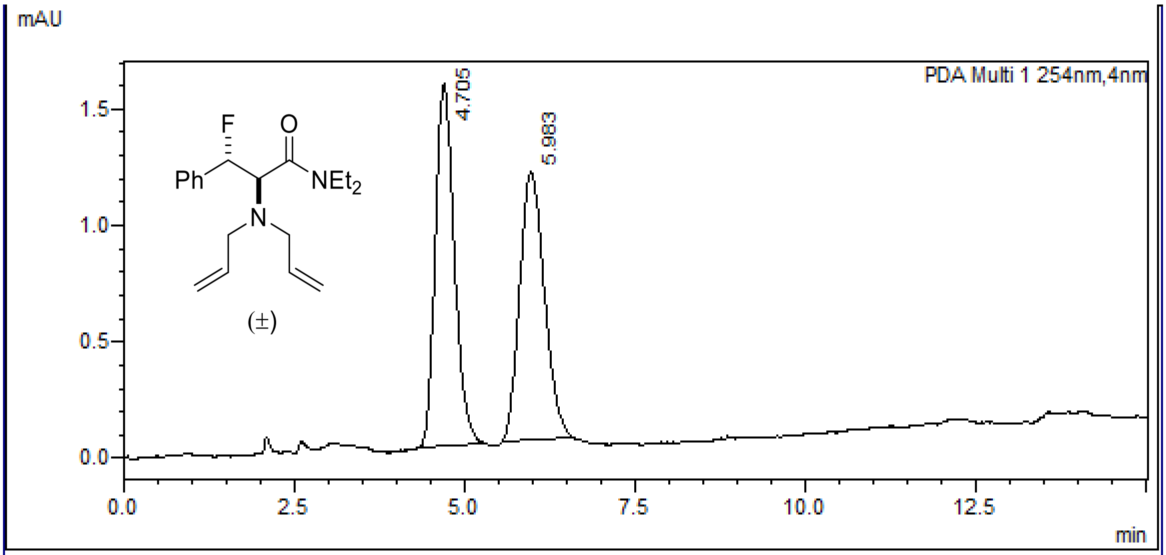

<Peak Table>

PDA Ch1 254nm

Peak#	Ret. Time	Area%
1	4.705	50.671
2	5.983	49.329

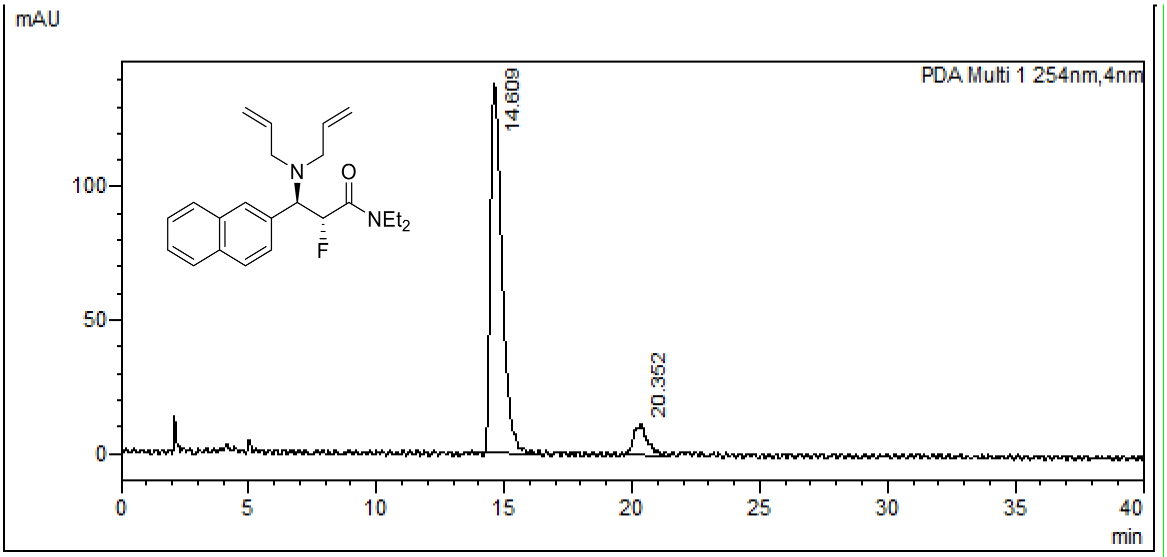

<Peak Table>

PDA Ch1 254nm

Peak#	Ret. Time	Area%
1	14.609	91.180
2	20.352	8.820

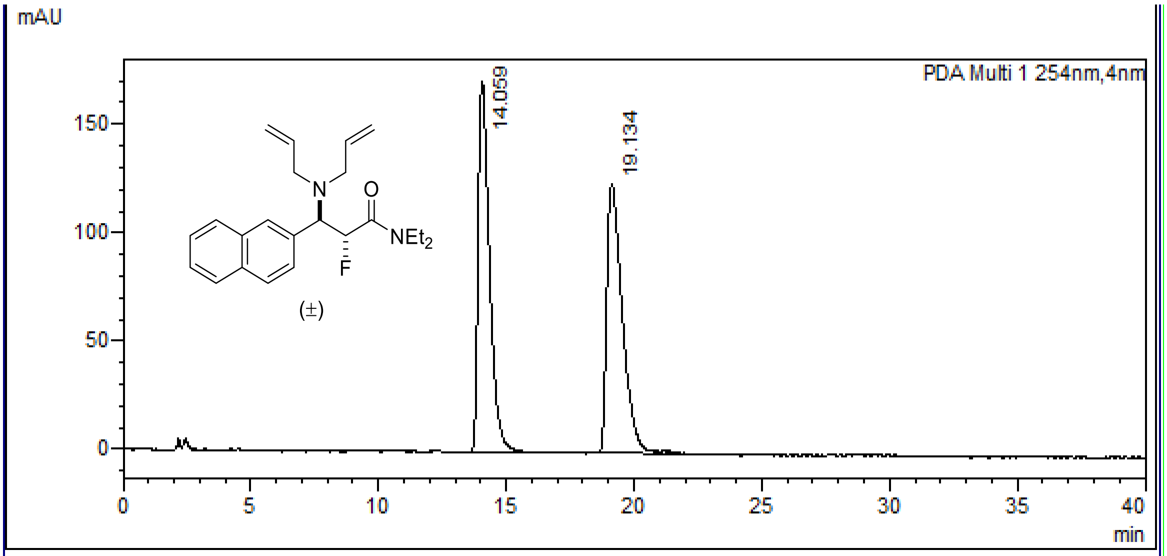

<Peak Table>

PDA Ch1 254nm

Peak#	Ret. Time	Area%
1	14.059	50.314
2	19.134	49.686

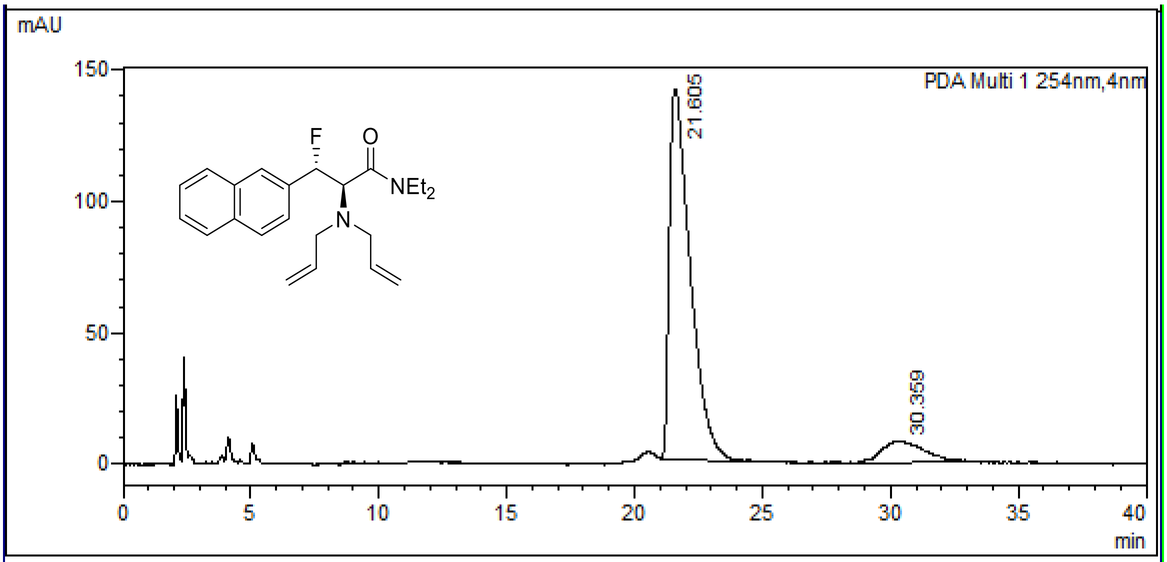

<Peak Table>

PDA Ch1 254nm

Peak#	Ret. Time	Area%
1	21.605	89.654
2	30.359	10.346

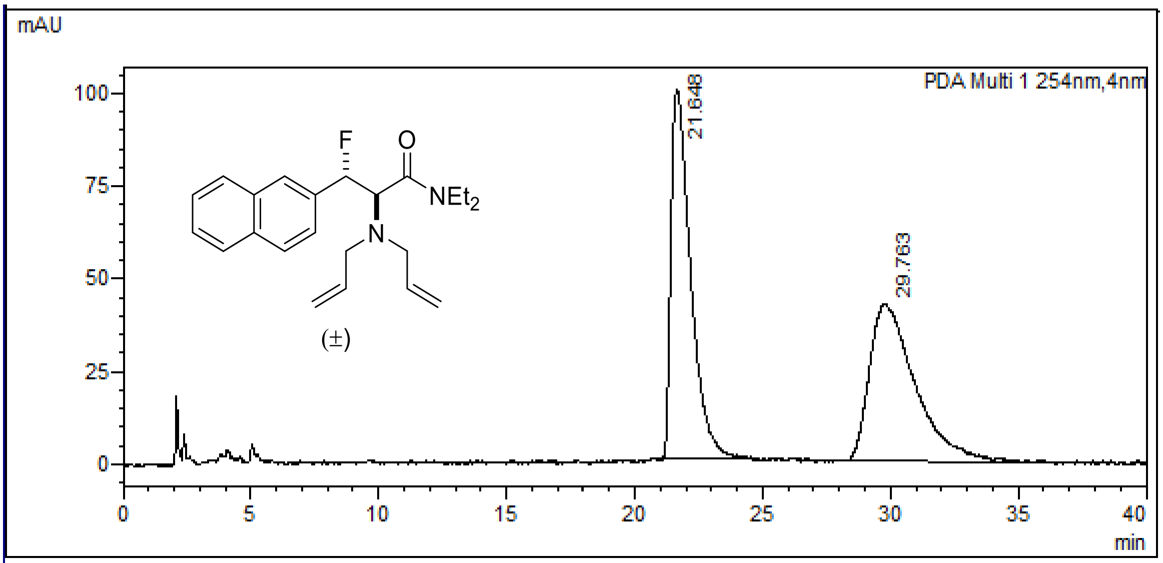

<Peak Table>

PDA Ch1 254nm

Peak#	Ret. Time	Area%
1	21.648	49.974
2	29.763	50.026

X-Ray Analysis

Low temperature single crystal X-ray diffraction data were collected using a Rigaku Oxford Diffraction SuperNovae diffractometer for **2q** and **3q** and data were collected using a XtaLAB Synergy R DW system mounted with a HyPix-Arc 150 detector for **3c**. Raw frame data were reduced using CrysAlisPro and the structures were solved using 'Superflip'[39] before refinement with CRYSTALS[40] as per the SI (CIF).

Table S9:. Crystal data and structure refinement for **2q**.

CCDC code	2232281	
Temperature	150 K	
Wavelength	1.54184 Å	
Crystal system / Space group	Monoclinic	P 2_1/c
Unit cell dimensions	a = 17.11680(10) Å	α= 90°.
	b = 5.71200(10) Å	β= 102.0315(8)°.
	c = 18.4819(2) Å	γ = 90°.
Volume	1767.30(4) Å^3	
Crystal size	0.26 x 0.20 x 0.10 mm^3	
Reflections collected	21187	
Independent reflections	3671 [R(int) = 0.022]	
Completeness to theta = 74.580°	99.7 %	
Absorption correction	Semi-empirical from equivalents	
Max. and min. transmission	0.93 and 0.88	
Refinement method	Full-matrix least-squares on F^2	
Data / restraints / parameters	3671 / 0 / 227	
Goodness-of-fit on F^2	0.9979	
Final R indices [I>2sigma(I)]	R1 = 0.0311, wR2 = 0.0845	
R indices (all data)	R1 = 0.0334, wR2 = 0.0873	
Extinction coefficient	29(5)	

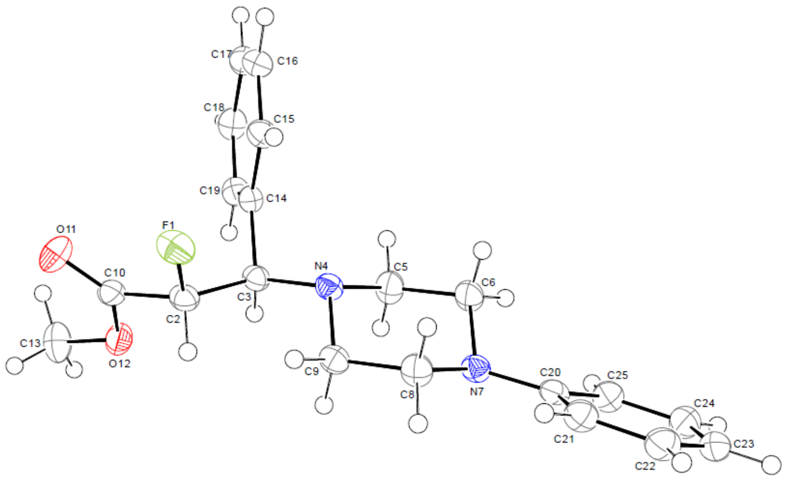

Table S10:. Crystal data and structure refinement for **3q**.

CCDC code	2232282	
Temperature	150 K	
Wavelength	1.54184 Å	
Crystal system / Space group	Monoclinic	P 2_1/n
Unit cell dimensions	a = 16.0593(3) Å	α= 90°.
	b = 5.96860(10) Å	β= 93.9460(15)°.
	c = 18.4106(3) Å	γ = 90°.
Volume	1760.50(5) $Å^3$	
Reflections collected	35417	
Independent reflections	3679 [R(int) = 0.035]	
Completeness to theta = 74.830°	99.8 %	
Absorption correction	Semi-empirical from equivalents	
Max. and min. transmission	0.99 and 0.86	
Refinement method	Full-matrix least-squares on F^2	
Data / restraints / parameters	3679 / 22 / 248	
Goodness-of-fit on F^2	1.0188	
Final R indices [I>2sigma(I)]	R1 = 0.0613, wR2 = 0.1292	
R indices (all data)	R1 = 0.0662, wR2 = 0.1319	

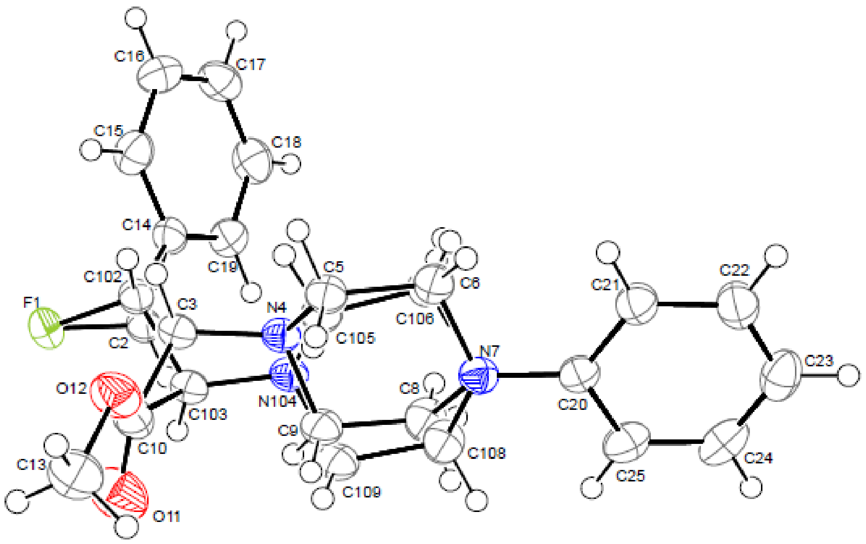

Any disorder has been off-set by 100.

***Table S11*:**. Crystal data and structure refinement for **3c**.

CCDC code	2232283	
Temperature	100 K	
Wavelength	1.54184 Å	
Crystal system / Space group	Hexagonal	P 6_1
Unit cell dimensions	a = 9.69470(10) Å	α= 90°.
	b = 9.69470(10) Å	β= 90°.
	c = 68.3133(11) Å	γ = 120°.
Volume	5560.38(14) $Å^3$	
Crystal size	0.12 x 0.07 x 0.05 mm^3	
Reflections collected	82821	
Independent reflections	7549 [R(int) = 0.075]	
Completeness to theta = 74.917°	99.7 %	
Absorption correction	Semi-empirical from equivalents	
Max. and min. transmission	0.97 and 0.80	
Refinement method	Full-matrix least-squares on F^2	
Data / restraints / parameters	7549 / 1 / 417	
Goodness-of-fit on F^2	1.0090	
Final R indices [I>2sigma(I)]	R1 = 0.0468, wR2 = 0.0851	
R indices (all data)	R1 = 0.0582, wR2 = 0.0896	
Absolute structure parameter	0.04(6)	
Extinction coefficient	98(9)	

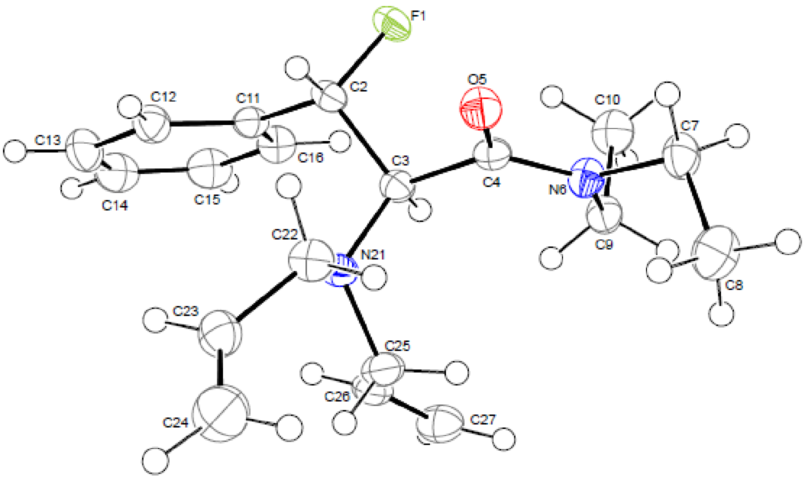

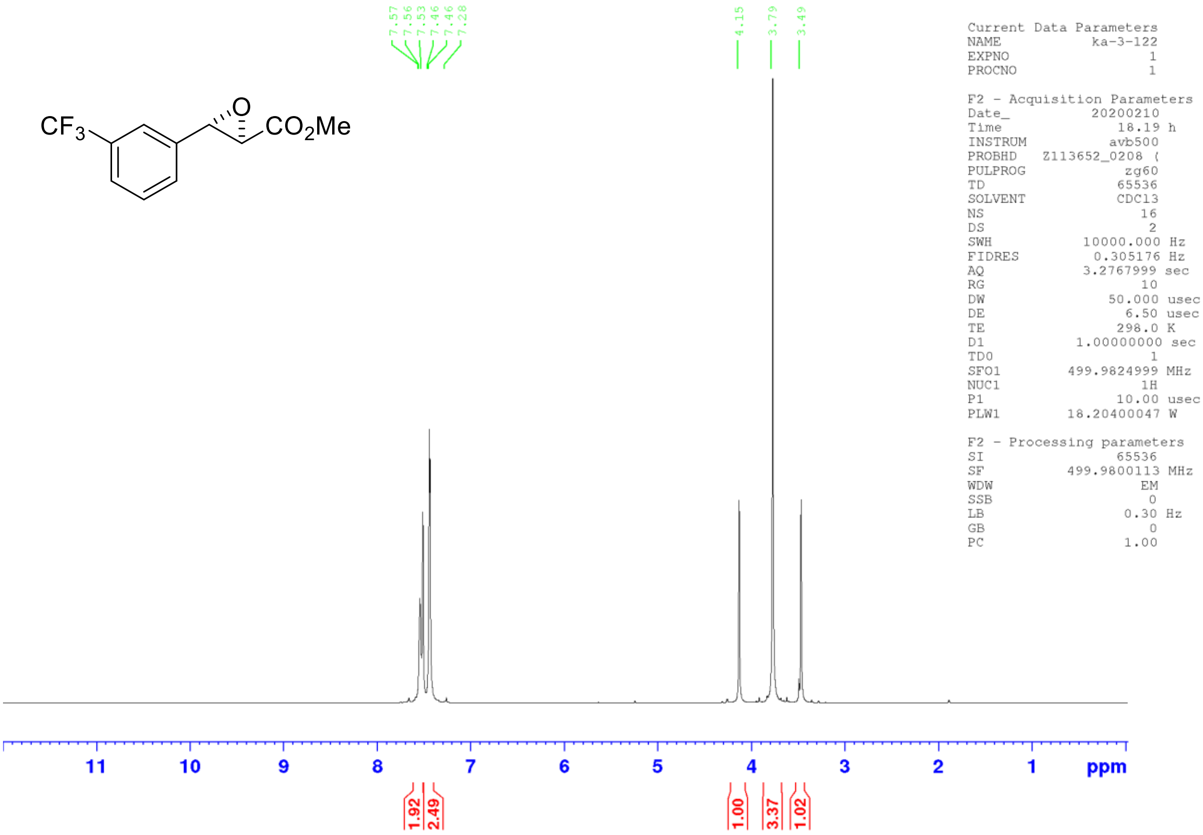

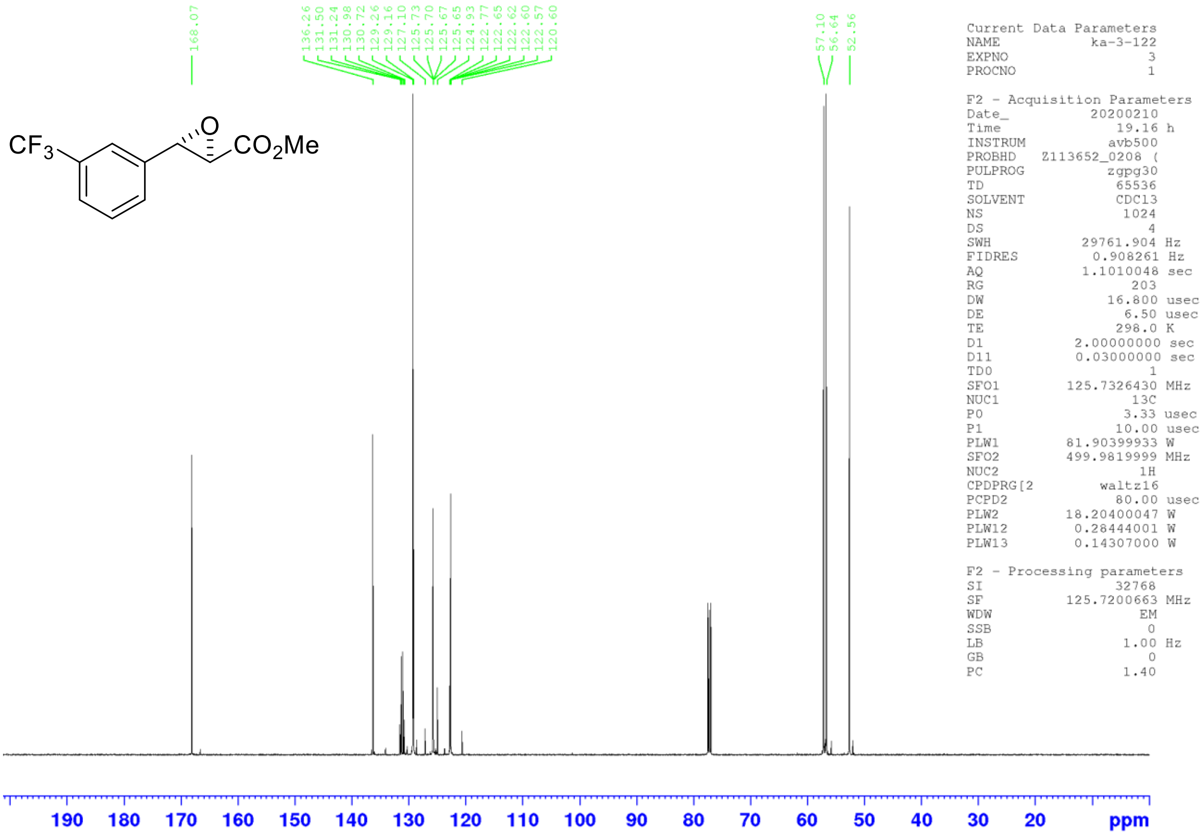

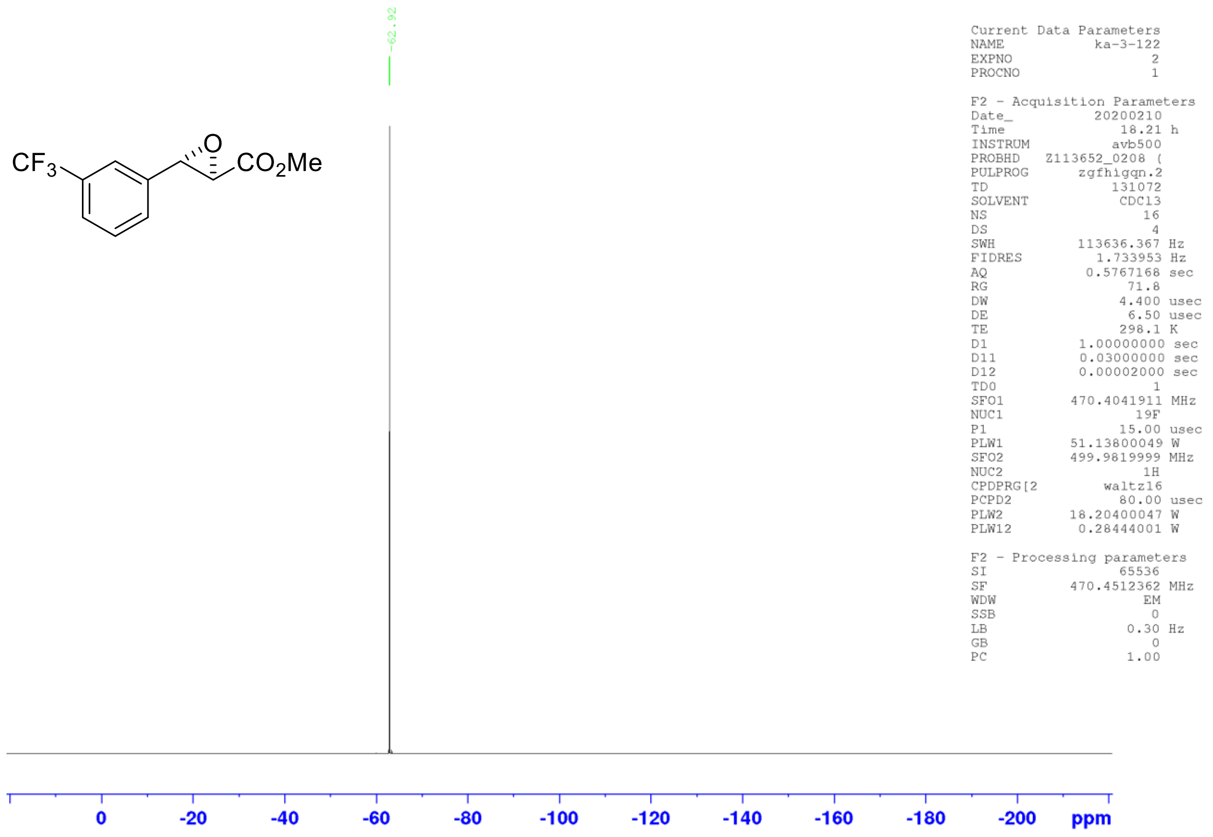

CF3
O
CO2Me
-62.92
Current Data Parameters
NAME ka-3-122
EXPNO 2
PROCNO 1
F2 - Acquisition Parameters
Date_ 20200210
Time 18.21 h
INSTRUM avb500
PROBHD Z113652_0208 (
PULPROG zgfhigqn.2
TD 131072
SOLVENT CDCl3
NS 16
DS 4
SWH 113636.367 Hz
FIDRES 1.733953 Hz
AQ 0.5767168 sec
RG 71.8
DW 4.400 usec
DE 6.50 usec
TE 298.1 K
D1 1.00000000 sec
D11 0.03000000 sec
D12 0.00002000 sec
TD0 1
SFO1 470.4041911 MHz
NUC1 19F
P1 15.00 usec
PLW1 51.13800049 W
SFO2 499.9819999 MHz
NUC2 1H
CPDPRG[2 waltz16
PCPD2 80.00 usec
PLW2 18.20400047 W
PLW12 0.28444001 W
F2 - Processing parameters
SI 65536
SF 470.4512362 MHz
WDW EM
SSB 0
LB 0.30 Hz
GB 0
PC 1.00
0 -20 -40 -60 -80 -100 -120 -140 -160 -180 -200 ppm

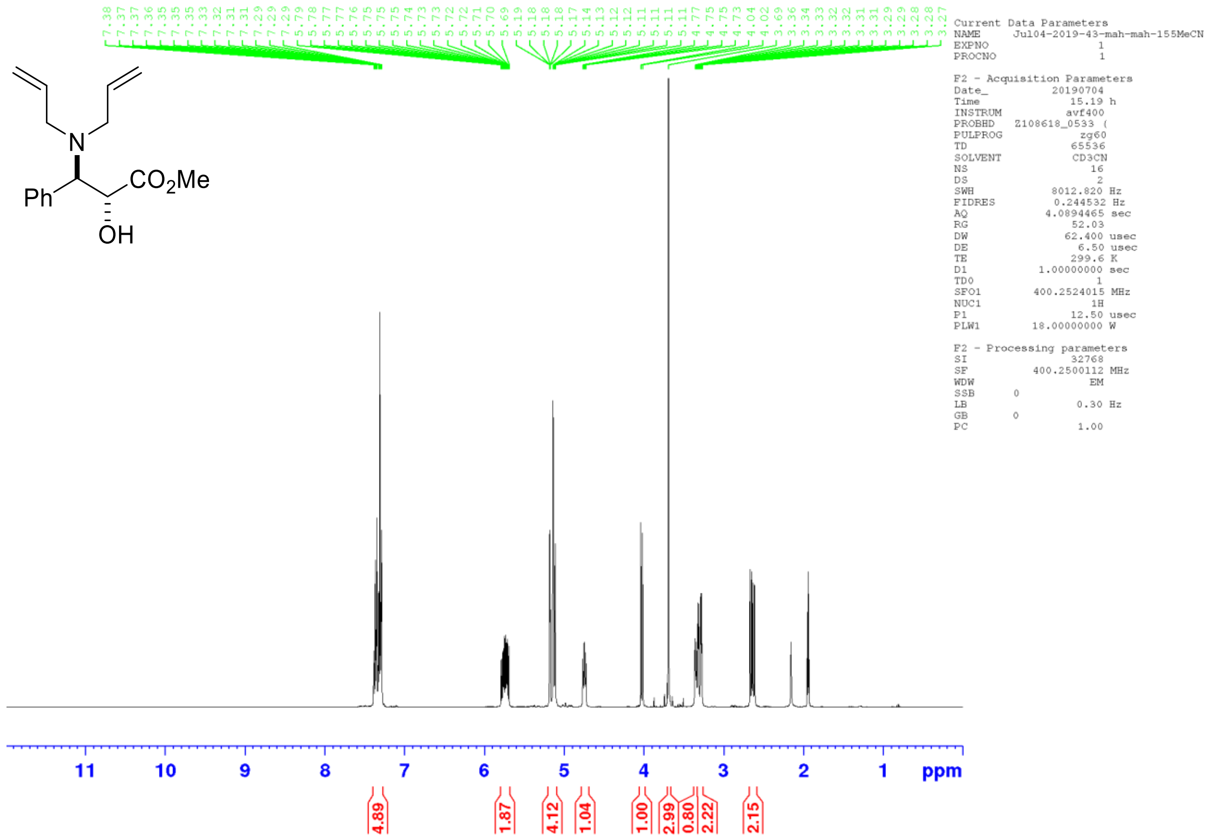

N
Ph
CO2Me
OH
7.38 7.37 7.36 7.35 7.35 7.33 7.32 7.31 7.31 7.29 7.29 7.29 5.79 5.78 5.77 5.76 5.75 5.75 5.74 5.73 5.73 5.72 5.72 5.71 5.70 5.69 5.19 5.18 5.18 5.17 5.14 5.13 5.12 5.11 5.11 5.11 5.11 4.77 4.75 4.75 4.73 4.04 4.02 3.69 3.36 3.34 3.33 3.32 3.31 3.29 3.29 3.28 3.27
Current Data Parameters
NAME Jul04-2019-43-mah-mah-155MeCN
EXPNO 1
PROCNO 1
F2 - Acquisition Parameters
Date_ 20190704
Time 15.19 h
INSTRUM avf400
PROBHD Z108618_0533 (
PULPROG zg60
TD 65536
SOLVENT CD3CN
NS 16
DS 2
SWH 8012.820 Hz
FIDRES 0.244532 Hz
AQ 4.0894465 sec
RG 52.03
DW 62.400 usec
DE 6.50 usec
TE 299.6 K
D1 1.00000000 sec
TD0 1
SFO1 400.2524015 MHz
NUC1 1H
P1 12.50 usec
PLW1 18.00000000 W
F2 - Processing parameters
SI 32768
SF 400.2500112 MHz
WDW EM
SSB 0
LB 0.30 Hz
GB 0
PC 1.00
11 10 9 8 7 6 5 4 3 2 1 ppm
4.89 1.87 4.12 1.04 1.00 2.99 0.80 2.22 2.15

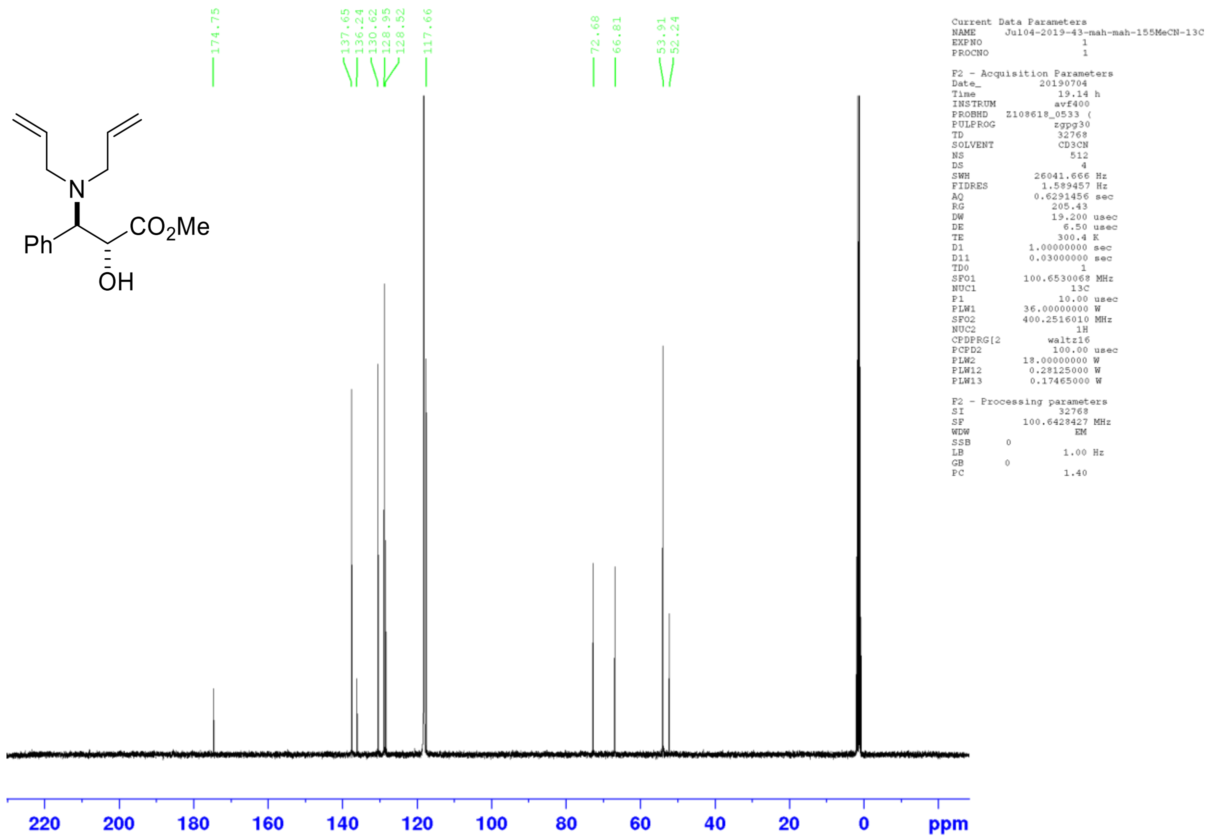

174.75
137.65
136.24
130.62
128.95
128.52
117.66
72.68
66.81
53.91
52.24
Ph
N
CO_2Me
OH
Current Data Parameters
NAME Jul04-2019-43-mah-mah-155MeCN-13C
EXPNO 1
PROCNO 1
F2 - Acquisition Parameters
Date_ 20190704
Time 19.14 h
INSTRUM avf400
PROBHD Z108618_0533 (
PULPROG zgpg30
TD 32768
SOLVENT CD3CN
NS 512
DS 4
SWH 26041.666 Hz
FIDRES 1.589457 Hz
AQ 0.6291456 sec
RG 205.43
DW 19.200 usec
DE 6.50 usec
TE 300.4 K
D1 1.00000000 sec
D11 0.03000000 sec
TD0 1
SFO1 100.6530068 MHz
NUC1 13C
P1 10.00 usec
PLW1 36.00000000 W
SFO2 400.2516010 MHz
NUC2 1H
CPDPRG[2 waltz16
PCPD2 100.00 usec
PLW2 18.00000000 W
PLW12 0.28125000 W
PLW13 0.17465000 W
F2 - Processing parameters
SI 32768
SF 100.6428427 MHz
WDW EM
SSB 0
LB 1.00 Hz
GB 0
PC 1.40
220
200
180
160
140
120
100
80
60
40
20
0
ppm

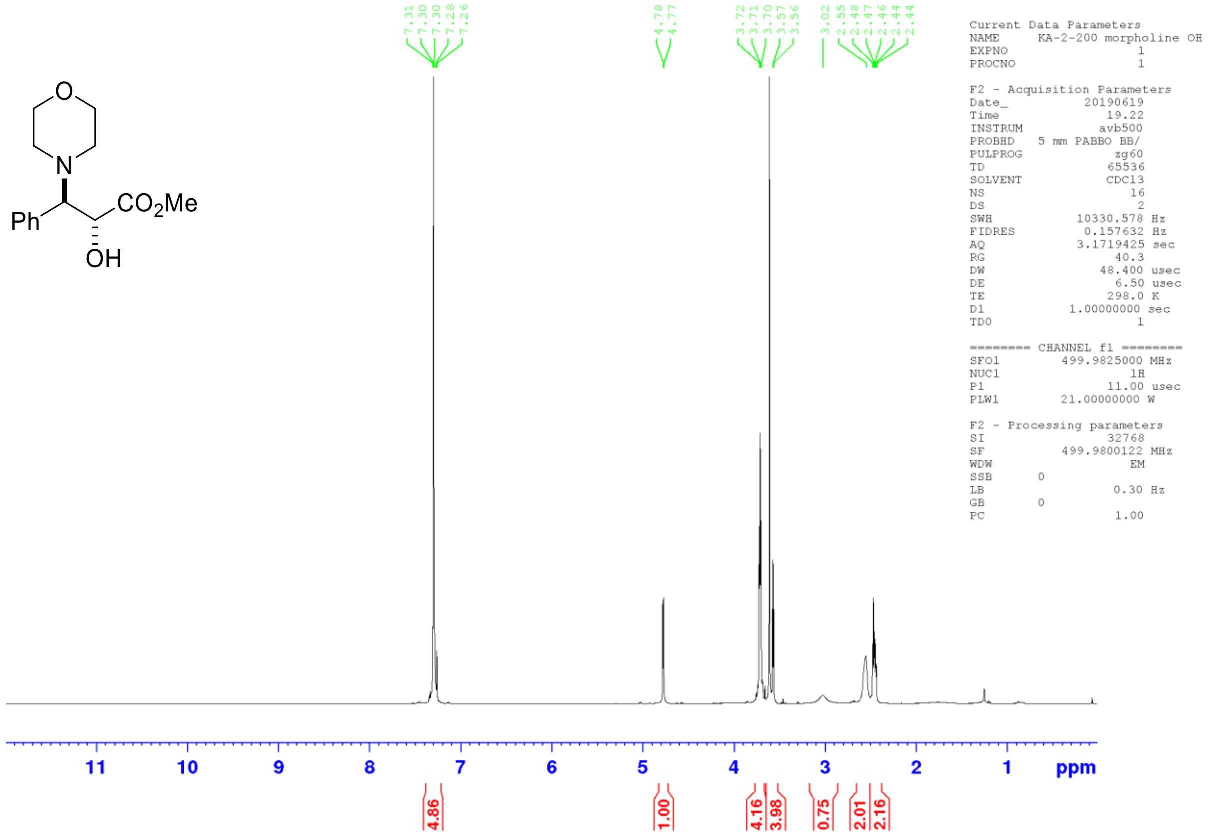

7.31
7.30
7.30
7.28
7.26
4.78
4.77
3.72
3.71
3.70
3.57
3.56
3.02
2.55
2.48
2.47
2.46
2.44
2.44
O
N
Ph
CO_2Me
OH
Current Data Parameters
NAME KA-2-200 morpholine OH
EXPNO 1
PROCNO 1
F2 - Acquisition Parameters
Date_ 20190619
Time 19.22
INSTRUM avb500
PROBHD 5 mm PABBO BB/
PULPROG zg60
TD 65536
SOLVENT CDCl3
NS 16
DS 2
SWH 10330.578 Hz
FIDRES 0.157632 Hz
AQ 3.1719425 sec
RG 40.3
DW 48.400 usec
DE 6.50 usec
TE 298.0 K
D1 1.00000000 sec
TD0 1
======== CHANNEL f1 ========
SFO1 499.9825000 MHz
NUC1 1H
P1 11.00 usec
PLW1 21.00000000 W
F2 - Processing parameters
SI 32768
SF 499.9800122 MHz
WDW EM
SSB 0
LB 0.30 Hz
GB 0
PC 1.00
11
10
9
8
7
6
5
4
3
2
1
ppm
4.86
1.00
4.16
3.98
0.75
2.01
2.16

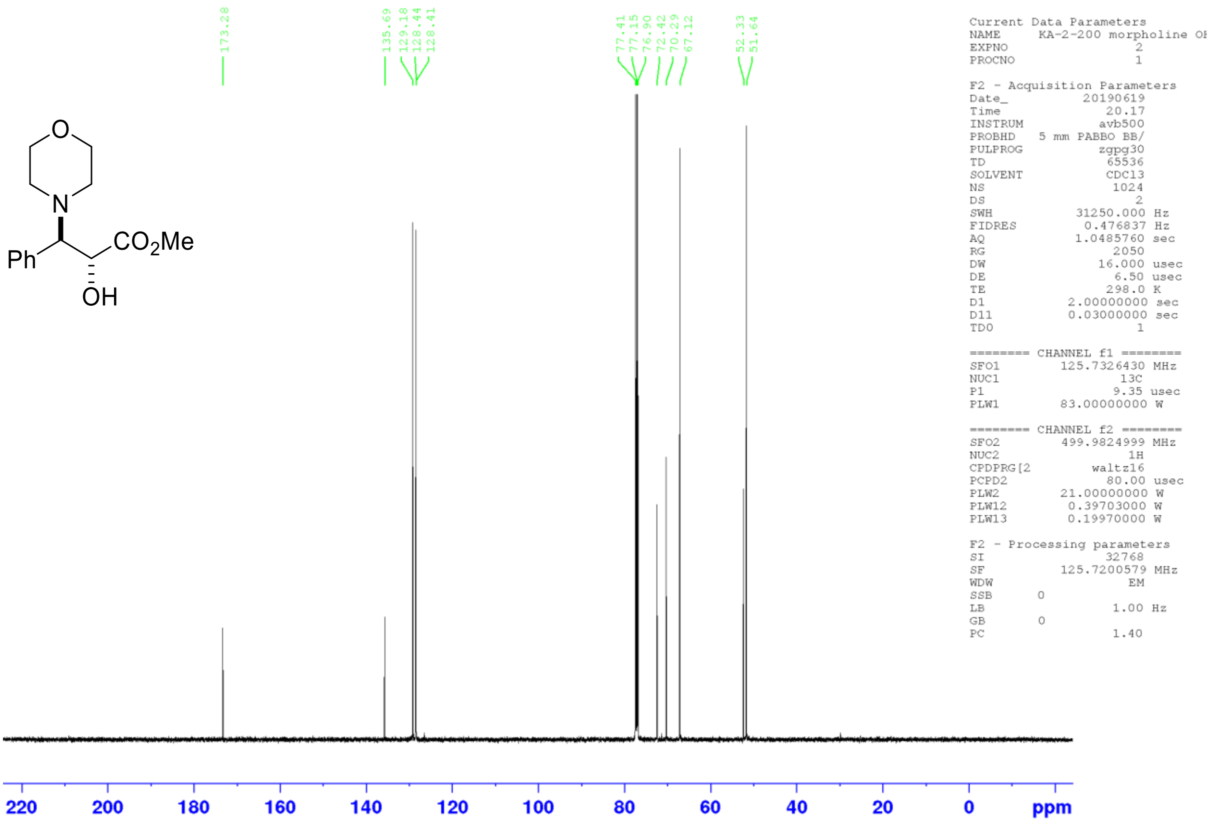

Current Data Parameters
NAME KA-2-200 morpholine O
EXPNO 2
PROCNO 1

F2 - Acquisition Parameters
Date_ 20190619
Time 20.17
INSTRUM avb500
PROBHD 5 mm PABBO BB/
PULPROG zgpg30
TD 65536
SOLVENT CDCl3
NS 1024
DS 2
SWH 31250.000 Hz
FIDRES 0.476837 Hz
AQ 1.0485760 sec
RG 2050
DW 16.000 usec
DE 6.50 usec
TE 298.0 K
D1 2.00000000 sec
D11 0.03000000 sec
TD0 1

======== CHANNEL f1 ========
SFO1 125.7326430 MHz
NUC1 13C
P1 9.35 usec
PLW1 83.00000000 W

======== CHANNEL f2 ========
SFO2 499.9824999 MHz
NUC2 1H
CPDPRG[2 waltz16
PCPD2 80.00 usec
PLW2 21.00000000 W
PLW12 0.39703000 W
PLW13 0.19970000 W

F2 - Processing parameters
SI 32768
SF 125.7200579 MHz
WDW EM
SSB 0
LB 1.00 Hz
GB 0
PC 1.40

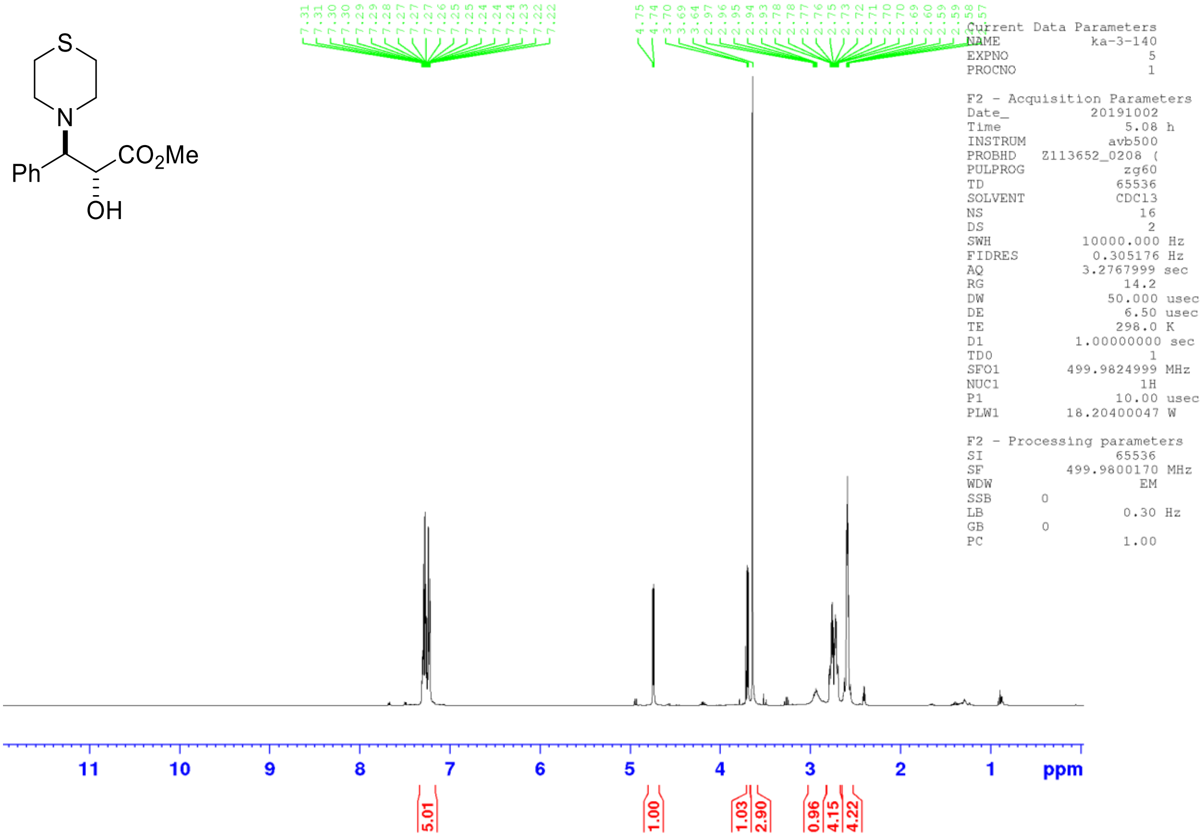

Current Data Parameters
NAME ka-3-140
EXPNO 5
PROCNO 1

F2 - Acquisition Parameters
Date_ 20191002
Time 5.08 h
INSTRUM avb500
PROBHD Z113652_0208 (
PULPROG zg60
TD 65536
SOLVENT CDCl3
NS 16
DS 2
SWH 10000.000 Hz
FIDRES 0.305176 Hz
AQ 3.2767999 sec
RG 14.2
DW 50.000 usec
DE 6.50 usec
TE 298.0 K
D1 1.00000000 sec
TD0 1
SFO1 499.9824999 MHz
NUC1 1H
P1 10.00 usec
PLW1 18.20400047 W

F2 - Processing parameters
SI 65536
SF 499.9800170 MHz
WDW EM
SSB 0
LB 0.30 Hz
GB 0
PC 1.00

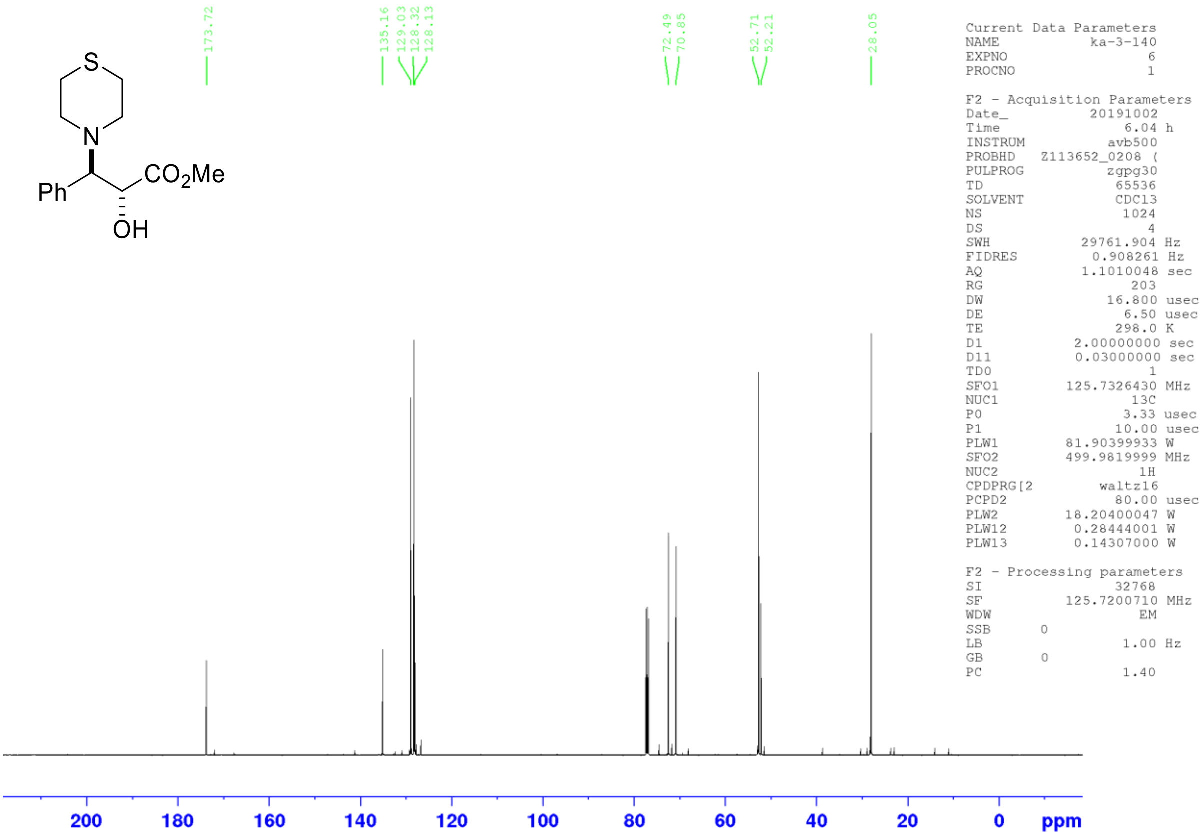
S
N
Ph
CO2Me
OH
173.72
135.16
129.03
128.32
128.13
72.49
70.85
52.71
52.21
28.05
Current Data Parameters
NAME ka-3-140
EXPNO 6
PROCNO 1
F2 - Acquisition Parameters
Date_ 20191002
Time 6.04 h
INSTRUM avb500
PROBHD Z113652_0208 (
PULPROG zgpg30
TD 65536
SOLVENT CDCl3
NS 1024
DS 4
SWH 29761.904 Hz
FIDRES 0.908261 Hz
AQ 1.1010048 sec
RG 203
DW 16.800 usec
DE 6.50 usec
TE 298.0 K
D1 2.00000000 sec
D11 0.03000000 sec
TD0 1
SFO1 125.7326430 MHz
NUC1 13C
P0 3.33 usec
P1 10.00 usec
PLW1 81.90399933 W
SFO2 499.9819999 MHz
NUC2 1H
CPDPRG[2 waltz16
PCPD2 80.00 usec
PLW2 18.20400047 W
PLW12 0.28444001 W
PLW13 0.14307000 W
F2 - Processing parameters
SI 32768
SF 125.7200710 MHz
WDW EM
SSB 0
LB 1.00 Hz
GB 0
PC 1.40
200
180
160
140
120
100
80
60
40
20
0
ppm

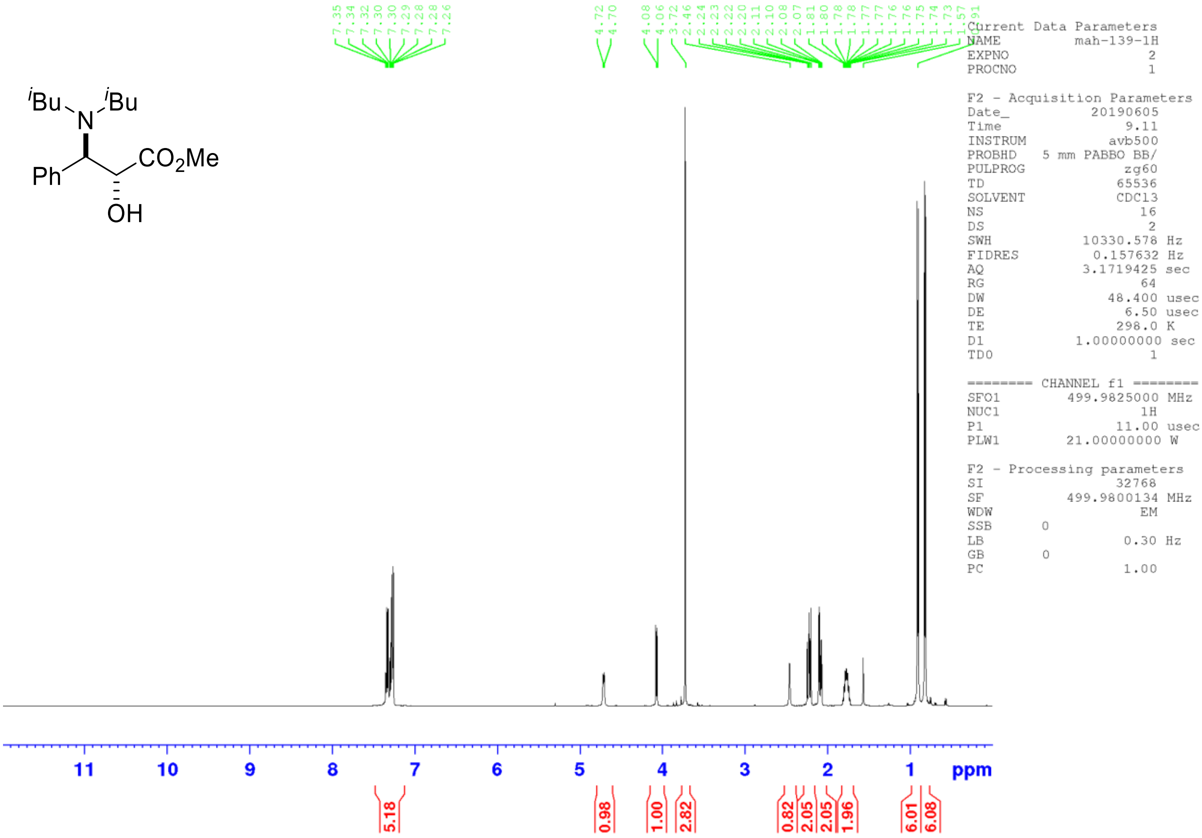
iBu
N
iBu
Ph
CO2Me
OH
7.35
7.34
7.32
7.30
7.29
7.28
7.28
7.26
4.72
4.70
4.08
4.06
3.72
2.46
2.24
2.23
2.22
2.20
2.11
2.10
2.08
2.07
1.81
1.80
1.78
1.77
1.77
1.76
1.75
1.74
1.73
1.57
0.91
Current Data Parameters
NAME mah-139-1H
EXPNO 2
PROCNO 1
F2 - Acquisition Parameters
Date_ 20190605
Time 9.11
INSTRUM avb500
PROBHD 5 mm PABBO BB/
PULPROG zg60
TD 65536
SOLVENT CDCl3
NS 16
DS 2
SWH 10330.578 Hz
FIDRES 0.157632 Hz
AQ 3.1719425 sec
RG 64
DW 48.400 usec
DE 6.50 usec
TE 298.0 K
D1 1.00000000 sec
TD0 1
======== CHANNEL f1 ========
SFO1 499.9825000 MHz
NUC1 1H
P1 11.00 usec
PLW1 21.00000000 W
F2 - Processing parameters
SI 32768
SF 499.9800134 MHz
WDW EM
SSB 0
LB 0.30 Hz
GB 0
PC 1.00
11
10
9
8
7
6
5
4
3
2
1
ppm
5.18
0.98
1.00
2.82
0.82
2.05
2.05
1.96
6.01
6.08

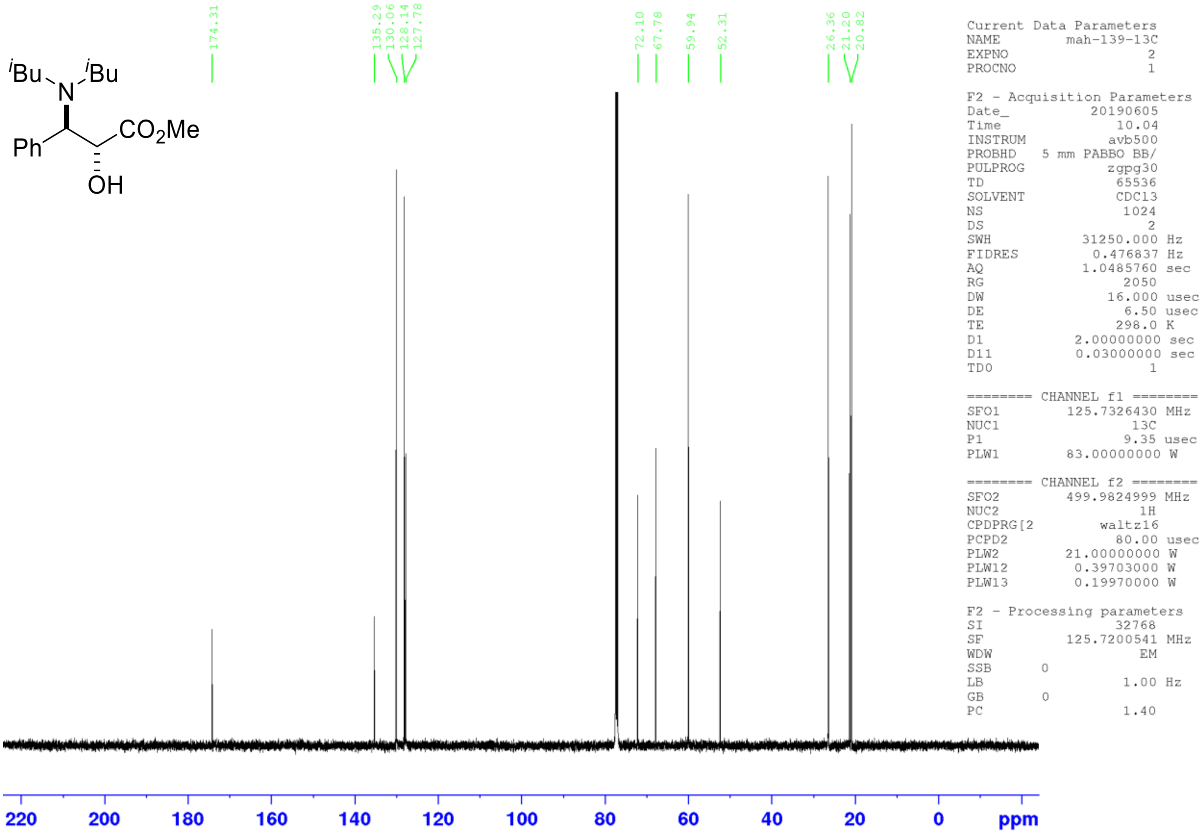


```
Current Data Parameters
NAME          mah-139-13C
EXPNO                   2
PROCNO                  1

F2 - Acquisition Parameters
Date_            20190605
Time                10.04
INSTRUM            avb500
PROBHD   5 mm PABBO BB/
PULPROG           zgpg30
TD                  65536
SOLVENT             CDCl3
NS                   1024
DS                      2
SWH             31250.000 Hz
FIDRES           0.476837 Hz
AQ              1.0485760 sec
RG                   2050
DW                 16.000 usec
DE                   6.50 usec
TE                  298.0 K
D1             2.00000000 sec
D11            0.03000000 sec
TD0                     1

======== CHANNEL f1 ========
SFO1          125.7326430 MHz
NUC1                  13C
P1                   9.35 usec
PLW1          83.00000000 W

======== CHANNEL f2 ========
SFO2          499.9824999 MHz
NUC2                   1H
CPDPRG[2           waltz16
PCPD2               80.00 usec
PLW2          21.00000000 W
PLW12          0.39703000 W
PLW13          0.19970000 W

F2 - Processing parameters
SI                  32768
SF            125.7200541 MHz
WDW                    EM
SSB      0
LB                   1.00 Hz
GB       0
PC                   1.40
```

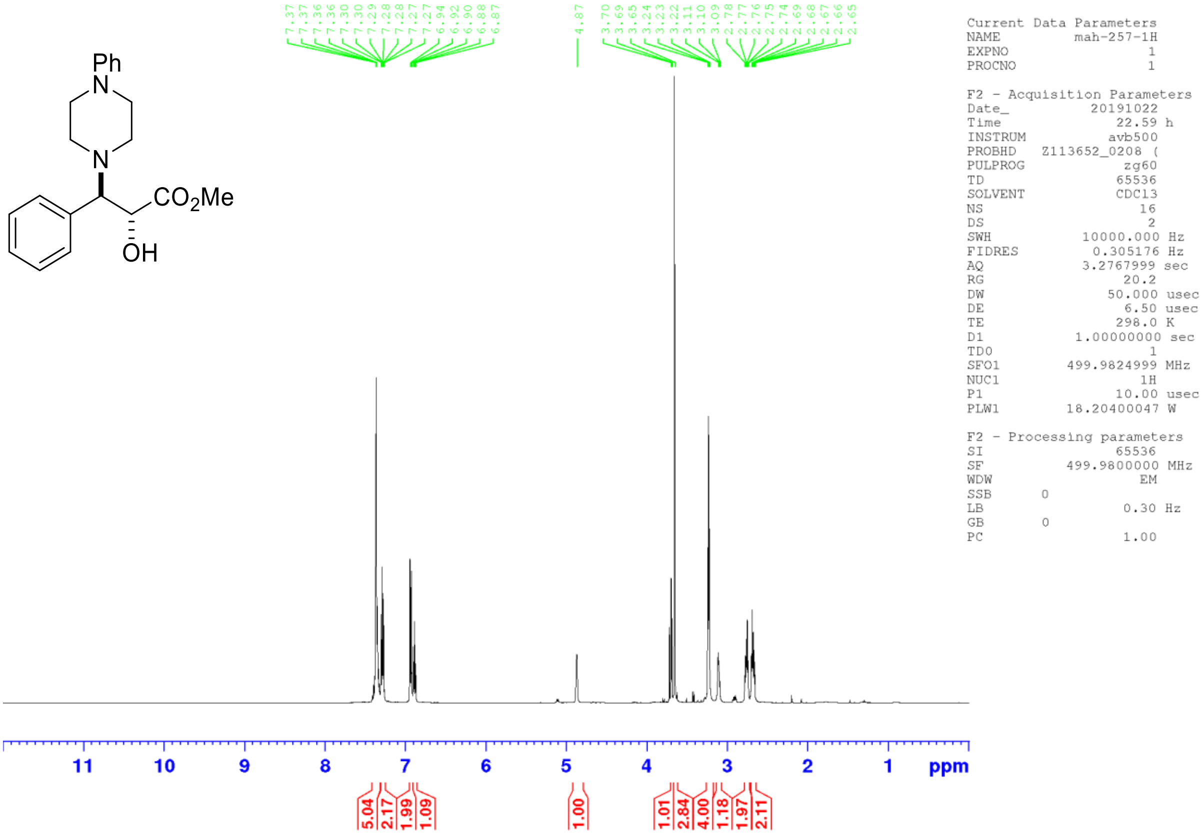


```
Current Data Parameters
NAME           mah-257-1H
EXPNO                   1
PROCNO                  1

F2 - Acquisition Parameters
Date_            20191022
Time                22.59 h
INSTRUM            avb500
PROBHD   Z113652_0208 (
PULPROG              zg60
TD                  65536
SOLVENT             CDCl3
NS                     16
DS                      2
SWH             10000.000 Hz
FIDRES           0.305176 Hz
AQ              3.2767999 sec
RG                   20.2
DW                 50.000 usec
DE                   6.50 usec
TE                  298.0 K
D1             1.00000000 sec
TD0                     1
SFO1          499.9824999 MHz
NUC1                   1H
P1                  10.00 usec
PLW1          18.20400047 W

F2 - Processing parameters
SI                  65536
SF            499.9800000 MHz
WDW                    EM
SSB      0
LB                   0.30 Hz
GB       0
PC                   1.00
```

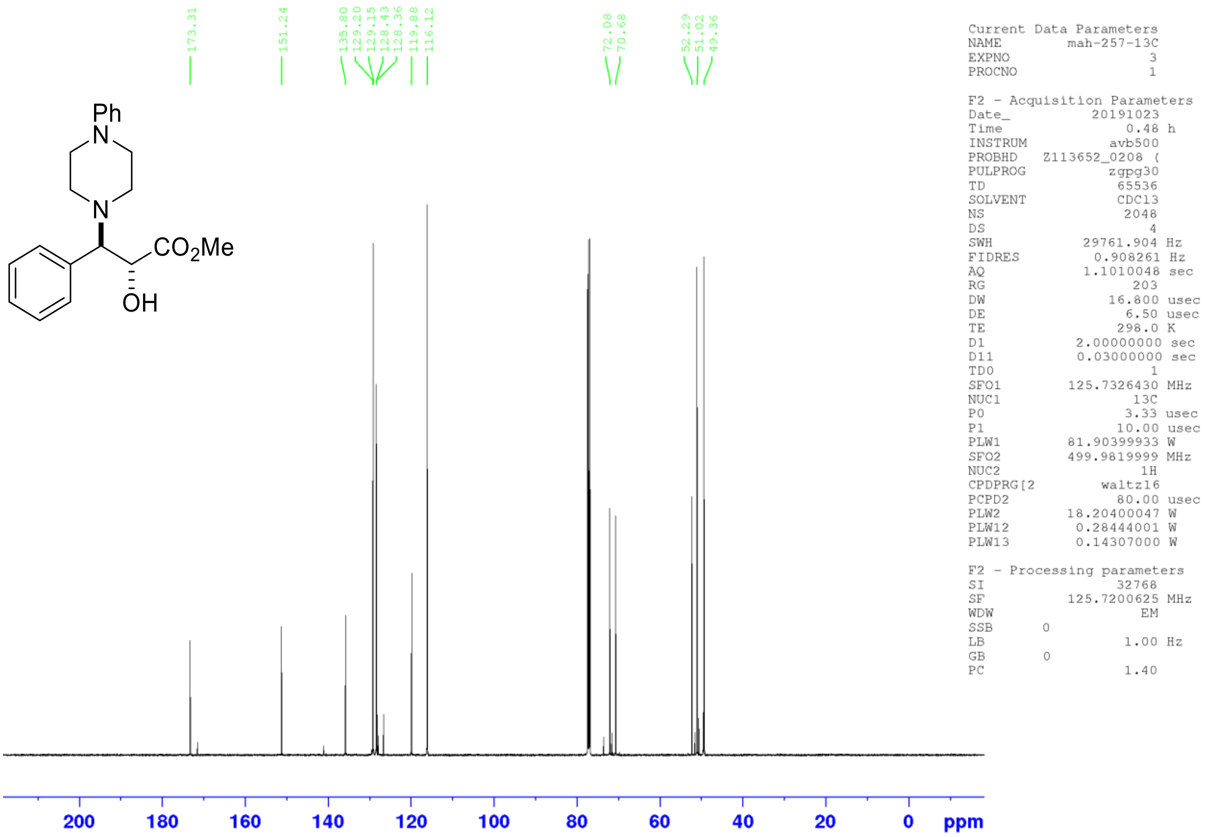
Ph
N
N
CO_2Me
OH
173.31
151.24
135.80
129.20
129.15
128.43
128.36
119.88
116.12
72.08
70.68
52.29
51.02
49.36
200 180 160 140 120 100 80 60 40 20 0 ppm
Current Data Parameters
NAME mah-257-13C
EXPNO 3
PROCNO 1
F2 - Acquisition Parameters
Date_ 20191023
Time 0.48 h
INSTRUM avb500
PROBHD Z113652_0208 (
PULPROG zgpg30
TD 65536
SOLVENT CDCl3
NS 2048
DS 4
SWH 29761.904 Hz
FIDRES 0.908261 Hz
AQ 1.1010048 sec
RG 203
DW 16.800 usec
DE 6.50 usec
TE 298.0 K
D1 2.00000000 sec
D11 0.03000000 sec
TD0 1
SFO1 125.7326430 MHz
NUC1 13C
P0 3.33 usec
P1 10.00 usec
PLW1 81.90399933 W
SFO2 499.9819999 MHz
NUC2 1H
CPDPRG[2 waltz16
PCPD2 80.00 usec
PLW2 18.20400047 W
PLW12 0.28444001 W
PLW13 0.14307000 W
F2 - Processing parameters
SI 32768
SF 125.7200625 MHz
WDW EM
SSB 0
LB 1.00 Hz
GB 0
PC 1.40

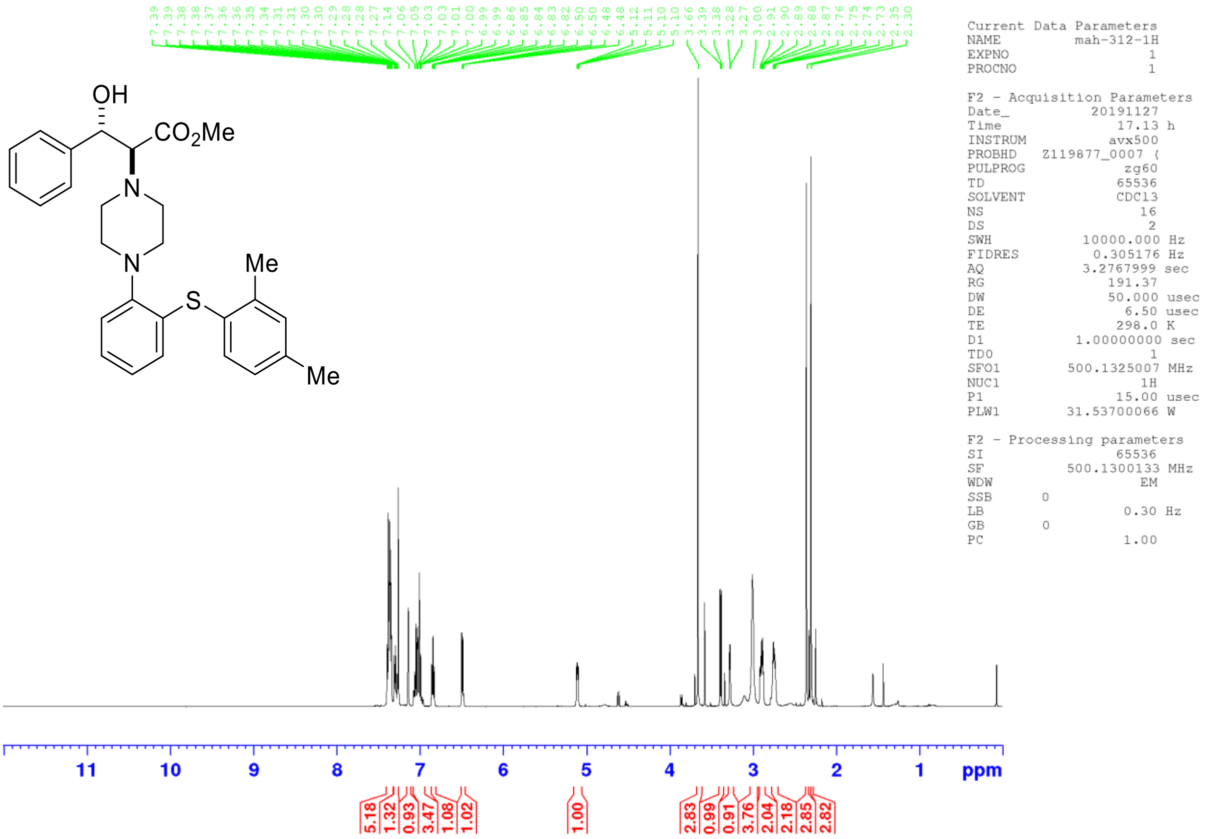
OH
CO_2Me
N
N
S
Me
Me
7.39 7.39 7.38 7.38 7.37 7.36 7.36 7.35 7.34 7.31 7.31 7.30 7.30 7.29 7.28 7.28 7.27 7.14 7.06 7.05 7.03 7.03 7.01 7.00 6.99 6.99 6.86 6.85 6.84 6.83 6.82 6.50 6.50 6.48 6.48 5.12 5.11 5.10 5.10 3.66 3.39 3.38 3.28 3.27 3.00 2.91 2.90 2.89 2.88 2.87 2.76 2.75 2.74 2.73 2.35 2.30
11 10 9 8 7 6 5 4 3 2 1 ppm
5.18 1.32 0.93 3.47 1.08 1.02 1.00 2.83 0.99 0.91 3.76 2.04 2.18 2.85 2.82
Current Data Parameters
NAME mah-312-1H
EXPNO 1
PROCNO 1
F2 - Acquisition Parameters
Date_ 20191127
Time 17.13 h
INSTRUM avx500
PROBHD Z119877_0007 (
PULPROG zg60
TD 65536
SOLVENT CDCl3
NS 16
DS 2
SWH 10000.000 Hz
FIDRES 0.305176 Hz
AQ 3.2767999 sec
RG 191.37
DW 50.000 usec
DE 6.50 usec
TE 298.0 K
D1 1.00000000 sec
TD0 1
SFO1 500.1325007 MHz
NUC1 1H
P1 15.00 usec
PLW1 31.53700066 W
F2 - Processing parameters
SI 65536
SF 500.1300133 MHz
WDW EM
SSB 0
LB 0.30 Hz
GB 0
PC 1.00

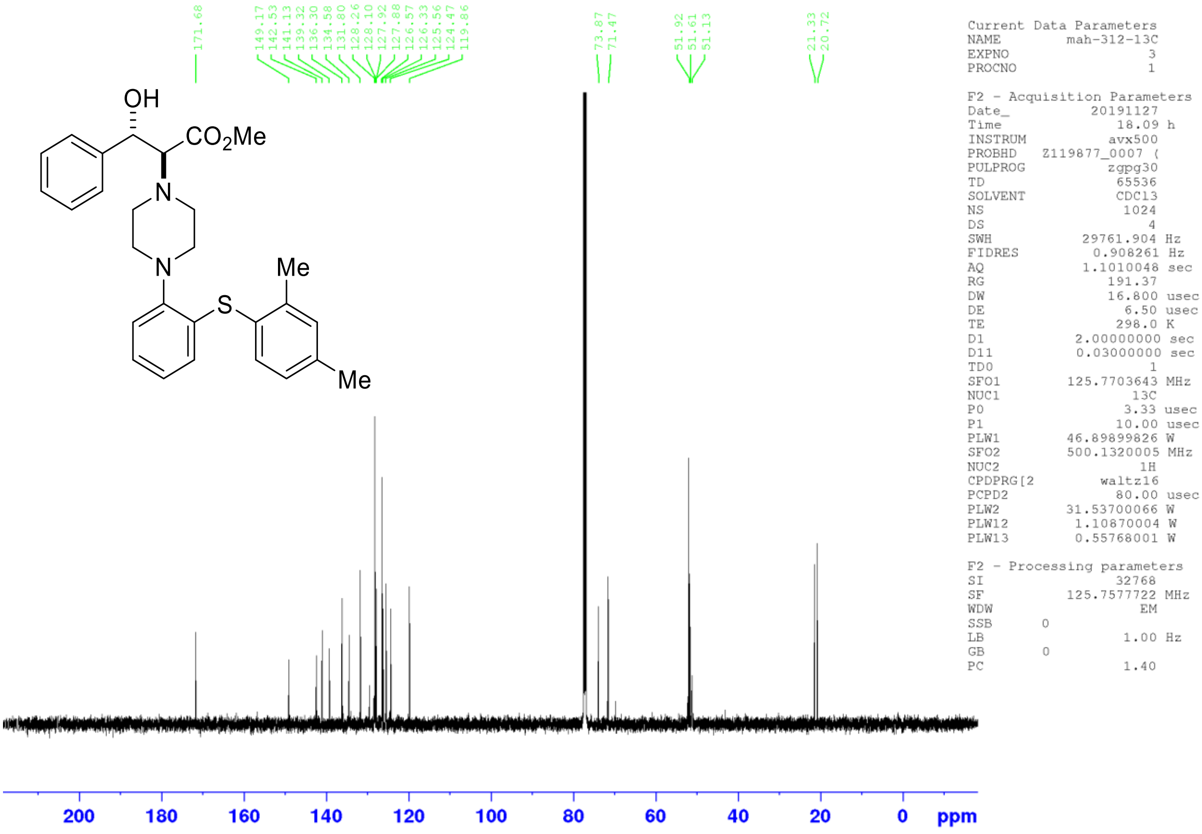


```
Current Data Parameters
NAME          mah-312-13C
EXPNO                   3
PROCNO                  1

F2 - Acquisition Parameters
Date_            20191127
Time                18.09 h
INSTRUM            avx500
PROBHD   Z119877_0007 (
PULPROG           zgpg30
TD                 65536
SOLVENT            CDCl3
NS                  1024
DS                     4
SWH            29761.904 Hz
FIDRES          0.908261 Hz
AQ             1.1010048 sec
RG                191.37
DW                16.800 usec
DE                  6.50 usec
TE                 298.0 K
D1            2.00000000 sec
D11           0.03000000 sec
TD0                    1
SFO1         125.7703643 MHz
NUC1                 13C
P0                  3.33 usec
P1                 10.00 usec
PLW1         46.89899826 W
SFO2         500.1320005 MHz
NUC2                  1H
CPDPRG[2         waltz16
PCPD2              80.00 usec
PLW2         31.53700066 W
PLW12         1.10870004 W
PLW13         0.55768001 W

F2 - Processing parameters
SI                 32768
SF           125.7577722 MHz
WDW                   EM
SSB      0
LB                  1.00 Hz
GB       0
PC                  1.40
```

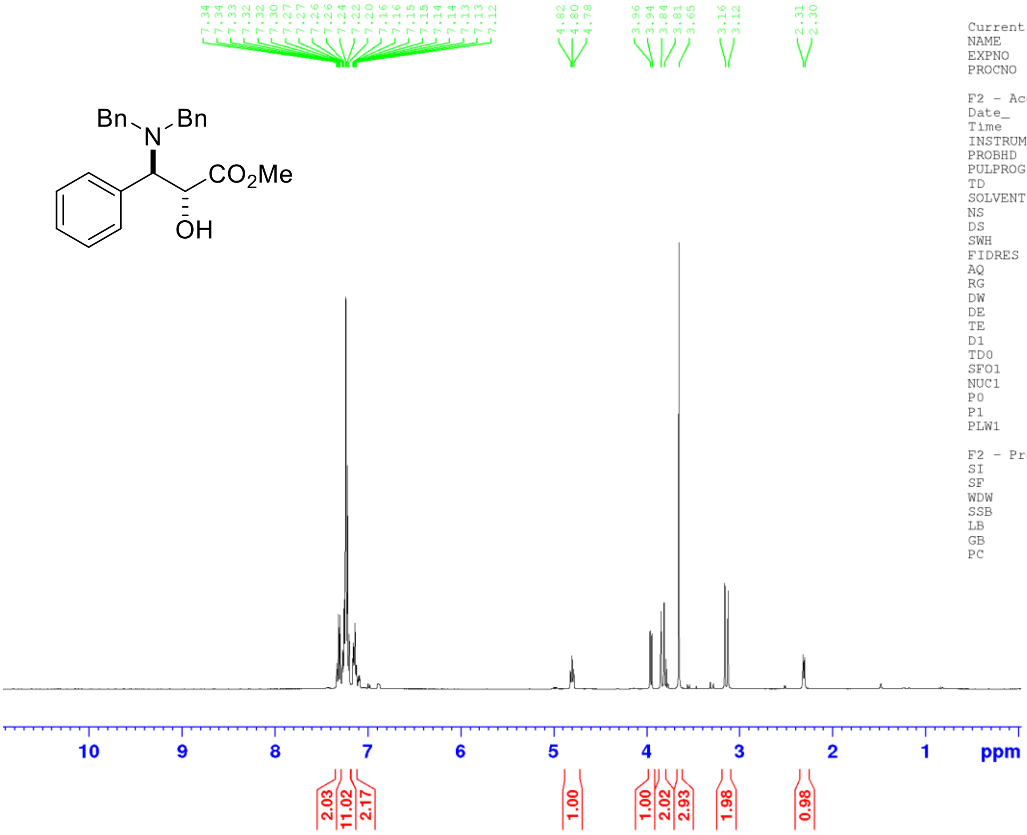


```
Current Data Parameters
NAME              ka-3-96
EXPNO                   1
PROCNO                  1

F2 - Acquisition Parameters
Date_            20191002
Time                18.39 h
INSTRUM           Avance
PROBHD   Z116098_0219 (
PULPROG             zg30
TD                 65536
SOLVENT            CDCl3
NS                    16
DS                     2
SWH             7812.500 Hz
FIDRES          0.238419 Hz
AQ             4.1943040 sec
RG               57.7984
DW                64.000 usec
DE                  6.50 usec
TE                 298.0 K
D1            1.00000000 sec
TD0                    1
SFO1         400.1320007 MHz
NUC1                  1H
P0                  3.33 usec
P1                 10.00 usec
PLW1         16.35899925 W

F2 - Processing parameters
SI                 65536
SF           400.1300578 MHz
WDW                   EM
SSB      0
LB                  0.30 Hz
GB       0
PC                  1.00
```

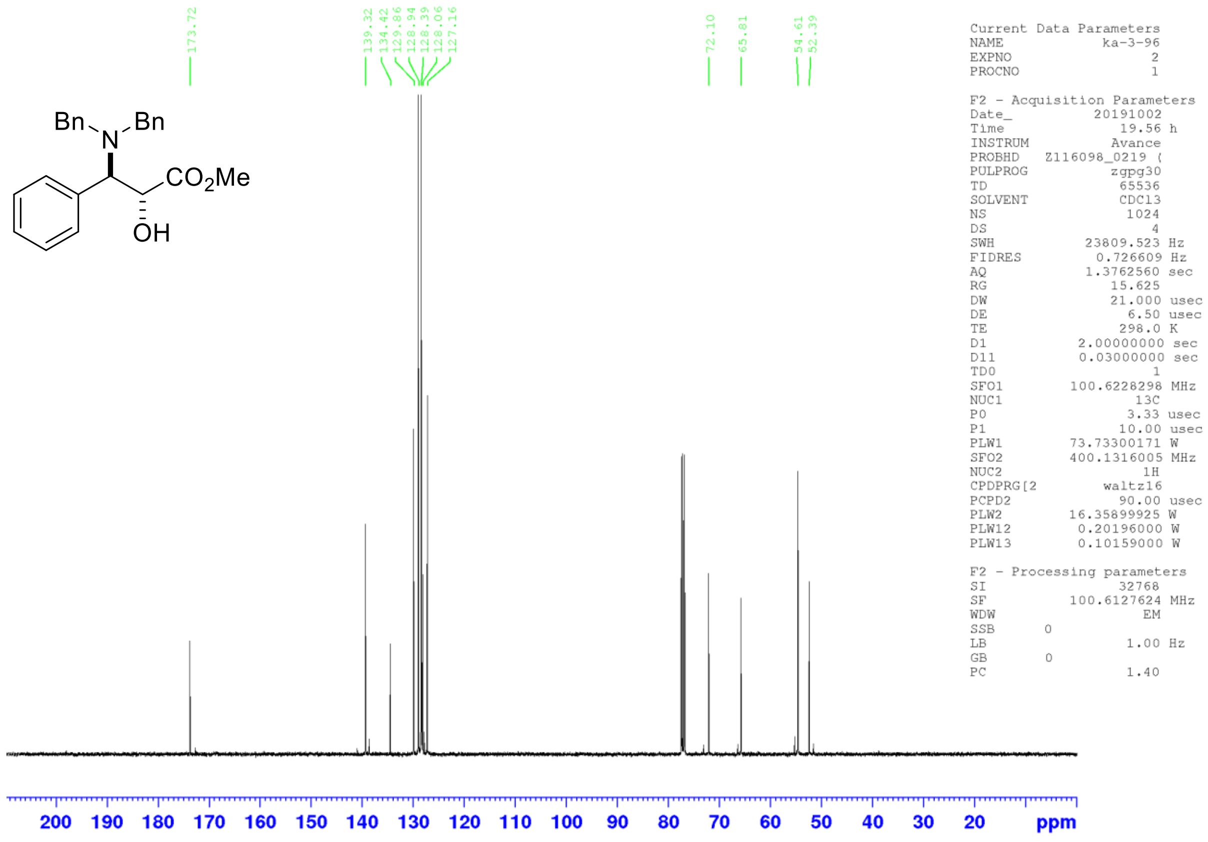

Bn
N
Bn
CO2Me
OH
173.72
139.32
134.42
129.86
128.94
128.39
128.06
127.16
72.10
65.81
54.61
52.39
Current Data Parameters
NAME ka-3-96
EXPNO 2
PROCNO 1
F2 - Acquisition Parameters
Date_ 20191002
Time 19.56 h
INSTRUM Avance
PROBHD Z116098_0219 (
PULPROG zgpg30
TD 65536
SOLVENT CDCl3
NS 1024
DS 4
SWH 23809.523 Hz
FIDRES 0.726609 Hz
AQ 1.3762560 sec
RG 15.625
DW 21.000 usec
DE 6.50 usec
TE 298.0 K
D1 2.00000000 sec
D11 0.03000000 sec
TD0 1
SFO1 100.6228298 MHz
NUC1 13C
P0 3.33 usec
P1 10.00 usec
PLW1 73.73300171 W
SFO2 400.1316005 MHz
NUC2 1H
CPDPRG[2 waltz16
PCPD2 90.00 usec
PLW2 16.35899925 W
PLW12 0.20196000 W
PLW13 0.10159000 W
F2 - Processing parameters
SI 32768
SF 100.6127624 MHz
WDW EM
SSB 0
LB 1.00 Hz
GB 0
PC 1.40
200 190 180 170 160 150 140 130 120 110 100 90 80 70 60 50 40 30 20 ppm

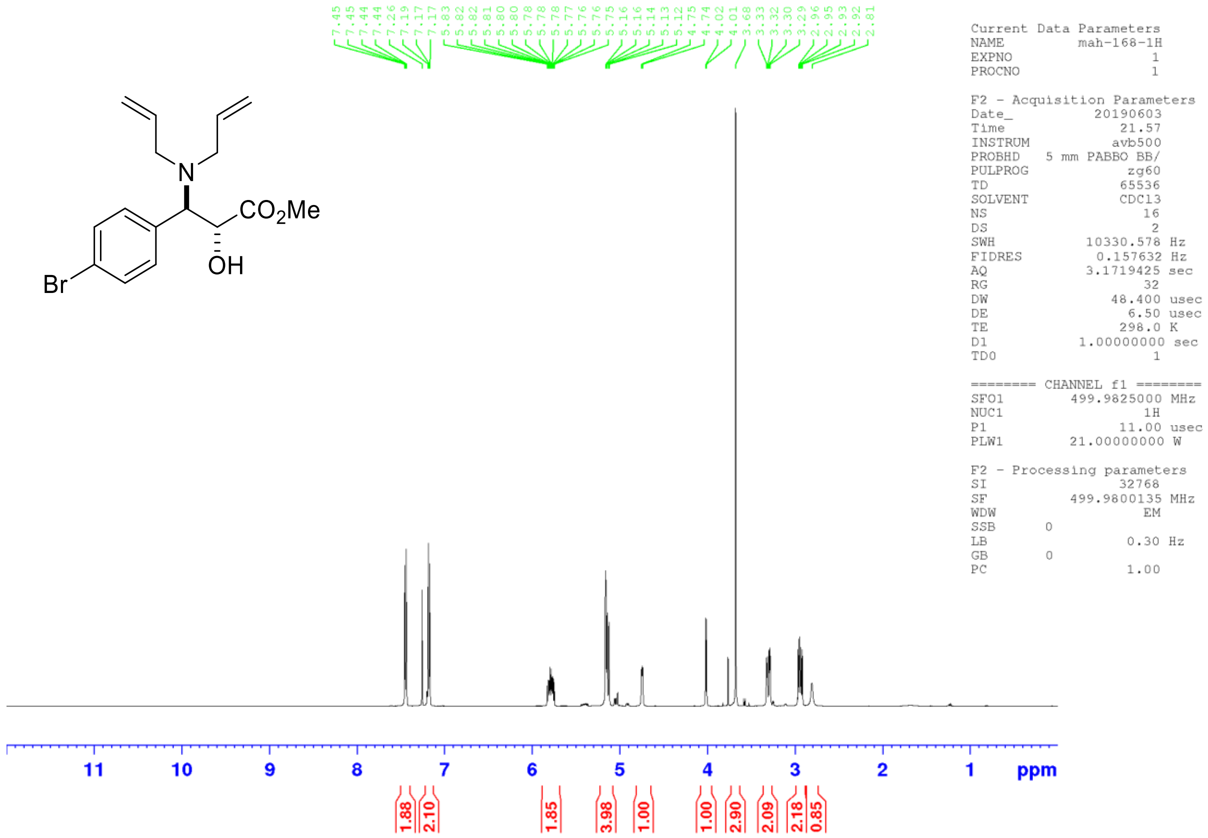

N
CO2Me
OH
Br
7.45
7.45
7.44
7.44
7.26
7.19
7.17
7.17
5.83
5.82
5.81
5.81
5.80
5.80
5.78
5.78
5.77
5.76
5.76
5.75
5.16
5.16
5.14
5.13
5.12
4.75
4.74
4.02
4.01
3.68
3.33
3.32
3.30
3.29
2.96
2.95
2.93
2.92
2.81
Current Data Parameters
NAME mah-168-1H
EXPNO 1
PROCNO 1
F2 - Acquisition Parameters
Date_ 20190603
Time 21.57
INSTRUM avb500
PROBHD 5 mm PABBO BB/
PULPROG zg60
TD 65536
SOLVENT CDCl3
NS 16
DS 2
SWH 10330.578 Hz
FIDRES 0.157632 Hz
AQ 3.1719425 sec
RG 32
DW 48.400 usec
DE 6.50 usec
TE 298.0 K
D1 1.00000000 sec
TD0 1
======== CHANNEL f1 ========
SFO1 499.9825000 MHz
NUC1 1H
P1 11.00 usec
PLW1 21.00000000 W
F2 - Processing parameters
SI 32768
SF 499.9800135 MHz
WDW EM
SSB 0
LB 0.30 Hz
GB 0
PC 1.00
11 10 9 8 7 6 5 4 3 2 1 ppm
1.88
2.10
1.85
3.98
1.00
1.00
2.90
2.09
2.18
0.85

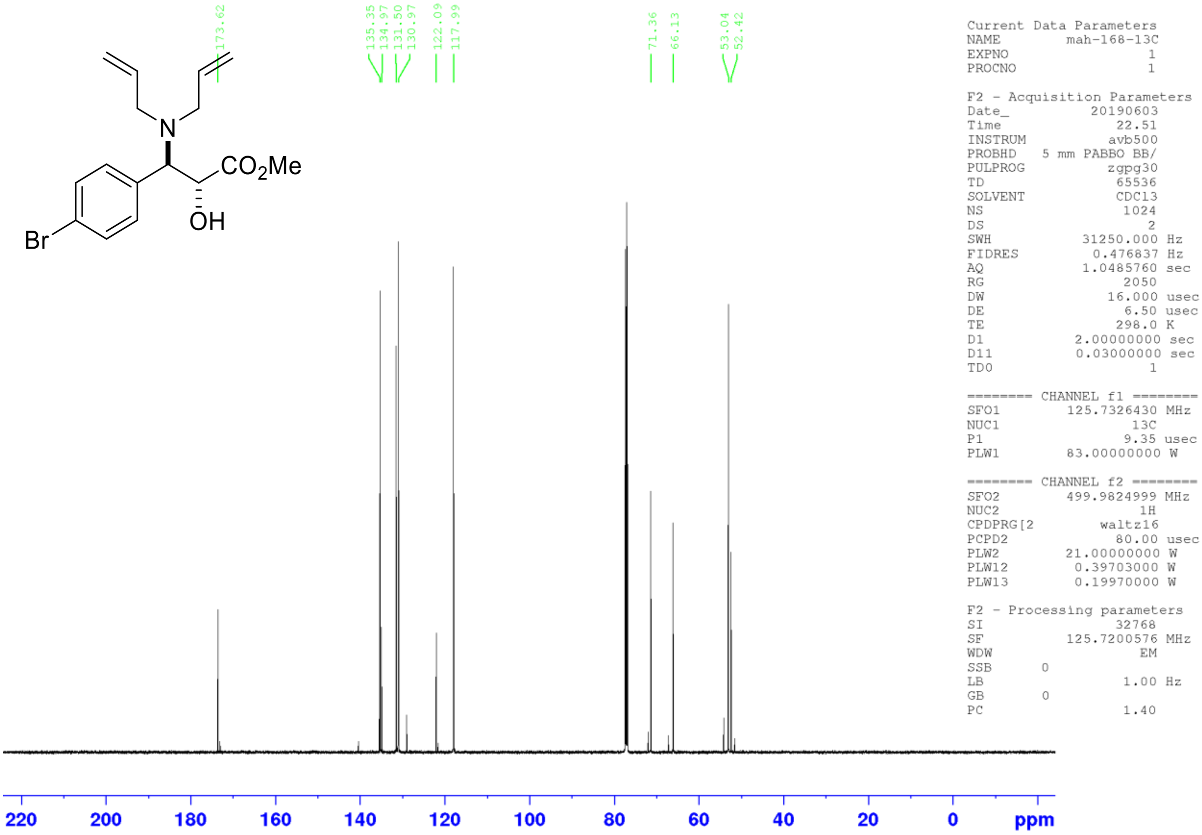
173.62
135.35
134.97
131.50
130.97
122.09
117.99
71.36
66.13
53.04
52.42
N
CO_2Me
OH
Br
220 200 180 160 140 120 100 80 60 40 20 0 ppm
Current Data Parameters
NAME mah-168-13C
EXPNO 1
PROCNO 1
F2 - Acquisition Parameters
Date_ 20190603
Time 22.51
INSTRUM avb500
PROBHD 5 mm PABBO BB/
PULPROG zgpg30
TD 65536
SOLVENT CDCl3
NS 1024
DS 2
SWH 31250.000 Hz
FIDRES 0.476837 Hz
AQ 1.0485760 sec
RG 2050
DW 16.000 usec
DE 6.50 usec
TE 298.0 K
D1 2.00000000 sec
D11 0.03000000 sec
TD0 1
======== CHANNEL f1 ========
SFO1 125.7326430 MHz
NUC1 13C
P1 9.35 usec
PLW1 83.00000000 W
======== CHANNEL f2 ========
SFO2 499.9824999 MHz
NUC2 1H
CPDPRG[2 waltz16
PCPD2 80.00 usec
PLW2 21.00000000 W
PLW12 0.39703000 W
PLW13 0.19970000 W
F2 - Processing parameters
SI 32768
SF 125.7200576 MHz
WDW EM
SSB 0
LB 1.00 Hz
GB 0
PC 1.40

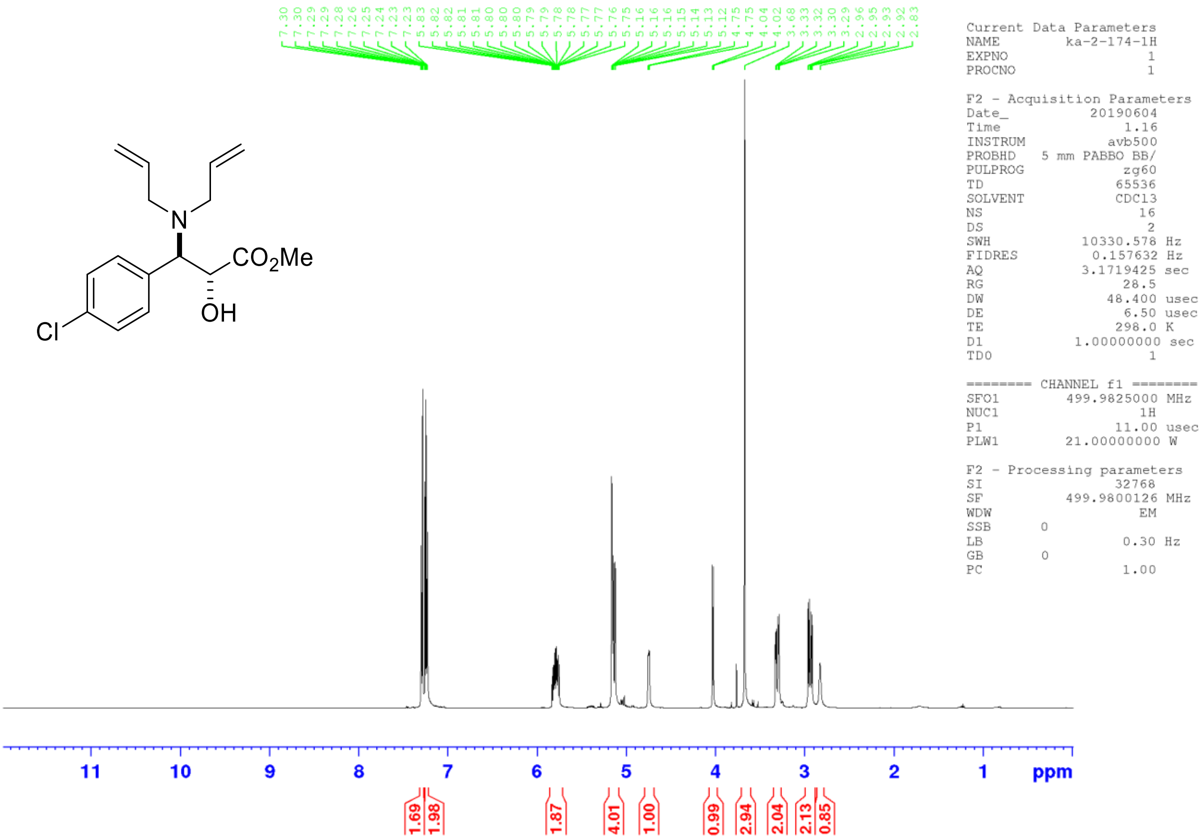
7.30
7.30
7.29
7.29
7.28
7.26
7.25
7.24
7.23
7.23
5.83
5.82
5.82
5.81
5.81
5.80
5.80
5.80
5.79
5.79
5.78
5.78
5.77
5.77
5.76
5.75
5.16
5.16
5.16
5.15
5.14
5.13
5.12
4.75
4.75
4.04
4.02
3.68
3.33
3.32
3.30
3.29
2.96
2.95
2.93
2.92
2.83
N
CO_2Me
OH
Cl
11 10 9 8 7 6 5 4 3 2 1 ppm
1.69
1.98
1.87
4.01
1.00
0.99
2.94
2.04
2.13
0.85
Current Data Parameters
NAME ka-2-174-1H
EXPNO 1
PROCNO 1
F2 - Acquisition Parameters
Date_ 20190604
Time 1.16
INSTRUM avb500
PROBHD 5 mm PABBO BB/
PULPROG zg60
TD 65536
SOLVENT CDCl3
NS 16
DS 2
SWH 10330.578 Hz
FIDRES 0.157632 Hz
AQ 3.1719425 sec
RG 28.5
DW 48.400 usec
DE 6.50 usec
TE 298.0 K
D1 1.00000000 sec
TD0 1
======== CHANNEL f1 ========
SFO1 499.9825000 MHz
NUC1 1H
P1 11.00 usec
PLW1 21.00000000 W
F2 - Processing parameters
SI 32768
SF 499.9800126 MHz
WDW EM
SSB 0
LB 0.30 Hz
GB 0
PC 1.00

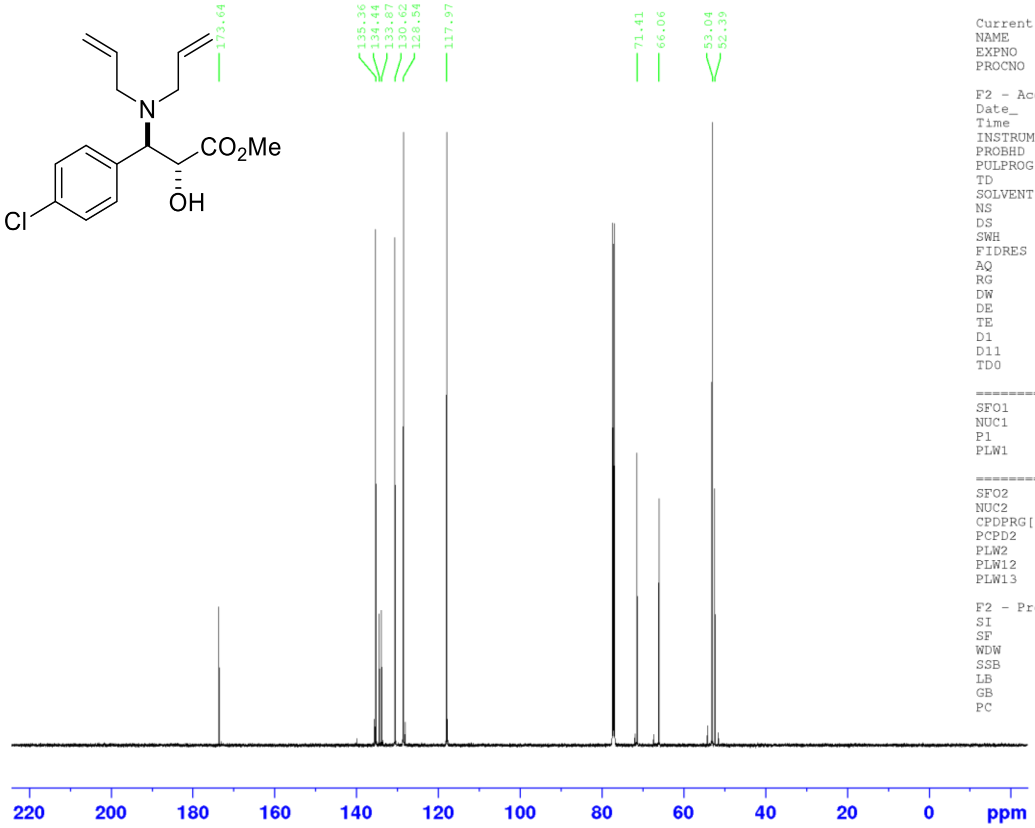


```
Current Data Parameters
NAME        ka-2-174-13C
EXPNO                  1
PROCNO                 1

F2 - Acquisition Parameters
Date_           20190604
Time                2.09
INSTRUM           avb500
PROBHD   5 mm PABBO BB/
PULPROG          zgpg30
TD                 65536
SOLVENT            CDCl3
NS                  1024
DS                     2
SWH            31250.000 Hz
FIDRES          0.476837 Hz
AQ             1.0485760 sec
RG                  2050
DW                16.000 usec
DE                  6.50 usec
TE                 298.0 K
D1            2.00000000 sec
D11           0.03000000 sec
TD0                    1

======== CHANNEL f1 ========
SFO1         125.7326430 MHz
NUC1                 13C
P1                  9.35 usec
PLW1         83.00000000 W

======== CHANNEL f2 ========
SFO2         499.9824999 MHz
NUC2                  1H
CPDPRG[2         waltz16
PCPD2              80.00 usec
PLW2         21.00000000 W
PLW12         0.39703000 W
PLW13         0.19970000 W

F2 - Processing parameters
SI                 32768
SF           125.7200577 MHz
WDW                   EM
SSB      0
LB                  1.00 Hz
GB       0
PC                  1.40
```

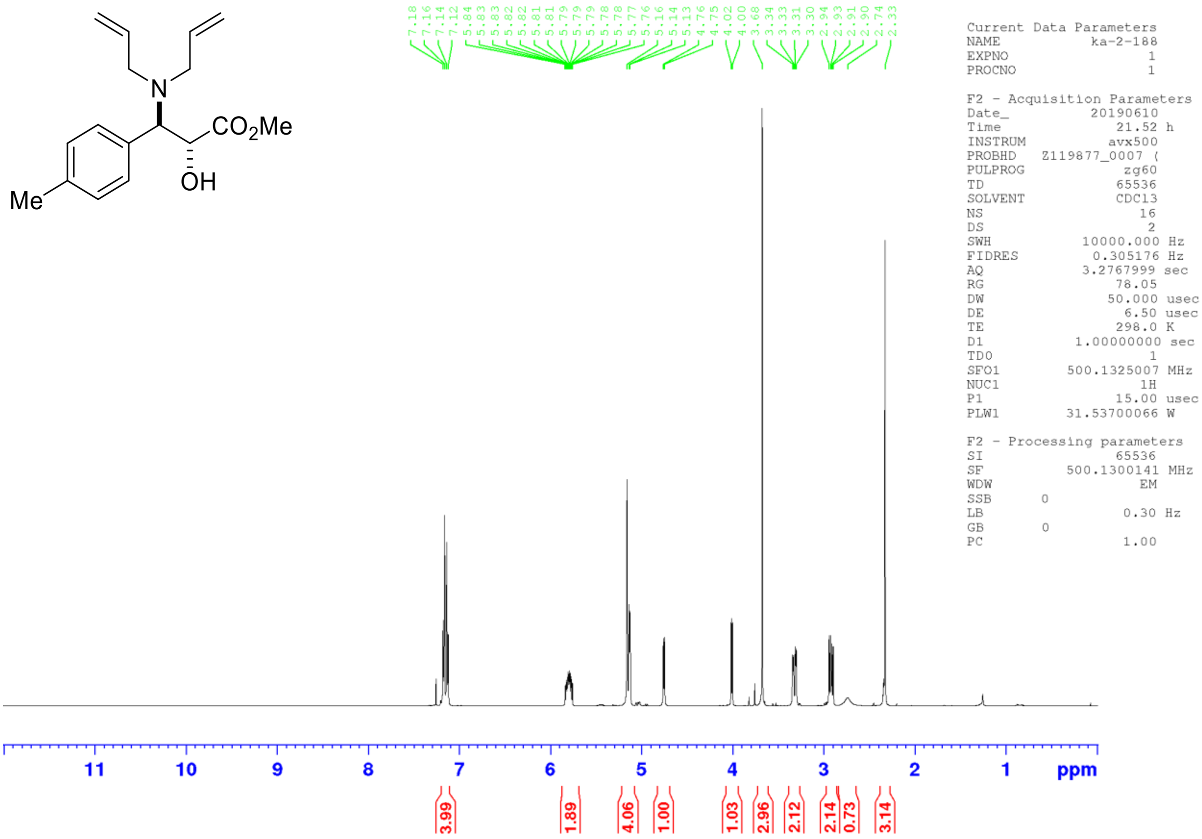


```
Current Data Parameters
NAME            ka-2-188
EXPNO                  1
PROCNO                 1

F2 - Acquisition Parameters
Date_           20190610
Time               21.52 h
INSTRUM           avx500
PROBHD   Z119877_0007 (
PULPROG             zg60
TD                 65536
SOLVENT            CDCl3
NS                    16
DS                     2
SWH            10000.000 Hz
FIDRES          0.305176 Hz
AQ             3.2767999 sec
RG                 78.05
DW                50.000 usec
DE                  6.50 usec
TE                 298.0 K
D1            1.00000000 sec
TD0                    1
SFO1         500.1325007 MHz
NUC1                  1H
P1                 15.00 usec
PLW1         31.53700066 W

F2 - Processing parameters
SI                 65536
SF           500.1300141 MHz
WDW                   EM
SSB      0
LB                  0.30 Hz
GB       0
PC                  1.00
```

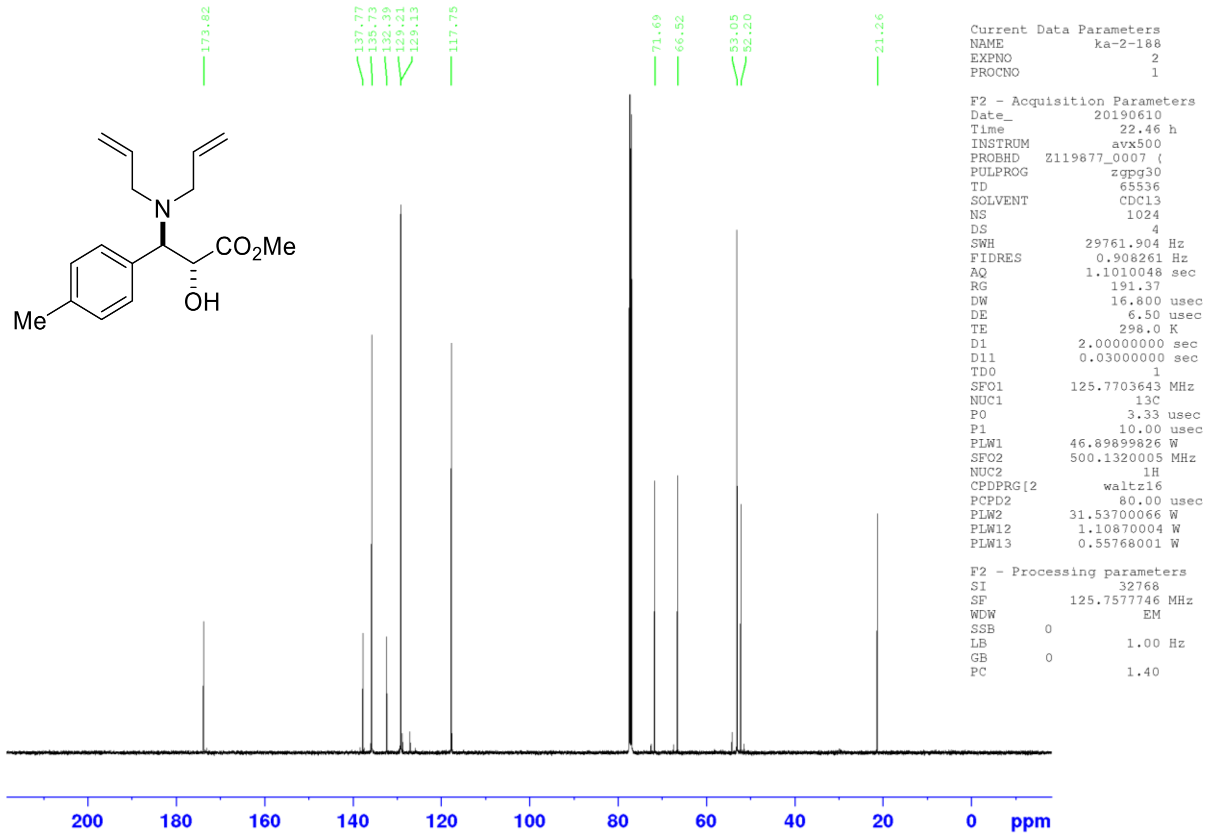
173.82
137.77
135.73
132.39
129.21
129.13
117.75
71.69
66.52
53.05
52.20
21.26
N
CO2Me
OH
Me
Current Data Parameters
NAME ka-2-188
EXPNO 2
PROCNO 1
F2 - Acquisition Parameters
Date_ 20190610
Time 22.46 h
INSTRUM avx500
PROBHD Z119877_0007 (
PULPROG zgpg30
TD 65536
SOLVENT CDCl3
NS 1024
DS 4
SWH 29761.904 Hz
FIDRES 0.908261 Hz
AQ 1.1010048 sec
RG 191.37
DW 16.800 usec
DE 6.50 usec
TE 298.0 K
D1 2.00000000 sec
D11 0.03000000 sec
TD0 1
SFO1 125.7703643 MHz
NUC1 13C
P0 3.33 usec
P1 10.00 usec
PLW1 46.89899826 W
SFO2 500.1320005 MHz
NUC2 1H
CPDPRG[2 waltz16
PCPD2 80.00 usec
PLW2 31.53700066 W
PLW12 1.10870004 W
PLW13 0.55768001 W
F2 - Processing parameters
SI 32768
SF 125.7577746 MHz
WDW EM
SSB 0
LB 1.00 Hz
GB 0
PC 1.40
200 180 160 140 120 100 80 60 40 20 0 ppm

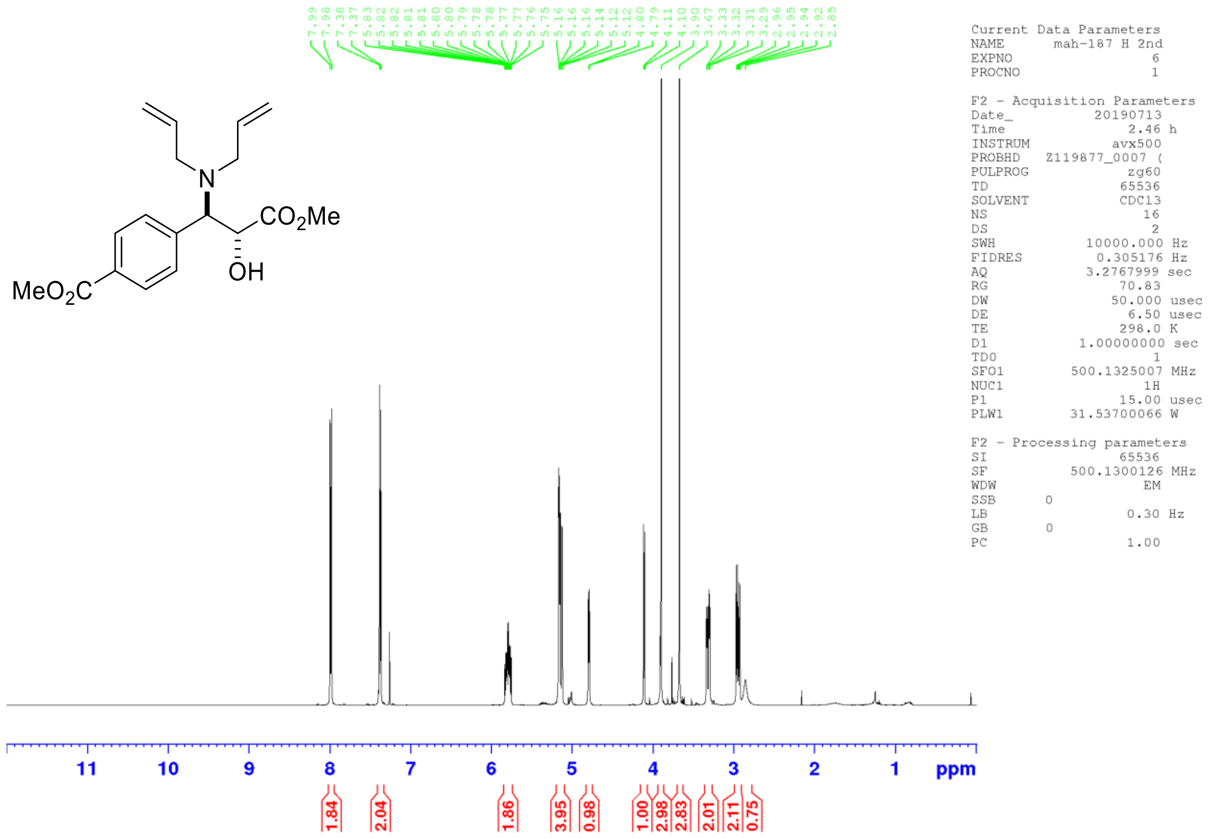
7.99
7.98
7.38
7.37
5.83
5.82
5.82
5.81
5.80
5.80
5.79
5.78
5.78
5.77
5.77
5.76
5.75
5.16
5.16
5.16
5.14
5.12
5.12
4.80
4.79
4.11
4.10
3.90
3.67
3.33
3.32
3.31
3.29
2.96
2.95
2.94
2.92
2.85
N
CO2Me
OH
MeO2C
Current Data Parameters
NAME mah-187 H 2nd
EXPNO 6
PROCNO 1
F2 - Acquisition Parameters
Date_ 20190713
Time 2.46 h
INSTRUM avx500
PROBHD Z119877_0007 (
PULPROG zg60
TD 65536
SOLVENT CDCl3
NS 16
DS 2
SWH 10000.000 Hz
FIDRES 0.305176 Hz
AQ 3.2767999 sec
RG 70.83
DW 50.000 usec
DE 6.50 usec
TE 298.0 K
D1 1.00000000 sec
TD0 1
SFO1 500.1325007 MHz
NUC1 1H
P1 15.00 usec
PLW1 31.53700066 W
F2 - Processing parameters
SI 65536
SF 500.1300126 MHz
WDW EM
SSB 0
LB 0.30 Hz
GB 0
PC 1.00
11 10 9 8 7 6 5 4 3 2 1 ppm
1.84
2.04
1.86
3.95
0.98
1.00
2.98
2.83
2.01
2.11
0.75

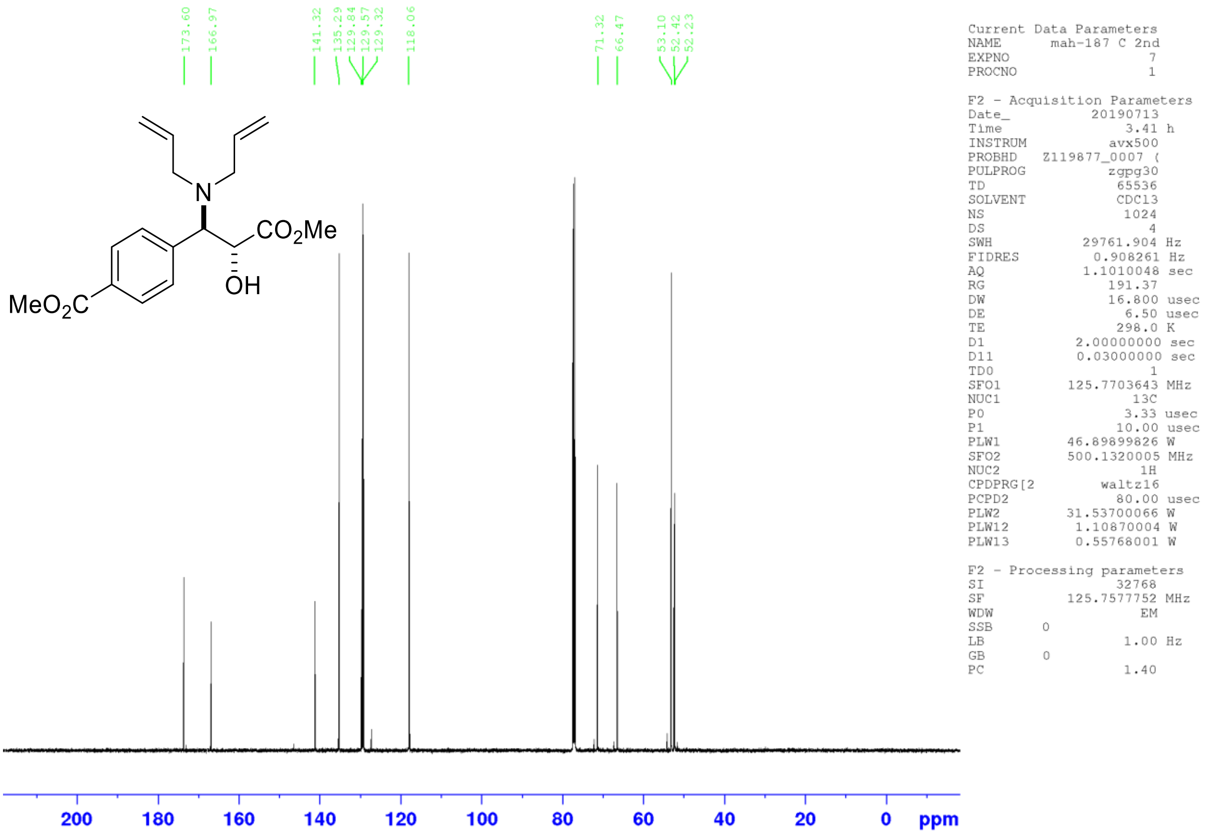
173.60
166.97
141.32
135.29
129.84
129.57
129.32
118.06
71.32
66.47
53.10
52.42
52.23
CO2Me
MeO2C
N
OH
Current Data Parameters
NAME mah-187 C 2nd
EXPNO 7
PROCNO 1
F2 - Acquisition Parameters
Date_ 20190713
Time 3.41 h
INSTRUM avx500
PROBHD Z119877_0007 (
PULPROG zgpg30
TD 65536
SOLVENT CDCl3
NS 1024
DS 4
SWH 29761.904 Hz
FIDRES 0.908261 Hz
AQ 1.1010048 sec
RG 191.37
DW 16.800 usec
DE 6.50 usec
TE 298.0 K
D1 2.00000000 sec
D11 0.03000000 sec
TD0 1
SFO1 125.7703643 MHz
NUC1 13C
P0 3.33 usec
P1 10.00 usec
PLW1 46.89899826 W
SFO2 500.1320005 MHz
NUC2 1H
CPDPRG[2 waltz16
PCPD2 80.00 usec
PLW2 31.53700066 W
PLW12 1.10870004 W
PLW13 0.55768001 W
F2 - Processing parameters
SI 32768
SF 125.7577752 MHz
WDW EM
SSB 0
LB 1.00 Hz
GB 0
PC 1.40
200
180
160
140
120
100
80
60
40
20
0
ppm

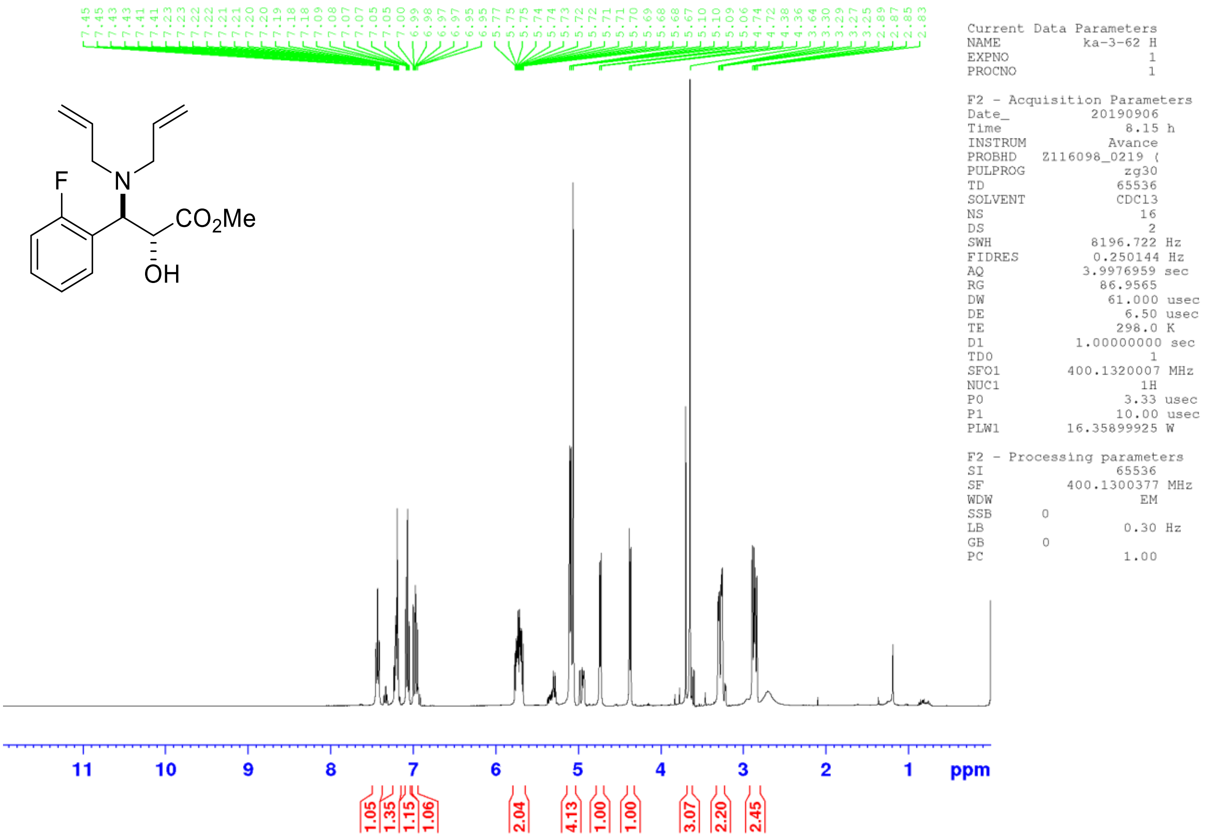
F
N
CO2Me
OH
Current Data Parameters
NAME ka-3-62 H
EXPNO 1
PROCNO 1
F2 - Acquisition Parameters
Date_ 20190906
Time 8.15 h
INSTRUM Avance
PROBHD Z116098_0219 (
PULPROG zg30
TD 65536
SOLVENT CDCl3
NS 16
DS 2
SWH 8196.722 Hz
FIDRES 0.250144 Hz
AQ 3.9976959 sec
RG 86.9565
DW 61.000 usec
DE 6.50 usec
TE 298.0 K
D1 1.00000000 sec
TD0 1
SFO1 400.1320007 MHz
NUC1 1H
P0 3.33 usec
P1 10.00 usec
PLW1 16.35899925 W
F2 - Processing parameters
SI 65536
SF 400.1300377 MHz
WDW EM
SSB 0
LB 0.30 Hz
GB 0
PC 1.00
11
10
9
8
7
6
5
4
3
2
1
ppm
1.05
1.35
1.15
1.06
2.04
4.13
1.00
1.00
3.07
2.20
2.45

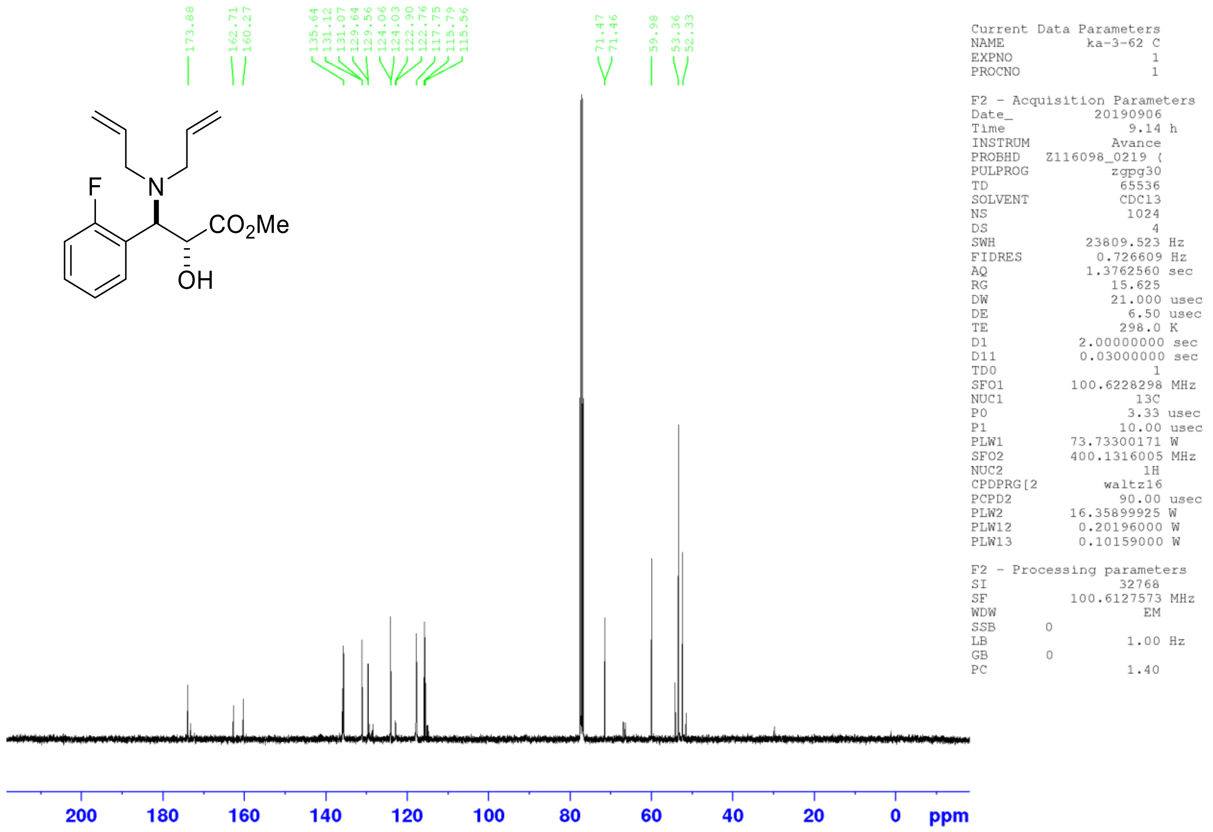

173.88
162.71
160.27
135.64
131.12
131.07
129.64
129.56
124.06
124.03
122.90
122.76
117.75
115.79
115.56
71.47
71.46
59.98
53.36
52.33
F
N
CO2Me
OH
Current Data Parameters
NAME ka-3-62 C
EXPNO 1
PROCNO 1
F2 - Acquisition Parameters
Date_ 20190906
Time 9.14 h
INSTRUM Avance
PROBHD Z116098_0219 (
PULPROG zgpg30
TD 65536
SOLVENT CDCl3
NS 1024
DS 4
SWH 23809.523 Hz
FIDRES 0.726609 Hz
AQ 1.3762560 sec
RG 15.625
DW 21.000 usec
DE 6.50 usec
TE 298.0 K
D1 2.00000000 sec
D11 0.03000000 sec
TD0 1
SFO1 100.6228298 MHz
NUC1 13C
P0 3.33 usec
P1 10.00 usec
PLW1 73.73300171 W
SFO2 400.1316005 MHz
NUC2 1H
CPDPRG[2 waltz16
PCPD2 90.00 usec
PLW2 16.35899925 W
PLW12 0.20196000 W
PLW13 0.10159000 W
F2 - Processing parameters
SI 32768
SF 100.6127573 MHz
WDW EM
SSB 0
LB 1.00 Hz
GB 0
PC 1.40
200
180
160
140
120
100
80
60
40
20
0
ppm

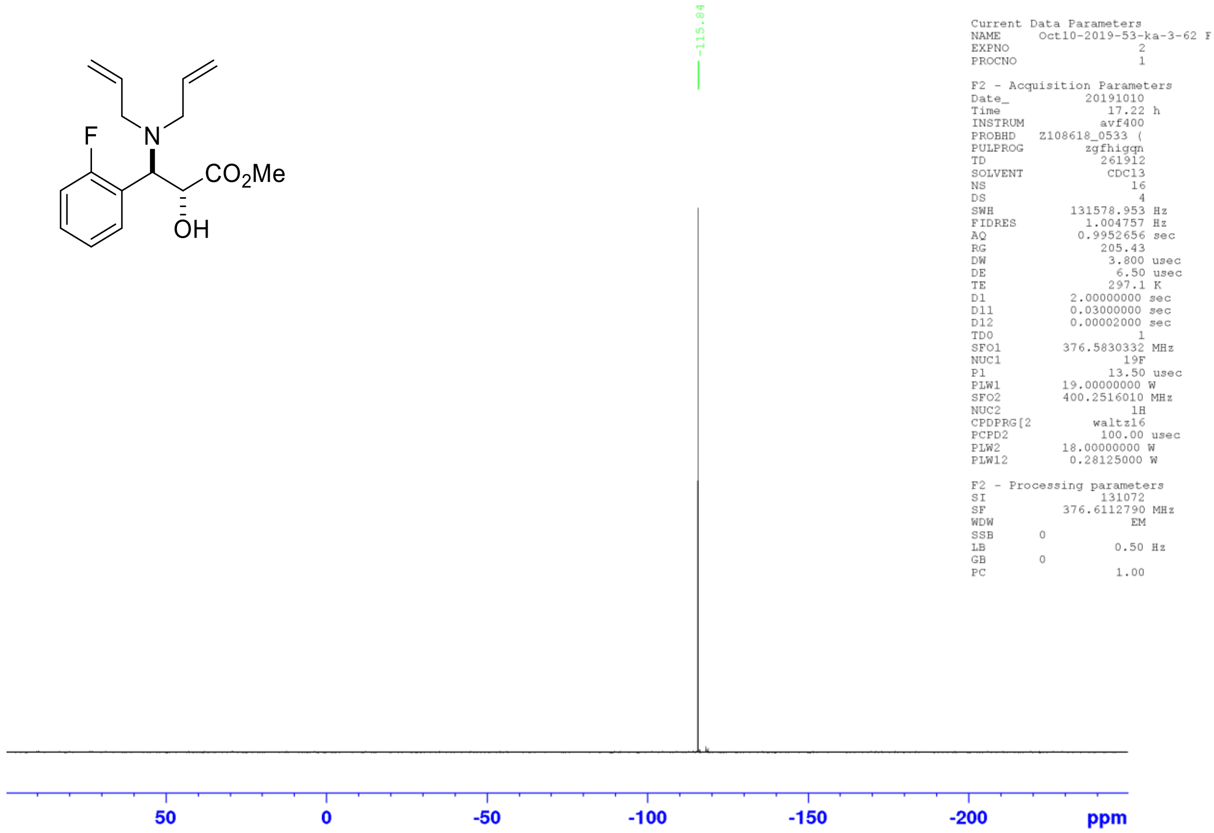

-115.84
F
N
CO2Me
OH
Current Data Parameters
NAME Oct10-2019-53-ka-3-62 F
EXPNO 2
PROCNO 1
F2 - Acquisition Parameters
Date_ 20191010
Time 17.22 h
INSTRUM avf400
PROBHD Z108618_0533 (
PULPROG zgfhigqn
TD 261912
SOLVENT CDCl3
NS 16
DS 4
SWH 131578.953 Hz
FIDRES 1.004757 Hz
AQ 0.9952656 sec
RG 205.43
DW 3.800 usec
DE 6.50 usec
TE 297.1 K
D1 2.00000000 sec
D11 0.03000000 sec
D12 0.00002000 sec
TD0 1
SFO1 376.5830332 MHz
NUC1 19F
P1 13.50 usec
PLW1 19.00000000 W
SFO2 400.2516010 MHz
NUC2 1H
CPDPRG[2 waltz16
PCPD2 100.00 usec
PLW2 18.00000000 W
PLW12 0.28125000 W
F2 - Processing parameters
SI 131072
SF 376.6112790 MHz
WDW EM
SSB 0
LB 0.50 Hz
GB 0
PC 1.00
50
0
-50
-100
-150
-200
ppm

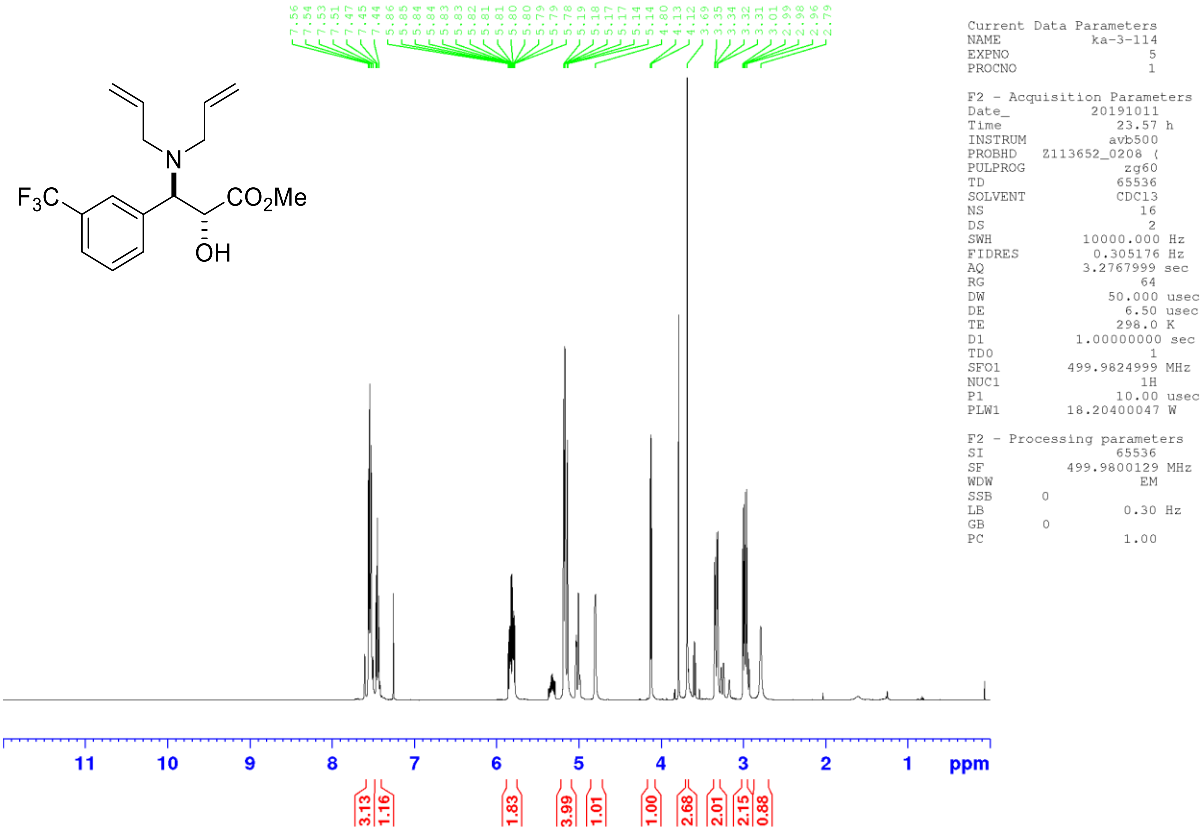


```
Current Data Parameters
NAME              ka-3-114
EXPNO                    5
PROCNO                   1

F2 - Acquisition Parameters
Date_             20191011
Time                 23.57 h
INSTRUM             avb500
PROBHD   Z113652_0208 (
PULPROG               zg60
TD                   65536
SOLVENT              CDCl3
NS                      16
DS                       2
SWH              10000.000 Hz
FIDRES            0.305176 Hz
AQ               3.2767999 sec
RG                      64
DW                  50.000 usec
DE                    6.50 usec
TE                   298.0 K
D1              1.00000000 sec
TD0                      1
SFO1           499.9824999 MHz
NUC1                    1H
P1                   10.00 usec
PLW1           18.20400047 W

F2 - Processing parameters
SI                   65536
SF             499.9800129 MHz
WDW                     EM
SSB      0
LB                    0.30 Hz
GB       0
PC                    1.00
```

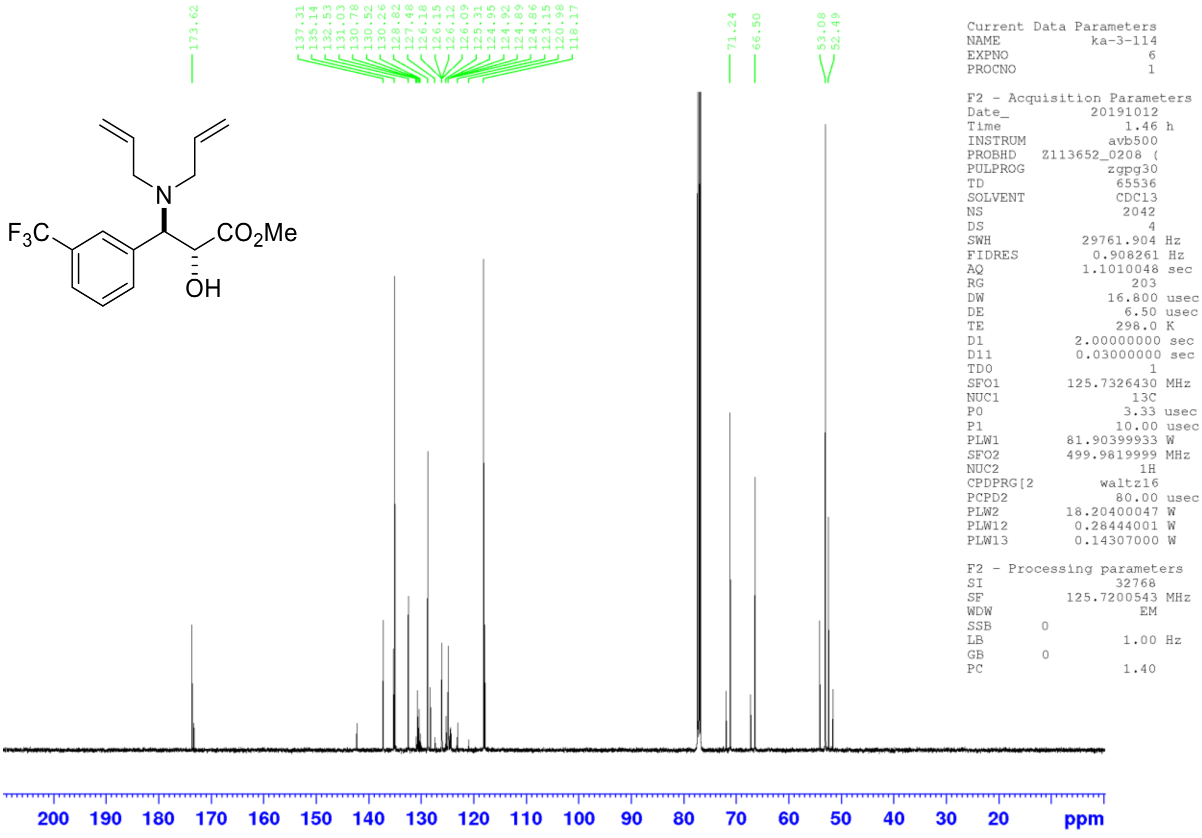


```
Current Data Parameters
NAME              ka-3-114
EXPNO                    6
PROCNO                   1

F2 - Acquisition Parameters
Date_             20191012
Time                  1.46 h
INSTRUM             avb500
PROBHD   Z113652_0208 (
PULPROG             zgpg30
TD                   65536
SOLVENT              CDCl3
NS                    2042
DS                       4
SWH              29761.904 Hz
FIDRES            0.908261 Hz
AQ               1.1010048 sec
RG                     203
DW                  16.800 usec
DE                    6.50 usec
TE                   298.0 K
D1              2.00000000 sec
D11             0.03000000 sec
TD0                      1
SFO1           125.7326430 MHz
NUC1                   13C
P0                    3.33 usec
P1                   10.00 usec
PLW1           81.90399933 W
SFO2           499.9819999 MHz
NUC2                    1H
CPDPRG[2           waltz16
PCPD2                80.00 usec
PLW2           18.20400047 W
PLW12           0.28444001 W
PLW13           0.14307000 W

F2 - Processing parameters
SI                   32768
SF             125.7200543 MHz
WDW                     EM
SSB      0
LB                    1.00 Hz
GB       0
PC                    1.40
```

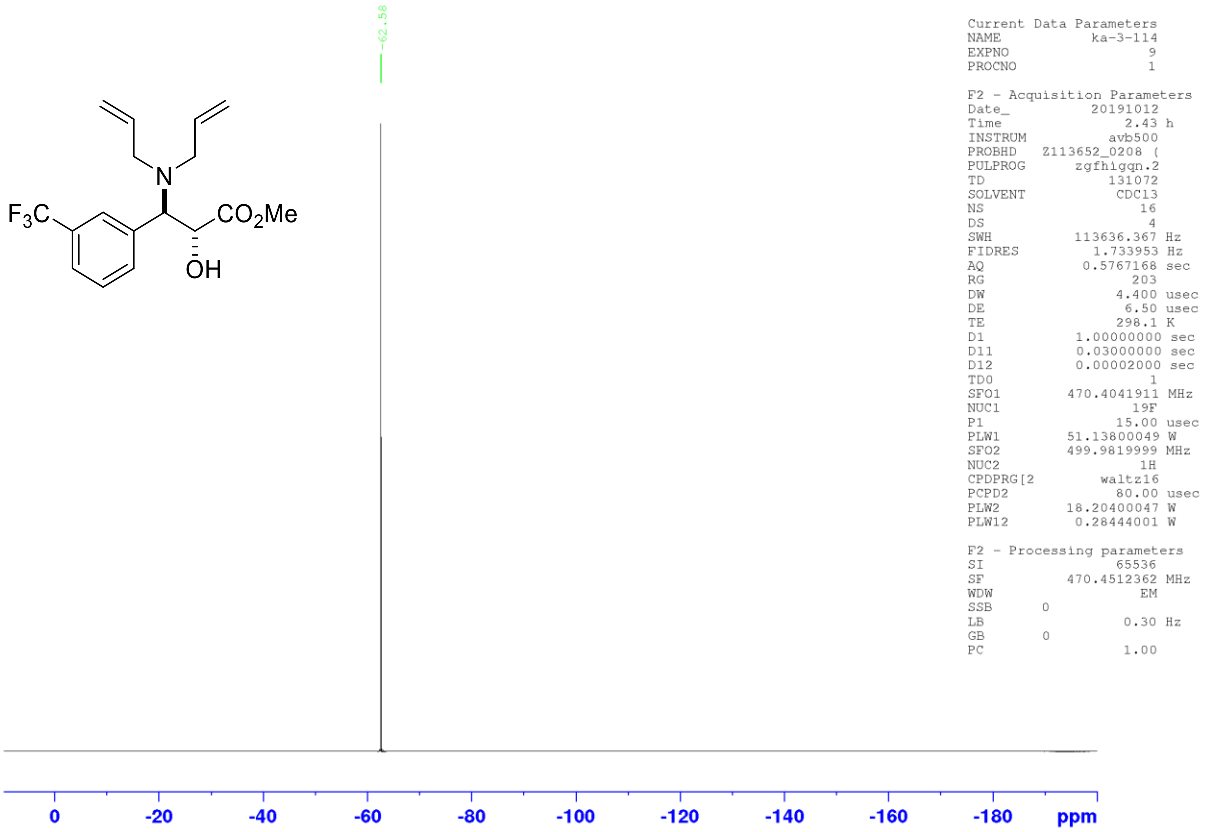

F3C
N
CO2Me
OH
-62.58
Current Data Parameters
NAME ka-3-114
EXPNO 9
PROCNO 1
F2 - Acquisition Parameters
Date_ 20191012
Time 2.43 h
INSTRUM avb500
PROBHD Z113652_0208 (
PULPROG zgfhigqn.2
TD 131072
SOLVENT CDCl3
NS 16
DS 4
SWH 113636.367 Hz
FIDRES 1.733953 Hz
AQ 0.5767168 sec
RG 203
DW 4.400 usec
DE 6.50 usec
TE 298.1 K
D1 1.00000000 sec
D11 0.03000000 sec
D12 0.00002000 sec
TD0 1
SFO1 470.4041911 MHz
NUC1 19F
P1 15.00 usec
PLW1 51.13800049 W
SFO2 499.9819999 MHz
NUC2 1H
CPDPRG[2 waltz16
PCPD2 80.00 usec
PLW2 18.20400047 W
PLW12 0.28444001 W
F2 - Processing parameters
SI 65536
SF 470.4512362 MHz
WDW EM
SSB 0
LB 0.30 Hz
GB 0
PC 1.00
0
-20
-40
-60
-80
-100
-120
-140
-160
-180
ppm

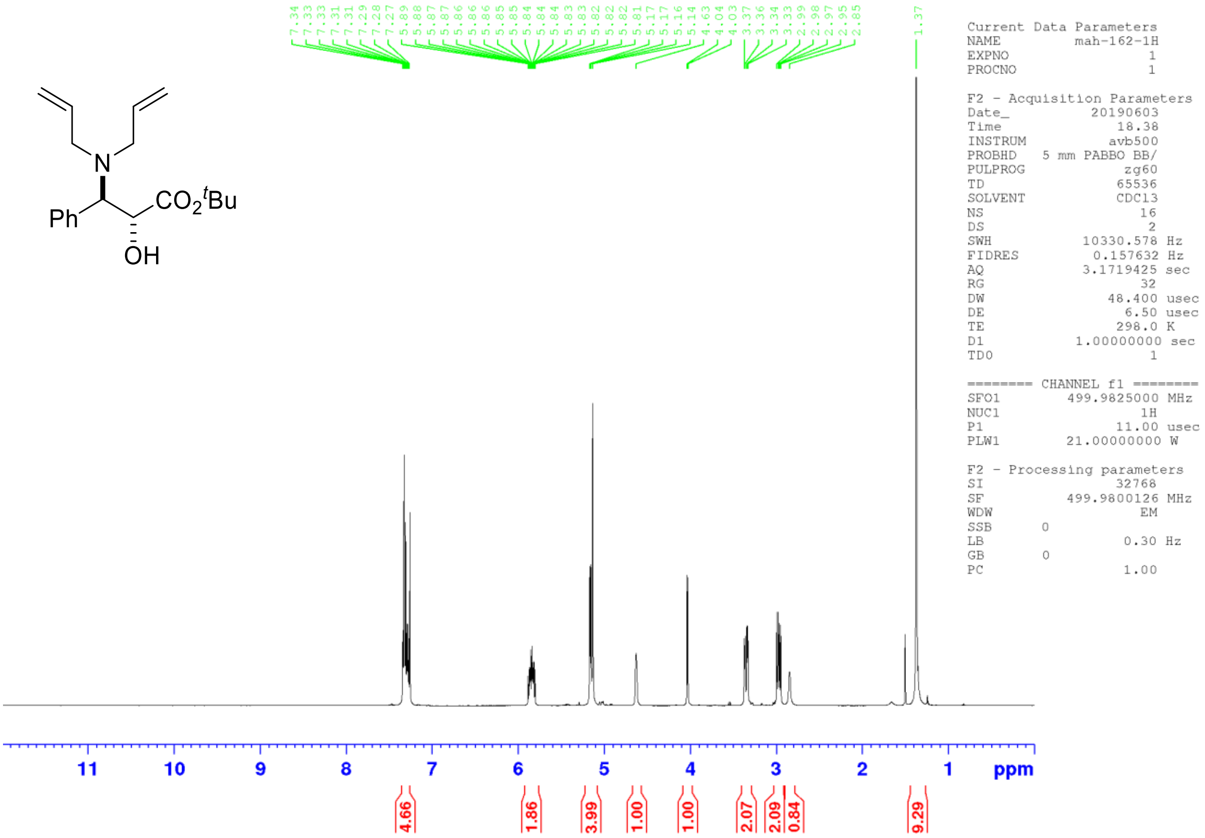

N
Ph
CO2tBu
OH
7.34 7.33 7.33 7.31 7.31 7.29 7.28 7.27 5.89 5.88 5.87 5.87 5.86 5.86 5.86 5.85 5.85 5.84 5.84 5.84 5.83 5.83 5.82 5.82 5.82 5.81 5.17 5.16 5.14 4.63 4.04 4.03 3.37 3.36 3.34 3.33 2.99 2.98 2.97 2.95 2.85
1.37
Current Data Parameters
NAME mah-162-1H
EXPNO 1
PROCNO 1
F2 - Acquisition Parameters
Date_ 20190603
Time 18.38
INSTRUM avb500
PROBHD 5 mm PABBO BB/
PULPROG zg60
TD 65536
SOLVENT CDCl3
NS 16
DS 2
SWH 10330.578 Hz
FIDRES 0.157632 Hz
AQ 3.1719425 sec
RG 32
DW 48.400 usec
DE 6.50 usec
TE 298.0 K
D1 1.00000000 sec
TD0 1
======== CHANNEL f1 ========
SFO1 499.9825000 MHz
NUC1 1H
P1 11.00 usec
PLW1 21.00000000 W
F2 - Processing parameters
SI 32768
SF 499.9800126 MHz
WDW EM
SSB 0
LB 0.30 Hz
GB 0
PC 1.00
11
10
9
8
7
6
5
4
3
2
1
ppm
4.66
1.86
3.99
1.00
1.00
2.07
2.09
0.84
9.29

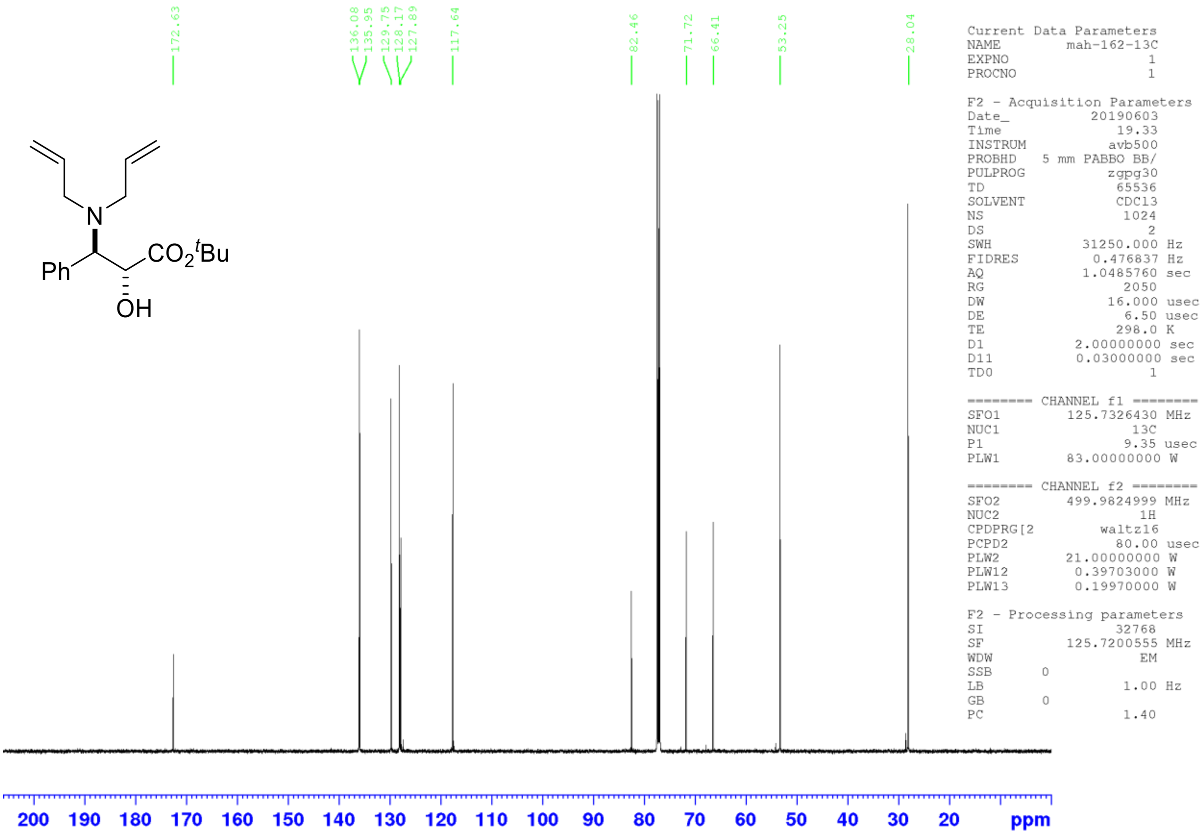
172.63
136.08
135.95
129.75
128.17
127.89
117.64
82.46
71.72
66.41
53.25
28.04
N
Ph
$CO_2{}^tBu$
OH
Current Data Parameters
NAME mah-162-13C
EXPNO 1
PROCNO 1
F2 - Acquisition Parameters
Date_ 20190603
Time 19.33
INSTRUM avb500
PROBHD 5 mm PABBO BB/
PULPROG zgpg30
TD 65536
SOLVENT CDCl3
NS 1024
DS 2
SWH 31250.000 Hz
FIDRES 0.476837 Hz
AQ 1.0485760 sec
RG 2050
DW 16.000 usec
DE 6.50 usec
TE 298.0 K
D1 2.00000000 sec
D11 0.03000000 sec
TD0 1
======== CHANNEL f1 ========
SFO1 125.7326430 MHz
NUC1 13C
P1 9.35 usec
PLW1 83.00000000 W
======== CHANNEL f2 ========
SFO2 499.9824999 MHz
NUC2 1H
CPDPRG[2 waltz16
PCPD2 80.00 usec
PLW2 21.00000000 W
PLW12 0.39703000 W
PLW13 0.19970000 W
F2 - Processing parameters
SI 32768
SF 125.7200555 MHz
WDW EM
SSB 0
LB 1.00 Hz
GB 0
PC 1.40
200 190 180 170 160 150 140 130 120 110 100 90 80 70 60 50 40 30 20 ppm

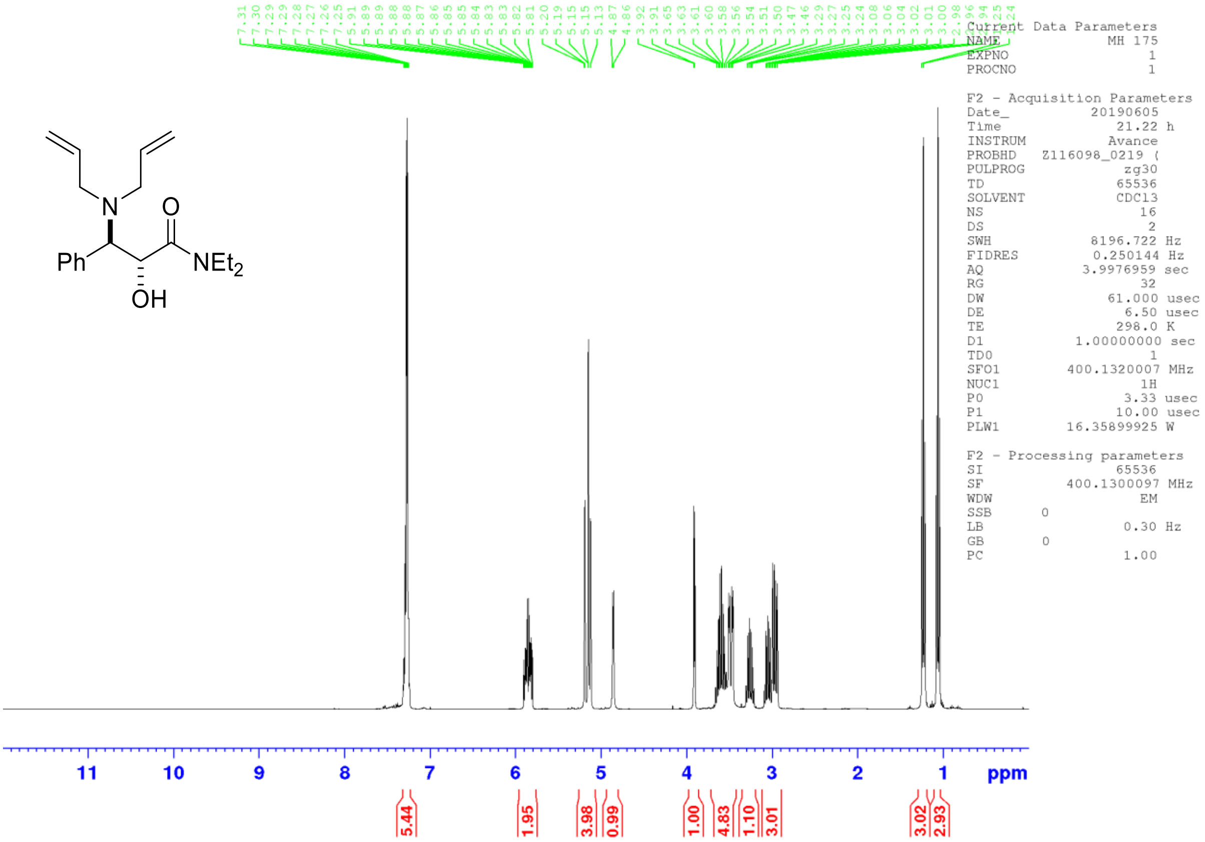
7.31
7.30
7.29
7.28
7.27
7.26
7.25
5.91
5.89
5.89
5.88
5.87
5.86
5.85
5.84
5.83
5.82
5.81
5.20
5.19
5.15
5.15
5.13
4.87
4.86
3.92
3.91
3.65
3.63
3.61
3.60
3.58
3.56
3.54
3.51
3.50
3.47
3.46
3.29
3.27
3.25
3.24
3.08
3.06
3.04
3.02
3.01
3.00
2.98
N
O
Ph
NEt_2
OH
Current Data Parameters
NAME MH 175
EXPNO 1
PROCNO 1
F2 - Acquisition Parameters
Date_ 20190605
Time 21.22 h
INSTRUM Avance
PROBHD Z116098_0219 (
PULPROG zg30
TD 65536
SOLVENT CDCl3
NS 16
DS 2
SWH 8196.722 Hz
FIDRES 0.250144 Hz
AQ 3.9976959 sec
RG 32
DW 61.000 usec
DE 6.50 usec
TE 298.0 K
D1 1.00000000 sec
TD0 1
SFO1 400.1320007 MHz
NUC1 1H
P0 3.33 usec
P1 10.00 usec
PLW1 16.35899925 W
F2 - Processing parameters
SI 65536
SF 400.1300097 MHz
WDW EM
SSB 0
LB 0.30 Hz
GB 0
PC 1.00
11 10 9 8 7 6 5 4 3 2 1 ppm
5.44
1.95
3.98
0.99
1.00
4.83
1.10
3.01
3.02
2.93

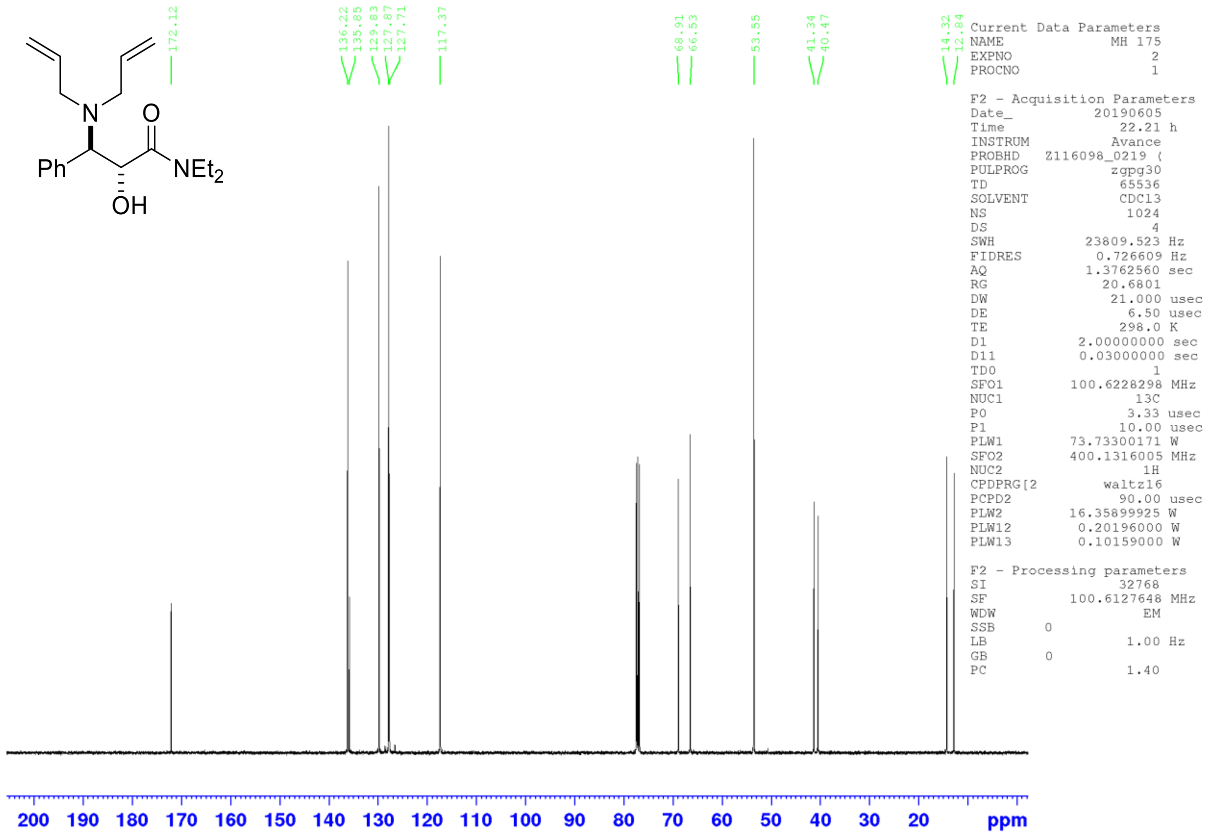
N
O
Ph
NEt_2
OH
172.12
136.22
135.85
129.83
127.87
127.71
117.37
68.91
66.53
53.55
41.34
40.47
14.32
12.84
Current Data Parameters
NAME MH 175
EXPNO 2
PROCNO 1
F2 - Acquisition Parameters
Date_ 20190605
Time 22.21 h
INSTRUM Avance
PROBHD Z116098_0219 (
PULPROG zgpg30
TD 65536
SOLVENT CDCl3
NS 1024
DS 4
SWH 23809.523 Hz
FIDRES 0.726609 Hz
AQ 1.3762560 sec
RG 20.6801
DW 21.000 usec
DE 6.50 usec
TE 298.0 K
D1 2.00000000 sec
D11 0.03000000 sec
TD0 1
SFO1 100.6228298 MHz
NUC1 13C
P0 3.33 usec
P1 10.00 usec
PLW1 73.73300171 W
SFO2 400.1316005 MHz
NUC2 1H
CPDPRG[2 waltz16
PCPD2 90.00 usec
PLW2 16.35899925 W
PLW12 0.20196000 W
PLW13 0.10159000 W
F2 - Processing parameters
SI 32768
SF 100.6127648 MHz
WDW EM
SSB 0
LB 1.00 Hz
GB 0
PC 1.40
200 190 180 170 160 150 140 130 120 110 100 90 80 70 60 50 40 30 20 ppm

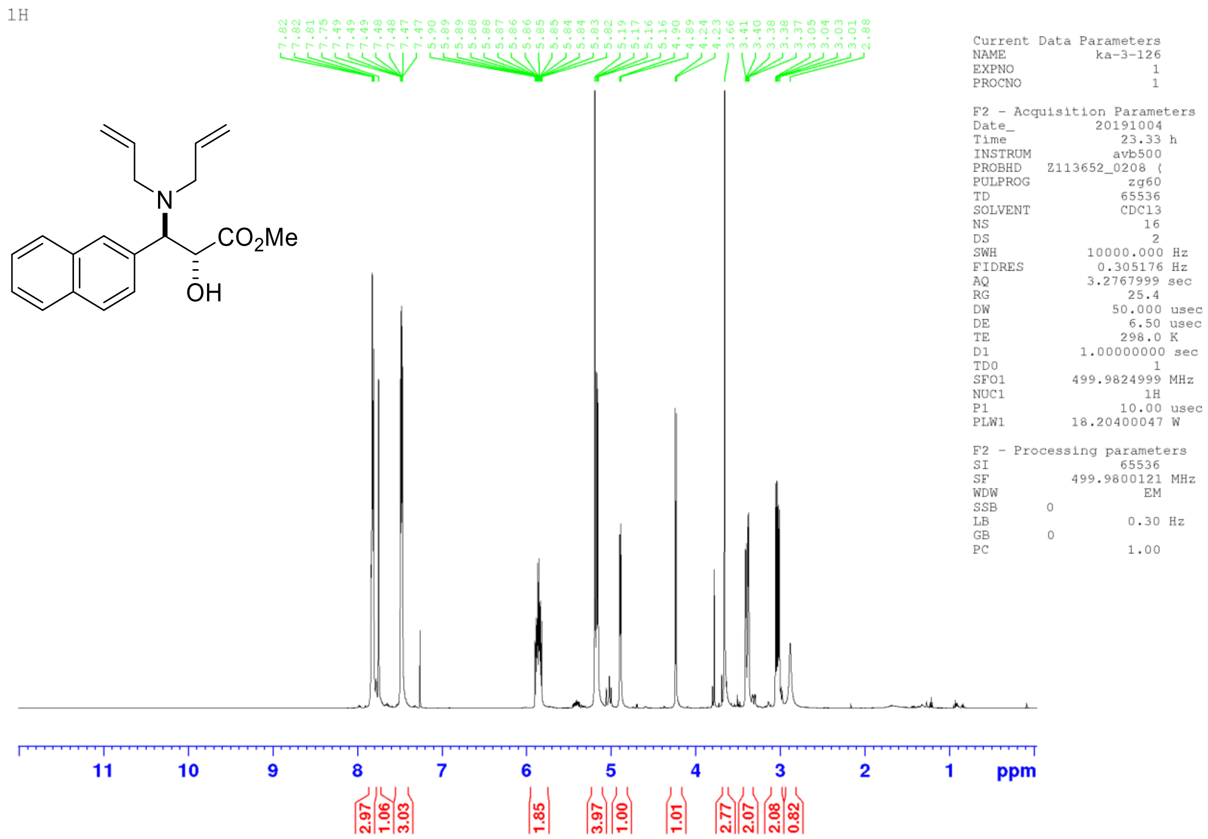
1H
N
CO_2Me
OH
7.82
7.82
7.81
7.75
7.49
7.49
7.49
7.48
7.48
7.47
7.47
5.90
5.89
5.89
5.88
5.87
5.86
5.86
5.85
5.85
5.84
5.84
5.83
5.82
5.19
5.17
5.16
5.16
4.90
4.89
4.24
4.23
3.66
3.41
3.40
3.38
3.38
3.37
3.05
3.04
3.03
3.01
2.88
Current Data Parameters
NAME ka-3-126
EXPNO 1
PROCNO 1
F2 - Acquisition Parameters
Date_ 20191004
Time 23.33 h
INSTRUM avb500
PROBHD Z113652_0208 (
PULPROG zg60
TD 65536
SOLVENT CDCl3
NS 16
DS 2
SWH 10000.000 Hz
FIDRES 0.305176 Hz
AQ 3.2767999 sec
RG 25.4
DW 50.000 usec
DE 6.50 usec
TE 298.0 K
D1 1.00000000 sec
TD0 1
SFO1 499.9824999 MHz
NUC1 1H
P1 10.00 usec
PLW1 18.20400047 W
F2 - Processing parameters
SI 65536
SF 499.9800121 MHz
WDW EM
SSB 0
LB 0.30 Hz
GB 0
PC 1.00
11 10 9 8 7 6 5 4 3 2 1 ppm
2.97
1.06
3.03
1.85
3.97
1.00
1.01
2.77
2.07
2.08
0.82

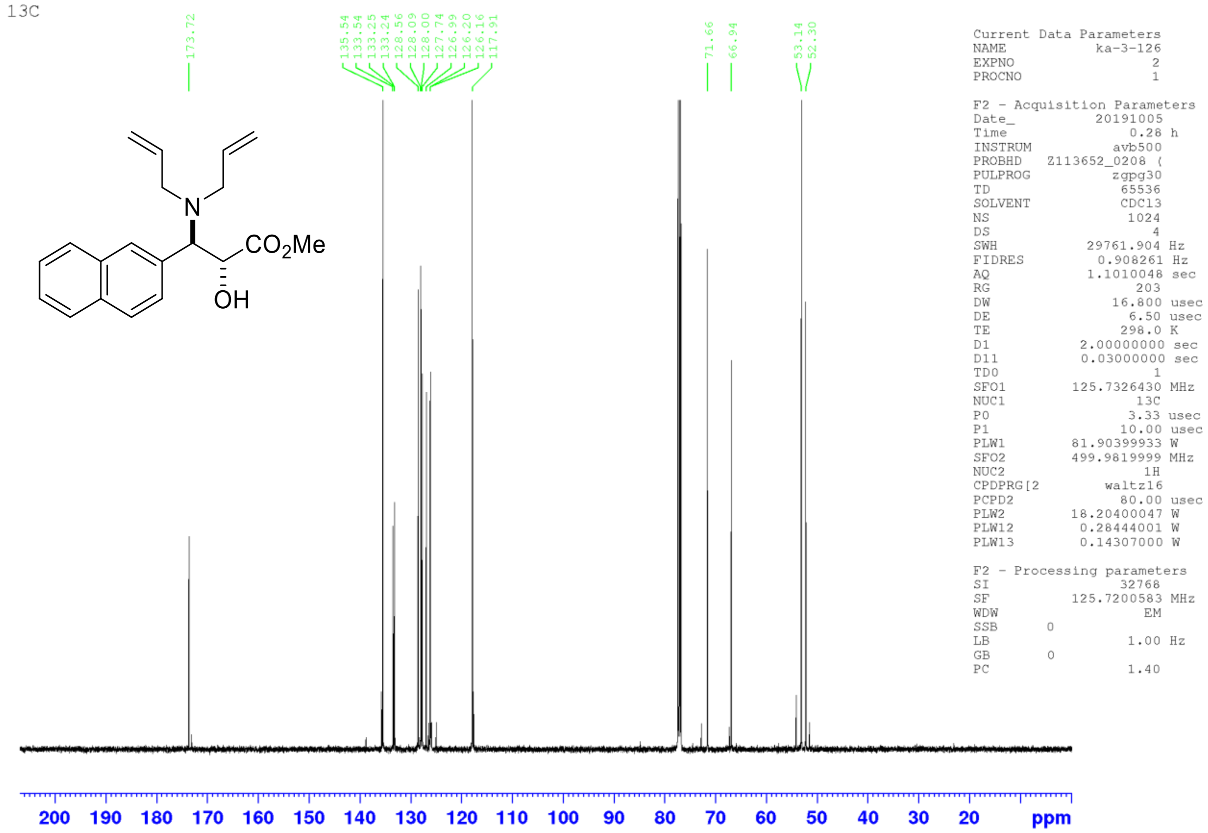
13C
173.72
135.54
133.54
133.25
133.24
128.56
128.09
128.00
127.74
126.99
126.20
126.16
117.91
71.66
66.94
53.14
52.30
N
CO2Me
OH
Current Data Parameters
NAME ka-3-126
EXPNO 2
PROCNO 1
F2 - Acquisition Parameters
Date_ 20191005
Time 0.28 h
INSTRUM avb500
PROBHD Z113652_0208 (
PULPROG zgpg30
TD 65536
SOLVENT CDCl3
NS 1024
DS 4
SWH 29761.904 Hz
FIDRES 0.908261 Hz
AQ 1.1010048 sec
RG 203
DW 16.800 usec
DE 6.50 usec
TE 298.0 K
D1 2.00000000 sec
D11 0.03000000 sec
TD0 1
SFO1 125.7326430 MHz
NUC1 13C
P0 3.33 usec
P1 10.00 usec
PLW1 81.90399933 W
SFO2 499.9819999 MHz
NUC2 1H
CPDPRG[2 waltz16
PCPD2 80.00 usec
PLW2 18.20400047 W
PLW12 0.28444001 W
PLW13 0.14307000 W
F2 - Processing parameters
SI 32768
SF 125.7200583 MHz
WDW EM
SSB 0
LB 1.00 Hz
GB 0
PC 1.40
200 190 180 170 160 150 140 130 120 110 100 90 80 70 60 50 40 30 20 ppm

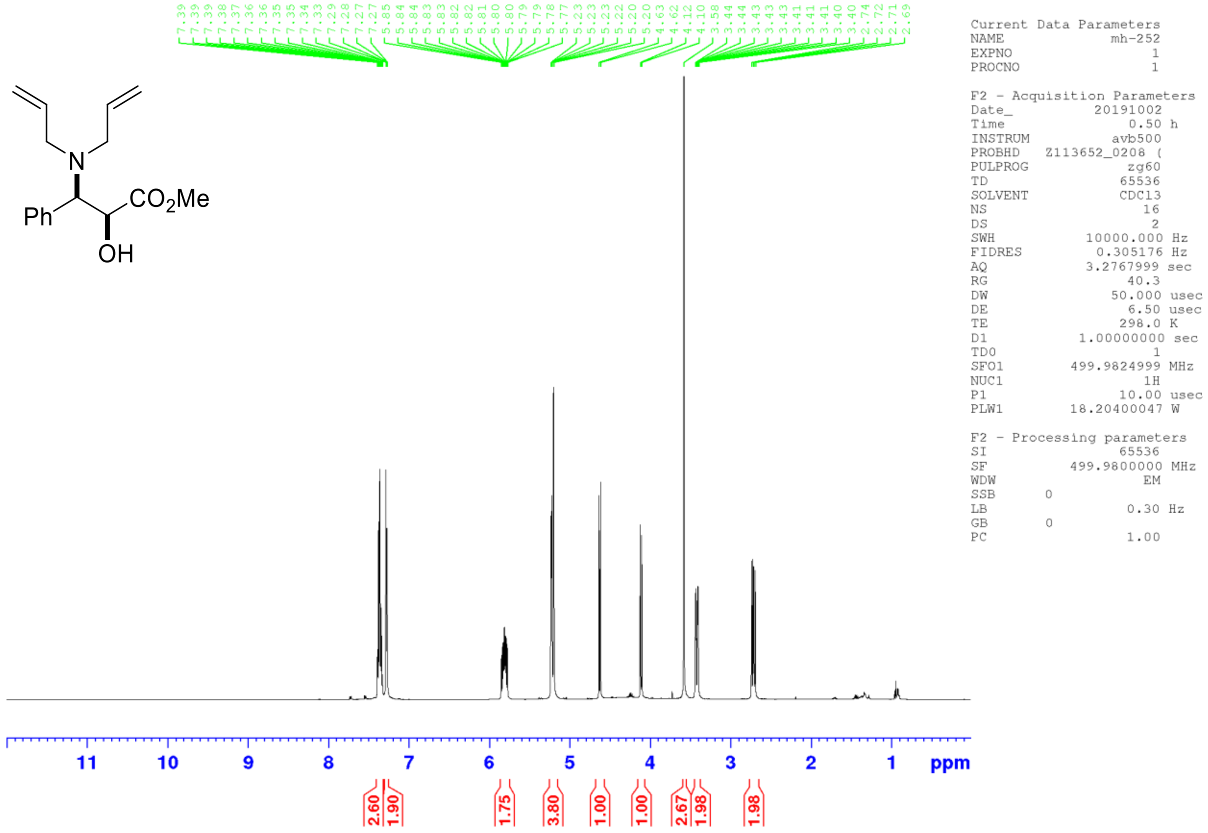
7.39 7.39 7.38 7.37 7.36 7.35 7.35 7.33 7.29 7.28 7.27 5.85 5.84 5.83 5.83 5.82 5.81 5.80 5.79 5.78 5.77 5.23 5.23 5.22 5.20 4.63 4.62 4.12 4.10 3.58 3.44 3.43 3.43 3.41 3.40 2.74 2.72 2.71 2.69
N
Ph
CO2Me
OH
Current Data Parameters
NAME mh-252
EXPNO 1
PROCNO 1
F2 - Acquisition Parameters
Date_ 20191002
Time 0.50 h
INSTRUM avb500
PROBHD Z113652_0208 (
PULPROG zg60
TD 65536
SOLVENT CDCl3
NS 16
DS 2
SWH 10000.000 Hz
FIDRES 0.305176 Hz
AQ 3.2767999 sec
RG 40.3
DW 50.000 usec
DE 6.50 usec
TE 298.0 K
D1 1.00000000 sec
TD0 1
SFO1 499.9824999 MHz
NUC1 1H
P1 10.00 usec
PLW1 18.20400047 W
F2 - Processing parameters
SI 65536
SF 499.9800000 MHz
WDW EM
SSB 0
LB 0.30 Hz
GB 0
PC 1.00
11 10 9 8 7 6 5 4 3 2 1 ppm
2.60 1.90 1.75 3.80 1.00 1.00 2.67 1.98 1.98

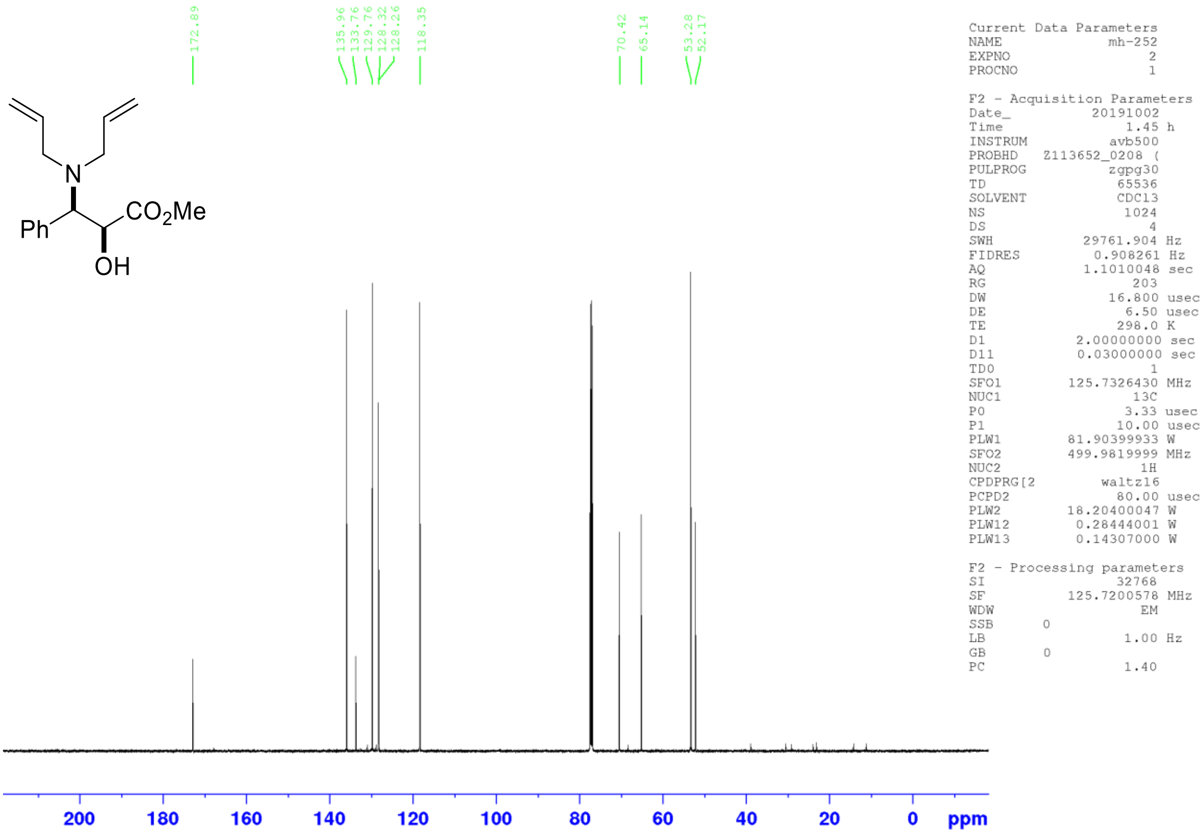


```
Current Data Parameters
NAME              mh-252
EXPNO                  2
PROCNO                 1

F2 - Acquisition Parameters
Date_           20191002
Time                1.45 h
INSTRUM           avb500
PROBHD    Z113652_0208 (
PULPROG          zgpg30
TD                65536
SOLVENT            CDCl3
NS                 1024
DS                    4
SWH           29761.904 Hz
FIDRES         0.908261 Hz
AQ            1.1010048 sec
RG                  203
DW               16.800 usec
DE                 6.50 usec
TE                298.0 K
D1           2.00000000 sec
D11          0.03000000 sec
TD0                   1
SFO1        125.7326430 MHz
NUC1                13C
P0                 3.33 usec
P1                10.00 usec
PLW1        81.90399933 W
SFO2        499.9819999 MHz
NUC2                 1H
CPDPRG[2        waltz16
PCPD2             80.00 usec
PLW2        18.20400047 W
PLW12        0.28444001 W
PLW13        0.14307000 W

F2 - Processing parameters
SI                32768
SF          125.7200578 MHz
WDW                  EM
SSB      0
LB                 1.00 Hz
GB       0
PC                 1.40
```

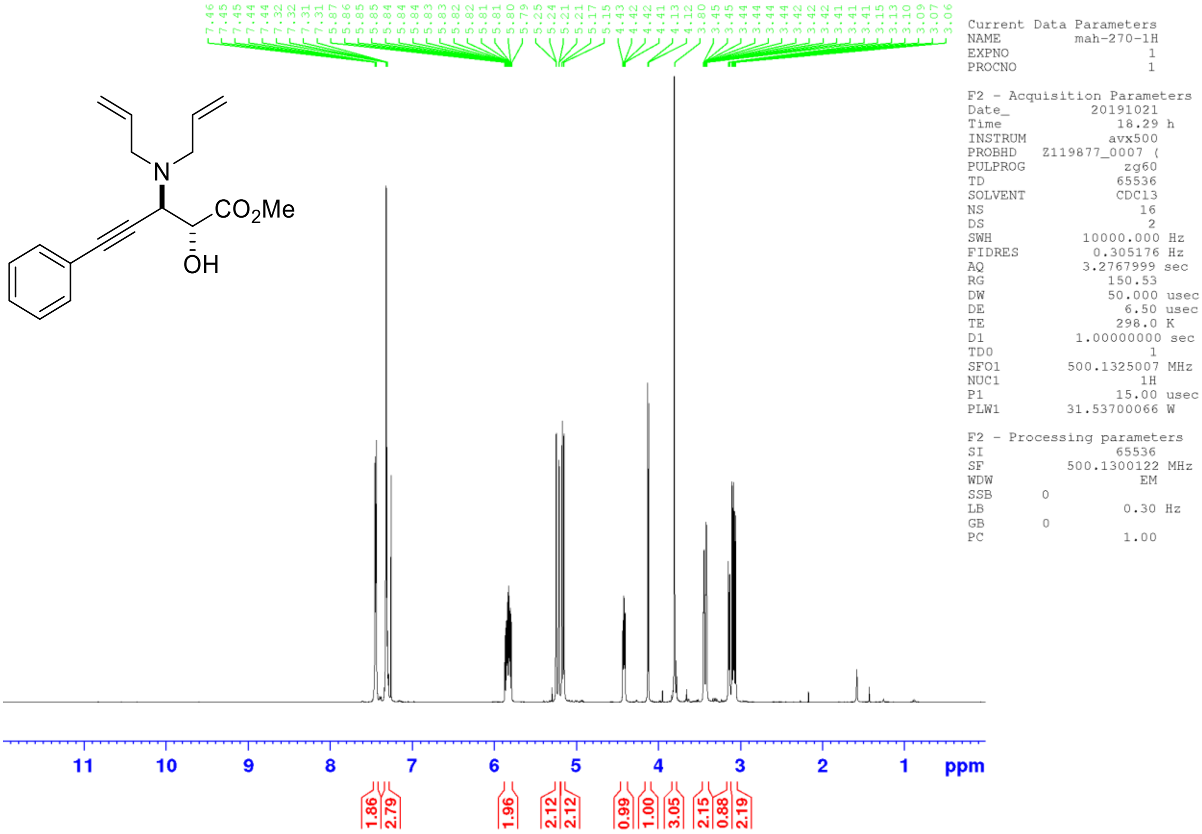


```
Current Data Parameters
NAME            mah-270-1H
EXPNO                  1
PROCNO                 1

F2 - Acquisition Parameters
Date_           20191021
Time               18.29 h
INSTRUM           avx500
PROBHD    Z119877_0007 (
PULPROG            zg60
TD                65536
SOLVENT            CDCl3
NS                   16
DS                    2
SWH           10000.000 Hz
FIDRES         0.305176 Hz
AQ            3.2767999 sec
RG               150.53
DW               50.000 usec
DE                 6.50 usec
TE                298.0 K
D1           1.00000000 sec
TD0                   1
SFO1        500.1325007 MHz
NUC1                 1H
P1                15.00 usec
PLW1        31.53700066 W

F2 - Processing parameters
SI                65536
SF          500.1300122 MHz
WDW                  EM
SSB      0
LB                 0.30 Hz
GB       0
PC                 1.00
```

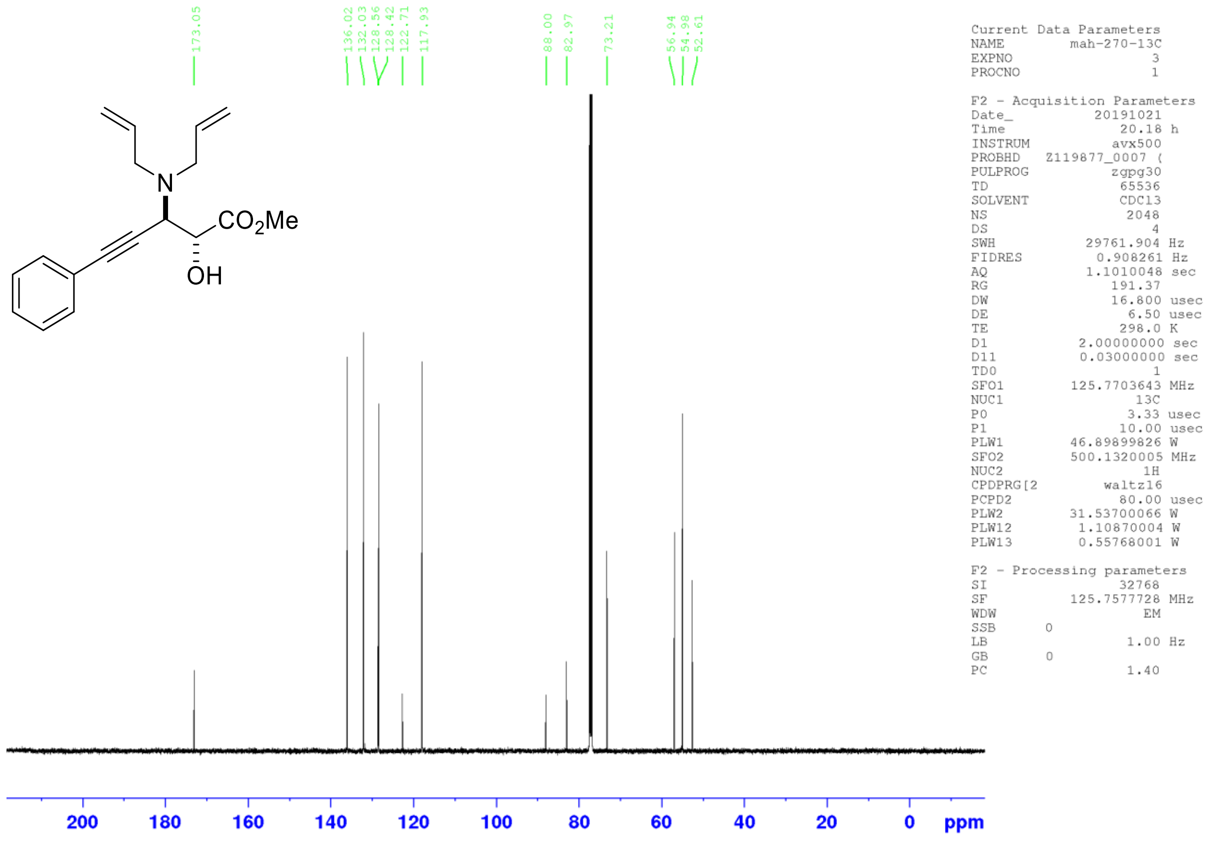
173.05
136.02
132.03
128.56
128.42
122.71
117.93
88.00
82.97
73.21
56.94
54.98
52.61
N
CO_2Me
OH
Current Data Parameters
NAME mah-270-13C
EXPNO 3
PROCNO 1
F2 - Acquisition Parameters
Date_ 20191021
Time 20.18 h
INSTRUM avx500
PROBHD Z119877_0007 (
PULPROG zgpg30
TD 65536
SOLVENT CDCl3
NS 2048
DS 4
SWH 29761.904 Hz
FIDRES 0.908261 Hz
AQ 1.1010048 sec
RG 191.37
DW 16.800 usec
DE 6.50 usec
TE 298.0 K
D1 2.00000000 sec
D11 0.03000000 sec
TD0 1
SFO1 125.7703643 MHz
NUC1 13C
P0 3.33 usec
P1 10.00 usec
PLW1 46.89899826 W
SFO2 500.1320005 MHz
NUC2 1H
CPDPRG[2 waltz16
PCPD2 80.00 usec
PLW2 31.53700066 W
PLW12 1.10870004 W
PLW13 0.55768001 W
F2 - Processing parameters
SI 32768
SF 125.7577728 MHz
WDW EM
SSB 0
LB 1.00 Hz
GB 0
PC 1.40
200 180 160 140 120 100 80 60 40 20 0 ppm

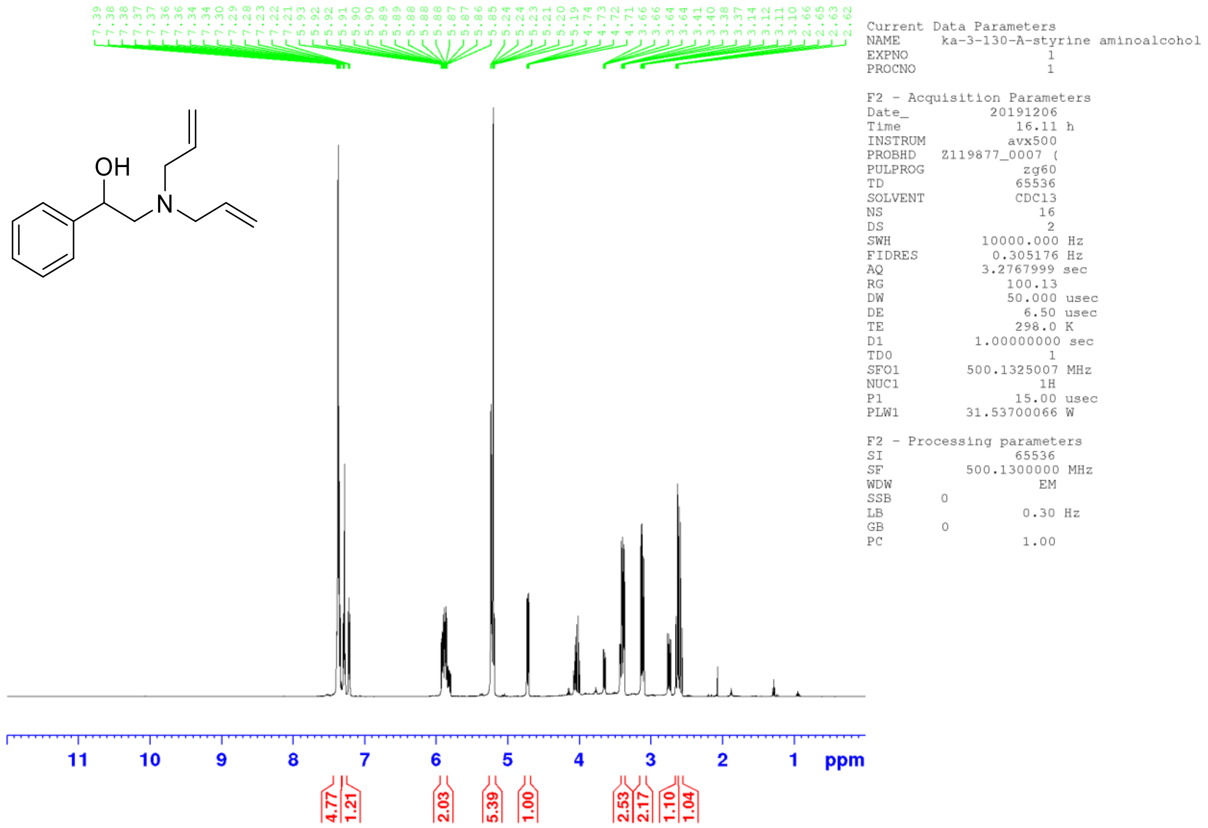
7.39 7.38 7.38 7.37 7.37 7.36 7.36 7.34 7.34 7.30 7.29 7.28 7.23 7.22 7.21 5.93 5.92 5.91 5.90 5.90 5.89 5.89 5.88 5.88 5.87 5.87 5.86 5.85 5.24 5.24 5.23 5.21 5.20 5.19 4.74 4.73 4.72 4.71 3.66 3.66 3.64 3.64 3.41 3.40 3.38 3.37 3.14 3.12 3.11 3.10 2.66 2.65 2.63 2.62
OH
N
Current Data Parameters
NAME ka-3-130-A-styrine aminoalcohol
EXPNO 1
PROCNO 1
F2 - Acquisition Parameters
Date_ 20191206
Time 16.11 h
INSTRUM avx500
PROBHD Z119877_0007 (
PULPROG zg60
TD 65536
SOLVENT CDCl3
NS 16
DS 2
SWH 10000.000 Hz
FIDRES 0.305176 Hz
AQ 3.2767999 sec
RG 100.13
DW 50.000 usec
DE 6.50 usec
TE 298.0 K
D1 1.00000000 sec
TD0 1
SFO1 500.1325007 MHz
NUC1 1H
P1 15.00 usec
PLW1 31.53700066 W
F2 - Processing parameters
SI 65536
SF 500.1300000 MHz
WDW EM
SSB 0
LB 0.30 Hz
GB 0
PC 1.00
11 10 9 8 7 6 5 4 3 2 1 ppm
4.77 1.21 2.03 5.39 1.00 2.53 2.17 1.10 1.04

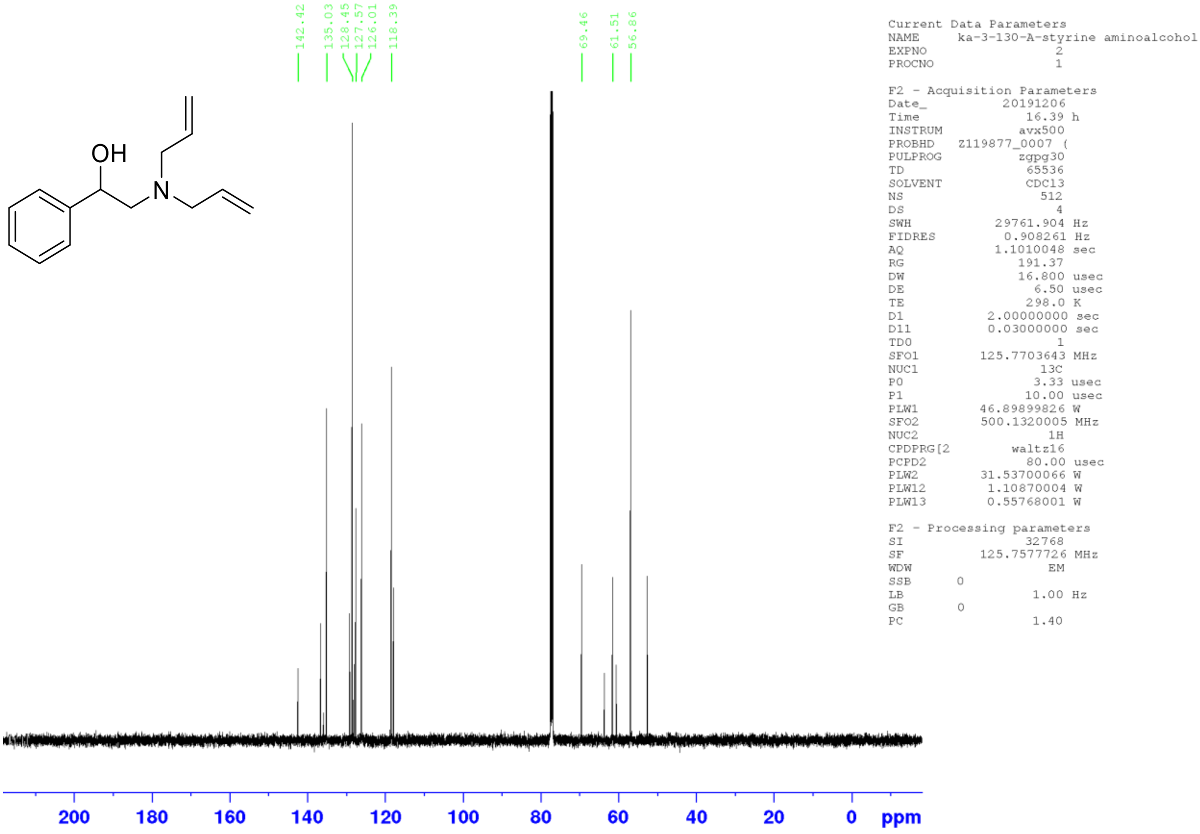

Current Data Parameters
NAME ka-3-130-A-styrine aminoalcohol
EXPNO 2
PROCNO 1

F2 - Acquisition Parameters
Date_ 20191206
Time 16.39 h
INSTRUM avx500
PROBHD Z119877_0007 (
PULPROG zgpg30
TD 65536
SOLVENT CDCl3
NS 512
DS 4
SWH 29761.904 Hz
FIDRES 0.908261 Hz
AQ 1.1010048 sec
RG 191.37
DW 16.800 usec
DE 6.50 usec
TE 298.0 K
D1 2.00000000 sec
D11 0.03000000 sec
TD0 1
SFO1 125.7703643 MHz
NUC1 13C
P0 3.33 usec
P1 10.00 usec
PLW1 46.89899826 W
SFO2 500.1320005 MHz
NUC2 1H
CPDPRG[2 waltz16
PCPD2 80.00 usec
PLW2 31.53700066 W
PLW12 1.10870004 W
PLW13 0.55768001 W

F2 - Processing parameters
SI 32768
SF 125.7577726 MHz
WDW EM
SSB 0
LB 1.00 Hz
GB 0
PC 1.40

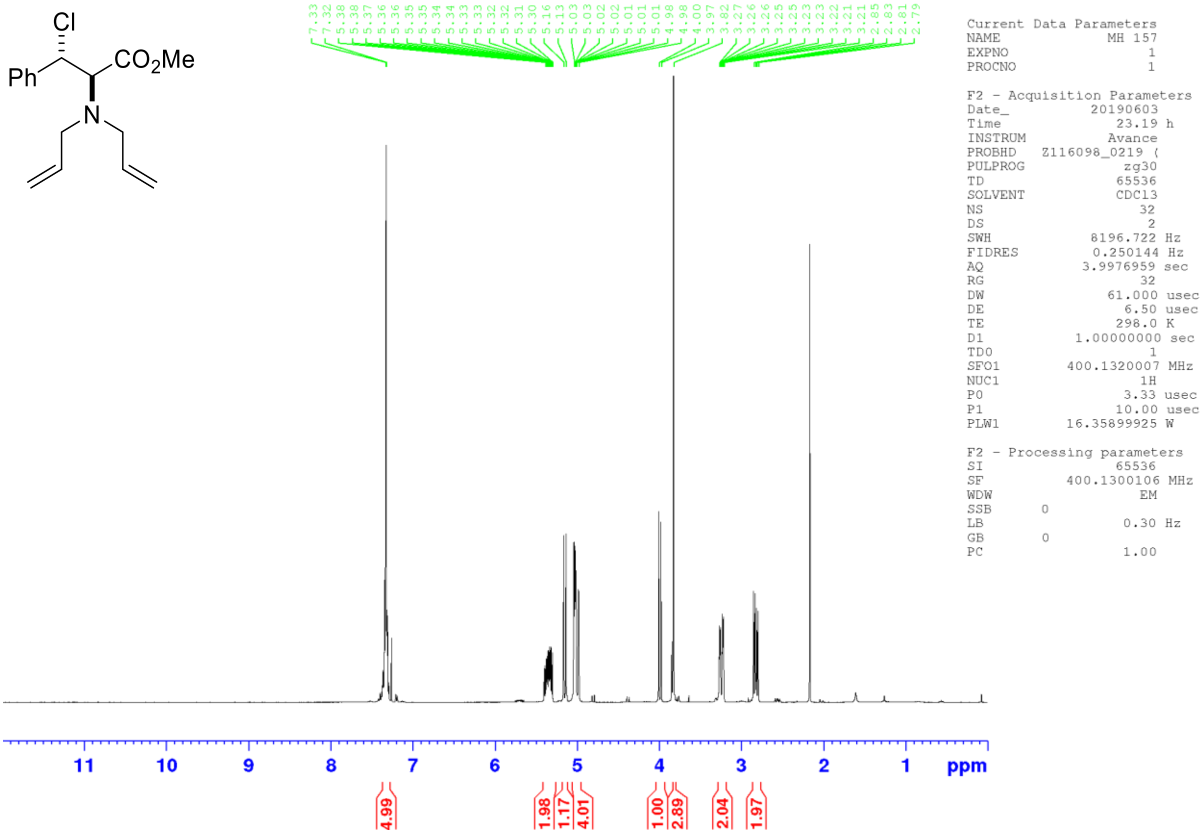

Current Data Parameters
NAME MH 157
EXPNO 1
PROCNO 1

F2 - Acquisition Parameters
Date_ 20190603
Time 23.19 h
INSTRUM Avance
PROBHD Z116098_0219 (
PULPROG zg30
TD 65536
SOLVENT CDCl3
NS 32
DS 2
SWH 8196.722 Hz
FIDRES 0.250144 Hz
AQ 3.9976959 sec
RG 32
DW 61.000 usec
DE 6.50 usec
TE 298.0 K
D1 1.00000000 sec
TD0 1
SFO1 400.1320007 MHz
NUC1 1H
P0 3.33 usec
P1 10.00 usec
PLW1 16.35899925 W

F2 - Processing parameters
SI 65536
SF 400.1300106 MHz
WDW EM
SSB 0
LB 0.30 Hz
GB 0
PC 1.00

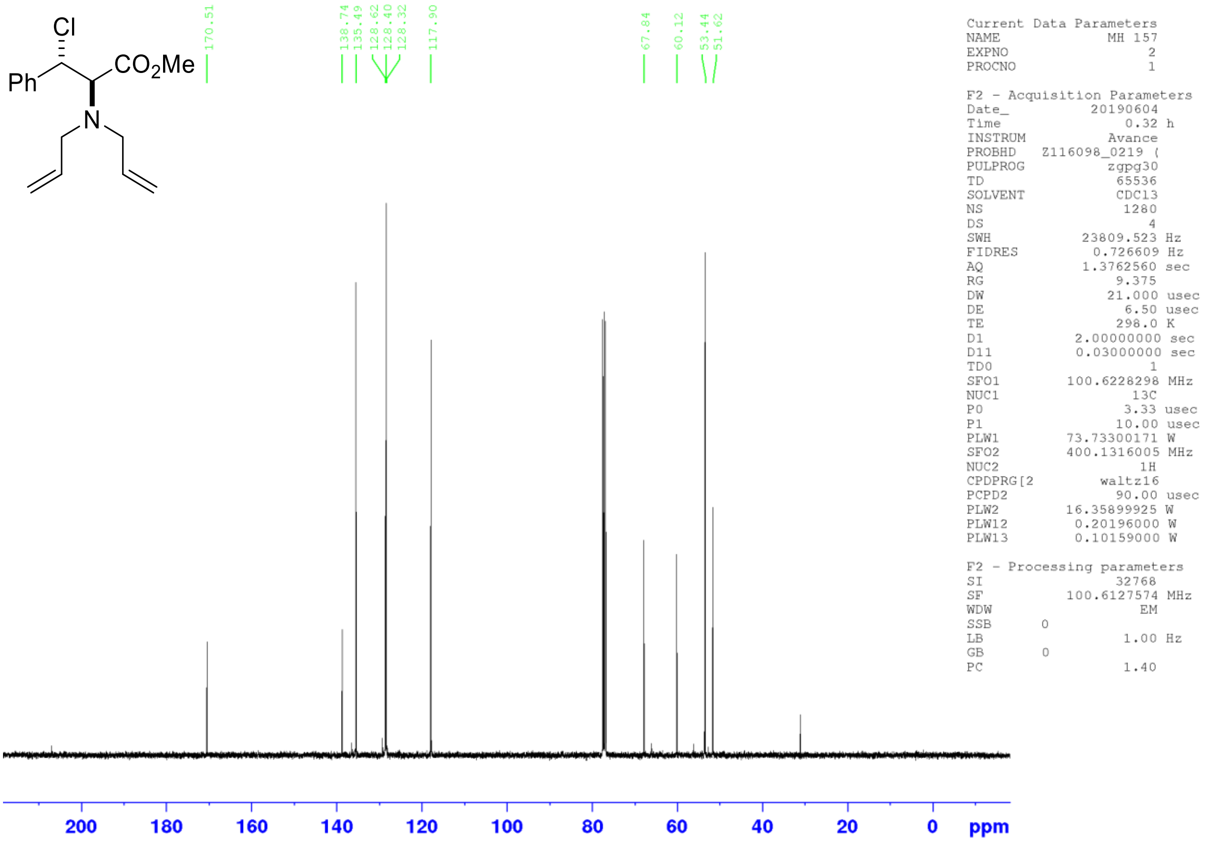
Cl
Ph
CO2Me
N
170.51
138.74
135.49
128.62
128.40
128.32
117.90
67.84
60.12
53.44
51.62
Current Data Parameters
NAME MH 157
EXPNO 2
PROCNO 1
F2 - Acquisition Parameters
Date_ 20190604
Time 0.32 h
INSTRUM Avance
PROBHD Z116098_0219 (
PULPROG zgpg30
TD 65536
SOLVENT CDCl3
NS 1280
DS 4
SWH 23809.523 Hz
FIDRES 0.726609 Hz
AQ 1.3762560 sec
RG 9.375
DW 21.000 usec
DE 6.50 usec
TE 298.0 K
D1 2.00000000 sec
D11 0.03000000 sec
TD0 1
SFO1 100.6228298 MHz
NUC1 13C
P0 3.33 usec
P1 10.00 usec
PLW1 73.73300171 W
SFO2 400.1316005 MHz
NUC2 1H
CPDPRG[2 waltz16
PCPD2 90.00 usec
PLW2 16.35899925 W
PLW12 0.20196000 W
PLW13 0.10159000 W
F2 - Processing parameters
SI 32768
SF 100.6127574 MHz
WDW EM
SSB 0
LB 1.00 Hz
GB 0
PC 1.40
200
180
160
140
120
100
80
60
40
20
0
ppm

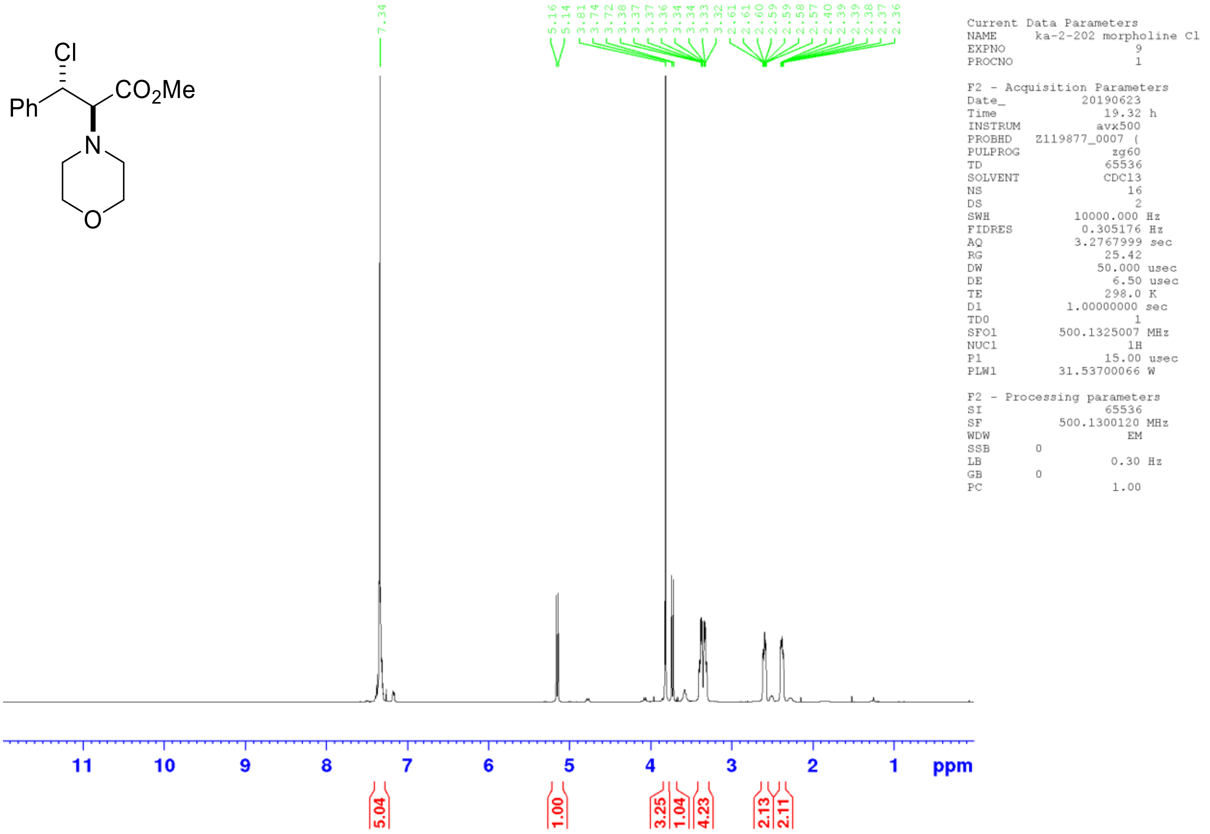
Cl
Ph
CO2Me
N
O
7.34
5.16
5.14
3.81
3.74
3.72
3.38
3.37
3.36
3.34
3.34
3.33
3.32
2.61
2.61
2.60
2.59
2.59
2.58
2.57
2.40
2.39
2.39
2.38
2.37
2.36
Current Data Parameters
NAME ka-2-202 morpholine Cl
EXPNO 9
PROCNO 1
F2 - Acquisition Parameters
Date_ 20190623
Time 19.32 h
INSTRUM avx500
PROBHD Z119877_0007 (
PULPROG zg60
TD 65536
SOLVENT CDCl3
NS 16
DS 2
SWH 10000.000 Hz
FIDRES 0.305176 Hz
AQ 3.2767999 sec
RG 25.42
DW 50.000 usec
DE 6.50 usec
TE 298.0 K
D1 1.00000000 sec
TD0 1
SFO1 500.1325007 MHz
NUC1 1H
P1 15.00 usec
PLW1 31.53700066 W
F2 - Processing parameters
SI 65536
SF 500.1300120 MHz
WDW EM
SSB 0
LB 0.30 Hz
GB 0
PC 1.00
11
10
9
8
7
6
5
4
3
2
1
ppm
5.04
1.00
3.25
1.04
4.23
2.13
2.11

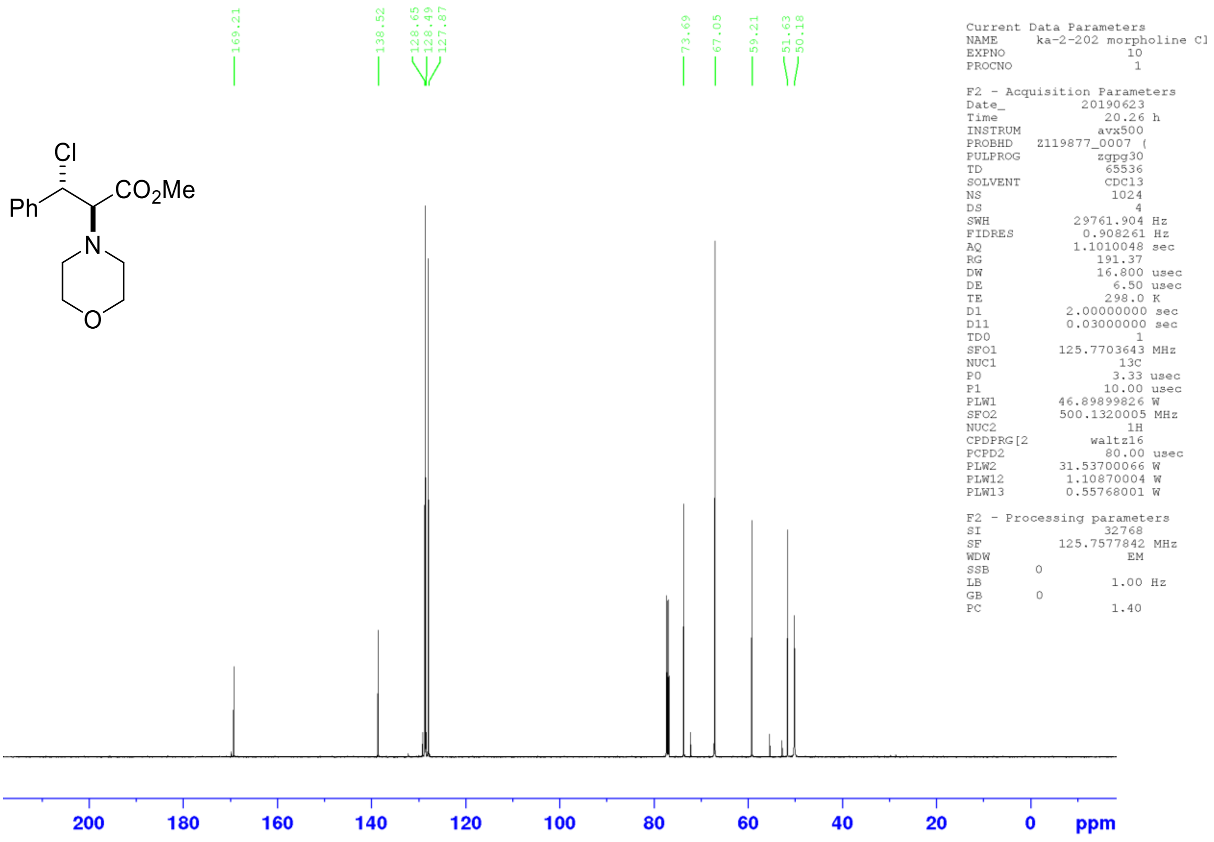
Cl
Ph
CO2Me
N
O
169.21
138.52
128.65
128.49
127.87
73.69
67.05
59.21
51.63
50.18
Current Data Parameters
NAME ka-2-202 morpholine Cl
EXPNO 10
PROCNO 1
F2 - Acquisition Parameters
Date_ 20190623
Time 20.26 h
INSTRUM avx500
PROBHD Z119877_0007 (
PULPROG zgpg30
TD 65536
SOLVENT CDCl3
NS 1024
DS 4
SWH 29761.904 Hz
FIDRES 0.908261 Hz
AQ 1.1010048 sec
RG 191.37
DW 16.800 usec
DE 6.50 usec
TE 298.0 K
D1 2.00000000 sec
D11 0.03000000 sec
TD0 1
SFO1 125.7703643 MHz
NUC1 13C
P0 3.33 usec
P1 10.00 usec
PLW1 46.89899826 W
SFO2 500.1320005 MHz
NUC2 1H
CPDPRG[2 waltz16
PCPD2 80.00 usec
PLW2 31.53700066 W
PLW12 1.10870004 W
PLW13 0.55768001 W
F2 - Processing parameters
SI 32768
SF 125.7577842 MHz
WDW EM
SSB 0
LB 1.00 Hz
GB 0
PC 1.40
200
180
160
140
120
100
80
60
40
20
0
ppm

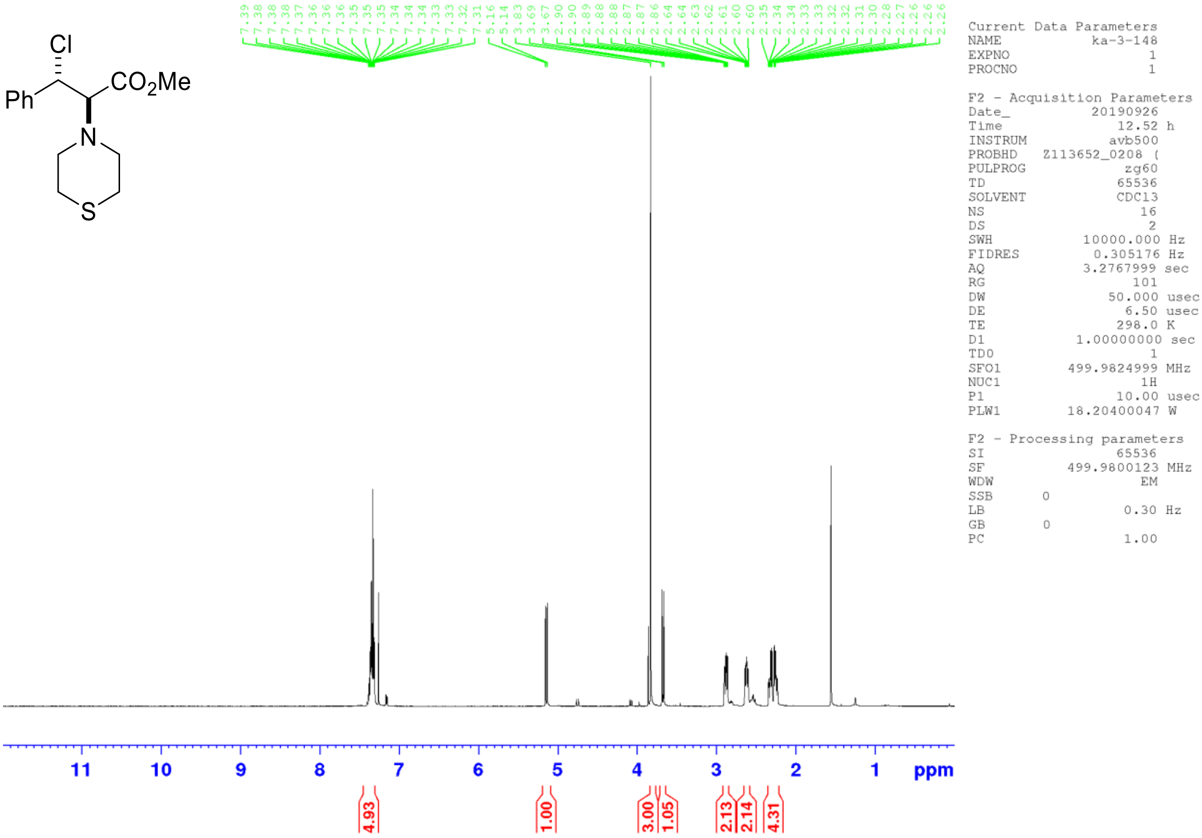
Cl
Ph
CO2Me
N
S
7.39
7.38
7.38
7.38
7.37
7.36
7.36
7.35
7.35
7.35
7.34
7.34
7.34
7.33
7.33
7.32
7.31
5.16
5.14
3.83
3.69
3.67
2.90
2.90
2.89
2.88
2.88
2.87
2.86
2.64
2.64
2.63
2.62
2.61
2.60
2.60
2.35
2.34
2.34
2.33
2.32
2.32
2.31
2.30
2.28
2.27
2.26
2.26
2.26
Current Data Parameters
NAME ka-3-148
EXPNO 1
PROCNO 1
F2 - Acquisition Parameters
Date_ 20190926
Time 12.52 h
INSTRUM avb500
PROBHD Z113652_0208 (
PULPROG zg60
TD 65536
SOLVENT CDCl3
NS 16
DS 2
SWH 10000.000 Hz
FIDRES 0.305176 Hz
AQ 3.2767999 sec
RG 101
DW 50.000 usec
DE 6.50 usec
TE 298.0 K
D1 1.00000000 sec
TD0 1
SFO1 499.9824999 MHz
NUC1 1H
P1 10.00 usec
PLW1 18.20400047 W
F2 - Processing parameters
SI 65536
SF 499.9800123 MHz
WDW EM
SSB 0
LB 0.30 Hz
GB 0
PC 1.00
11
10
9
8
7
6
5
4
3
2
1
ppm
4.93
1.00
3.00
1.05
2.13
2.14
4.31

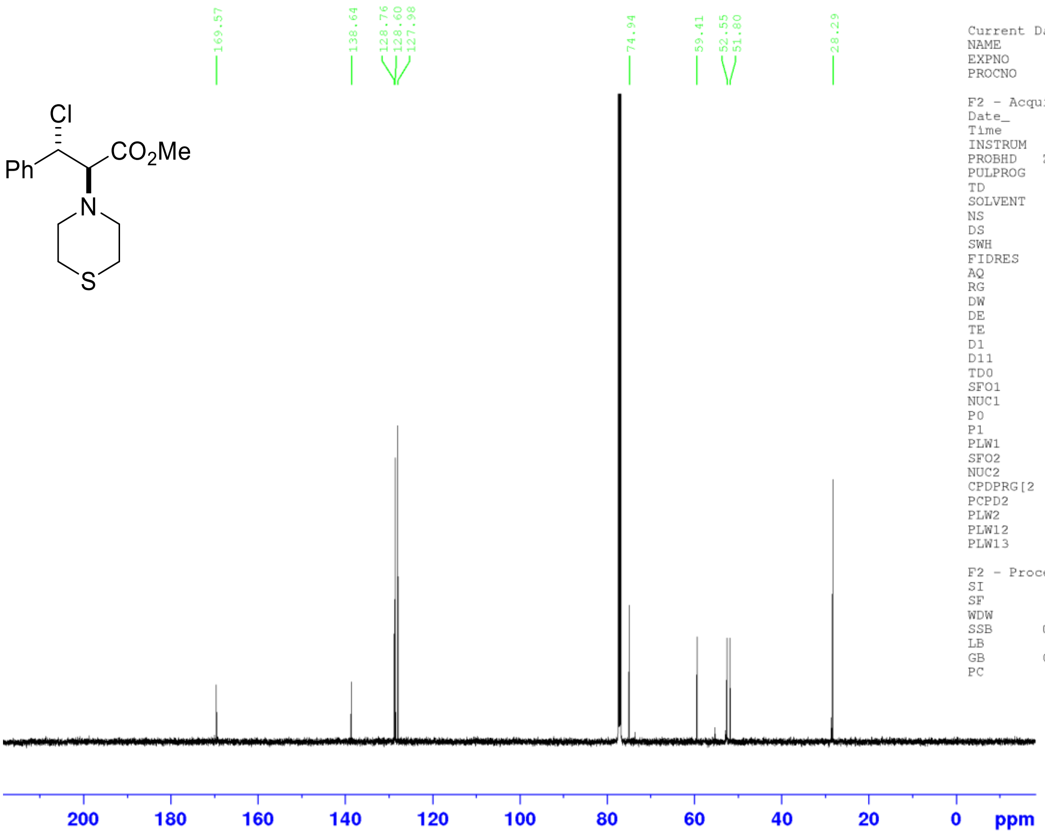

Current Data Parameters
NAME ka-3-148
EXPNO 2
PROCNO 1

F2 - Acquisition Parameters
Date_ 20190926
Time 13.47 h
INSTRUM avb500
PROBHD Z113652_0208 (
PULPROG zgpg30
TD 65536
SOLVENT CDCl3
NS 1024
DS 4
SWH 29761.904 Hz
FIDRES 0.908261 Hz
AQ 1.1010048 sec
RG 203
DW 16.800 usec
DE 6.50 usec
TE 298.2 K
D1 2.00000000 sec
D11 0.03000000 sec
TD0 1
SFO1 125.7326430 MHz
NUC1 13C
P0 3.33 usec
P1 10.00 usec
PLW1 81.90399933 W
SFO2 499.9819999 MHz
NUC2 1H
CPDPRG[2 waltz16
PCPD2 80.00 usec
PLW2 18.20400047 W
PLW12 0.28444001 W
PLW13 0.14307000 W

F2 - Processing parameters
SI 32768
SF 125.7200550 MHz
WDW EM
SSB 0
LB 1.00 Hz
GB 0
PC 1.40

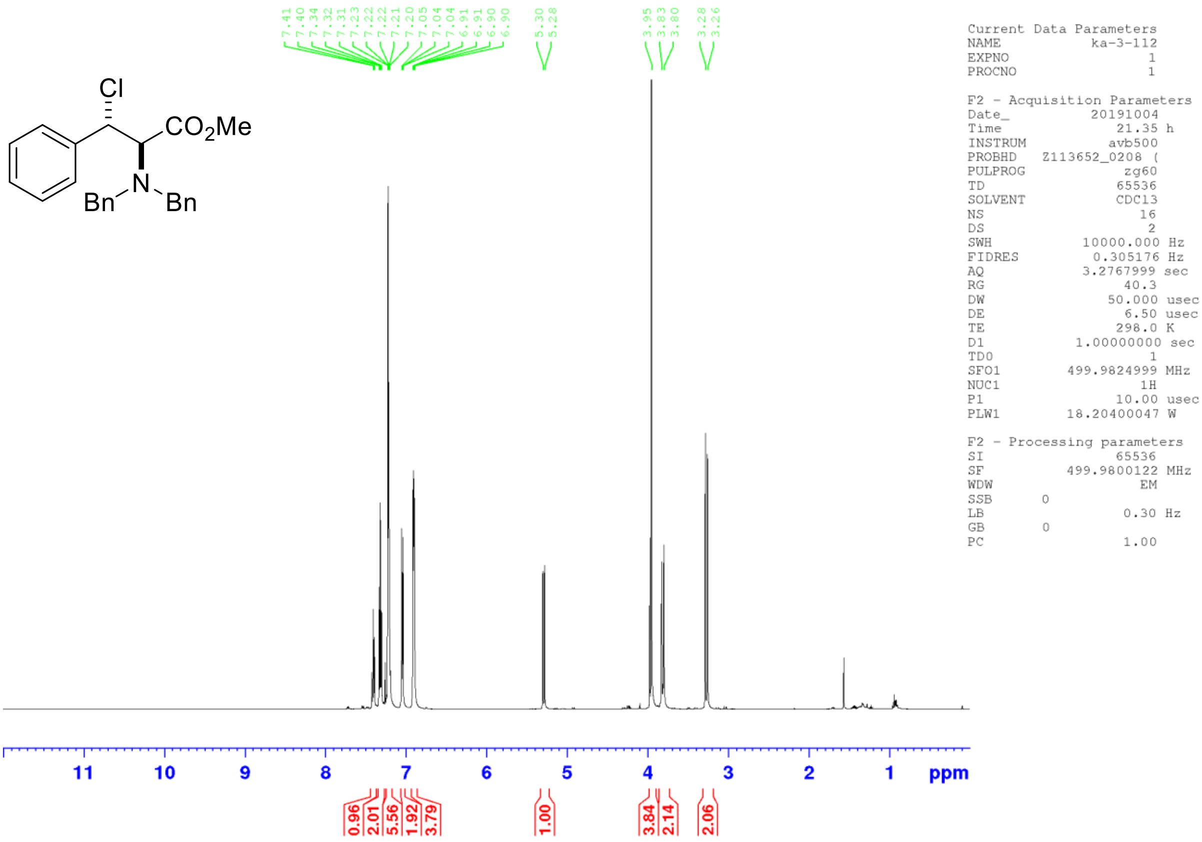

Current Data Parameters
NAME ka-3-112
EXPNO 1
PROCNO 1

F2 - Acquisition Parameters
Date_ 20191004
Time 21.35 h
INSTRUM avb500
PROBHD Z113652_0208 (
PULPROG zg60
TD 65536
SOLVENT CDCl3
NS 16
DS 2
SWH 10000.000 Hz
FIDRES 0.305176 Hz
AQ 3.2767999 sec
RG 40.3
DW 50.000 usec
DE 6.50 usec
TE 298.0 K
D1 1.00000000 sec
TD0 1
SFO1 499.9824999 MHz
NUC1 1H
P1 10.00 usec
PLW1 18.20400047 W

F2 - Processing parameters
SI 65536
SF 499.9800122 MHz
WDW EM
SSB 0
LB 0.30 Hz
GB 0
PC 1.00

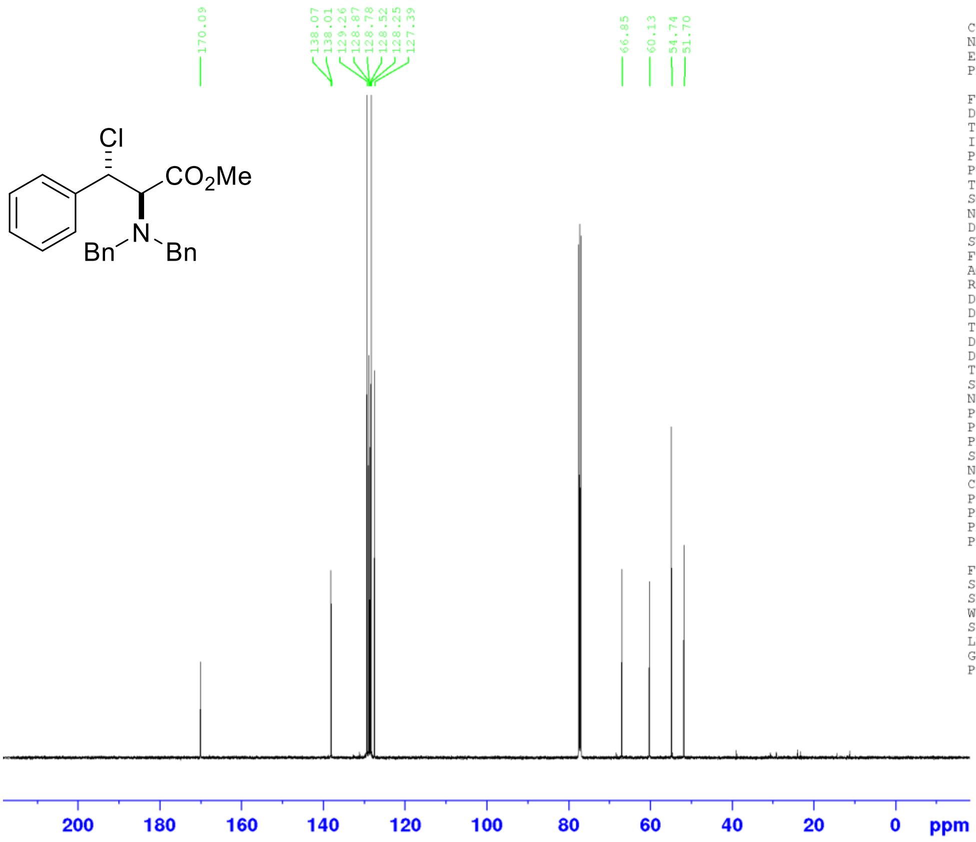


```
Current Data Parameters
NAME              ka-3-112
EXPNO                    2
PROCNO                   1

F2 - Acquisition Parameters
Date_             20191004
Time               22.30 h
INSTRUM             avb500
PROBHD   Z113652_0208 (
PULPROG            zgpg30
TD                  65536
SOLVENT             CDCl3
NS                   1024
DS                      4
SWH             29761.904 Hz
FIDRES           0.908261 Hz
AQ              1.1010048 sec
RG                    203
DW                 16.800 usec
DE                   6.50 usec
TE                  298.0 K
D1             2.00000000 sec
D11            0.03000000 sec
TD0                     1
SFO1          125.7326430 MHz
NUC1                  13C
P0                   3.33 usec
P1                  10.00 usec
PLW1          81.90399933 W
SFO2          499.9819999 MHz
NUC2                   1H
CPDPRG[2          waltz16
PCPD2               80.00 usec
PLW2          18.20400047 W
PLW12          0.28444001 W
PLW13          0.14307000 W

F2 - Processing parameters
SI                  32768
SF            125.7200579 MHz
WDW                    EM
SSB      0
LB                   1.00 Hz
GB       0
PC                   1.40
```

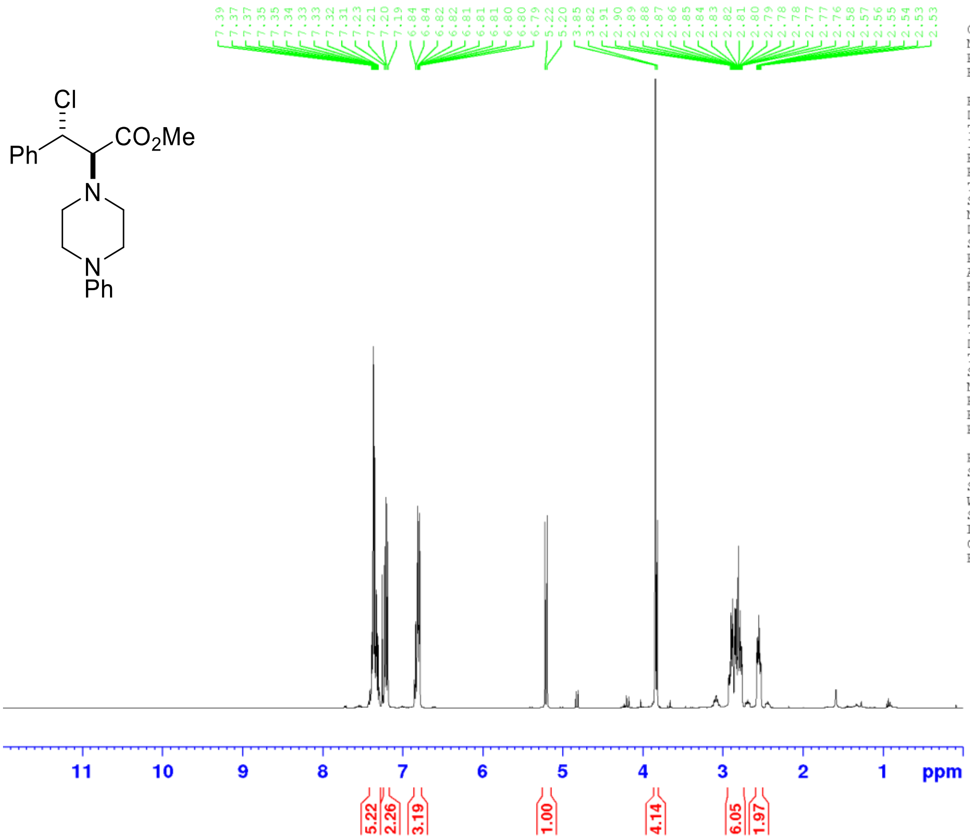


```
Current Data Parameters
NAME                MH-261
EXPNO                    1
PROCNO                   1

F2 - Acquisition Parameters
Date_             20191002
Time               18.53 h
INSTRUM             Avance
PROBHD   Z116098_0219 (
PULPROG              zg30
TD                  65536
SOLVENT             CDCl3
NS                     16
DS                      2
SWH              7812.500 Hz
FIDRES           0.238419 Hz
AQ              4.1943040 sec
RG                    101
DW                 64.000 usec
DE                   6.50 usec
TE                  298.0 K
D1             1.00000000 sec
TD0                     1
SFO1          400.1320007 MHz
NUC1                   1H
P0                   3.33 usec
P1                  10.00 usec
PLW1          16.35899925 W

F2 - Processing parameters
SI                  65536
SF            400.1300098 MHz
WDW                    EM
SSB      0
LB                   0.30 Hz
GB       0
PC                   1.00
```

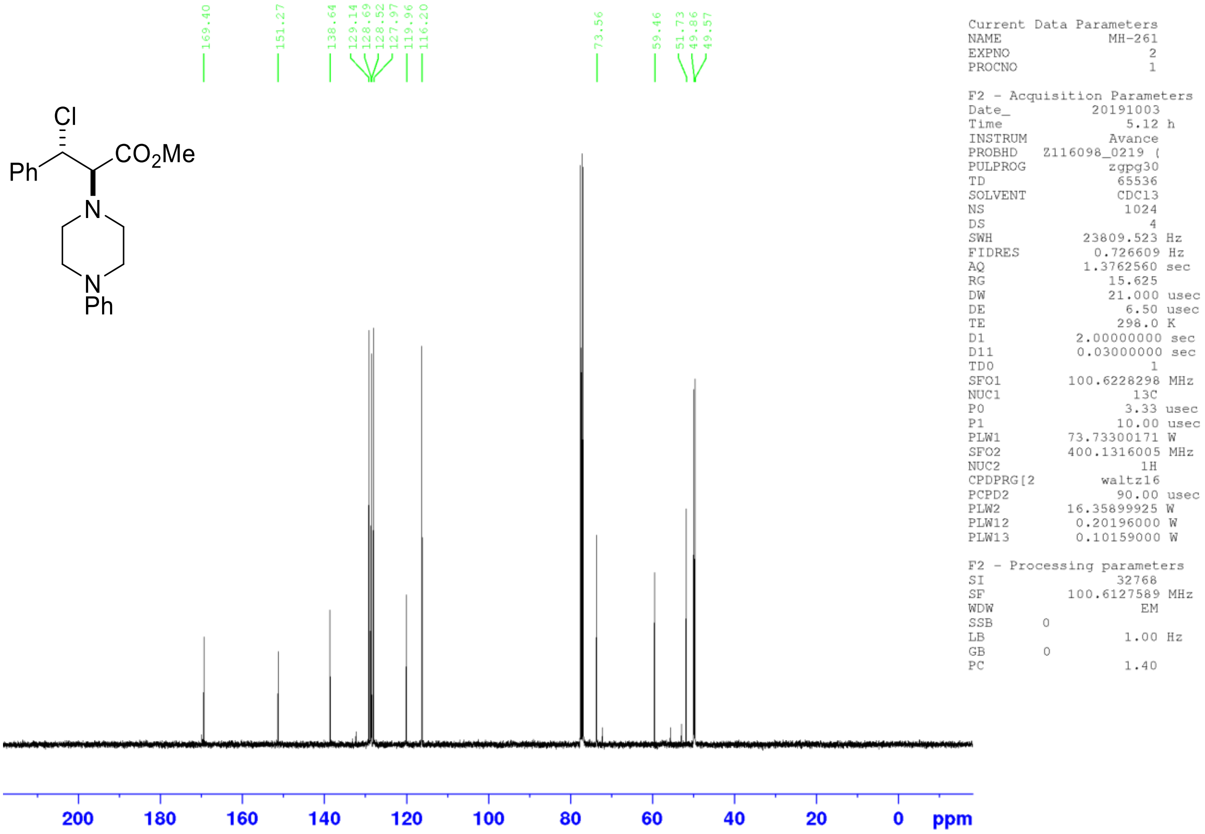
Cl
Ph
CO2Me
N
N
Ph
169.40
151.27
138.64
129.14
128.69
128.52
127.97
119.96
116.20
73.56
59.46
51.73
49.86
49.57
Current Data Parameters
NAME MH-261
EXPNO 2
PROCNO 1
F2 - Acquisition Parameters
Date_ 20191003
Time 5.12 h
INSTRUM Avance
PROBHD Z116098_0219 (
PULPROG zgpg30
TD 65536
SOLVENT CDCl3
NS 1024
DS 4
SWH 23809.523 Hz
FIDRES 0.726609 Hz
AQ 1.3762560 sec
RG 15.625
DW 21.000 usec
DE 6.50 usec
TE 298.0 K
D1 2.00000000 sec
D11 0.03000000 sec
TD0 1
SFO1 100.6228298 MHz
NUC1 13C
P0 3.33 usec
P1 10.00 usec
PLW1 73.73300171 W
SFO2 400.1316005 MHz
NUC2 1H
CPDPRG[2 waltz16
PCPD2 90.00 usec
PLW2 16.35899925 W
PLW12 0.20196000 W
PLW13 0.10159000 W
F2 - Processing parameters
SI 32768
SF 100.6127589 MHz
WDW EM
SSB 0
LB 1.00 Hz
GB 0
PC 1.40
200 180 160 140 120 100 80 60 40 20 0 ppm

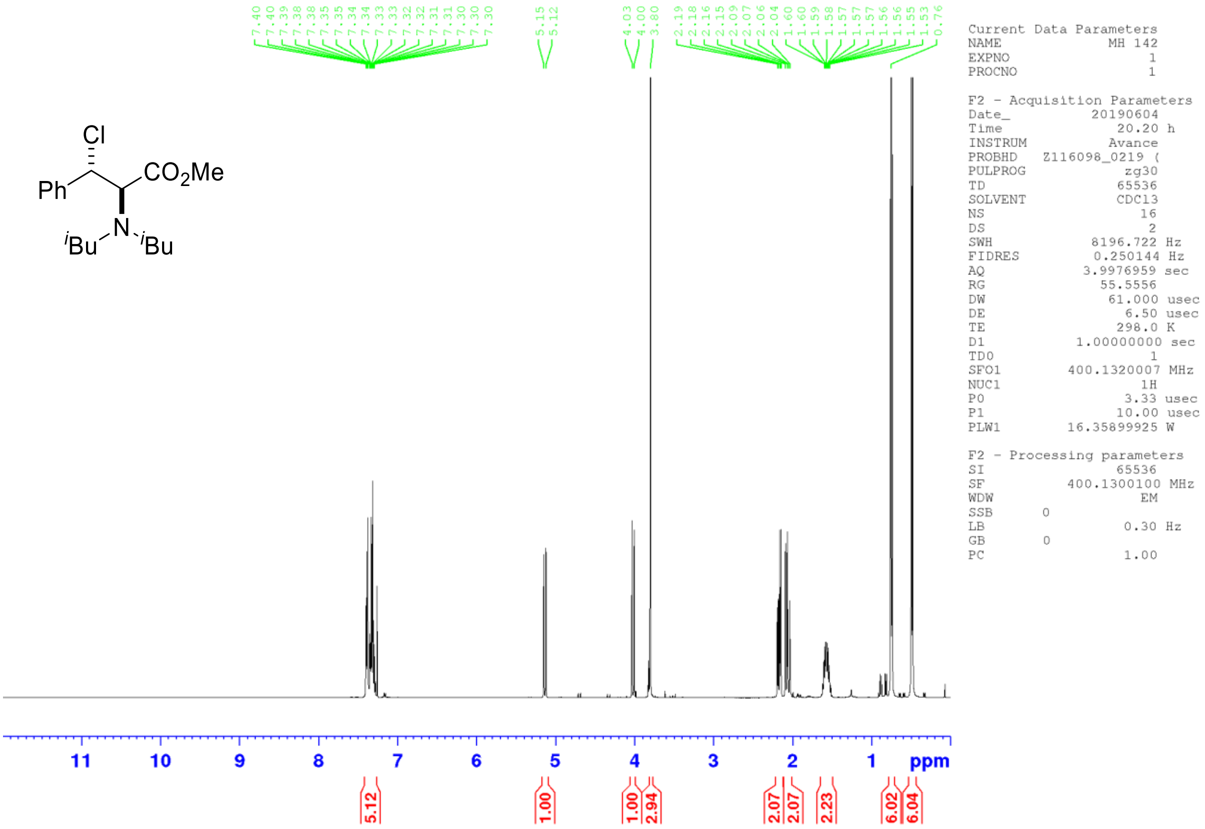
Cl
Ph
CO2Me
N
iBu
iBu
7.40
7.40
7.39
7.38
7.38
7.35
7.35
7.34
7.34
7.33
7.33
7.32
7.32
7.31
7.31
7.30
7.30
7.30
5.15
5.12
4.03
4.00
3.80
2.19
2.18
2.16
2.15
2.09
2.07
2.06
2.04
1.60
1.59
1.58
1.57
1.57
1.56
1.56
1.55
1.53
0.76
Current Data Parameters
NAME MH 142
EXPNO 1
PROCNO 1
F2 - Acquisition Parameters
Date_ 20190604
Time 20.20 h
INSTRUM Avance
PROBHD Z116098_0219 (
PULPROG zg30
TD 65536
SOLVENT CDCl3
NS 16
DS 2
SWH 8196.722 Hz
FIDRES 0.250144 Hz
AQ 3.9976959 sec
RG 55.5556
DW 61.000 usec
DE 6.50 usec
TE 298.0 K
D1 1.00000000 sec
TD0 1
SFO1 400.1320007 MHz
NUC1 1H
P0 3.33 usec
P1 10.00 usec
PLW1 16.35899925 W
F2 - Processing parameters
SI 65536
SF 400.1300100 MHz
WDW EM
SSB 0
LB 0.30 Hz
GB 0
PC 1.00
11 10 9 8 7 6 5 4 3 2 1 ppm
5.12
1.00
1.00
2.94
2.07
2.07
2.23
6.02
6.04

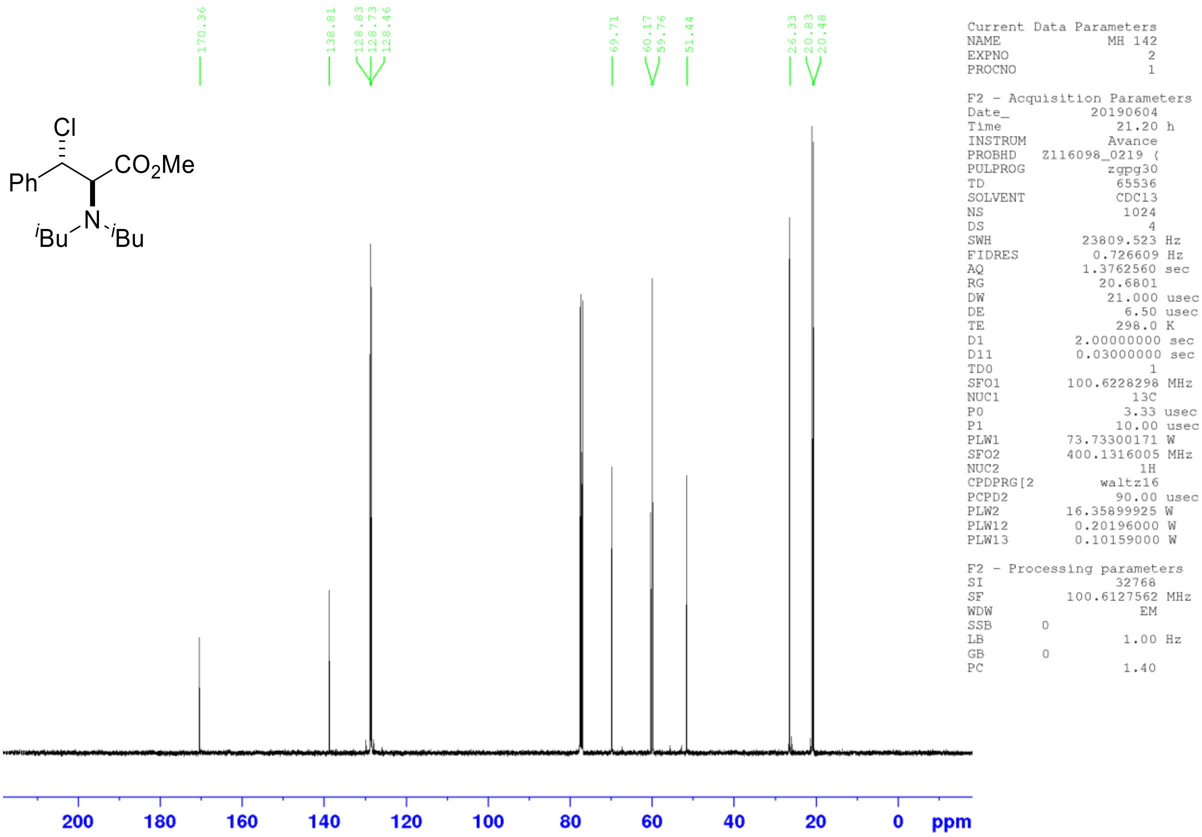
170.36
138.81
128.83
128.73
128.46
69.71
60.17
59.76
51.44
26.33
20.83
20.48
Cl
Ph
CO2Me
N
iBu
iBu
200 180 160 140 120 100 80 60 40 20 0 ppm
Current Data Parameters
NAME MH 142
EXPNO 2
PROCNO 1
F2 - Acquisition Parameters
Date_ 20190604
Time 21.20 h
INSTRUM Avance
PROBHD Z116098_0219 (
PULPROG zgpg30
TD 65536
SOLVENT CDCl3
NS 1024
DS 4
SWH 23809.523 Hz
FIDRES 0.726609 Hz
AQ 1.3762560 sec
RG 20.6801
DW 21.000 usec
DE 6.50 usec
TE 298.0 K
D1 2.00000000 sec
D11 0.03000000 sec
TD0 1
SFO1 100.6228298 MHz
NUC1 13C
P0 3.33 usec
P1 10.00 usec
PLW1 73.73300171 W
SFO2 400.1316005 MHz
NUC2 1H
CPDPRG[2 waltz16
PCPD2 90.00 usec
PLW2 16.35899925 W
PLW12 0.20196000 W
PLW13 0.10159000 W
F2 - Processing parameters
SI 32768
SF 100.6127562 MHz
WDW EM
SSB 0
LB 1.00 Hz
GB 0
PC 1.40

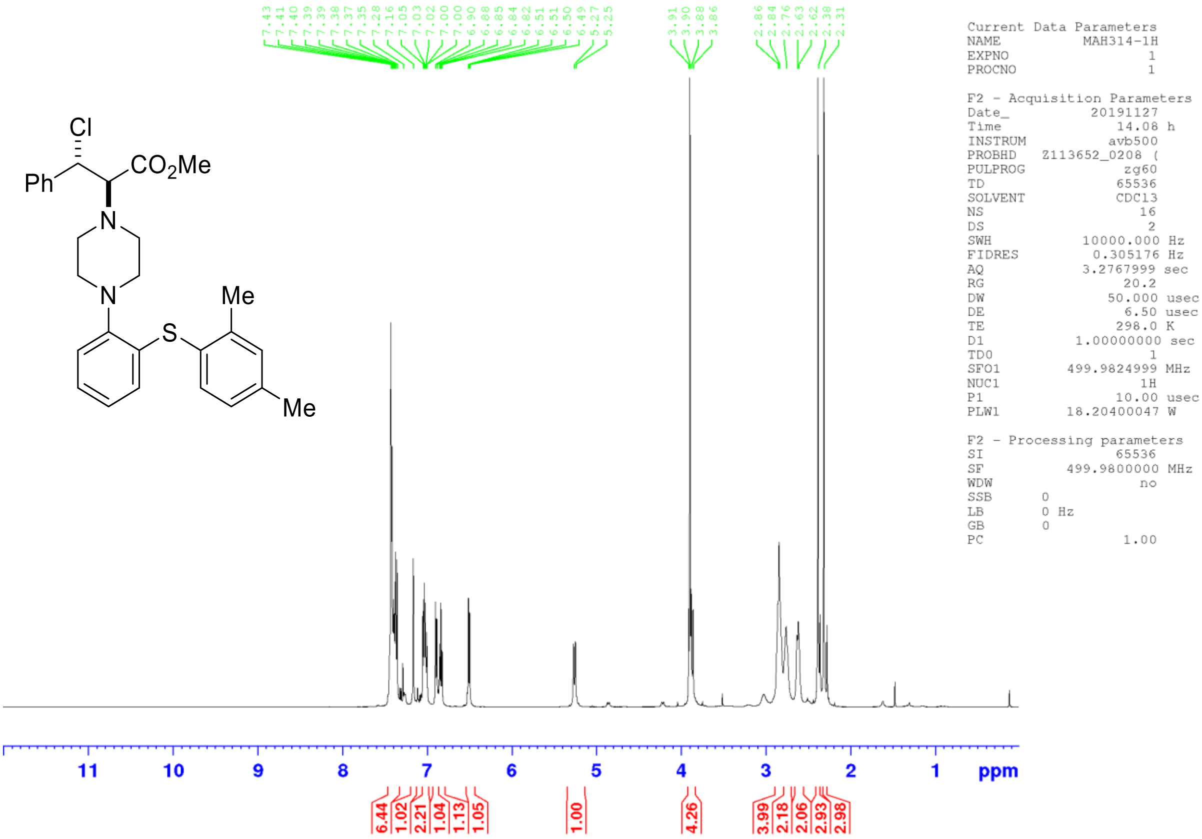
7.43
7.41
7.40
7.39
7.39
7.38
7.37
7.35
7.28
7.16
7.05
7.03
7.02
7.00
7.00
6.90
6.88
6.85
6.84
6.82
6.51
6.51
6.50
6.49
5.27
5.25
3.91
3.90
3.88
3.86
2.86
2.84
2.76
2.63
2.62
2.38
2.31
Cl
Ph
CO2Me
N
N
S
Me
Me
11 10 9 8 7 6 5 4 3 2 1 ppm
6.44
1.02
2.21
1.04
1.13
1.05
1.00
4.26
3.99
2.18
2.06
2.93
2.98
Current Data Parameters
NAME MAH314-1H
EXPNO 1
PROCNO 1
F2 - Acquisition Parameters
Date_ 20191127
Time 14.08 h
INSTRUM avb500
PROBHD Z113652_0208 (
PULPROG zg60
TD 65536
SOLVENT CDCl3
NS 16
DS 2
SWH 10000.000 Hz
FIDRES 0.305176 Hz
AQ 3.2767999 sec
RG 20.2
DW 50.000 usec
DE 6.50 usec
TE 298.0 K
D1 1.00000000 sec
TD0 1
SFO1 499.9824999 MHz
NUC1 1H
P1 10.00 usec
PLW1 18.20400047 W
F2 - Processing parameters
SI 65536
SF 499.9800000 MHz
WDW no
SSB 0
LB 0 Hz
GB 0
PC 1.00

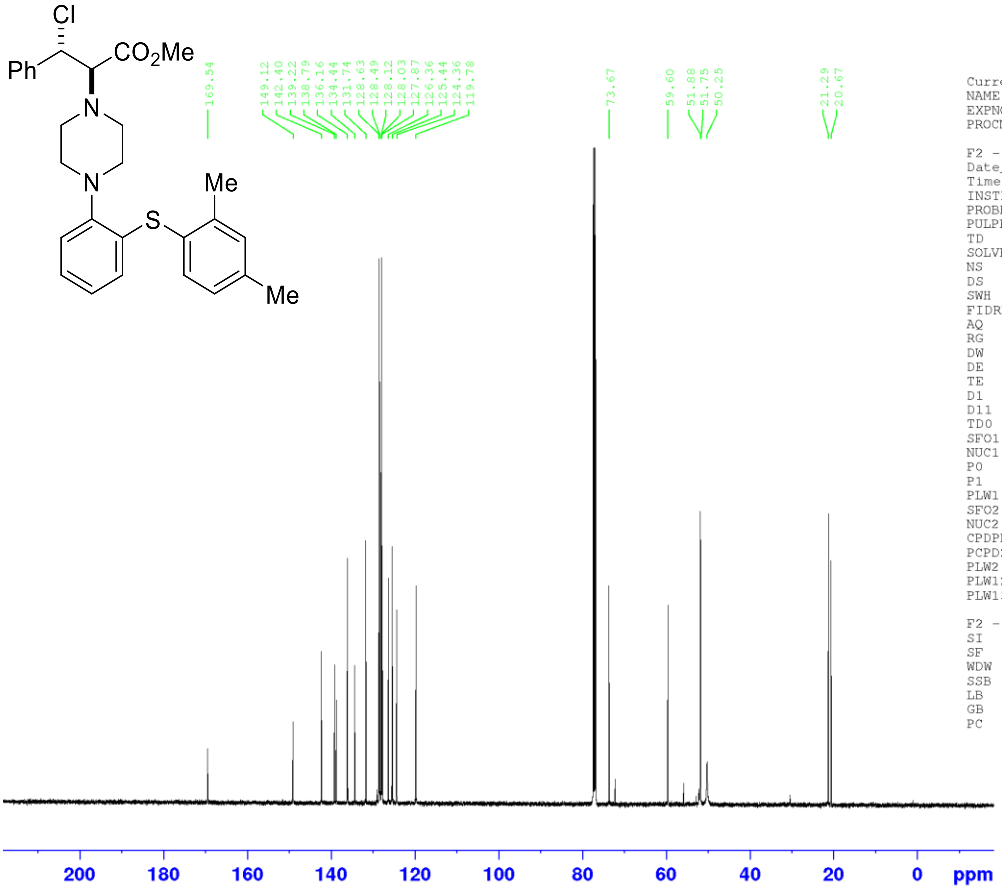


```
Current Data Parameters
NAME          MAH314-13C
EXPNO                  2
PROCNO                 1

F2 - Acquisition Parameters
Date_           20191127
Time               15.04 h
INSTRUM           avb500
PROBHD   Z113652_0208 (
PULPROG          zgpg30
TD                 65536
SOLVENT            CDCl3
NS                   935
DS                     4
SWH            29761.904 Hz
FIDRES          0.908261 Hz
AQ             1.1010048 sec
RG                   203
DW                16.800 usec
DE                  6.50 usec
TE                 298.2 K
D1            2.00000000 sec
D11           0.03000000 sec
TD0                    1
SFO1         125.7326430 MHz
NUC1                 13C
P0                  3.33 usec
P1                 10.00 usec
PLW1         81.90399933 W
SFO2         499.9819999 MHz
NUC2                  1H
CPDPRG[2         waltz16
PCPD2              80.00 usec
PLW2         18.20400047 W
PLW12         0.28444001 W
PLW13         0.14307000 W

F2 - Processing parameters
SI                 32768
SF           125.7200603 MHz
WDW                   EM
SSB      0
LB                  1.00 Hz
GB       0
PC                  1.40
```

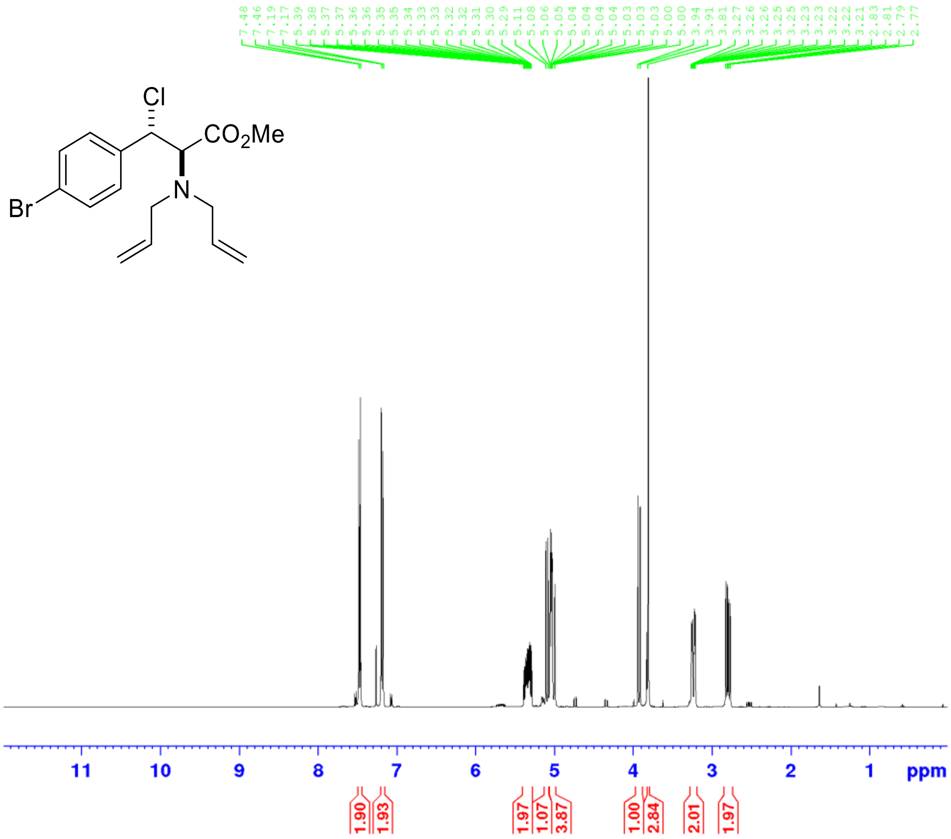


```
Current Data Parameters
NAME              MH 172
EXPNO                  1
PROCNO                 1

F2 - Acquisition Parameters
Date_           20190605
Time               19.19 h
INSTRUM          Avance
PROBHD   Z116098_0219 (
PULPROG             zg30
TD                 65536
SOLVENT            CDCl3
NS                    16
DS                     2
SWH             8196.722 Hz
FIDRES          0.250144 Hz
AQ             3.9976959 sec
RG                    32
DW                61.000 usec
DE                  6.50 usec
TE                 298.0 K
D1            1.00000000 sec
TD0                    1
SFO1         400.1320007 MHz
NUC1                  1H
P0                  3.33 usec
P1                 10.00 usec
PLW1         16.35899925 W

F2 - Processing parameters
SI                 65536
SF           400.1300098 MHz
WDW                   EM
SSB      0
LB                  0.30 Hz
GB       0
PC                  1.00
```

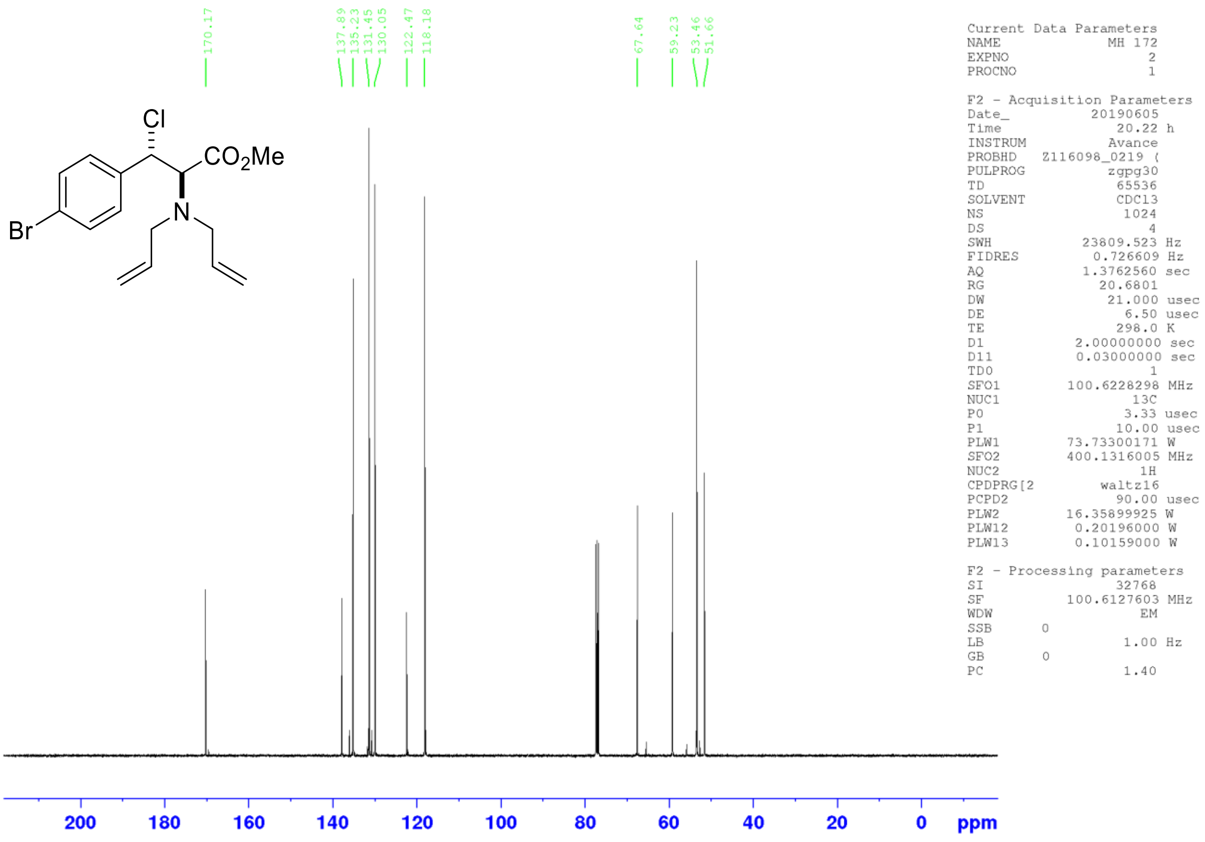
170.17
137.89
135.23
131.45
130.05
122.47
118.18
67.64
59.23
53.46
51.66
Cl
CO2Me
Br
N
Current Data Parameters
NAME MH 172
EXPNO 2
PROCNO 1
F2 - Acquisition Parameters
Date_ 20190605
Time 20.22 h
INSTRUM Avance
PROBHD Z116098_0219 (
PULPROG zgpg30
TD 65536
SOLVENT CDCl3
NS 1024
DS 4
SWH 23809.523 Hz
FIDRES 0.726609 Hz
AQ 1.3762560 sec
RG 20.6801
DW 21.000 usec
DE 6.50 usec
TE 298.0 K
D1 2.00000000 sec
D11 0.03000000 sec
TD0 1
SFO1 100.6228298 MHz
NUC1 13C
P0 3.33 usec
P1 10.00 usec
PLW1 73.73300171 W
SFO2 400.1316005 MHz
NUC2 1H
CPDPRG[2 waltz16
PCPD2 90.00 usec
PLW2 16.35899925 W
PLW12 0.20196000 W
PLW13 0.10159000 W
F2 - Processing parameters
SI 32768
SF 100.6127603 MHz
WDW EM
SSB 0
LB 1.00 Hz
GB 0
PC 1.40
200 180 160 140 120 100 80 60 40 20 0 ppm

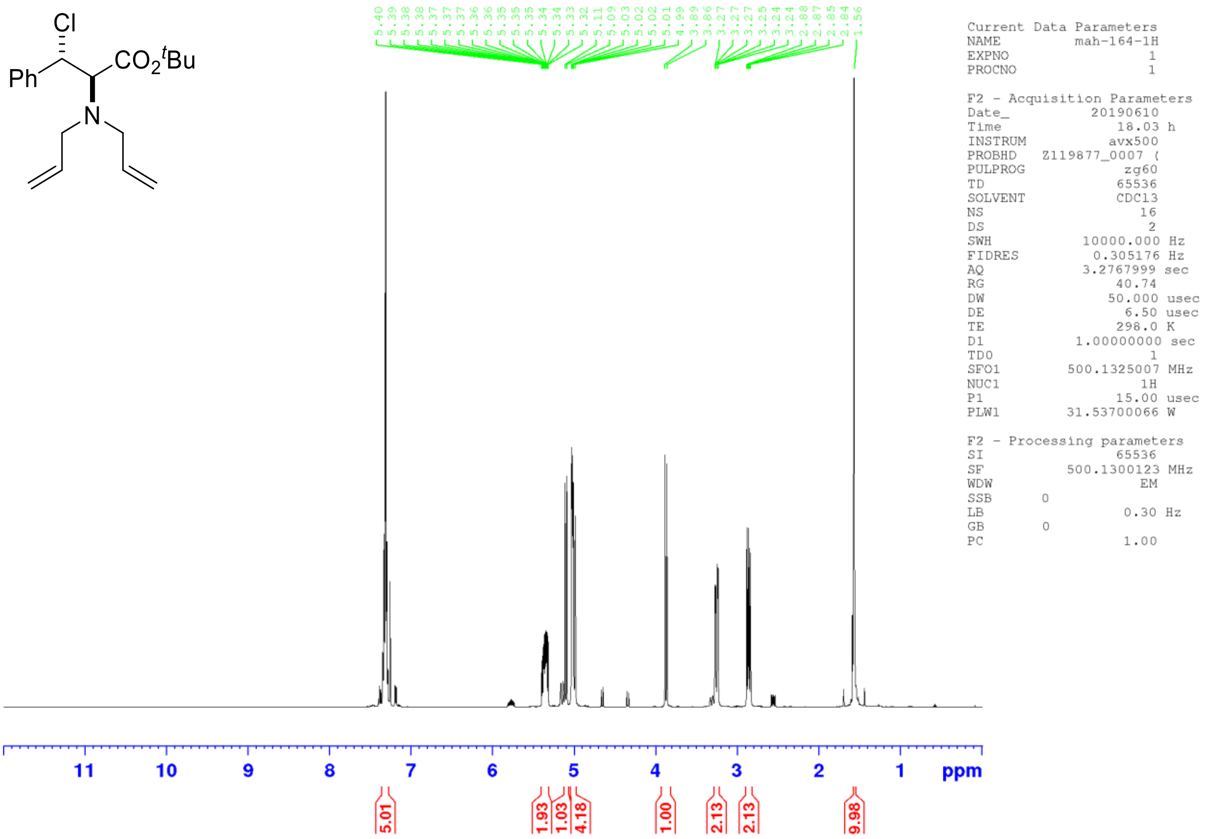
Cl
Ph
CO2tBu
N
5.40 5.39 5.38 5.38 5.37 5.37 5.37 5.36 5.36 5.35 5.35 5.35 5.34 5.34 5.33 5.32 5.11 5.09 5.03 5.02 5.02 5.01 4.99 3.89 3.86 3.27 3.27 3.27 3.25 3.24 3.24 2.88 2.87 2.85 2.84 1.56
Current Data Parameters
NAME mah-164-1H
EXPNO 1
PROCNO 1
F2 - Acquisition Parameters
Date_ 20190610
Time 18.03 h
INSTRUM avx500
PROBHD Z119877_0007 (
PULPROG zg60
TD 65536
SOLVENT CDCl3
NS 16
DS 2
SWH 10000.000 Hz
FIDRES 0.305176 Hz
AQ 3.2767999 sec
RG 40.74
DW 50.000 usec
DE 6.50 usec
TE 298.0 K
D1 1.00000000 sec
TD0 1
SFO1 500.1325007 MHz
NUC1 1H
P1 15.00 usec
PLW1 31.53700066 W
F2 - Processing parameters
SI 65536
SF 500.1300123 MHz
WDW EM
SSB 0
LB 0.30 Hz
GB 0
PC 1.00
11 10 9 8 7 6 5 4 3 2 1 ppm
5.01
1.93
1.03
4.18
1.00
2.13
2.13
9.98

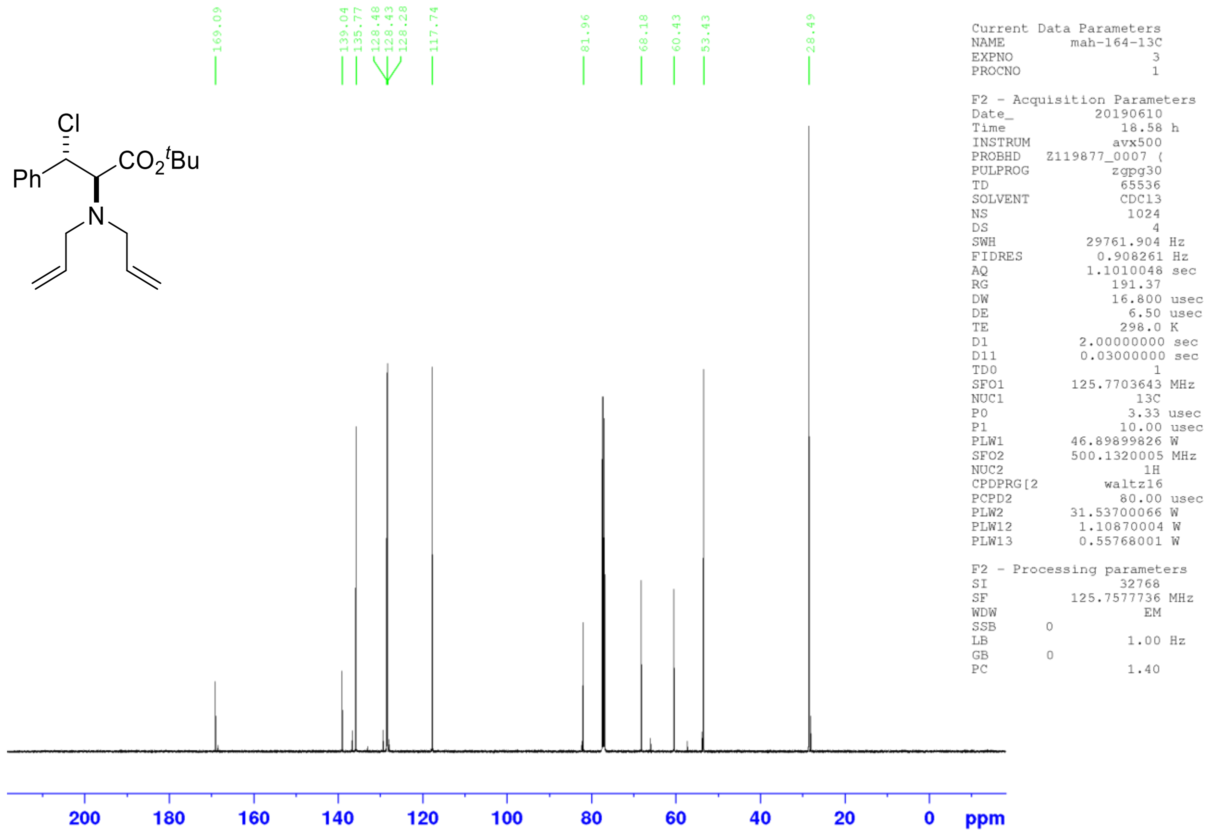

Current Data Parameters
NAME mah-164-13C
EXPNO 3
PROCNO 1

F2 - Acquisition Parameters
Date_ 20190610
Time 18.58 h
INSTRUM avx500
PROBHD Z119877_0007 (
PULPROG zgpg30
TD 65536
SOLVENT CDCl3
NS 1024
DS 4
SWH 29761.904 Hz
FIDRES 0.908261 Hz
AQ 1.1010048 sec
RG 191.37
DW 16.800 usec
DE 6.50 usec
TE 298.0 K
D1 2.00000000 sec
D11 0.03000000 sec
TD0 1
SFO1 125.7703643 MHz
NUC1 13C
P0 3.33 usec
P1 10.00 usec
PLW1 46.89899826 W
SFO2 500.1320005 MHz
NUC2 1H
CPDPRG[2 waltz16
PCPD2 80.00 usec
PLW2 31.53700066 W
PLW12 1.10870004 W
PLW13 0.55768001 W

F2 - Processing parameters
SI 32768
SF 125.7577736 MHz
WDW EM
SSB 0
LB 1.00 Hz
GB 0
PC 1.40

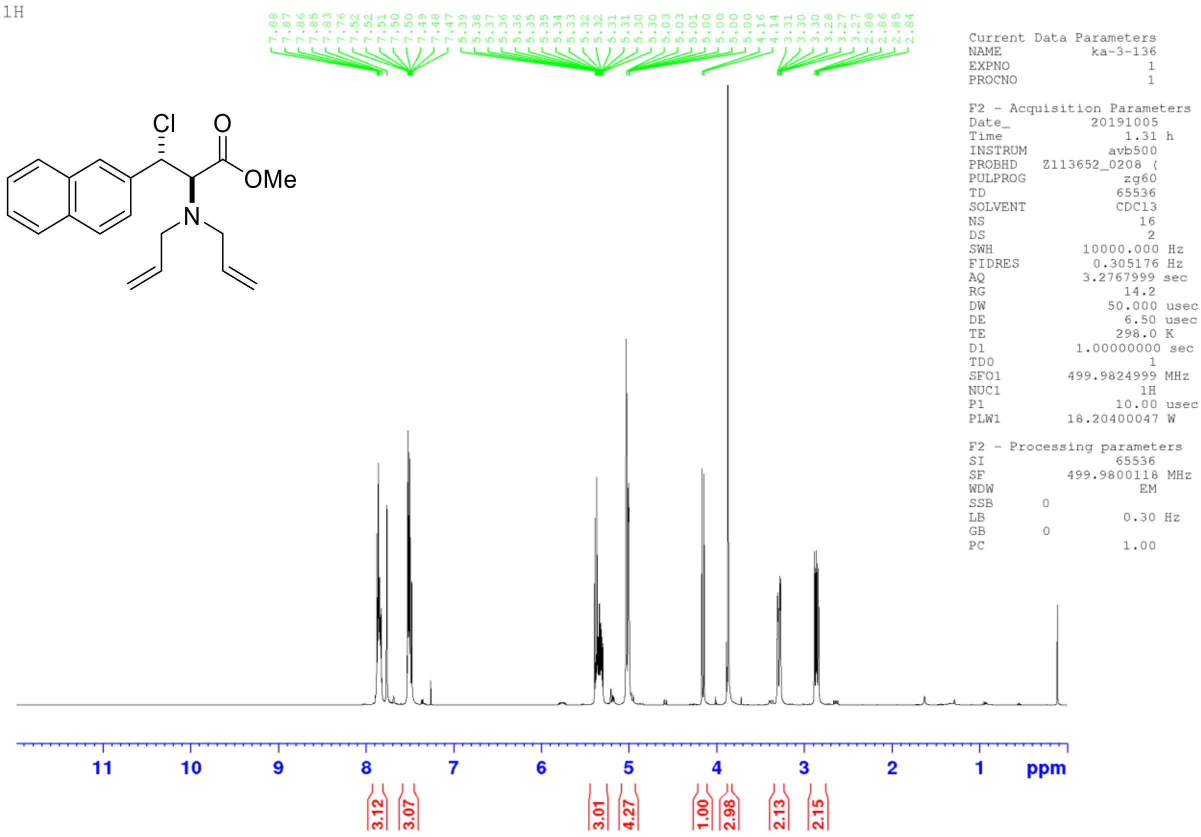

Current Data Parameters
NAME ka-3-136
EXPNO 1
PROCNO 1

F2 - Acquisition Parameters
Date_ 20191005
Time 1.31 h
INSTRUM avb500
PROBHD Z113652_0208 (
PULPROG zg60
TD 65536
SOLVENT CDCl3
NS 16
DS 2
SWH 10000.000 Hz
FIDRES 0.305176 Hz
AQ 3.2767999 sec
RG 14.2
DW 50.000 usec
DE 6.50 usec
TE 298.0 K
D1 1.00000000 sec
TD0 1
SFO1 499.9824999 MHz
NUC1 1H
P1 10.00 usec
PLW1 18.20400047 W

F2 - Processing parameters
SI 65536
SF 499.9800118 MHz
WDW EM
SSB 0
LB 0.30 Hz
GB 0
PC 1.00

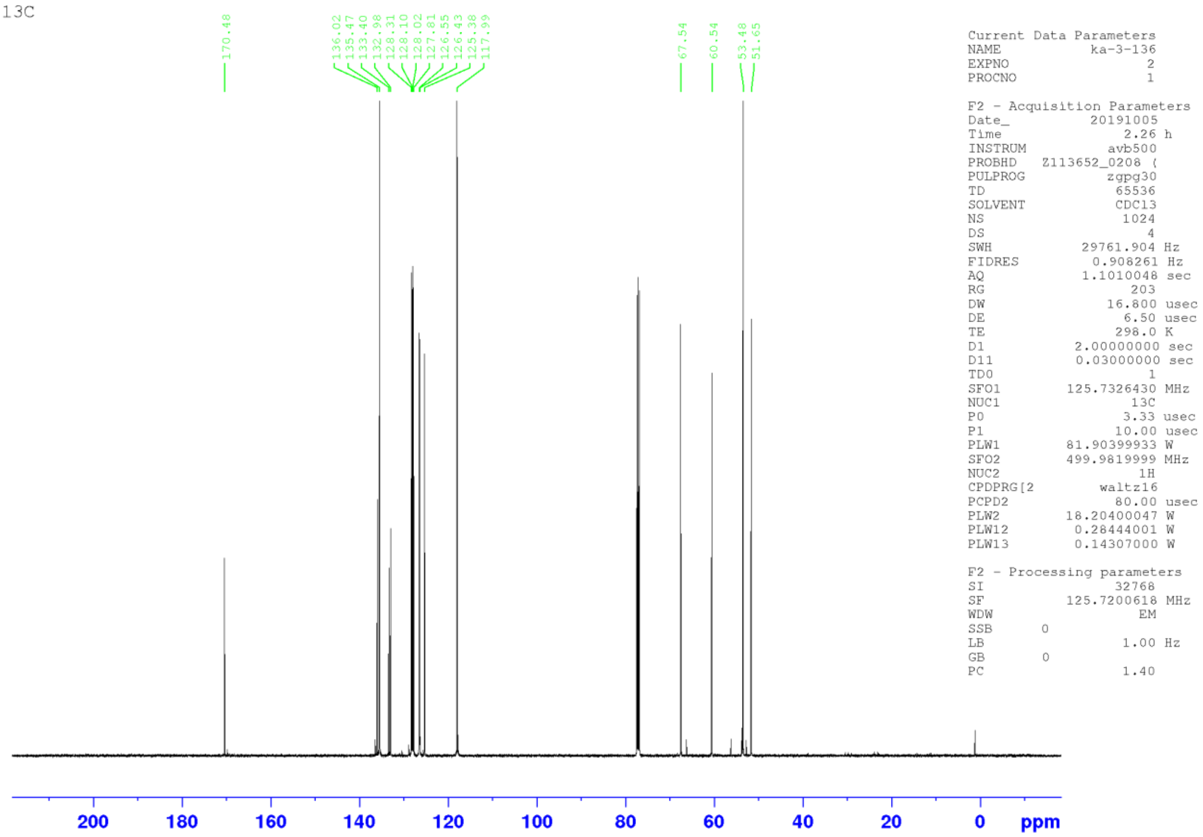
13C
Current Data Parameters
NAME ka-3-136
EXPNO 2
PROCNO 1
F2 - Acquisition Parameters
Date_ 20191005
Time 2.26 h
INSTRUM avb500
PROBHD Z113652_0208 (
PULPROG zgpg30
TD 65536
SOLVENT CDCl3
NS 1024
DS 4
SWH 29761.904 Hz
FIDRES 0.908261 Hz
AQ 1.1010048 sec
RG 203
DW 16.800 usec
DE 6.50 usec
TE 298.0 K
D1 2.00000000 sec
D11 0.03000000 sec
TD0 1
SFO1 125.7326430 MHz
NUC1 13C
P0 3.33 usec
P1 10.00 usec
PLW1 81.90399933 W
SFO2 499.9819999 MHz
NUC2 1H
CPDPRG[2 waltz16
PCPD2 80.00 usec
PLW2 18.20400047 W
PLW12 0.28444001 W
PLW13 0.14307000 W
F2 - Processing parameters
SI 32768
SF 125.7200618 MHz
WDW EM
SSB 0
LB 1.00 Hz
GB 0
PC 1.40
ppm

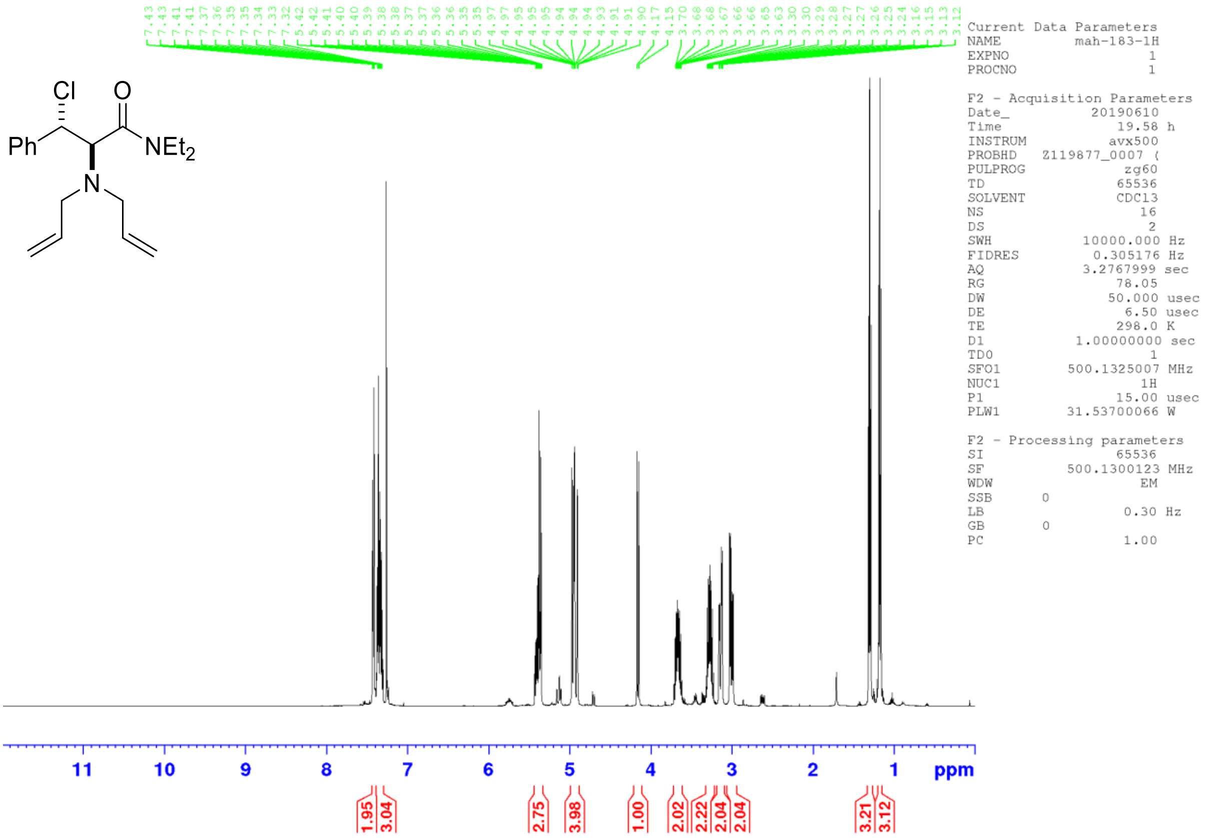
Cl
O
Ph
NEt2
N
Current Data Parameters
NAME mah-183-1H
EXPNO 1
PROCNO 1
F2 - Acquisition Parameters
Date_ 20190610
Time 19.58 h
INSTRUM avx500
PROBHD Z119877_0007 (
PULPROG zg60
TD 65536
SOLVENT CDCl3
NS 16
DS 2
SWH 10000.000 Hz
FIDRES 0.305176 Hz
AQ 3.2767999 sec
RG 78.05
DW 50.000 usec
DE 6.50 usec
TE 298.0 K
D1 1.00000000 sec
TD0 1
SFO1 500.1325007 MHz
NUC1 1H
P1 15.00 usec
PLW1 31.53700066 W
F2 - Processing parameters
SI 65536
SF 500.1300123 MHz
WDW EM
SSB 0
LB 0.30 Hz
GB 0
PC 1.00
ppm

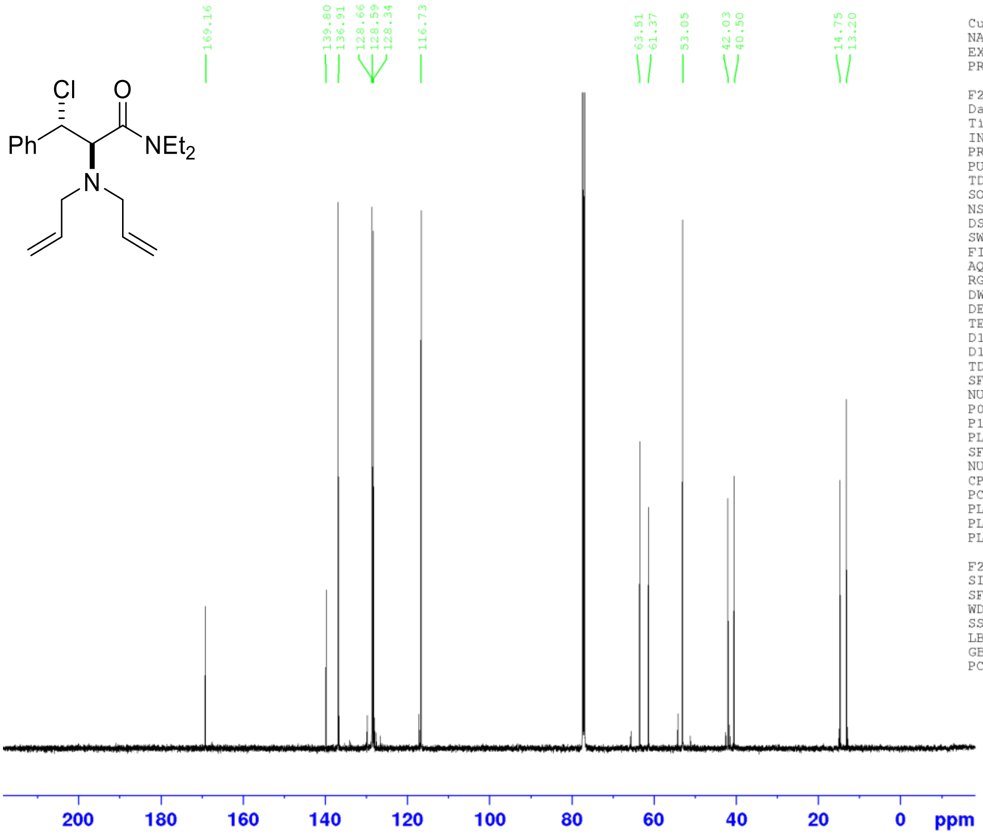


```
Current Data Parameters
NAME          mah-183-13C
EXPNO                   3
PROCNO                  1

F2 - Acquisition Parameters
Date_            20190610
Time                20.53 h
INSTRUM            avx500
PROBHD   Z119877_0007 (
PULPROG           zgpg30
TD                 65536
SOLVENT            CDCl3
NS                  1024
DS                     4
SWH           29761.904 Hz
FIDRES         0.908261 Hz
AQ            1.1010048 sec
RG               191.37
DW               16.800 usec
DE                 6.50 usec
TE                298.0 K
D1           2.00000000 sec
D11          0.03000000 sec
TD0                    1
SFO1        125.7703643 MHz
NUC1                 13C
P0                 3.33 usec
P1                10.00 usec
PLW1        46.89899826 W
SFO2        500.1320005 MHz
NUC2                  1H
CPDPRG[2         waltz16
PCPD2             80.00 usec
PLW2        31.53700066 W
PLW12        1.10870004 W
PLW13        0.55768001 W

F2 - Processing parameters
SI                 32768
SF          125.7577745 MHz
WDW                   EM
SSB      0
LB                  1.00 Hz
GB       0
PC                  1.40
```

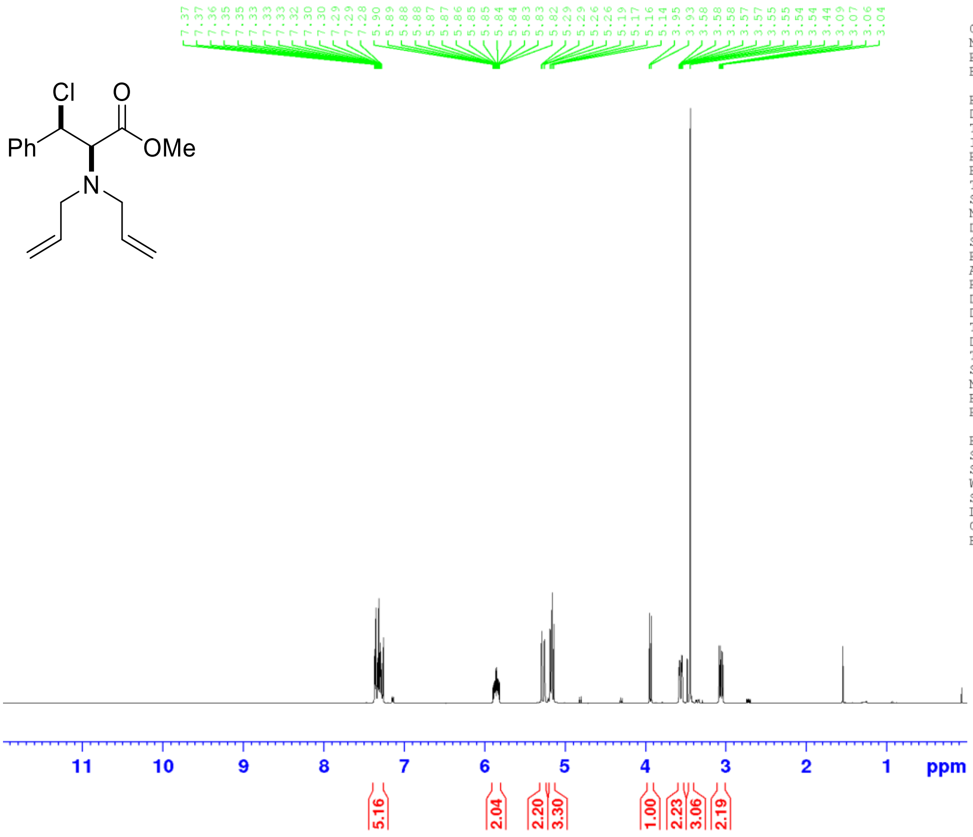


```
Current Data Parameters
NAME          mah-254-1H
EXPNO                  1
PROCNO                 1

F2 - Acquisition Parameters
Date_            20191004
Time                13.05 h
INSTRUM            avx500
PROBHD   Z119877_0007 (
PULPROG             zg60
TD                 65536
SOLVENT            CDCl3
NS                    16
DS                     2
SWH           10000.000 Hz
FIDRES         0.305176 Hz
AQ            3.2767999 sec
RG               170.58
DW               50.000 usec
DE                 6.50 usec
TE                298.0 K
D1           1.00000000 sec
TD0                    1
SFO1        500.1325007 MHz
NUC1                  1H
P1                15.00 usec
PLW1        31.53700066 W

F2 - Processing parameters
SI                 65536
SF          500.1300123 MHz
WDW                   EM
SSB      0
LB                  0.30 Hz
GB       0
PC                  1.00
```

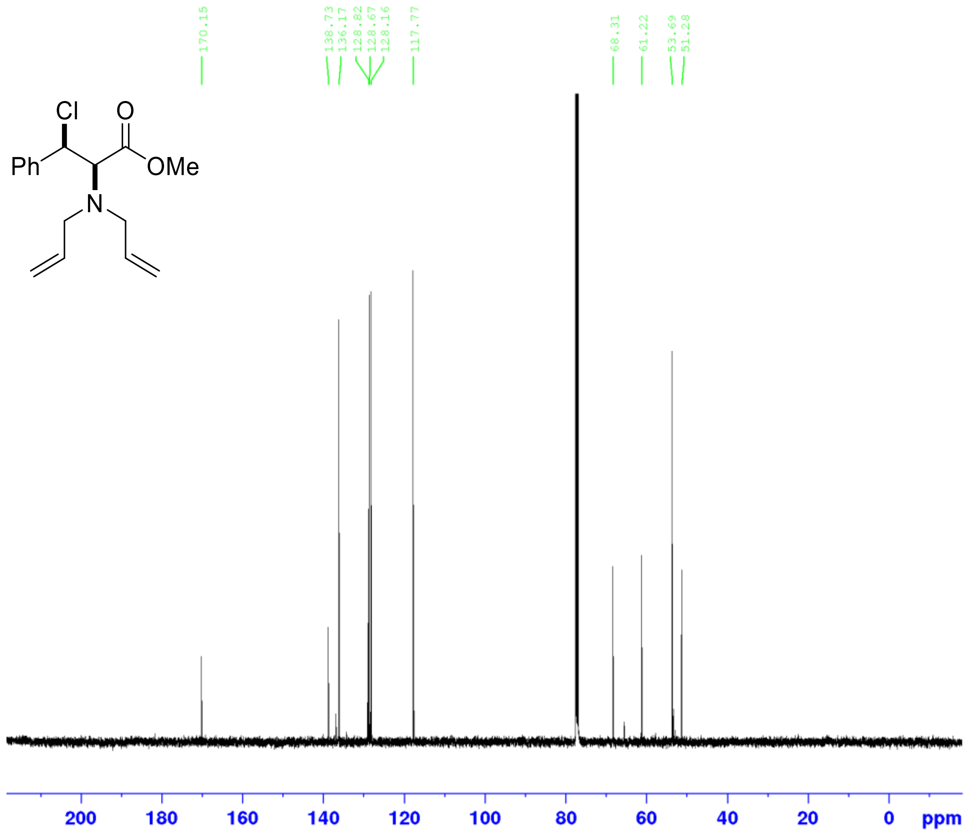

Current Data Parameters
NAME mah-254-13C
EXPNO 3
PROCNO 1

F2 - Acquisition Parameters
Date_ 20191004
Time 14.00 h
INSTRUM avx500
PROBHD Z119877_0007 (
PULPROG zgpg30
TD 65536
SOLVENT CDCl3
NS 1024
DS 4
SWH 29761.904 Hz
FIDRES 0.908261 Hz
AQ 1.1010048 sec
RG 191.37
DW 16.800 usec
DE 6.50 usec
TE 298.0 K
D1 2.00000000 sec
D11 0.03000000 sec
TD0 1
SFO1 125.7703643 MHz
NUC1 13C
P0 3.33 usec
P1 10.00 usec
PLW1 46.89899826 W
SFO2 500.1320005 MHz
NUC2 1H
CPDPRG[2 waltz16
PCPD2 80.00 usec
PLW2 31.53700066 W
PLW12 1.10870004 W
PLW13 0.55768001 W

F2 - Processing parameters
SI 32768
SF 125.7577718 MHz
WDW EM
SSB 0
LB 1.00 Hz
GB 0
PC 1.40

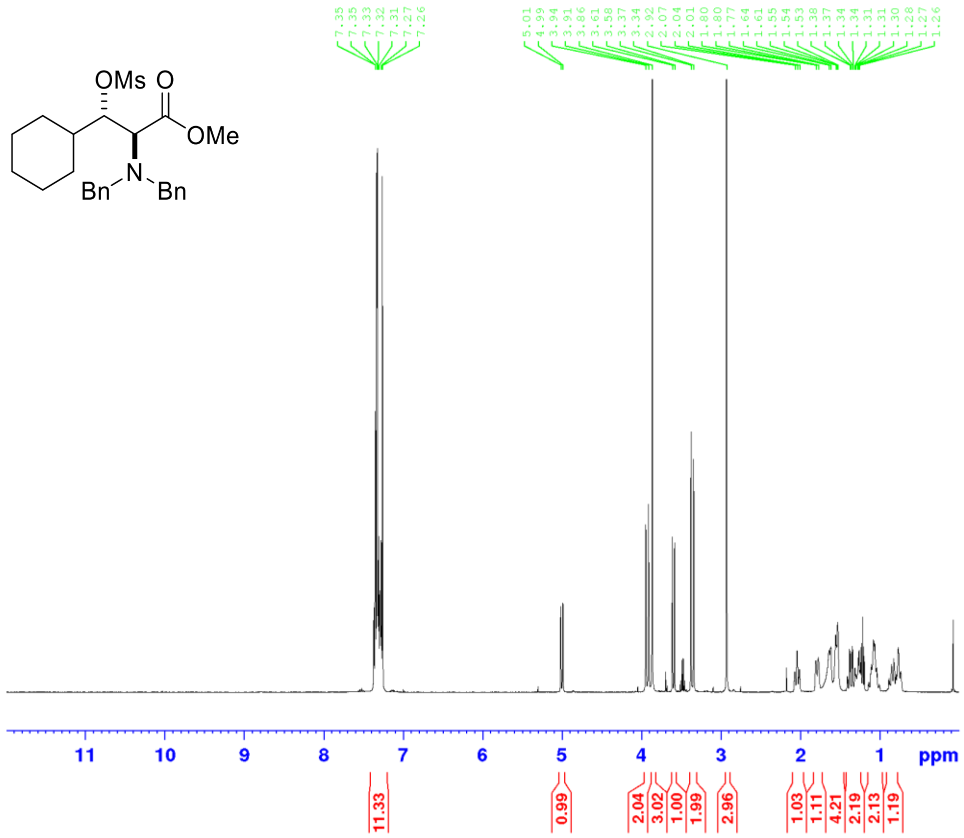

Current Data Parameters
NAME ka-3-70
EXPNO 1
PROCNO 1

F2 - Acquisition Parameters
Date_ 20190823
Time 9.22 h
INSTRUM Avance
PROBHD Z116098_0219 (
PULPROG zg30
TD 65536
SOLVENT CDCl3
NS 32
DS 2
SWH 8196.722 Hz
FIDRES 0.250144 Hz
AQ 3.9976959 sec
RG 101
DW 61.000 usec
DE 6.50 usec
TE 298.0 K
D1 1.00000000 sec
TD0 1
SFO1 400.1320007 MHz
NUC1 1H
P0 3.33 usec
P1 10.00 usec
PLW1 16.35899925 W

F2 - Processing parameters
SI 65536
SF 400.1300097 MHz
WDW EM
SSB 0
LB 0.30 Hz
GB 0
PC 1.00

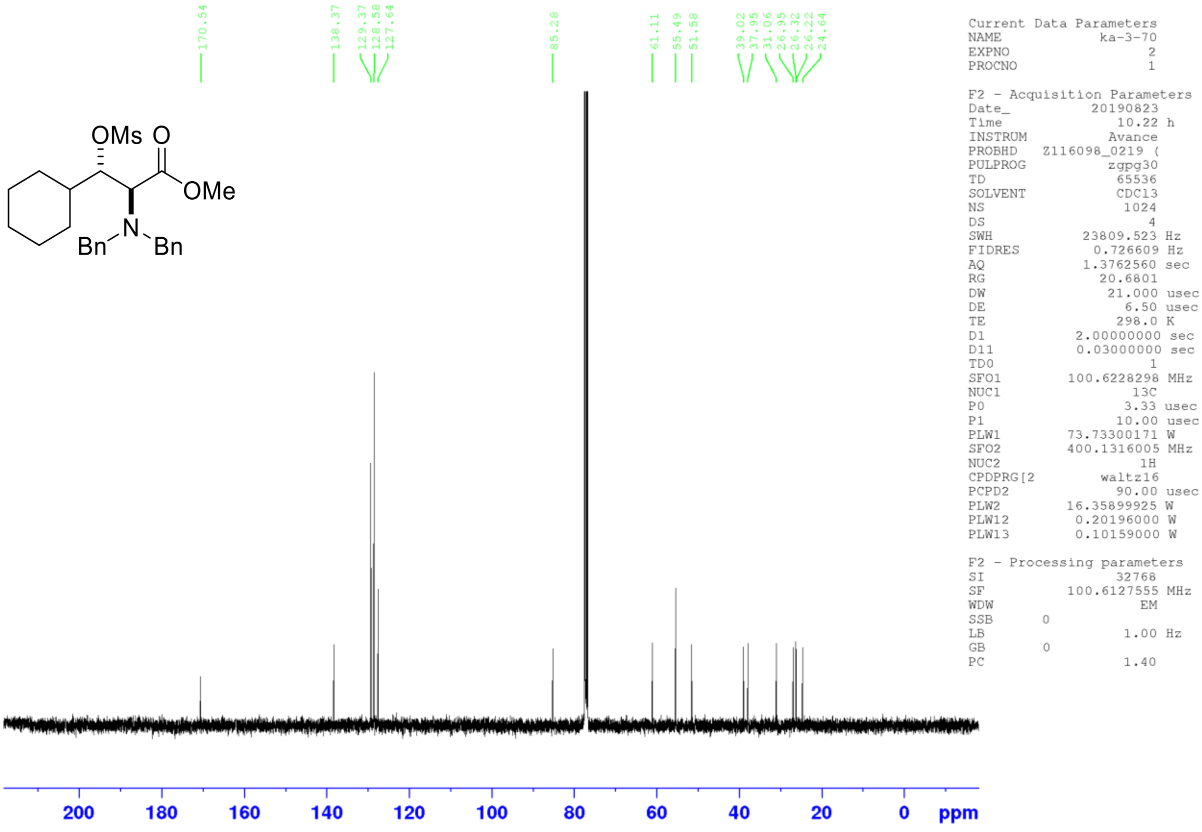


```
Current Data Parameters
NAME              ka-3-70
EXPNO                   2
PROCNO                  1

F2 - Acquisition Parameters
Date_            20190823
Time                10.22 h
INSTRUM            Avance
PROBHD   Z116098_0219 (
PULPROG           zgpg30
TD                  65536
SOLVENT             CDCl3
NS                   1024
DS                      4
SWH             23809.523 Hz
FIDRES           0.726609 Hz
AQ              1.3762560 sec
RG                20.6801
DW                 21.000 usec
DE                   6.50 usec
TE                  298.0 K
D1             2.00000000 sec
D11            0.03000000 sec
TD0                     1
SFO1          100.6228298 MHz
NUC1                  13C
P0                   3.33 usec
P1                  10.00 usec
PLW1          73.73300171 W
SFO2          400.1316005 MHz
NUC2                   1H
CPDPRG[2          waltz16
PCPD2               90.00 usec
PLW2          16.35899925 W
PLW12          0.20196000 W
PLW13          0.10159000 W

F2 - Processing parameters
SI                  32768
SF            100.6127555 MHz
WDW                    EM
SSB      0
LB                   1.00 Hz
GB       0
PC                   1.40
```

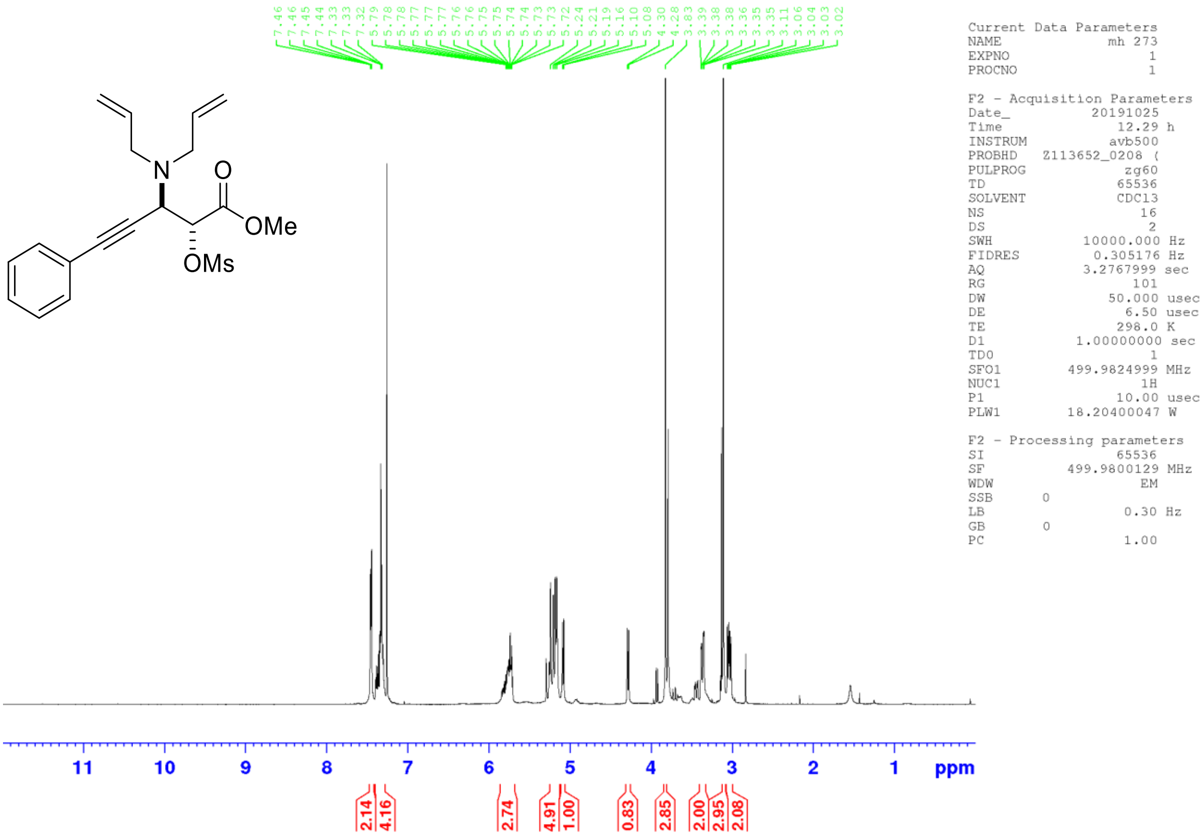


```
Current Data Parameters
NAME               mh 273
EXPNO                   1
PROCNO                  1

F2 - Acquisition Parameters
Date_            20191025
Time                12.29 h
INSTRUM            avb500
PROBHD   Z113652_0208 (
PULPROG              zg60
TD                  65536
SOLVENT             CDCl3
NS                     16
DS                      2
SWH             10000.000 Hz
FIDRES           0.305176 Hz
AQ              3.2767999 sec
RG                    101
DW                 50.000 usec
DE                   6.50 usec
TE                  298.0 K
D1             1.00000000 sec
TD0                     1
SFO1          499.9824999 MHz
NUC1                   1H
P1                  10.00 usec
PLW1          18.20400047 W

F2 - Processing parameters
SI                  65536
SF            499.9800129 MHz
WDW                    EM
SSB      0
LB                   0.30 Hz
GB       0
PC                   1.00
```

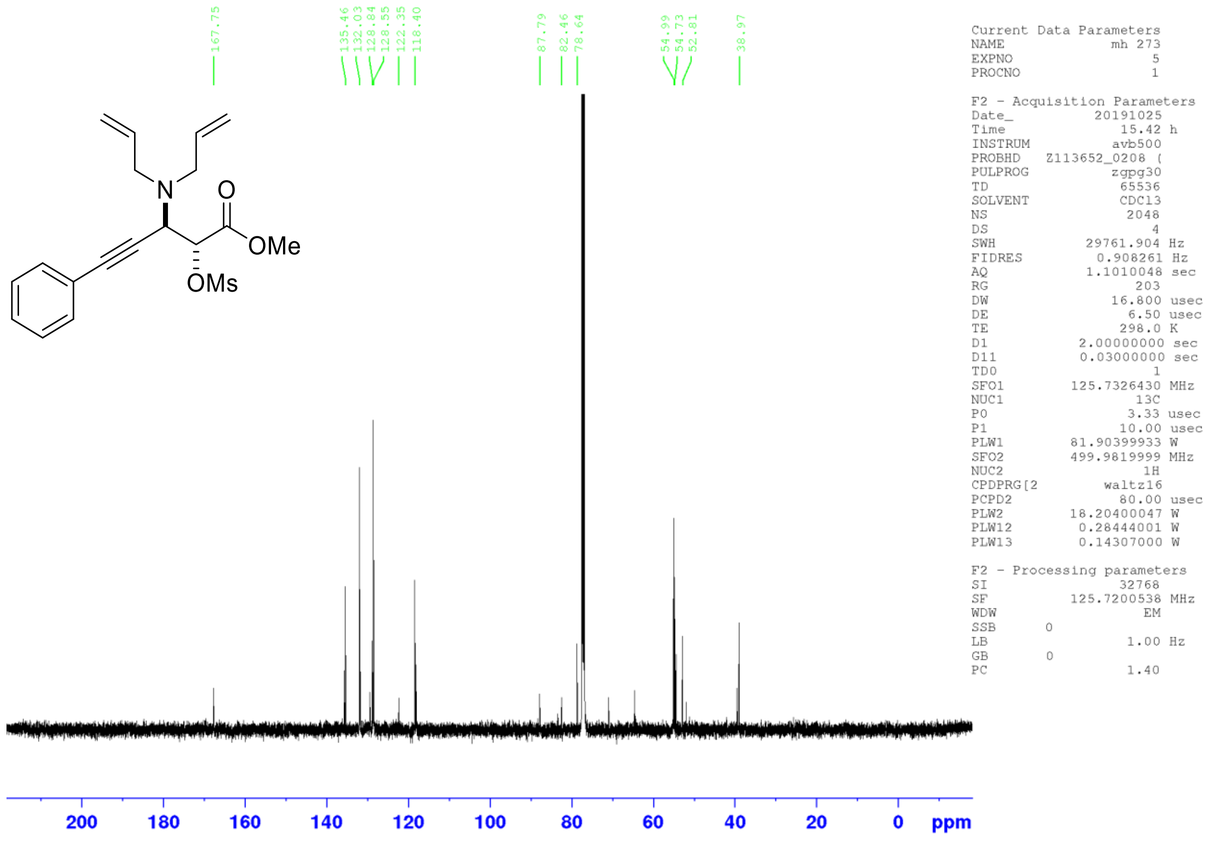

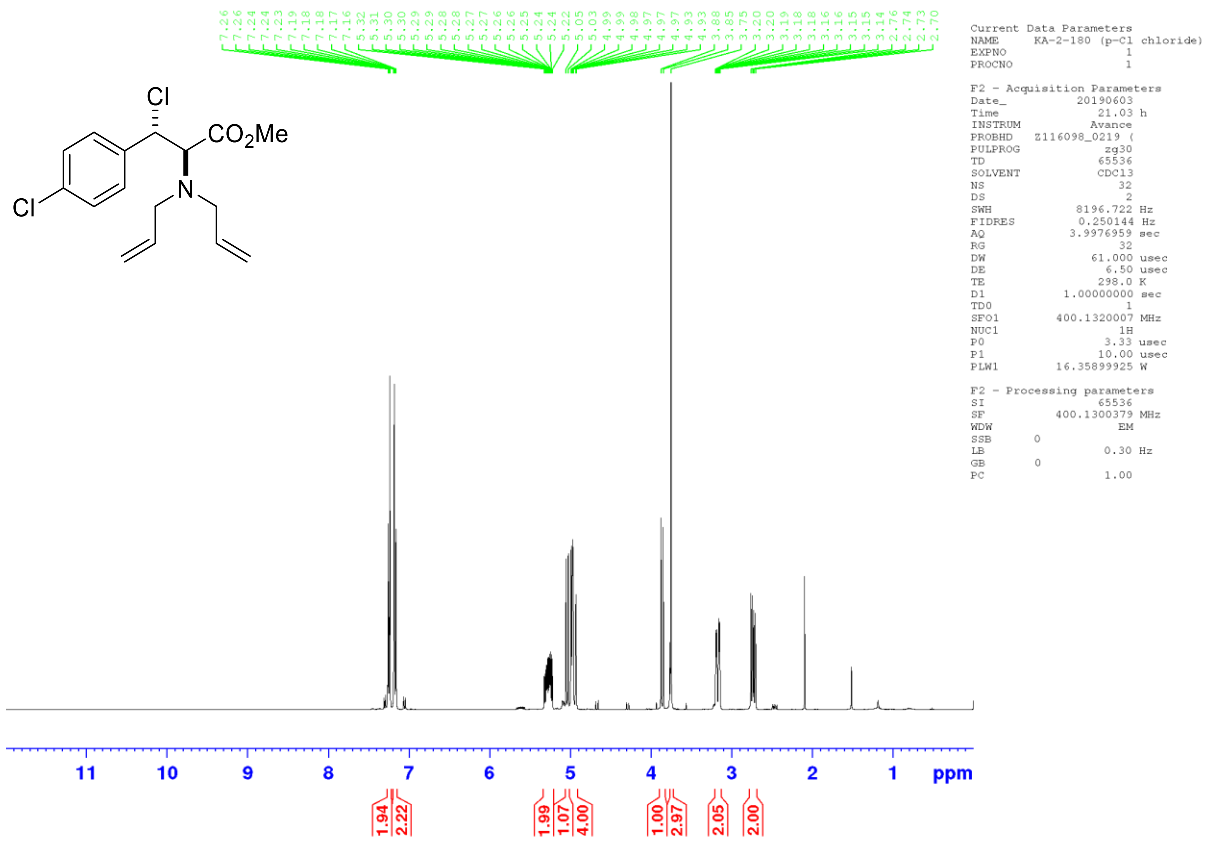

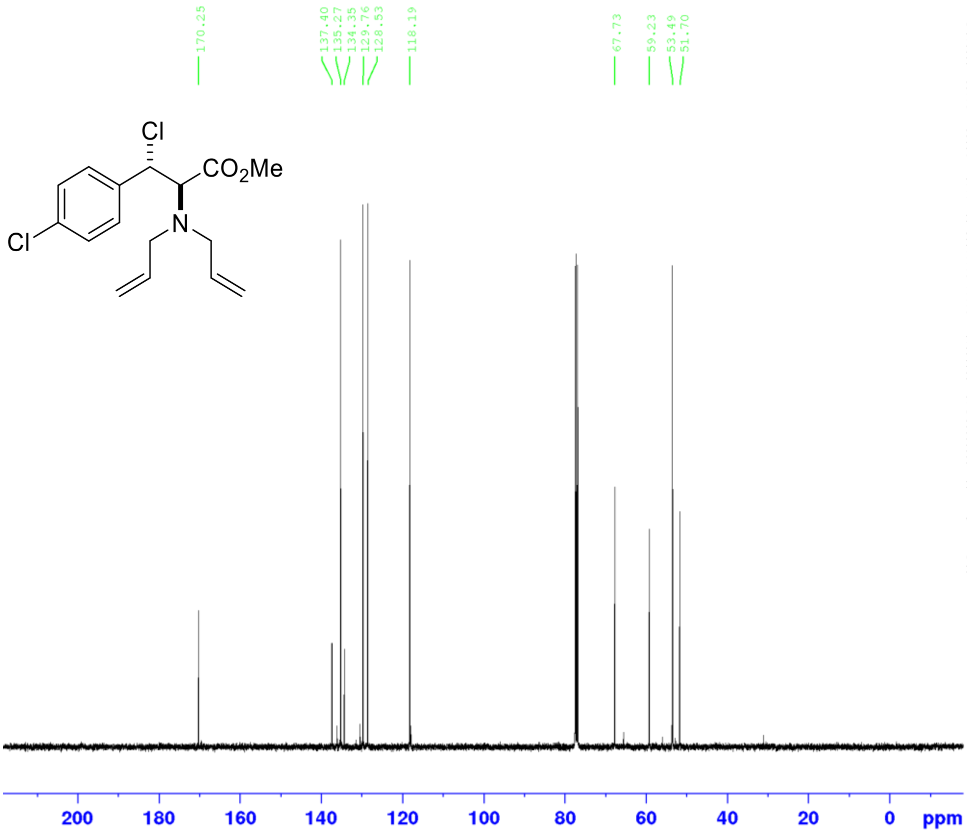

Current Data Parameters
NAME KA-2-180 (p-Cl chloride)
EXPNO 2
PROCNO 1

F2 - Acquisition Parameters
Date_ 20190603
Time 22.17 h
INSTRUM Avance
PROBHD Z116098_0219 (
PULPROG zgpg30
TD 65536
SOLVENT CDCl3
NS 1280
DS 4
SWH 23809.523 Hz
FIDRES 0.726609 Hz
AQ 1.3762560 sec
RG 9.375
DW 21.000 usec
DE 6.50 usec
TE 298.0 K
D1 2.00000000 sec
D11 0.03000000 sec
TD0 1
SFO1 100.6228298 MHz
NUC1 13C
P0 3.33 usec
P1 10.00 usec
PLW1 73.73300171 W
SFO2 400.1316005 MHz
NUC2 1H
CPDPRG[2 waltz16
PCPD2 90.00 usec
PLW2 16.35899925 W
PLW12 0.20196000 W
PLW13 0.10159000 W

F2 - Processing parameters
SI 32768
SF 100.6127564 MHz
WDW EM
SSB 0
LB 1.00 Hz
GB 0
PC 1.40

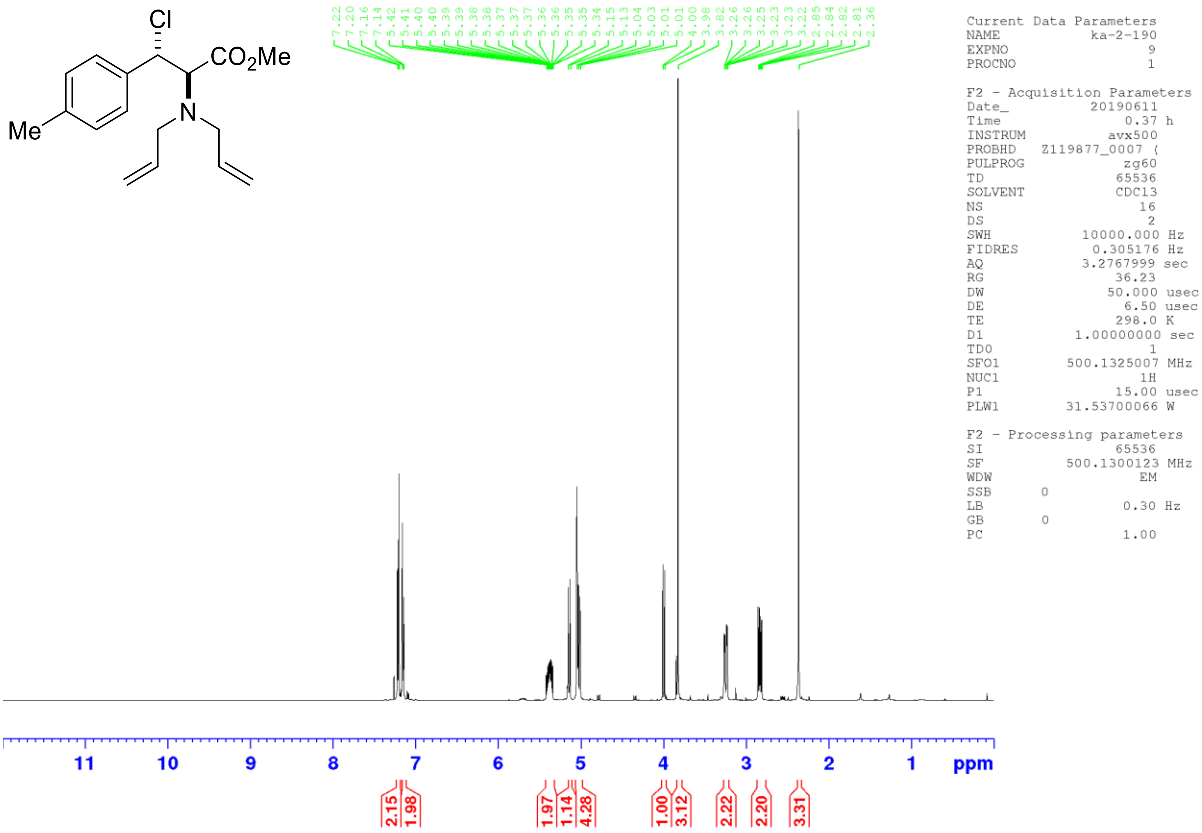

Current Data Parameters
NAME ka-2-190
EXPNO 9
PROCNO 1

F2 - Acquisition Parameters
Date_ 20190611
Time 0.37 h
INSTRUM avx500
PROBHD Z119877_0007 (
PULPROG zg60
TD 65536
SOLVENT CDCl3
NS 16
DS 2
SWH 10000.000 Hz
FIDRES 0.305176 Hz
AQ 3.2767999 sec
RG 36.23
DW 50.000 usec
DE 6.50 usec
TE 298.0 K
D1 1.00000000 sec
TD0 1
SFO1 500.1325007 MHz
NUC1 1H
P1 15.00 usec
PLW1 31.53700066 W

F2 - Processing parameters
SI 65536
SF 500.1300123 MHz
WDW EM
SSB 0
LB 0.30 Hz
GB 0
PC 1.00

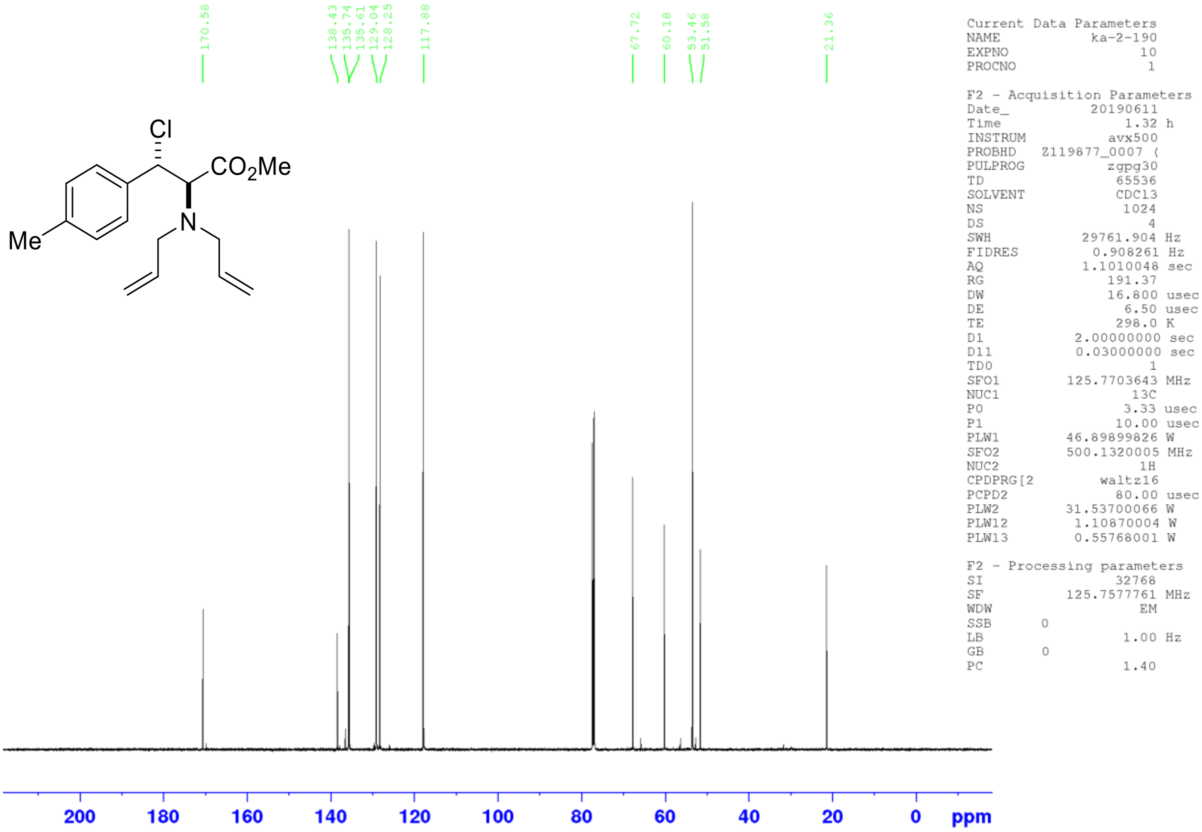
170.58
138.43
135.74
135.61
129.04
128.25
117.88
67.72
60.18
53.46
51.58
21.36
Cl
CO2Me
Me
N
200
180
160
140
120
100
80
60
40
20
0
ppm
Current Data Parameters
NAME ka-2-190
EXPNO 10
PROCNO 1
F2 - Acquisition Parameters
Date_ 20190611
Time 1.32 h
INSTRUM avx500
PROBHD Z119877_0007 (
PULPROG zgpg30
TD 65536
SOLVENT CDCl3
NS 1024
DS 4
SWH 29761.904 Hz
FIDRES 0.908261 Hz
AQ 1.1010048 sec
RG 191.37
DW 16.800 usec
DE 6.50 usec
TE 298.0 K
D1 2.00000000 sec
D11 0.03000000 sec
TD0 1
SFO1 125.7703643 MHz
NUC1 13C
P0 3.33 usec
P1 10.00 usec
PLW1 46.89899826 W
SFO2 500.1320005 MHz
NUC2 1H
CPDPRG[2 waltz16
PCPD2 80.00 usec
PLW2 31.53700066 W
PLW12 1.10870004 W
PLW13 0.55768001 W
F2 - Processing parameters
SI 32768
SF 125.7577761 MHz
WDW EM
SSB 0
LB 1.00 Hz
GB 0
PC 1.40

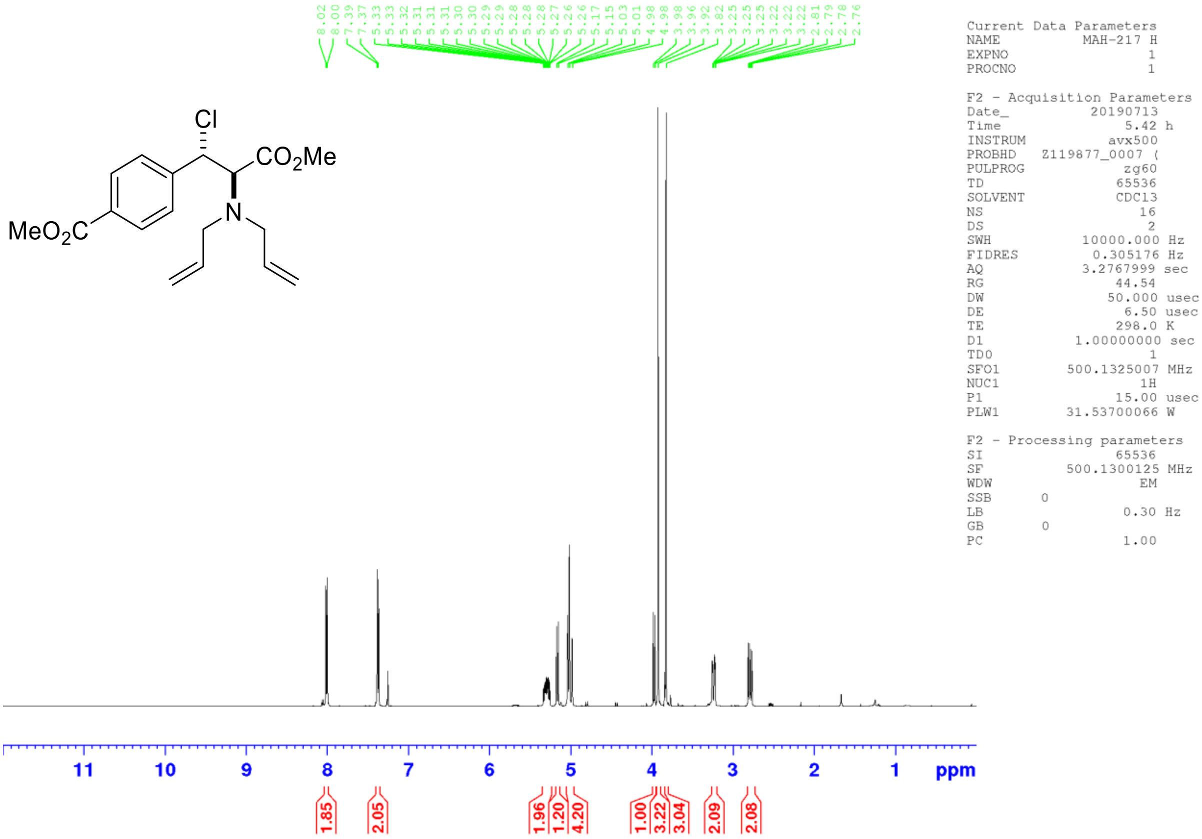
8.02
8.00
7.39
7.37
5.33
5.33
5.32
5.31
5.31
5.31
5.30
5.29
5.29
5.28
5.28
5.28
5.27
5.26
5.26
5.17
5.15
5.03
5.01
4.98
4.98
3.98
3.96
3.92
3.82
3.25
3.25
3.22
3.22
3.22
2.81
2.79
2.78
2.76
Cl
CO2Me
MeO2C
N
11
10
9
8
7
6
5
4
3
2
1
ppm
1.85
2.05
1.96
1.20
4.20
1.00
3.22
3.04
2.09
2.08
Current Data Parameters
NAME MAH-217 H
EXPNO 1
PROCNO 1
F2 - Acquisition Parameters
Date_ 20190713
Time 5.42 h
INSTRUM avx500
PROBHD Z119877_0007 (
PULPROG zg60
TD 65536
SOLVENT CDCl3
NS 16
DS 2
SWH 10000.000 Hz
FIDRES 0.305176 Hz
AQ 3.2767999 sec
RG 44.54
DW 50.000 usec
DE 6.50 usec
TE 298.0 K
D1 1.00000000 sec
TD0 1
SFO1 500.1325007 MHz
NUC1 1H
P1 15.00 usec
PLW1 31.53700066 W
F2 - Processing parameters
SI 65536
SF 500.1300125 MHz
WDW EM
SSB 0
LB 0.30 Hz
GB 0
PC 1.00

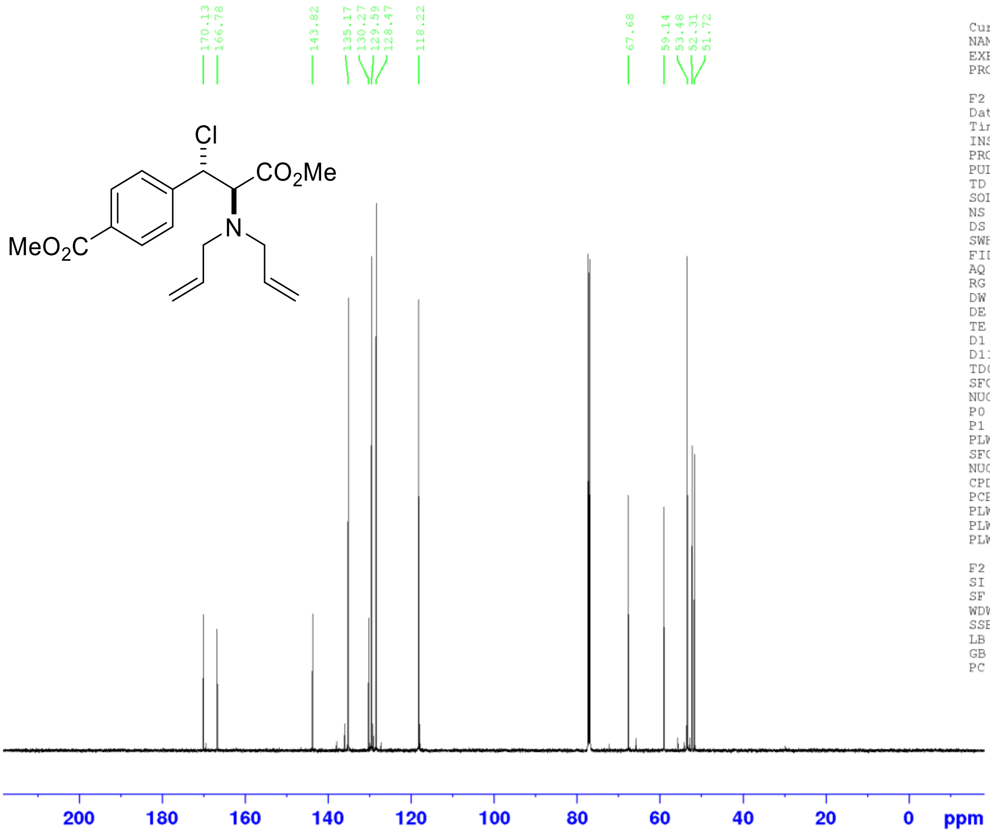

Current Data Parameters
NAME MAH-217 C
EXPNO 5
PROCNO 1

F2 - Acquisition Parameters
Date_ 20190713
Time 6.36 h
INSTRUM avx500
PROBHD Z119877_0007 (
PULPROG zgpg30
TD 65536
SOLVENT CDCl3
NS 1024
DS 4
SWH 29761.904 Hz
FIDRES 0.908261 Hz
AQ 1.1010048 sec
RG 191.37
DW 16.800 usec
DE 6.50 usec
TE 298.0 K
D1 2.00000000 sec
D11 0.03000000 sec
TD0 1
SFO1 125.7703643 MHz
NUC1 13C
P0 3.33 usec
P1 10.00 usec
PLW1 46.89899826 W
SFO2 500.1320005 MHz
NUC2 1H
CPDPRG[2 waltz16
PCPD2 80.00 usec
PLW2 31.53700066 W
PLW12 1.10870004 W
PLW13 0.55768001 W

F2 - Processing parameters
SI 32768
SF 125.7577749 MHz
WDW EM
SSB 0
LB 1.00 Hz
GB 0
PC 1.40

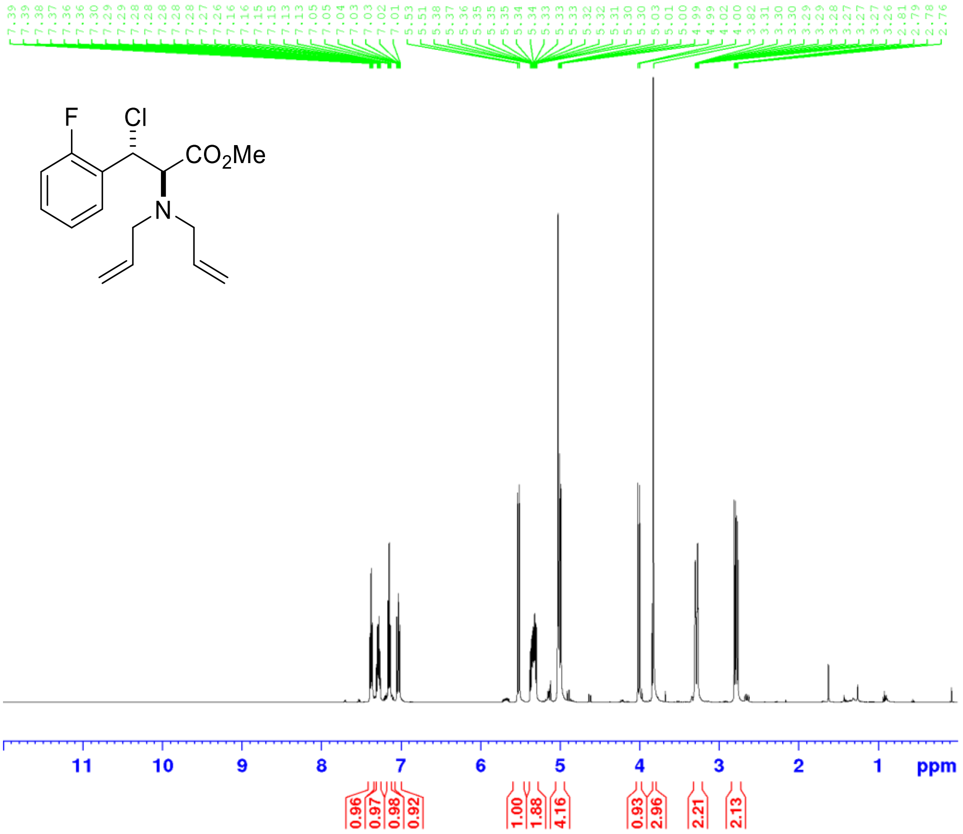

Current Data Parameters
NAME ka-3-72
EXPNO 1
PROCNO 1

F2 - Acquisition Parameters
Date_ 20191001
Time 22.53 h
INSTRUM avb500
PROBHD Z113652_0208 (
PULPROG zg60
TD 65536
SOLVENT CDCl3
NS 16
DS 2
SWH 10000.000 Hz
FIDRES 0.305176 Hz
AQ 3.2767999 sec
RG 18
DW 50.000 usec
DE 6.50 usec
TE 298.0 K
D1 1.00000000 sec
TD0 1
SFO1 499.9824999 MHz
NUC1 1H
P1 10.00 usec
PLW1 18.20400047 W

F2 - Processing parameters
SI 65536
SF 499.9800121 MHz
WDW EM
SSB 0
LB 0.30 Hz
GB 0
PC 1.00

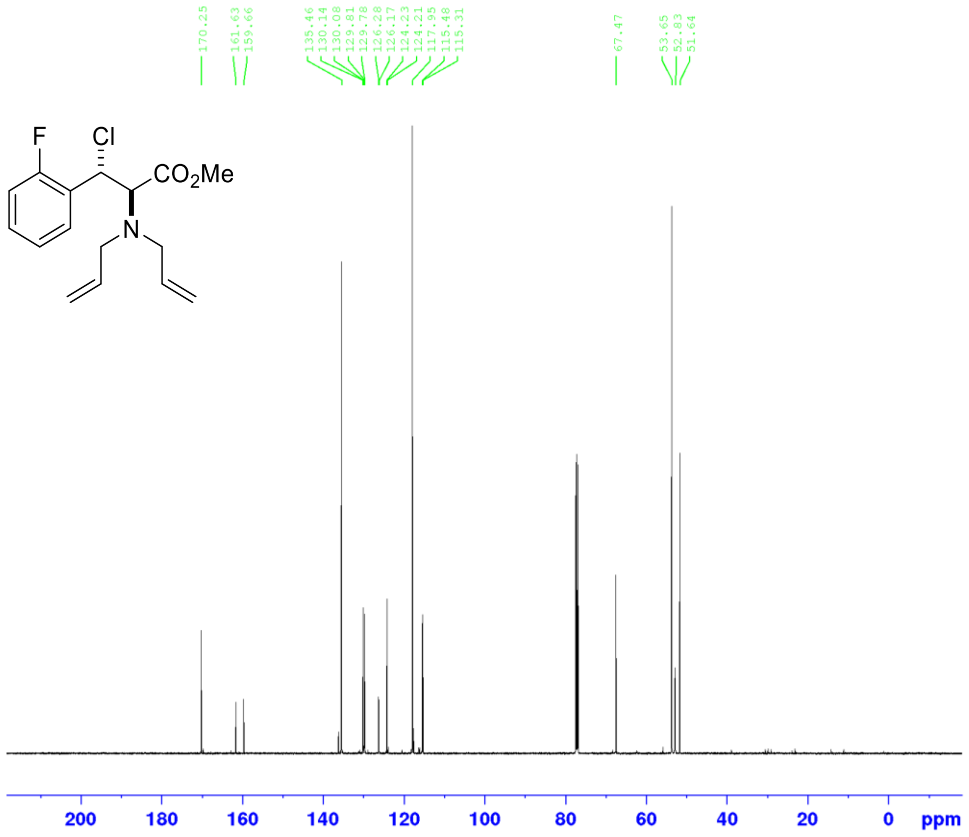

Current Data Parameters
NAME ka-3-72
EXPNO 2
PROCNO 1

F2 - Acquisition Parameters
Date_ 20191001
Time 23.48 h
INSTRUM avb500
PROBHD Z113652_0208 (
PULPROG zgpg30
TD 65536
SOLVENT CDCl3
NS 1024
DS 4
SWH 29761.904 Hz
FIDRES 0.908261 Hz
AQ 1.1010048 sec
RG 203
DW 16.800 usec
DE 6.50 usec
TE 298.0 K
D1 2.00000000 sec
D11 0.03000000 sec
TD0 1
SFO1 125.7326430 MHz
NUC1 13C
P0 3.33 usec
P1 10.00 usec
PLW1 81.90399933 W
SFO2 499.9819999 MHz
NUC2 1H
CPDPRG[2 waltz16
PCPD2 80.00 usec
PLW2 18.20400047 W
PLW12 0.28444001 W
PLW13 0.14307000 W

F2 - Processing parameters
SI 32768
SF 125.7200574 MHz
WDW EM
SSB 0
LB 1.00 Hz
GB 0
PC 1.40

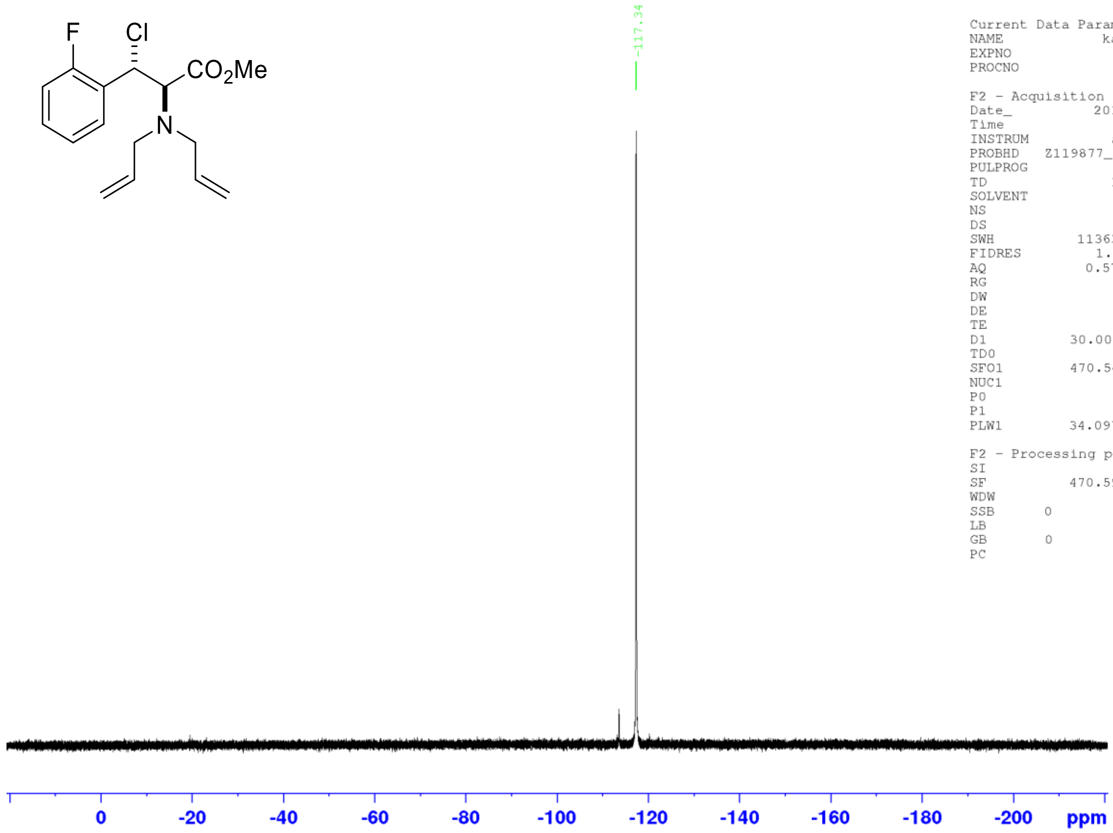

Current Data Parameters
NAME ka-3-72
EXPNO 22
PROCNO 1

F2 - Acquisition Parameters
Date_ 20191011
Time 15.19 h
INSTRUM avx500
PROBHD Z119877_0007 (
PULPROG zg30
TD 130840
SOLVENT CDCl3
NS 16
DS 4
SWH 113636.367 Hz
FIDRES 1.737028 Hz
AQ 0.5756960 sec
RG 40.74
DW 4.400 usec
DE 6.50 usec
TE 298.0 K
D1 30.00000000 sec
TD0 1
SFO1 470.5453178 MHz
NUC1 19F
P0 7.33 usec
P1 22.00 usec
PLW1 34.09799957 W

F2 - Processing parameters
SI 65536
SF 470.5923770 MHz
WDW EM
SSB 0
LB 0.30 Hz
GB 0
PC 1.00

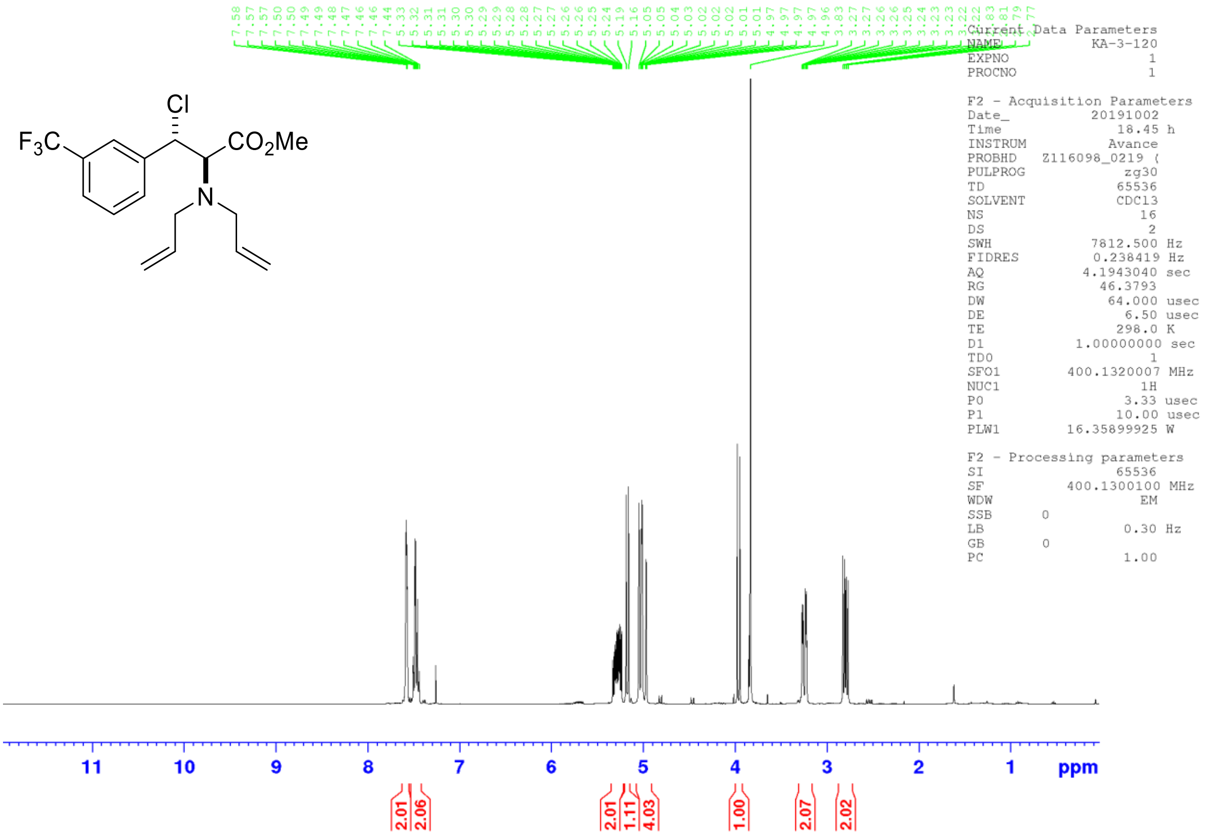
Cl
F3C
CO2Me
N
Current Data Parameters
NAME KA-3-120
EXPNO 1
PROCNO 1
F2 - Acquisition Parameters
Date_ 20191002
Time 18.45 h
INSTRUM Avance
PROBHD Z116098_0219 (
PULPROG zg30
TD 65536
SOLVENT CDCl3
NS 16
DS 2
SWH 7812.500 Hz
FIDRES 0.238419 Hz
AQ 4.1943040 sec
RG 46.3793
DW 64.000 usec
DE 6.50 usec
TE 298.0 K
D1 1.00000000 sec
TD0 1
SFO1 400.1320007 MHz
NUC1 1H
P0 3.33 usec
P1 10.00 usec
PLW1 16.35899925 W
F2 - Processing parameters
SI 65536
SF 400.1300100 MHz
WDW EM
SSB 0
LB 0.30 Hz
GB 0
PC 1.00
11 10 9 8 7 6 5 4 3 2 1 ppm
2.01 2.06 2.01 1.11 4.03 1.00 2.07 2.02

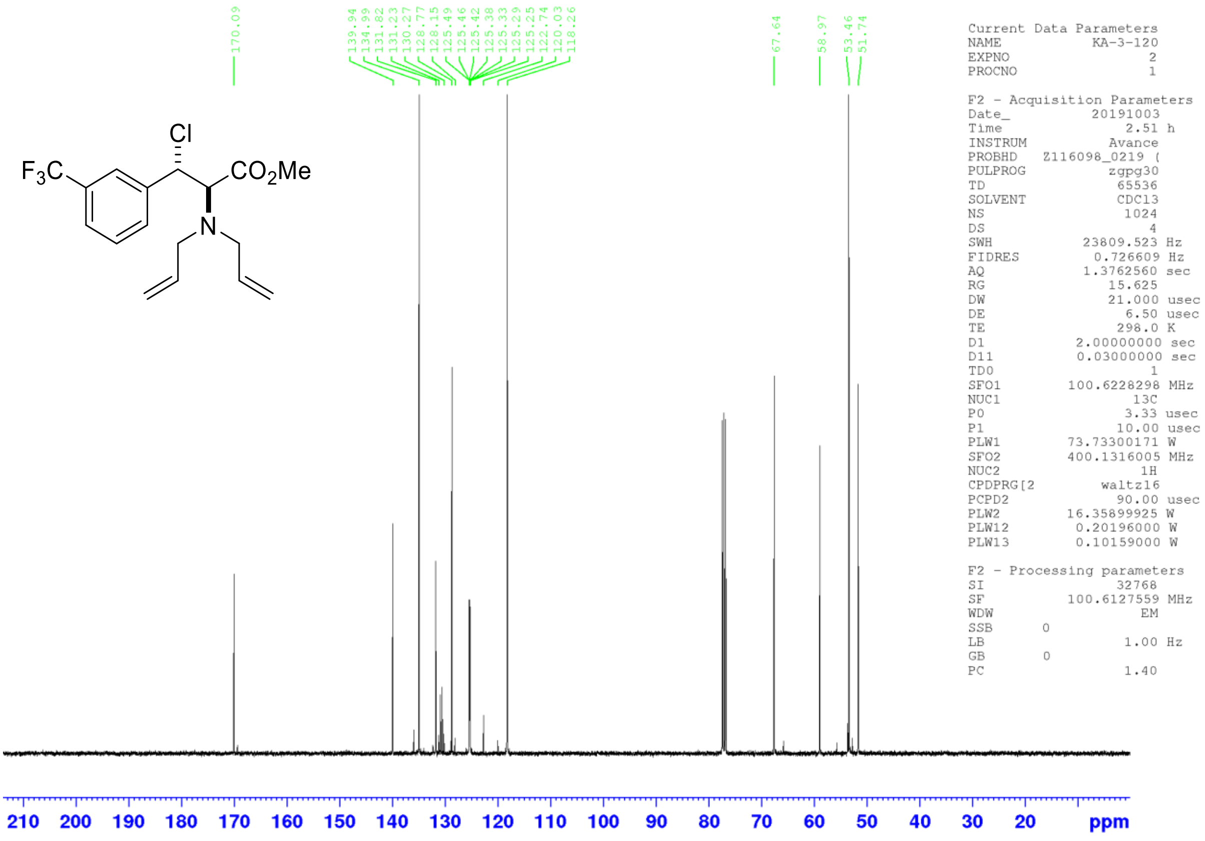
170.09
139.94 134.99 131.82 131.23 130.27 128.77 128.15 125.49 125.46 125.42 125.38 125.33 125.29 125.25 122.74 120.03 118.26
67.64 58.97 53.46 51.74
Cl
F3C
CO2Me
N
Current Data Parameters
NAME KA-3-120
EXPNO 2
PROCNO 1
F2 - Acquisition Parameters
Date_ 20191003
Time 2.51 h
INSTRUM Avance
PROBHD Z116098_0219 (
PULPROG zgpg30
TD 65536
SOLVENT CDCl3
NS 1024
DS 4
SWH 23809.523 Hz
FIDRES 0.726609 Hz
AQ 1.3762560 sec
RG 15.625
DW 21.000 usec
DE 6.50 usec
TE 298.0 K
D1 2.00000000 sec
D11 0.03000000 sec
TD0 1
SFO1 100.6228298 MHz
NUC1 13C
P0 3.33 usec
P1 10.00 usec
PLW1 73.73300171 W
SFO2 400.1316005 MHz
NUC2 1H
CPDPRG[2 waltz16
PCPD2 90.00 usec
PLW2 16.35899925 W
PLW12 0.20196000 W
PLW13 0.10159000 W
F2 - Processing parameters
SI 32768
SF 100.6127559 MHz
WDW EM
SSB 0
LB 1.00 Hz
GB 0
PC 1.40
210 200 190 180 170 160 150 140 130 120 110 100 90 80 70 60 50 40 30 20 ppm

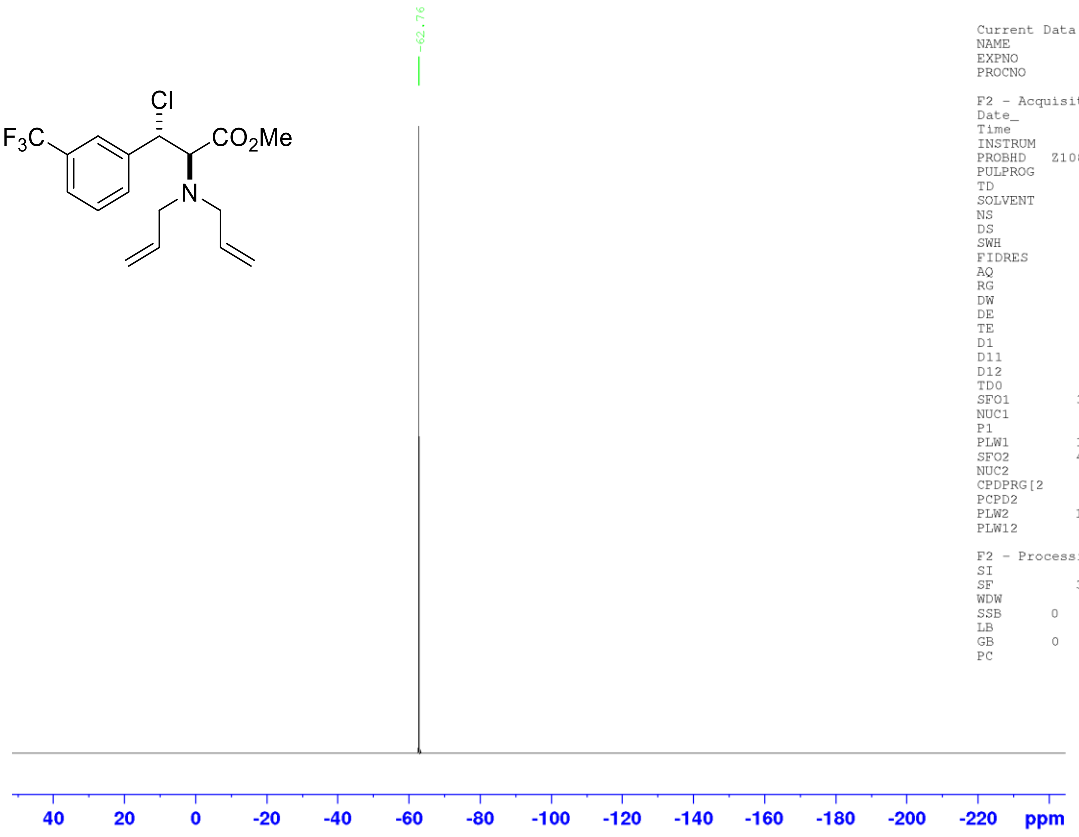


```
Current Data Parameters
NAME                19F
EXPNO                 1
PROCNO                1

F2 - Acquisition Parameters
Date_          20191010
Time              12.32 h
INSTRUM          avf400
PROBHD   Z108618_0533 (
PULPROG        zgfhigqn
TD               261912
SOLVENT           CDCl3
NS                   16
DS                    4
SWH          131578.953 Hz
FIDRES         1.004757 Hz
AQ            0.9952656 sec
RG               205.43
DW                3.800 usec
DE                 6.50 usec
TE                296.9 K
D1           2.00000000 sec
D11          0.03000000 sec
D12          0.00002000 sec
TD0                   1
SFO1        376.5830332 MHz
NUC1                19F
P1                13.50 usec
PLW1        19.00000000 W
SFO2        400.2516010 MHz
NUC2                 1H
CPDPRG[2        waltz16
PCPD2            100.00 usec
PLW2        18.00000000 W
PLW12        0.28125000 W

F2 - Processing parameters
SI               131072
SF          376.6112790 MHz
WDW                  EM
SSB      0
LB                 0.50 Hz
GB       0
PC                 1.00
```

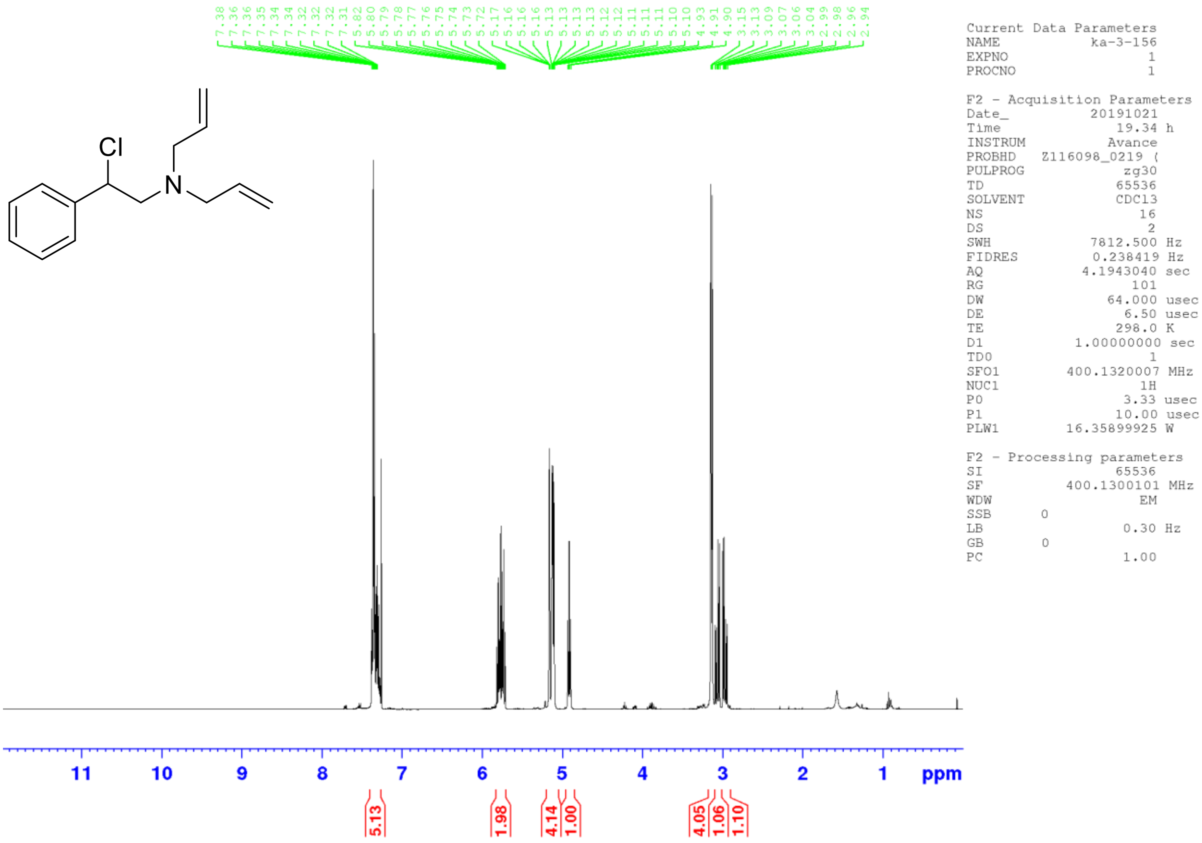


```
Current Data Parameters
NAME           ka-3-156
EXPNO                 1
PROCNO                1

F2 - Acquisition Parameters
Date_          20191021
Time              19.34 h
INSTRUM          Avance
PROBHD   Z116098_0219 (
PULPROG            zg30
TD                65536
SOLVENT           CDCl3
NS                   16
DS                    2
SWH            7812.500 Hz
FIDRES         0.238419 Hz
AQ            4.1943040 sec
RG                  101
DW               64.000 usec
DE                 6.50 usec
TE                298.0 K
D1           1.00000000 sec
TD0                   1
SFO1        400.1320007 MHz
NUC1                 1H
P0                 3.33 usec
P1                10.00 usec
PLW1        16.35899925 W

F2 - Processing parameters
SI                65536
SF          400.1300101 MHz
WDW                  EM
SSB      0
LB                 0.30 Hz
GB       0
PC                 1.00
```

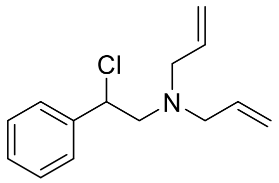
Cl
N

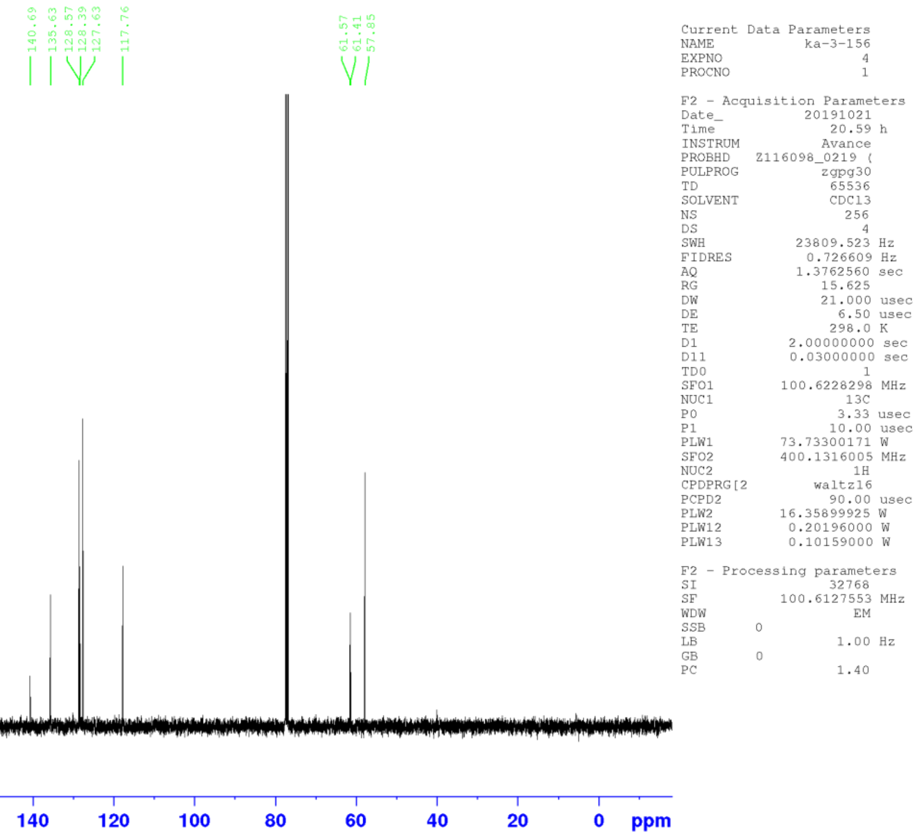
140.69
135.63
128.57
128.39
127.63
117.76
61.57
61.41
57.85
200 180 160 140 120 100 80 60 40 20 0 ppm
Current Data Parameters
NAME ka-3-156
EXPNO 4
PROCNO 1
F2 - Acquisition Parameters
Date_ 20191021
Time 20.59 h
INSTRUM Avance
PROBHD Z116098_0219 (
PULPROG zgpg30
TD 65536
SOLVENT CDCl3
NS 256
DS 4
SWH 23809.523 Hz
FIDRES 0.726609 Hz
AQ 1.3762560 sec
RG 15.625
DW 21.000 usec
DE 6.50 usec
TE 298.0 K
D1 2.00000000 sec
D11 0.03000000 sec
TD0 1
SFO1 100.6228298 MHz
NUC1 13C
P0 3.33 usec
P1 10.00 usec
PLW1 73.73300171 W
SFO2 400.1316005 MHz
NUC2 1H
CPDPRG[2 waltz16
PCPD2 90.00 usec
PLW2 16.35899925 W
PLW12 0.20196000 W
PLW13 0.10159000 W
F2 - Processing parameters
SI 32768
SF 100.6127553 MHz
WDW EM
SSB 0
LB 1.00 Hz
GB 0
PC 1.40

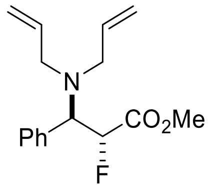
N
Ph
CO2Me
F

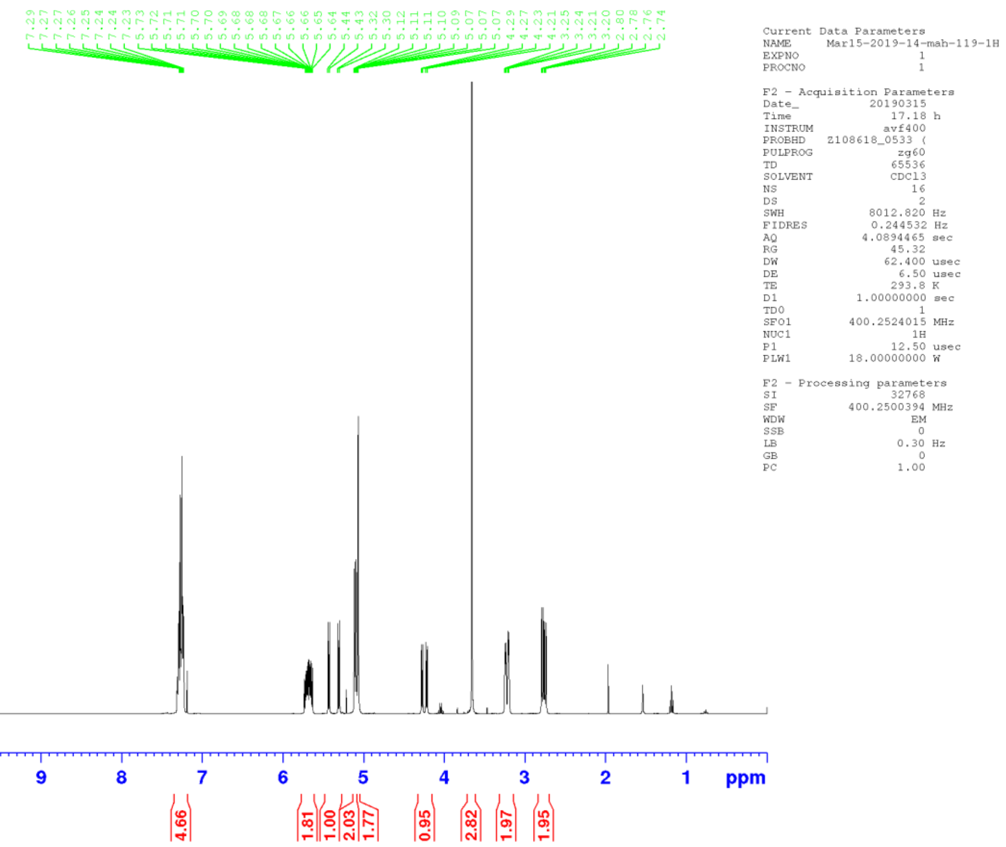
7.29
7.27
7.27
7.26
7.25
7.24
7.24
7.23
5.73
5.72
5.71
5.71
5.70
5.70
5.69
5.68
5.68
5.68
5.67
5.66
5.66
5.65
5.64
5.44
5.43
5.32
5.30
5.12
5.11
5.11
5.10
5.09
5.07
5.07
5.07
4.29
4.27
4.23
4.21
3.25
3.24
3.21
3.20
2.80
2.78
2.76
2.74
11 10 9 8 7 6 5 4 3 2 1 ppm
4.66
1.81
1.00
2.03
1.77
0.95
2.82
1.97
1.95
Current Data Parameters
NAME Mar15-2019-14-mah-119-1H
EXPNO 1
PROCNO 1
F2 - Acquisition Parameters
Date_ 20190315
Time 17.18 h
INSTRUM avf400
PROBHD Z108618_0533 (
PULPROG zg60
TD 65536
SOLVENT CDCl3
NS 16
DS 2
SWH 8012.820 Hz
FIDRES 0.244532 Hz
AQ 4.0894465 sec
RG 45.32
DW 62.400 usec
DE 6.50 usec
TE 293.8 K
D1 1.00000000 sec
TD0 1
SFO1 400.2524015 MHz
NUC1 1H
P1 12.50 usec
PLW1 18.00000000 W
F2 - Processing parameters
SI 32768
SF 400.2500394 MHz
WDW EM
SSB 0
LB 0.30 Hz
GB 0
PC 1.00

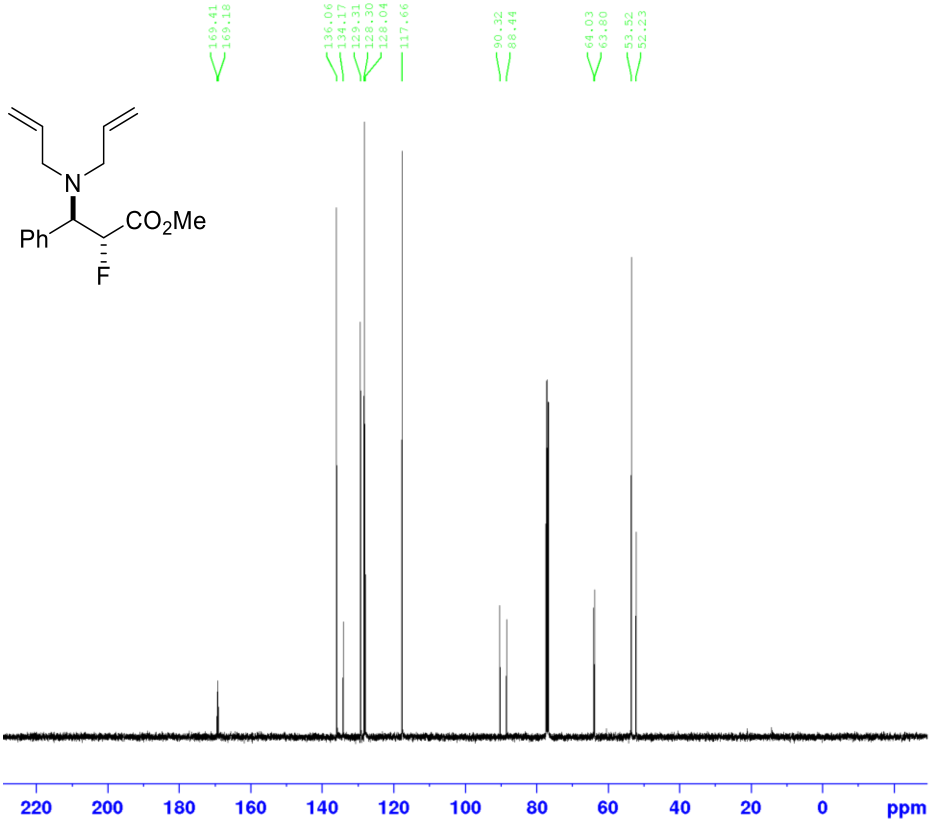


```
Current Data Parameters
NAME     Mar15-2019-14-mah-119-13C
EXPNO                    1
PROCNO                   1

F2 - Acquisition Parameters
Date_             20190316
Time                1.16 h
INSTRUM             avf400
PROBHD   Z108618_0533 (
PULPROG            zgpg30
TD                  32768
SOLVENT             CDCl3
NS                    512
DS                      4
SWH             26041.666 Hz
FIDRES           1.589457 Hz
AQ              0.6291456 sec
RG                 205.43
DW                 19.200 usec
DE                   6.50 usec
TE                  294.4 K
D1             1.00000000 sec
D11            0.03000000 sec
TD0                     1
SFO1          100.6530068 MHz
NUC1                  13C
P1                  10.00 usec
PLW1          36.00000000 W
SFO2          400.2516010 MHz
NUC2                   1H
CPDPRG[2          waltz16
PCPD2              100.00 usec
PLW2          18.00000000 W
PLW12          0.28125000 W
PLW13          0.17465000 W

F2 - Processing parameters
SI                  32768
SF            100.6429430 MHz
WDW                    EM
SSB      0
LB                   1.00 Hz
GB       0
PC                   1.40
```

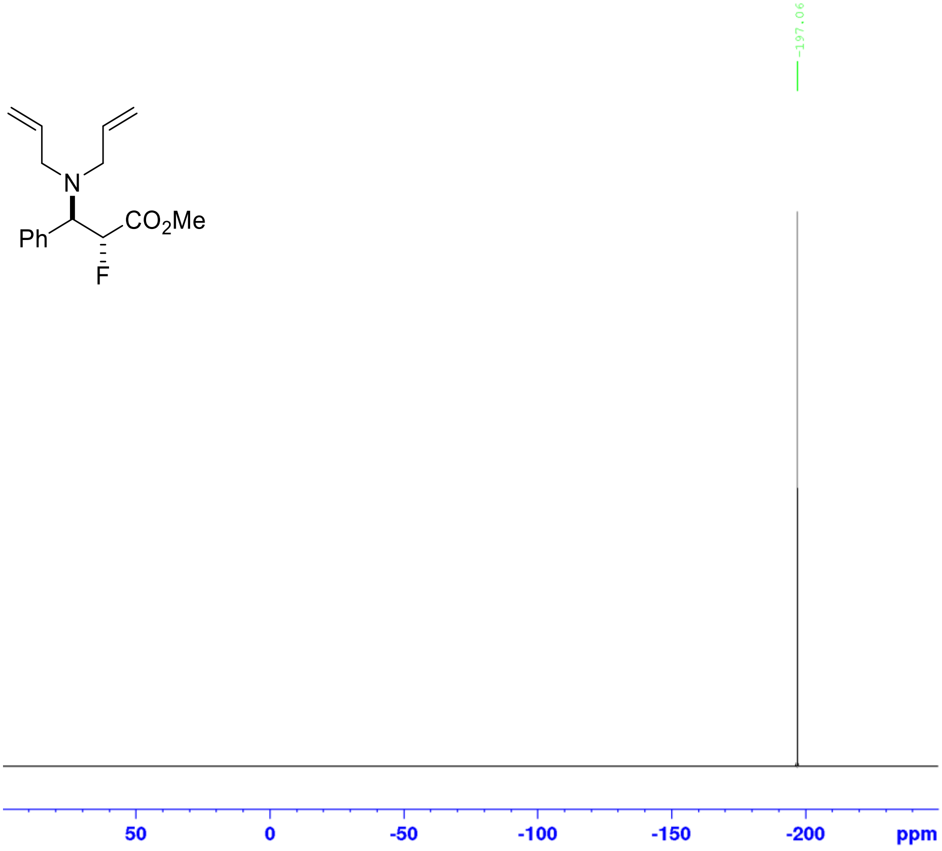


```
Current Data Parameters
NAME     Mar15-2019-14-mah-119-19F
EXPNO                    1
PROCNO                   1

F2 - Acquisition Parameters
Date_             20190315
Time               17.19 h
INSTRUM             avf400
PROBHD   Z108618_0533 (
PULPROG           zgfhigqn
TD                 261912
SOLVENT             CDCl3
NS                     16
DS                      4
SWH            131578.953 Hz
FIDRES           1.004757 Hz
AQ              0.9952656 sec
RG                 205.43
DW                  3.800 usec
DE                   6.50 usec
TE                  294.0 K
D1             2.00000000 sec
D11            0.03000000 sec
D12            0.00002000 sec
TD0                     1
SFO1          376.5830332 MHz
NUC1                  19F
P1                  13.50 usec
PLW1          19.00000000 W
SFO2          400.2516010 MHz
NUC2                   1H
CPDPRG[2          waltz16
PCPD2              100.00 usec
PLW2          18.00000000 W
PLW12          0.28125000 W

F2 - Processing parameters
SI                 131072
SF            376.6112790 MHz
WDW                    EM
SSB      0
LB                   0.50 Hz
GB       0
PC                   1.00
```

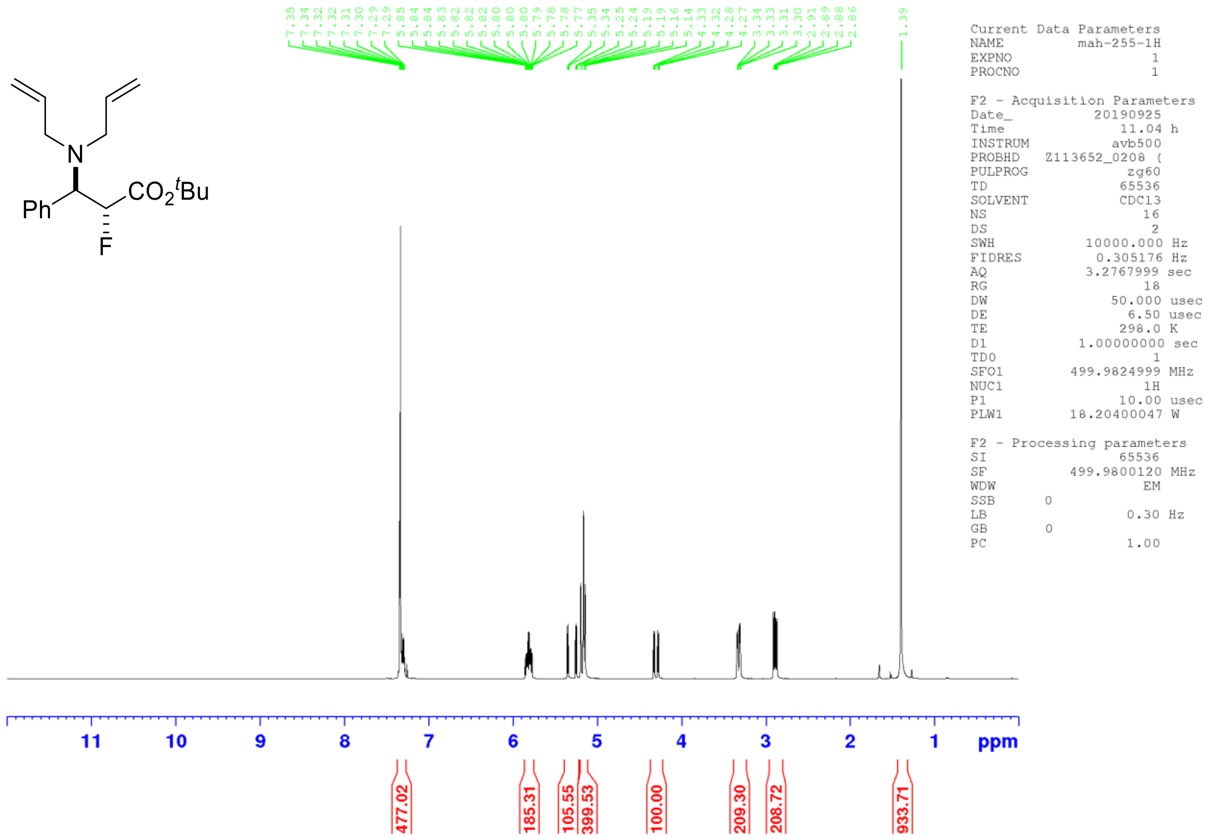
N
Ph
$CO_2{}^tBu$
F
Current Data Parameters
NAME mah-255-1H
EXPNO 1
PROCNO 1
F2 - Acquisition Parameters
Date_ 20190925
Time 11.04 h
INSTRUM avb500
PROBHD Z113652_0208 (
PULPROG zg60
TD 65536
SOLVENT CDCl3
NS 16
DS 2
SWH 10000.000 Hz
FIDRES 0.305176 Hz
AQ 3.2767999 sec
RG 18
DW 50.000 usec
DE 6.50 usec
TE 298.0 K
D1 1.00000000 sec
TD0 1
SFO1 499.9824999 MHz
NUC1 1H
P1 10.00 usec
PLW1 18.20400047 W
F2 - Processing parameters
SI 65536
SF 499.9800120 MHz
WDW EM
SSB 0
LB 0.30 Hz
GB 0
PC 1.00
ppm
477.02
185.31
105.55
399.53
100.00
209.30
208.72
933.71
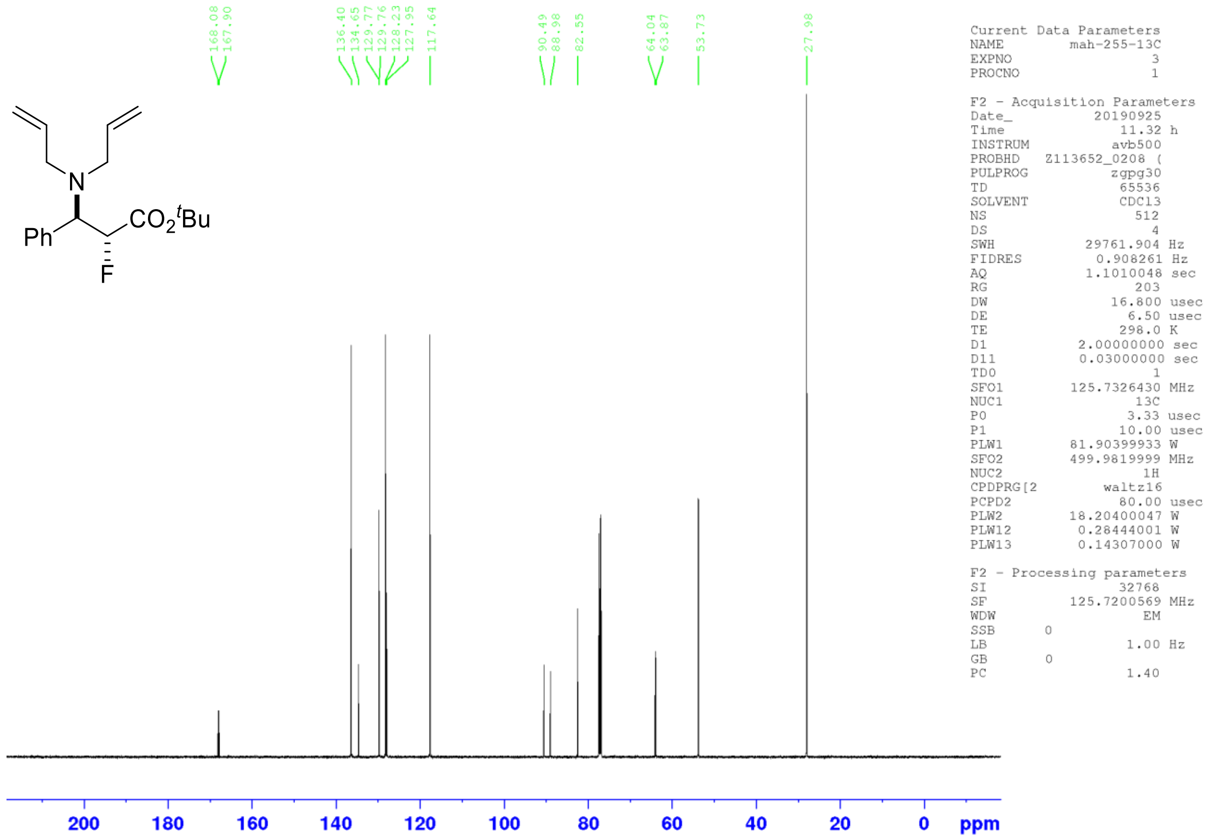
N
Ph
$CO_2{}^tBu$
F
168.08
167.90
136.40
134.65
129.77
129.76
128.23
127.95
117.64
90.49
88.98
82.55
64.04
63.87
53.73
27.98
Current Data Parameters
NAME mah-255-13C
EXPNO 3
PROCNO 1
F2 - Acquisition Parameters
Date_ 20190925
Time 11.32 h
INSTRUM avb500
PROBHD Z113652_0208 (
PULPROG zgpg30
TD 65536
SOLVENT CDCl3
NS 512
DS 4
SWH 29761.904 Hz
FIDRES 0.908261 Hz
AQ 1.1010048 sec
RG 203
DW 16.800 usec
DE 6.50 usec
TE 298.0 K
D1 2.00000000 sec
D11 0.03000000 sec
TD0 1
SFO1 125.7326430 MHz
NUC1 13C
P0 3.33 usec
P1 10.00 usec
PLW1 81.90399933 W
SFO2 499.9819999 MHz
NUC2 1H
CPDPRG[2 waltz16
PCPD2 80.00 usec
PLW2 18.20400047 W
PLW12 0.28444001 W
PLW13 0.14307000 W
F2 - Processing parameters
SI 32768
SF 125.7200569 MHz
WDW EM
SSB 0
LB 1.00 Hz
GB 0
PC 1.40
ppm

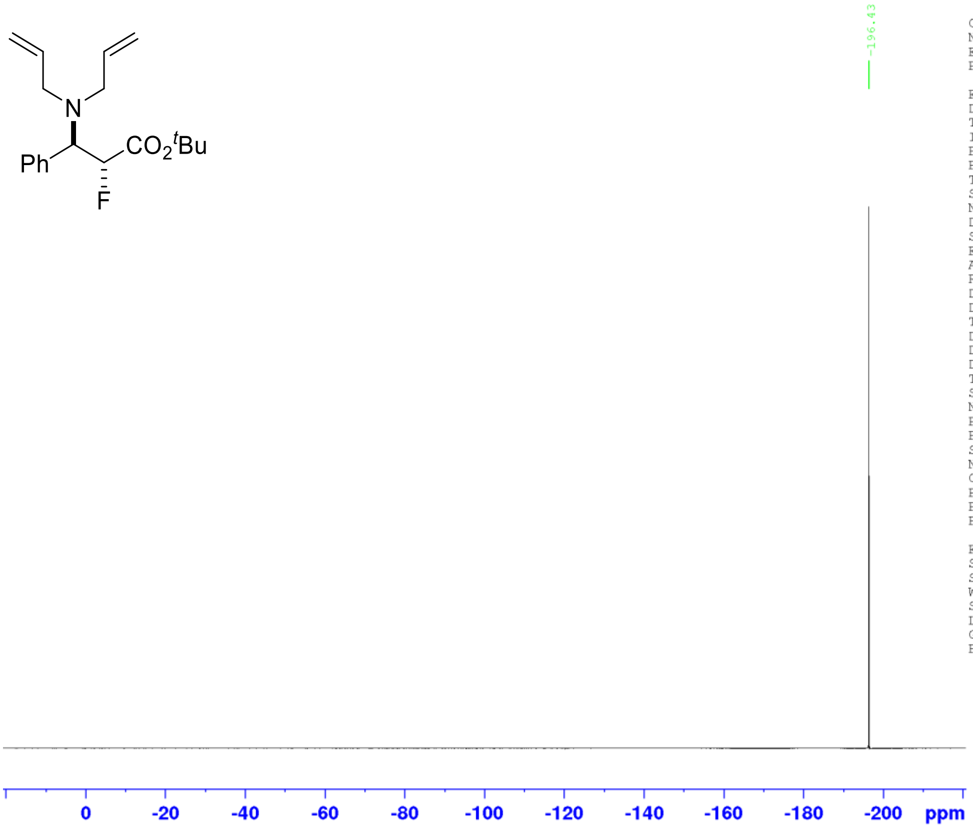

Current Data Parameters
NAME mah-255-19F
EXPNO 5
PROCNO 1

F2 - Acquisition Parameters
Date_ 20190925
Time 11.34 h
INSTRUM avb500
PROBHD Z113652_0208 (
PULPROG zgfhigqn
TD 131072
SOLVENT CDCl3
NS 16
DS 4
SWH 113636.367 Hz
FIDRES 1.733953 Hz
AQ 0.5767168 sec
RG 203
DW 4.400 usec
DE 6.50 usec
TE 298.1 K
D1 1.00000000 sec
D11 0.03000000 sec
D12 0.00002000 sec
TD0 1
SFO1 470.4041915 MHz
NUC1 19F
P1 15.00 usec
PLW1 51.13800049 W
SFO2 499.9819999 MHz
NUC2 1H
CPDPRG[2 waltz16
PCPD2 80.00 usec
PLW2 18.20400047 W
PLW12 0.28444001 W

F2 - Processing parameters
SI 65536
SF 470.4512362 MHz
WDW EM
SSB 0
LB 0.30 Hz
GB 0
PC 1.00

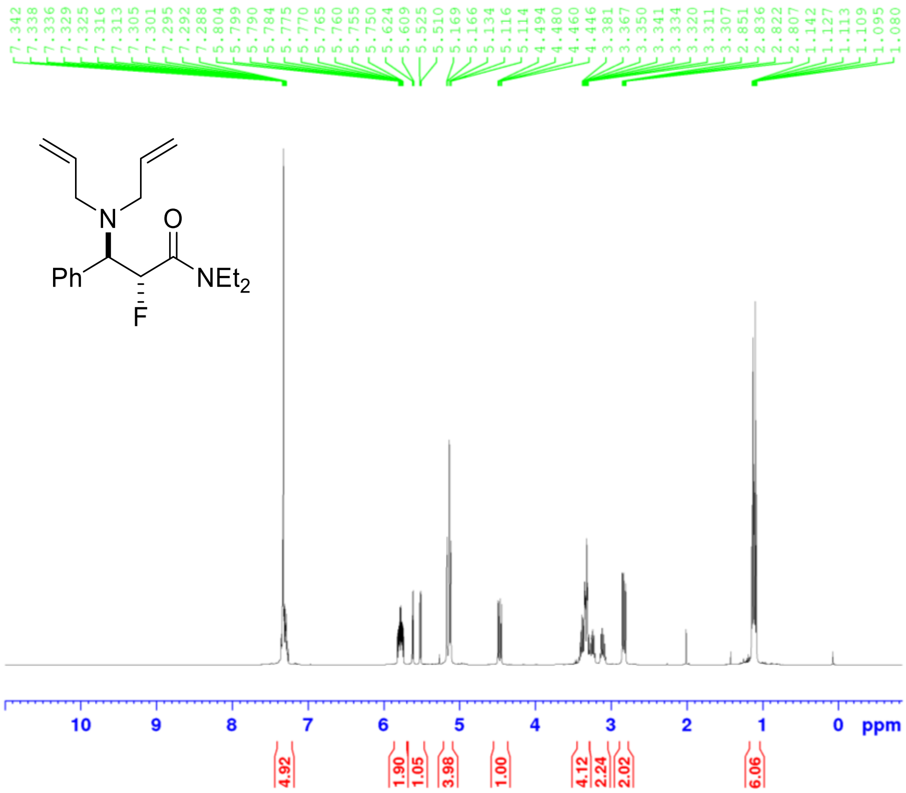

Current Data Parameters
NAME ZC13-34-A-SI
EXPNO 1
PROCNO 1

F2 - Acquisition Parameters
Date_ 20221214
Time 2.28 h
INSTRUM avx500
PROBHD Z119877_0007 (
PULPROG zg60
TD 65536
SOLVENT CDCl3
NS 16
DS 2
SWH 10000.000 Hz
FIDRES 0.305176 Hz
AQ 3.2767999 sec
RG 19.51
DW 50.000 usec
DE 6.50 usec
TE 298.0 K
D1 1.00000000 sec
TD0 1
SFO1 500.3025015 MHz
NUC1 1H
P1 15.00 usec
PLW1 31.53700066 W

F2 - Processing parameters
SI 65536
SF 500.3000120 MHz
WDW EM
SSB 0
LB 0.30 Hz
GB 0
PC 1.00

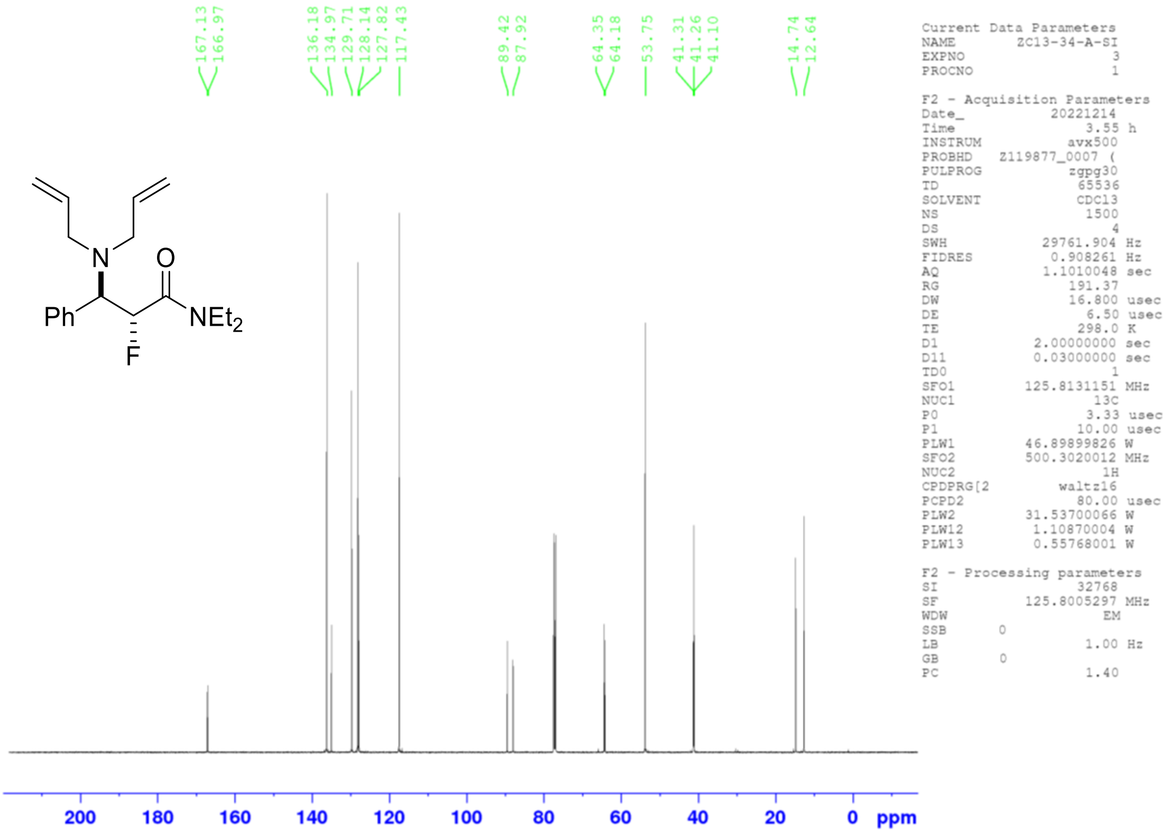
167.13
166.97
136.18
134.97
129.71
128.14
127.82
117.43
89.42
87.92
64.35
64.18
53.75
41.31
41.26
41.10
14.74
12.64
N
O
Ph
NEt2
F
Current Data Parameters
NAME ZC13-34-A-SI
EXPNO 3
PROCNO 1
F2 - Acquisition Parameters
Date_ 20221214
Time 3.55 h
INSTRUM avx500
PROBHD Z119877_0007 (
PULPROG zgpg30
TD 65536
SOLVENT CDCl3
NS 1500
DS 4
SWH 29761.904 Hz
FIDRES 0.908261 Hz
AQ 1.1010048 sec
RG 191.37
DW 16.800 usec
DE 6.50 usec
TE 298.0 K
D1 2.00000000 sec
D11 0.03000000 sec
TD0 1
SFO1 125.8131151 MHz
NUC1 13C
P0 3.33 usec
P1 10.00 usec
PLW1 46.89899826 W
SFO2 500.3020012 MHz
NUC2 1H
CPDPRG[2 waltz16
PCPD2 80.00 usec
PLW2 31.53700066 W
PLW12 1.10870004 W
PLW13 0.55768001 W
F2 - Processing parameters
SI 32768
SF 125.8005297 MHz
WDW EM
SSB 0
LB 1.00 Hz
GB 0
PC 1.40
200
180
160
140
120
100
80
60
40
20
0
ppm

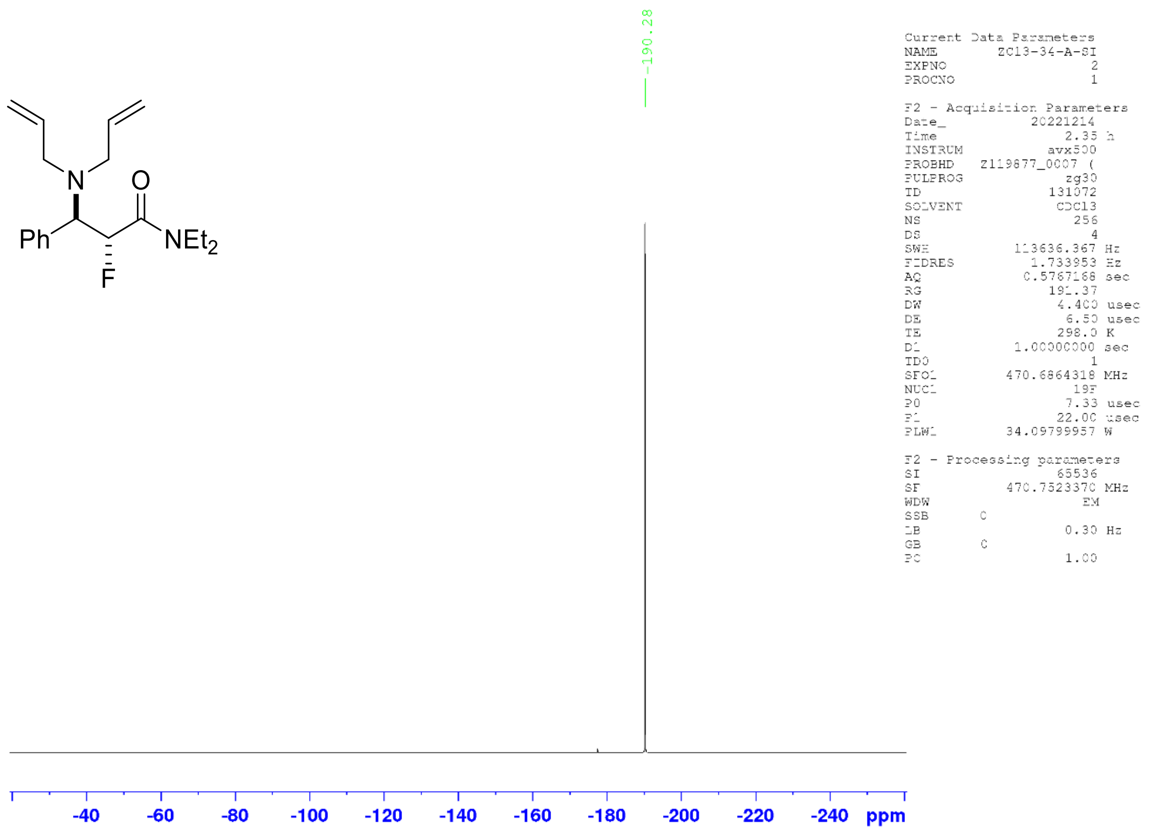
-190.28
N
O
Ph
NEt2
F
Current Data Parameters
NAME ZC13-34-A-SI
EXPNO 2
PROCNO 1
F2 - Acquisition Parameters
Date_ 20221214
Time 2.35 h
INSTRUM avx500
PROBHD Z119877_0007 (
PULPROG zg30
TD 131072
SOLVENT CDCl3
NS 256
DS 4
SWH 113636.367 Hz
FIDRES 1.733953 Hz
AQ 0.5767168 sec
RG 191.37
DW 4.400 usec
DE 6.50 usec
TE 298.0 K
D1 1.00000000 sec
TD0 1
SFO1 470.6864318 MHz
NUC1 19F
P0 7.33 usec
P1 22.00 usec
PLW1 34.09799957 W
F2 - Processing parameters
SI 65536
SF 470.7523370 MHz
WDW EM
SSB 0
LB 0.30 Hz
GB 0
PC 1.00
-40
-60
-80
-100
-120
-140
-160
-180
-200
-220
-240
ppm

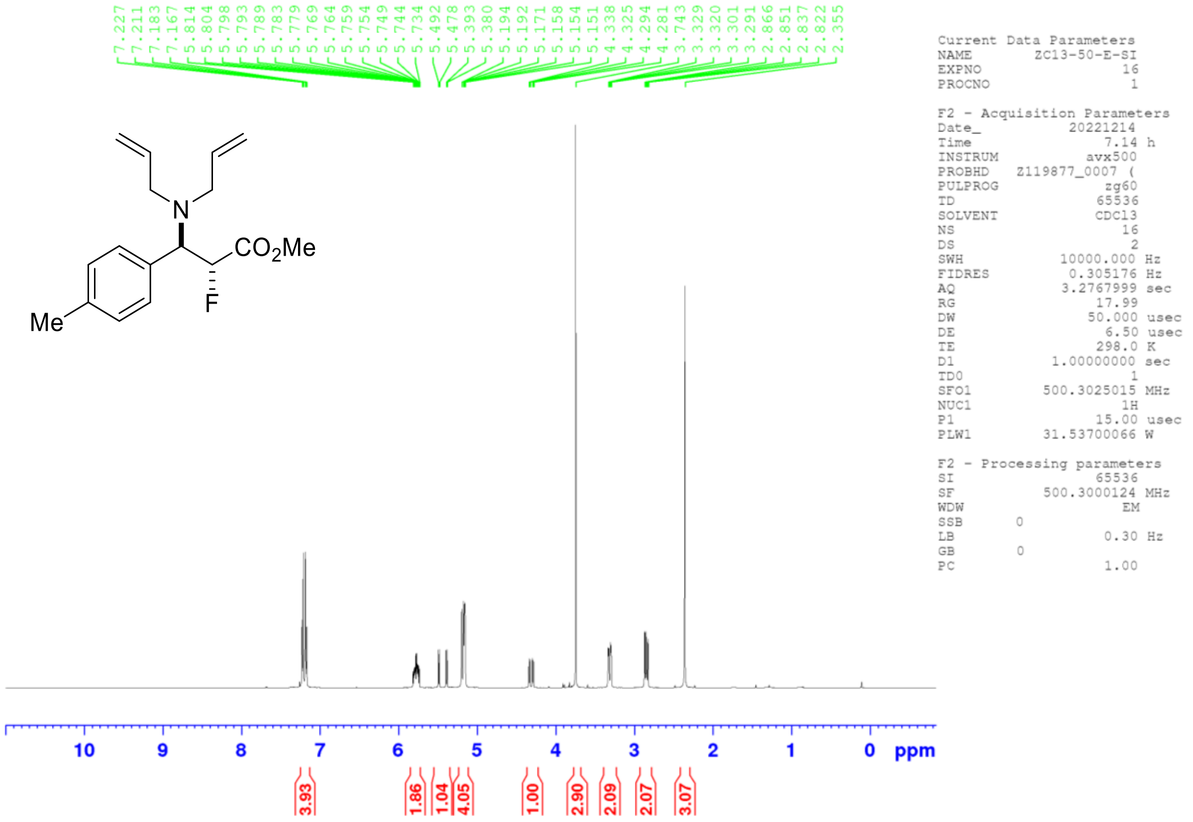

7.227
7.211
7.183
7.167
5.814
5.798
5.793
5.789
5.783
5.779
5.769
5.764
5.759
5.754
5.749
5.744
5.734
5.492
5.478
5.393
5.380
5.194
5.192
5.171
5.158
5.154
5.151
4.338
4.325
4.294
4.281
3.743
3.329
3.320
3.301
3.291
2.866
2.851
2.837
2.822
2.355
N
CO2Me
F
Me
Current Data Parameters
NAME ZC13-50-E-SI
EXPNO 16
PROCNO 1
F2 - Acquisition Parameters
Date_ 20221214
Time 7.14 h
INSTRUM avx500
PROBHD Z119877_0007 (
PULPROG zg60
TD 65536
SOLVENT CDCl3
NS 16
DS 2
SWH 10000.000 Hz
FIDRES 0.305176 Hz
AQ 3.2767999 sec
RG 17.99
DW 50.000 usec
DE 6.50 usec
TE 298.0 K
D1 1.00000000 sec
TD0 1
SFO1 500.3025015 MHz
NUC1 1H
P1 15.00 usec
PLW1 31.53700066 W
F2 - Processing parameters
SI 65536
SF 500.3000124 MHz
WDW EM
SSB 0
LB 0.30 Hz
GB 0
PC 1.00
10 9 8 7 6 5 4 3 2 1 0 ppm
3.93
1.86
1.04
4.05
1.00
2.90
2.09
2.07
3.07

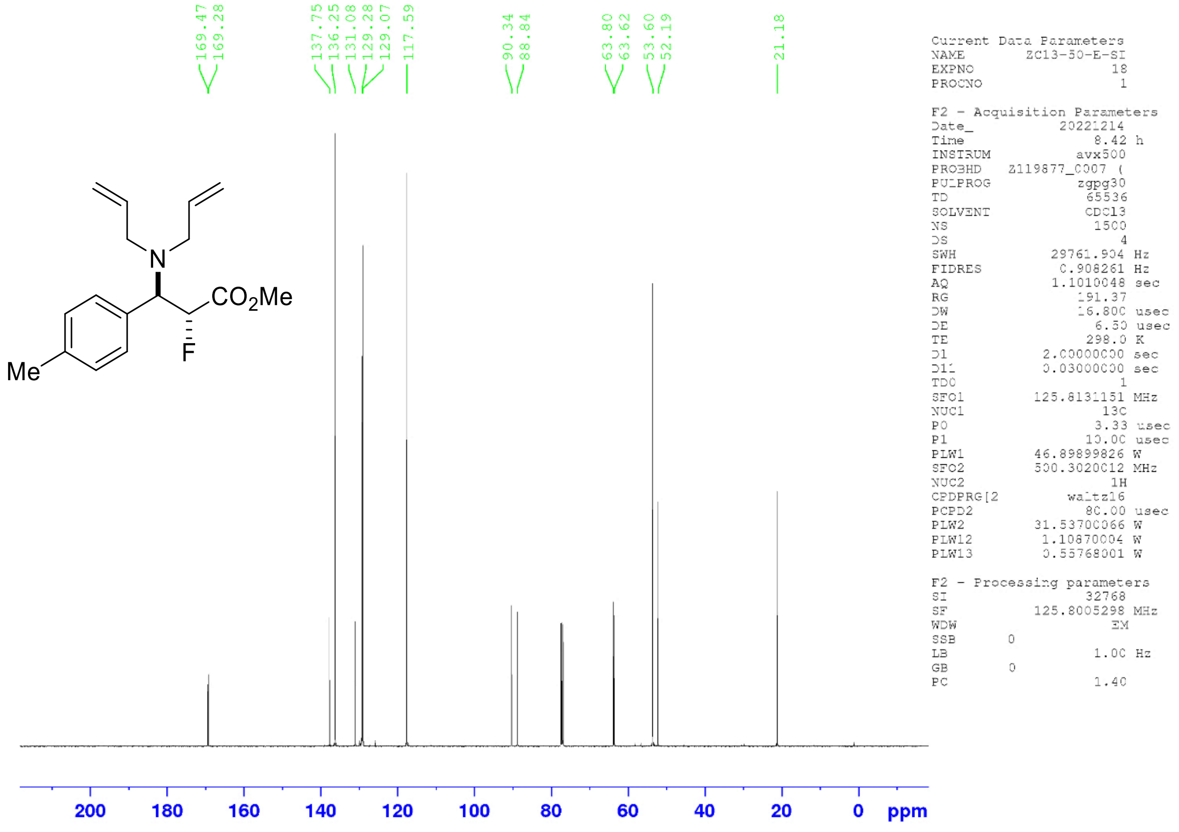

169.47
169.28
137.75
136.25
131.08
129.28
129.07
117.59
90.34
88.84
63.80
63.62
53.60
52.19
21.18
N
CO2Me
F
Me
Current Data Parameters
NAME ZC13-50-E-SI
EXPNO 18
PROCNO 1
F2 - Acquisition Parameters
Date_ 20221214
Time 8.42 h
INSTRUM avx500
PROBHD Z119877_0007 (
PULPROG zgpg30
TD 65536
SOLVENT CDCl3
NS 1500
DS 4
SWH 29761.904 Hz
FIDRES 0.908261 Hz
AQ 1.1010048 sec
RG 191.37
DW 16.800 usec
DE 6.50 usec
TE 298.0 K
D1 2.00000000 sec
D11 0.03000000 sec
TD0 1
SFO1 125.8131151 MHz
NUC1 13C
P0 3.33 usec
P1 10.00 usec
PLW1 46.89899826 W
SFO2 500.3020012 MHz
NUC2 1H
CPDPRG[2 waltz16
PCPD2 80.00 usec
PLW2 31.53700066 W
PLW12 1.10870004 W
PLW13 0.55768001 W
F2 - Processing parameters
SI 32768
SF 125.8005298 MHz
WDW EM
SSB 0
LB 1.00 Hz
GB 0
PC 1.40
200 180 160 140 120 100 80 60 40 20 0 ppm

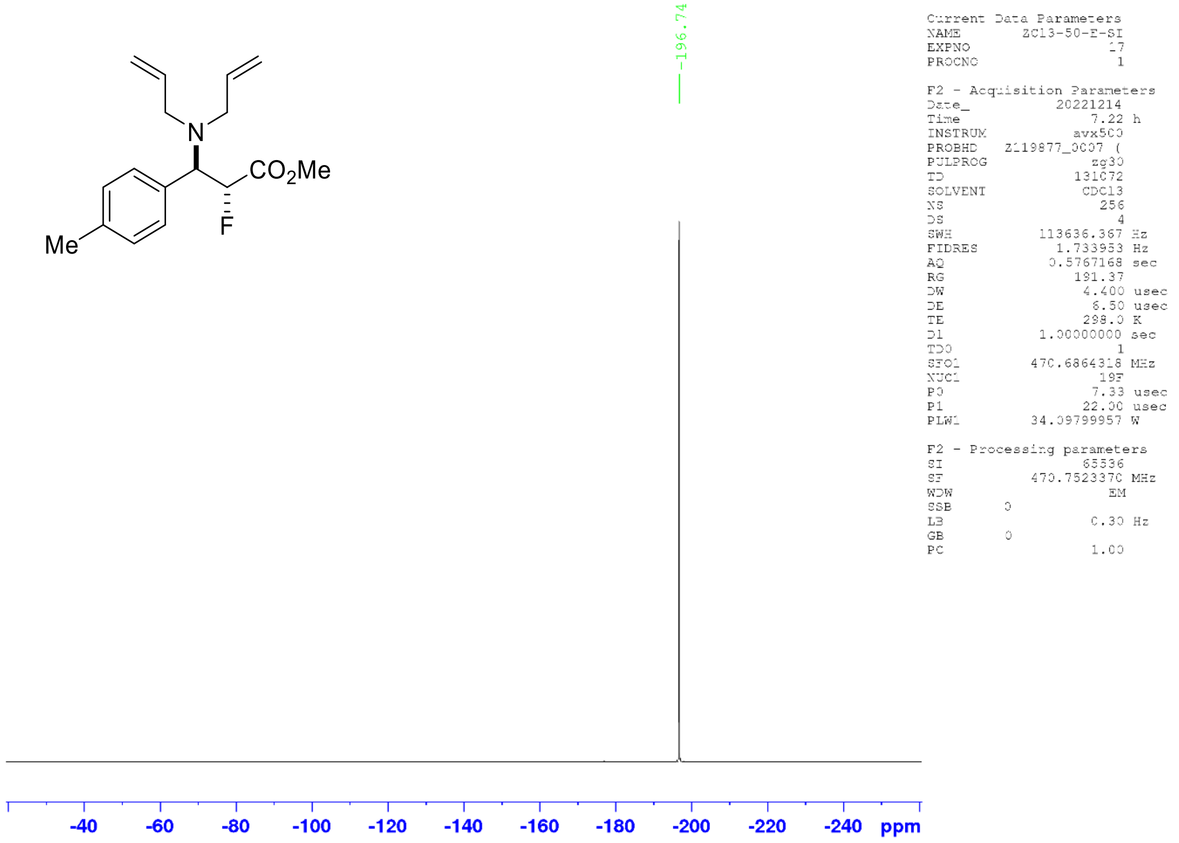

N
CO2Me
F
Me
-196.74
Current Data Parameters
NAME ZC13-50-E-SI
EXPNO 17
PROCNO 1
F2 - Acquisition Parameters
Date_ 20221214
Time 7.22 h
INSTRUM avx500
PROBHD Z119877_0007 (
PULPROG zg30
TD 131072
SOLVENT CDCl3
NS 256
DS 4
SWH 113636.367 Hz
FIDRES 1.733953 Hz
AQ 0.5767168 sec
RG 191.37
DW 4.400 usec
DE 6.50 usec
TE 298.0 K
D1 1.00000000 sec
TD0 1
SFO1 470.6864318 MHz
NUC1 19F
P0 7.33 usec
P1 22.00 usec
PLW1 34.09799957 W
F2 - Processing parameters
SI 65536
SF 470.7523370 MHz
WDW EM
SSB 0
LB 0.30 Hz
GB 0
PC 1.00
-40 -60 -80 -100 -120 -140 -160 -180 -200 -220 -240 ppm

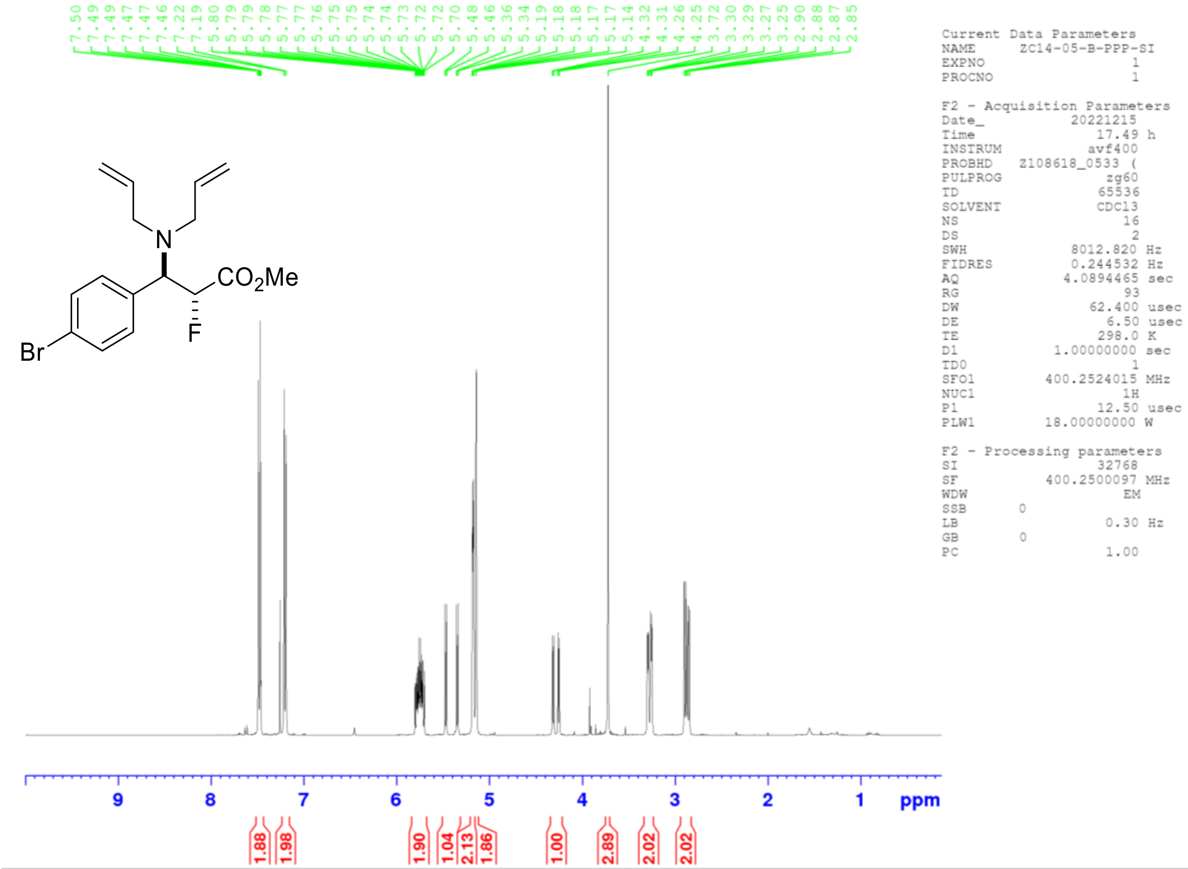

7.50 7.49 7.49 7.47 7.47 7.46 7.22 7.19 5.80 5.79 5.79 5.78 5.77 5.77 5.76 5.75 5.75 5.74 5.74 5.73 5.72 5.72 5.70 5.48 5.46 5.36 5.34 5.19 5.18 5.18 5.17 5.17 5.14 4.32 4.31 4.26 4.25 3.72 3.30 3.29 3.27 3.25 2.90 2.88 2.87 2.85
N
CO2Me
F
Br
Current Data Parameters
NAME ZC14-05-B-PPP-SI
EXPNO 1
PROCNO 1
F2 - Acquisition Parameters
Date_ 20221215
Time 17.49 h
INSTRUM avf400
PROBHD Z108618_0533 (
PULPROG zg60
TD 65536
SOLVENT CDCl3
NS 16
DS 2
SWH 8012.820 Hz
FIDRES 0.244532 Hz
AQ 4.0894465 sec
RG 93
DW 62.400 usec
DE 6.50 usec
TE 298.0 K
D1 1.00000000 sec
TD0 1
SFO1 400.2524015 MHz
NUC1 1H
P1 12.50 usec
PLW1 18.00000000 W
F2 - Processing parameters
SI 32768
SF 400.2500097 MHz
WDW EM
SSB 0
LB 0.30 Hz
GB 0
PC 1.00
9 8 7 6 5 4 3 2 1 ppm
1.88 1.98 1.90 1.04 2.13 1.86 1.00 2.89 2.02 2.02

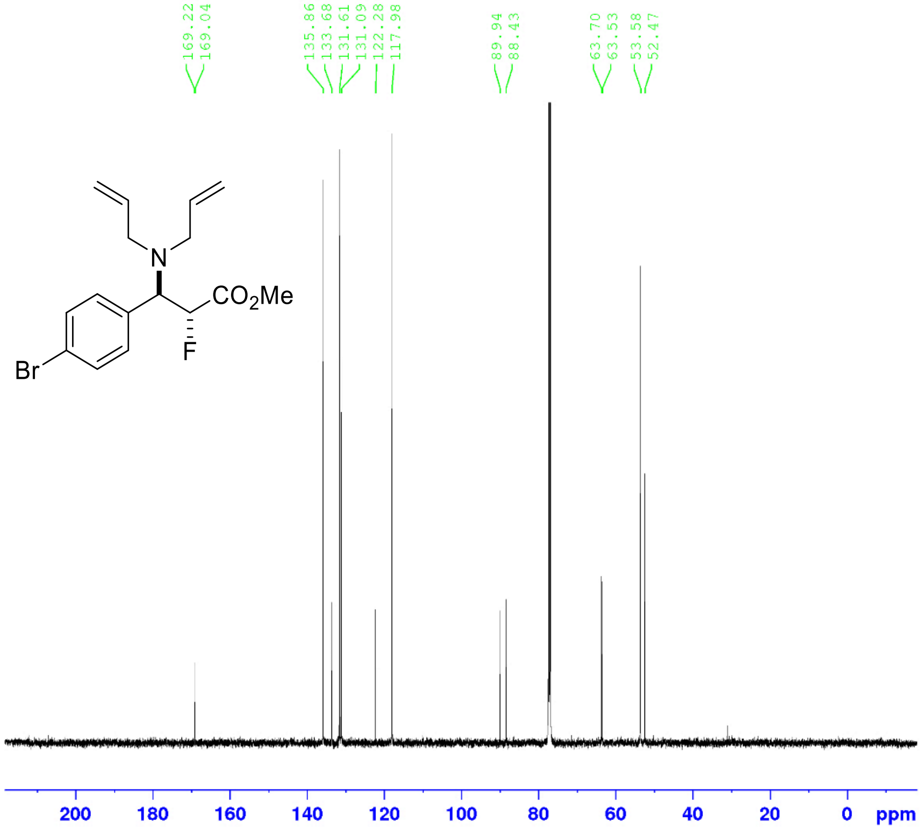


```
Current Data Parameters
NAME                ZC14-05
EXPNO                     6
PROCNO                    1

F2 - Acquisition Parameters
Date_              20221014
Time                   0.33 h
INSTRUM              avx500
PROBHD    Z119877_0007 (
PULPROG             zgpg30
TD                    65536
SOLVENT               CDCl3
NS                     2048
DS                        4
SWH               29761.904 Hz
FIDRES             0.908261 Hz
AQ                1.1010048 sec
RG                   191.37
DW                   16.800 usec
DE                     6.50 usec
TE                    298.0 K
D1               2.00000000 sec
D11              0.03000000 sec
TD0                       1
SFO1            125.8131151 MHz
NUC1                    13C
P0                     3.33 usec
P1                    10.00 usec
PLW1            46.89899826 W
SFO2            500.3020012 MHz
NUC2                     1H
CPDPRG[2            waltz16
PCPD2                 80.00 usec
PLW2            31.53700066 W
PLW12            1.10870004 W
PLW13            0.55768001 W

F2 - Processing parameters
SI                    32768
SF              125.8005189 MHz
WDW                      EM
SSB      0
LB                     1.00 Hz
GB       0
PC                     1.40
```

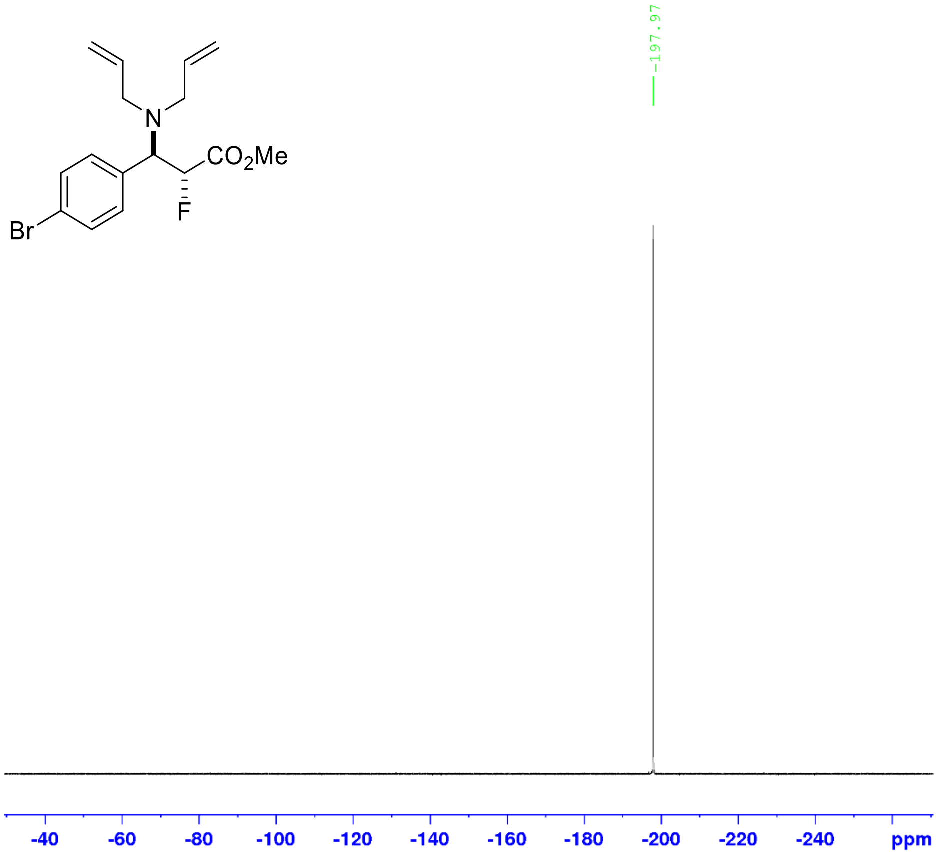


```
Current Data Parameters
NAME                ZC14-05
EXPNO                     5
PROCNO                    1

F2 - Acquisition Parameters
Date_              20221013
Time                  22.44 h
INSTRUM              avx500
PROBHD    Z119877_0007 (
PULPROG                zg30
TD                   131072
SOLVENT               CDCl3
NS                      256
DS                        4
SWH              113636.367 Hz
FIDRES             1.733953 Hz
AQ                0.5767168 sec
RG                   191.37
DW                    4.400 usec
DE                     6.50 usec
TE                    298.0 K
D1               1.00000000 sec
TD0                       1
SFO1            470.6817242 MHz
NUC1                    19F
P0                     7.33 usec
P1                    22.00 usec
PLW1            34.09799957 W

F2 - Processing parameters
SI                    65536
SF              470.7523370 MHz
WDW                      EM
SSB      0
LB                     0.30 Hz
GB       0
PC                     1.00
```

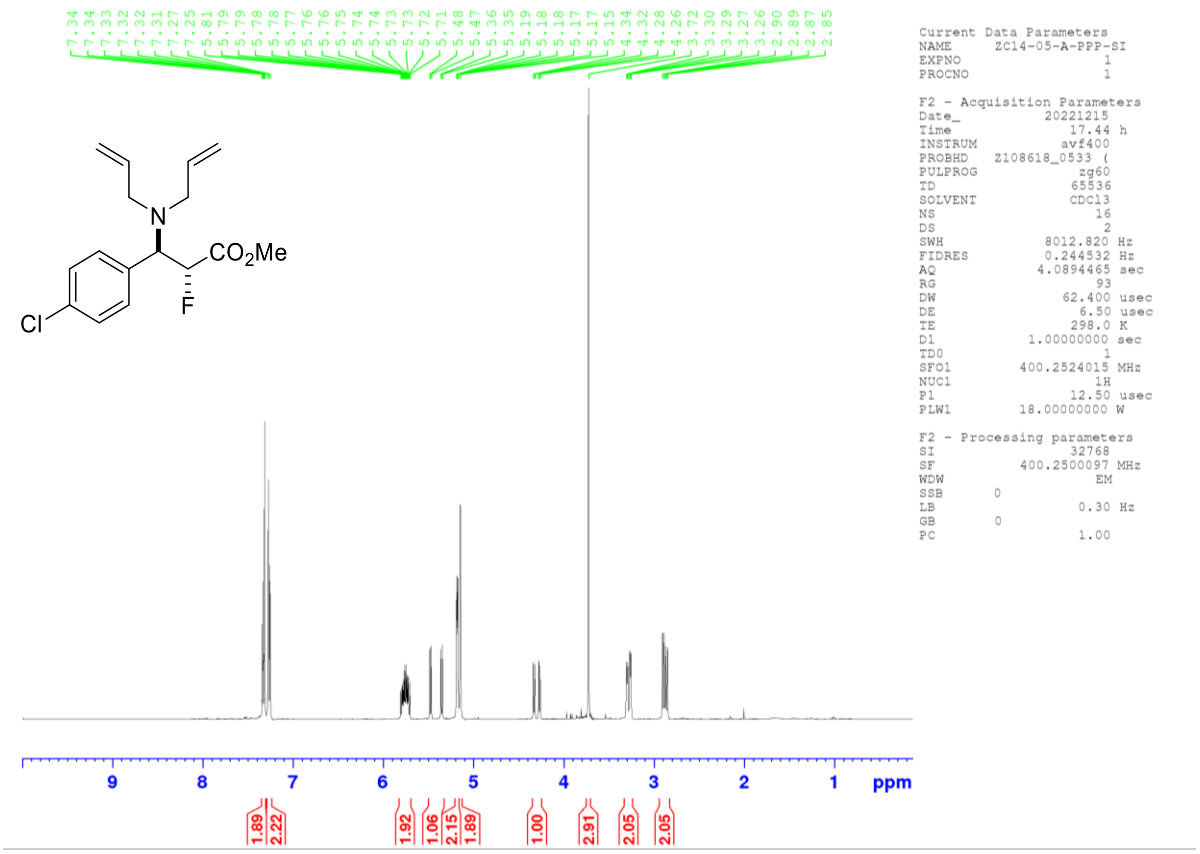

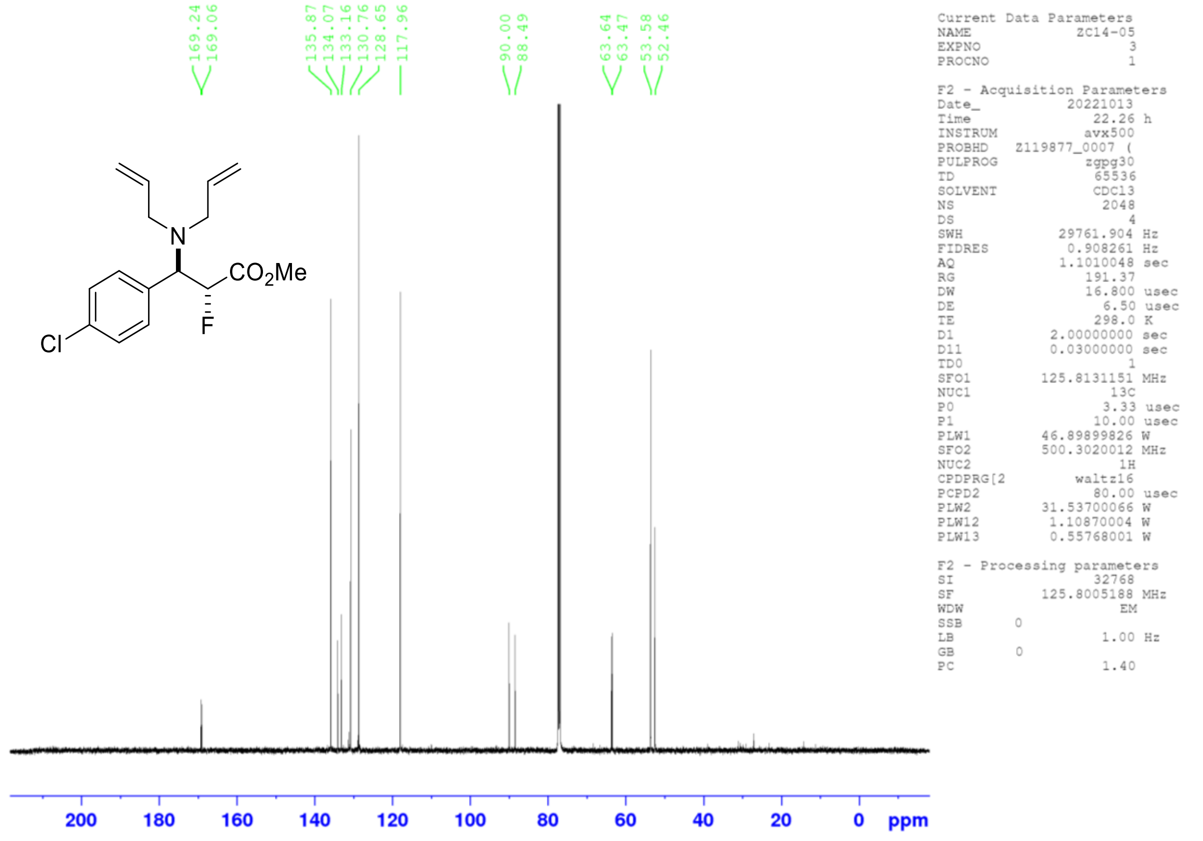

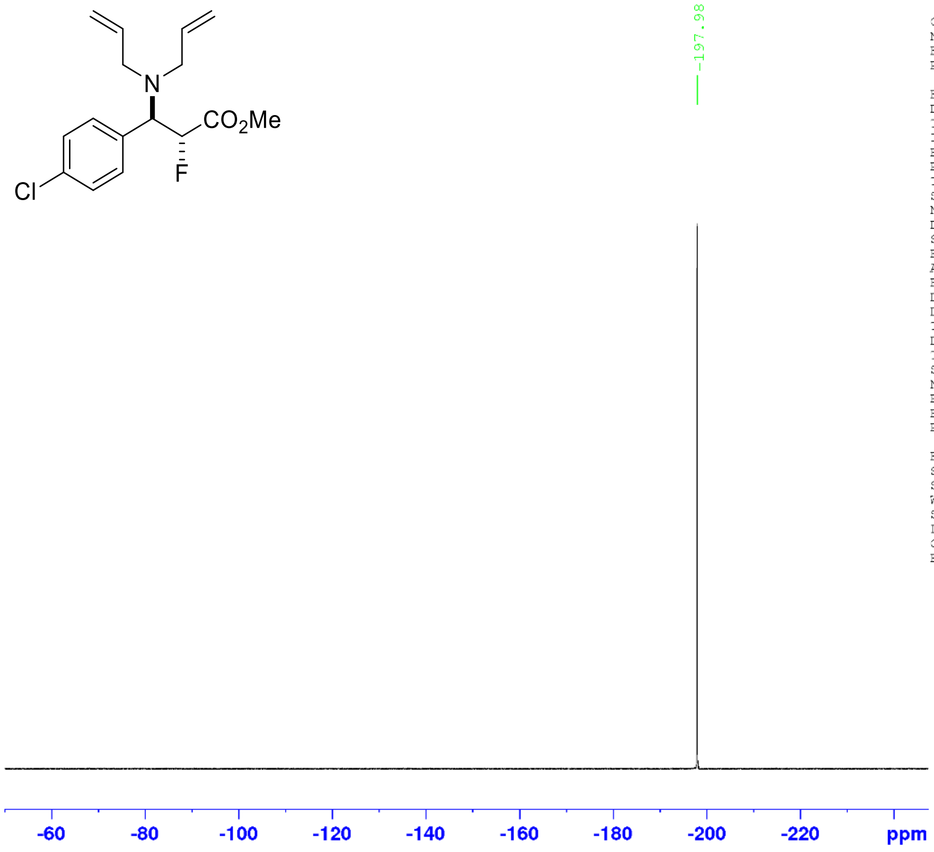


```
Current Data Parameters
NAME              ZC14-05
EXPNO                   2
PROCNO                  1

F2 - Acquisition Parameters
Date_            20221013
Time                20.37 h
INSTRUM            avx500
PROBHD   Z119877_0007 (
PULPROG              zg30
TD                 131072
SOLVENT             CDCl3
NS                    256
DS                      4
SWH            113636.367 Hz
FIDRES           1.733953 Hz
AQ              0.5767168 sec
RG                 191.37
DW                  4.400 usec
DE                   6.50 usec
TE                  298.0 K
D1             1.00000000 sec
TD0                     1
SFO1          470.6817242 MHz
NUC1                  19F
P0                   7.33 usec
P1                  22.00 usec
PLW1          34.09799957 W

F2 - Processing parameters
SI                  65536
SF            470.7523370 MHz
WDW                    EM
SSB      0
LB                   0.30 Hz
GB       0
PC                   1.00
```

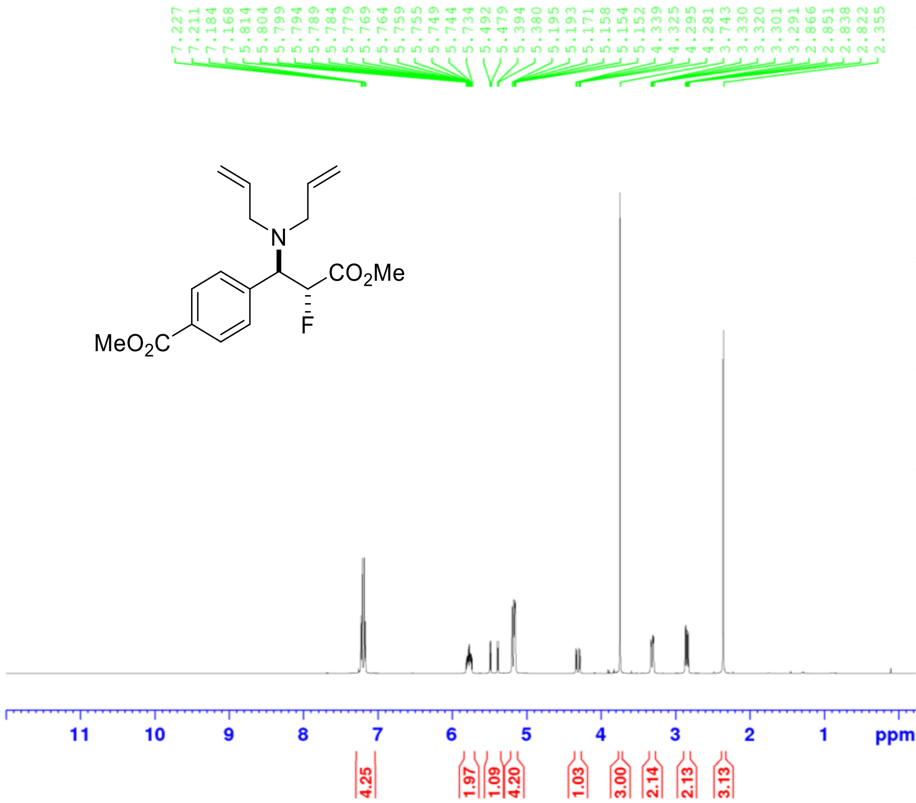


```
Current Data Parameters
NAME          ZC13-50-E-SI
EXPNO                  16
PROCNO                  1

F2 - Acquisition Parameters
Date_            20221214
Time                 7.14 h
INSTRUM            avx500
PROBHD   Z119877_0007 (
PULPROG              zg60
TD                  65536
SOLVENT             CDCl3
NS                     16
DS                      2
SWH             10000.000 Hz
FIDRES           0.305176 Hz
AQ              3.2767999 sec
RG                  17.99
DW                 50.000 usec
DE                   6.50 usec
TE                  298.0 K
D1             1.00000000 sec
TD0                     1
SFO1          500.3025015 MHz
NUC1                   1H
P1                  15.00 usec
PLW1          31.53700066 W

F2 - Processing parameters
SI                  65536
SF            500.3000122 MHz
WDW                    EM
SSB      0
LB                   0.30 Hz
GB       0
PC                   1.00
```

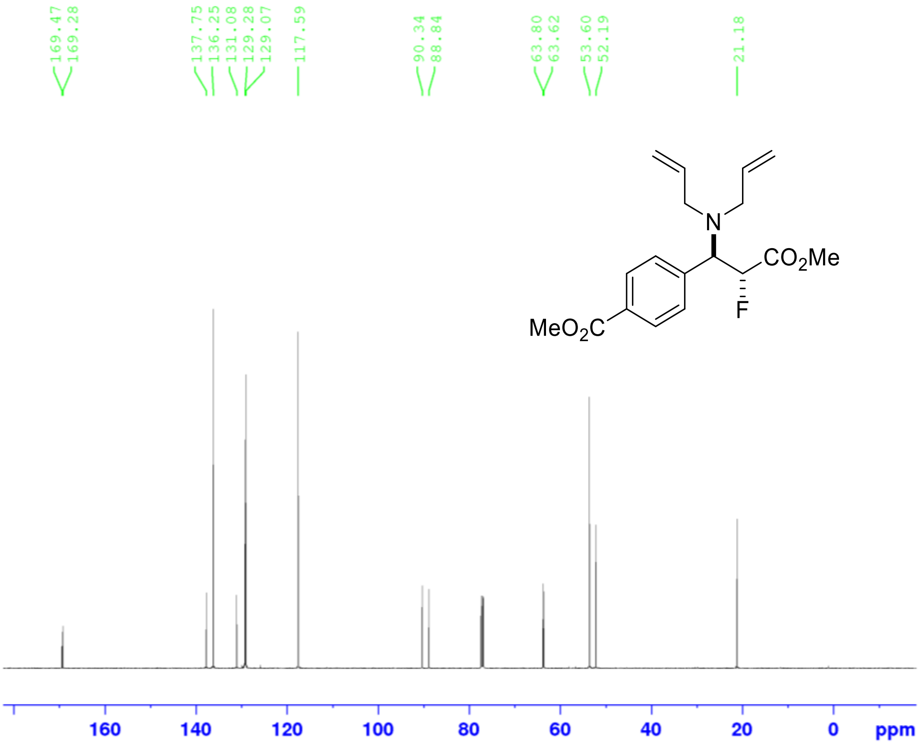


```
Current Data Parameters
NAME          ZC13-50-E-SI
EXPNO                   18
PROCNO                   1

F2 - Acquisition Parameters
Date_             20221214
Time                  8.42 h
INSTRUM             avx500
PROBHD   Z119877_0007 (
PULPROG            zgpg30
TD                  65536
SOLVENT             CDCl3
NS                   1500
DS                      4
SWH             29761.904 Hz
FIDRES           0.908261 Hz
AQ              1.1010048 sec
RG                 191.37
DW                 16.800 usec
DE                   6.50 usec
TE                  298.0 K
D1             2.00000000 sec
D11            0.03000000 sec
TD0                     1
SFO1          125.8131151 MHz
NUC1                  13C
P0                   3.33 usec
P1                  10.00 usec
PLW1          46.89899826 W
SFO2          500.3020012 MHz
NUC2                   1H
CPDPRG[2          waltz16
PCPD2               80.00 usec
PLW2          31.53700066 W
PLW12          1.10870004 W
PLW13          0.55768001 W

F2 - Processing parameters
SI                  32768
SF            125.8005298 MHz
WDW                    EM
SSB      0
LB                   1.00 Hz
GB       0
PC                   1.40
```

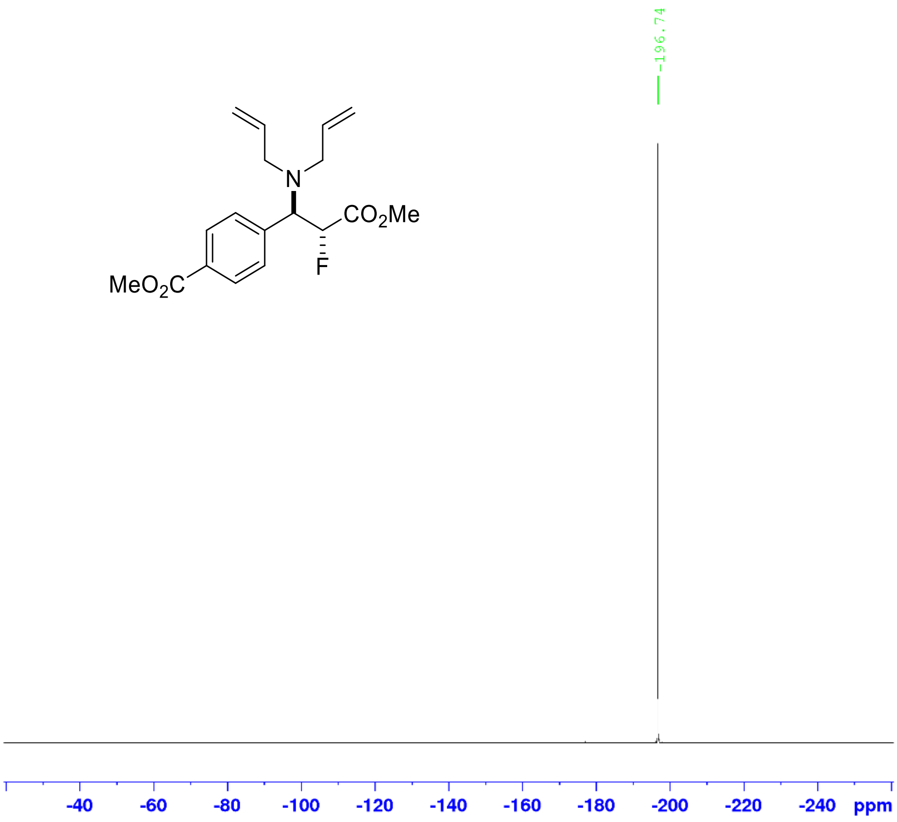


```
Current Data Parameters
NAME          ZC13-50-E-SI
EXPNO                   17
PROCNO                   1

F2 - Acquisition Parameters
Date_             20221214
Time                  7.22 h
INSTRUM             avx500
PROBHD   Z119877_0007 (
PULPROG              zg30
TD                 131072
SOLVENT             CDCl3
NS                    256
DS                      4
SWH            113636.367 Hz
FIDRES           1.733953 Hz
AQ              0.5767168 sec
RG                 191.37
DW                  4.400 usec
DE                   6.50 usec
TE                  298.0 K
D1             1.00000000 sec
TD0                     1
SFO1          470.6864318 MHz
NUC1                  19F
P0                   7.33 usec
P1                  22.00 usec
PLW1          34.09799957 W

F2 - Processing parameters
SI                  65536
SF            470.7523370 MHz
WDW                    EM
SSB      0
LB                   0.30 Hz
GB       0
PC                   1.00
```

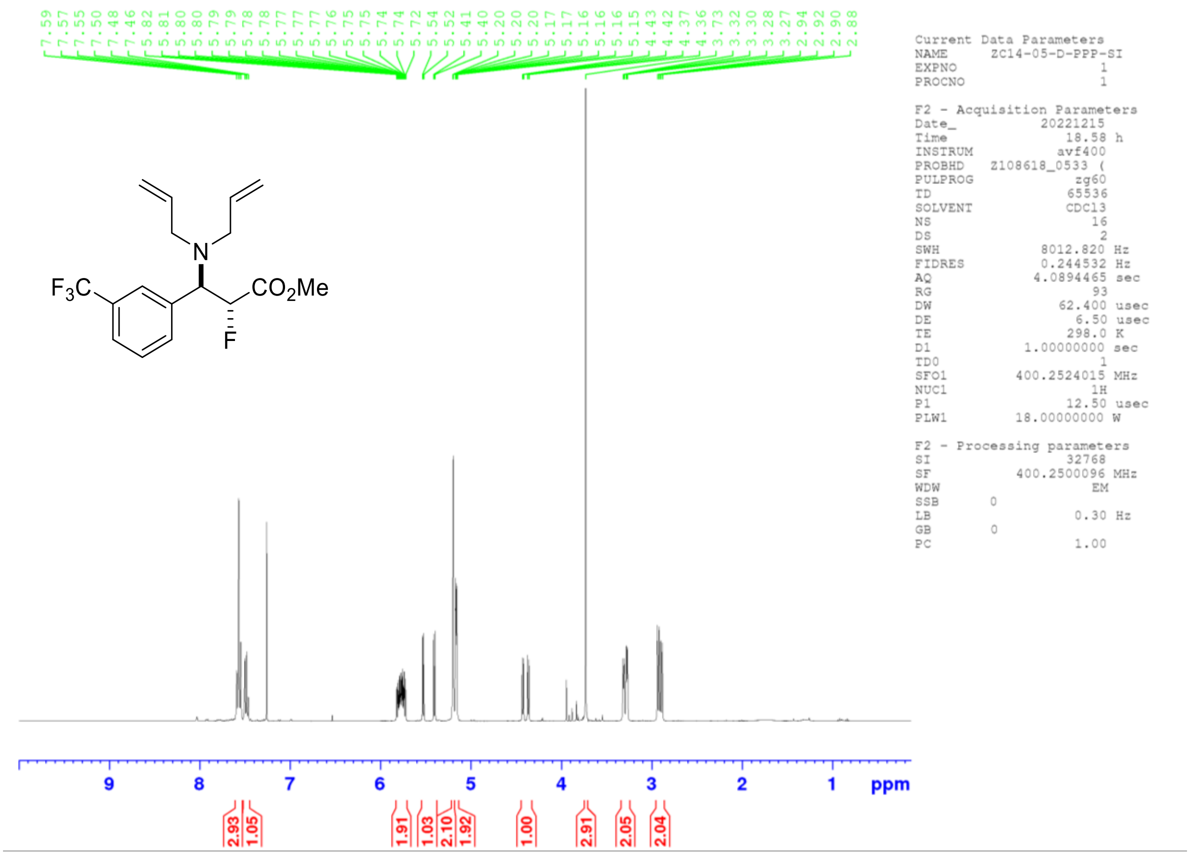

F3C
N
CO2Me
F
Current Data Parameters
NAME ZC14-05-D-PPP-SI
EXPNO 1
PROCNO 1
F2 - Acquisition Parameters
Date_ 20221215
Time 18.58 h
INSTRUM avf400
PROBHD Z108618_0533 (
PULPROG zg60
TD 65536
SOLVENT CDCl3
NS 16
DS 2
SWH 8012.820 Hz
FIDRES 0.244532 Hz
AQ 4.0894465 sec
RG 93
DW 62.400 usec
DE 6.50 usec
TE 298.0 K
D1 1.00000000 sec
TD0 1
SFO1 400.2524015 MHz
NUC1 1H
P1 12.50 usec
PLW1 18.00000000 W
F2 - Processing parameters
SI 32768
SF 400.2500096 MHz
WDW EM
SSB 0
LB 0.30 Hz
GB 0
PC 1.00
9 8 7 6 5 4 3 2 1 ppm

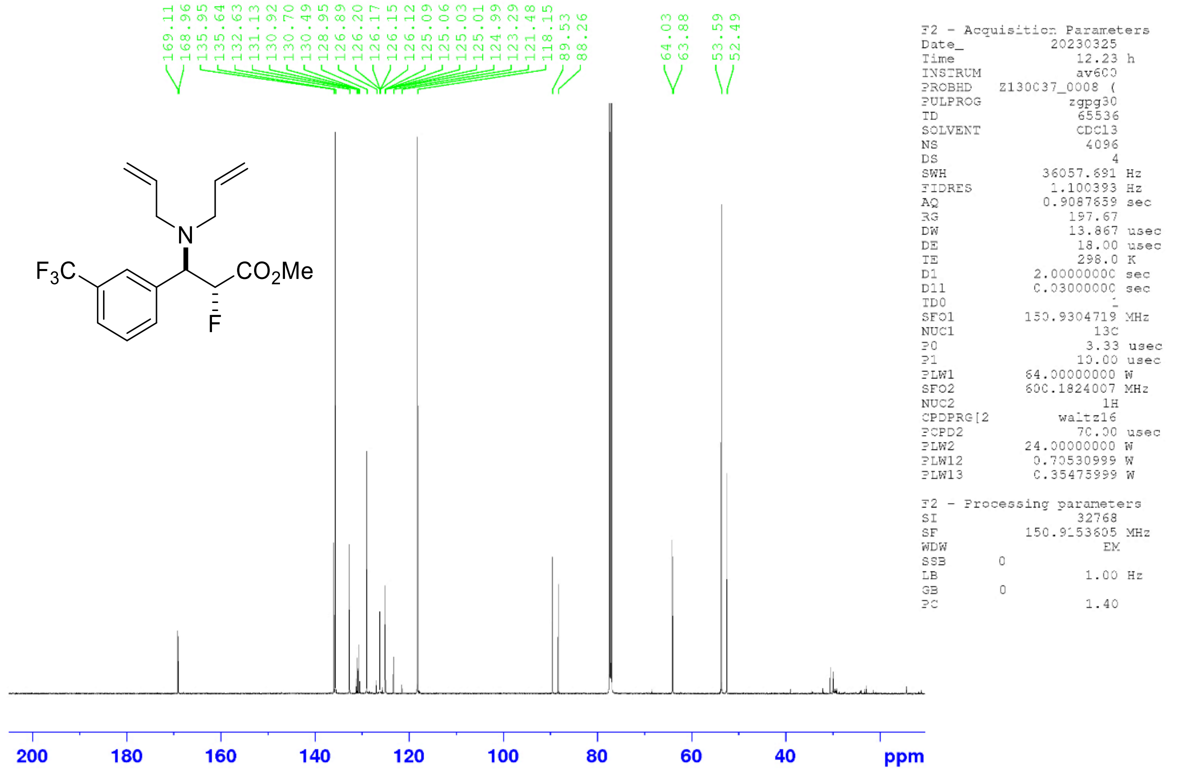

F3C
N
CO2Me
F
F2 - Acquisition Parameters
Date_ 20230325
Time 12.23 h
INSTRUM av600
PROBHD Z130037_0008 (
PULPROG zgpg30
TD 65536
SOLVENT CDCl3
NS 4096
DS 4
SWH 36057.691 Hz
FIDRES 1.100393 Hz
AQ 0.9087659 sec
RG 197.67
DW 13.867 usec
DE 18.00 usec
TE 298.0 K
D1 2.00000000 sec
D11 0.03000000 sec
TD0 1
SFO1 150.9304719 MHz
NUC1 13C
P0 3.33 usec
P1 10.00 usec
PLW1 64.00000000 W
SFO2 600.1824007 MHz
NUC2 1H
CPDPRG[2 waltz16
PCPD2 70.00 usec
PLW2 24.00000000 W
PLW12 0.70530999 W
PLW13 0.35475999 W
F2 - Processing parameters
SI 32768
SF 150.9153605 MHz
WDW EM
SSB 0
LB 1.00 Hz
GB 0
PC 1.40
200 180 160 140 120 100 80 60 40 ppm

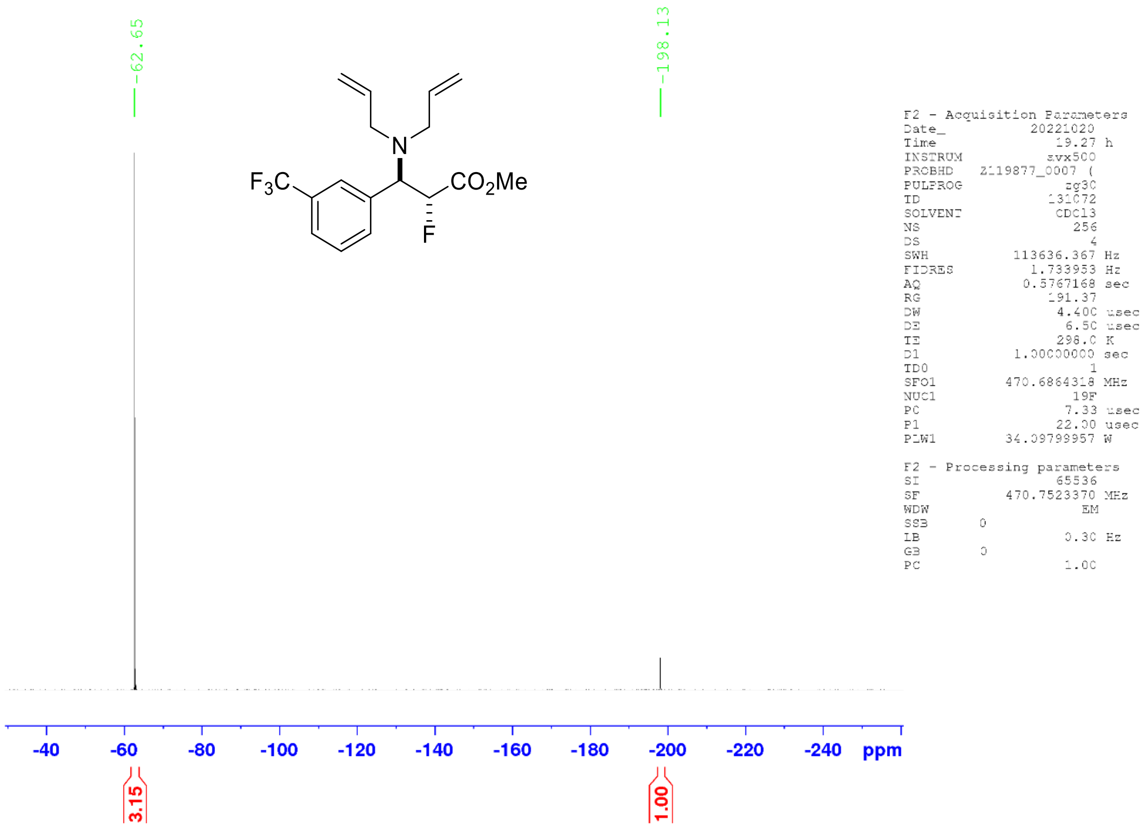

-62.65
-198.13
F_3C
N
CO_2Me
F
F2 - Acquisition Parameters
Date_ 20221020
Time 19.27 h
INSTRUM avx500
PROBHD Z119877_0007 (
PULPROG zg30
TD 131072
SOLVENT CDCl3
NS 256
DS 4
SWH 113636.367 Hz
FIDRES 1.733953 Hz
AQ 0.5767168 sec
RG 191.37
DW 4.400 usec
DE 6.50 usec
TE 298.0 K
D1 1.00000000 sec
TD0 1
SFO1 470.6864318 MHz
NUC1 19F
PC 7.33 usec
P1 22.00 usec
PLW1 34.09799957 W
F2 - Processing parameters
SI 65536
SF 470.7523370 MHz
WDW EM
SSB 0
LB 0.30 Hz
GB 0
PC 1.00
-40 -60 -80 -100 -120 -140 -160 -180 -200 -220 -240 ppm
3.15
1.00

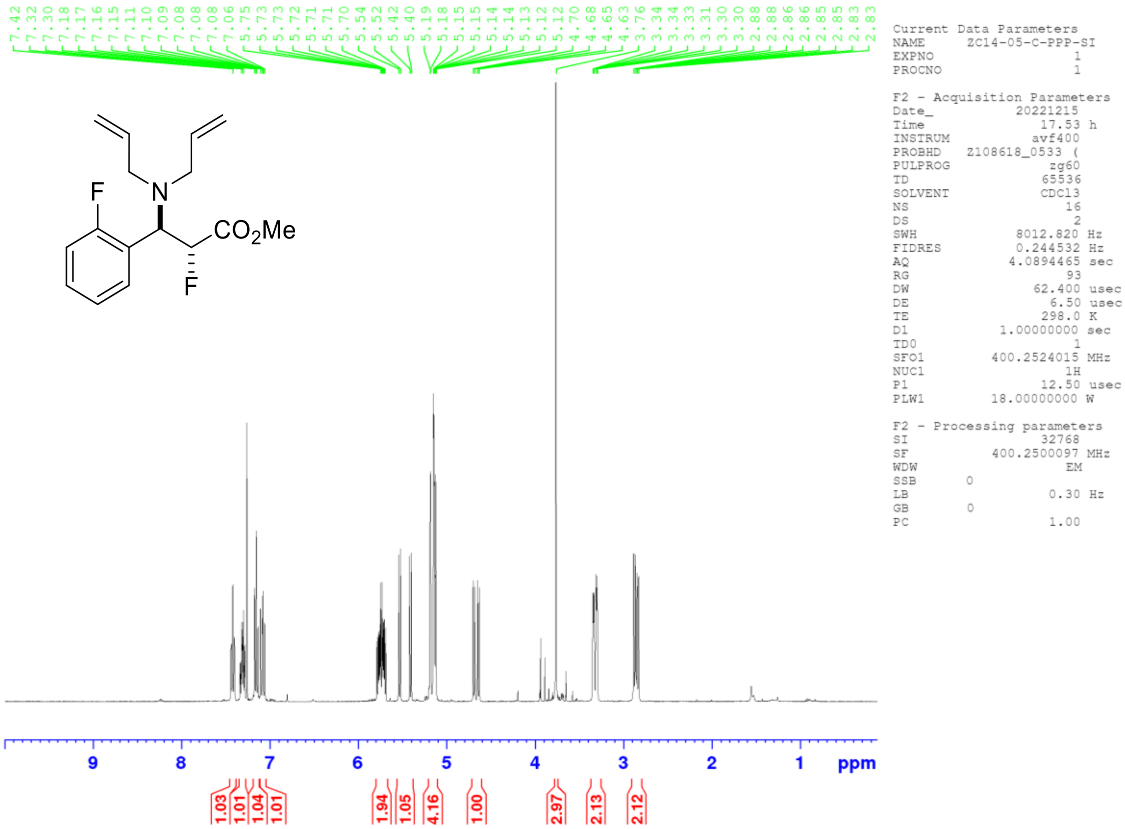

7.42 7.32 7.30 7.18 7.17 7.16 7.15 7.11 7.10 7.09 7.08 7.08 7.06 5.75 5.73 5.73 5.72 5.71 5.71 5.70 5.54 5.52 5.42 5.40 5.19 5.18 5.15 5.15 5.14 5.14 5.13 5.12 5.12 4.70 4.68 4.65 4.63 3.76 3.34 3.34 3.33 3.31 3.30 3.30 2.88 2.88 2.86 2.86 2.85 2.85 2.83 2.83
F
N
CO_2Me
F
Current Data Parameters
NAME ZC14-05-C-PPP-SI
EXPNO 1
PROCNO 1
F2 - Acquisition Parameters
Date_ 20221215
Time 17.53 h
INSTRUM avf400
PROBHD Z108618_0533 (
PULPROG zg60
TD 65536
SOLVENT CDCl3
NS 16
DS 2
SWH 8012.820 Hz
FIDRES 0.244532 Hz
AQ 4.0894465 sec
RG 93
DW 62.400 usec
DE 6.50 usec
TE 298.0 K
D1 1.00000000 sec
TD0 1
SFO1 400.2524015 MHz
NUC1 1H
P1 12.50 usec
PLW1 18.00000000 W
F2 - Processing parameters
SI 32768
SF 400.2500097 MHz
WDW EM
SSB 0
LB 0.30 Hz
GB 0
PC 1.00
9 8 7 6 5 4 3 2 1 ppm
1.03 1.01 1.04 1.01 1.94 1.05 4.16 1.00 2.97 2.13 2.12

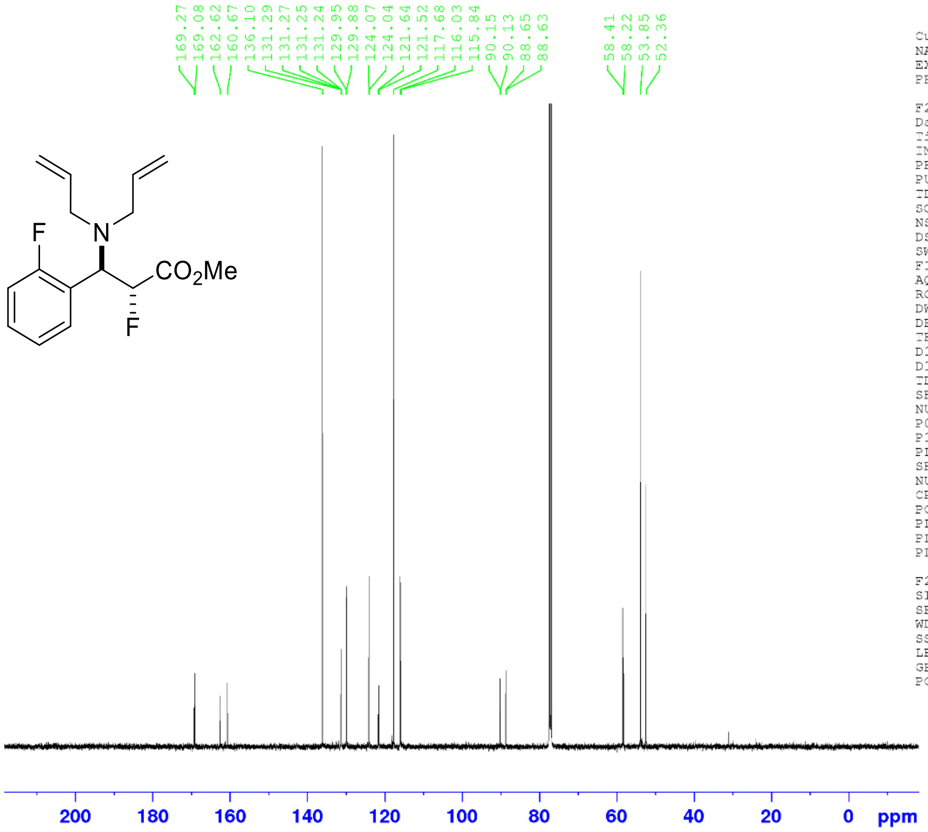

Current Data Parameters
NAME ZC14-05
EXPNO 9
PROCNO 1

F2 - Acquisition Parameters
Date_ 20221014
Time 2.39 h
INSTRUM avx500
PROBHD Z119877_0007 (
PULPROG zgpg30
TD 65536
SOLVENT CDCl3
NS 2048
DS 4
SWH 29761.904 Hz
FIDRES 0.908261 Hz
AQ 1.1010048 sec
RG 191.37
DW 16.800 usec
DE 6.50 usec
TE 298.0 K
D1 2.00000000 sec
D11 0.03000000 sec
TD0 1
SFO1 125.8131151 MHz
NUC1 13C
P0 3.33 usec
P1 10.00 usec
PLW1 46.89899826 W
SFO2 500.3020012 MHz
NUC2 1H
CPDPRG[2 waltz16
PCPD2 80.00 usec
PLW2 31.53700066 W
PLW12 1.10870004 W
PLW13 0.55768001 W

F2 - Processing parameters
SI 32768
SF 125.8005185 MHz
WDW EM
SSB 0
LB 1.00 Hz
GB 0
PC 1.40

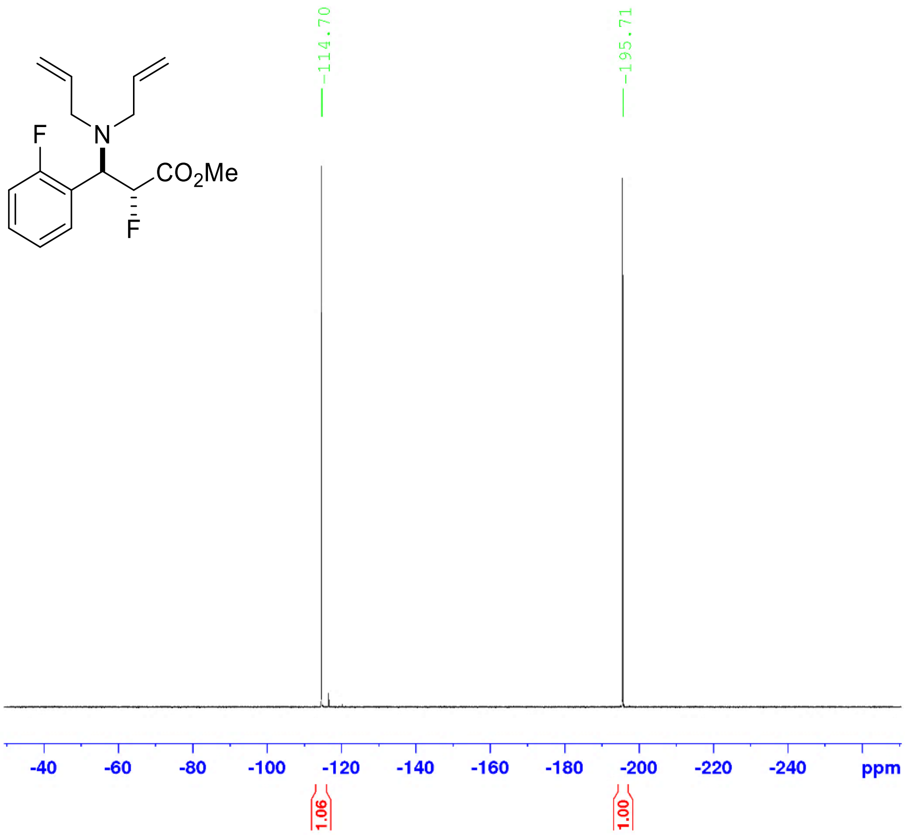

F2 - Acquisition Parameters
Date_ 20221014
Time 0.50 h
INSTRUM avx500
PROBHD Z119877_0007 (
PULPROG zg30
TD 131072
SOLVENT CDCl3
NS 256
DS 4
SWH 113636.367 Hz
FIDRES 1.733953 Hz
AQ 0.5767168 sec
RG 191.37
DW 4.400 usec
DE 6.50 usec
TE 298.0 K
D1 1.00000000 sec
TD0 1
SFO1 470.6817242 MHz
NUC1 19F
P0 7.33 usec
P1 22.00 usec
PLW1 34.09799957 W

F2 - Processing parameters
SI 65536
SF 470.7523370 MHz
WDW EM
SSB 0
LB 0.30 Hz
GB 0
PC 1.00

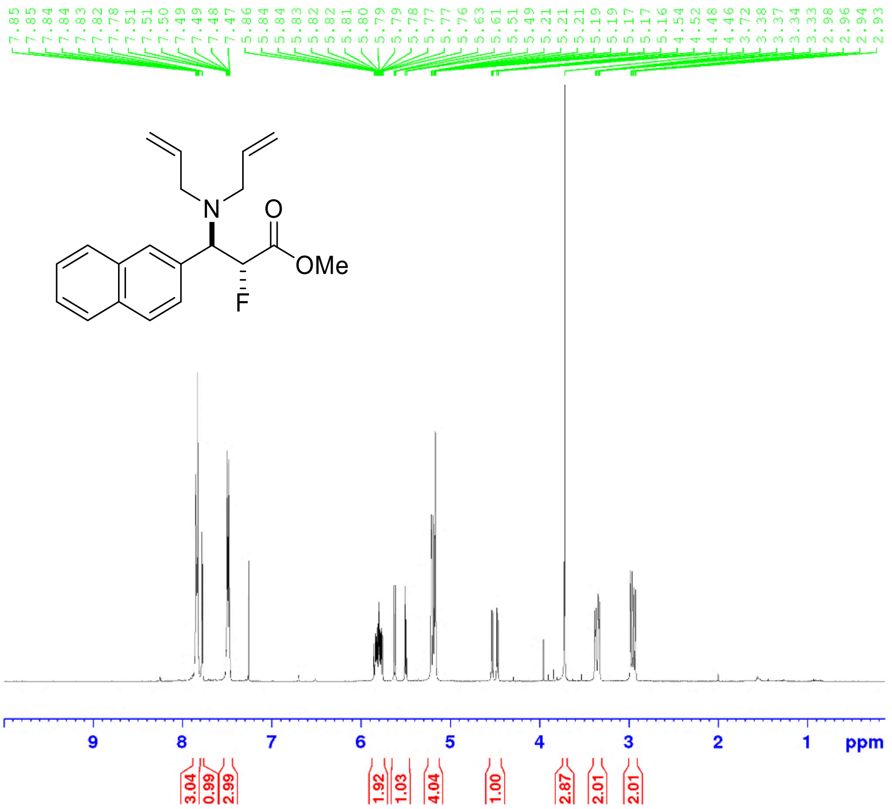

Current Data Parameters
NAME ZC14-05-H-PP-SI
EXPNO 1
PROCNO 1

F2 - Acquisition Parameters
Date_ 20221215
Time 16.59 h
INSTRUM avx400
PROBHD Z108618_0533 (
PULPROG zg60
TD 65536
SOLVENT CDCl3
NS 16
DS 2
SWH 8012.820 Hz
FIDRES 0.244532 Hz
AQ 4.0894465 sec
RG 93
DW 62.400 usec
DE 6.50 usec
TE 298.0 K
D1 1.00000000 sec
TD0 1
SFO1 400.2524015 MHz
NUC1 1H
P1 12.50 usec
PLW1 18.00000000 W

F2 - Processing parameters
SI 32768
SF 400.2500101 MHz
WDW EM
SSB 0
LB 0.30 Hz
GB 0
PC 1.00

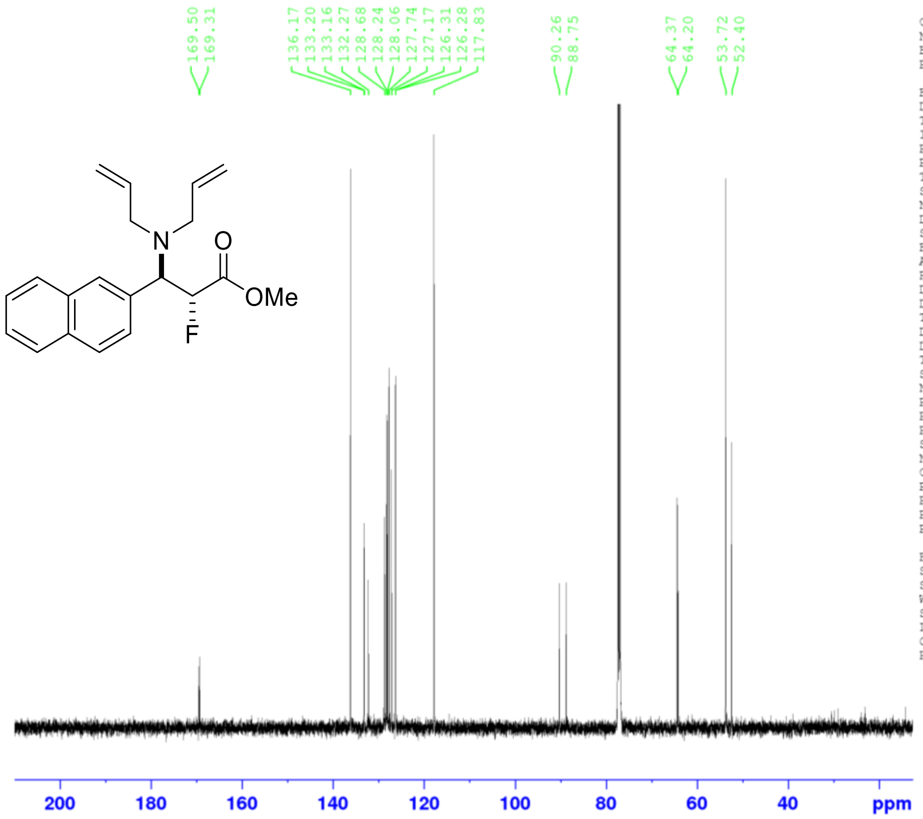

Current Data Parameters
NAME ZC14-05-H-SI
EXPNO 21
PROCNO 1

F2 - Acquisition Parameters
Date_ 20221021
Time 5.37 h
INSTRUM avx500
PROBHD Z119877_0007 (
PULPROG zgpg30
TD 65536
SOLVENT CDCl3
NS 2048
DS 4
SWH 29761.904 Hz
FIDRES 0.908261 Hz
AQ 1.1010048 sec
RG 191.37
DW 16.800 usec
DE 6.50 usec
TE 298.0 K
D1 2.00000000 sec
D11 0.03000000 sec
TD0 1
SFO1 125.8131151 MHz
NUC1 13C
P0 3.33 usec
P1 10.00 usec
PLW1 46.89899826 W
SFO2 500.3020012 MHz
NUC2 1H
CPDPRG[2 waltz16
PCPD2 80.00 usec
PLW2 31.53700066 W
PLW12 1.10870004 W
PLW13 0.55768001 W

F2 - Processing parameters
SI 32768
SF 125.8005189 MHz
WDW EM
SSB 0
LB 1.00 Hz
GB 0
PC 1.40

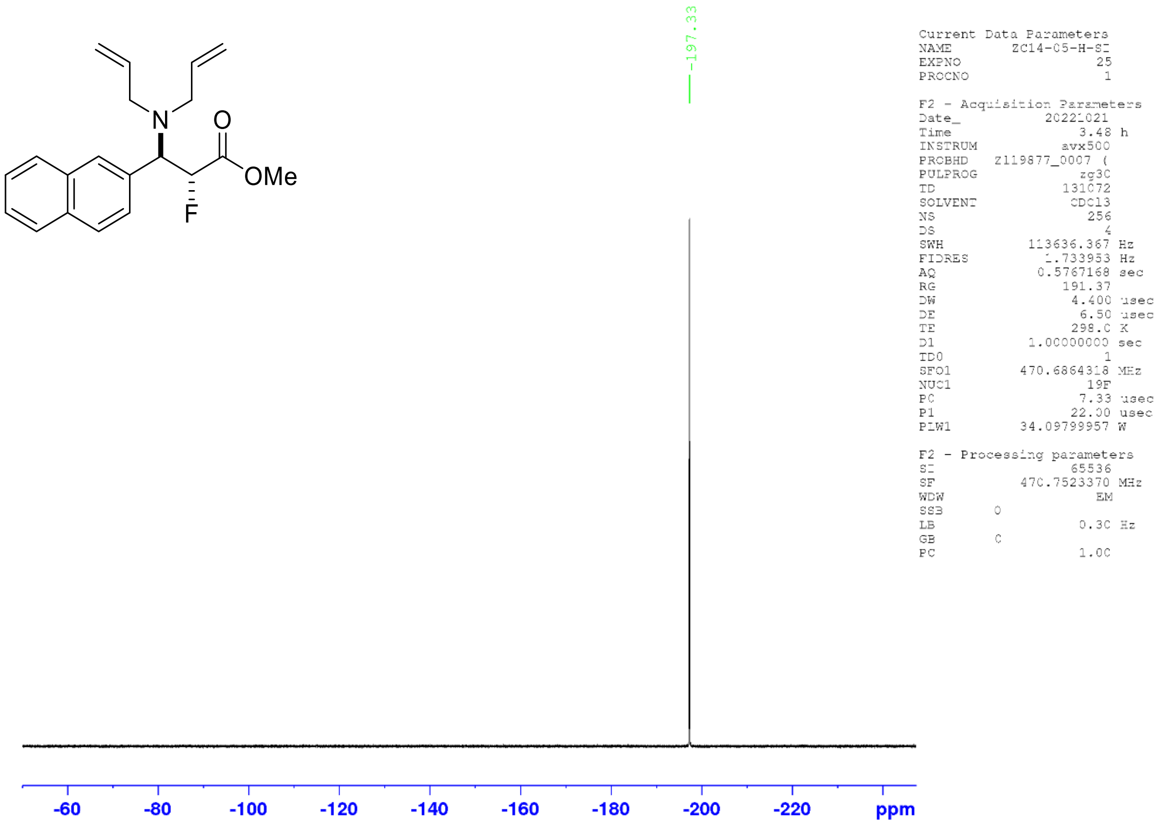
N
O
OMe
F
-197.33
Current Data Parameters
NAME ZC14-05-H-SI
EXPNO 25
PROCNO 1
F2 - Acquisition Parameters
Date_ 20221021
Time 3.48 h
INSTRUM avx500
PROBHD Z119877_0007 (
PULPROG zg30
TD 131072
SOLVENT CDCl3
NS 256
DS 4
SWH 113636.367 Hz
FIDRES 1.733953 Hz
AQ 0.5767168 sec
RG 191.37
DW 4.400 usec
DE 6.50 usec
TE 298.0 K
D1 1.00000000 sec
TD0 1
SFO1 470.6864318 MHz
NUC1 19F
P0 7.33 usec
P1 22.00 usec
PLW1 34.09799957 W
F2 - Processing parameters
SI 65536
SF 470.7523370 MHz
WDW EM
SSB 0
LB 0.30 Hz
GB 0
PC 1.00
-60 -80 -100 -120 -140 -160 -180 -200 -220 ppm

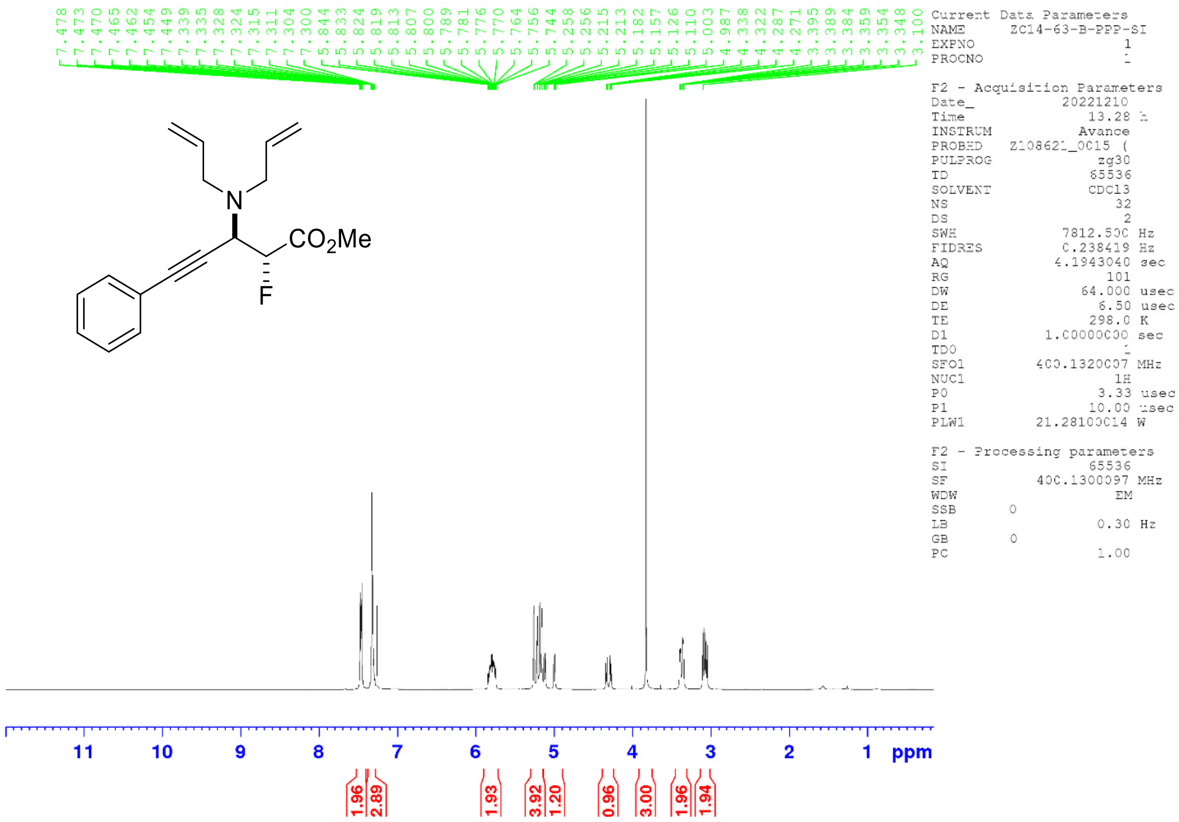
7.478 7.473 7.470 7.465 7.462 7.454 7.449 7.339 7.335 7.328 7.324 7.315 7.311 7.304 7.300 5.844 5.833 5.824 5.819 5.813 5.807 5.800 5.789 5.781 5.776 5.770 5.764 5.756 5.744 5.258 5.256 5.215 5.213 5.182 5.157 5.126 5.110 5.003 4.987 4.338 4.322 4.287 4.271 3.395 3.389 3.384 3.359 3.354 3.348 3.100
N
CO_2Me
F
Current Data Parameters
NAME ZC14-63-B-PPP-SI
EXPNO 1
PROCNO 1
F2 - Acquisition Parameters
Date_ 20221210
Time 13.28 h
INSTRUM Avance
PROBHD Z108621_0015 (
PULPROG zg30
TD 65536
SOLVENT CDCl3
NS 32
DS 2
SWH 7812.500 Hz
FIDRES 0.238419 Hz
AQ 4.1943040 sec
RG 101
DW 64.000 usec
DE 6.50 usec
TE 298.0 K
D1 1.00000000 sec
TD0 1
SFO1 400.1320007 MHz
NUC1 1H
P0 3.33 usec
P1 10.00 usec
PLW1 21.28100014 W
F2 - Processing parameters
SI 65536
SF 400.1300097 MHz
WDW EM
SSB 0
LB 0.30 Hz
GB 0
PC 1.00
11 10 9 8 7 6 5 4 3 2 1 ppm
1.96 2.89 1.93 3.92 1.20 0.96 3.00 1.96 1.94

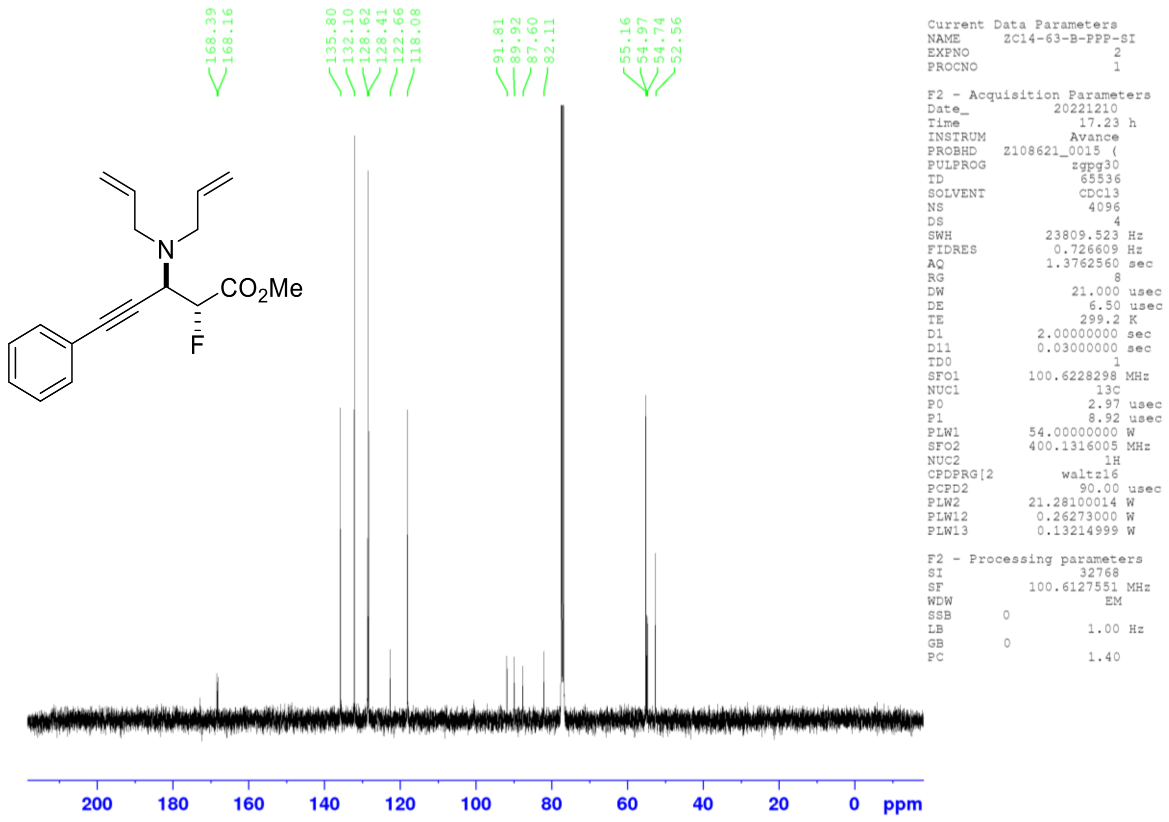
168.39
168.16
135.80
132.10
128.62
128.41
122.66
118.08
91.81
89.92
87.60
82.11
55.16
54.97
54.74
52.56
N
CO2Me
F
Current Data Parameters
NAME ZC14-63-B-PPP-SI
EXPNO 2
PROCNO 1
F2 - Acquisition Parameters
Date_ 20221210
Time 17.23 h
INSTRUM Avance
PROBHD Z108621_0015 (
PULPROG zgpg30
TD 65536
SOLVENT CDCl3
NS 4096
DS 4
SWH 23809.523 Hz
FIDRES 0.726609 Hz
AQ 1.3762560 sec
RG 8
DW 21.000 usec
DE 6.50 usec
TE 299.2 K
D1 2.00000000 sec
D11 0.03000000 sec
TD0 1
SFO1 100.6228298 MHz
NUC1 13C
P0 2.97 usec
P1 8.92 usec
PLW1 54.00000000 W
SFO2 400.1316005 MHz
NUC2 1H
CPDPRG[2 waltz16
PCPD2 90.00 usec
PLW2 21.28100014 W
PLW12 0.26273000 W
PLW13 0.13214999 W
F2 - Processing parameters
SI 32768
SF 100.6127551 MHz
WDW EM
SSB 0
LB 1.00 Hz
GB 0
PC 1.40
200 180 160 140 120 100 80 60 40 20 0 ppm

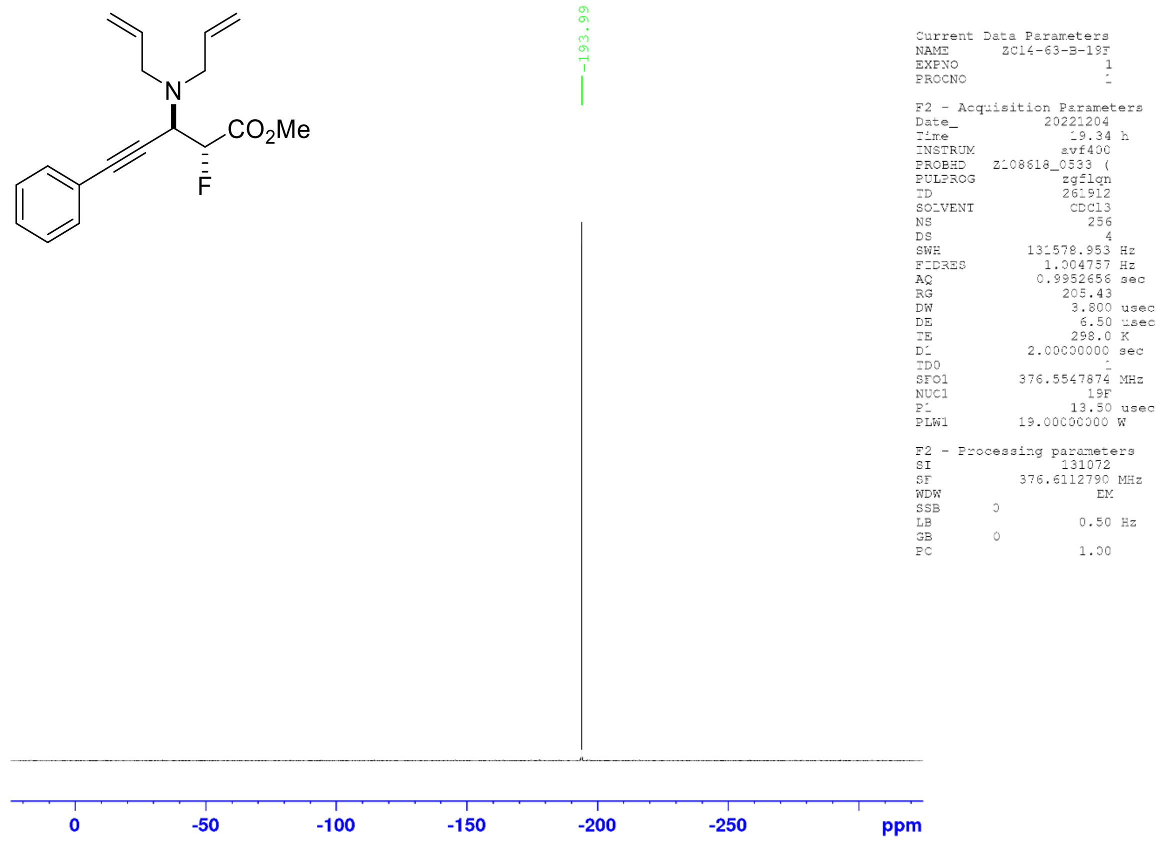
-193.99
N
CO2Me
F
Current Data Parameters
NAME ZC14-63-B-19F
EXPNO 1
PROCNO 1
F2 - Acquisition Parameters
Date_ 20221204
Time 19.34 h
INSTRUM avf400
PROBHD Z108618_0533 (
PULPROG zgflqn
TD 261912
SOLVENT CDCl3
NS 256
DS 4
SWH 131578.953 Hz
FIDRES 1.004757 Hz
AQ 0.9952656 sec
RG 205.43
DW 3.800 usec
DE 6.50 usec
TE 298.0 K
D1 2.00000000 sec
TD0 1
SFO1 376.5547874 MHz
NUC1 19F
P1 13.50 usec
PLW1 19.00000000 W
F2 - Processing parameters
SI 131072
SF 376.6112790 MHz
WDW EM
SSB 0
LB 0.50 Hz
GB 0
PC 1.00
0 -50 -100 -150 -200 -250 ppm

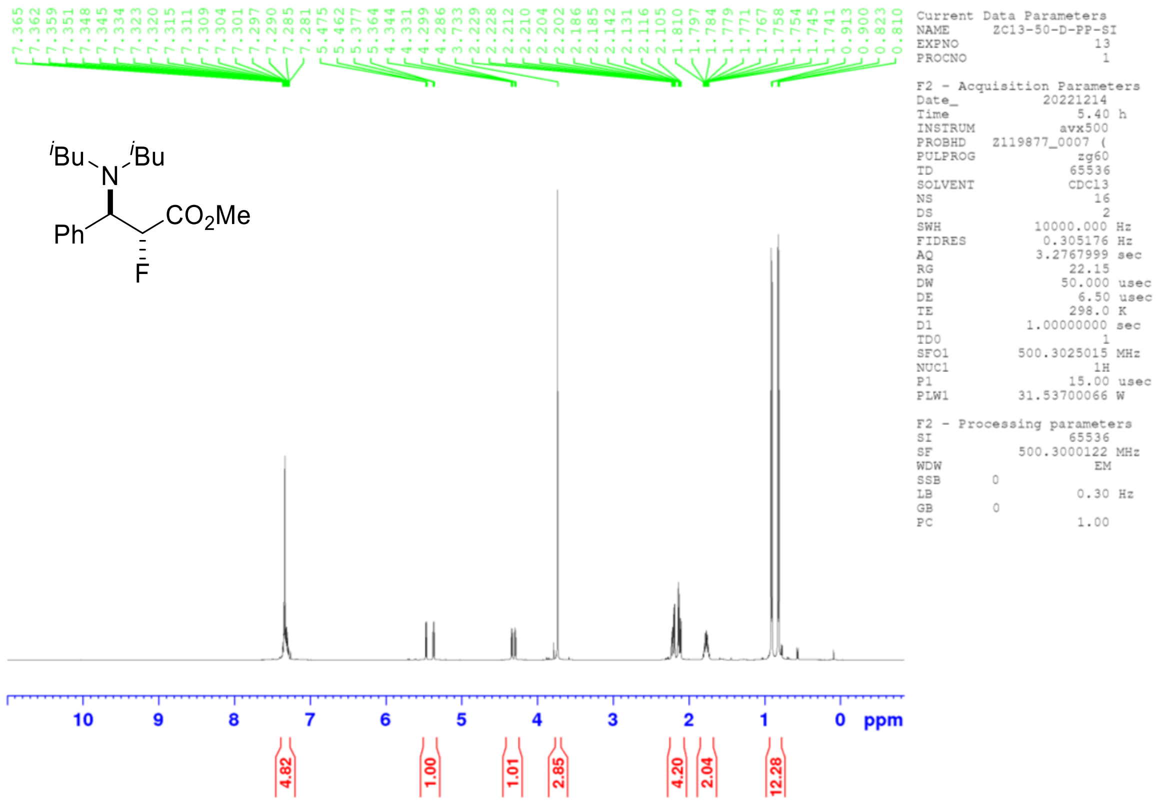
7.365 7.362 7.359 7.351 7.348 7.345 7.334 7.323 7.320 7.315 7.311 7.309 7.304 7.301 7.297 7.290 7.285 7.281 5.475 5.462 5.377 5.364 4.344 4.331 4.299 4.286 3.733 2.229 2.228 2.212 2.210 2.204 2.202 2.186 2.185 2.142 2.131 2.116 2.105 1.810 1.797 1.784 1.779 1.771 1.767 1.758 1.754 1.745 1.741 0.913 0.900 0.823 0.810
iBu
N
iBu
Ph
CO2Me
F
Current Data Parameters
NAME ZC13-50-D-PP-SI
EXPNO 13
PROCNO 1
F2 - Acquisition Parameters
Date_ 20221214
Time 5.40 h
INSTRUM avx500
PROBHD Z119877_0007 (
PULPROG zg60
TD 65536
SOLVENT CDCl3
NS 16
DS 2
SWH 10000.000 Hz
FIDRES 0.305176 Hz
AQ 3.2767999 sec
RG 22.15
DW 50.000 usec
DE 6.50 usec
TE 298.0 K
D1 1.00000000 sec
TD0 1
SFO1 500.3025015 MHz
NUC1 1H
P1 15.00 usec
PLW1 31.53700066 W
F2 - Processing parameters
SI 65536
SF 500.3000122 MHz
WDW EM
SSB 0
LB 0.30 Hz
GB 0
PC 1.00
10 9 8 7 6 5 4 3 2 1 0 ppm
4.82 1.00 1.01 2.85 4.20 2.04 12.28

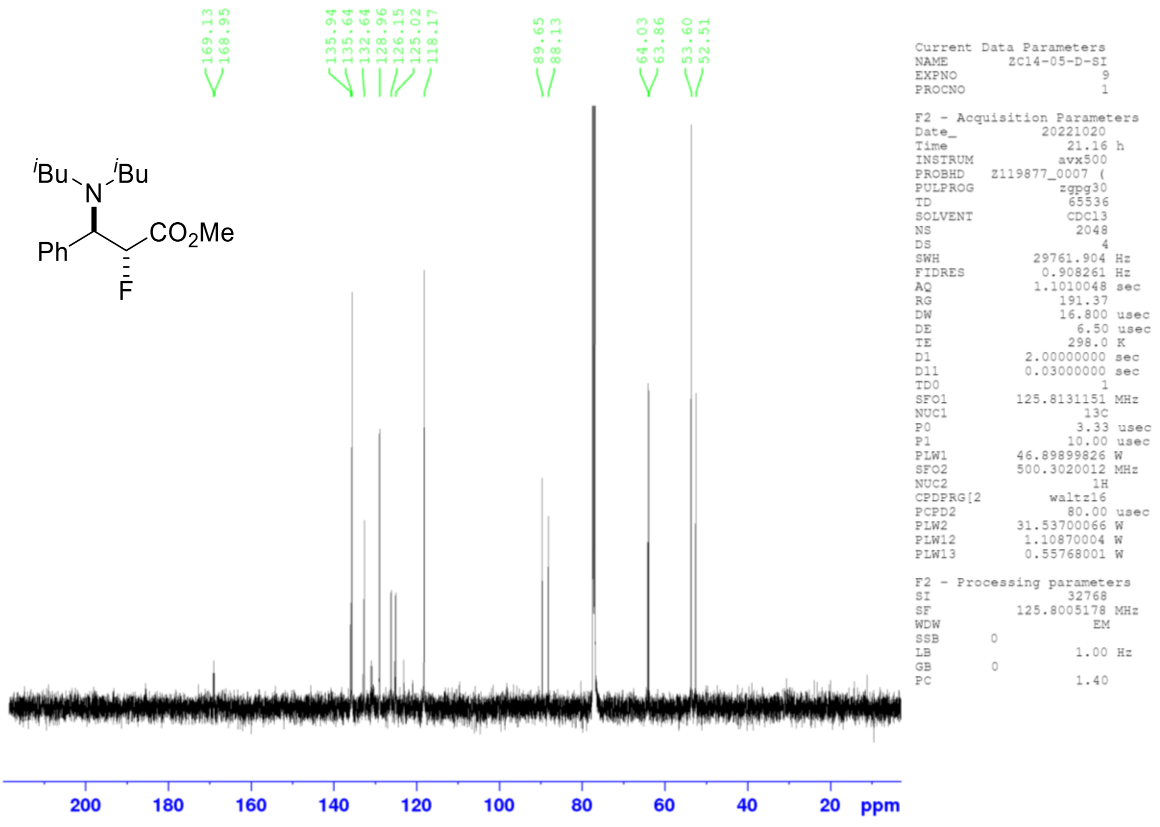
169.13 168.95 135.94 135.64 132.64 128.96 126.15 125.02 118.17 89.65 88.13 64.03 63.86 53.60 52.51
iBu
N
iBu
Ph
CO2Me
F
Current Data Parameters
NAME ZC14-05-D-SI
EXPNO 9
PROCNO 1
F2 - Acquisition Parameters
Date_ 20221020
Time 21.16 h
INSTRUM avx500
PROBHD Z119877_0007 (
PULPROG zgpg30
TD 65536
SOLVENT CDCl3
NS 2048
DS 4
SWH 29761.904 Hz
FIDRES 0.908261 Hz
AQ 1.1010048 sec
RG 191.37
DW 16.800 usec
DE 6.50 usec
TE 298.0 K
D1 2.00000000 sec
D11 0.03000000 sec
TD0 1
SFO1 125.8131151 MHz
NUC1 13C
P0 3.33 usec
P1 10.00 usec
PLW1 46.89899826 W
SFO2 500.3020012 MHz
NUC2 1H
CPDPRG[2 waltz16
PCPD2 80.00 usec
PLW2 31.53700066 W
PLW12 1.10870004 W
PLW13 0.55768001 W
F2 - Processing parameters
SI 32768
SF 125.8005178 MHz
WDW EM
SSB 0
LB 1.00 Hz
GB 0
PC 1.40
200 180 160 140 120 100 80 60 40 20 ppm

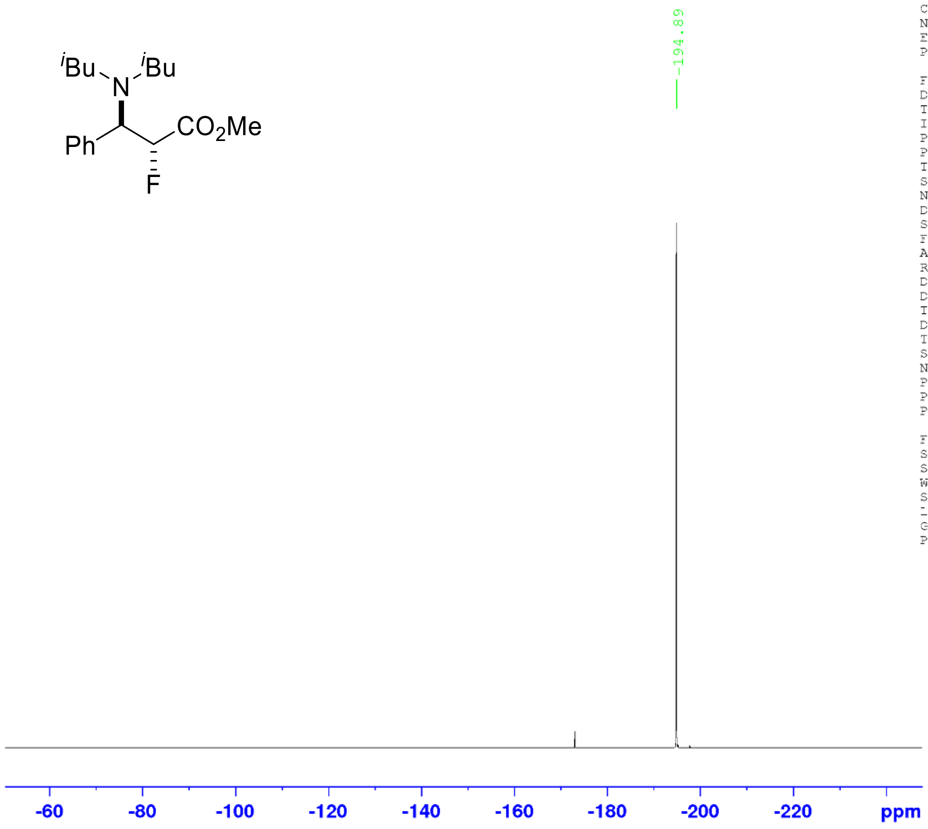

Current Data Parameters
NAME ZC13-50-D-PP-SI
EXPNO 14
PROCNO 1

F2 - Acquisition Parameters
Date_ 20221214
Time 5.47 h
INSTRUM avx500
PROBHD Z119877_0007 (
PULPROG zg30
TD 131072
SOLVENT CDCl3
NS 256
DS 4
SWH 113636.367 Hz
FIDRES 1.733953 Hz
AQ 0.5767168 sec
RG 191.37
DW 4.400 usec
DE 6.50 usec
TE 298.0 K
D1 1.00000000 sec
TD0 1
SFO1 470.6864318 MHz
NUC1 19F
P0 7.33 usec
P1 22.00 usec
PLW1 34.09799957 W

F2 - Processing parameters
SI 65536
SF 470.7523370 MHz
WDW EM
SSB 0
LB 0.30 Hz
GB 0
PC 1.00

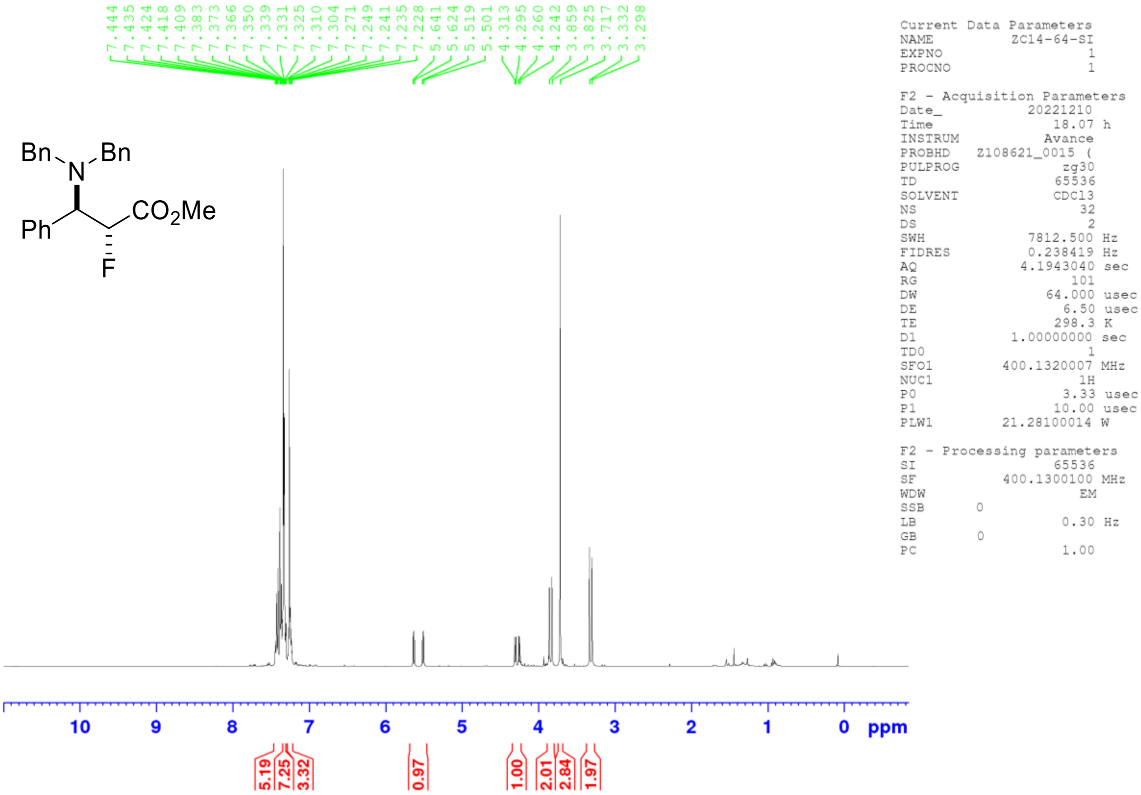

Current Data Parameters
NAME ZC14-64-SI
EXPNO 1
PROCNO 1

F2 - Acquisition Parameters
Date_ 20221210
Time 18.07 h
INSTRUM Avance
PROBHD Z108621_0015 (
PULPROG zg30
TD 65536
SOLVENT CDCl3
NS 32
DS 2
SWH 7812.500 Hz
FIDRES 0.238419 Hz
AQ 4.1943040 sec
RG 101
DW 64.000 usec
DE 6.50 usec
TE 298.3 K
D1 1.00000000 sec
TD0 1
SFO1 400.1320007 MHz
NUC1 1H
P0 3.33 usec
P1 10.00 usec
PLW1 21.28100014 W

F2 - Processing parameters
SI 65536
SF 400.1300100 MHz
WDW EM
SSB 0
LB 0.30 Hz
GB 0
PC 1.00

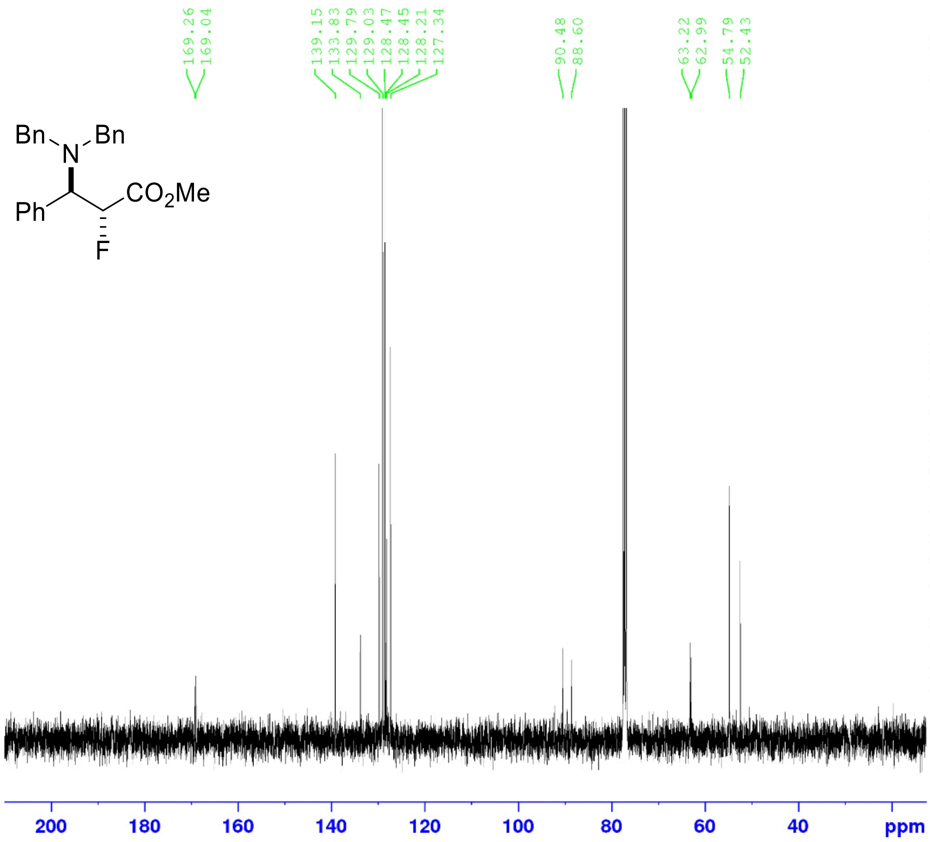


```
Current Data Parameters
NAME              ZC14-64-SI
EXPNO                      2
PROCNO                     1

F2 - Acquisition Parameters
Date_               20221210
Time                 19.50 h
INSTRUM               Avance
PROBHD    Z108621_0015 (
PULPROG              zgpg30
TD                    65536
SOLVENT               CDCl3
NS                     1800
DS                        4
SWH               23809.523 Hz
FIDRES             0.726609 Hz
AQ                1.3762560 sec
RG                        8
DW                   21.000 usec
DE                     6.50 usec
TE                    300.2 K
D1               2.00000000 sec
D11              0.03000000 sec
TD0                       1
SFO1            100.6228298 MHz
NUC1                    13C
P0                     2.97 usec
P1                     8.92 usec
PLW1            54.00000000 W
SFO2            400.1316005 MHz
NUC2                     1H
CPDPRG[2            waltz16
PCPD2                 90.00 usec
PLW2            21.28100014 W
PLW12            0.26273000 W
PLW13            0.13214999 W

F2 - Processing parameters
SI                    32768
SF              100.6127554 MHz
WDW                      EM
SSB      0
LB                     1.00 Hz
GB       0
PC                     1.40
```

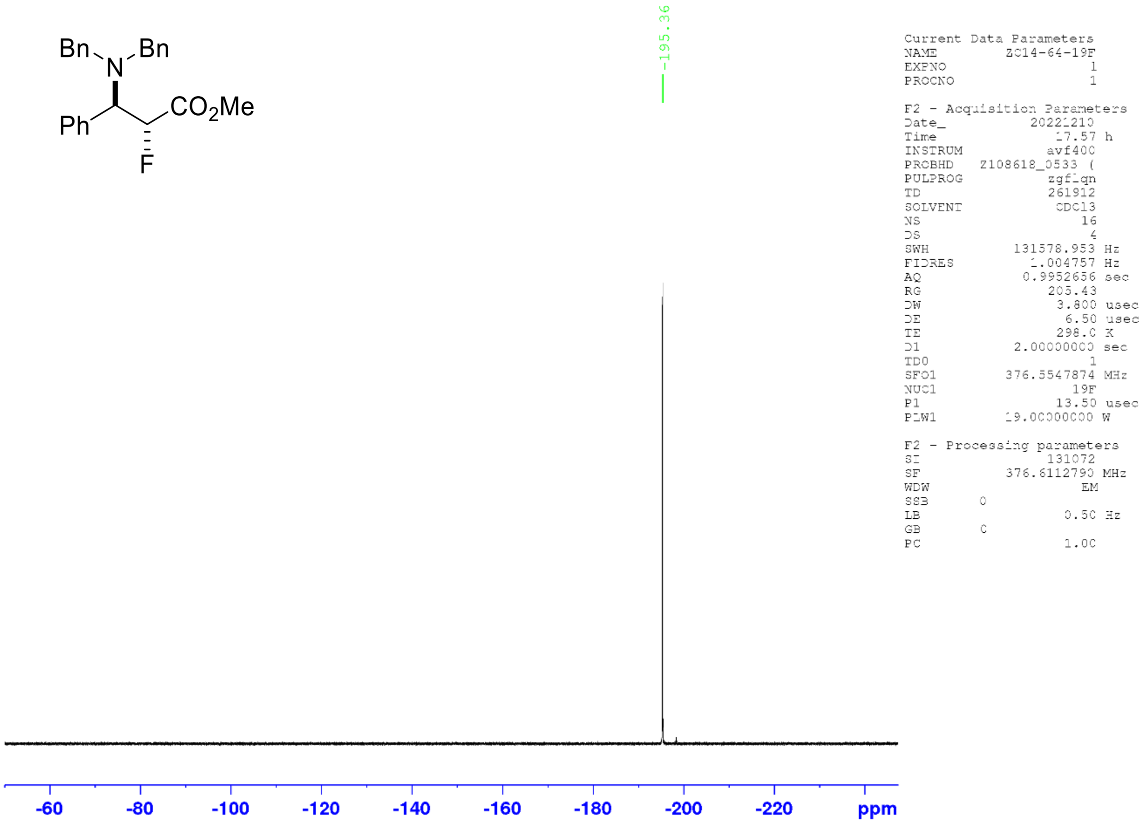


```
Current Data Parameters
NAME              ZC14-64-19F
EXPNO                      1
PROCNO                     1

F2 - Acquisition Parameters
Date_               20221210
Time                 17.57 h
INSTRUM              avf400
PROBHD    Z108618_0533 (
PULPROG              zgflqn
TD                   261912
SOLVENT               CDCl3
NS                       16
DS                        4
SWH              131578.953 Hz
FIDRES             1.004757 Hz
AQ                0.9952656 sec
RG                   205.43
DW                    3.800 usec
DE                     6.50 usec
TE                    298.0 K
D1               2.00000000 sec
TD0                       1
SFO1            376.5547874 MHz
NUC1                    19F
P1                    13.50 usec
PLW1            19.00000000 W

F2 - Processing parameters
SI                   131072
SF              376.6112790 MHz
WDW                      EM
SSB      0
LB                     0.50 Hz
GB       0
PC                     1.00
```

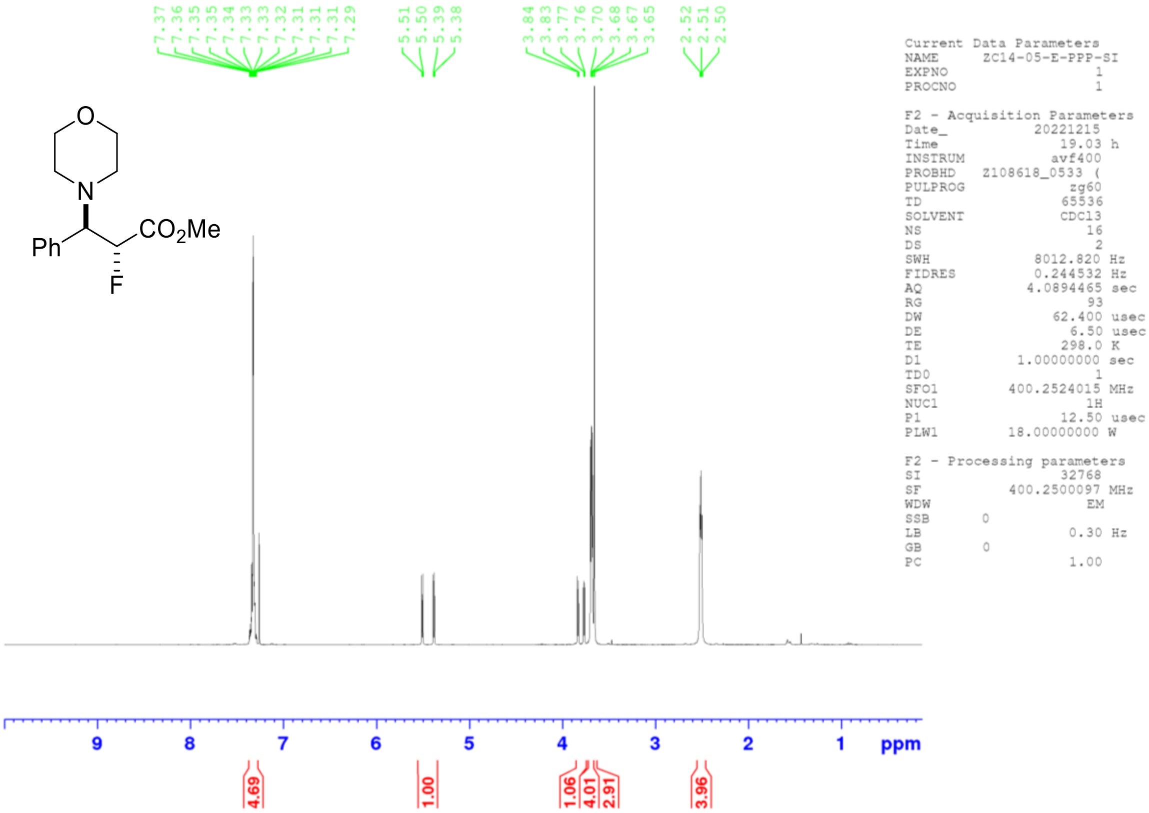


```
Current Data Parameters
NAME       ZC14-05-E-PPP-SI
EXPNO                     1
PROCNO                    1

F2 - Acquisition Parameters
Date_              20221215
Time                  19.03 h
INSTRUM              avf400
PROBHD    Z108618_0533 (
PULPROG                zg60
TD                    65536
SOLVENT               CDCl3
NS                       16
DS                        2
SWH                8012.820 Hz
FIDRES             0.244532 Hz
AQ                4.0894465 sec
RG                       93
DW                   62.400 usec
DE                     6.50 usec
TE                    298.0 K
D1               1.00000000 sec
TD0                       1
SFO1            400.2524015 MHz
NUC1                     1H
P1                    12.50 usec
PLW1            18.00000000 W

F2 - Processing parameters
SI                    32768
SF              400.2500097 MHz
WDW                      EM
SSB      0
LB                     0.30 Hz
GB       0
PC                     1.00
```

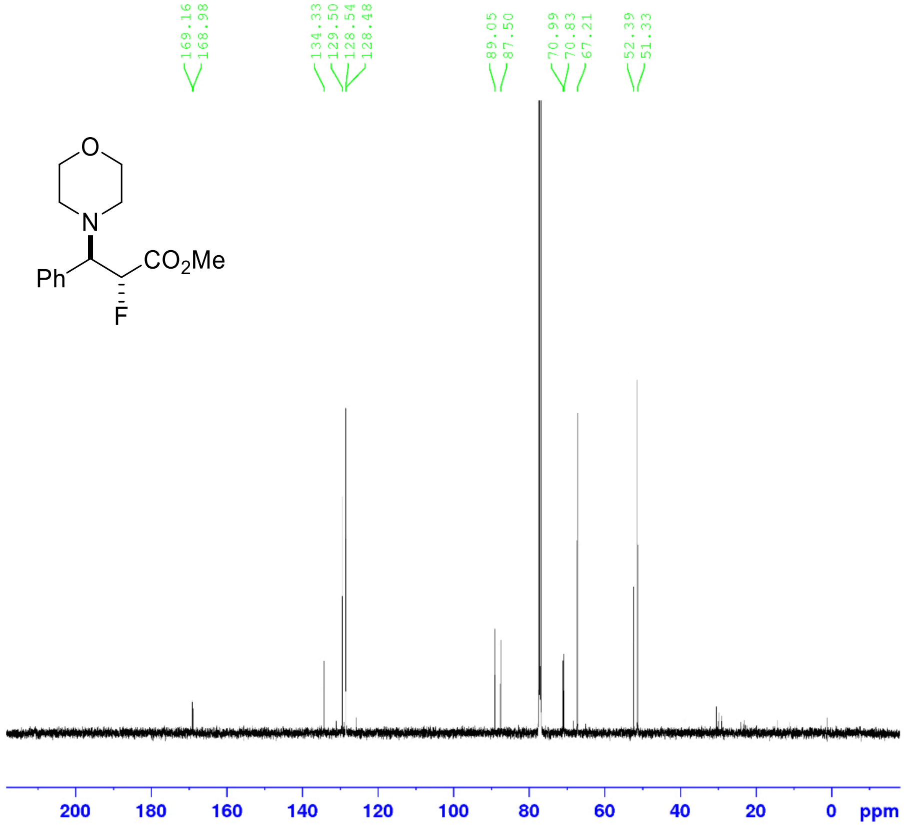


```
Current Data Parameters
NAME         ZC14-05-E-SI
EXPNO                  12
PROCNO                  1

F2 - Acquisition Parameters
Date_            20221020
Time                23.22 h
INSTRUM            avx500
PROBHD    Z119877_0007 (
PULPROG           zgpg30
TD                 65536
SOLVENT            CDCl3
NS                  2048
DS                     4
SWH            29761.904 Hz
FIDRES          0.908261 Hz
AQ             1.1010048 sec
RG                191.37
DW                16.800 usec
DE                  6.50 usec
TE                 298.0 K
D1            2.00000000 sec
D11           0.03000000 sec
TD0                    1
SFO1         125.8131151 MHz
NUC1                 13C
P0                  3.33 usec
P1                 10.00 usec
PLW1         46.89899826 W
SFO2         500.3020012 MHz
NUC2                  1H
CPDPRG[2         waltz16
PCPD2              80.00 usec
PLW2         31.53700066 W
PLW12         1.10870004 W
PLW13         0.55768001 W

F2 - Processing parameters
SI                 32768
SF           125.8005185 MHz
WDW                   EM
SSB      0
LB                  1.00 Hz
GB       0
PC                  1.40
```

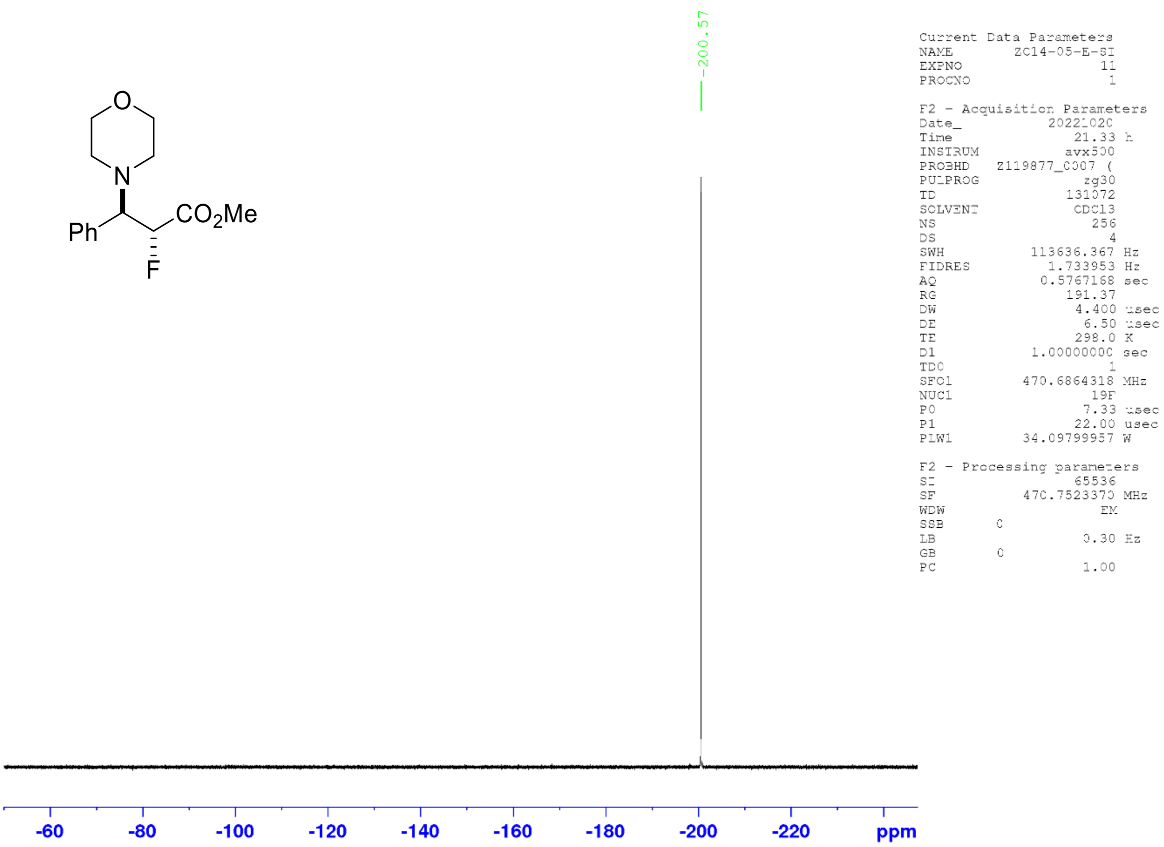
O
N
Ph
CO2Me
F
-200.57
-60
-80
-100
-120
-140
-160
-180
-200
-220
ppm
Current Data Parameters
NAME ZC14-05-E-SI
EXPNO 11
PROCNO 1
F2 - Acquisition Parameters
Date_ 20221020
Time 21.33 h
INSTRUM avx500
PROBHD Z119877_0007 (
PULPROG zg30
TD 131072
SOLVENT CDCl3
NS 256
DS 4
SWH 113636.367 Hz
FIDRES 1.733953 Hz
AQ 0.5767168 sec
RG 191.37
DW 4.400 usec
DE 6.50 usec
TE 298.0 K
D1 1.00000000 sec
TD0 1
SFO1 470.6864318 MHz
NUC1 19F
P0 7.33 usec
P1 22.00 usec
PLW1 34.09799957 W
F2 - Processing parameters
SI 65536
SF 470.7523370 MHz
WDW EM
SSB 0
LB 0.30 Hz
GB 0
PC 1.00

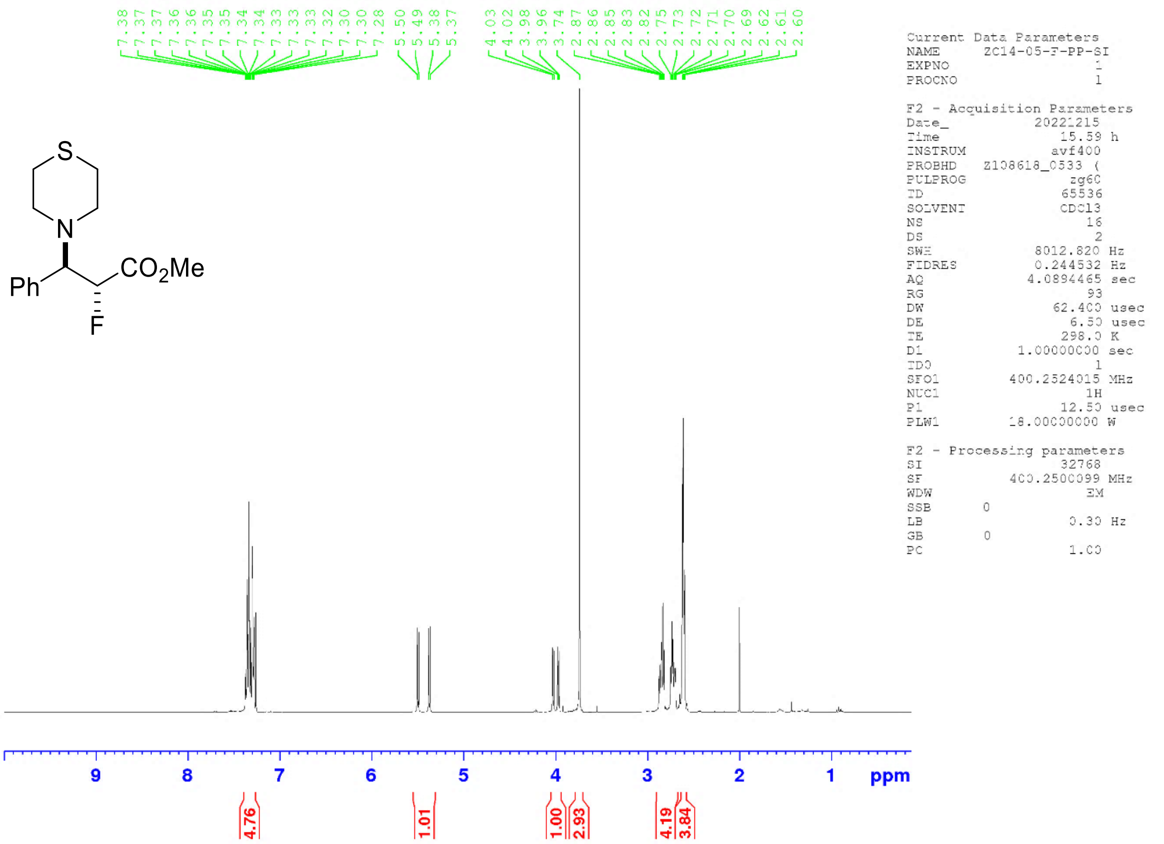
S
N
Ph
CO2Me
F
7.38 7.37 7.37 7.36 7.35 7.35 7.34 7.34 7.33 7.33 7.33 7.32 7.30 7.30 7.28
5.50 5.49 5.38 5.37
4.03 4.02 3.98 3.96 3.74 2.87 2.86 2.85 2.83 2.82 2.75 2.73 2.72 2.71 2.70 2.69 2.62 2.61 2.60
9
8
7
6
5
4
3
2
1
ppm
4.76
1.01
1.00
2.93
4.19
3.84
Current Data Parameters
NAME ZC14-05-F-PP-SI
EXPNO 1
PROCNO 1
F2 - Acquisition Parameters
Date_ 20221215
Time 15.59 h
INSTRUM avf400
PROBHD Z108618_0533 (
PULPROG zg60
TD 65536
SOLVENT CDCl3
NS 16
DS 2
SWH 8012.820 Hz
FIDRES 0.244532 Hz
AQ 4.0894465 sec
RG 93
DW 62.400 usec
DE 6.50 usec
TE 298.0 K
D1 1.00000000 sec
TD0 1
SFO1 400.2524015 MHz
NUC1 1H
P1 12.50 usec
PLW1 18.00000000 W
F2 - Processing parameters
SI 32768
SF 400.2500099 MHz
WDW EM
SSB 0
LB 0.30 Hz
GB 0
PC 1.00

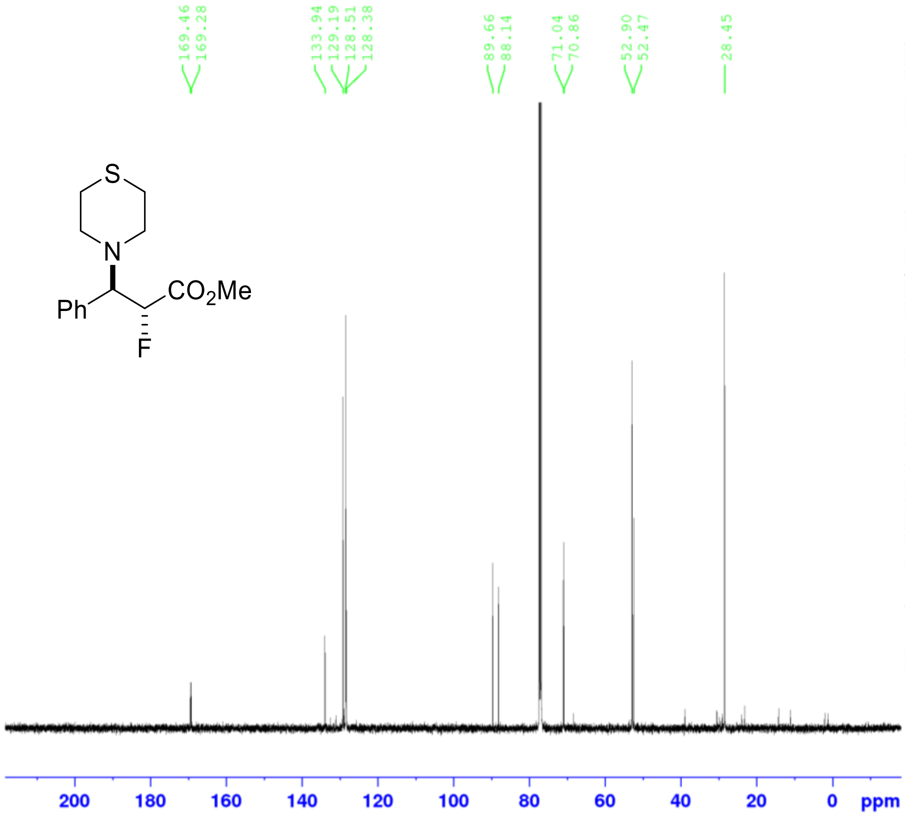


```
Current Data Parameters
NAME          ZC14-05-F-SI
EXPNO                   15
PROCNO                   1

F2 - Acquisition Parameters
Date_             20221021
Time                  1.27 h
INSTRUM             avx500
PROBHD     Z119877_0007 (
PULPROG             zgpg30
TD                   65536
SOLVENT              CDCl3
NS                    2048
DS                       4
SWH              29761.904 Hz
FIDRES            0.908261 Hz
AQ               1.1010048 sec
RG                  191.37
DW                  16.800 usec
DE                    6.50 usec
TE                   298.0 K
D1              2.00000000 sec
D11             0.03000000 sec
TD0                      1
SFO1           125.8131151 MHz
NUC1                   13C
P0                    3.33 usec
P1                   10.00 usec
PLW1           46.89899826 W
SFO2           500.3020012 MHz
NUC2                    1H
CPDPRG[2           waltz16
PCPD2                80.00 usec
PLW2           31.53700066 W
PLW12           1.10870004 W
PLW13           0.55768001 W

F2 - Processing parameters
SI                   32768
SF             125.8005196 MHz
WDW                     EM
SSB      0
LB                    1.00 Hz
GB       0
PC                    1.40
```

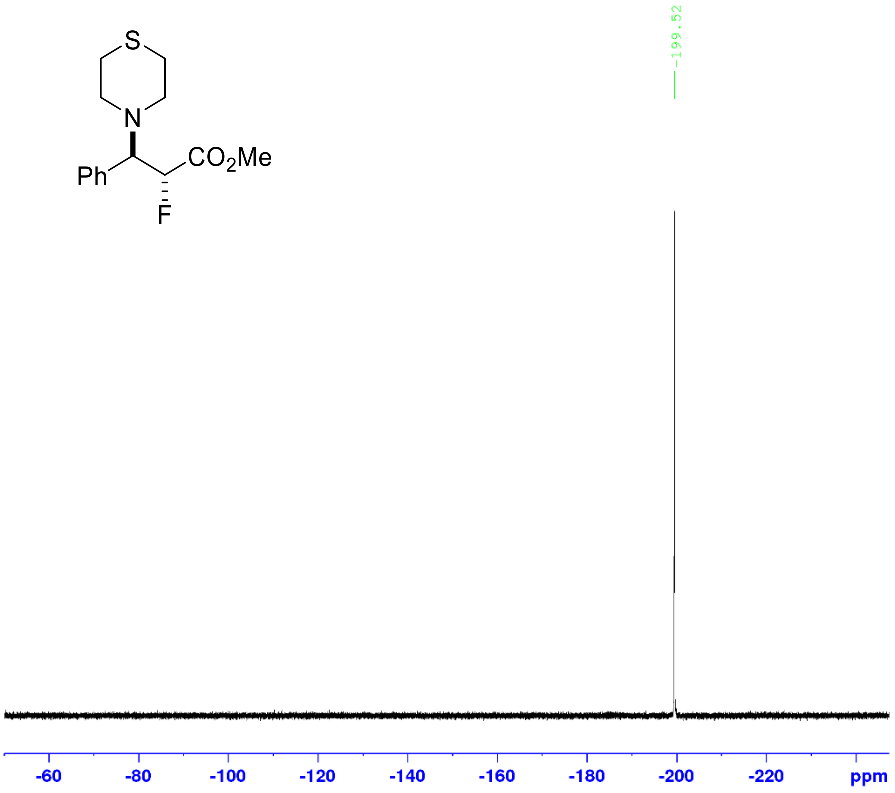


```
Current Data Parameters
NAME          ZC14-05-G-SI
EXPNO                   17
PROCNO                   1

F2 - Acquisition Parameters
Date_             20221021
Time                  1.43 h
INSTRUM             avx500
PROBHD     Z119877_0007 (
PULPROG               zg30
TD                  131072
SOLVENT              CDCl3
NS                     256
DS                       4
SWH             113636.367 Hz
FIDRES            1.733953 Hz
AQ               0.5767168 sec
RG                  191.37
DW                   4.400 usec
DE                    6.50 usec
TE                   298.0 K
D1              1.00000000 sec
TD0                      1
SFO1           470.6864318 MHz
NUC1                   19F
P0                    7.33 usec
P1                   22.00 usec
PLW1           34.09799957 W

F2 - Processing parameters
SI                   65536
SF             470.7523370 MHz
WDW                     EM
SSB      0
LB                    0.30 Hz
GB       0
PC                    1.00
```

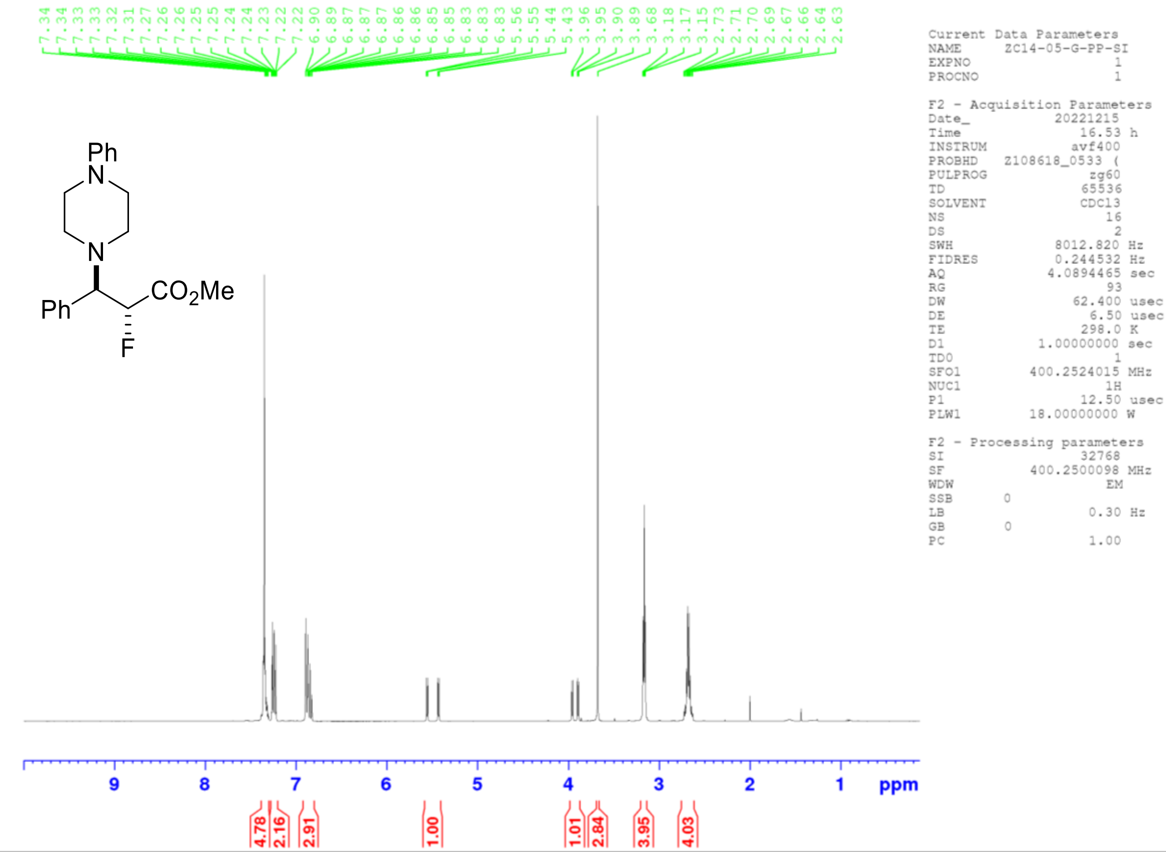
7.34 7.34 7.33 7.33 7.32 7.31 7.27 7.26 7.25 7.25 7.24 7.24 7.23 7.22 7.22 6.90 6.89 6.87 6.87 6.86 6.86 6.85 6.85 6.83 6.83 5.56 5.55 5.44 5.43 3.96 3.95 3.90 3.89 3.68 3.18 3.17 3.15 2.73 2.71 2.70 2.69 2.67 2.66 2.64 2.63
Ph
N
N
Ph
CO2Me
F
Current Data Parameters
NAME ZC14-05-G-PP-SI
EXPNO 1
PROCNO 1
F2 - Acquisition Parameters
Date_ 20221215
Time 16.53 h
INSTRUM avf400
PROBHD Z108618_0533 (
PULPROG zg60
TD 65536
SOLVENT CDCl3
NS 16
DS 2
SWH 8012.820 Hz
FIDRES 0.244532 Hz
AQ 4.0894465 sec
RG 93
DW 62.400 usec
DE 6.50 usec
TE 298.0 K
D1 1.00000000 sec
TD0 1
SFO1 400.2524015 MHz
NUC1 1H
P1 12.50 usec
PLW1 18.00000000 W
F2 - Processing parameters
SI 32768
SF 400.2500098 MHz
WDW EM
SSB 0
LB 0.30 Hz
GB 0
PC 1.00
9 8 7 6 5 4 3 2 1 ppm
4.78 2.16 2.91 1.00 1.01 2.84 3.95 4.03

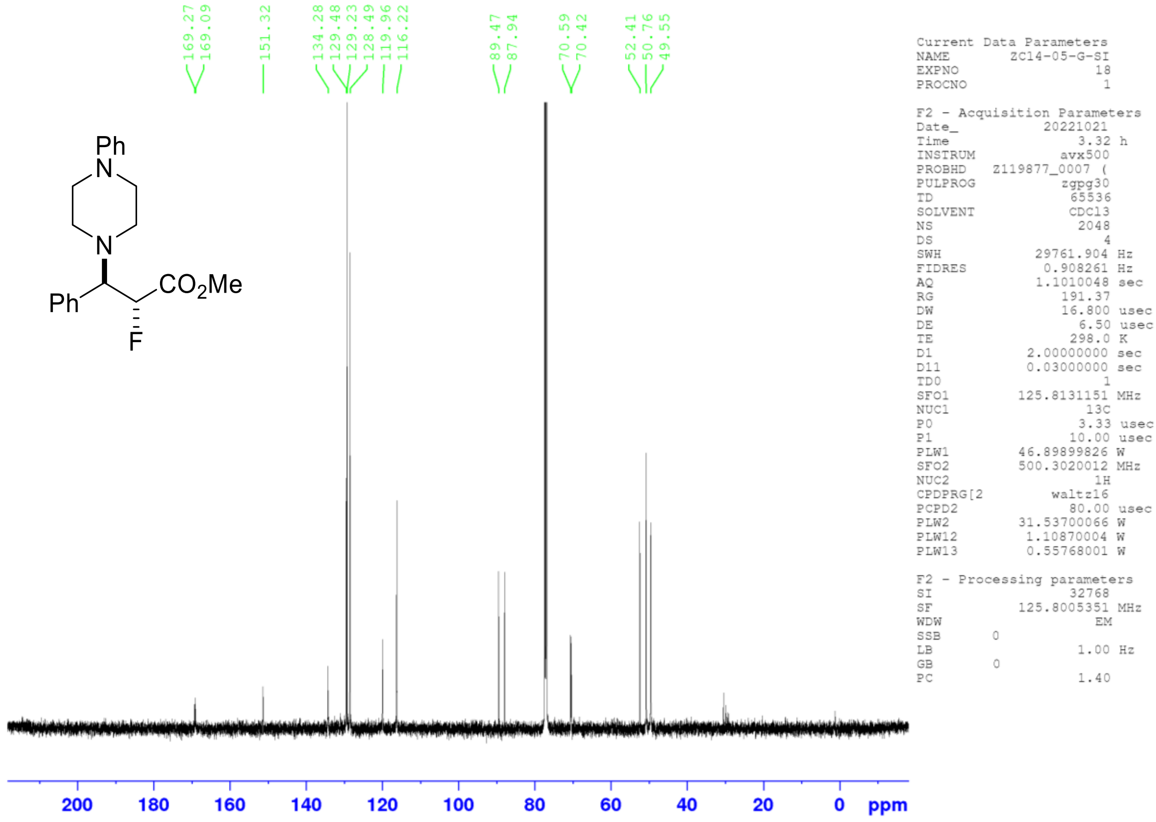
169.27 169.09 151.32 134.28 129.48 129.23 128.49 119.96 116.22 89.47 87.94 70.59 70.42 52.41 50.76 49.55
Ph
N
N
Ph
CO2Me
F
Current Data Parameters
NAME ZC14-05-G-SI
EXPNO 18
PROCNO 1
F2 - Acquisition Parameters
Date_ 20221021
Time 3.32 h
INSTRUM avx500
PROBHD Z119877_0007 (
PULPROG zgpg30
TD 65536
SOLVENT CDCl3
NS 2048
DS 4
SWH 29761.904 Hz
FIDRES 0.908261 Hz
AQ 1.1010048 sec
RG 191.37
DW 16.800 usec
DE 6.50 usec
TE 298.0 K
D1 2.00000000 sec
D11 0.03000000 sec
TD0 1
SFO1 125.8131151 MHz
NUC1 13C
P0 3.33 usec
P1 10.00 usec
PLW1 46.89899826 W
SFO2 500.3020012 MHz
NUC2 1H
CPDPRG[2 waltz16
PCPD2 80.00 usec
PLW2 31.53700066 W
PLW12 1.10870004 W
PLW13 0.55768001 W
F2 - Processing parameters
SI 32768
SF 125.8005351 MHz
WDW EM
SSB 0
LB 1.00 Hz
GB 0
PC 1.40
200 180 160 140 120 100 80 60 40 20 0 ppm

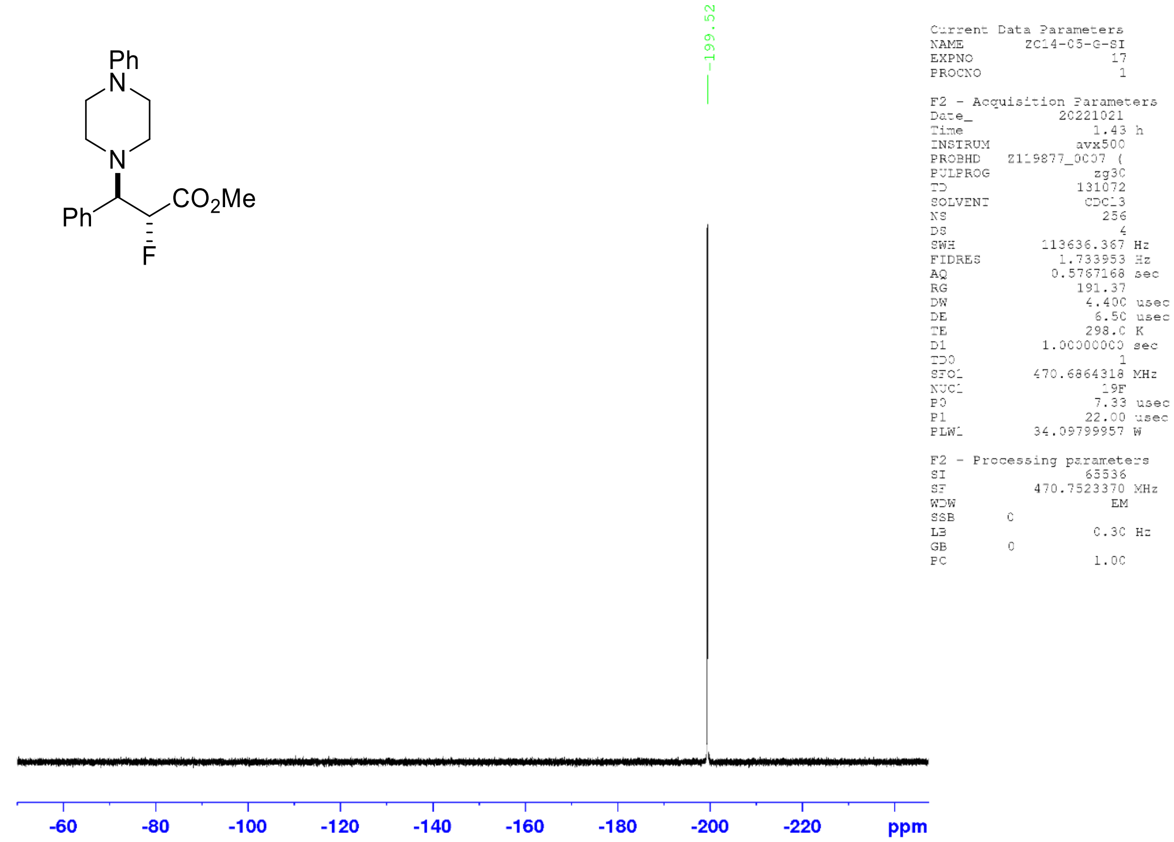
Ph
N
N
Ph
CO2Me
F
-199.52
-60 -80 -100 -120 -140 -160 -180 -200 -220 ppm
Current Data Parameters
NAME ZC14-05-G-SI
EXPNO 17
PROCNO 1
F2 - Acquisition Parameters
Date_ 20221021
Time 1.43 h
INSTRUM avx500
PROBHD Z119877_0007 (
PULPROG zg30
TD 131072
SOLVENT CDCl3
NS 256
DS 4
SWH 113636.367 Hz
FIDRES 1.733953 Hz
AQ 0.5767168 sec
RG 191.37
DW 4.400 usec
DE 6.50 usec
TE 298.0 K
D1 1.00000000 sec
TD0 1
SFO1 470.6864318 MHz
NUC1 19F
P0 7.33 usec
P1 22.00 usec
PLW1 34.09799957 W
F2 - Processing parameters
SI 65536
SF 470.7523370 MHz
WDW EM
SSB 0
LB 0.30 Hz
GB 0
PC 1.00

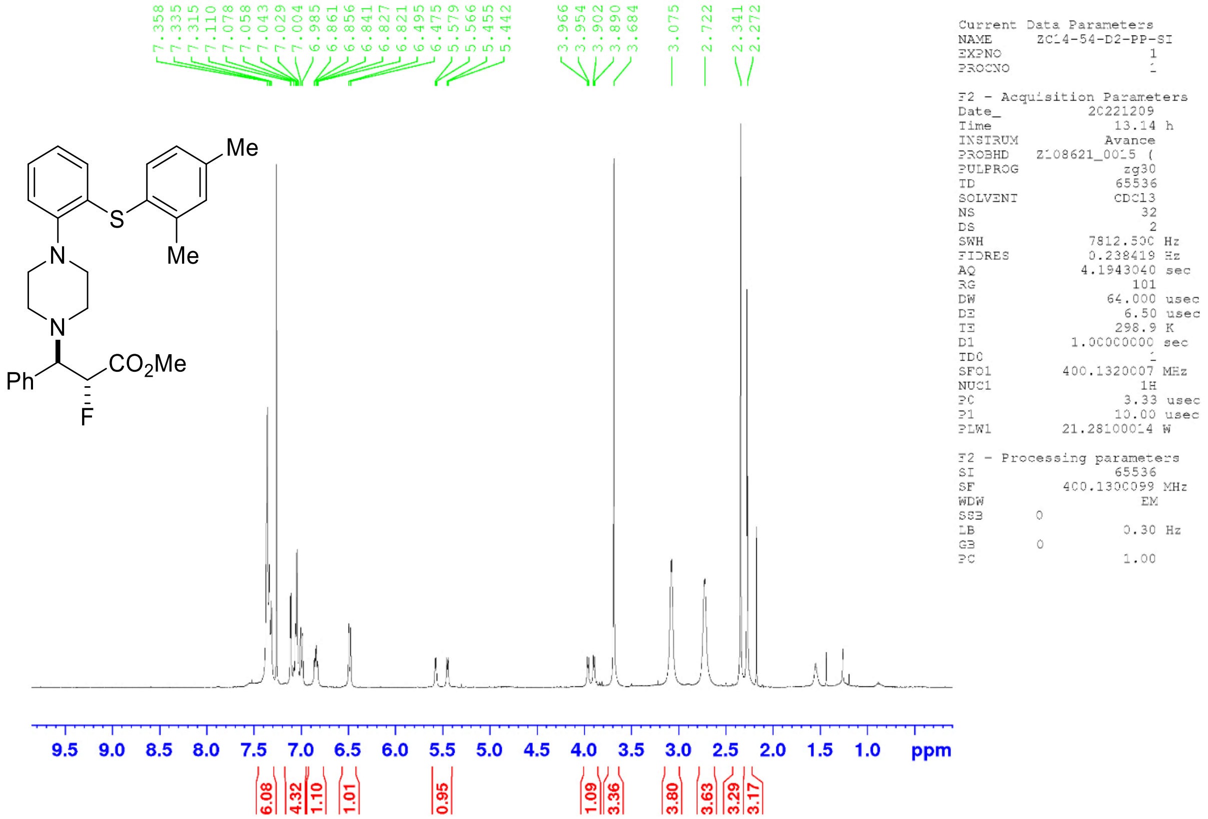
7.358 7.335 7.315 7.110 7.078 7.058 7.043 7.029 7.004 6.985 6.861 6.856 6.841 6.827 6.821 6.495 6.475 5.579 5.566 5.455 5.442
3.966 3.954 3.902 3.890 3.684 3.075 2.722 2.341 2.272
Me
S
Me
N
N
Ph
CO2Me
F
9.5 9.0 8.5 8.0 7.5 7.0 6.5 6.0 5.5 5.0 4.5 4.0 3.5 3.0 2.5 2.0 1.5 1.0 ppm
6.08 4.32 1.10 1.01 0.95 1.09 3.36 3.80 3.63 3.29 3.17
Current Data Parameters
NAME ZC14-54-D2-PP-SI
EXPNO 1
PROCNO 1
F2 - Acquisition Parameters
Date_ 20221209
Time 13.14 h
INSTRUM Avance
PROBHD Z108621_0015 (
PULPROG zg30
TD 65536
SOLVENT CDCl3
NS 32
DS 2
SWH 7812.500 Hz
FIDRES 0.238419 Hz
AQ 4.1943040 sec
RG 101
DW 64.000 usec
DE 6.50 usec
TE 298.9 K
D1 1.00000000 sec
TD0 1
SFO1 400.1320007 MHz
NUC1 1H
P0 3.33 usec
P1 10.00 usec
PLW1 21.28100014 W
F2 - Processing parameters
SI 65536
SF 400.1300099 MHz
WDW EM
SSB 0
LB 0.30 Hz
GB 0
PC 1.00

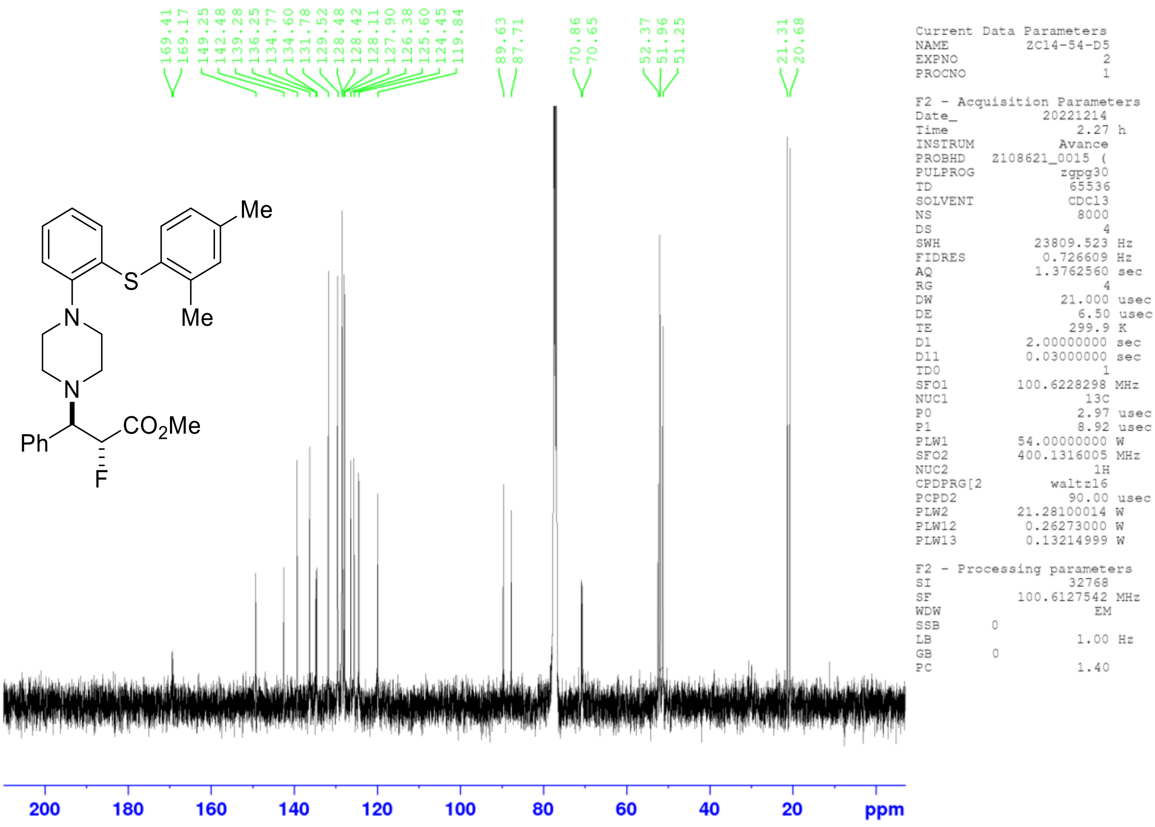
169.41
169.17
149.25
142.48
139.28
136.25
134.77
134.60
131.78
129.52
128.48
128.42
128.11
127.90
126.38
125.60
124.45
119.84
89.63
87.71
70.86
70.65
52.37
51.96
51.25
21.31
20.68
Me
S
Me
N
N
Ph
CO2Me
F
200
180
160
140
120
100
80
60
40
20
ppm
Current Data Parameters
NAME ZC14-54-D5
EXPNO 2
PROCNO 1
F2 - Acquisition Parameters
Date_ 20221214
Time 2.27 h
INSTRUM Avance
PROBHD Z108621_0015 (
PULPROG zgpg30
TD 65536
SOLVENT CDCl3
NS 8000
DS 4
SWH 23809.523 Hz
FIDRES 0.726609 Hz
AQ 1.3762560 sec
RG 4
DW 21.000 usec
DE 6.50 usec
TE 299.9 K
D1 2.00000000 sec
D11 0.03000000 sec
TD0 1
SFO1 100.6228298 MHz
NUC1 13C
P0 2.97 usec
P1 8.92 usec
PLW1 54.00000000 W
SFO2 400.1316005 MHz
NUC2 1H
CPDPRG[2 waltz16
PCPD2 90.00 usec
PLW2 21.28100014 W
PLW12 0.26273000 W
PLW13 0.13214999 W
F2 - Processing parameters
SI 32768
SF 100.6127542 MHz
WDW EM
SSB 0
LB 1.00 Hz
GB 0
PC 1.40

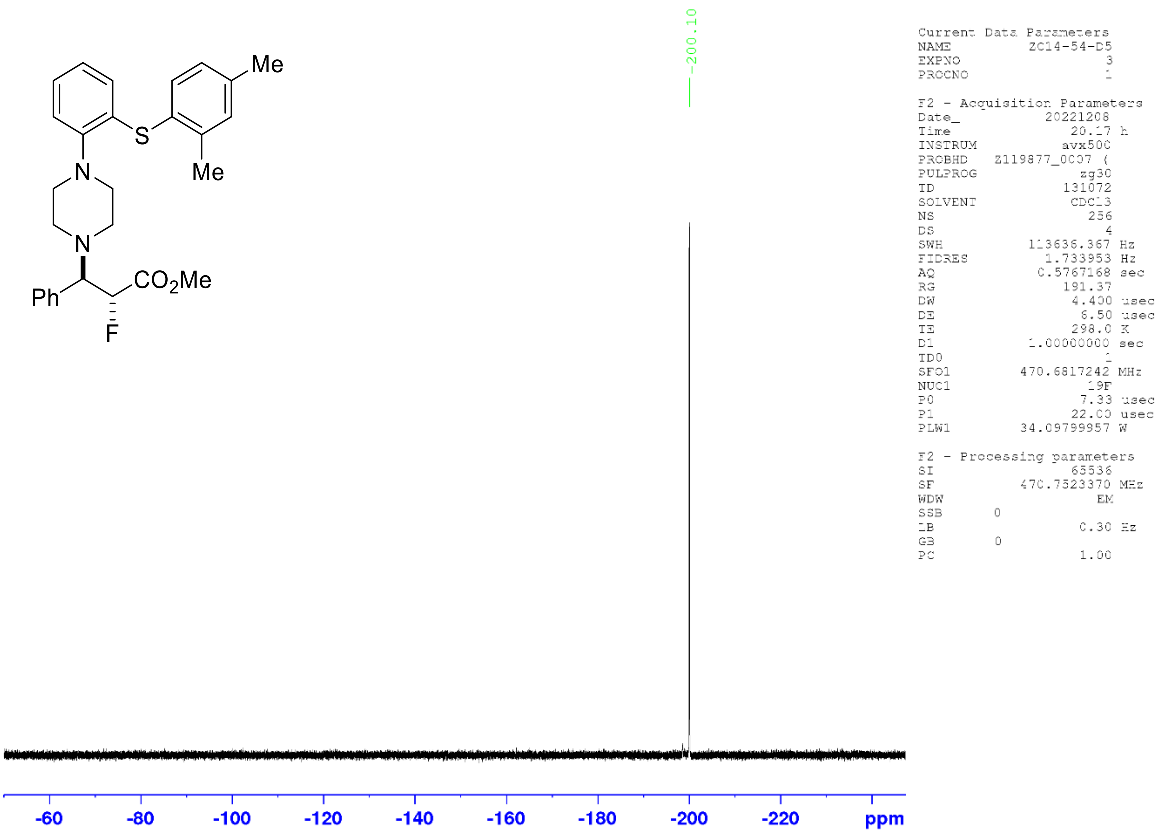
-200.10
Me
S
Me
N
N
Ph
CO2Me
F
-60
-80
-100
-120
-140
-160
-180
-200
-220
ppm
Current Data Parameters
NAME ZC14-54-D5
EXPNO 3
PROCNO 1
F2 - Acquisition Parameters
Date_ 20221208
Time 20.17 h
INSTRUM avx500
PROBHD Z119877_0007 (
PULPROG zg30
TD 131072
SOLVENT CDCl3
NS 256
DS 4
SWH 113636.367 Hz
FIDRES 1.733953 Hz
AQ 0.5767168 sec
RG 191.37
DW 4.400 usec
DE 6.50 usec
TE 298.0 K
D1 1.00000000 sec
TD0 1
SFO1 470.6817242 MHz
NUC1 19F
P0 7.33 usec
P1 22.00 usec
PLW1 34.09799957 W
F2 - Processing parameters
SI 65536
SF 470.7523370 MHz
WDW EM
SSB 0
LB 0.30 Hz
GB 0
PC 1.00

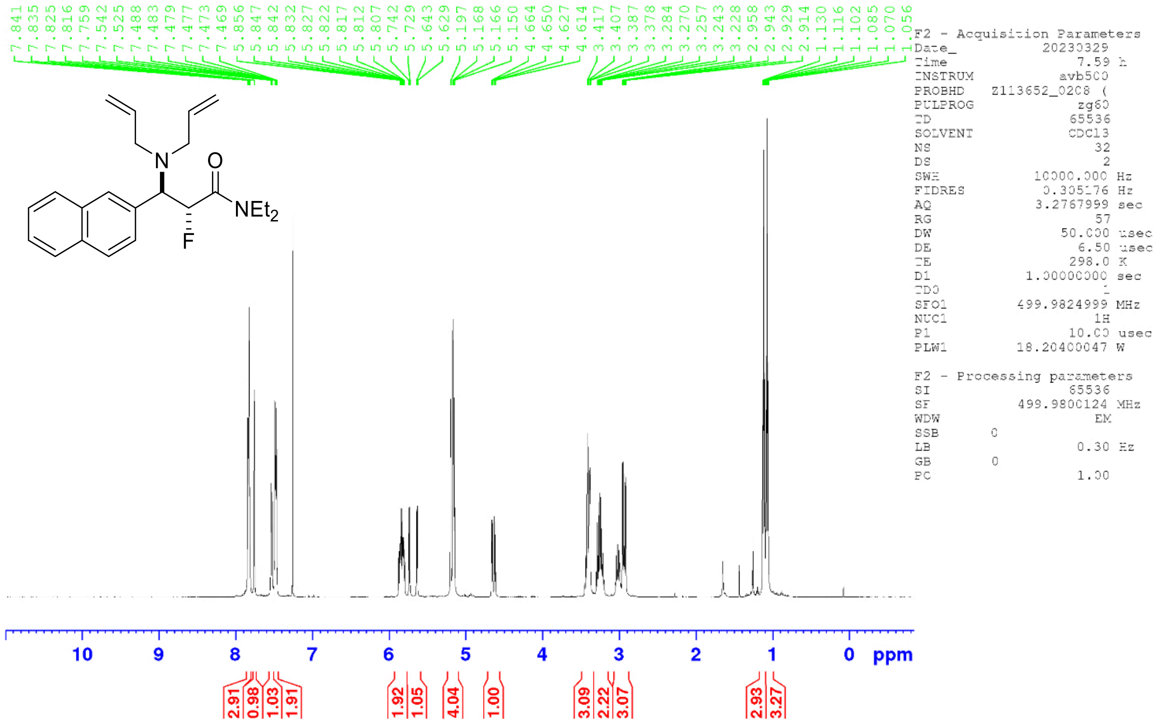

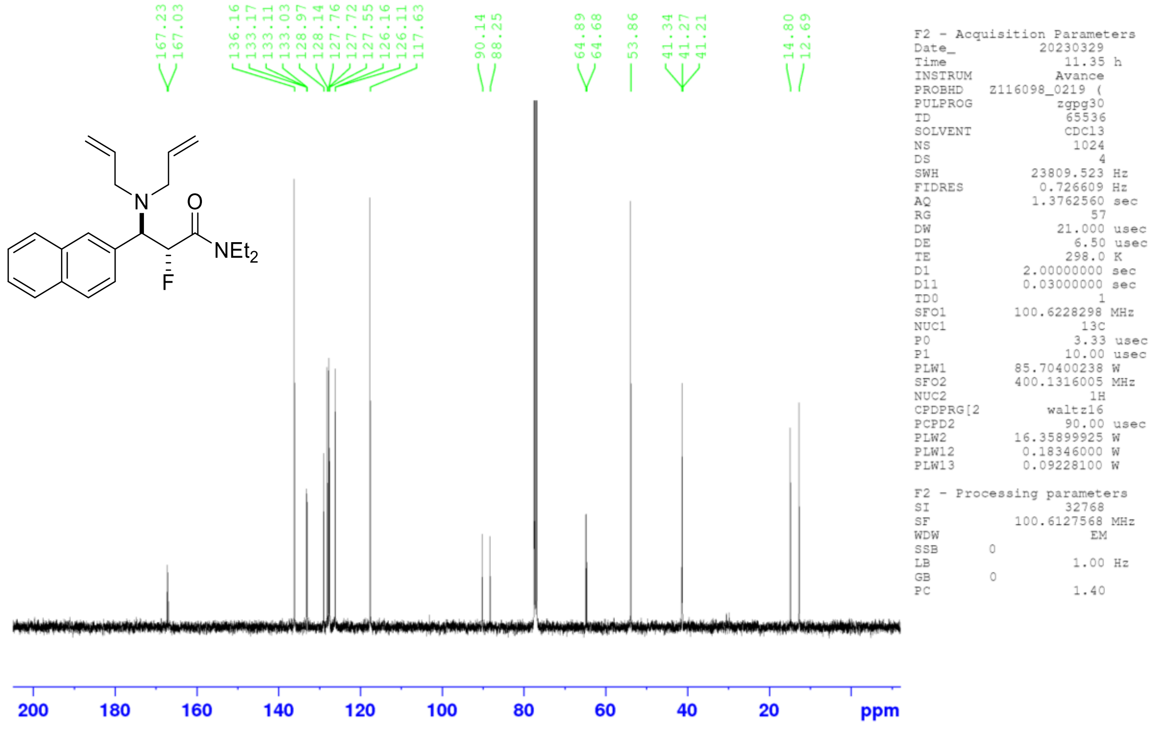

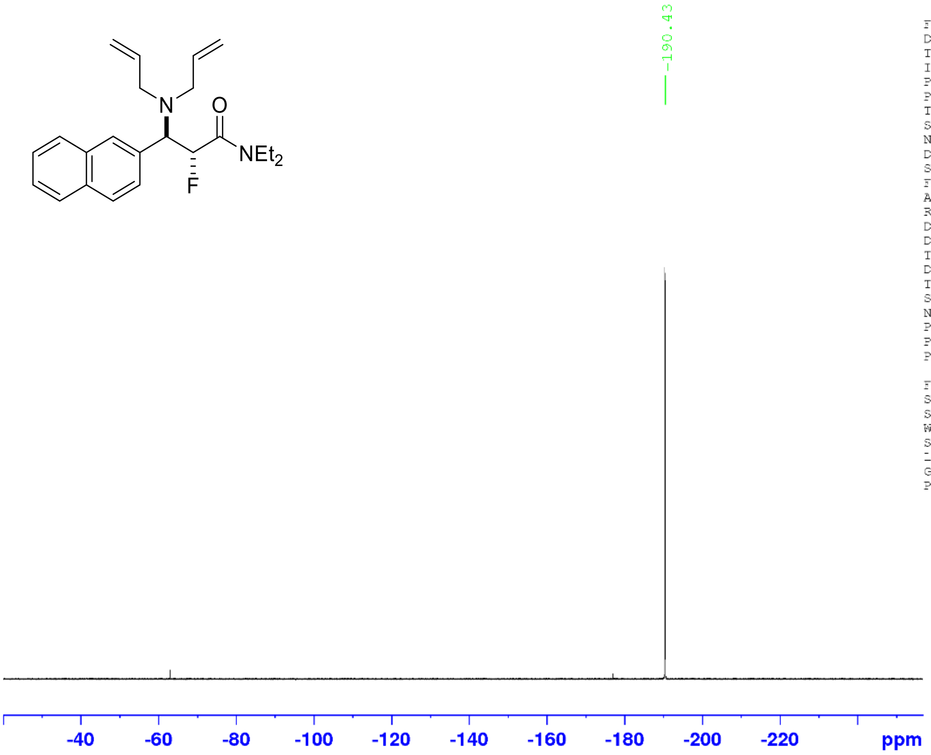

F2 - Acquisition Parameters
Date_ 20230324
Time 1.50 h
INSTRUM avx500
PROBHD Z119877_0007 (
PULPROG zg30
TD 131072
SOLVENT CDCl3
NS 256
DS 4
SWH 113636.367 Hz
FIDRES 1.733953 Hz
AQ 0.5767168 sec
RG 191.37
DW 4.400 usec
DE 6.50 usec
TE 298.0 K
D1 1.00000000 sec
TD0 1
SFO1 470.6864318 MHz
NUC1 19F
P0 7.33 usec
P1 22.00 usec
PLW1 34.09799957 W

F2 - Processing parameters
SI 65536
SF 470.7523370 MHz
WDW EM
SSB 0
LB 0.30 Hz
GB 0
PC 1.00

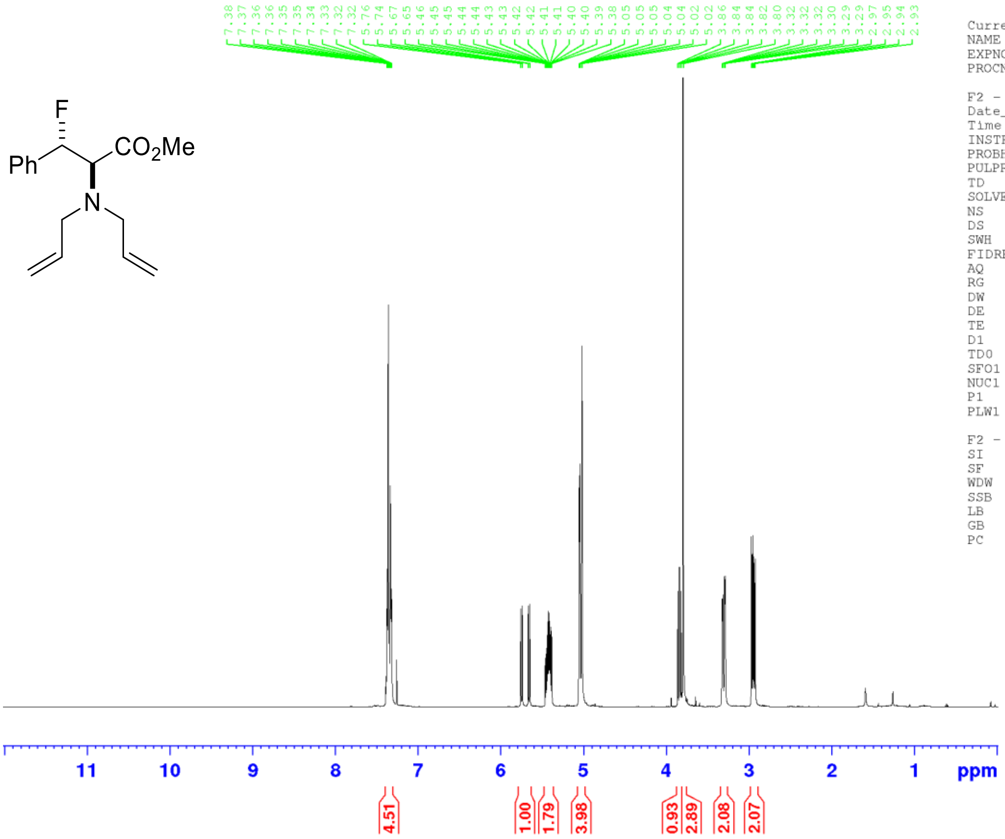

Current Data Parameters
NAME mah-216E-1H
EXPNO 1
PROCNO 1

F2 - Acquisition Parameters
Date_ 20190726
Time 11.14 h
INSTRUM avb500
PROBHD Z113652_0208 (
PULPROG zg60
TD 65536
SOLVENT CDCl3
NS 16
DS 2
SWH 10330.578 Hz
FIDRES 0.315264 Hz
AQ 3.1719425 sec
RG 25.4
DW 48.400 usec
DE 6.50 usec
TE 298.0 K
D1 1.00000000 sec
TD0 1
SFO1 499.9825000 MHz
NUC1 1H
P1 10.00 usec
PLW1 18.20400047 W

F2 - Processing parameters
SI 32768
SF 499.9800120 MHz
WDW EM
SSB 0
LB 0.30 Hz
GB 0
PC 1.00

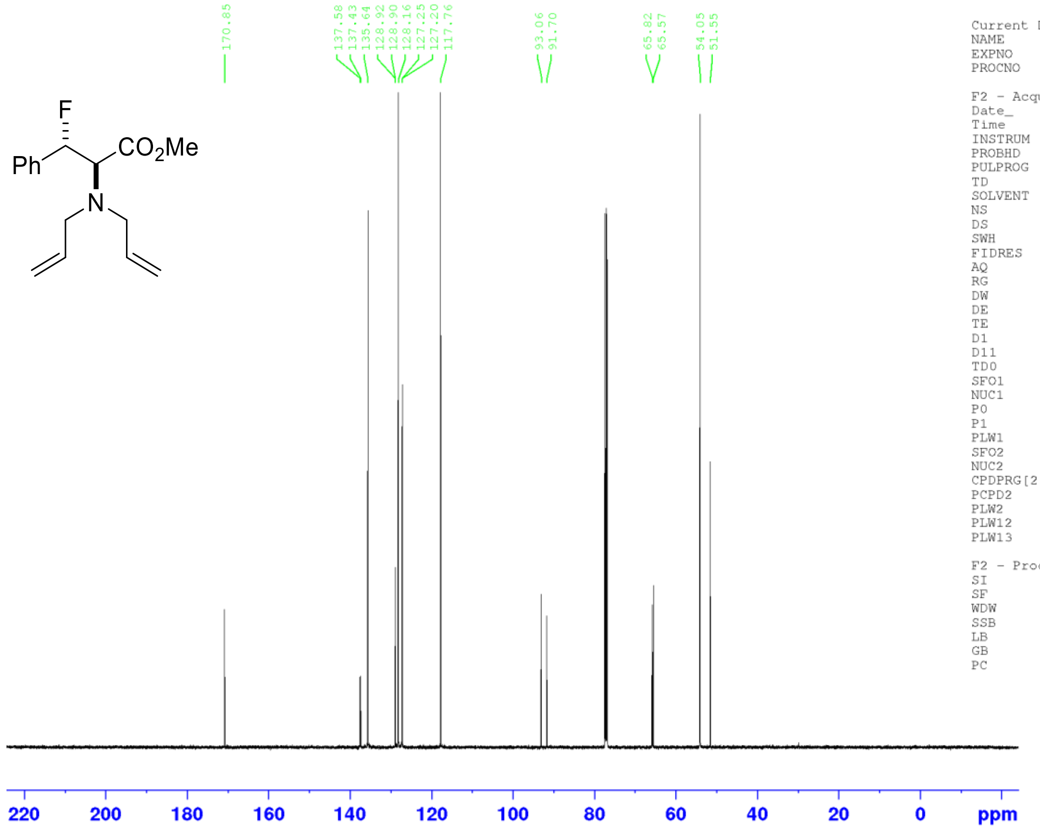


```
Current Data Parameters
NAME          mah-216E-13C
EXPNO                    3
PROCNO                   1

F2 - Acquisition Parameters
Date_             20190726
Time                 12.08 h
INSTRUM             avb500
PROBHD    Z113652_0208 (
PULPROG            zgpg30
TD                   65536
SOLVENT              CDCl3
NS                    1024
DS                       2
SWH              31250.000 Hz
FIDRES            0.953674 Hz
AQ               1.0485760 sec
RG                     203
DW                  16.000 usec
DE                    6.50 usec
TE                   299.0 K
D1              2.00000000 sec
D11             0.03000000 sec
TD0                      1
SFO1           125.7326430 MHz
NUC1                   13C
P0                    3.33 usec
P1                   10.00 usec
PLW1           81.90399933 W
SFO2           499.9824999 MHz
NUC2                    1H
CPDPRG[2           waltz16
PCPD2                80.00 usec
PLW2           18.20400047 W
PLW12           0.28444001 W
PLW13           0.14307000 W

F2 - Processing parameters
SI                   32768
SF             125.7200558 MHz
WDW                     EM
SSB       0
LB                    1.00 Hz
GB        0
PC                    1.40
```

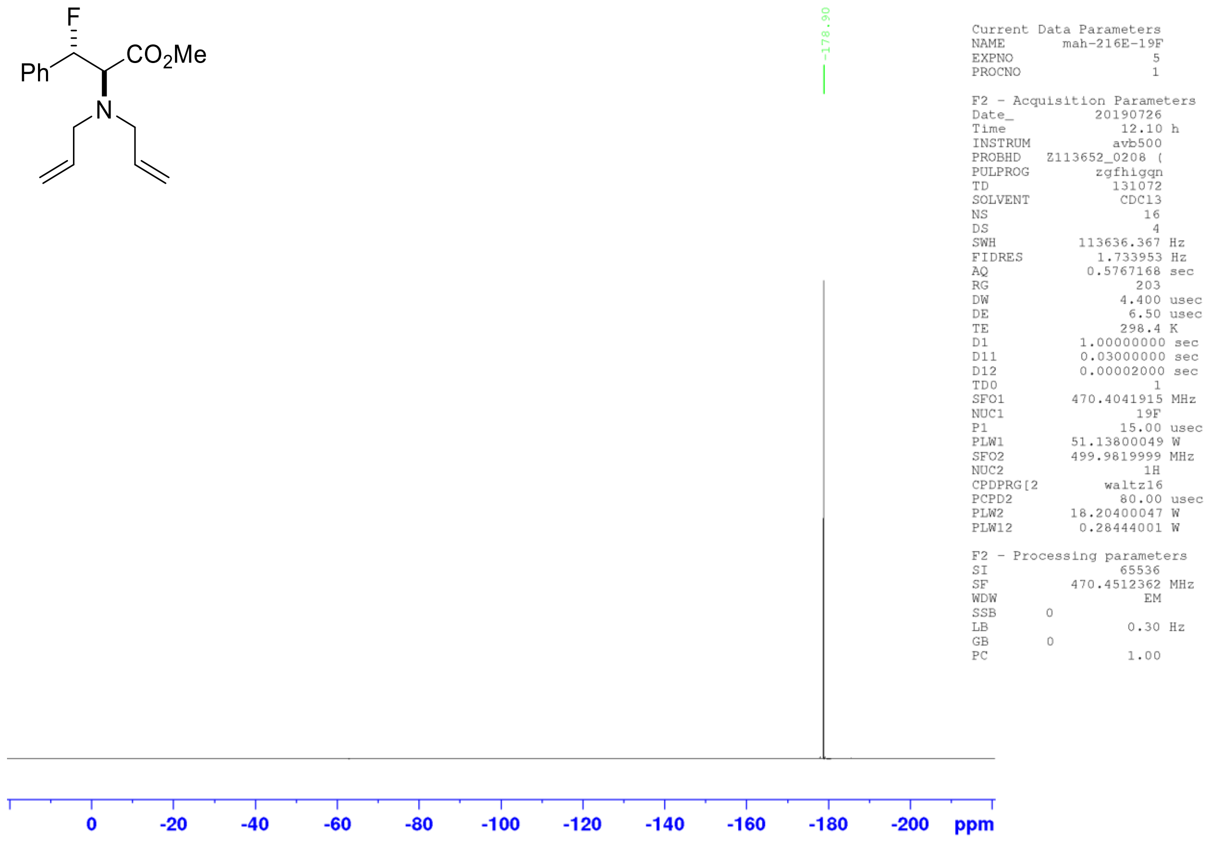


```
Current Data Parameters
NAME          mah-216E-19F
EXPNO                    5
PROCNO                   1

F2 - Acquisition Parameters
Date_             20190726
Time                 12.10 h
INSTRUM             avb500
PROBHD    Z113652_0208 (
PULPROG           zgfhigqn
TD                  131072
SOLVENT              CDCl3
NS                      16
DS                       4
SWH             113636.367 Hz
FIDRES            1.733953 Hz
AQ               0.5767168 sec
RG                     203
DW                   4.400 usec
DE                    6.50 usec
TE                   298.4 K
D1              1.00000000 sec
D11             0.03000000 sec
D12             0.00002000 sec
TD0                      1
SFO1           470.4041915 MHz
NUC1                   19F
P1                   15.00 usec
PLW1           51.13800049 W
SFO2           499.9819999 MHz
NUC2                    1H
CPDPRG[2           waltz16
PCPD2                80.00 usec
PLW2           18.20400047 W
PLW12           0.28444001 W

F2 - Processing parameters
SI                   65536
SF             470.4512362 MHz
WDW                     EM
SSB       0
LB                    0.30 Hz
GB        0
PC                    1.00
```

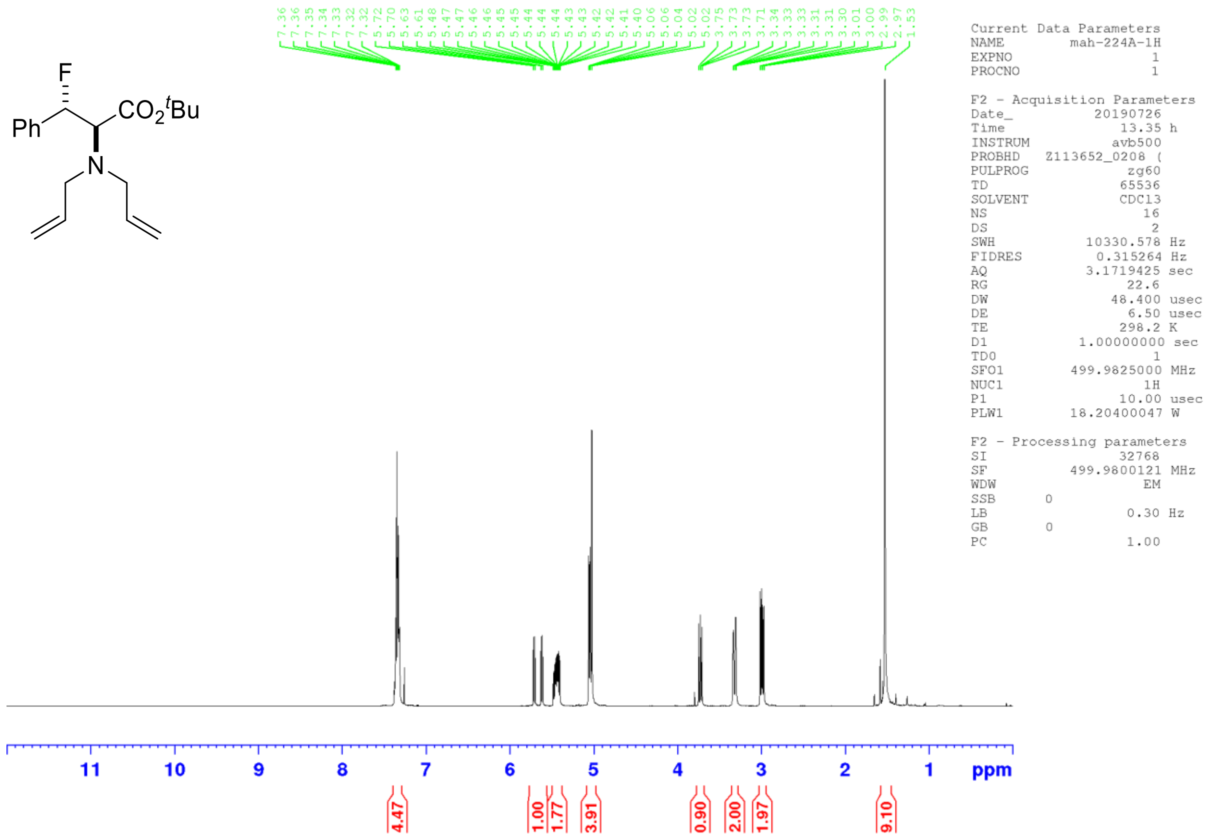
F
Ph
CO2tBu
N
7.36 7.36 7.35 7.34 7.33 7.32 7.32 5.72 5.70 5.63 5.61 5.48 5.47 5.47 5.46 5.46 5.45 5.45 5.44 5.44 5.44 5.43 5.43 5.42 5.42 5.41 5.40 5.06 5.06 5.04 5.02 5.02 3.75 3.73 3.73 3.71 3.34 3.33 3.33 3.31 3.31 3.30 3.01 3.00 2.99 2.97 1.53
11 10 9 8 7 6 5 4 3 2 1 ppm
4.47 1.00 1.77 3.91 0.90 2.00 1.97 9.10
Current Data Parameters
NAME mah-224A-1H
EXPNO 1
PROCNO 1
F2 - Acquisition Parameters
Date_ 20190726
Time 13.35 h
INSTRUM avb500
PROBHD Z113652_0208 (
PULPROG zg60
TD 65536
SOLVENT CDCl3
NS 16
DS 2
SWH 10330.578 Hz
FIDRES 0.315264 Hz
AQ 3.1719425 sec
RG 22.6
DW 48.400 usec
DE 6.50 usec
TE 298.2 K
D1 1.00000000 sec
TD0 1
SFO1 499.9825000 MHz
NUC1 1H
P1 10.00 usec
PLW1 18.20400047 W
F2 - Processing parameters
SI 32768
SF 499.9800121 MHz
WDW EM
SSB 0
LB 0.30 Hz
GB 0
PC 1.00

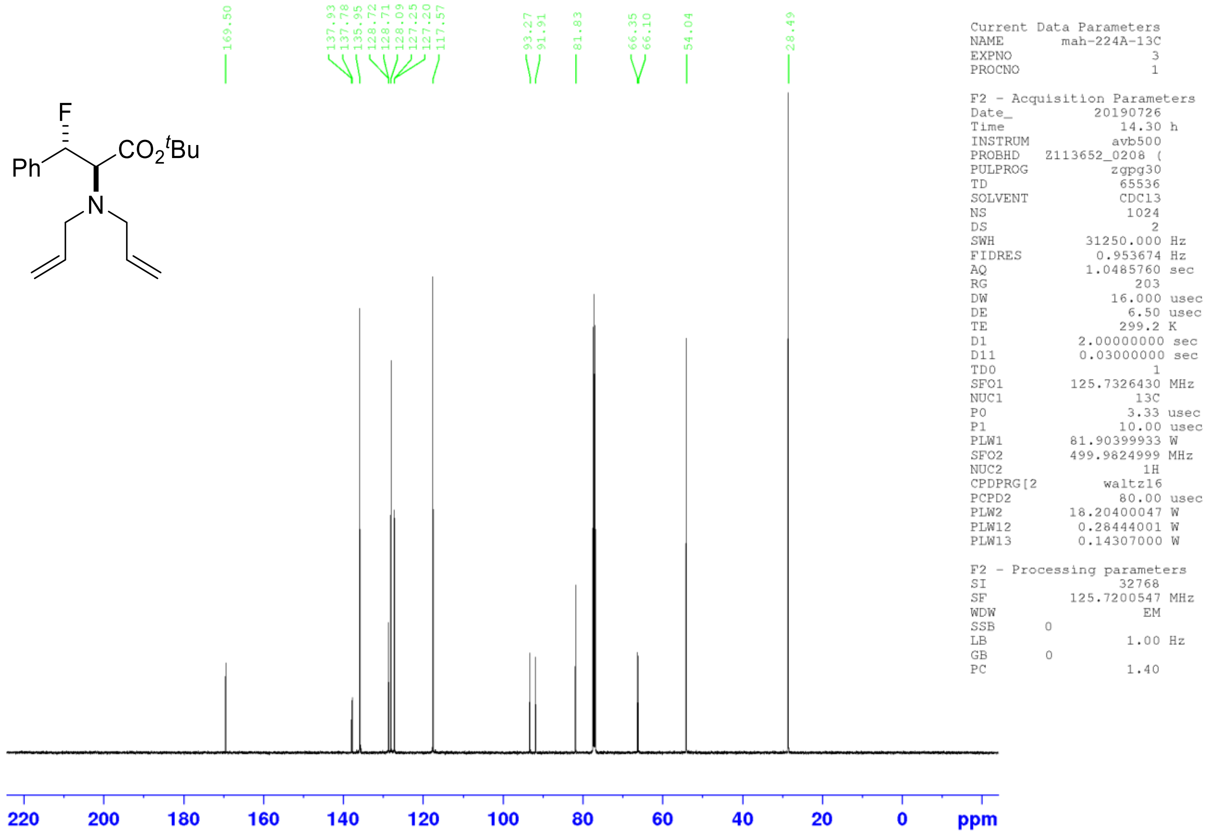
F
Ph
CO2tBu
N
169.50
137.93 137.78 135.95 128.72 128.71 128.09 127.25 127.20 117.57
93.27 91.91
81.83
66.35 66.10
54.04
28.49
220 200 180 160 140 120 100 80 60 40 20 0 ppm
Current Data Parameters
NAME mah-224A-13C
EXPNO 3
PROCNO 1
F2 - Acquisition Parameters
Date_ 20190726
Time 14.30 h
INSTRUM avb500
PROBHD Z113652_0208 (
PULPROG zgpg30
TD 65536
SOLVENT CDCl3
NS 1024
DS 2
SWH 31250.000 Hz
FIDRES 0.953674 Hz
AQ 1.0485760 sec
RG 203
DW 16.000 usec
DE 6.50 usec
TE 299.2 K
D1 2.00000000 sec
D11 0.03000000 sec
TD0 1
SFO1 125.7326430 MHz
NUC1 13C
P0 3.33 usec
P1 10.00 usec
PLW1 81.90399933 W
SFO2 499.9824999 MHz
NUC2 1H
CPDPRG[2 waltz16
PCPD2 80.00 usec
PLW2 18.20400047 W
PLW12 0.28444001 W
PLW13 0.14307000 W
F2 - Processing parameters
SI 32768
SF 125.7200547 MHz
WDW EM
SSB 0
LB 1.00 Hz
GB 0
PC 1.40

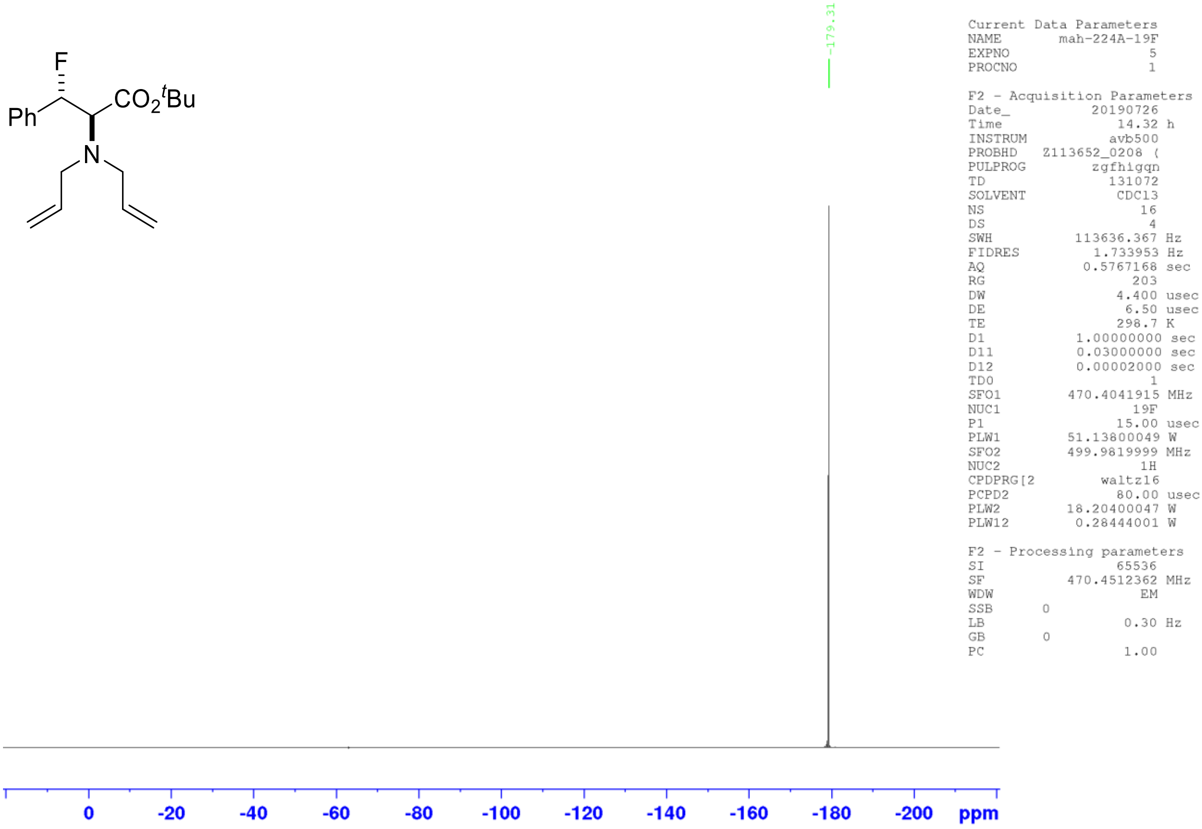


```
Current Data Parameters
NAME          mah-224A-19F
EXPNO                    5
PROCNO                   1

F2 - Acquisition Parameters
Date_             20190726
Time                 14.32 h
INSTRUM             avb500
PROBHD    Z113652_0208 (
PULPROG           zgfhigqn
TD                  131072
SOLVENT              CDCl3
NS                      16
DS                       4
SWH            113636.367 Hz
FIDRES            1.733953 Hz
AQ               0.5767168 sec
RG                     203
DW                   4.400 usec
DE                    6.50 usec
TE                   298.7 K
D1              1.00000000 sec
D11             0.03000000 sec
D12             0.00002000 sec
TD0                      1
SFO1           470.4041915 MHz
NUC1                   19F
P1                   15.00 usec
PLW1           51.13800049 W
SFO2           499.9819999 MHz
NUC2                    1H
CPDPRG[2           waltz16
PCPD2                80.00 usec
PLW2           18.20400047 W
PLW12           0.28444001 W

F2 - Processing parameters
SI                   65536
SF             470.4512362 MHz
WDW                     EM
SSB      0
LB                    0.30 Hz
GB       0
PC                    1.00
```

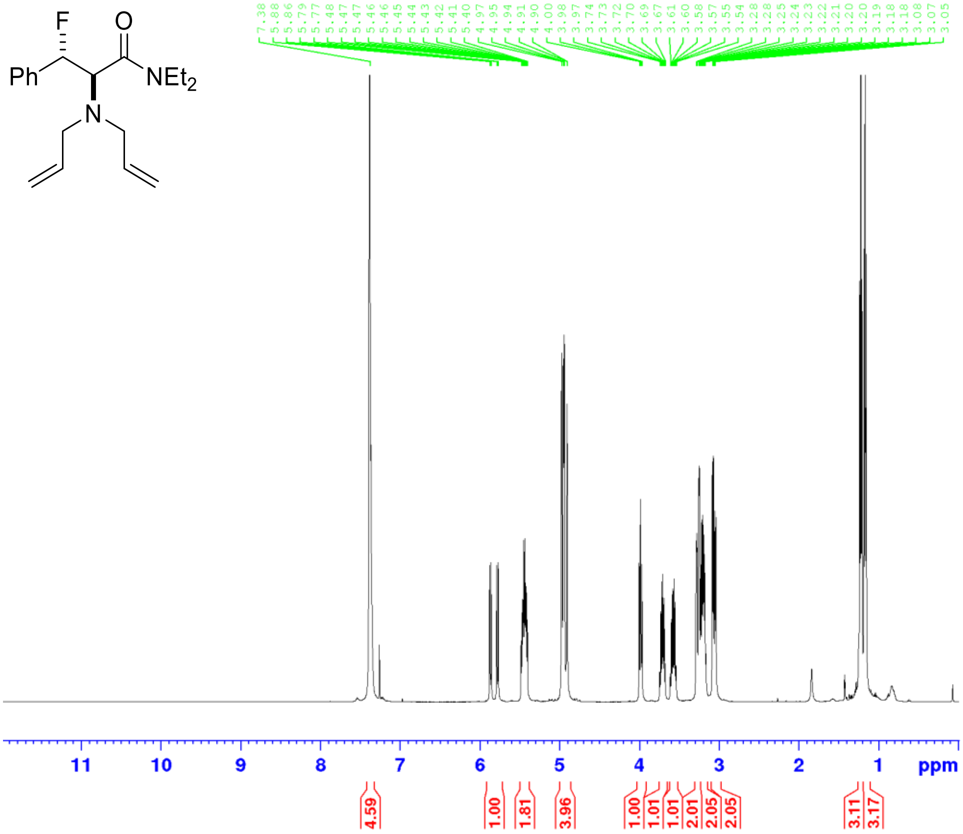


```
Current Data Parameters
NAME               mh-227B
EXPNO                    1
PROCNO                   1

F2 - Acquisition Parameters
Date_             20190814
Time                 18.26 h
INSTRUM             avb500
PROBHD    Z113652_0208 (
PULPROG               zg60
TD                   65536
SOLVENT              CDCl3
NS                      16
DS                       2
SWH              10330.578 Hz
FIDRES            0.315264 Hz
AQ               3.1719425 sec
RG                      18
DW                  48.400 usec
DE                    6.50 usec
TE                   294.2 K
D1              1.00000000 sec
TD0                      1
SFO1           499.9825000 MHz
NUC1                    1H
P1                   10.00 usec
PLW1           18.20400047 W

F2 - Processing parameters
SI                   32768
SF             499.9800123 MHz
WDW                     EM
SSB      0
LB                    0.30 Hz
GB       0
PC                    1.00
```

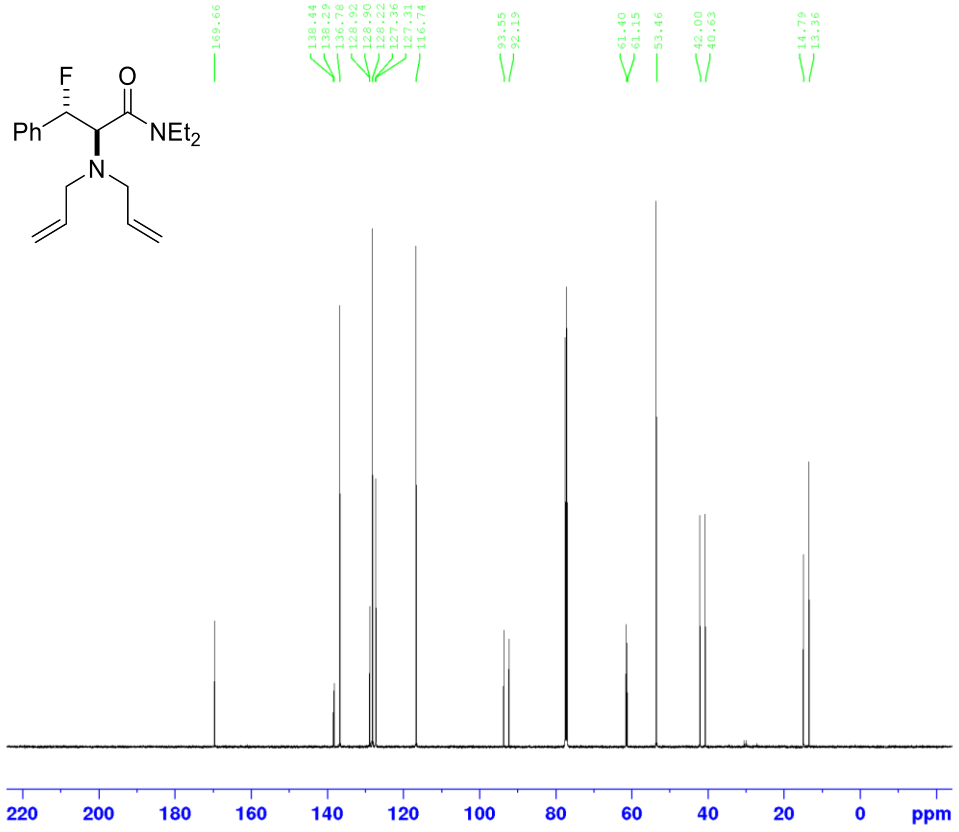

Current Data Parameters
NAME mh-227B
EXPNO 2
PROCNO 1

F2 - Acquisition Parameters
Date_ 20190814
Time 19.20 h
INSTRUM avb500
PROBHD Z113652_0208 (
PULPROG zgpg30
TD 65536
SOLVENT CDCl3
NS 1024
DS 2
SWH 31250.000 Hz
FIDRES 0.953674 Hz
AQ 1.0485760 sec
RG 203
DW 16.000 usec
DE 6.50 usec
TE 294.2 K
D1 2.00000000 sec
D11 0.03000000 sec
TD0 1
SFO1 125.7326430 MHz
NUC1 13C
P0 3.33 usec
P1 10.00 usec
PLW1 81.90399933 W
SFO2 499.9824999 MHz
NUC2 1H
CPDPRG[2 waltz16
PCPD2 80.00 usec
PLW2 18.20400047 W
PLW12 0.28444001 W
PLW13 0.14307000 W

F2 - Processing parameters
SI 32768
SF 125.7200602 MHz
WDW EM
SSB 0
LB 1.00 Hz
GB 0
PC 1.40

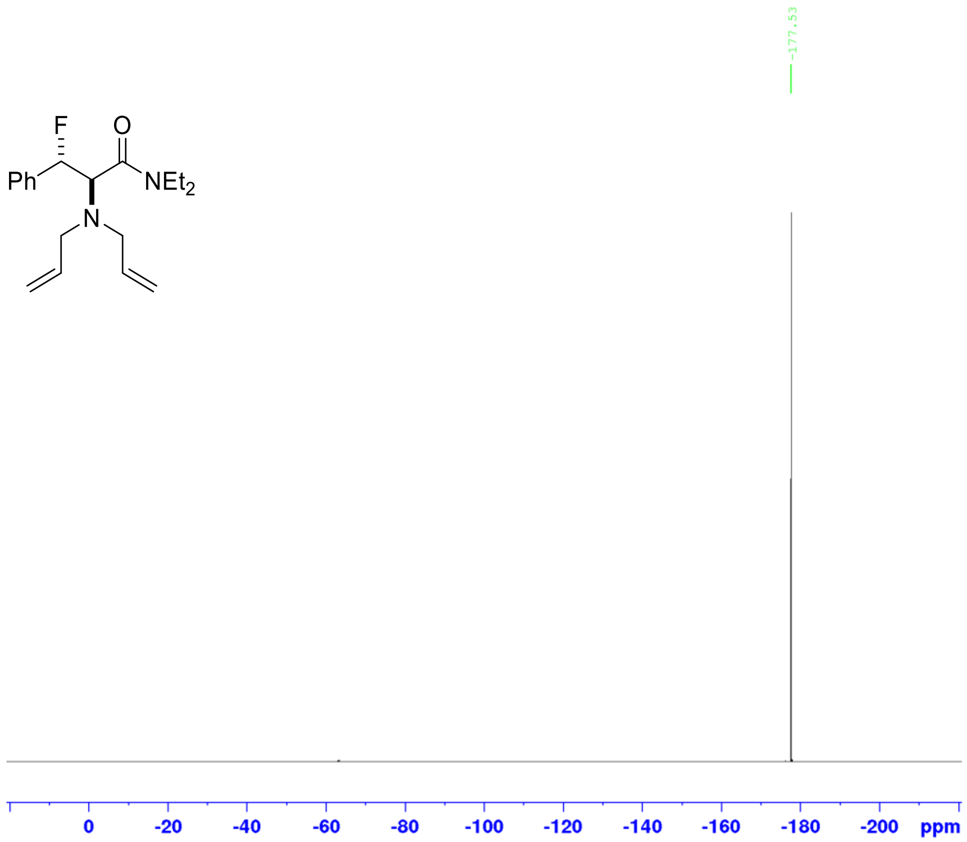

Current Data Parameters
NAME mh-227 B F dec
EXPNO 7
PROCNO 1

F2 - Acquisition Parameters
Date_ 20190819
Time 17.26 h
INSTRUM avb500
PROBHD Z113652_0208 (
PULPROG zgfhigqn
TD 131072
SOLVENT CDCl3
NS 16
DS 4
SWH 113636.367 Hz
FIDRES 1.733953 Hz
AQ 0.5767168 sec
RG 203
DW 4.400 usec
DE 6.50 usec
TE 298.1 K
D1 1.00000000 sec
D11 0.03000000 sec
D12 0.00002000 sec
TD0 1
SFO1 470.4041915 MHz
NUC1 19F
P1 15.00 usec
PLW1 51.13800049 W
SFO2 499.9819999 MHz
NUC2 1H
CPDPRG[2 waltz16
PCPD2 80.00 usec
PLW2 18.20400047 W
PLW12 0.28444001 W

F2 - Processing parameters
SI 65536
SF 470.4512362 MHz
WDW EM
SSB 0
LB 0.30 Hz
GB 0
PC 1.00

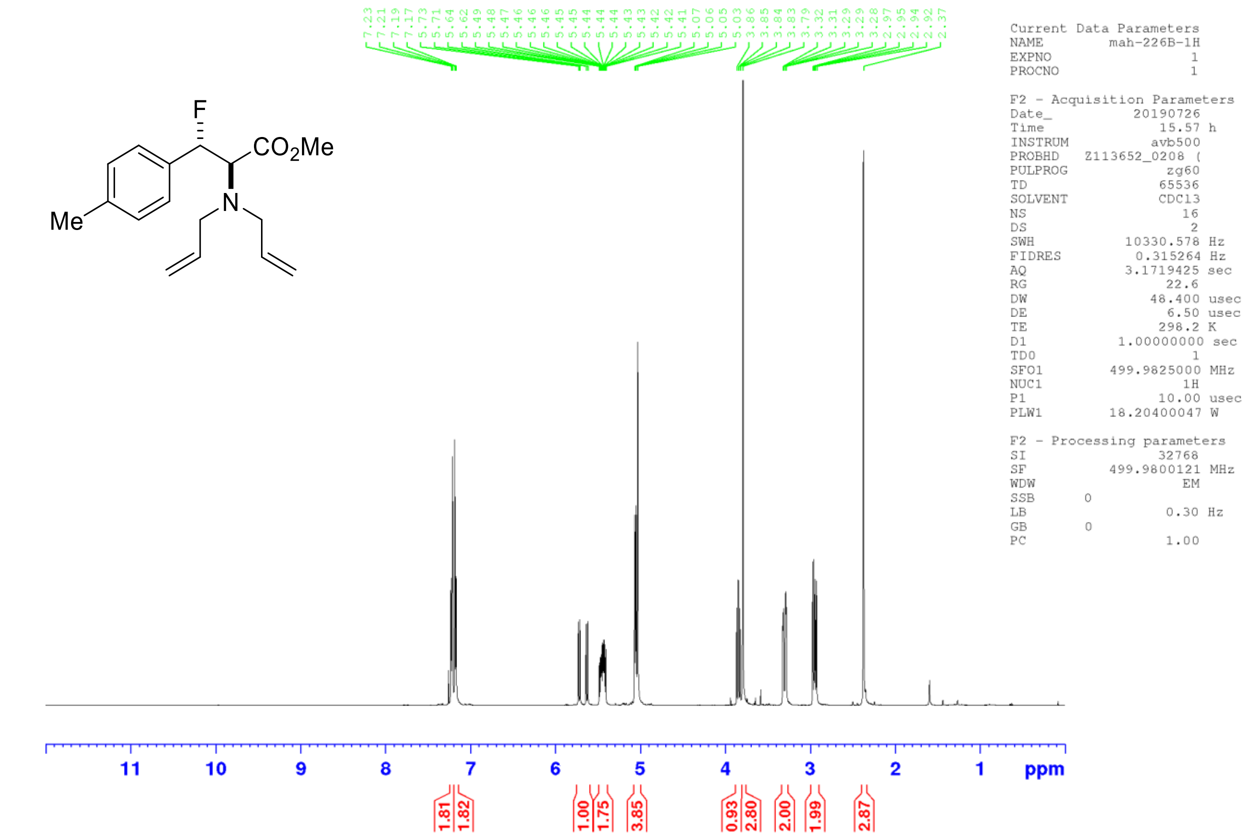
F
CO2Me
Me
N
7.23 7.21 7.19 7.17 5.73 5.71 5.64 5.62 5.49 5.48 5.47 5.46 5.46 5.46 5.45 5.45 5.44 5.44 5.44 5.43 5.43 5.42 5.42 5.41 5.07 5.06 5.05 5.03 3.86 3.85 3.84 3.83 3.79 3.32 3.31 3.29 3.29 3.28 2.97 2.95 2.94 2.92 2.37
11 10 9 8 7 6 5 4 3 2 1 ppm
1.81 1.82 1.00 1.75 3.85 0.93 2.80 2.00 1.99 2.87
Current Data Parameters
NAME mah-226B-1H
EXPNO 1
PROCNO 1
F2 - Acquisition Parameters
Date_ 20190726
Time 15.57 h
INSTRUM avb500
PROBHD Z113652_0208 (
PULPROG zg60
TD 65536
SOLVENT CDCl3
NS 16
DS 2
SWH 10330.578 Hz
FIDRES 0.315264 Hz
AQ 3.1719425 sec
RG 22.6
DW 48.400 usec
DE 6.50 usec
TE 298.2 K
D1 1.00000000 sec
TD0 1
SFO1 499.9825000 MHz
NUC1 1H
P1 10.00 usec
PLW1 18.20400047 W
F2 - Processing parameters
SI 32768
SF 499.9800121 MHz
WDW EM
SSB 0
LB 0.30 Hz
GB 0
PC 1.00

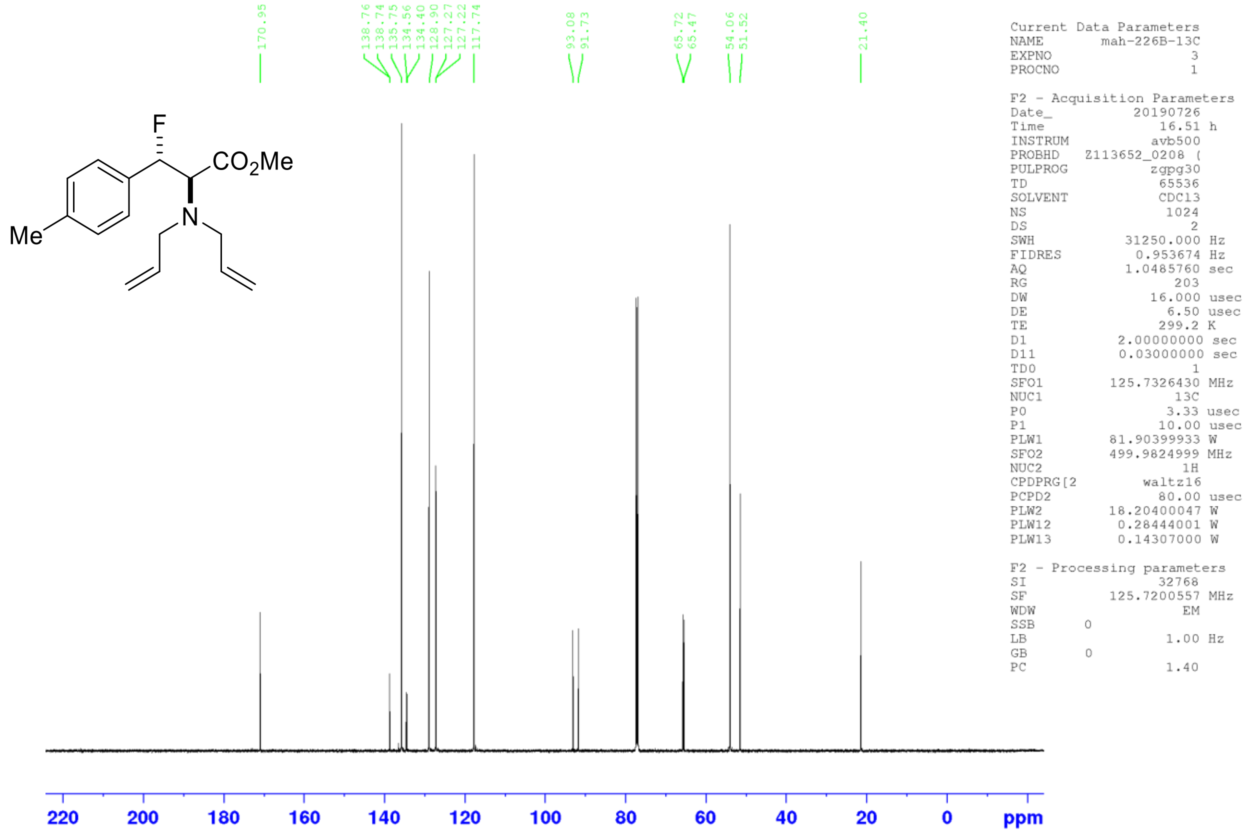
F
CO2Me
Me
N
170.95
138.76 138.74 135.75 134.56 134.40 128.90 127.27 127.22 117.74
93.08 91.73
65.72 65.47
54.06 51.52
21.40
220 200 180 160 140 120 100 80 60 40 20 0 ppm
Current Data Parameters
NAME mah-226B-13C
EXPNO 3
PROCNO 1
F2 - Acquisition Parameters
Date_ 20190726
Time 16.51 h
INSTRUM avb500
PROBHD Z113652_0208 (
PULPROG zgpg30
TD 65536
SOLVENT CDCl3
NS 1024
DS 2
SWH 31250.000 Hz
FIDRES 0.953674 Hz
AQ 1.0485760 sec
RG 203
DW 16.000 usec
DE 6.50 usec
TE 299.2 K
D1 2.00000000 sec
D11 0.03000000 sec
TD0 1
SFO1 125.7326430 MHz
NUC1 13C
P0 3.33 usec
P1 10.00 usec
PLW1 81.90399933 W
SFO2 499.9824999 MHz
NUC2 1H
CPDPRG[2 waltz16
PCPD2 80.00 usec
PLW2 18.20400047 W
PLW12 0.28444001 W
PLW13 0.14307000 W
F2 - Processing parameters
SI 32768
SF 125.7200557 MHz
WDW EM
SSB 0
LB 1.00 Hz
GB 0
PC 1.40

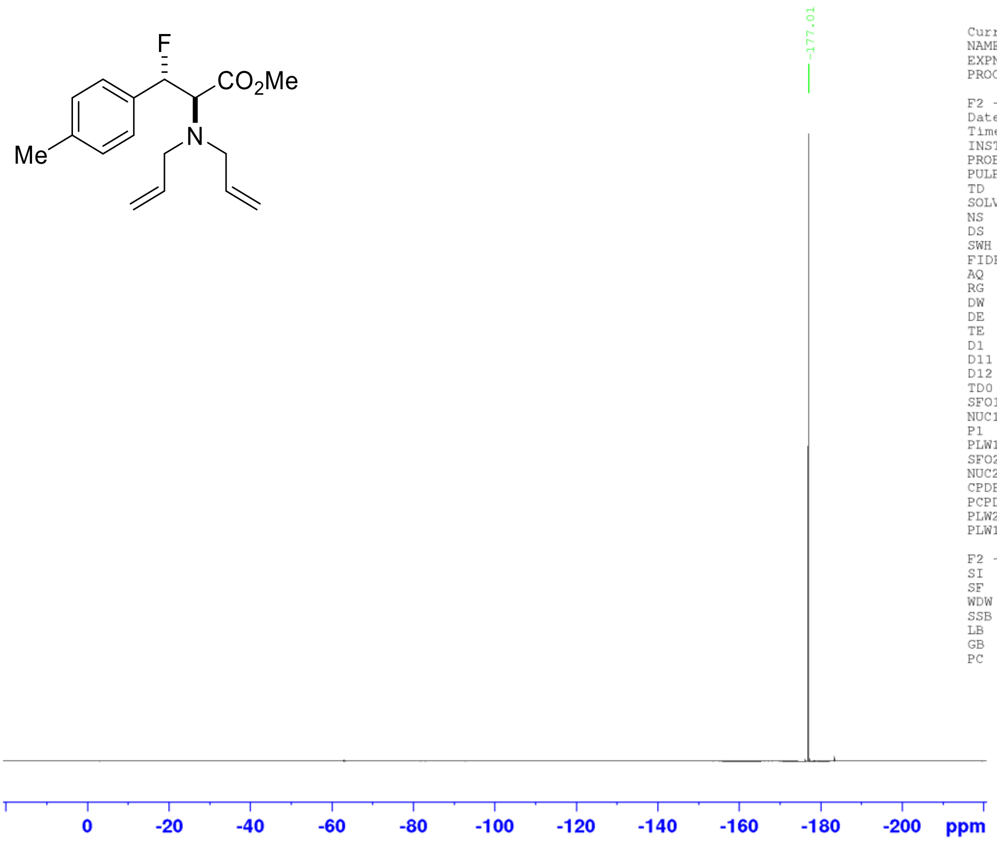


```
Current Data Parameters
NAME          mah-226B-19F
EXPNO                    5
PROCNO                   1

F2 - Acquisition Parameters
Date_             20190726
Time                 16.53 h
INSTRUM             avb500
PROBHD   Z113652_0208 (
PULPROG           zgfhigqn
TD                  131072
SOLVENT              CDCl3
NS                      16
DS                       4
SWH           113636.367 Hz
FIDRES          1.733953 Hz
AQ             0.5767168 sec
RG                     203
DW                   4.400 usec
DE                    6.50 usec
TE                   298.6 K
D1              1.00000000 sec
D11             0.03000000 sec
D12             0.00002000 sec
TD0                      1
SFO1        470.4041915 MHz
NUC1                   19F
P1                   15.00 usec
PLW1           51.13800049 W
SFO2        499.9819999 MHz
NUC2                    1H
CPDPRG[2           waltz16
PCPD2                80.00 usec
PLW2           18.20400047 W
PLW12           0.28444001 W

F2 - Processing parameters
SI                   65536
SF          470.4512362 MHz
WDW                     EM
SSB      0
LB                    0.30 Hz
GB       0
PC                    1.00
```

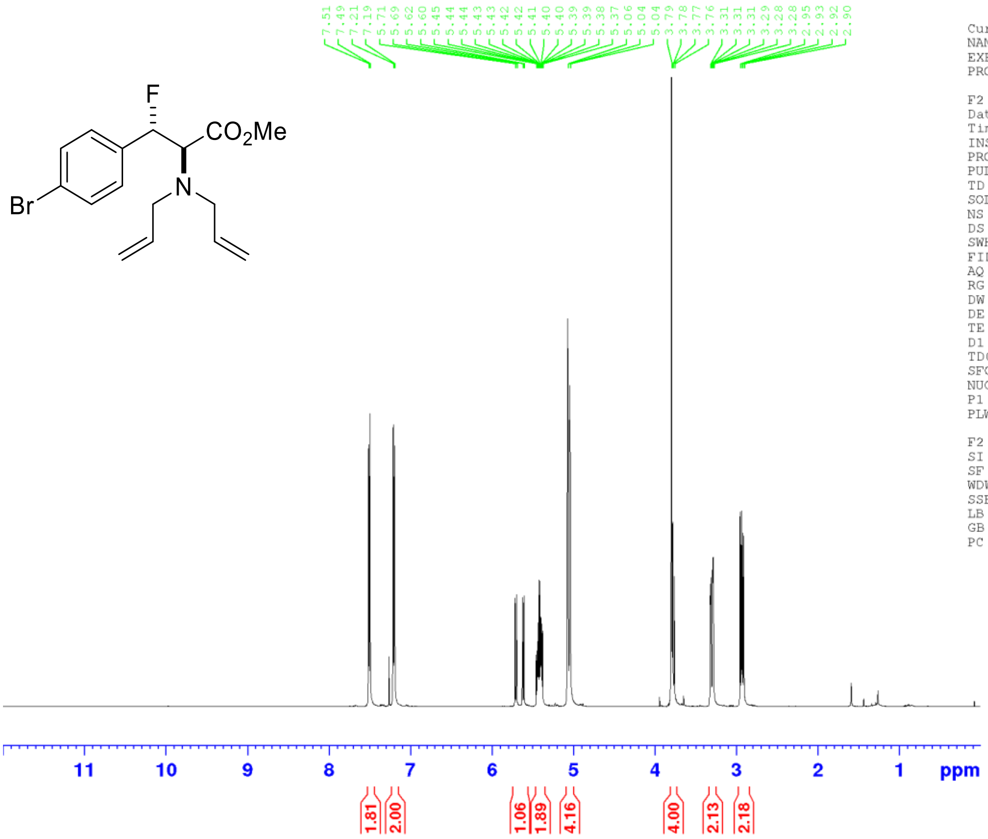


```
Current Data Parameters
NAME           mah-230A-1H
EXPNO                    1
PROCNO                   1

F2 - Acquisition Parameters
Date_             20190731
Time                 11.10 h
INSTRUM             avb500
PROBHD   Z113652_0208 (
PULPROG               zg60
TD                   65536
SOLVENT              CDCl3
NS                      16
DS                       2
SWH            10330.578 Hz
FIDRES          0.315264 Hz
AQ             3.1719425 sec
RG                    25.4
DW                  48.400 usec
DE                    6.50 usec
TE                   298.0 K
D1              1.00000000 sec
TD0                      1
SFO1        499.9825000 MHz
NUC1                    1H
P1                   10.00 usec
PLW1           18.20400047 W

F2 - Processing parameters
SI                   32768
SF          499.9800119 MHz
WDW                     EM
SSB      0
LB                    0.30 Hz
GB       0
PC                    1.00
```

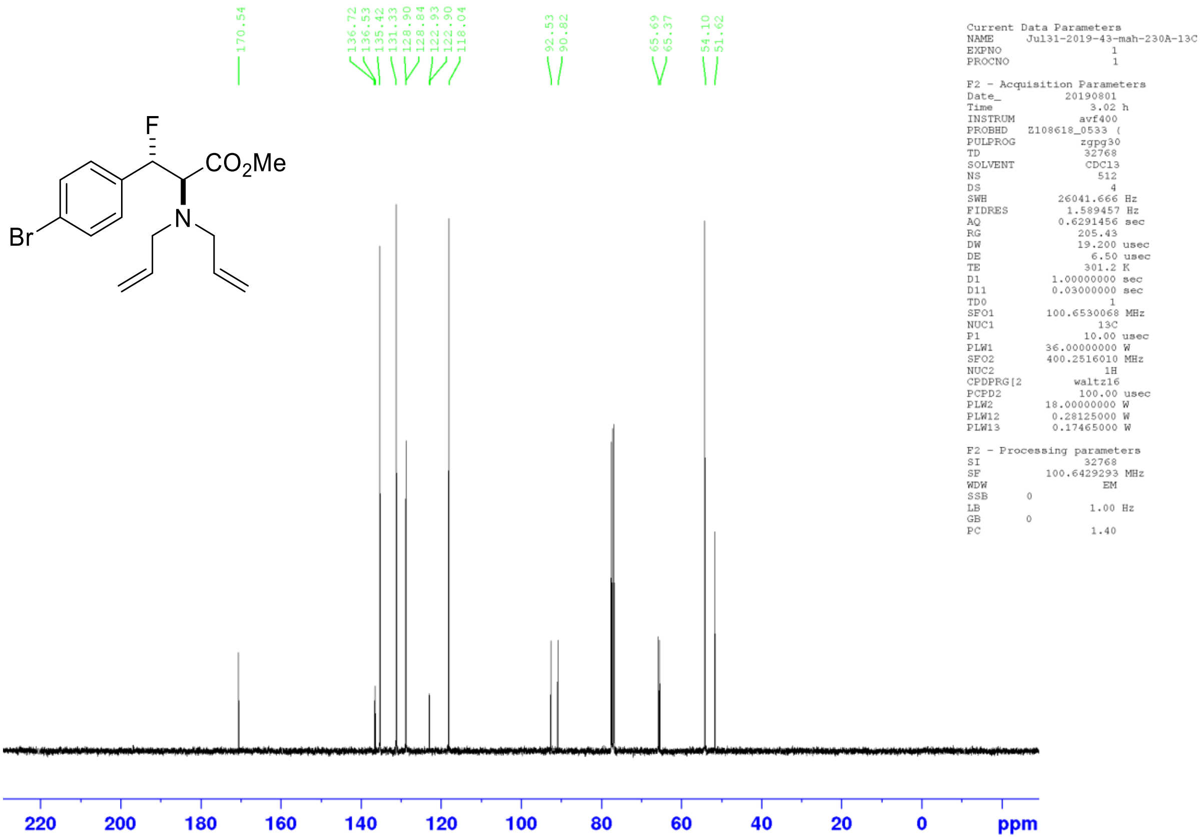
170.54
136.72
136.53
135.42
131.33
128.90
128.84
122.93
122.90
118.04
92.53
90.82
65.69
65.37
54.10
51.62
F
CO_2Me
N
Br
220 200 180 160 140 120 100 80 60 40 20 0 ppm
Current Data Parameters
NAME Jul31-2019-43-mah-230A-13C
EXPNO 1
PROCNO 1
F2 - Acquisition Parameters
Date_ 20190801
Time 3.02 h
INSTRUM avf400
PROBHD Z108618_0533 (
PULPROG zgpg30
TD 32768
SOLVENT CDCl3
NS 512
DS 4
SWH 26041.666 Hz
FIDRES 1.589457 Hz
AQ 0.6291456 sec
RG 205.43
DW 19.200 usec
DE 6.50 usec
TE 301.2 K
D1 1.00000000 sec
D11 0.03000000 sec
TD0 1
SFO1 100.6530068 MHz
NUC1 13C
P1 10.00 usec
PLW1 36.00000000 W
SFO2 400.2516010 MHz
NUC2 1H
CPDPRG[2 waltz16
PCPD2 100.00 usec
PLW2 18.00000000 W
PLW12 0.28125000 W
PLW13 0.17465000 W
F2 - Processing parameters
SI 32768
SF 100.6429293 MHz
WDW EM
SSB 0
LB 1.00 Hz
GB 0
PC 1.40

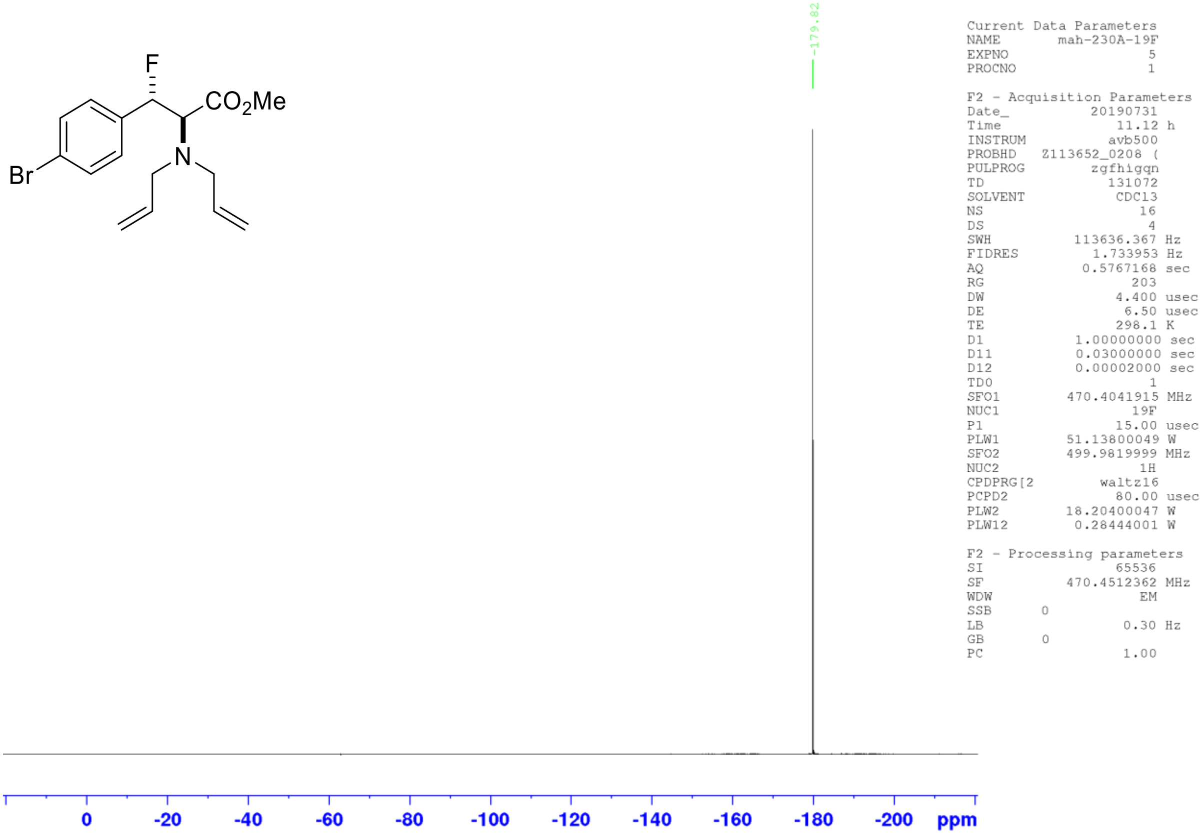
-179.82
F
CO_2Me
N
Br
0 -20 -40 -60 -80 -100 -120 -140 -160 -180 -200 ppm
Current Data Parameters
NAME mah-230A-19F
EXPNO 5
PROCNO 1
F2 - Acquisition Parameters
Date_ 20190731
Time 11.12 h
INSTRUM avb500
PROBHD Z113652_0208 (
PULPROG zgfhigqn
TD 131072
SOLVENT CDCl3
NS 16
DS 4
SWH 113636.367 Hz
FIDRES 1.733953 Hz
AQ 0.5767168 sec
RG 203
DW 4.400 usec
DE 6.50 usec
TE 298.1 K
D1 1.00000000 sec
D11 0.03000000 sec
D12 0.00002000 sec
TD0 1
SFO1 470.4041915 MHz
NUC1 19F
P1 15.00 usec
PLW1 51.13800049 W
SFO2 499.9819999 MHz
NUC2 1H
CPDPRG[2 waltz16
PCPD2 80.00 usec
PLW2 18.20400047 W
PLW12 0.28444001 W
F2 - Processing parameters
SI 65536
SF 470.4512362 MHz
WDW EM
SSB 0
LB 0.30 Hz
GB 0
PC 1.00

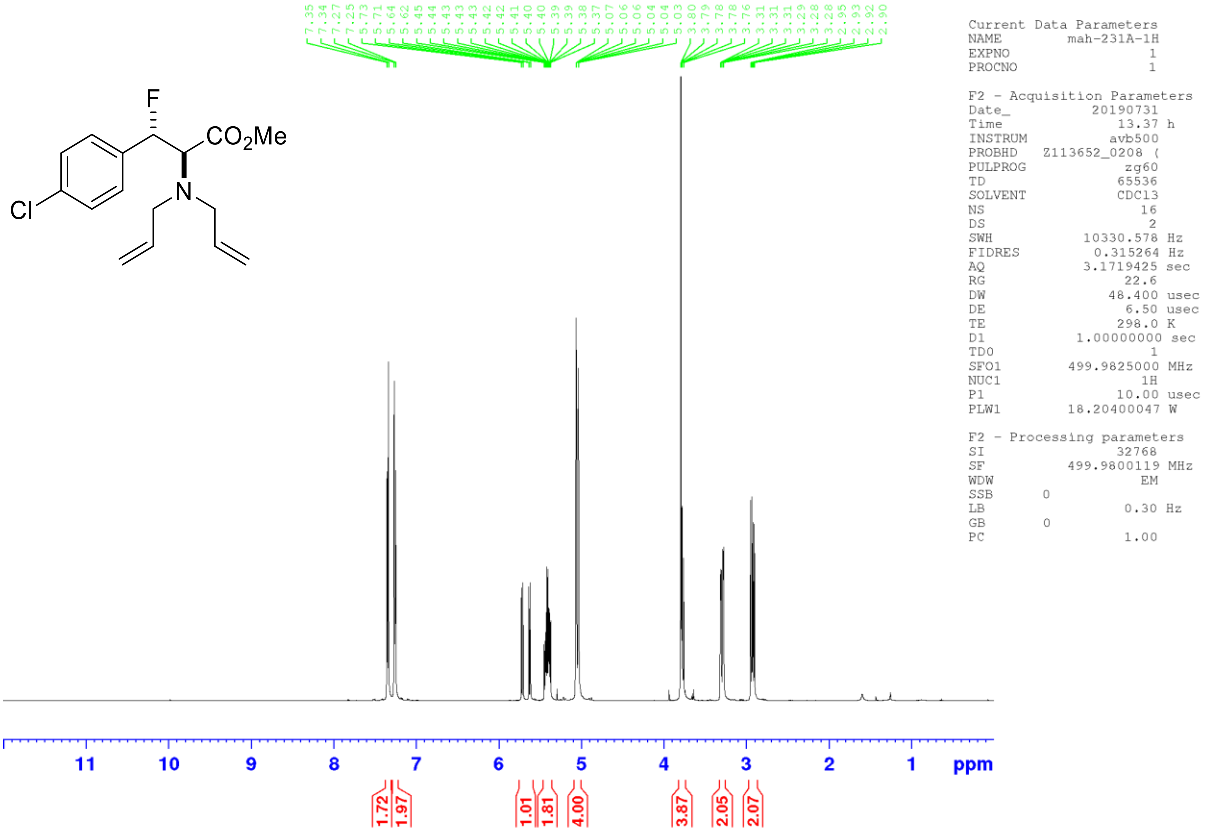
F
CO2Me
Cl
N
7.35 7.34 7.27 7.25 5.73 5.71 5.64 5.62 5.45 5.44 5.43 5.43 5.43 5.42 5.42 5.41 5.40 5.40 5.39 5.39 5.38 5.37 5.07 5.06 5.06 5.04 5.04 5.03 3.80 3.79 3.78 3.78 3.76 3.31 3.31 3.31 3.29 3.28 3.28 2.95 2.93 2.92 2.90
11 10 9 8 7 6 5 4 3 2 1 ppm
1.72 1.97 1.01 1.81 4.00 3.87 2.05 2.07
Current Data Parameters
NAME mah-231A-1H
EXPNO 1
PROCNO 1
F2 - Acquisition Parameters
Date_ 20190731
Time 13.37 h
INSTRUM avb500
PROBHD Z113652_0208 (
PULPROG zg60
TD 65536
SOLVENT CDCl3
NS 16
DS 2
SWH 10330.578 Hz
FIDRES 0.315264 Hz
AQ 3.1719425 sec
RG 22.6
DW 48.400 usec
DE 6.50 usec
TE 298.0 K
D1 1.00000000 sec
TD0 1
SFO1 499.9825000 MHz
NUC1 1H
P1 10.00 usec
PLW1 18.20400047 W
F2 - Processing parameters
SI 32768
SF 499.9800119 MHz
WDW EM
SSB 0
LB 0.30 Hz
GB 0
PC 1.00

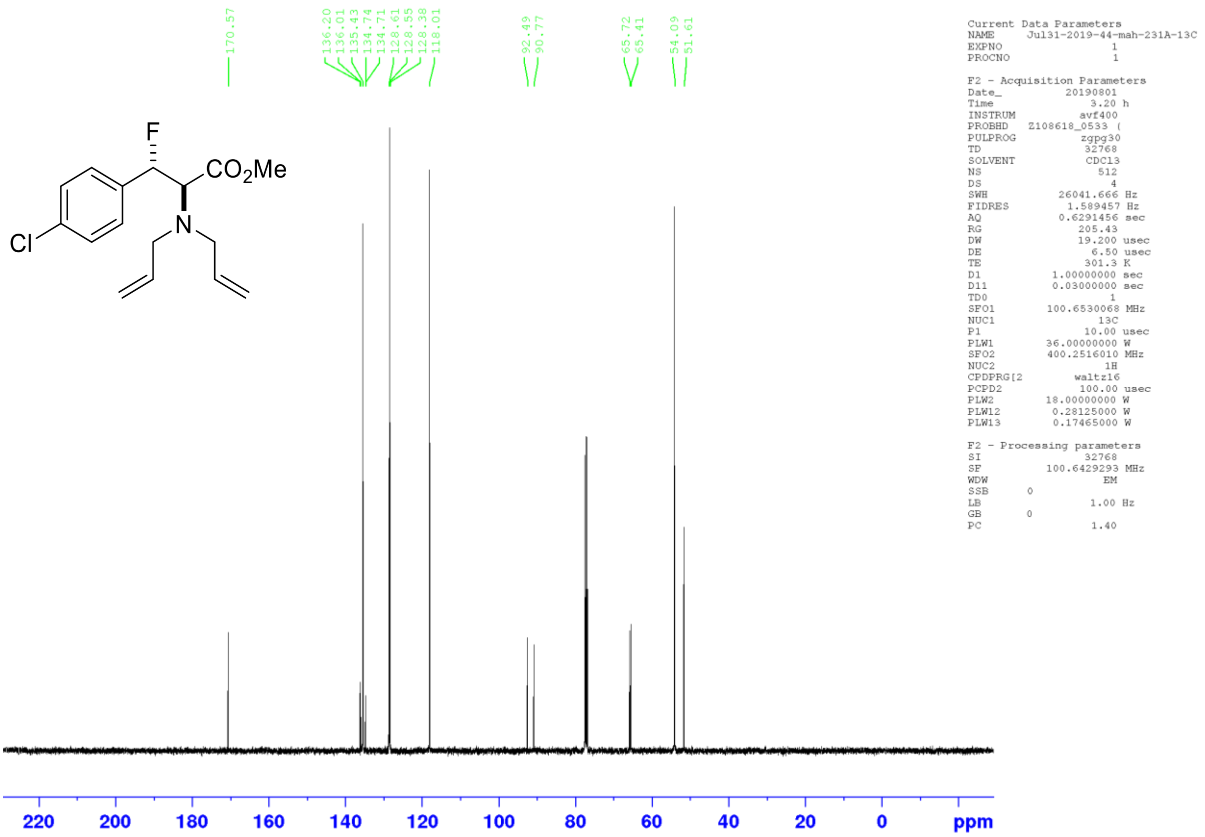
F
CO2Me
Cl
N
170.57
136.20 136.01 135.43 134.74 134.71 128.61 128.55 128.38 118.01
92.49 90.77
65.72 65.41
54.09 51.61
220 200 180 160 140 120 100 80 60 40 20 0 ppm
Current Data Parameters
NAME Jul31-2019-44-mah-231A-13C
EXPNO 1
PROCNO 1
F2 - Acquisition Parameters
Date_ 20190801
Time 3.20 h
INSTRUM avf400
PROBHD Z108618_0533 (
PULPROG zgpg30
TD 32768
SOLVENT CDCl3
NS 512
DS 4
SWH 26041.666 Hz
FIDRES 1.589457 Hz
AQ 0.6291456 sec
RG 205.43
DW 19.200 usec
DE 6.50 usec
TE 301.3 K
D1 1.00000000 sec
D11 0.03000000 sec
TD0 1
SFO1 100.6530068 MHz
NUC1 13C
P1 10.00 usec
PLW1 36.00000000 W
SFO2 400.2516010 MHz
NUC2 1H
CPDPRG[2 waltz16
PCPD2 100.00 usec
PLW2 18.00000000 W
PLW12 0.28125000 W
PLW13 0.17465000 W
F2 - Processing parameters
SI 32768
SF 100.6429293 MHz
WDW EM
SSB 0
LB 1.00 Hz
GB 0
PC 1.40

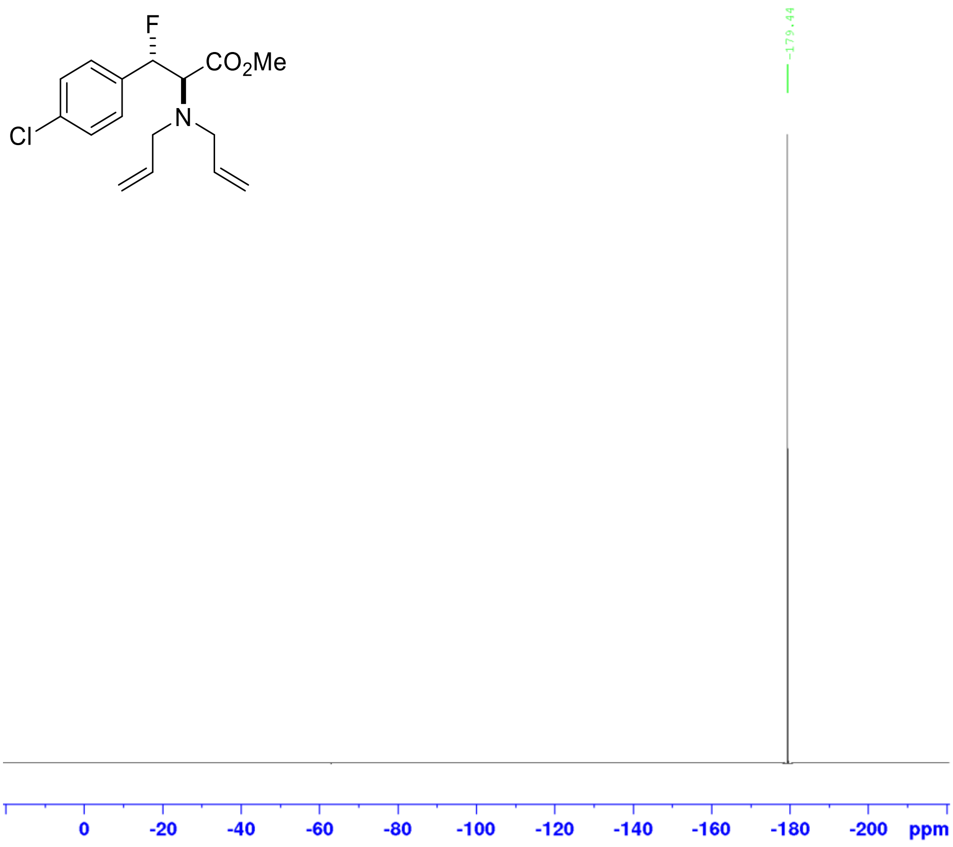


```
Current Data Parameters
NAME          mah-231A-19F
EXPNO                    5
PROCNO                   1

F2 - Acquisition Parameters
Date_             20190731
Time                 13.39 h
INSTRUM             avb500
PROBHD   Z113652_0208 (
PULPROG           zgfhigqn
TD                  131072
SOLVENT              CDCl3
NS                      16
DS                       4
SWH             113636.367 Hz
FIDRES            1.733953 Hz
AQ               0.5767168 sec
RG                     203
DW                   4.400 usec
DE                    6.50 usec
TE                   298.1 K
D1              1.00000000 sec
D11             0.03000000 sec
D12             0.00002000 sec
TD0                      1
SFO1           470.4041915 MHz
NUC1                   19F
P1                   15.00 usec
PLW1           51.13800049 W
SFO2           499.9819999 MHz
NUC2                    1H
CPDPRG[2           waltz16
PCPD2                80.00 usec
PLW2           18.20400047 W
PLW12           0.28444001 W

F2 - Processing parameters
SI                   65536
SF             470.4512362 MHz
WDW                     EM
SSB      0
LB                    0.30 Hz
GB       0
PC                    1.00
```

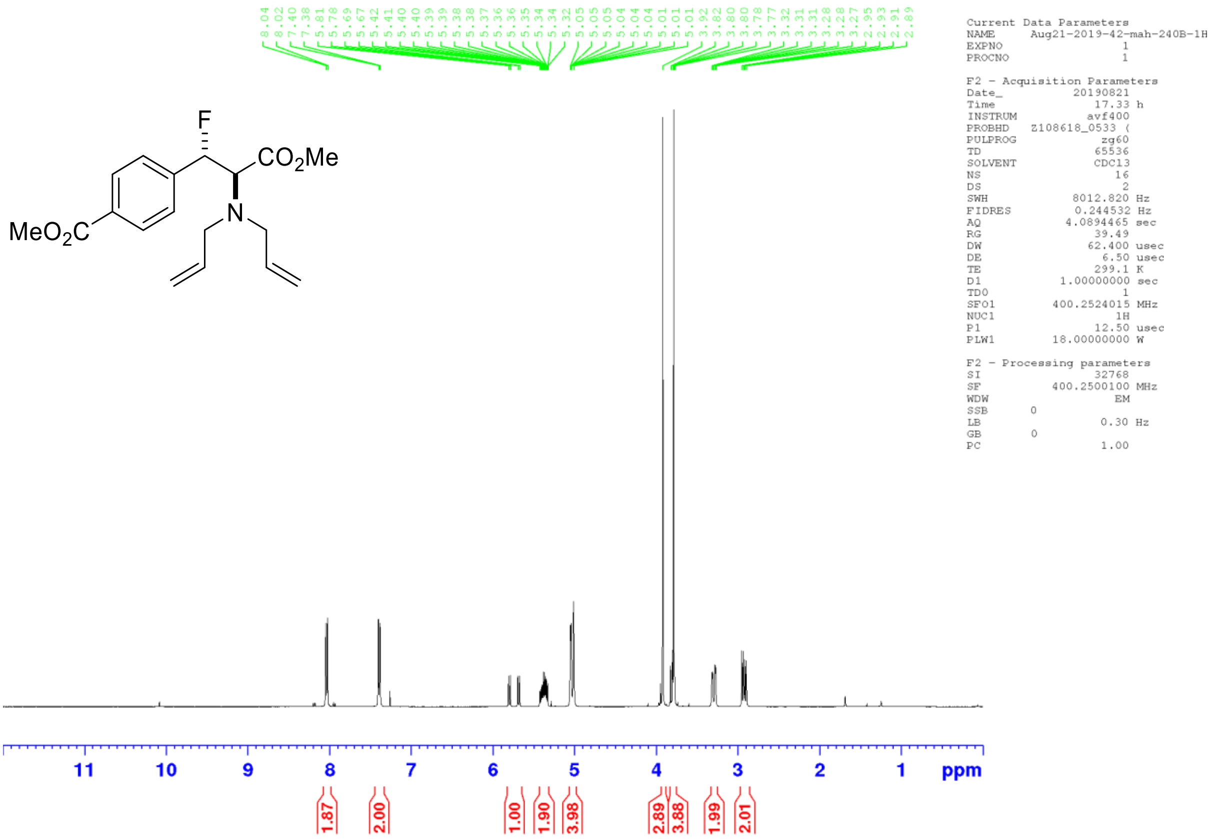


```
Current Data Parameters
NAME      Aug21-2019-42-mah-240B-1H
EXPNO                    1
PROCNO                   1

F2 - Acquisition Parameters
Date_             20190821
Time                 17.33 h
INSTRUM             avf400
PROBHD   Z108618_0533 (
PULPROG               zg60
TD                   65536
SOLVENT              CDCl3
NS                      16
DS                       2
SWH               8012.820 Hz
FIDRES            0.244532 Hz
AQ               4.0894465 sec
RG                   39.49
DW                  62.400 usec
DE                    6.50 usec
TE                   299.1 K
D1              1.00000000 sec
TD0                      1
SFO1           400.2524015 MHz
NUC1                    1H
P1                   12.50 usec
PLW1           18.00000000 W

F2 - Processing parameters
SI                   32768
SF             400.2500100 MHz
WDW                     EM
SSB      0
LB                    0.30 Hz
GB       0
PC                    1.00
```

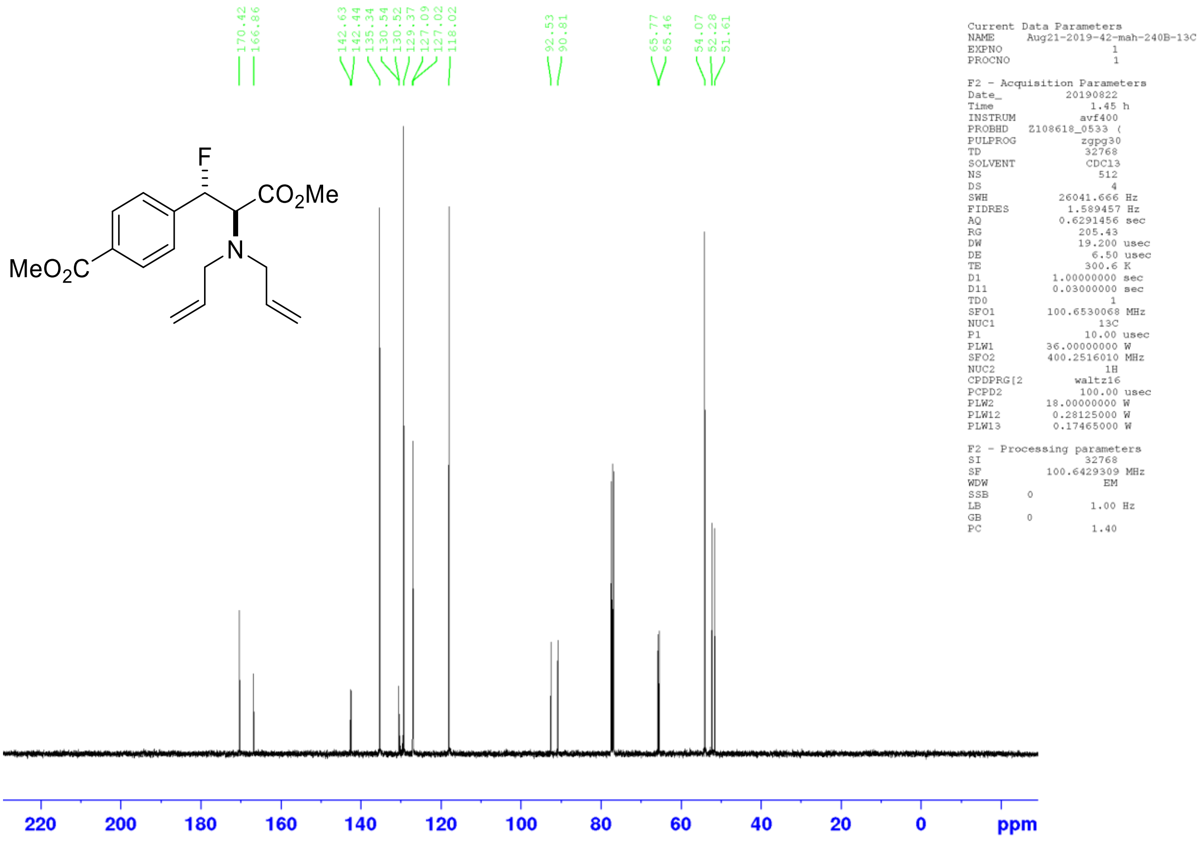
170.42
166.86
142.63
142.44
135.34
130.54
130.52
129.37
127.09
127.02
118.02
92.53
90.81
65.77
65.46
54.07
52.28
51.61
F
CO_2Me
MeO_2C
N
Current Data Parameters
NAME Aug21-2019-42-mah-240B-13C
EXPNO 1
PROCNO 1
F2 - Acquisition Parameters
Date_ 20190822
Time 1.45 h
INSTRUM avf400
PROBHD Z108618_0533 (
PULPROG zgpg30
TD 32768
SOLVENT CDCl3
NS 512
DS 4
SWH 26041.666 Hz
FIDRES 1.589457 Hz
AQ 0.6291456 sec
RG 205.43
DW 19.200 usec
DE 6.50 usec
TE 300.6 K
D1 1.00000000 sec
D11 0.03000000 sec
TD0 1
SFO1 100.6530068 MHz
NUC1 13C
P1 10.00 usec
PLW1 36.00000000 W
SFO2 400.2516010 MHz
NUC2 1H
CPDPRG[2 waltz16
PCPD2 100.00 usec
PLW2 18.00000000 W
PLW12 0.28125000 W
PLW13 0.17465000 W
F2 - Processing parameters
SI 32768
SF 100.6429309 MHz
WDW EM
SSB 0
LB 1.00 Hz
GB 0
PC 1.40
220
200
180
160
140
120
100
80
60
40
20
0
ppm

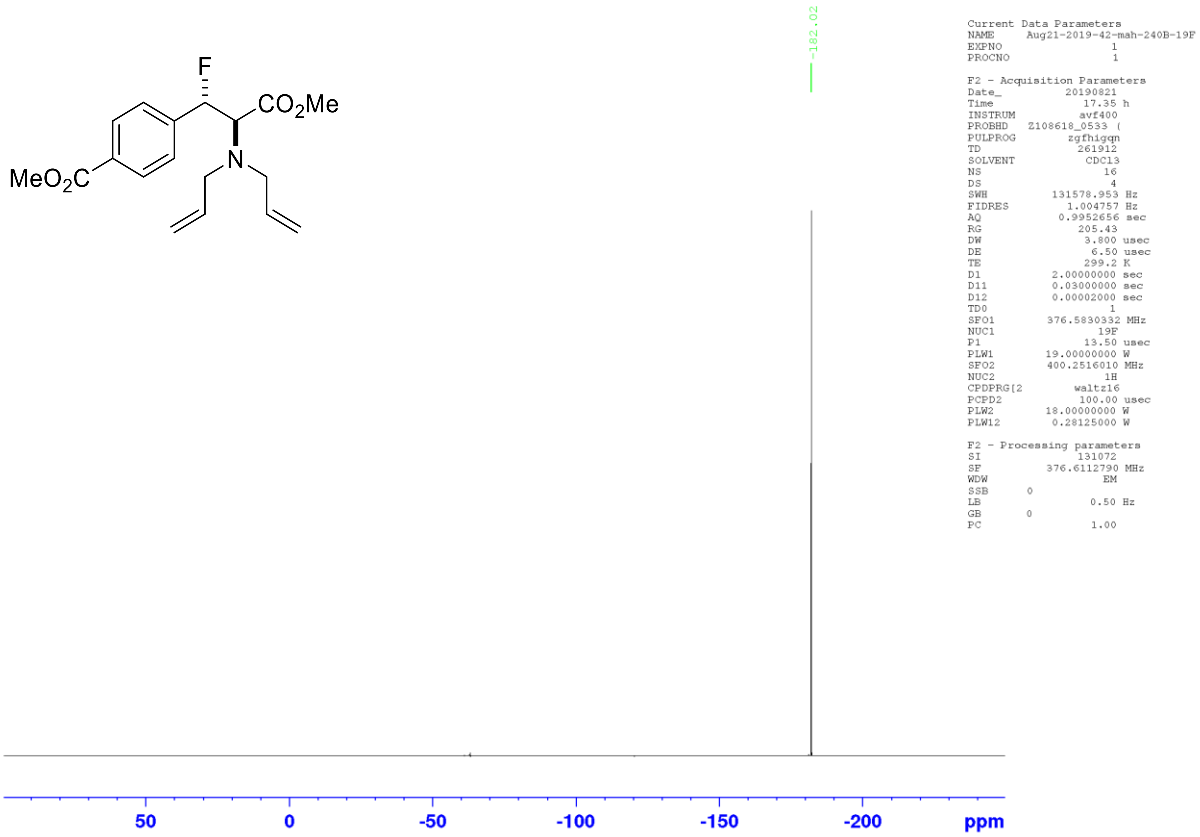
-182.02
F
CO_2Me
MeO_2C
N
Current Data Parameters
NAME Aug21-2019-42-mah-240B-19F
EXPNO 1
PROCNO 1
F2 - Acquisition Parameters
Date_ 20190821
Time 17.35 h
INSTRUM avf400
PROBHD Z108618_0533 (
PULPROG zgfhigqn
TD 261912
SOLVENT CDCl3
NS 16
DS 4
SWH 131578.953 Hz
FIDRES 1.004757 Hz
AQ 0.9952656 sec
RG 205.43
DW 3.800 usec
DE 6.50 usec
TE 299.2 K
D1 2.00000000 sec
D11 0.03000000 sec
D12 0.00002000 sec
TD0 1
SFO1 376.5830332 MHz
NUC1 19F
P1 13.50 usec
PLW1 19.00000000 W
SFO2 400.2516010 MHz
NUC2 1H
CPDPRG[2 waltz16
PCPD2 100.00 usec
PLW2 18.00000000 W
PLW12 0.28125000 W
F2 - Processing parameters
SI 131072
SF 376.6112790 MHz
WDW EM
SSB 0
LB 0.50 Hz
GB 0
PC 1.00
50
0
-50
-100
-150
-200
ppm

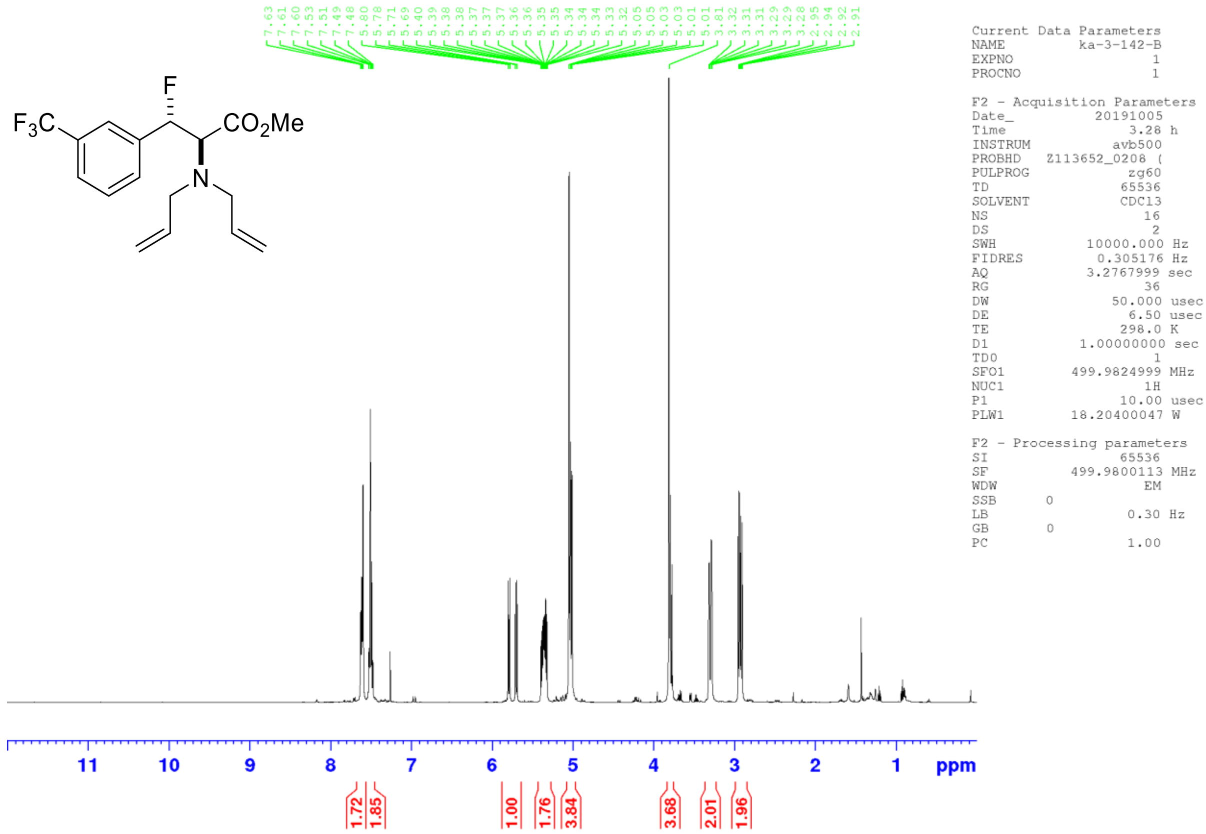

Current Data Parameters
NAME ka-3-142-B
EXPNO 1
PROCNO 1

F2 - Acquisition Parameters
Date_ 20191005
Time 3.28 h
INSTRUM avb500
PROBHD Z113652_0208 (
PULPROG zg60
TD 65536
SOLVENT CDCl3
NS 16
DS 2
SWH 10000.000 Hz
FIDRES 0.305176 Hz
AQ 3.2767999 sec
RG 36
DW 50.000 usec
DE 6.50 usec
TE 298.0 K
D1 1.00000000 sec
TD0 1
SFO1 499.9824999 MHz
NUC1 1H
P1 10.00 usec
PLW1 18.20400047 W

F2 - Processing parameters
SI 65536
SF 499.9800113 MHz
WDW EM
SSB 0
LB 0.30 Hz
GB 0
PC 1.00

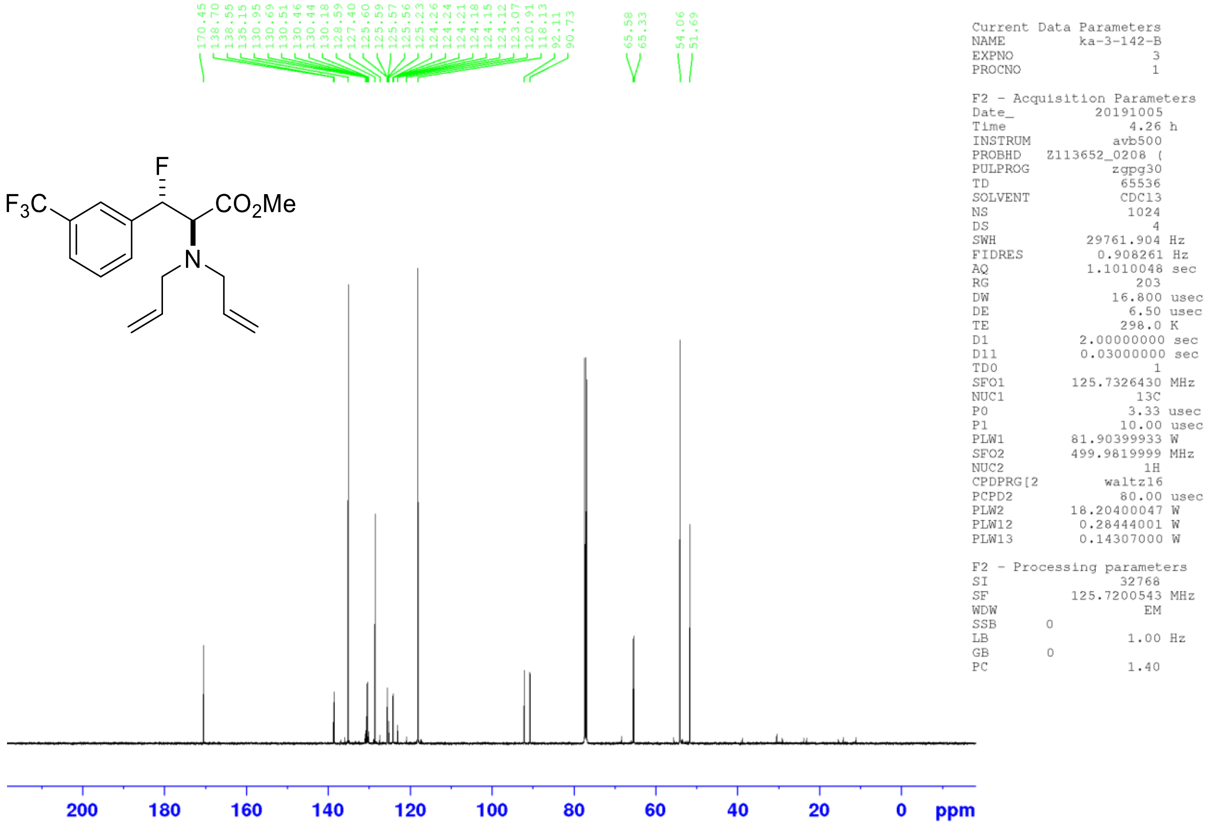

Current Data Parameters
NAME ka-3-142-B
EXPNO 3
PROCNO 1

F2 - Acquisition Parameters
Date_ 20191005
Time 4.26 h
INSTRUM avb500
PROBHD Z113652_0208 (
PULPROG zgpg30
TD 65536
SOLVENT CDCl3
NS 1024
DS 4
SWH 29761.904 Hz
FIDRES 0.908261 Hz
AQ 1.1010048 sec
RG 203
DW 16.800 usec
DE 6.50 usec
TE 298.0 K
D1 2.00000000 sec
D11 0.03000000 sec
TD0 1
SFO1 125.7326430 MHz
NUC1 13C
P0 3.33 usec
P1 10.00 usec
PLW1 81.90399933 W
SFO2 499.9819999 MHz
NUC2 1H
CPDPRG[2 waltz16
PCPD2 80.00 usec
PLW2 18.20400047 W
PLW12 0.28444001 W
PLW13 0.14307000 W

F2 - Processing parameters
SI 32768
SF 125.7200543 MHz
WDW EM
SSB 0
LB 1.00 Hz
GB 0
PC 1.40

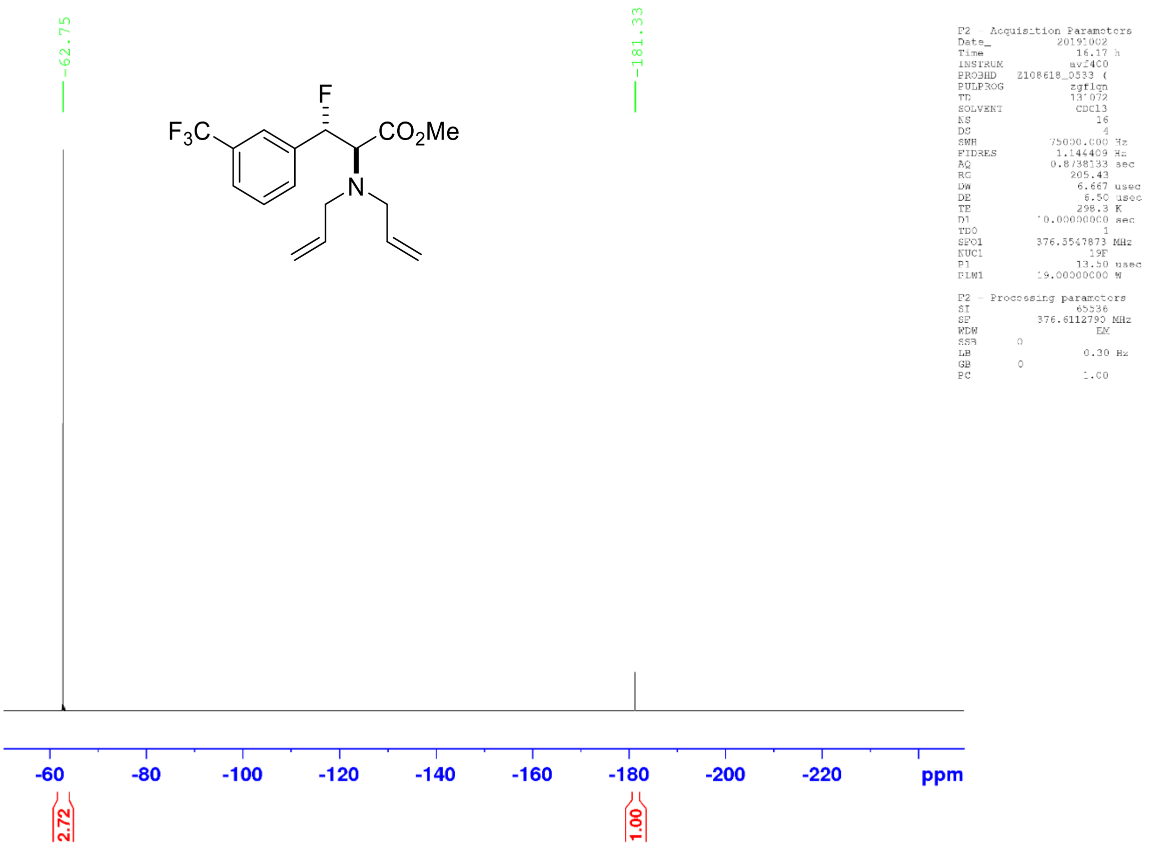
-62.75
-181.33
F
F3C
CO2Me
N
F2 Acquisition Parameters
Date_ 20191002
Time 16.17 h
INSTRUM av400
PROBHD Z108618_0533 (
PULPROG zgflqn
TD 131072
SOLVENT CDCl3
NS 16
DS 4
SWH 75000.000 Hz
FIDRES 1.144409 Hz
AQ 0.8738133 sec
RG 205.43
DW 6.667 usec
DE 6.50 usec
TE 298.3 K
D1 1.00000000 sec
TD0 1
SFO1 376.5547873 MHz
NUC1 19F
P1 13.50 usec
PLW1 19.00000000 W
F2 - Processing parameters
SI 65536
SF 376.6112790 MHz
WDW EM
SSB 0
LB 0.30 Hz
GB 0
PC 1.00
-60 -80 -100 -120 -140 -160 -180 -200 -220 ppm
2.72
1.00

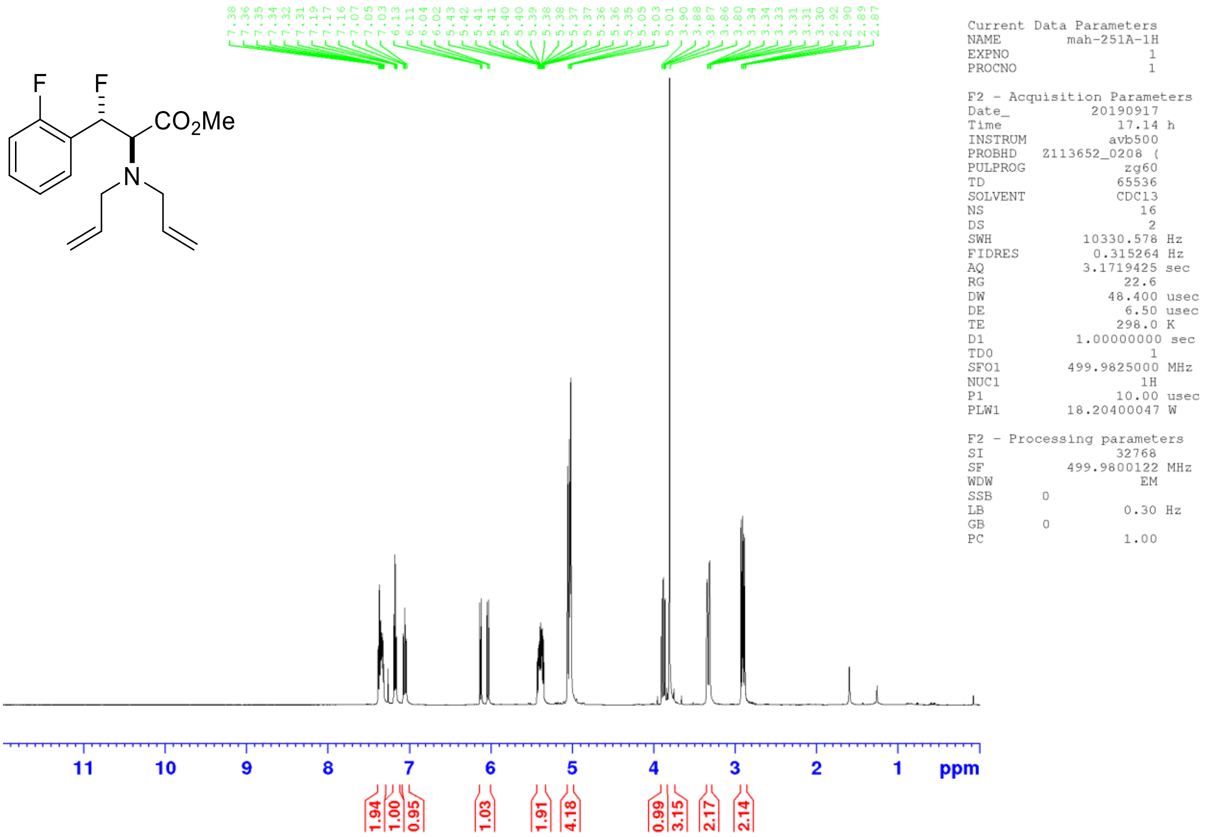
7.38 7.36 7.35 7.34 7.32 7.31 7.19 7.17 7.16 7.07 7.05 7.03 6.13 6.11 6.04 6.02 5.43 5.42 5.41 5.40 5.39 5.38 5.38 5.37 5.36 5.35 5.05 5.03 5.01 3.90 3.88 3.87 3.86 3.80 3.34 3.33 3.31 3.30 2.92 2.90 2.89 2.87
F
F
CO2Me
N
Current Data Parameters
NAME mah-251A-1H
EXPNO 1
PROCNO 1
F2 - Acquisition Parameters
Date_ 20190917
Time 17.14 h
INSTRUM avb500
PROBHD Z113652_0208 (
PULPROG zg60
TD 65536
SOLVENT CDCl3
NS 16
DS 2
SWH 10330.578 Hz
FIDRES 0.315264 Hz
AQ 3.1719425 sec
RG 22.6
DW 48.400 usec
DE 6.50 usec
TE 298.0 K
D1 1.00000000 sec
TD0 1
SFO1 499.9825000 MHz
NUC1 1H
P1 10.00 usec
PLW1 18.20400047 W
F2 - Processing parameters
SI 32768
SF 499.9800122 MHz
WDW EM
SSB 0
LB 0.30 Hz
GB 0
PC 1.00
11 10 9 8 7 6 5 4 3 2 1 ppm
1.94 1.00 0.95 1.03 1.91 4.18 0.99 3.15 2.17 2.14

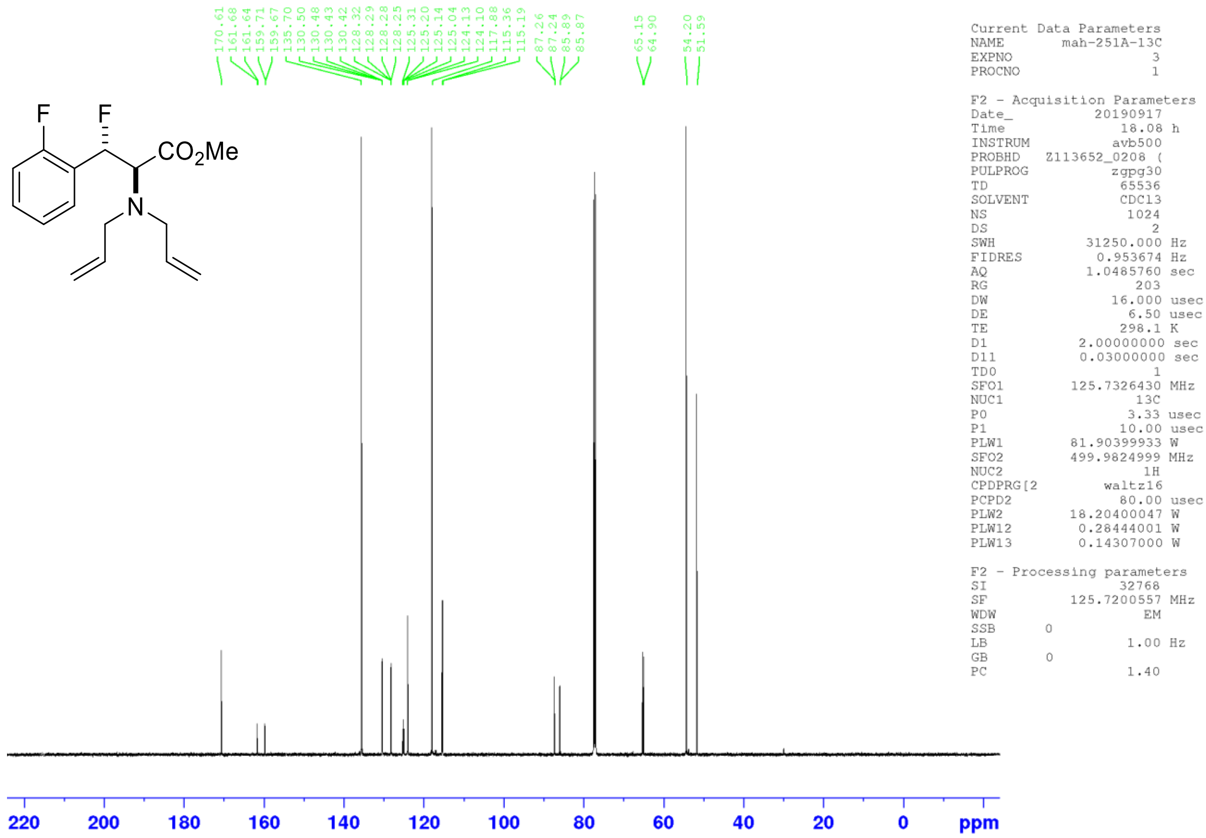

Current Data Parameters
NAME mah-251A-13C
EXPNO 3
PROCNO 1

F2 - Acquisition Parameters
Date_ 20190917
Time 18.08 h
INSTRUM avb500
PROBHD Z113652_0208 (
PULPROG zgpg30
TD 65536
SOLVENT CDCl3
NS 1024
DS 2
SWH 31250.000 Hz
FIDRES 0.953674 Hz
AQ 1.0485760 sec
RG 203
DW 16.000 usec
DE 6.50 usec
TE 298.1 K
D1 2.00000000 sec
D11 0.03000000 sec
TD0 1
SFO1 125.7326430 MHz
NUC1 13C
P0 3.33 usec
P1 10.00 usec
PLW1 81.90399933 W
SFO2 499.9824999 MHz
NUC2 1H
CPDPRG[2 waltz16
PCPD2 80.00 usec
PLW2 18.20400047 W
PLW12 0.28444001 W
PLW13 0.14307000 W

F2 - Processing parameters
SI 32768
SF 125.7200557 MHz
WDW EM
SSB 0
LB 1.00 Hz
GB 0
PC 1.40

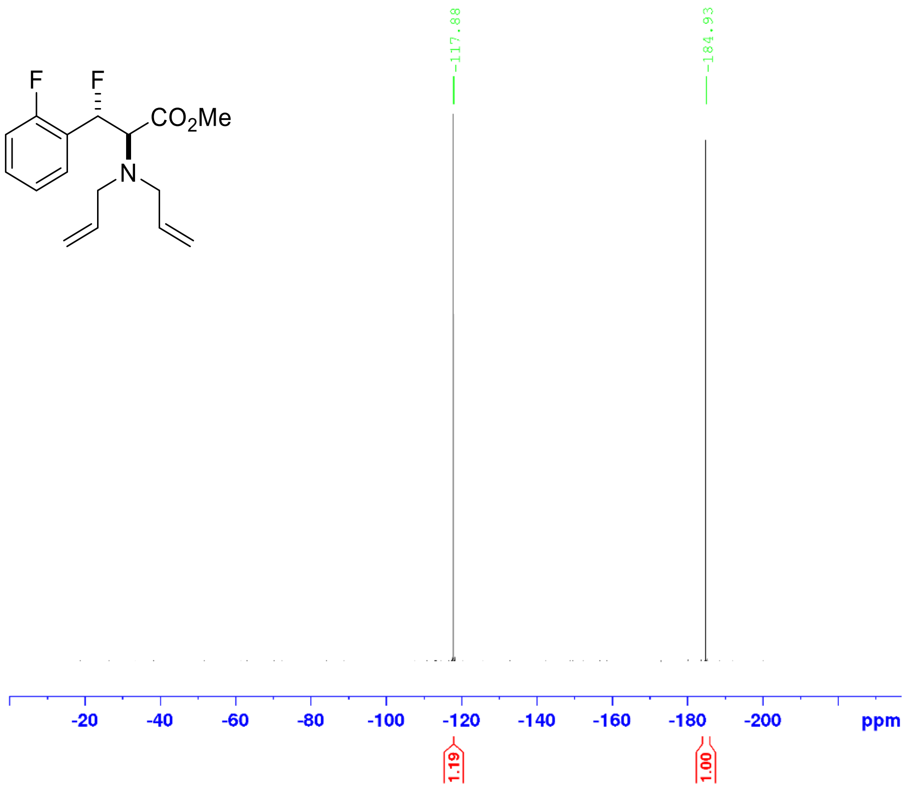

Current Data Parameters
NAME Sep16 2019 46 mah 251A 19F
EXPNO 100001
PROCNO 1

F2 - Acquisition Parameters
Date_ 20190916
Time 9.36 h
INSTRUM avf400
PROBHD Z108618_0533 (
PULPROG zgfhigqn
TD 262144
SOLVENT CDCl3
NS 16
DS 4
SWH 131578.953 Hz
FIDRES 1.003860 Hz
AQ 0.9961472 sec
RG 205.43
DW 3.800 usec
DE 6.50 usec
TE 299.7 K
D1 2.00000000 sec
D11 0.03000000 sec
D12 0.00002000 sec
TD0 1
SFO1 376.5830332 MHz
NUC1 19F
P1 13.50 usec
PLW1 19.00000000 W
SFO2 400.2516010 MHz
NUC2 1H
CPDPRG[2 waltz16
PCPD2 100.00 usec
PLW2 18.00000000 W
PLW12 0.28125000 W

F2 - Processing parameters
SI 131072
SF 376.6112790 MHz
WDW EM
SSB 0
LB 0.50 Hz
GB 0
PC 1.00

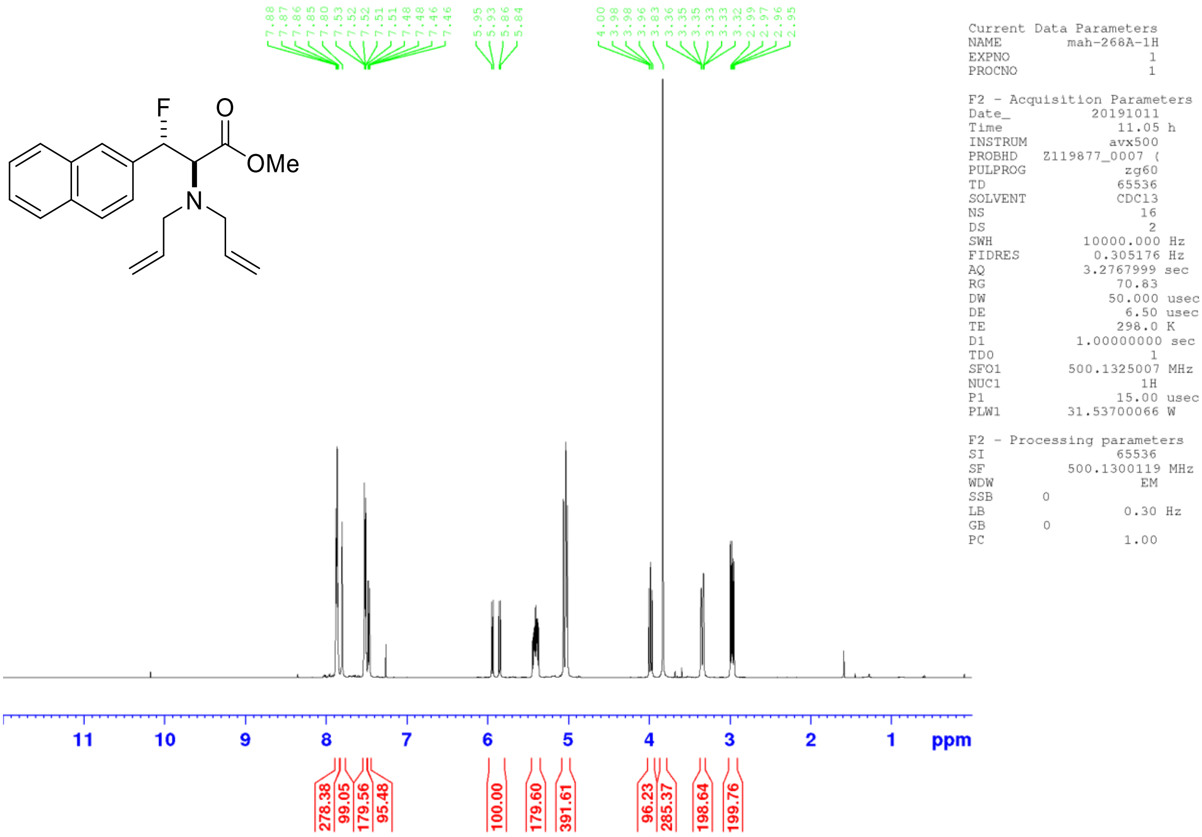
7.88 7.87 7.86 7.85 7.80 7.53 7.52 7.52 7.51 7.51 7.48 7.48 7.46 7.46
5.95 5.93 5.86 5.84
4.00 3.98 3.98 3.96 3.83 3.36 3.35 3.35 3.33 3.33 3.32 2.99 2.97 2.96 2.95
F
O
OMe
N
11 10 9 8 7 6 5 4 3 2 1 ppm
278.38 99.05 179.56 95.48 100.00 179.60 391.61 96.23 285.37 198.64 199.76
Current Data Parameters
NAME mah-268A-1H
EXPNO 1
PROCNO 1
F2 - Acquisition Parameters
Date_ 20191011
Time 11.05 h
INSTRUM avx500
PROBHD Z119877_0007 (
PULPROG zg60
TD 65536
SOLVENT CDCl3
NS 16
DS 2
SWH 10000.000 Hz
FIDRES 0.305176 Hz
AQ 3.2767999 sec
RG 70.83
DW 50.000 usec
DE 6.50 usec
TE 298.0 K
D1 1.00000000 sec
TD0 1
SFO1 500.1325007 MHz
NUC1 1H
P1 15.00 usec
PLW1 31.53700066 W
F2 - Processing parameters
SI 65536
SF 500.1300119 MHz
WDW EM
SSB 0
LB 0.30 Hz
GB 0
PC 1.00

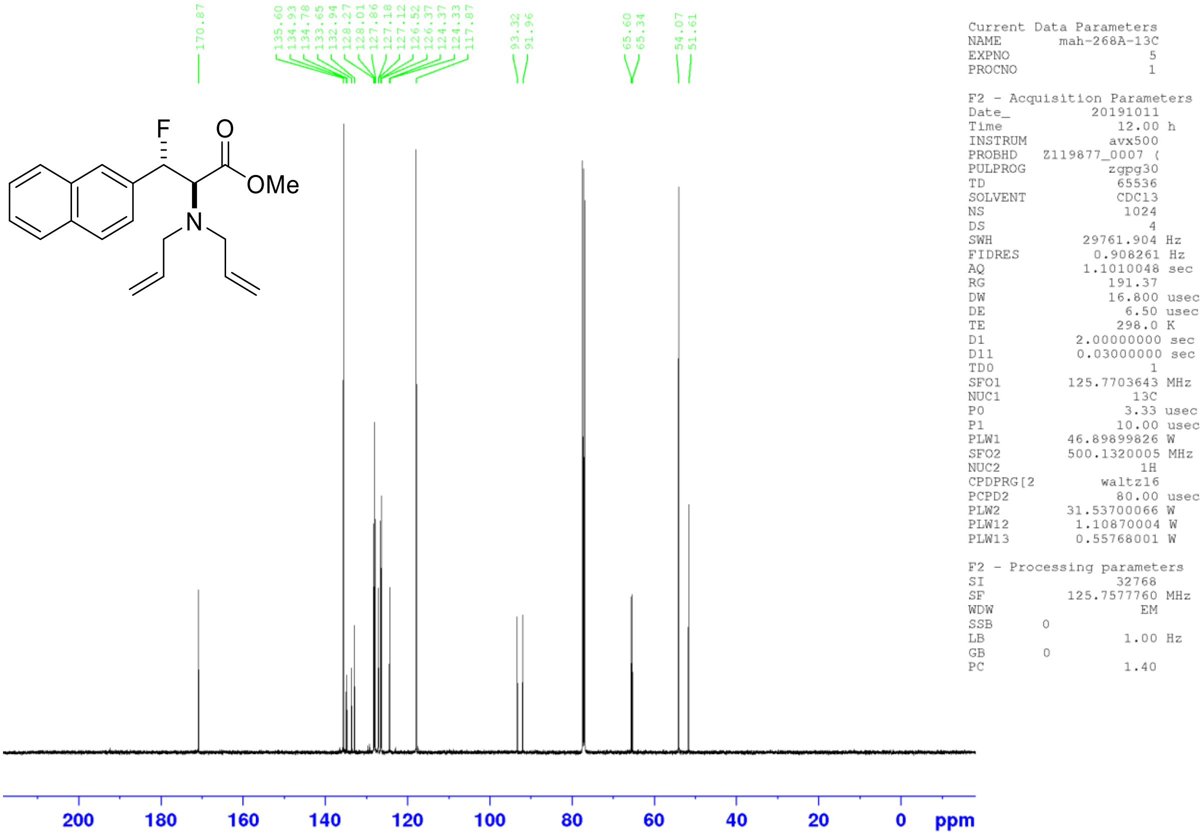
170.87
135.60 134.93 134.78 133.65 132.94 128.27 128.01 127.86 127.18 127.12 126.52 126.37 124.37 124.33 117.87
93.32 91.96
65.60 65.34
54.07 51.61
F
O
OMe
N
200 180 160 140 120 100 80 60 40 20 0 ppm
Current Data Parameters
NAME mah-268A-13C
EXPNO 5
PROCNO 1
F2 - Acquisition Parameters
Date_ 20191011
Time 12.00 h
INSTRUM avx500
PROBHD Z119877_0007 (
PULPROG zgpg30
TD 65536
SOLVENT CDCl3
NS 1024
DS 4
SWH 29761.904 Hz
FIDRES 0.908261 Hz
AQ 1.1010048 sec
RG 191.37
DW 16.800 usec
DE 6.50 usec
TE 298.0 K
D1 2.00000000 sec
D11 0.03000000 sec
TD0 1
SFO1 125.7703643 MHz
NUC1 13C
P0 3.33 usec
P1 10.00 usec
PLW1 46.89899826 W
SFO2 500.1320005 MHz
NUC2 1H
CPDPRG[2 waltz16
PCPD2 80.00 usec
PLW2 31.53700066 W
PLW12 1.10870004 W
PLW13 0.55768001 W
F2 - Processing parameters
SI 32768
SF 125.7577760 MHz
WDW EM
SSB 0
LB 1.00 Hz
GB 0
PC 1.40

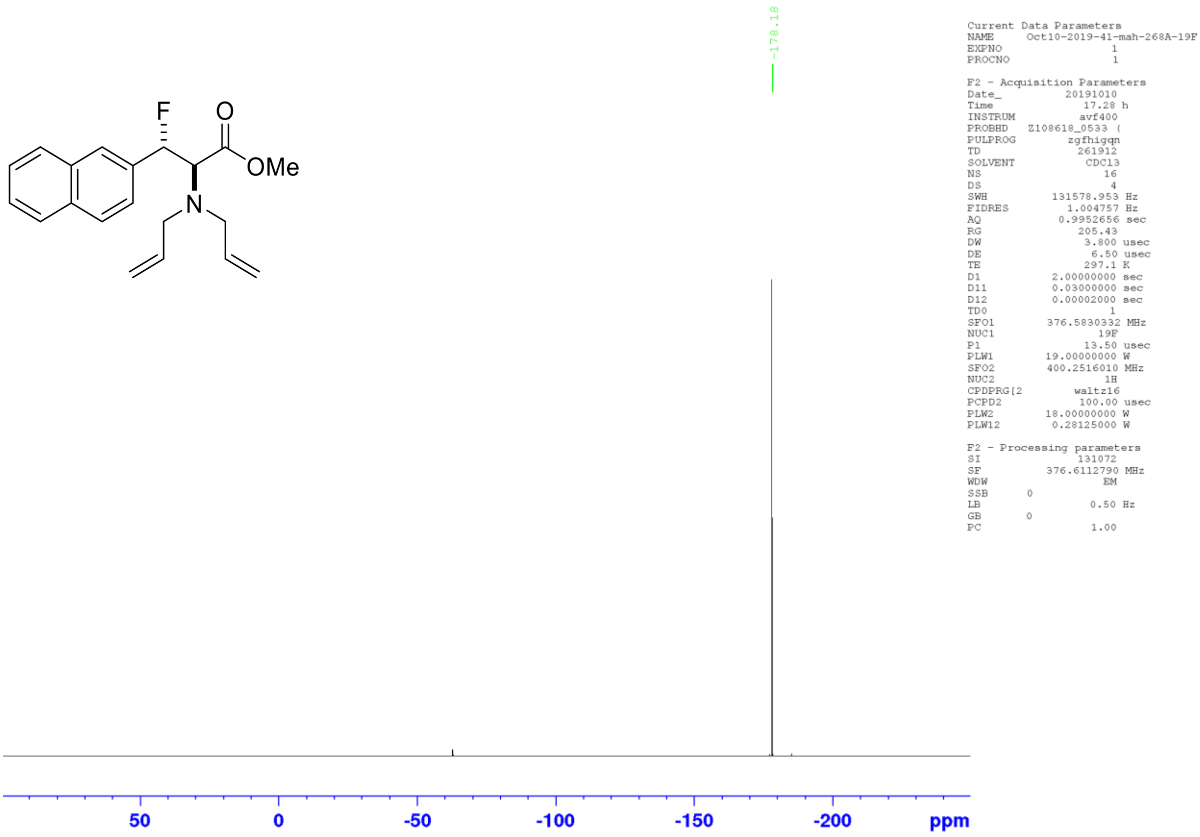

F
O
OMe
N
-178.18
50
0
-50
-100
-150
-200
ppm
Current Data Parameters
NAME Oct10-2019-41-mah-268A-19F
EXPNO 1
PROCNO 1
F2 - Acquisition Parameters
Date_ 20191010
Time 17.28 h
INSTRUM avf400
PROBHD Z108618_0533 (
PULPROG zgfhigqn
TD 261912
SOLVENT CDCl3
NS 16
DS 4
SWH 131578.953 Hz
FIDRES 1.004757 Hz
AQ 0.9952656 sec
RG 205.43
DW 3.800 usec
DE 6.50 usec
TE 297.1 K
D1 2.00000000 sec
D11 0.03000000 sec
D12 0.00002000 sec
TD0 1
SFO1 376.5830332 MHz
NUC1 19F
P1 13.50 usec
PLW1 19.00000000 W
SFO2 400.2516010 MHz
NUC2 1H
CPDPRG[2 waltz16
PCPD2 100.00 usec
PLW2 18.00000000 W
PLW12 0.28125000 W
F2 - Processing parameters
SI 131072
SF 376.6112790 MHz
WDW EM
SSB 0
LB 0.50 Hz
GB 0
PC 1.00

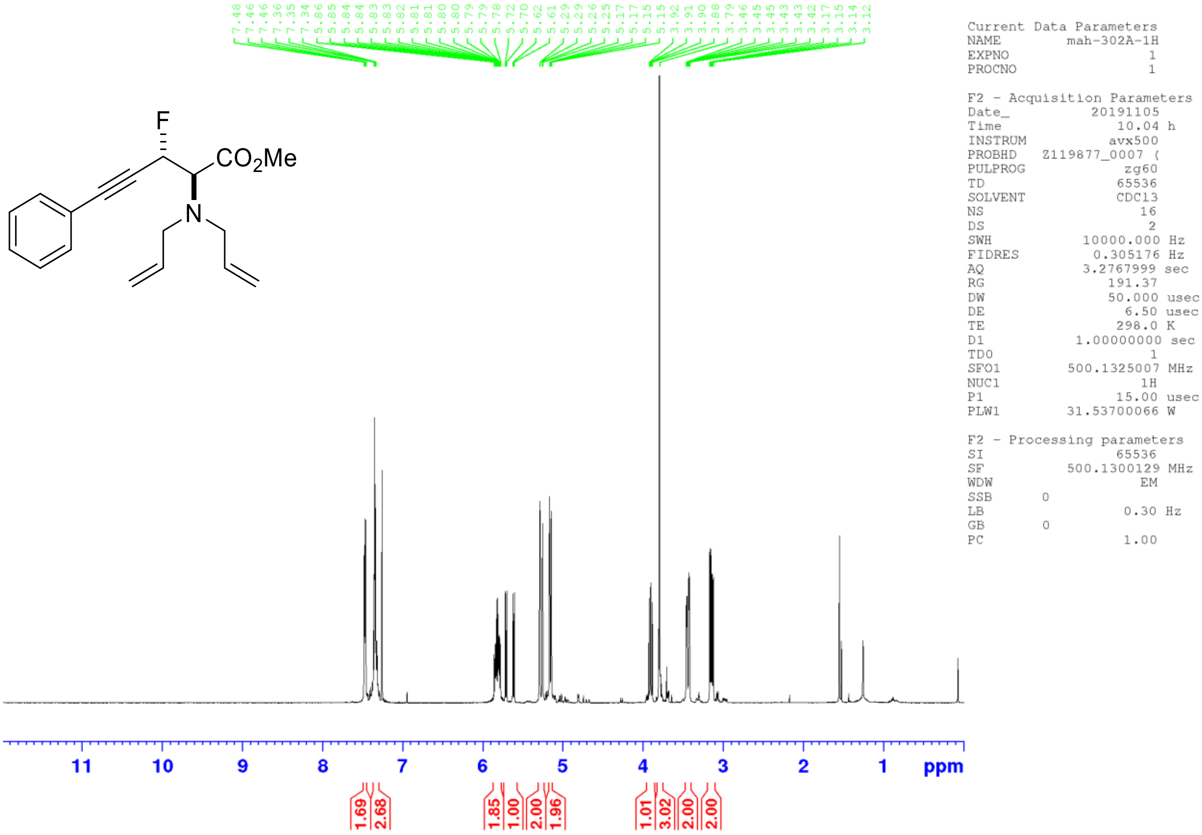

F
CO_2Me
N
7.48 7.46 7.46 7.36 7.35 7.34 5.86 5.85 5.84 5.84 5.83 5.83 5.82 5.81 5.81 5.80 5.80 5.79 5.79 5.78 5.72 5.70 5.62 5.61 5.29 5.29 5.26 5.25 5.17 5.17 5.15 5.15 3.92 3.91 3.90 3.88 3.79 3.46 3.45 3.45 3.43 3.43 3.42 3.17 3.15 3.14 3.12
11
10
9
8
7
6
5
4
3
2
1
ppm
1.69 2.68 1.85 1.00 2.00 1.96 1.01 3.02 2.00 2.00
Current Data Parameters
NAME mah-302A-1H
EXPNO 1
PROCNO 1
F2 - Acquisition Parameters
Date_ 20191105
Time 10.04 h
INSTRUM avx500
PROBHD Z119877_0007 (
PULPROG zg60
TD 65536
SOLVENT CDCl3
NS 16
DS 2
SWH 10000.000 Hz
FIDRES 0.305176 Hz
AQ 3.2767999 sec
RG 191.37
DW 50.000 usec
DE 6.50 usec
TE 298.0 K
D1 1.00000000 sec
TD0 1
SFO1 500.1325007 MHz
NUC1 1H
P1 15.00 usec
PLW1 31.53700066 W
F2 - Processing parameters
SI 65536
SF 500.1300129 MHz
WDW EM
SSB 0
LB 0.30 Hz
GB 0
PC 1.00

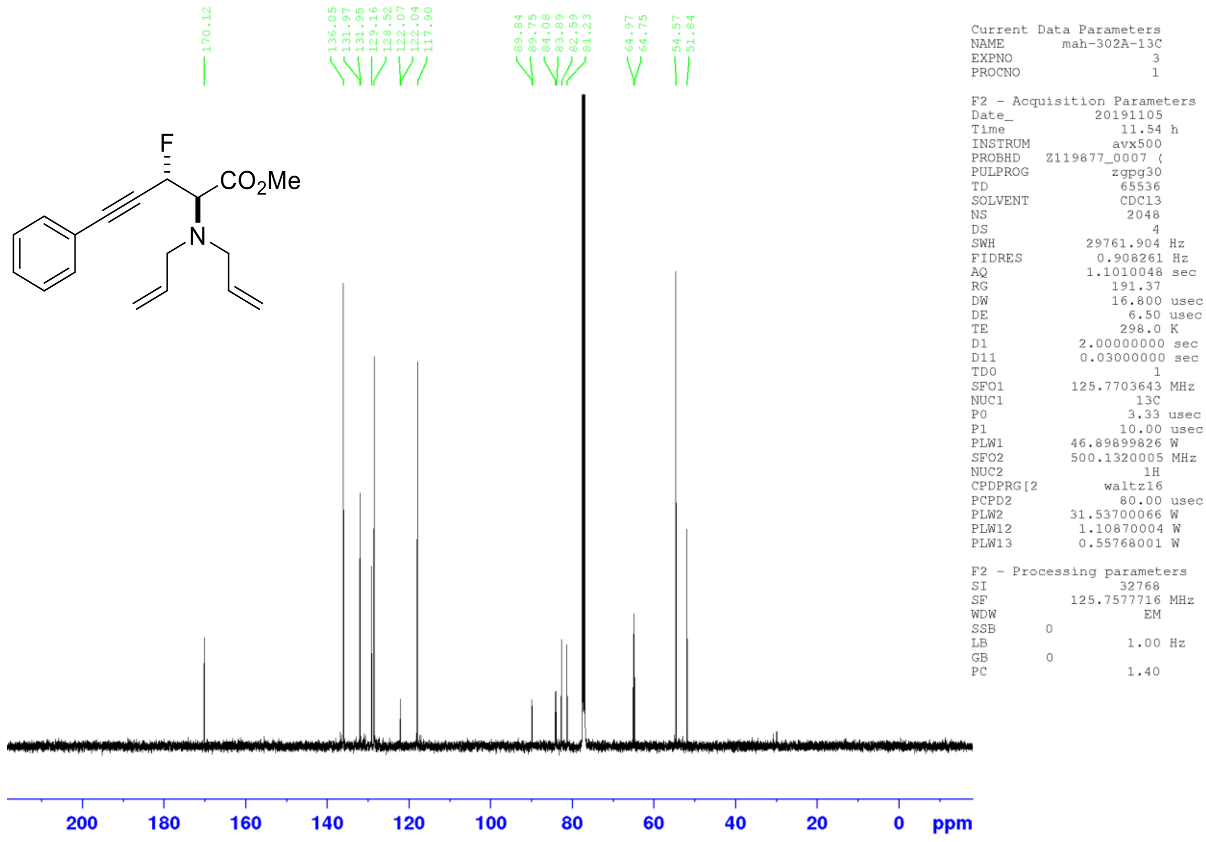

Current Data Parameters
NAME mah-302A-13C
EXPNO 3
PROCNO 1

F2 - Acquisition Parameters
Date_ 20191105
Time 11.54 h
INSTRUM avx500
PROBHD Z119877_0007 (
PULPROG zgpg30
TD 65536
SOLVENT CDCl3
NS 2048
DS 4
SWH 29761.904 Hz
FIDRES 0.908261 Hz
AQ 1.1010048 sec
RG 191.37
DW 16.800 usec
DE 6.50 usec
TE 298.0 K
D1 2.00000000 sec
D11 0.03000000 sec
TD0 1
SFO1 125.7703643 MHz
NUC1 13C
P0 3.33 usec
P1 10.00 usec
PLW1 46.89899826 W
SFO2 500.1320005 MHz
NUC2 1H
CPDPRG[2 waltz16
PCPD2 80.00 usec
PLW2 31.53700066 W
PLW12 1.10870004 W
PLW13 0.55768001 W

F2 - Processing parameters
SI 32768
SF 125.7577716 MHz
WDW EM
SSB 0
LB 1.00 Hz
GB 0
PC 1.40

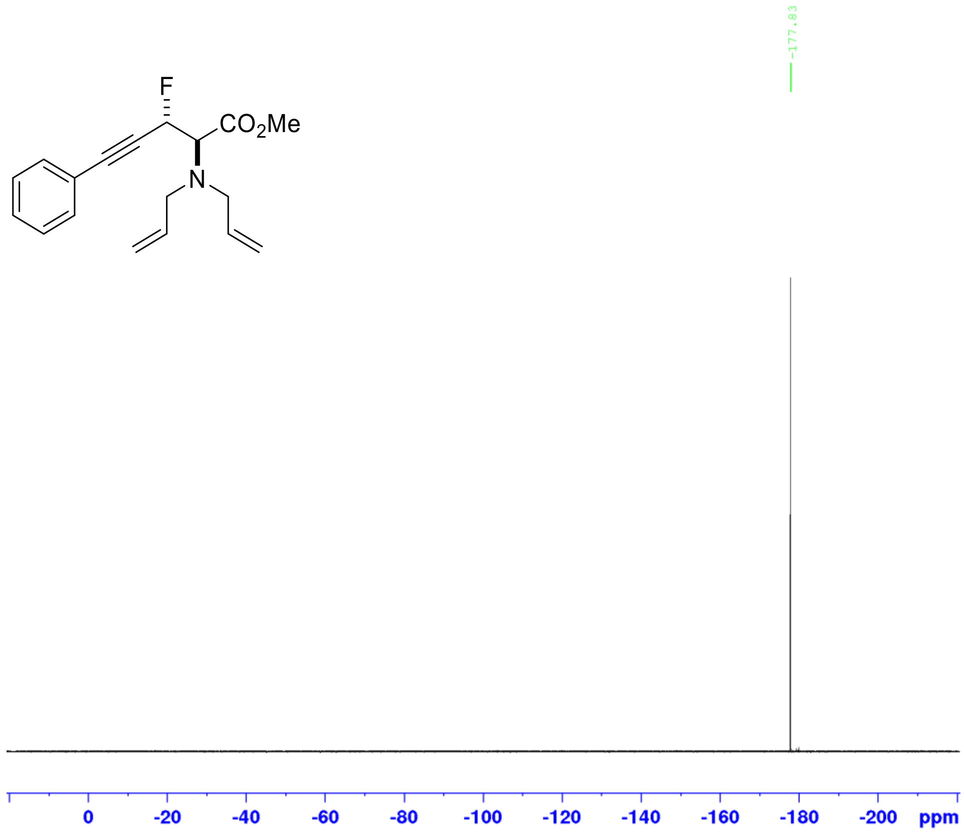

Current Data Parameters
NAME mah-302A-19F
EXPNO 5
PROCNO 1

F2 - Acquisition Parameters
Date_ 20191105
Time 11.55 h
INSTRUM avx500
PROBHD Z119877_0007 (
PULPROG zgig30
TD 130840
SOLVENT CDCl3
NS 16
DS 4
SWH 113636.367 Hz
FIDRES 1.737028 Hz
AQ 0.5756960 sec
RG 50.85
DW 4.400 usec
DE 6.50 usec
TE 298.0 K
D1 1.00000000 sec
D11 0.03000000 sec
TD0 1
SFO1 470.5453178 MHz
NUC1 19F
P0 7.33 usec
P1 22.00 usec
PLW1 34.09799957 W
SFO2 500.1320005 MHz
NUC2 1H
CPDPRG[2 waltz16
PCPD2 80.00 usec
PLW2 31.53700066 W
PLW12 1.10870004 W

F2 - Processing parameters
SI 65536
SF 470.5923772 MHz
WDW EM
SSB 0
LB 0.30 Hz
GB 0
PC 1.00

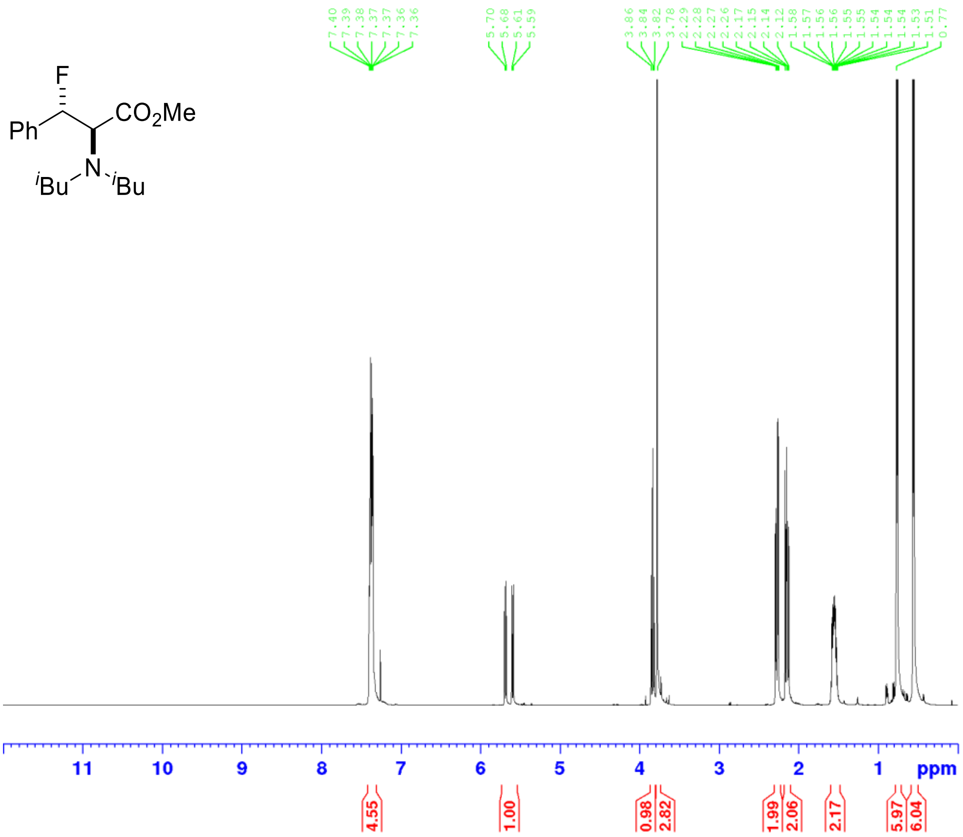

Current Data Parameters
NAME mah-245B-1H
EXPNO 1
PROCNO 1

F2 - Acquisition Parameters
Date_ 20190911
Time 14.06 h
INSTRUM avb500
PROBHD Z113652_0208 (
PULPROG zg60
TD 65536
SOLVENT CDCl3
NS 16
DS 2
SWH 10330.578 Hz
FIDRES 0.315264 Hz
AQ 3.1719425 sec
RG 22.6
DW 48.400 usec
DE 6.50 usec
TE 298.0 K
D1 1.00000000 sec
TD0 1
SFO1 499.9825000 MHz
NUC1 1H
P1 10.00 usec
PLW1 18.20400047 W

F2 - Processing parameters
SI 32768
SF 499.9800120 MHz
WDW EM
SSB 0
LB 0.30 Hz
GB 0
PC 1.00

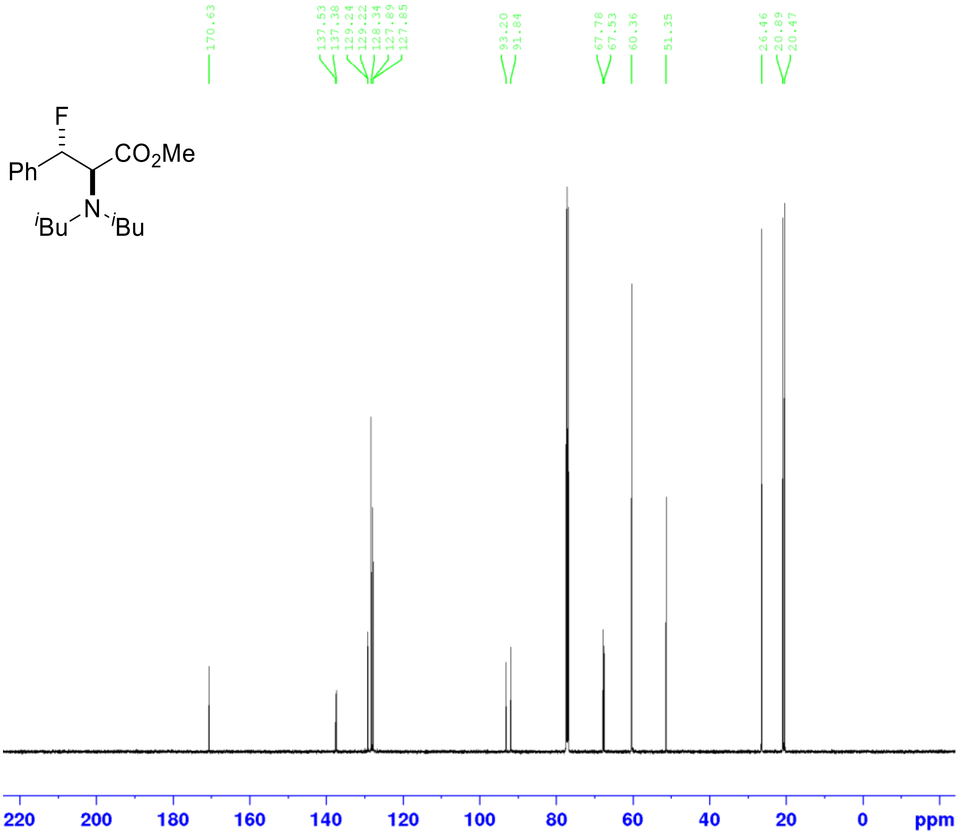

Current Data Parameters
NAME mah-245B-13C
EXPNO 5
PROCNO 1

F2 - Acquisition Parameters
Date_ 20190911
Time 15.03 h
INSTRUM avb500
PROBHD Z113652_0208 (
PULPROG zgpg30
TD 65536
SOLVENT CDCl3
NS 1024
DS 2
SWH 31250.000 Hz
FIDRES 0.953674 Hz
AQ 1.0485760 sec
RG 203
DW 16.000 usec
DE 6.50 usec
TE 298.0 K
D1 2.00000000 sec
D11 0.03000000 sec
TD0 1
SFO1 125.7326430 MHz
NUC1 13C
P0 3.33 usec
P1 10.00 usec
PLW1 81.90399933 W
SFO2 499.9824999 MHz
NUC2 1H
CPDPRG[2 waltz16
PCPD2 80.00 usec
PLW2 18.20400047 W
PLW12 0.28444001 W
PLW13 0.14307000 W

F2 - Processing parameters
SI 32768
SF 125.7200549 MHz
WDW EM
SSB 0
LB 1.00 Hz
GB 0
PC 1.40

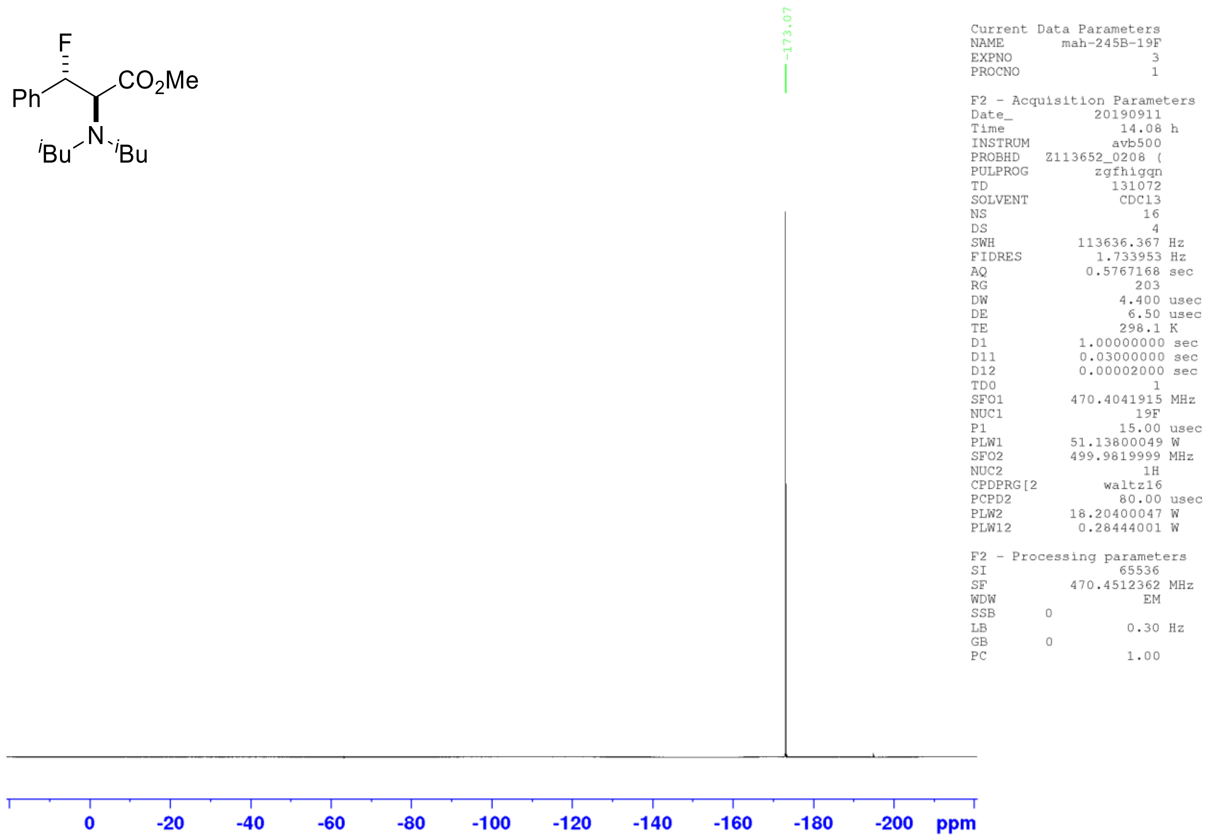

F
Ph
CO2Me
N
iBu
iBu
-173.07
Current Data Parameters
NAME mah-245B-19F
EXPNO 3
PROCNO 1
F2 - Acquisition Parameters
Date_ 20190911
Time 14.08 h
INSTRUM avb500
PROBHD Z113652_0208 (
PULPROG zgfhigqn
TD 131072
SOLVENT CDCl3
NS 16
DS 4
SWH 113636.367 Hz
FIDRES 1.733953 Hz
AQ 0.5767168 sec
RG 203
DW 4.400 usec
DE 6.50 usec
TE 298.1 K
D1 1.00000000 sec
D11 0.03000000 sec
D12 0.00002000 sec
TD0 1
SFO1 470.4041915 MHz
NUC1 19F
P1 15.00 usec
PLW1 51.13800049 W
SFO2 499.9819999 MHz
NUC2 1H
CPDPRG[2 waltz16
PCPD2 80.00 usec
PLW2 18.20400047 W
PLW12 0.28444001 W
F2 - Processing parameters
SI 65536
SF 470.4512362 MHz
WDW EM
SSB 0
LB 0.30 Hz
GB 0
PC 1.00
0
-20
-40
-60
-80
-100
-120
-140
-160
-180
-200
ppm

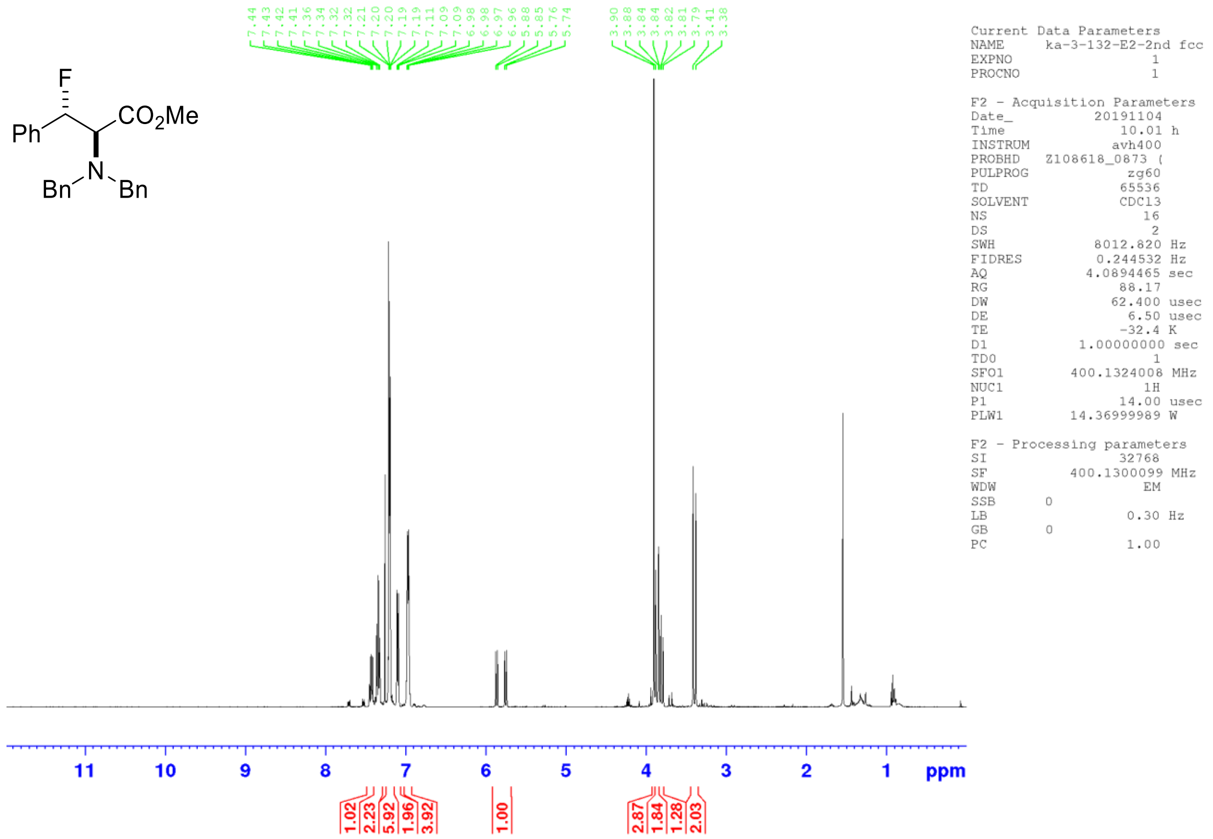

F
Ph
CO2Me
N
Bn
Bn
7.44 7.43 7.42 7.41 7.36 7.34 7.32 7.21 7.20 7.20 7.19 7.19 7.11 7.09 6.98 6.97 6.96 5.88 5.85 5.76 5.74
3.90 3.88 3.84 3.84 3.82 3.81 3.79 3.41 3.38
Current Data Parameters
NAME ka-3-132-E2-2nd fcc
EXPNO 1
PROCNO 1
F2 - Acquisition Parameters
Date_ 20191104
Time 10.01 h
INSTRUM avh400
PROBHD Z108618_0873 (
PULPROG zg60
TD 65536
SOLVENT CDCl3
NS 16
DS 2
SWH 8012.820 Hz
FIDRES 0.244532 Hz
AQ 4.0894465 sec
RG 88.17
DW 62.400 usec
DE 6.50 usec
TE -32.4 K
D1 1.00000000 sec
TD0 1
SFO1 400.1324008 MHz
NUC1 1H
P1 14.00 usec
PLW1 14.36999989 W
F2 - Processing parameters
SI 32768
SF 400.1300099 MHz
WDW EM
SSB 0
LB 0.30 Hz
GB 0
PC 1.00
11
10
9
8
7
6
5
4
3
2
1
ppm
1.02 2.23 5.92 1.96 3.92
1.00
2.87 1.84 1.28 2.03

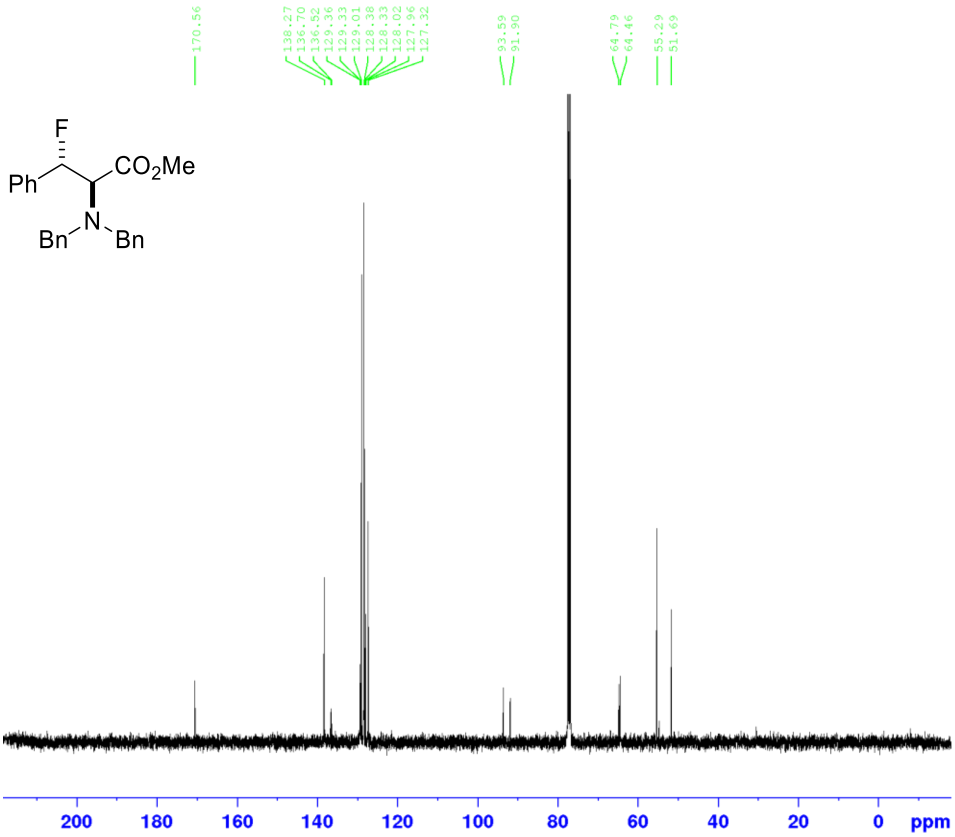

Current Data Parameters
NAME ka-3-132-E2-2nd fcc
EXPNO 2
PROCNO 1

F2 - Acquisition Parameters
Date_ 20191102
Time 22.10 h
INSTRUM Avance
PROBHD Z116098_0219 (
PULPROG zgpg30
TD 65536
SOLVENT CDCl3
NS 1024
DS 4
SWH 23809.523 Hz
FIDRES 0.726609 Hz
AQ 1.3762560 sec
RG 15.625
DW 21.000 usec
DE 6.50 usec
TE 298.0 K
D1 2.00000000 sec
D11 0.03000000 sec
TD0 1
SFO1 100.6228298 MHz
NUC1 13C
P0 3.33 usec
P1 10.00 usec
PLW1 73.73300171 W
SFO2 400.1316005 MHz
NUC2 1H
CPDPRG[2 waltz16
PCPD2 90.00 usec
PLW2 16.35899925 W
PLW12 0.20196000 W
PLW13 0.10159000 W

F2 - Processing parameters
SI 32768
SF 100.6127553 MHz
WDW EM
SSB 0
LB 1.00 Hz
GB 0
PC 1.40

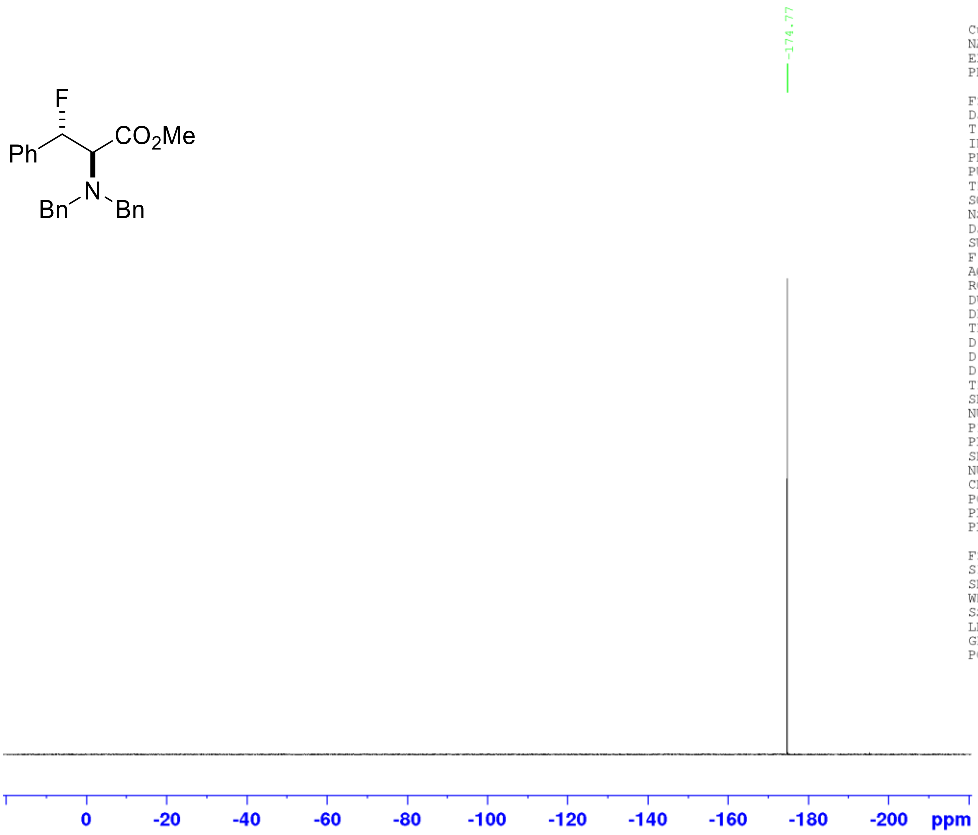

Current Data Parameters
NAME ka-3-132-E2-2nd fcc
EXPNO 19
PROCNO 1

F2 - Acquisition Parameters
Date_ 20191104
Time 21.47 h
INSTRUM avb500
PROBHD Z113652_0208 (
PULPROG zgfhigqn.2
TD 131072
SOLVENT CDCl3
NS 16
DS 4
SWH 113636.367 Hz
FIDRES 1.733953 Hz
AQ 0.5767168 sec
RG 203
DW 4.400 usec
DE 6.50 usec
TE 298.1 K
D1 1.00000000 sec
D11 0.03000000 sec
D12 0.00002000 sec
TD0 1
SFO1 470.4041911 MHz
NUC1 19F
P1 15.00 usec
PLW1 51.13800049 W
SFO2 499.9819999 MHz
NUC2 1H
CPDPRG[2 waltz16
PCPD2 80.00 usec
PLW2 18.20400047 W
PLW12 0.28444001 W

F2 - Processing parameters
SI 65536
SF 470.4512362 MHz
WDW EM
SSB 0
LB 0.30 Hz
GB 0
PC 1.00

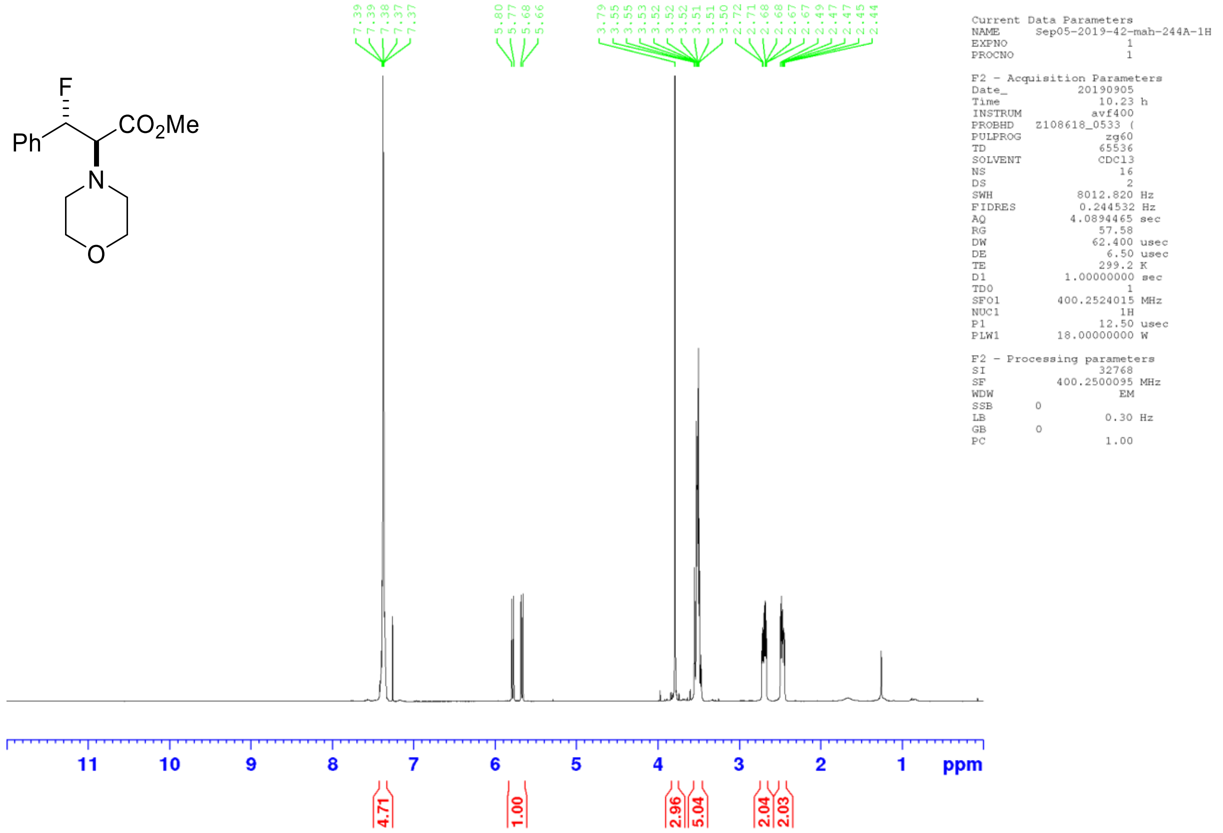
F
Ph
CO2Me
N
O
7.39 7.39 7.38 7.37 7.37
5.80 5.77 5.68 5.66
3.79 3.55 3.55 3.53 3.52 3.52 3.52 3.51 3.51 3.50 2.72 2.71 2.68 2.68 2.67 2.49 2.47 2.45 2.44
11 10 9 8 7 6 5 4 3 2 1 ppm
4.71 1.00 2.96 5.04 2.04 2.03
Current Data Parameters
NAME Sep05-2019-42-mah-244A-1H
EXPNO 1
PROCNO 1
F2 - Acquisition Parameters
Date_ 20190905
Time 10.23 h
INSTRUM avf400
PROBHD Z108618_0533 (
PULPROG zg60
TD 65536
SOLVENT CDCl3
NS 16
DS 2
SWH 8012.820 Hz
FIDRES 0.244532 Hz
AQ 4.0894465 sec
RG 57.58
DW 62.400 usec
DE 6.50 usec
TE 299.2 K
D1 1.00000000 sec
TD0 1
SFO1 400.2524015 MHz
NUC1 1H
P1 12.50 usec
PLW1 18.00000000 W
F2 - Processing parameters
SI 32768
SF 400.2500095 MHz
WDW EM
SSB 0
LB 0.30 Hz
GB 0
PC 1.00

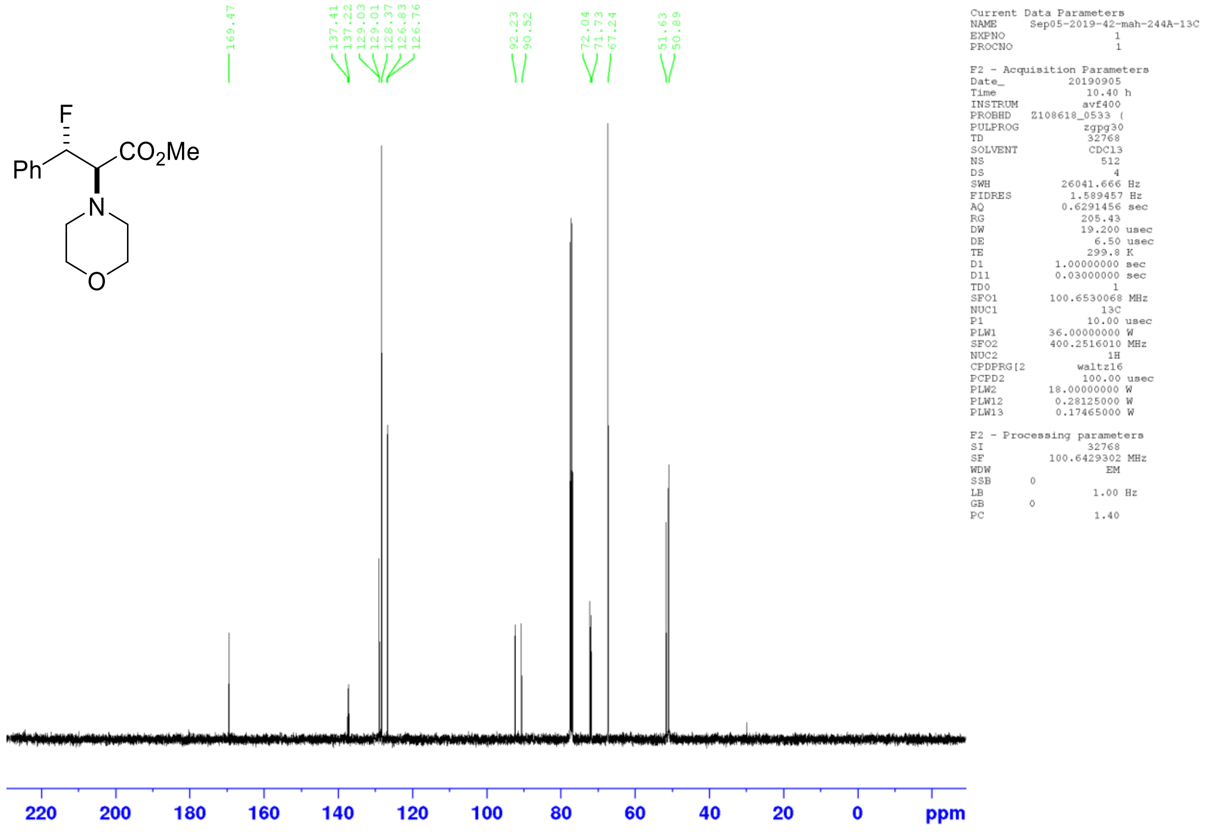
F
Ph
CO2Me
N
O
169.47
137.41 137.22 129.03 129.01 128.37 126.83 126.76
92.23 90.52
72.04 71.73 67.24
51.63 50.89
220 200 180 160 140 120 100 80 60 40 20 0 ppm
Current Data Parameters
NAME Sep05-2019-42-mah-244A-13C
EXPNO 1
PROCNO 1
F2 - Acquisition Parameters
Date_ 20190905
Time 10.40 h
INSTRUM avf400
PROBHD Z108618_0533 (
PULPROG zgpg30
TD 32768
SOLVENT CDCl3
NS 512
DS 4
SWH 26041.666 Hz
FIDRES 1.589457 Hz
AQ 0.6291456 sec
RG 205.43
DW 19.200 usec
DE 6.50 usec
TE 299.8 K
D1 1.00000000 sec
D11 0.03000000 sec
TD0 1
SFO1 100.6530068 MHz
NUC1 13C
P1 10.00 usec
PLW1 36.00000000 W
SFO2 400.2516010 MHz
NUC2 1H
CPDPRG[2 waltz16
PCPD2 100.00 usec
PLW2 18.00000000 W
PLW12 0.28125000 W
PLW13 0.17465000 W
F2 - Processing parameters
SI 32768
SF 100.6429302 MHz
WDW EM
SSB 0
LB 1.00 Hz
GB 0
PC 1.40

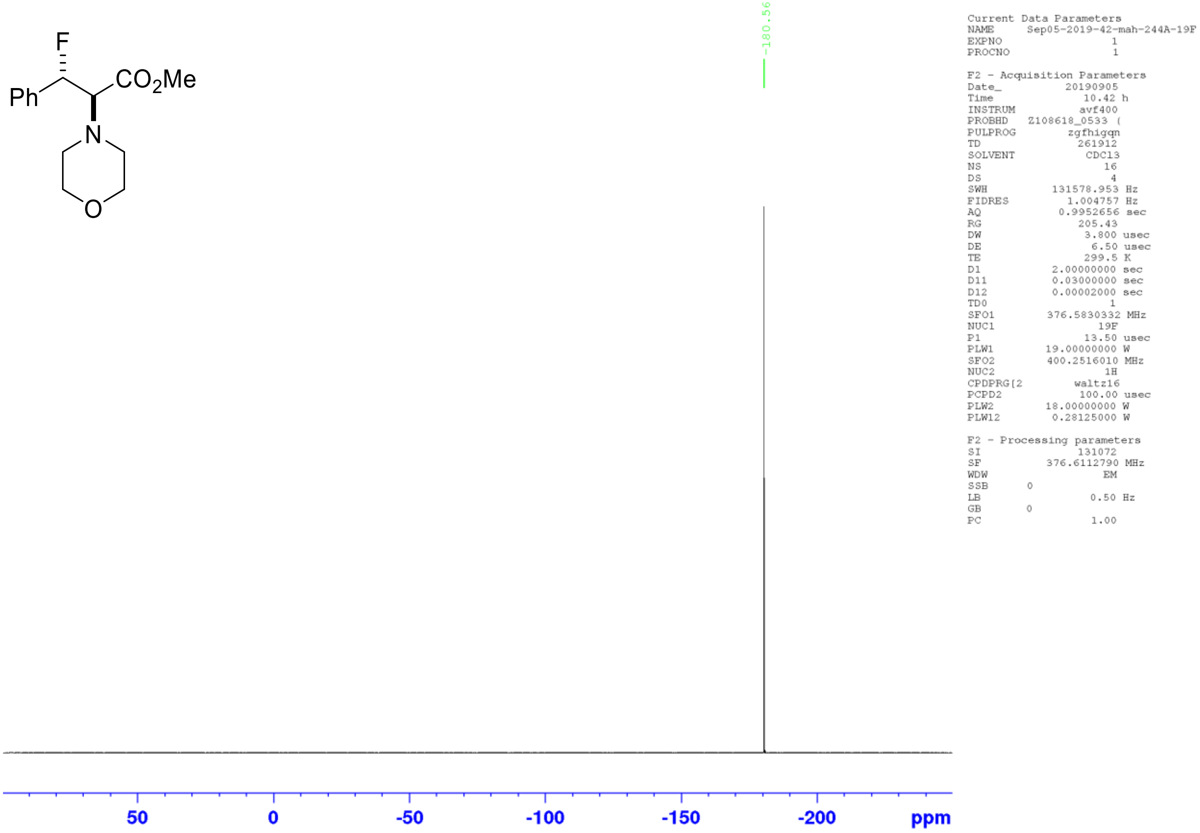
F
Ph
CO2Me
N
O
-180.56
Current Data Parameters
NAME Sep05-2019-42-mah-244A-19F
EXPNO 1
PROCNO 1
F2 - Acquisition Parameters
Date_ 20190905
Time 10.42 h
INSTRUM avf400
PROBHD Z108618_0533 (
PULPROG zgfhigqn
TD 261912
SOLVENT CDCl3
NS 16
DS 4
SWH 131578.953 Hz
FIDRES 1.004757 Hz
AQ 0.9952656 sec
RG 205.43
DW 3.800 usec
DE 6.50 usec
TE 299.5 K
D1 2.00000000 sec
D11 0.03000000 sec
D12 0.00002000 sec
TD0 1
SFO1 376.5830332 MHz
NUC1 19F
P1 13.50 usec
PLW1 19.00000000 W
SFO2 400.2516010 MHz
NUC2 1H
CPDPRG[2 waltz16
PCPD2 100.00 usec
PLW2 18.00000000 W
PLW12 0.28125000 W
F2 - Processing parameters
SI 131072
SF 376.6112790 MHz
WDW EM
SSB 0
LB 0.50 Hz
GB 0
PC 1.00
50
0
-50
-100
-150
-200
ppm

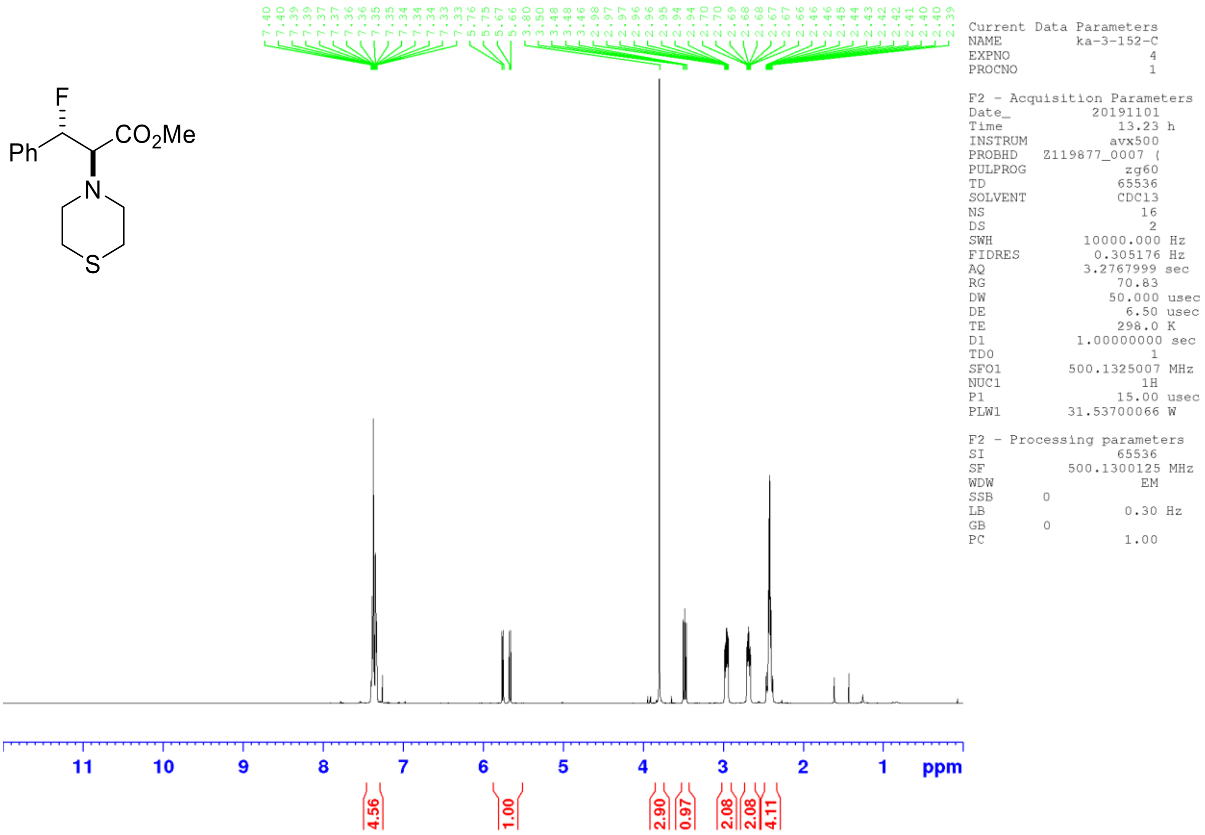
F
Ph
CO2Me
N
S
7.40 7.40 7.39 7.39 7.37 7.37 7.36 7.35 7.35 7.34 7.34 7.34 7.33 7.33 5.76 5.75 5.67 5.66 3.80 3.50 3.48 3.48 3.46 2.98 2.97 2.97 2.96 2.96 2.95 2.94 2.94 2.70 2.69 2.68 2.67 2.67 2.66 2.46 2.46 2.45 2.44 2.43 2.42 2.41 2.40 2.40 2.39
Current Data Parameters
NAME ka-3-152-C
EXPNO 4
PROCNO 1
F2 - Acquisition Parameters
Date_ 20191101
Time 13.23 h
INSTRUM avx500
PROBHD Z119877_0007 (
PULPROG zg60
TD 65536
SOLVENT CDCl3
NS 16
DS 2
SWH 10000.000 Hz
FIDRES 0.305176 Hz
AQ 3.2767999 sec
RG 70.83
DW 50.000 usec
DE 6.50 usec
TE 298.0 K
D1 1.00000000 sec
TD0 1
SFO1 500.1325007 MHz
NUC1 1H
P1 15.00 usec
PLW1 31.53700066 W
F2 - Processing parameters
SI 65536
SF 500.1300125 MHz
WDW EM
SSB 0
LB 0.30 Hz
GB 0
PC 1.00
11
10
9
8
7
6
5
4
3
2
1
ppm
4.56
1.00
2.90
0.97
2.08
2.08
4.11

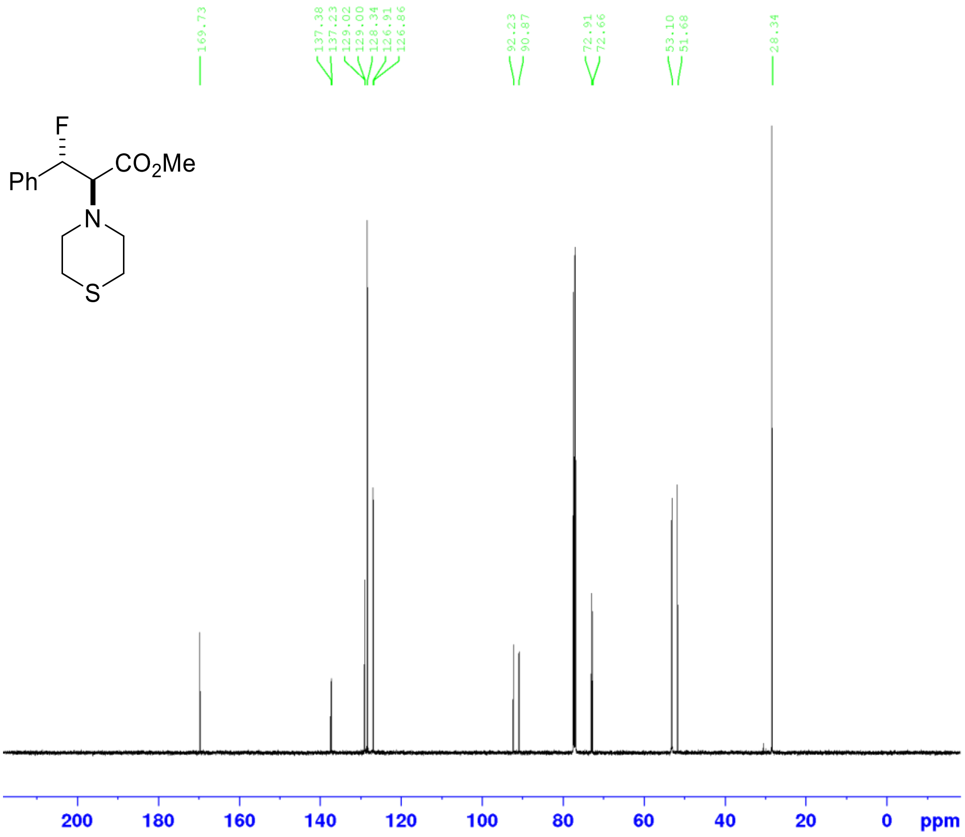

Current Data Parameters
NAME ka-3-152-C
EXPNO 3
PROCNO 1

F2 - Acquisition Parameters
Date_ 20191101
Time 13.53 h
INSTRUM avx500
PROBHD Z119877_0007 (
PULPROG zgpg30
TD 65536
SOLVENT CDCl3
NS 512
DS 4
SWH 29761.904 Hz
FIDRES 0.908261 Hz
AQ 1.1010048 sec
RG 191.37
DW 16.800 usec
DE 6.50 usec
TE 298.0 K
D1 2.00000000 sec
D11 0.03000000 sec
TD0 1
SFO1 125.7703643 MHz
NUC1 13C
P0 3.33 usec
P1 10.00 usec
PLW1 46.89899826 W
SFO2 500.1320005 MHz
NUC2 1H
CPDPRG[2 waltz16
PCPD2 80.00 usec
PLW2 31.53700066 W
PLW12 1.10870004 W
PLW13 0.55768001 W

F2 - Processing parameters
SI 32768
SF 125.7577773 MHz
WDW EM
SSB 0
LB 1.00 Hz
GB 0
PC 1.40

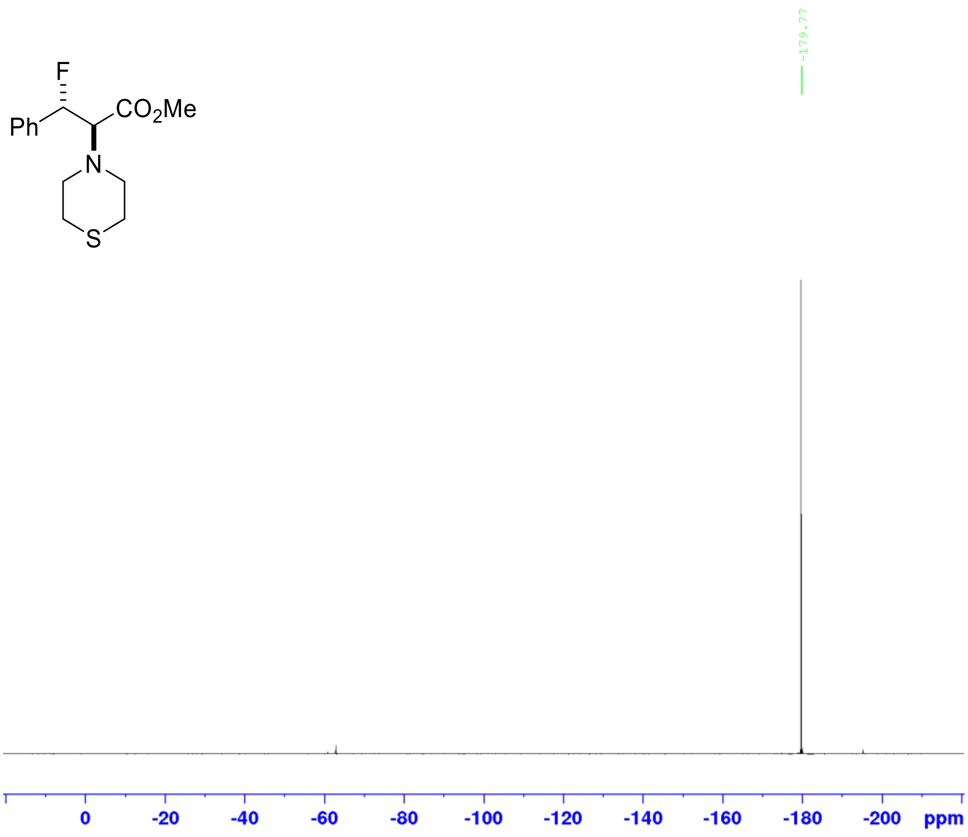

Current Data Parameters
NAME ka-3-152-C
EXPNO 23
PROCNO 1

F2 - Acquisition Parameters
Date_ 20191023
Time 13.55 h
INSTRUM Avance
PROBHD Z116098_0219 (
PULPROG zgig30
TD 130840
SOLVENT CDCl3
NS 8
DS 4
SWH 90909.094 Hz
FIDRES 1.389622 Hz
AQ 0.7196200 sec
RG 101
DW 5.500 usec
DE 6.50 usec
TE 298.0 K
D1 1.00000000 sec
D11 0.03000000 sec
TD0 1
SFO1 376.4607164 MHz
NUC1 19F
P0 4.67 usec
P1 14.00 usec
PLW1 25.46699905 W
SFO2 400.1316005 MHz
NUC2 1H
CPDPRG[2 waltz16
PCPD2 90.00 usec
PLW2 16.35899925 W
PLW12 0.20196000 W

F2 - Processing parameters
SI 65536
SF 376.4983662 MHz
WDW EM
SSB 0
LB 0.30 Hz
GB 0
PC 1.00

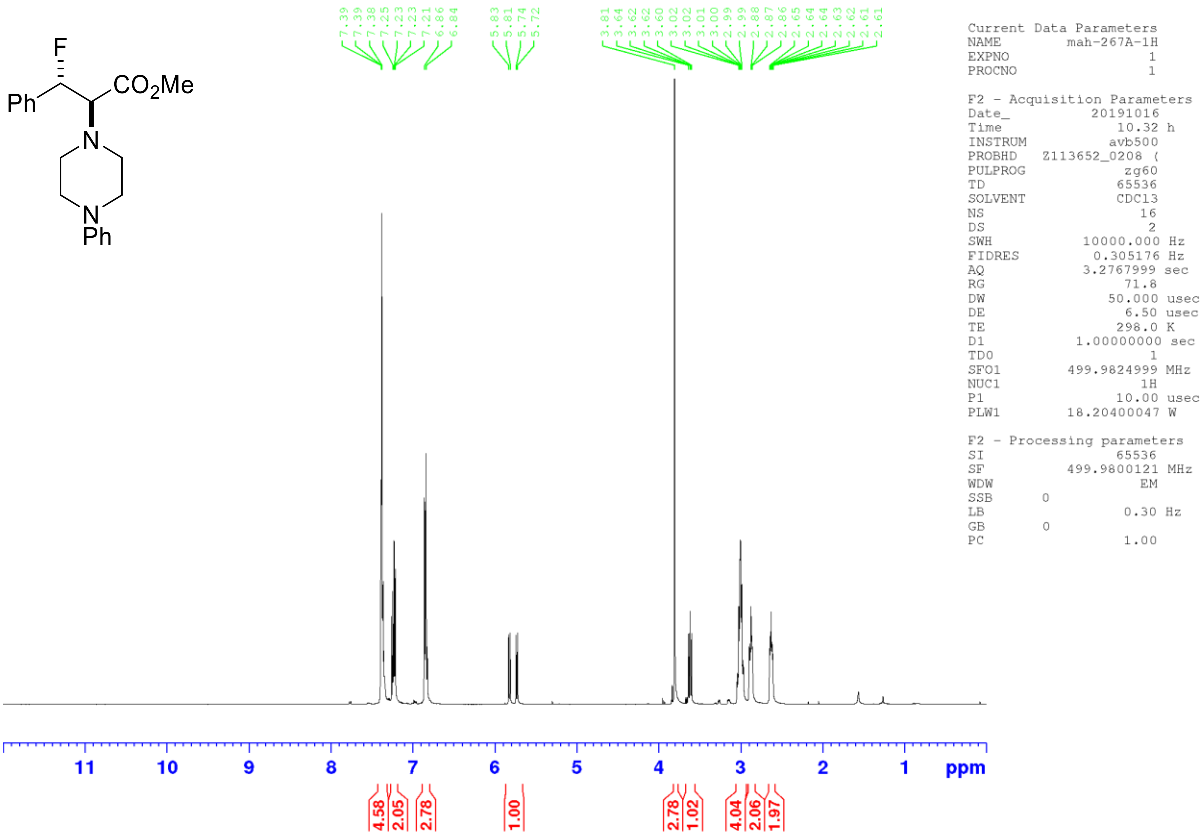


```
Current Data Parameters
NAME            mah-267A-1H
EXPNO                     1
PROCNO                    1

F2 - Acquisition Parameters
Date_              20191016
Time                10.32 h
INSTRUM              avb500
PROBHD    Z113652_0208 (
PULPROG                zg60
TD                    65536
SOLVENT               CDCl3
NS                       16
DS                        2
SWH           10000.000 Hz
FIDRES         0.305176 Hz
AQ            3.2767999 sec
RG                     71.8
DW               50.000 usec
DE                 6.50 usec
TE                298.0 K
D1           1.00000000 sec
TD0                       1
SFO1        499.9824999 MHz
NUC1                     1H
P1                10.00 usec
PLW1        18.20400047 W

F2 - Processing parameters
SI                    65536
SF          499.9800121 MHz
WDW                      EM
SSB       0
LB                 0.30 Hz
GB        0
PC                     1.00
```

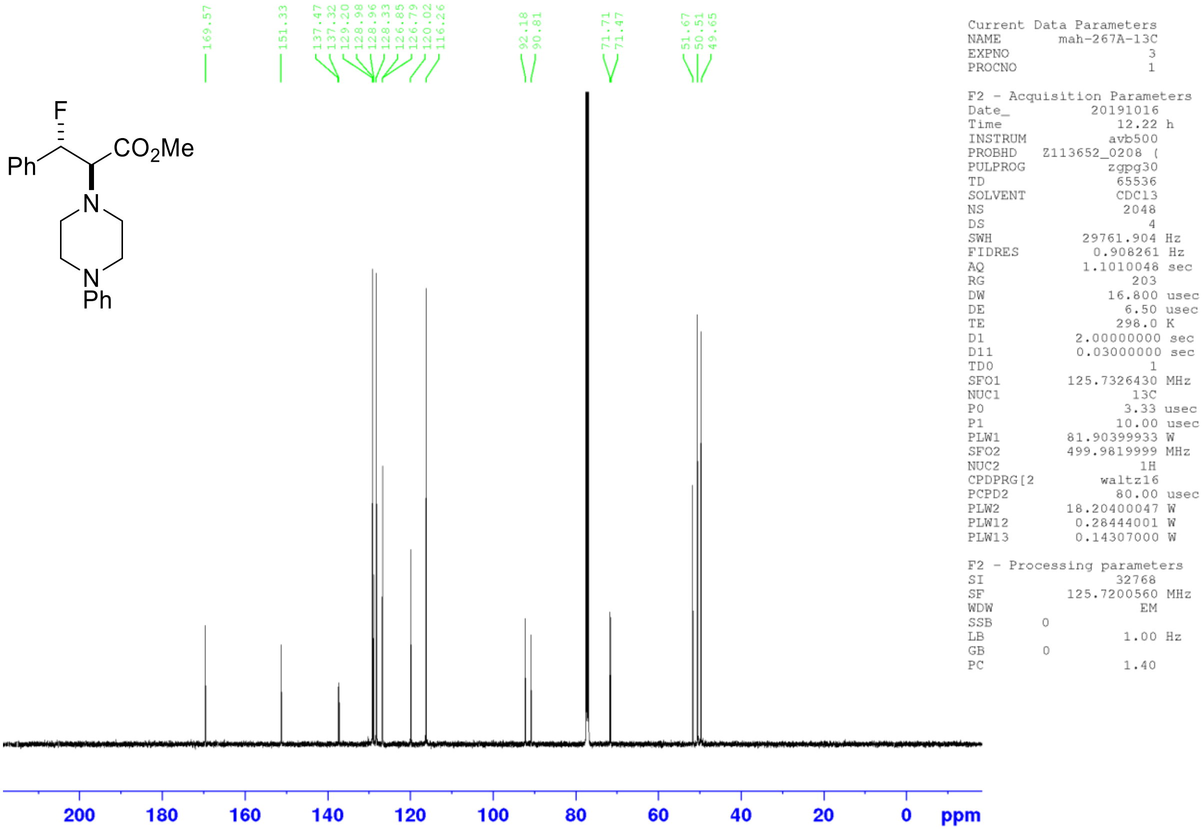


```
Current Data Parameters
NAME           mah-267A-13C
EXPNO                     3
PROCNO                    1

F2 - Acquisition Parameters
Date_              20191016
Time                12.22 h
INSTRUM              avb500
PROBHD    Z113652_0208 (
PULPROG             zgpg30
TD                    65536
SOLVENT               CDCl3
NS                     2048
DS                        4
SWH           29761.904 Hz
FIDRES         0.908261 Hz
AQ            1.1010048 sec
RG                      203
DW               16.800 usec
DE                 6.50 usec
TE                298.0 K
D1           2.00000000 sec
D11          0.03000000 sec
TD0                       1
SFO1        125.7326430 MHz
NUC1                    13C
P0                 3.33 usec
P1                10.00 usec
PLW1        81.90399933 W
SFO2        499.9819999 MHz
NUC2                     1H
CPDPRG[2            waltz16
PCPD2             80.00 usec
PLW2        18.20400047 W
PLW12        0.28444001 W
PLW13        0.14307000 W

F2 - Processing parameters
SI                    32768
SF          125.7200560 MHz
WDW                      EM
SSB       0
LB                 1.00 Hz
GB        0
PC                     1.40
```

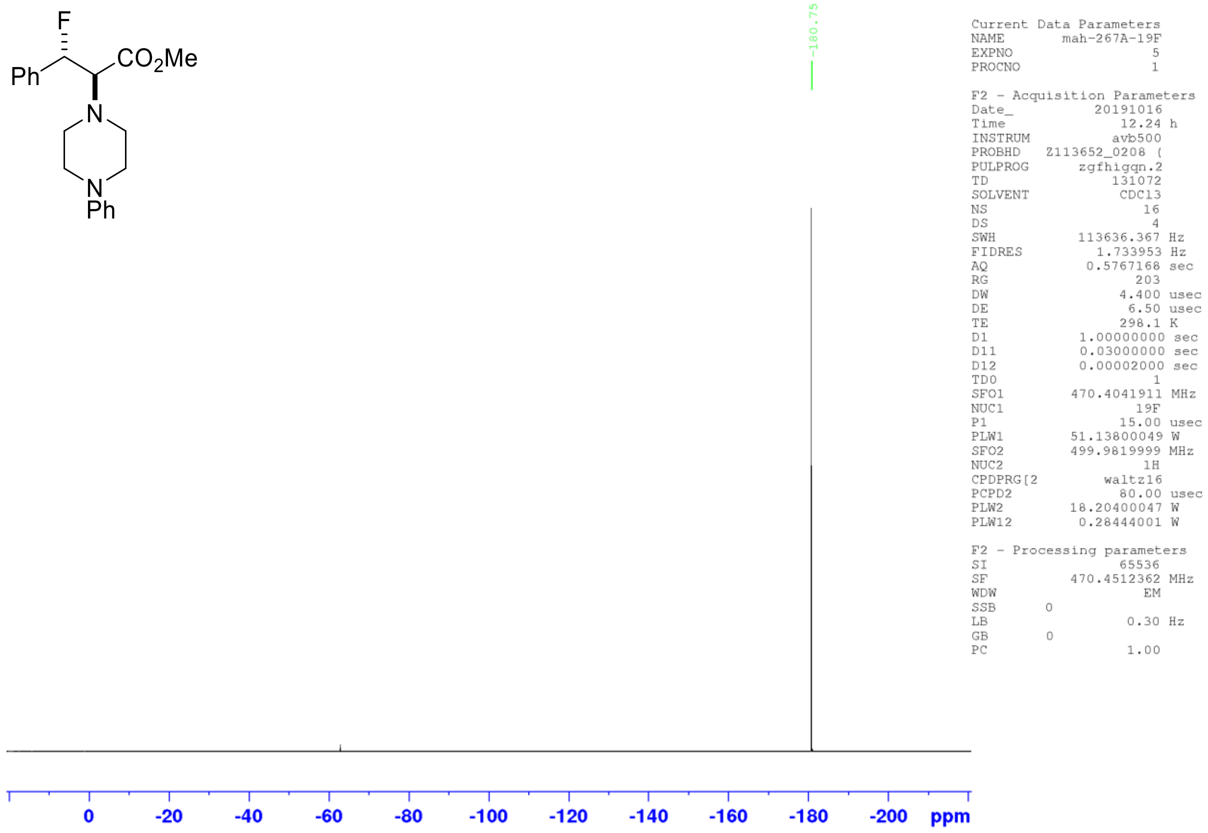
F
Ph
CO2Me
N
N
Ph
-180.75
Current Data Parameters
NAME mah-267A-19F
EXPNO 5
PROCNO 1
F2 - Acquisition Parameters
Date_ 20191016
Time 12.24 h
INSTRUM avb500
PROBHD Z113652_0208 (
PULPROG zgfhigqn.2
TD 131072
SOLVENT CDCl3
NS 16
DS 4
SWH 113636.367 Hz
FIDRES 1.733953 Hz
AQ 0.5767168 sec
RG 203
DW 4.400 usec
DE 6.50 usec
TE 298.1 K
D1 1.00000000 sec
D11 0.03000000 sec
D12 0.00002000 sec
TD0 1
SFO1 470.4041911 MHz
NUC1 19F
P1 15.00 usec
PLW1 51.13800049 W
SFO2 499.9819999 MHz
NUC2 1H
CPDPRG[2 waltz16
PCPD2 80.00 usec
PLW2 18.20400047 W
PLW12 0.28444001 W
F2 - Processing parameters
SI 65536
SF 470.4512362 MHz
WDW EM
SSB 0
LB 0.30 Hz
GB 0
PC 1.00
0 -20 -40 -60 -80 -100 -120 -140 -160 -180 -200 ppm

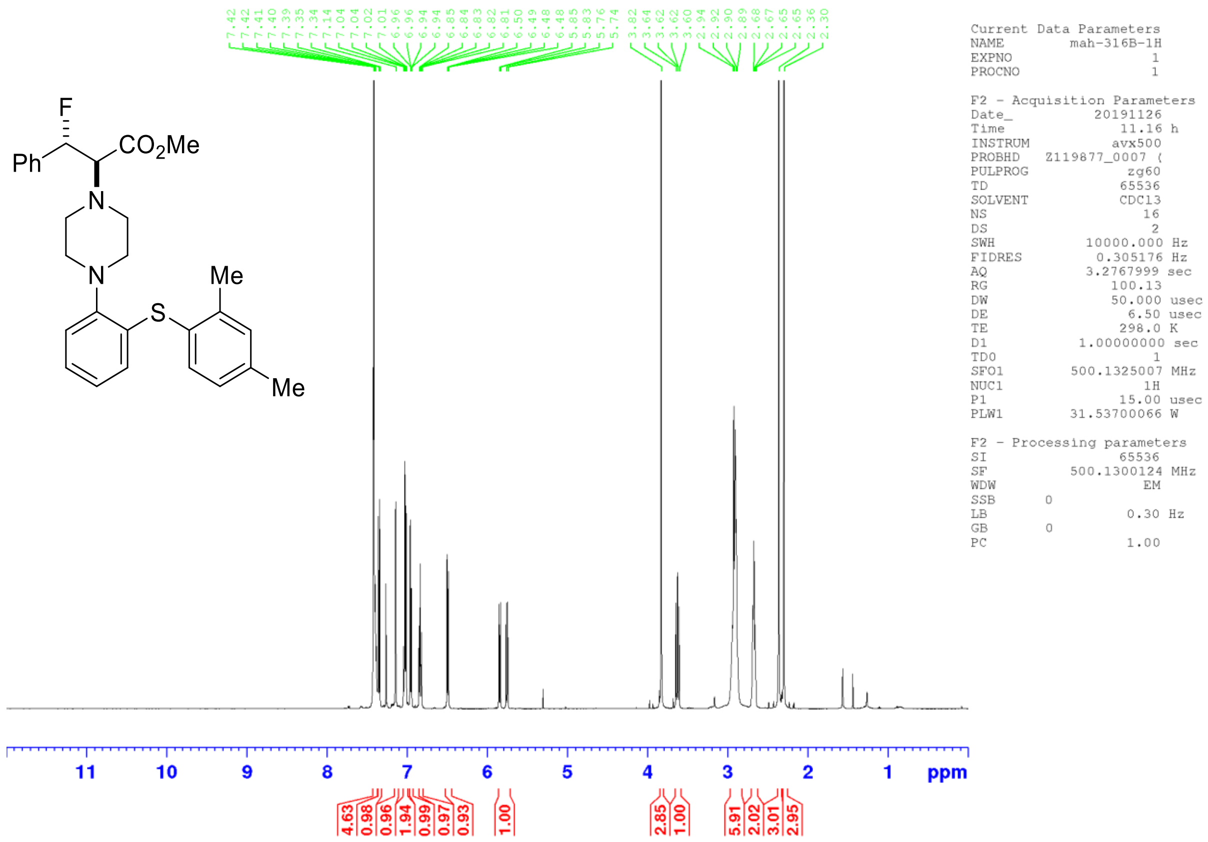
F
Ph
CO2Me
N
N
S
Me
Me
7.42 7.42 7.41 7.40 7.39 7.35 7.34 7.14 7.04 7.04 7.02 7.01 6.96 6.96 6.94 6.94 6.85 6.84 6.83 6.82 6.81 6.50 6.49 6.48 6.48 5.85 5.83 5.76 5.74 3.82 3.64 3.62 3.62 3.60 2.94 2.92 2.90 2.89 2.68 2.67 2.65 2.65 2.36 2.30
Current Data Parameters
NAME mah-316B-1H
EXPNO 1
PROCNO 1
F2 - Acquisition Parameters
Date_ 20191126
Time 11.16 h
INSTRUM avx500
PROBHD Z119877_0007 (
PULPROG zg60
TD 65536
SOLVENT CDCl3
NS 16
DS 2
SWH 10000.000 Hz
FIDRES 0.305176 Hz
AQ 3.2767999 sec
RG 100.13
DW 50.000 usec
DE 6.50 usec
TE 298.0 K
D1 1.00000000 sec
TD0 1
SFO1 500.1325007 MHz
NUC1 1H
P1 15.00 usec
PLW1 31.53700066 W
F2 - Processing parameters
SI 65536
SF 500.1300124 MHz
WDW EM
SSB 0
LB 0.30 Hz
GB 0
PC 1.00
11 10 9 8 7 6 5 4 3 2 1 ppm
4.63 0.98 0.96 1.94 0.99 0.97 0.93 1.00 2.85 1.00 5.91 2.02 3.01 2.95

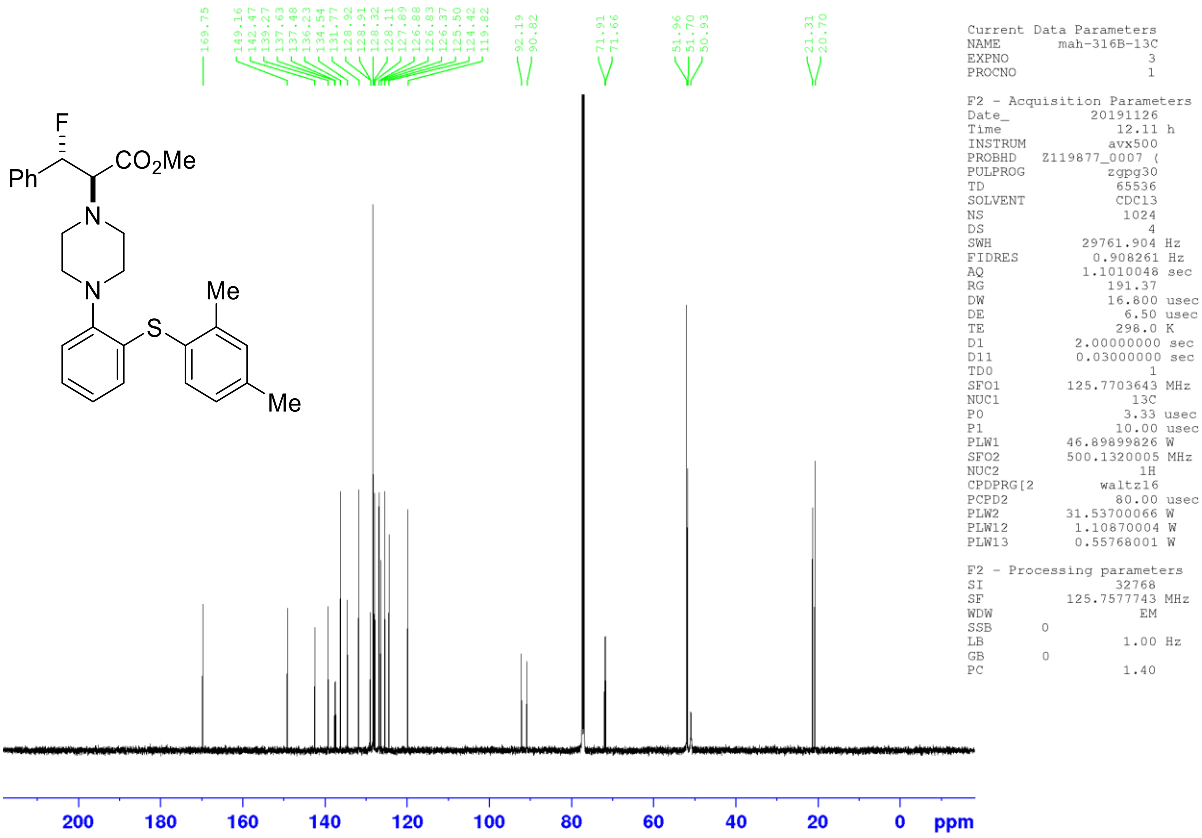


```
Current Data Parameters
NAME        mah-316B-13C
EXPNO                  3
PROCNO                 1

F2 - Acquisition Parameters
Date_           20191126
Time               12.11 h
INSTRUM           avx500
PROBHD    Z119877_0007 (
PULPROG          zgpg30
TD                 65536
SOLVENT            CDCl3
NS                  1024
DS                     4
SWH            29761.904 Hz
FIDRES          0.908261 Hz
AQ             1.1010048 sec
RG                191.37
DW                16.800 usec
DE                  6.50 usec
TE                 298.0 K
D1            2.00000000 sec
D11           0.03000000 sec
TD0                    1
SFO1         125.7703643 MHz
NUC1                 13C
P0                  3.33 usec
P1                 10.00 usec
PLW1         46.89899826 W
SFO2         500.1320005 MHz
NUC2                  1H
CPDPRG[2         waltz16
PCPD2              80.00 usec
PLW2         31.53700066 W
PLW12         1.10870004 W
PLW13         0.55768001 W

F2 - Processing parameters
SI                 32768
SF           125.7577743 MHz
WDW                   EM
SSB      0
LB                  1.00 Hz
GB       0
PC                  1.40
```

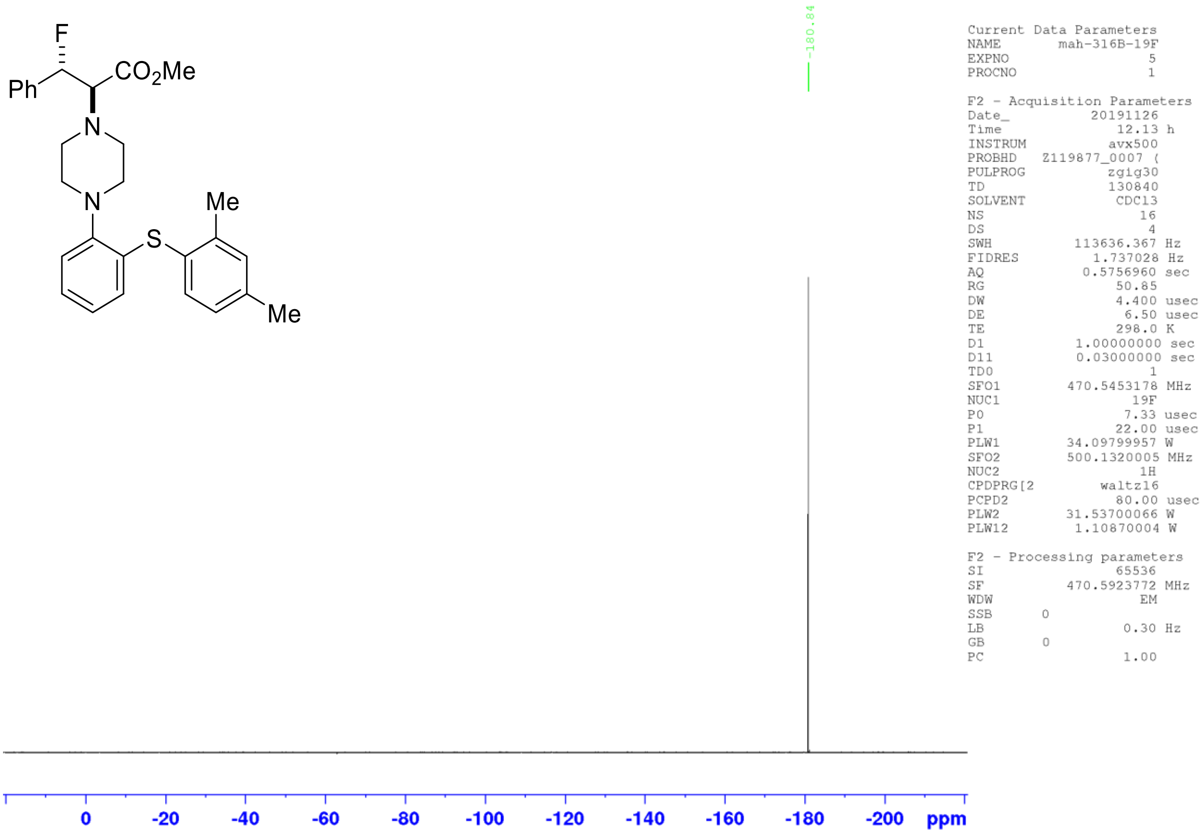


```
Current Data Parameters
NAME        mah-316B-19F
EXPNO                  5
PROCNO                 1

F2 - Acquisition Parameters
Date_           20191126
Time               12.13 h
INSTRUM           avx500
PROBHD    Z119877_0007 (
PULPROG          zgig30
TD                130840
SOLVENT            CDCl3
NS                    16
DS                     4
SWH           113636.367 Hz
FIDRES          1.737028 Hz
AQ             0.5756960 sec
RG                 50.85
DW                 4.400 usec
DE                  6.50 usec
TE                 298.0 K
D1            1.00000000 sec
D11           0.03000000 sec
TD0                    1
SFO1         470.5453178 MHz
NUC1                 19F
P0                  7.33 usec
P1                 22.00 usec
PLW1         34.09799957 W
SFO2         500.1320005 MHz
NUC2                  1H
CPDPRG[2         waltz16
PCPD2              80.00 usec
PLW2         31.53700066 W
PLW12         1.10870004 W

F2 - Processing parameters
SI                 65536
SF           470.5923772 MHz
WDW                   EM
SSB      0
LB                  0.30 Hz
GB       0
PC                  1.00
```

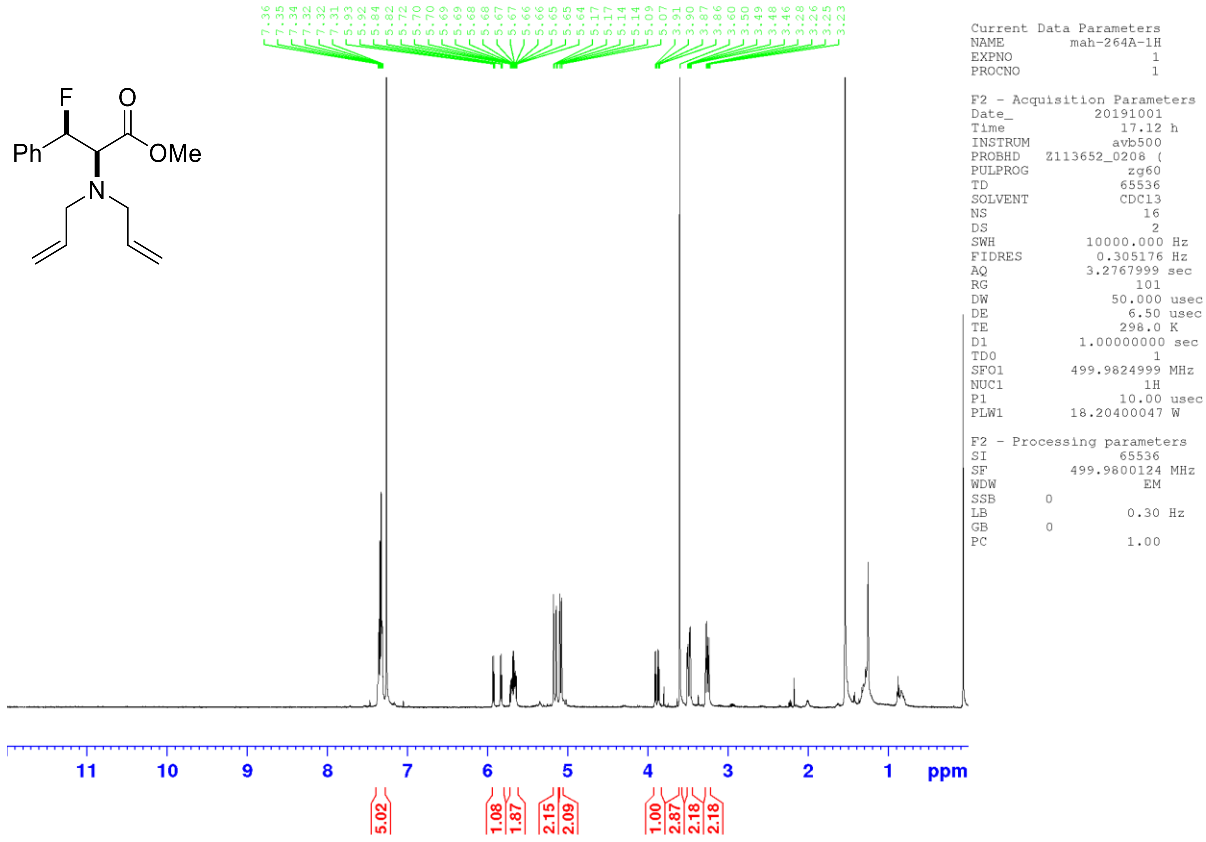


```
Current Data Parameters
NAME          mah-264A-1H
EXPNO                   1
PROCNO                  1

F2 - Acquisition Parameters
Date_            20191001
Time                17.12 h
INSTRUM            avb500
PROBHD   Z113652_0208 (
PULPROG              zg60
TD                  65536
SOLVENT             CDCl3
NS                     16
DS                      2
SWH             10000.000 Hz
FIDRES           0.305176 Hz
AQ              3.2767999 sec
RG                    101
DW                 50.000 usec
DE                   6.50 usec
TE                  298.0 K
D1             1.00000000 sec
TD0                     1
SFO1          499.9824999 MHz
NUC1                   1H
P1                  10.00 usec
PLW1          18.20400047 W

F2 - Processing parameters
SI                  65536
SF            499.9800124 MHz
WDW                    EM
SSB      0
LB                   0.30 Hz
GB       0
PC                   1.00
```

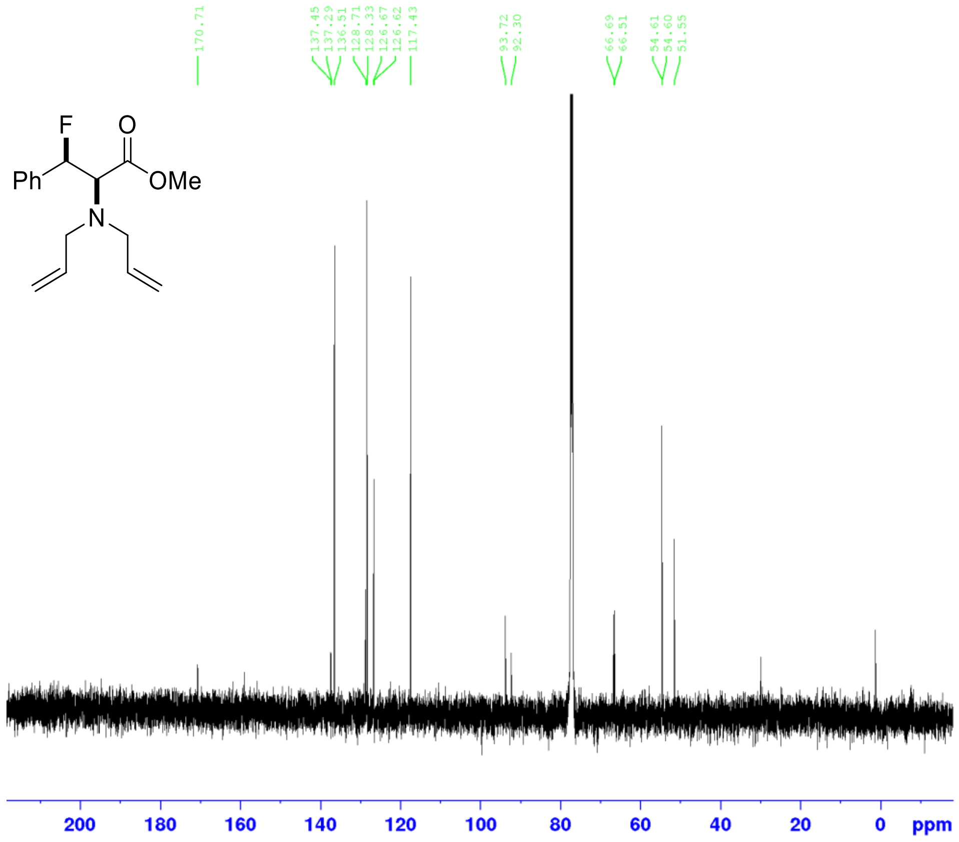


```
Current Data Parameters
NAME          mah-264A-13C
EXPNO                   4
PROCNO                  1

F2 - Acquisition Parameters
Date_            20191003
Time                14.59 h
INSTRUM            avb500
PROBHD   Z113652_0208 (
PULPROG           zgpg30
TD                  65536
SOLVENT             CDCl3
NS                   7168
DS                      4
SWH             29761.904 Hz
FIDRES           0.908261 Hz
AQ              1.1010048 sec
RG                    203
DW                 16.800 usec
DE                   6.50 usec
TE                  298.0 K
D1             2.00000000 sec
D11            0.03000000 sec
TD0                     1
SFO1          125.7326430 MHz
NUC1                  13C
P0                   3.33 usec
P1                  10.00 usec
PLW1          81.90399933 W
SFO2          499.9819999 MHz
NUC2                   1H
CPDPRG[2          waltz16
PCPD2               80.00 usec
PLW2          18.20400047 W
PLW12          0.28444001 W
PLW13          0.14307000 W

F2 - Processing parameters
SI                  32768
SF            125.7200533 MHz
WDW                    EM
SSB      0
LB                   1.00 Hz
GB       0
PC                   1.40
```

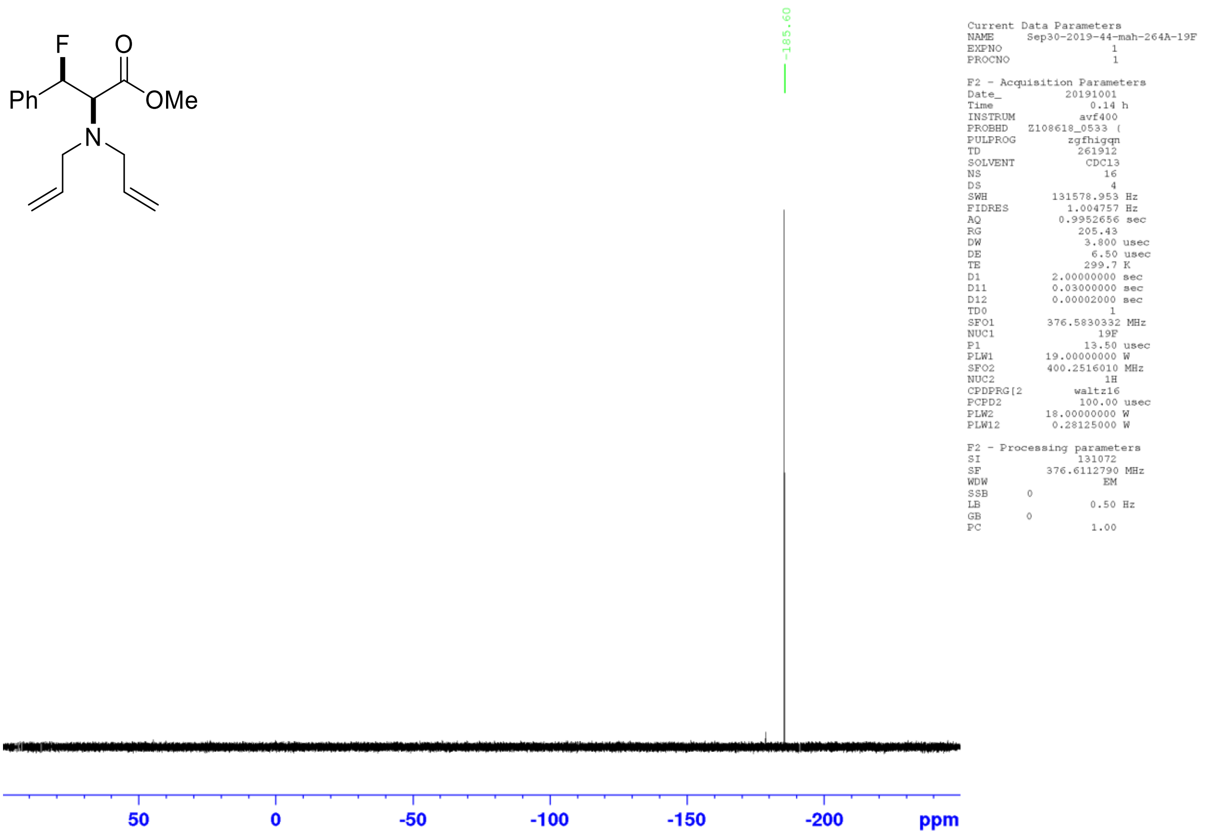
F
O
Ph
OMe
N
-185.60
Current Data Parameters
NAME Sep30-2019-44-mah-264A-19F
EXPNO 1
PROCNO 1
F2 - Acquisition Parameters
Date_ 20191001
Time 0.14 h
INSTRUM avf400
PROBHD Z108618_0533 (
PULPROG zgfhigqn
TD 261912
SOLVENT CDCl3
NS 16
DS 4
SWH 131578.953 Hz
FIDRES 1.004757 Hz
AQ 0.9952656 sec
RG 205.43
DW 3.800 usec
DE 6.50 usec
TE 299.7 K
D1 2.00000000 sec
D11 0.03000000 sec
D12 0.00002000 sec
TD0 1
SFO1 376.5830332 MHz
NUC1 19F
P1 13.50 usec
PLW1 19.00000000 W
SFO2 400.2516010 MHz
NUC2 1H
CPDPRG[2 waltz16
PCPD2 100.00 usec
PLW2 18.00000000 W
PLW12 0.28125000 W
F2 - Processing parameters
SI 131072
SF 376.6112790 MHz
WDW EM
SSB 0
LB 0.50 Hz
GB 0
PC 1.00
50
0
-50
-100
-150
-200
ppm

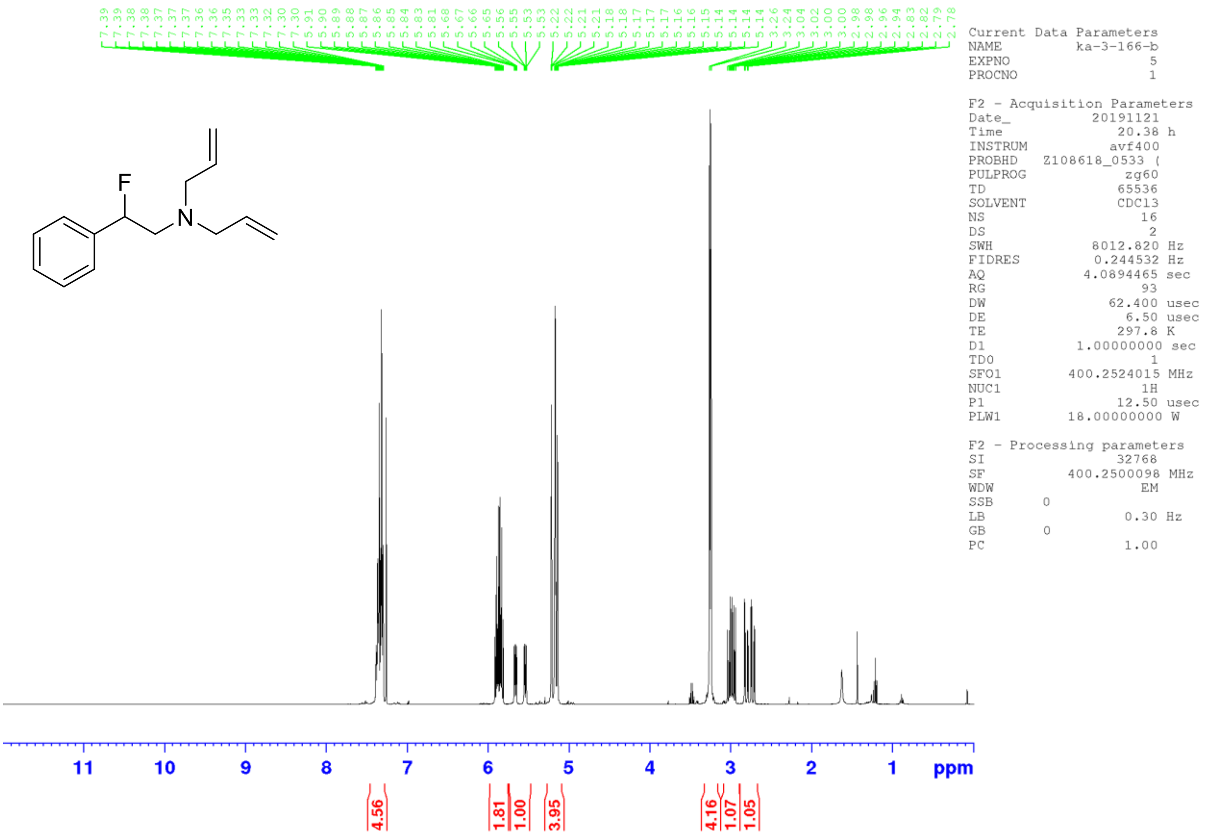
7.39 7.39 7.38 7.37 7.37 7.37 7.36 7.35 7.33 7.33 7.32 7.30 7.30 5.91 5.90 5.89 5.88 5.87 5.86 5.85 5.84 5.83 5.81 5.68 5.67 5.66 5.65 5.56 5.55 5.53 5.53 5.22 5.22 5.21 5.21 5.18 5.18 5.17 5.17 5.16 5.15 5.14 5.14 5.14 5.14 3.26 3.24 3.04 3.02 3.00 3.00 2.98 2.96 2.94 2.83 2.82 2.79 2.78
F
N
Current Data Parameters
NAME ka-3-166-b
EXPNO 5
PROCNO 1
F2 - Acquisition Parameters
Date_ 20191121
Time 20.38 h
INSTRUM avf400
PROBHD Z108618_0533 (
PULPROG zg60
TD 65536
SOLVENT CDCl3
NS 16
DS 2
SWH 8012.820 Hz
FIDRES 0.244532 Hz
AQ 4.0894465 sec
RG 93
DW 62.400 usec
DE 6.50 usec
TE 297.8 K
D1 1.00000000 sec
TD0 1
SFO1 400.2524015 MHz
NUC1 1H
P1 12.50 usec
PLW1 18.00000000 W
F2 - Processing parameters
SI 32768
SF 400.2500098 MHz
WDW EM
SSB 0
LB 0.30 Hz
GB 0
PC 1.00
11
10
9
8
7
6
5
4
3
2
1
ppm
4.56
1.81
1.00
3.95
4.16
1.07
1.05

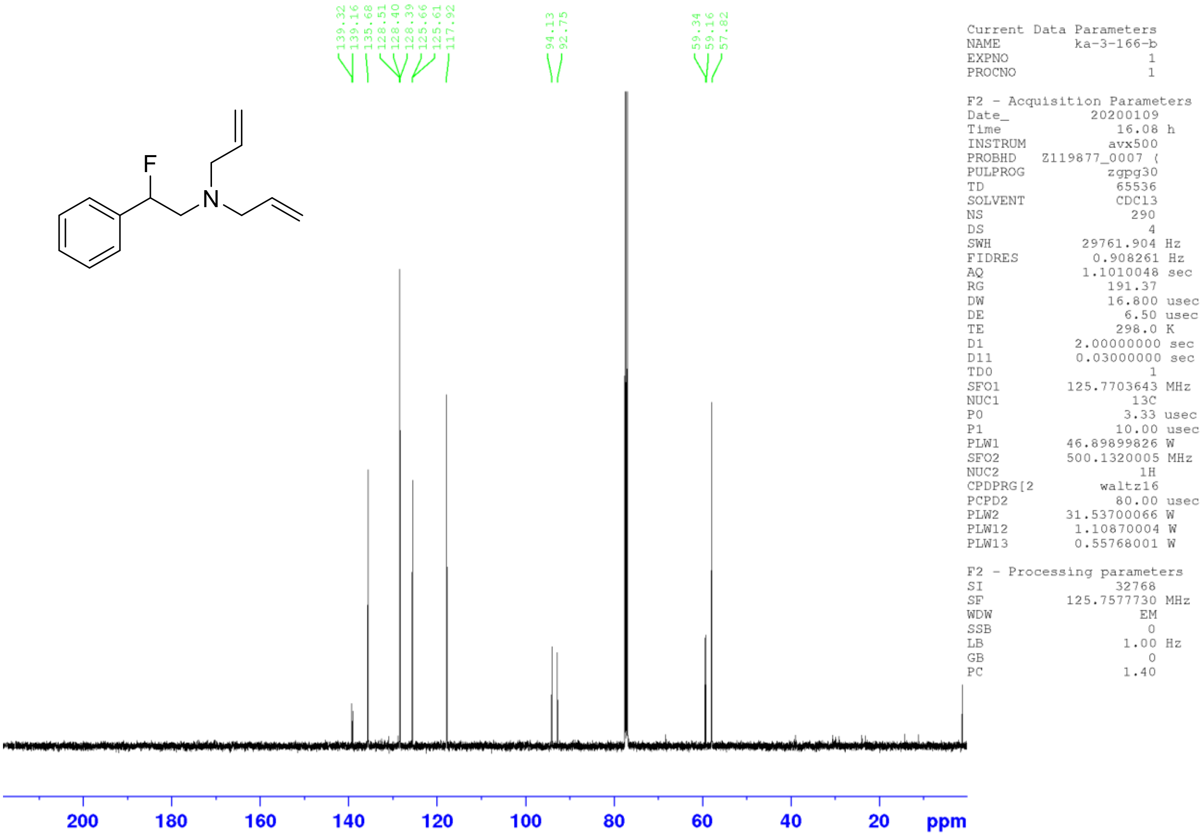

Current Data Parameters
NAME ka-3-166-b
EXPNO 1
PROCNO 1

F2 - Acquisition Parameters
Date_ 20200109
Time 16.08 h
INSTRUM avx500
PROBHD Z119877_0007 (
PULPROG zgpg30
TD 65536
SOLVENT CDCl3
NS 290
DS 4
SWH 29761.904 Hz
FIDRES 0.908261 Hz
AQ 1.1010048 sec
RG 191.37
DW 16.800 usec
DE 6.50 usec
TE 298.0 K
D1 2.00000000 sec
D11 0.03000000 sec
TD0 1
SFO1 125.7703643 MHz
NUC1 13C
P0 3.33 usec
P1 10.00 usec
PLW1 46.89899826 W
SFO2 500.1320005 MHz
NUC2 1H
CPDPRG[2 waltz16
PCPD2 80.00 usec
PLW2 31.53700066 W
PLW12 1.10870004 W
PLW13 0.55768001 W

F2 - Processing parameters
SI 32768
SF 125.7577730 MHz
WDW EM
SSB 0
LB 1.00 Hz
GB 0
PC 1.40

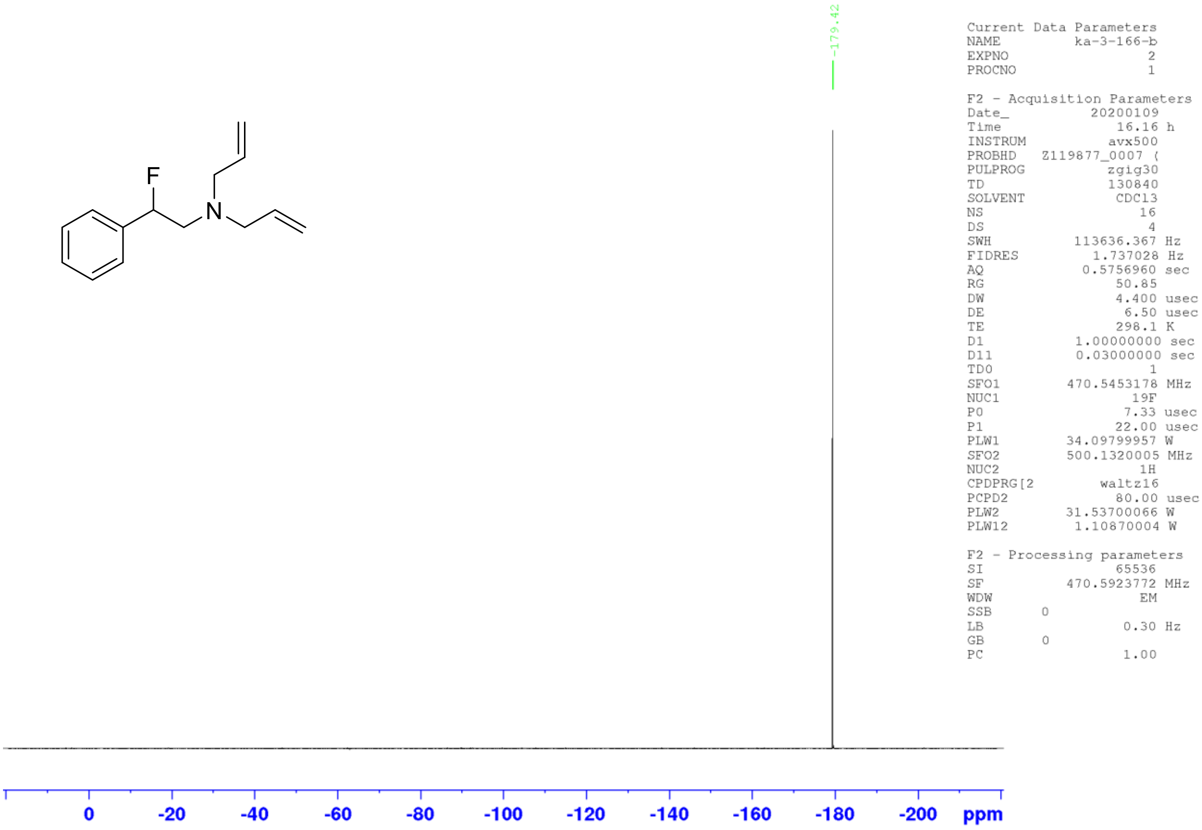

Current Data Parameters
NAME ka-3-166-b
EXPNO 2
PROCNO 1

F2 - Acquisition Parameters
Date_ 20200109
Time 16.16 h
INSTRUM avx500
PROBHD Z119877_0007 (
PULPROG zgig30
TD 130840
SOLVENT CDCl3
NS 16
DS 4
SWH 113636.367 Hz
FIDRES 1.737028 Hz
AQ 0.5756960 sec
RG 50.85
DW 4.400 usec
DE 6.50 usec
TE 298.1 K
D1 1.00000000 sec
D11 0.03000000 sec
TD0 1
SFO1 470.5453178 MHz
NUC1 19F
P0 7.33 usec
P1 22.00 usec
PLW1 34.09799957 W
SFO2 500.1320005 MHz
NUC2 1H
CPDPRG[2 waltz16
PCPD2 80.00 usec
PLW2 31.53700066 W
PLW12 1.10870004 W

F2 - Processing parameters
SI 65536
SF 470.5923772 MHz
WDW EM
SSB 0
LB 0.30 Hz
GB 0
PC 1.00

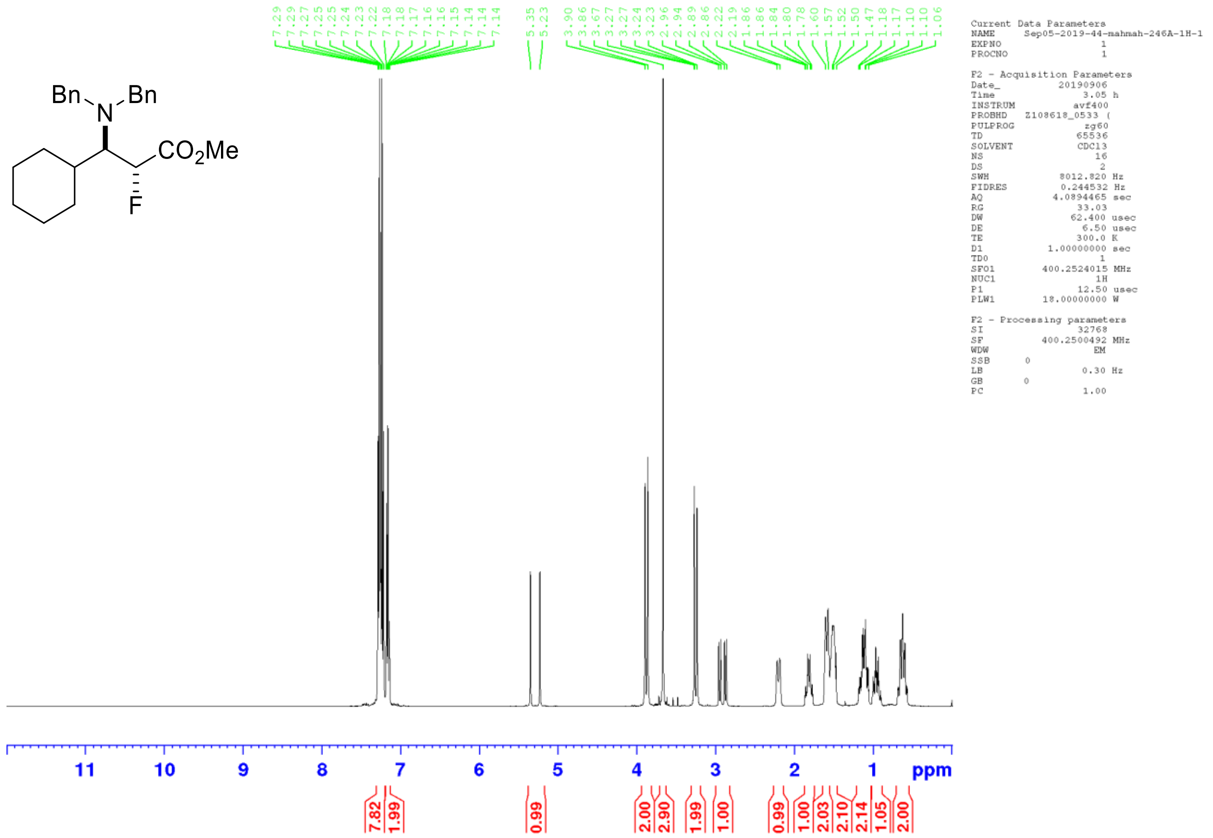
Bn
N
Bn
CO2Me
F
Current Data Parameters
NAME Sep05-2019-44-mahmah-246A-1H-1
EXPNO 1
PROCNO 1
F2 - Acquisition Parameters
Date_ 20190906
Time 3.05 h
INSTRUM avf400
PROBHD Z108618_0533 (
PULPROG zg60
TD 65536
SOLVENT CDCl3
NS 16
DS 2
SWH 8012.820 Hz
FIDRES 0.244532 Hz
AQ 4.0894465 sec
RG 33.03
DW 62.400 usec
DE 6.50 usec
TE 300.0 K
D1 1.00000000 sec
TD0 1
SFO1 400.2524015 MHz
NUC1 1H
P1 12.50 usec
PLW1 18.00000000 W
F2 - Processing parameters
SI 32768
SF 400.2500492 MHz
WDW EM
SSB 0
LB 0.30 Hz
GB 0
PC 1.00
ppm

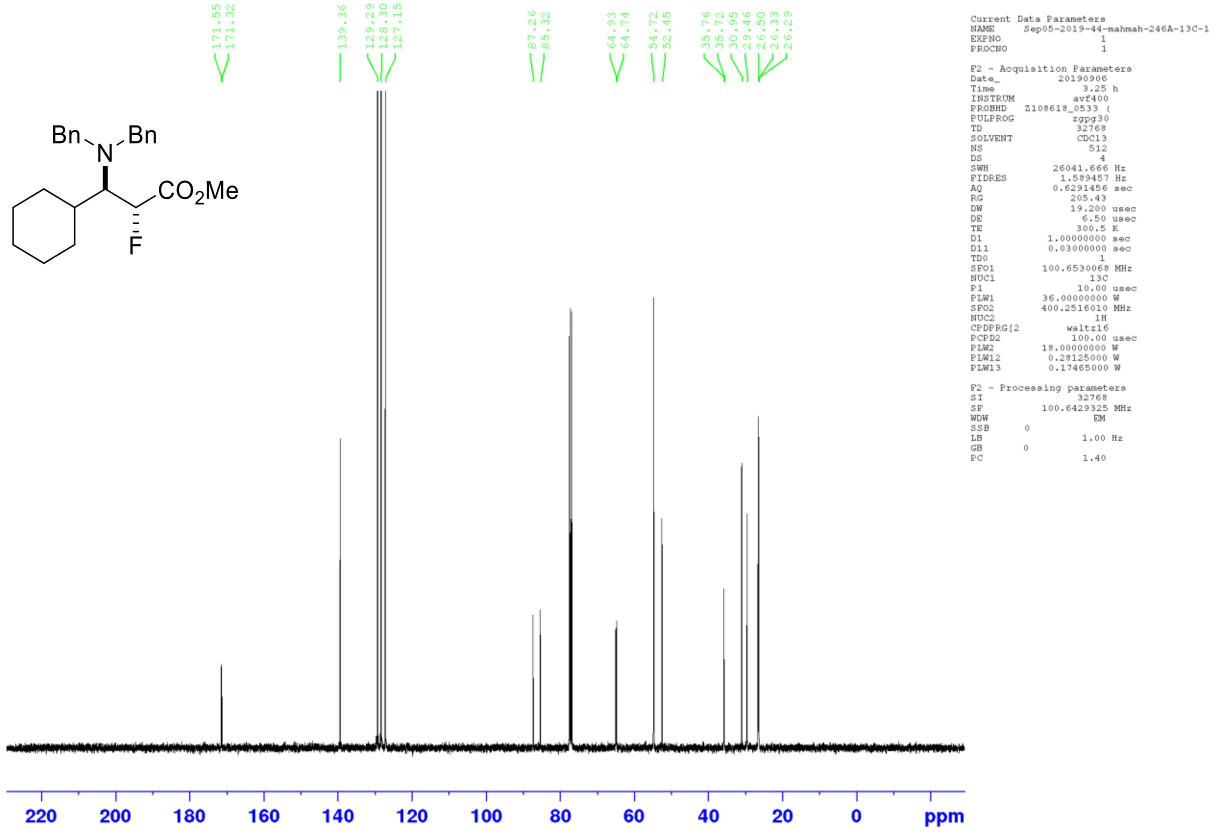
Bn
N
Bn
CO2Me
F
171.55
171.32
139.36
129.29
128.30
127.15
87.26
85.32
64.93
64.74
54.72
52.45
35.76
35.72
30.95
29.46
26.50
26.33
26.29
Current Data Parameters
NAME Sep05-2019-44-mahmah-246A-13C-1
EXPNO 1
PROCNO 1
F2 - Acquisition Parameters
Date_ 20190906
Time 3.25 h
INSTRUM avf400
PROBHD Z108618_0533 (
PULPROG zgpg30
TD 32768
SOLVENT CDCl3
NS 512
DS 4
SWH 26041.666 Hz
FIDRES 1.589457 Hz
AQ 0.6291456 sec
RG 205.43
DW 19.200 usec
DE 6.50 usec
TE 300.5 K
D1 1.00000000 sec
D11 0.03000000 sec
TD0 1
SFO1 100.6530068 MHz
NUC1 13C
P1 10.00 usec
PLW1 36.00000000 W
SFO2 400.2516010 MHz
NUC2 1H
CPDPRG[2 waltz16
PCPD2 100.00 usec
PLW2 18.00000000 W
PLW12 0.28125000 W
PLW13 0.17465000 W
F2 - Processing parameters
SI 32768
SF 100.6429325 MHz
WDW EM
SSB 0
LB 1.00 Hz
GB 0
PC 1.40
ppm

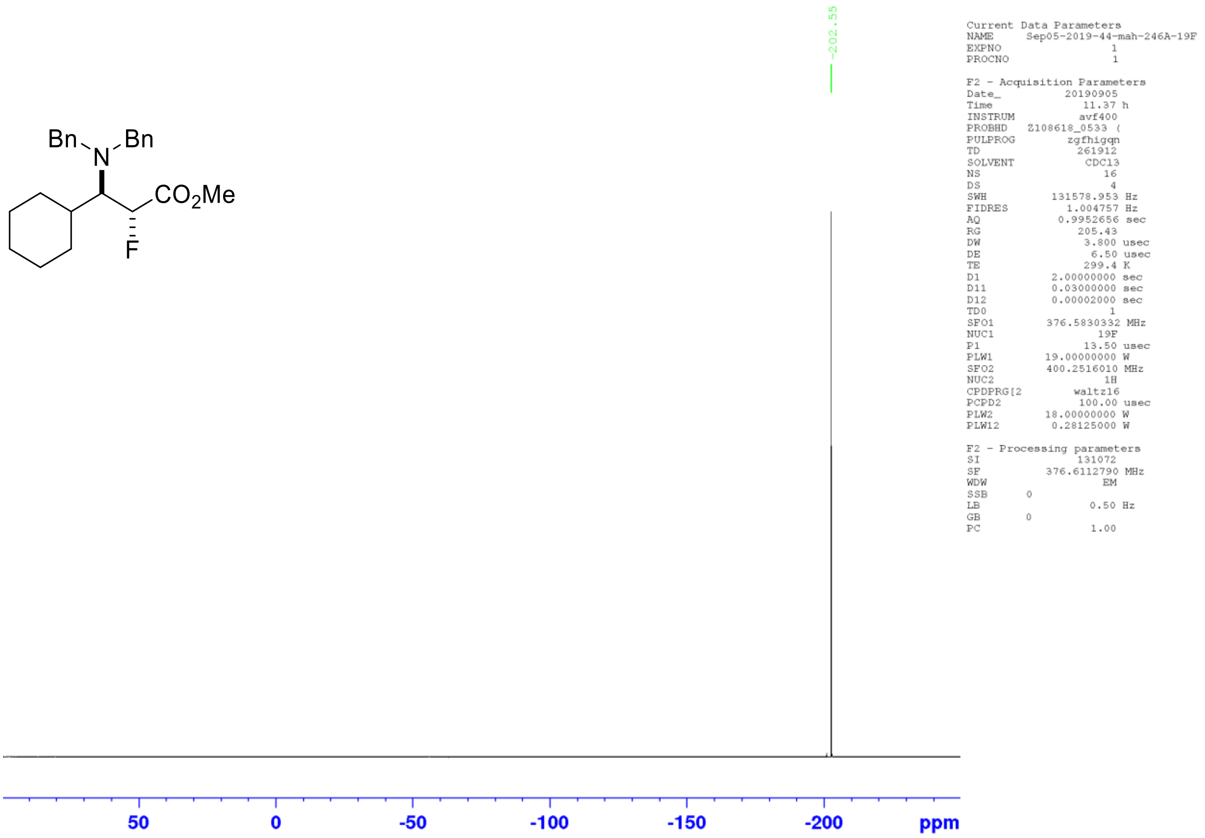
Bn
N
Bn
CO2Me
F
-202.55
Current Data Parameters
NAME Sep05-2019-44-mah-246A-19F
EXPNO 1
PROCNO 1
F2 - Acquisition Parameters
Date_ 20190905
Time 11.37 h
INSTRUM avf400
PROBHD Z108618_0533 (
PULPROG zgfhigqn
TD 261912
SOLVENT CDCl3
NS 16
DS 4
SWH 131578.953 Hz
FIDRES 1.004757 Hz
AQ 0.9952656 sec
RG 205.43
DW 3.800 usec
DE 6.50 usec
TE 299.4 K
D1 2.00000000 sec
D11 0.03000000 sec
D12 0.00002000 sec
TD0 1
SFO1 376.5830332 MHz
NUC1 19F
P1 13.50 usec
PLW1 19.00000000 W
SFO2 400.2516010 MHz
NUC2 1H
CPDPRG[2 waltz16
PCPD2 100.00 usec
PLW2 18.00000000 W
PLW12 0.28125000 W
F2 - Processing parameters
SI 131072
SF 376.6112790 MHz
WDW EM
SSB 0
LB 0.50 Hz
GB 0
PC 1.00
50
0
-50
-100
-150
-200
ppm

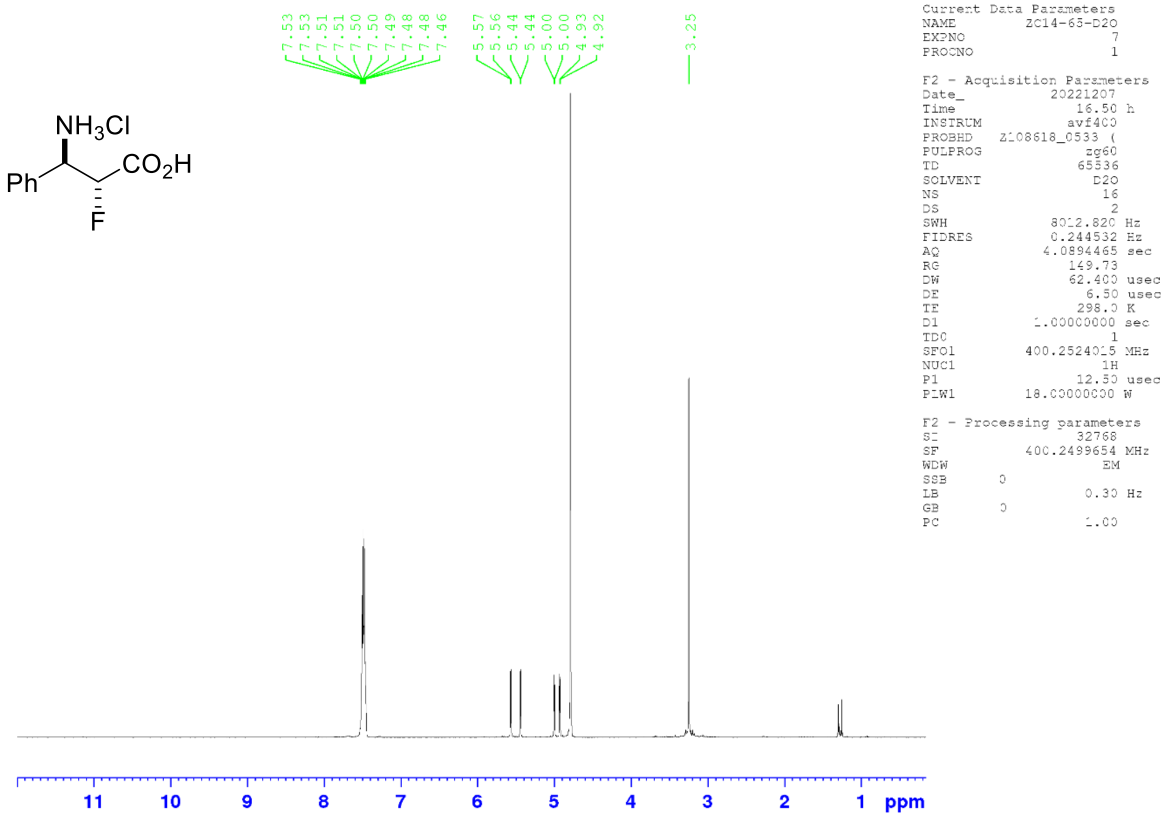
NH3Cl
Ph
CO2H
F
7.53 7.53 7.51 7.51 7.50 7.50 7.49 7.48 7.48 7.46
5.57 5.56 5.44 5.44 5.00 5.00 4.93 4.92
3.25
Current Data Parameters
NAME ZC14-65-D2O
EXPNO 7
PROCNO 1
F2 - Acquisition Parameters
Date_ 20221207
Time 16.50 h
INSTRUM avf400
PROBHD Z108618_0533 (
PULPROG zg60
TD 65536
SOLVENT D2O
NS 16
DS 2
SWH 8012.820 Hz
FIDRES 0.244532 Hz
AQ 4.0894465 sec
RG 149.73
DW 62.400 usec
DE 6.50 usec
TE 298.0 K
D1 1.00000000 sec
TD0 1
SFO1 400.2524015 MHz
NUC1 1H
P1 12.50 usec
PLW1 18.00000000 W
F2 - Processing parameters
SI 32768
SF 400.2499654 MHz
WDW EM
SSB 0
LB 0.30 Hz
GB 0
PC 1.00
11
10
9
8
7
6
5
4
3
2
1
ppm

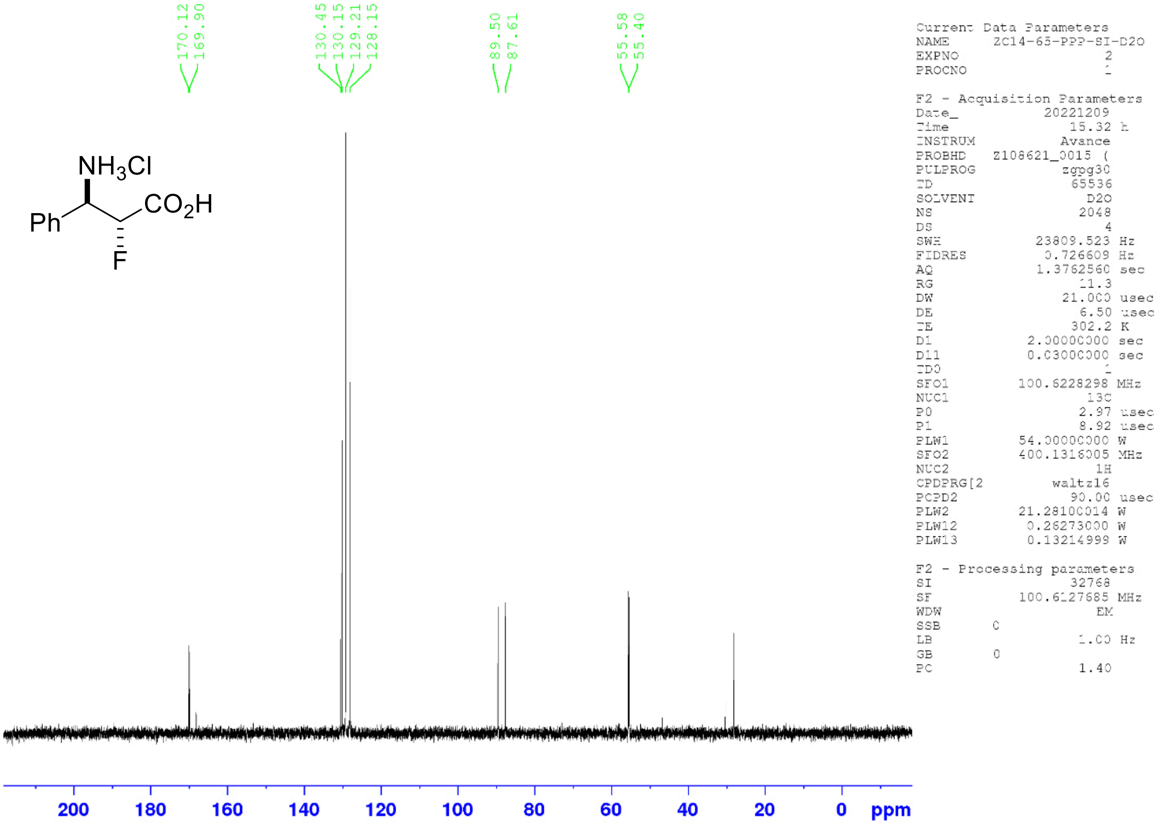
170.12
169.90
130.45
130.15
129.21
128.15
89.50
87.61
55.58
55.40
NH3Cl
CO2H
Ph
F
Current Data Parameters
NAME ZC14-65-PPP-SI-D2O
EXPNO 2
PROCNO 1
F2 - Acquisition Parameters
Date_ 20221209
Time 15.32 h
INSTRUM Avance
PROBHD Z108621_0015 (
PULPROG zgpg30
TD 65536
SOLVENT D2O
NS 2048
DS 4
SWH 23809.523 Hz
FIDRES 0.726609 Hz
AQ 1.3762560 sec
RG 11.3
DW 21.000 usec
DE 6.50 usec
TE 302.2 K
D1 2.00000000 sec
D11 0.03000000 sec
TD0 1
SFO1 100.6228298 MHz
NUC1 13C
P0 2.97 usec
P1 8.92 usec
PLW1 54.00000000 W
SFO2 400.1316005 MHz
NUC2 1H
CPDPRG[2 waltz16
PCPD2 90.00 usec
PLW2 21.28100014 W
PLW12 0.26273000 W
PLW13 0.13214999 W
F2 - Processing parameters
SI 32768
SF 100.6127685 MHz
WDW EM
SSB 0
LB 1.00 Hz
GB 0
PC 1.40
200
180
160
140
120
100
80
60
40
20
0
ppm

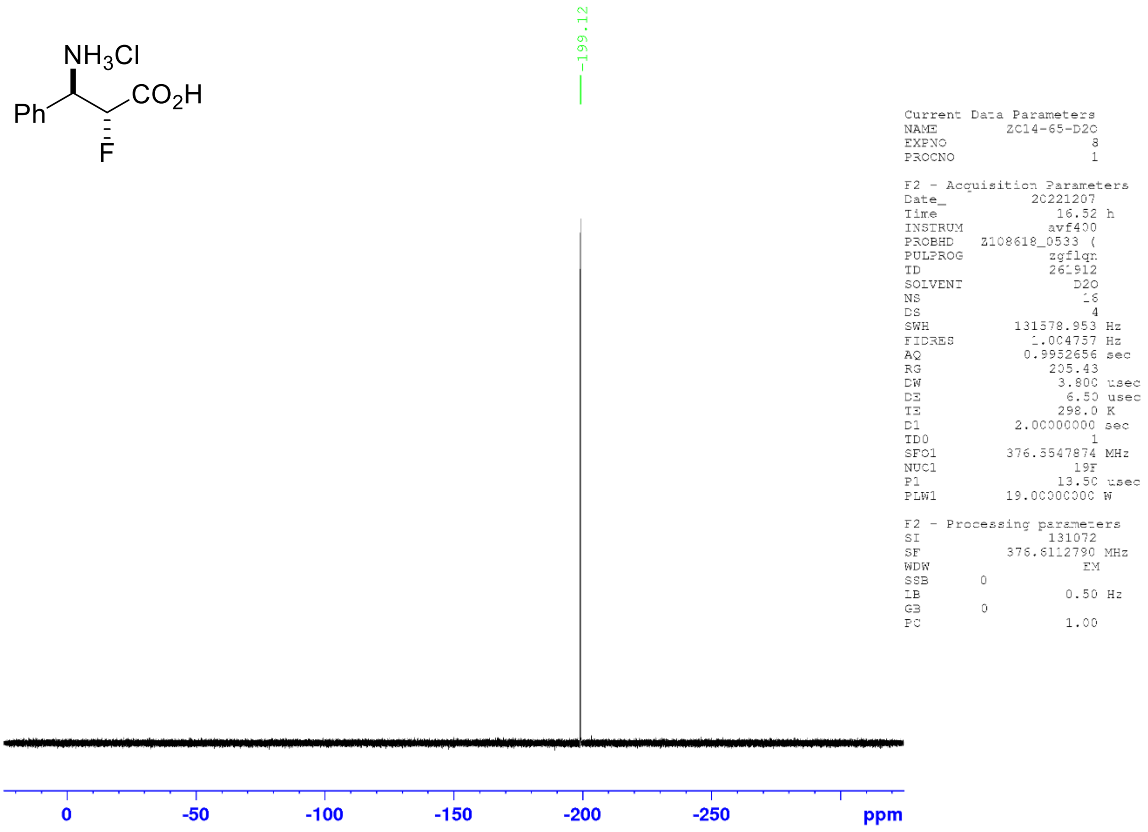
-199.12
NH3Cl
CO2H
Ph
F
Current Data Parameters
NAME ZC14-65-D2O
EXPNO 8
PROCNO 1
F2 - Acquisition Parameters
Date_ 20221207
Time 16.52 h
INSTRUM avf400
PROBHD Z108618_0533 (
PULPROG zgflqn
TD 261912
SOLVENT D2O
NS 16
DS 4
SWH 131578.953 Hz
FIDRES 1.004757 Hz
AQ 0.9952656 sec
RG 205.43
DW 3.800 usec
DE 6.50 usec
TE 298.0 K
D1 2.00000000 sec
TD0 1
SFO1 376.5547874 MHz
NUC1 19F
P1 13.50 usec
PLW1 19.00000000 W
F2 - Processing parameters
SI 131072
SF 376.6112790 MHz
WDW EM
SSB 0
LB 0.50 Hz
GB 0
PC 1.00
0
-50
-100
-150
-200
-250
ppm

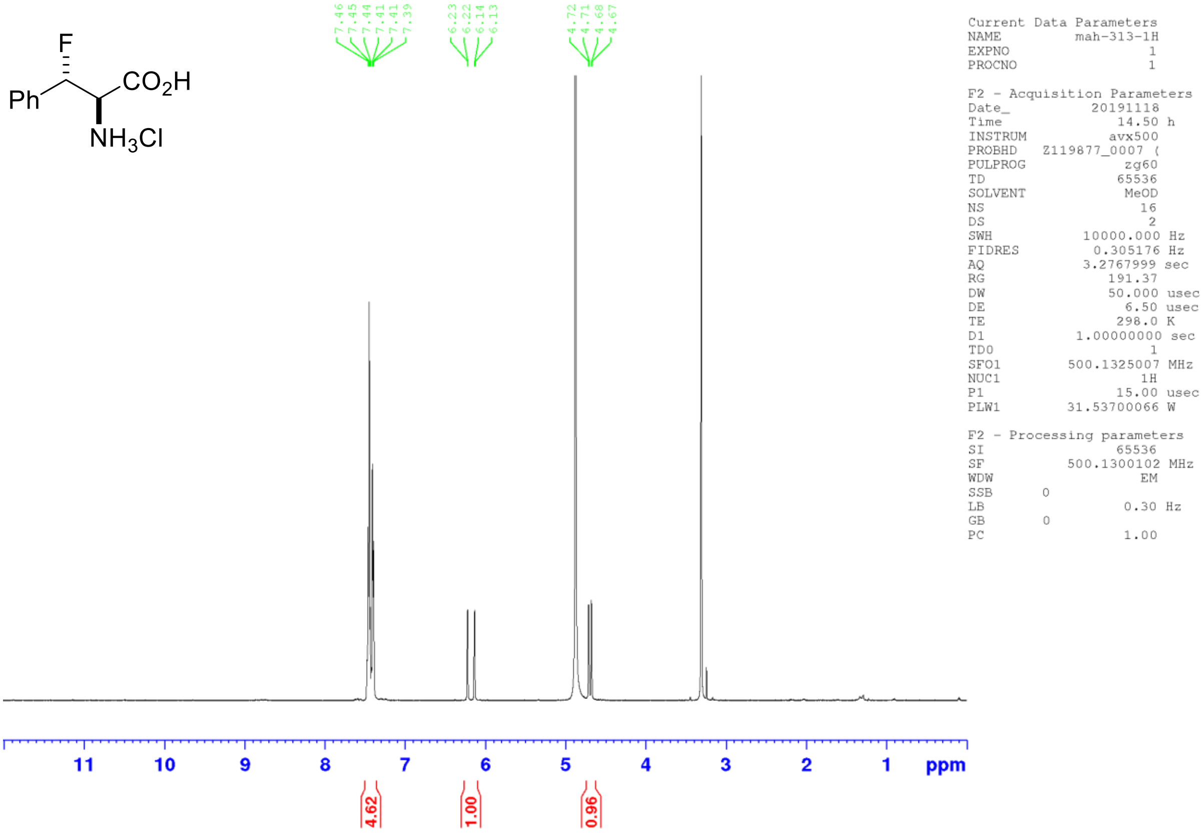
F
Ph
CO2H
NH3Cl
7.46 7.45 7.44 7.41 7.41 7.39
6.23 6.22 6.14 6.13
4.72 4.71 4.68 4.67
11 10 9 8 7 6 5 4 3 2 1 ppm
4.62
1.00
0.96
Current Data Parameters
NAME mah-313-1H
EXPNO 1
PROCNO 1
F2 - Acquisition Parameters
Date_ 20191118
Time 14.50 h
INSTRUM avx500
PROBHD Z119877_0007 (
PULPROG zg60
TD 65536
SOLVENT MeOD
NS 16
DS 2
SWH 10000.000 Hz
FIDRES 0.305176 Hz
AQ 3.2767999 sec
RG 191.37
DW 50.000 usec
DE 6.50 usec
TE 298.0 K
D1 1.00000000 sec
TD0 1
SFO1 500.1325007 MHz
NUC1 1H
P1 15.00 usec
PLW1 31.53700066 W
F2 - Processing parameters
SI 65536
SF 500.1300102 MHz
WDW EM
SSB 0
LB 0.30 Hz
GB 0
PC 1.00

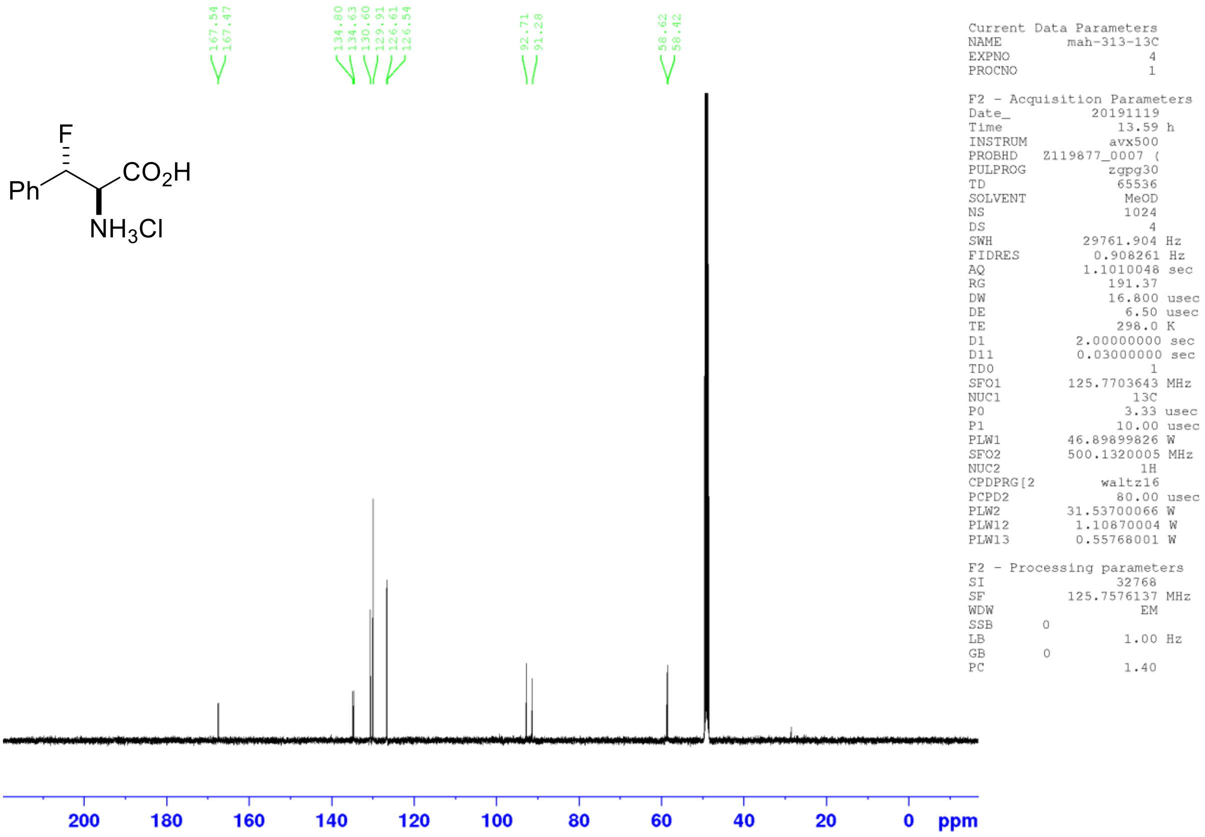
F
Ph
CO2H
NH3Cl
167.54 167.47
134.80 134.63 130.60 129.91 126.61 126.54
92.71 91.28
58.62 58.42
200 180 160 140 120 100 80 60 40 20 0 ppm
Current Data Parameters
NAME mah-313-13C
EXPNO 4
PROCNO 1
F2 - Acquisition Parameters
Date_ 20191119
Time 13.59 h
INSTRUM avx500
PROBHD Z119877_0007 (
PULPROG zgpg30
TD 65536
SOLVENT MeOD
NS 1024
DS 4
SWH 29761.904 Hz
FIDRES 0.908261 Hz
AQ 1.1010048 sec
RG 191.37
DW 16.800 usec
DE 6.50 usec
TE 298.0 K
D1 2.00000000 sec
D11 0.03000000 sec
TD0 1
SFO1 125.7703643 MHz
NUC1 13C
P0 3.33 usec
P1 10.00 usec
PLW1 46.89899826 W
SFO2 500.1320005 MHz
NUC2 1H
CPDPRG[2 waltz16
PCPD2 80.00 usec
PLW2 31.53700066 W
PLW12 1.10870004 W
PLW13 0.55768001 W
F2 - Processing parameters
SI 32768
SF 125.7576137 MHz
WDW EM
SSB 0
LB 1.00 Hz
GB 0
PC 1.40

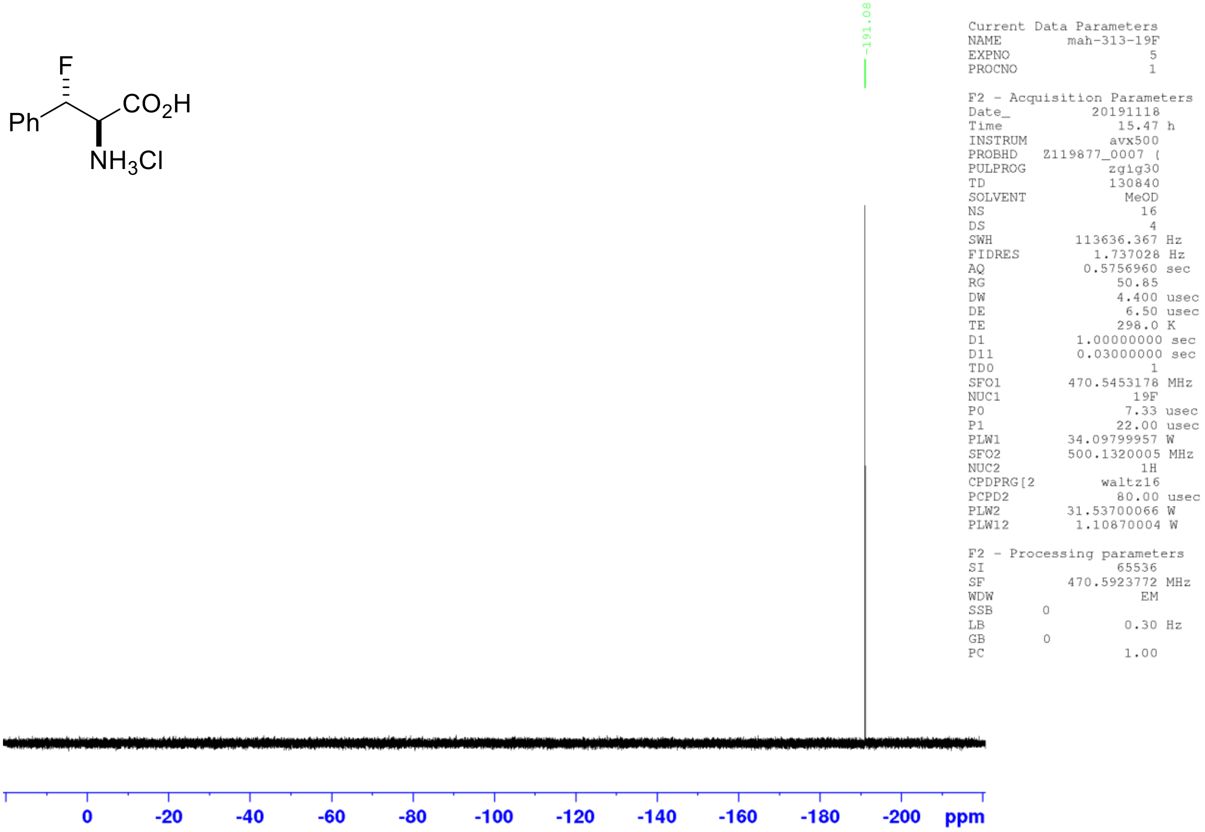
F
Ph
CO_2H
NH_3Cl
-191.08
Current Data Parameters
NAME mah-313-19F
EXPNO 5
PROCNO 1
F2 - Acquisition Parameters
Date_ 20191118
Time 15.47 h
INSTRUM avx500
PROBHD Z119877_0007 (
PULPROG zgig30
TD 130840
SOLVENT MeOD
NS 16
DS 4
SWH 113636.367 Hz
FIDRES 1.737028 Hz
AQ 0.5756960 sec
RG 50.85
DW 4.400 usec
DE 6.50 usec
TE 298.0 K
D1 1.00000000 sec
D11 0.03000000 sec
TD0 1
SFO1 470.5453178 MHz
NUC1 19F
P0 7.33 usec
P1 22.00 usec
PLW1 34.09799957 W
SFO2 500.1320005 MHz
NUC2 1H
CPDPRG[2 waltz16
PCPD2 80.00 usec
PLW2 31.53700066 W
PLW12 1.10870004 W
F2 - Processing parameters
SI 65536
SF 470.5923772 MHz
WDW EM
SSB 0
LB 0.30 Hz
GB 0
PC 1.00
0
-20
-40
-60
-80
-100
-120
-140
-160
-180
-200
ppm

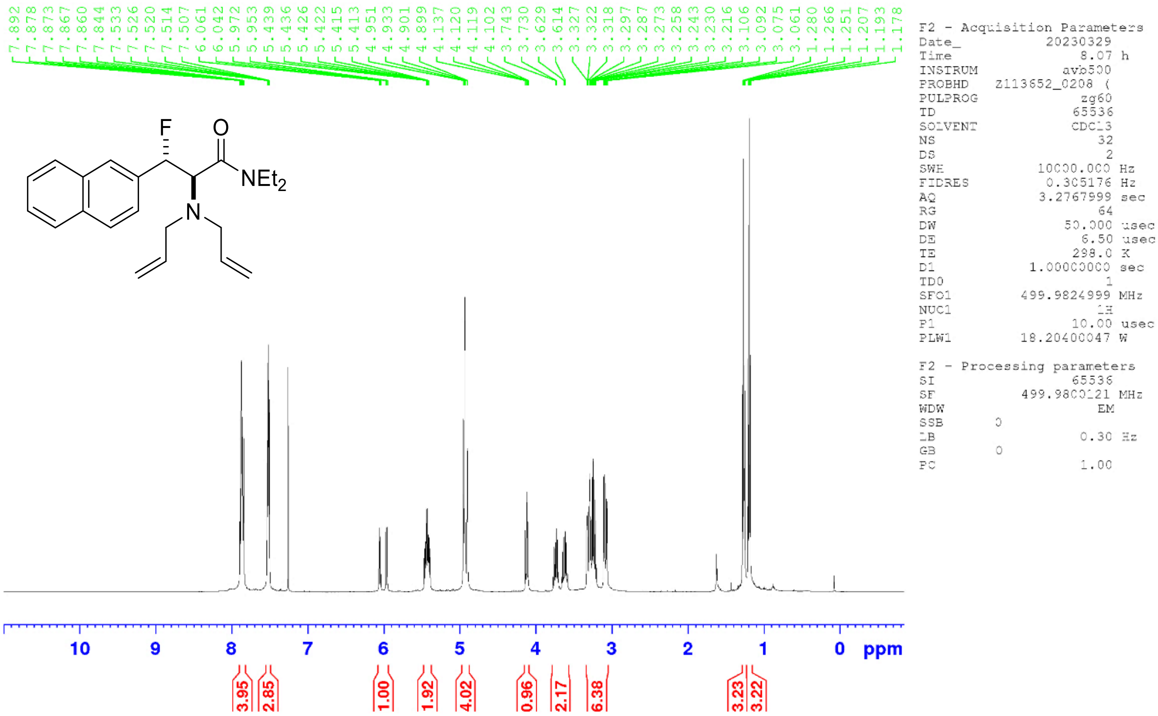
F
O
NEt_2
N
F2 - Acquisition Parameters
Date_ 20230329
Time 8.07 h
INSTRUM avb500
PROBHD Z113652_0208 (
PULPROG zg60
TD 65536
SOLVENT CDCl3
NS 32
DS 2
SWH 10000.000 Hz
FIDRES 0.305176 Hz
AQ 3.2767999 sec
RG 64
DW 50.000 usec
DE 6.50 usec
TE 298.0 K
D1 1.00000000 sec
TD0 1
SFO1 499.9824999 MHz
NUC1 1H
P1 10.00 usec
PLW1 18.20400047 W
F2 - Processing parameters
SI 65536
SF 499.9800121 MHz
WDW EM
SSB 0
LB 0.30 Hz
GB 0
PC 1.00
10
9
8
7
6
5
4
3
2
1
0
ppm
3.95
2.85
1.00
1.92
4.02
0.96
2.17
6.38
3.23
3.22

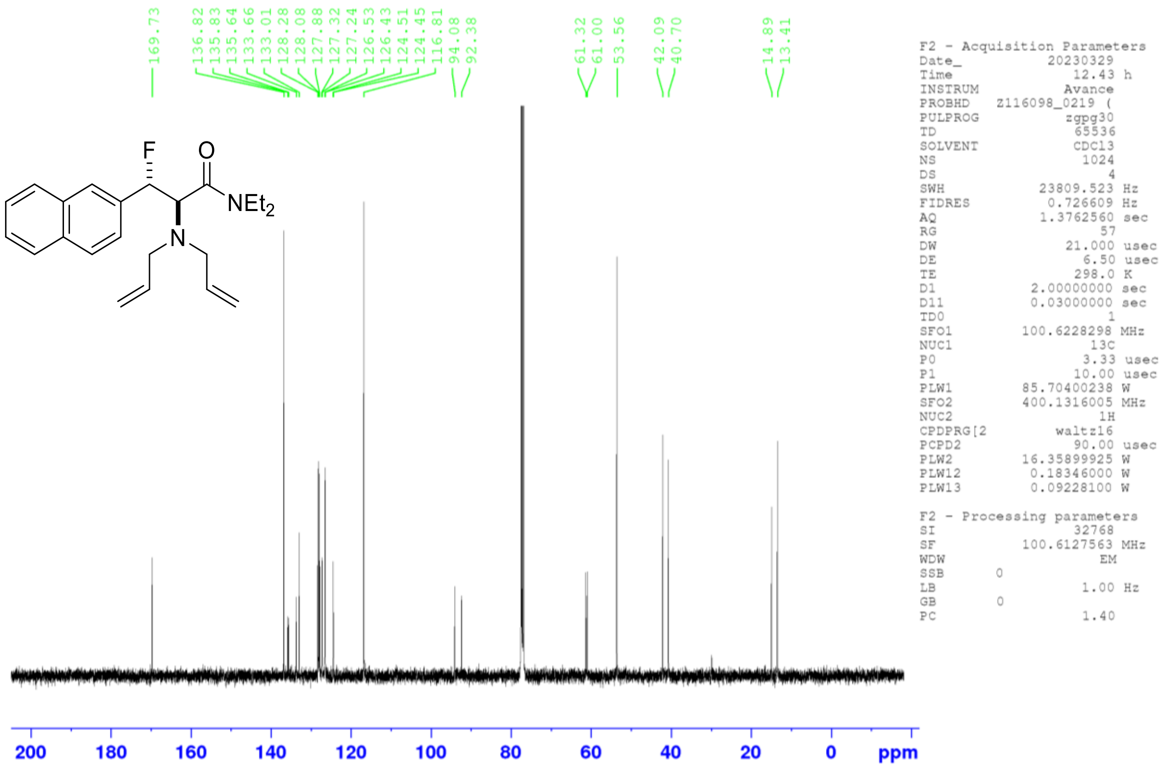

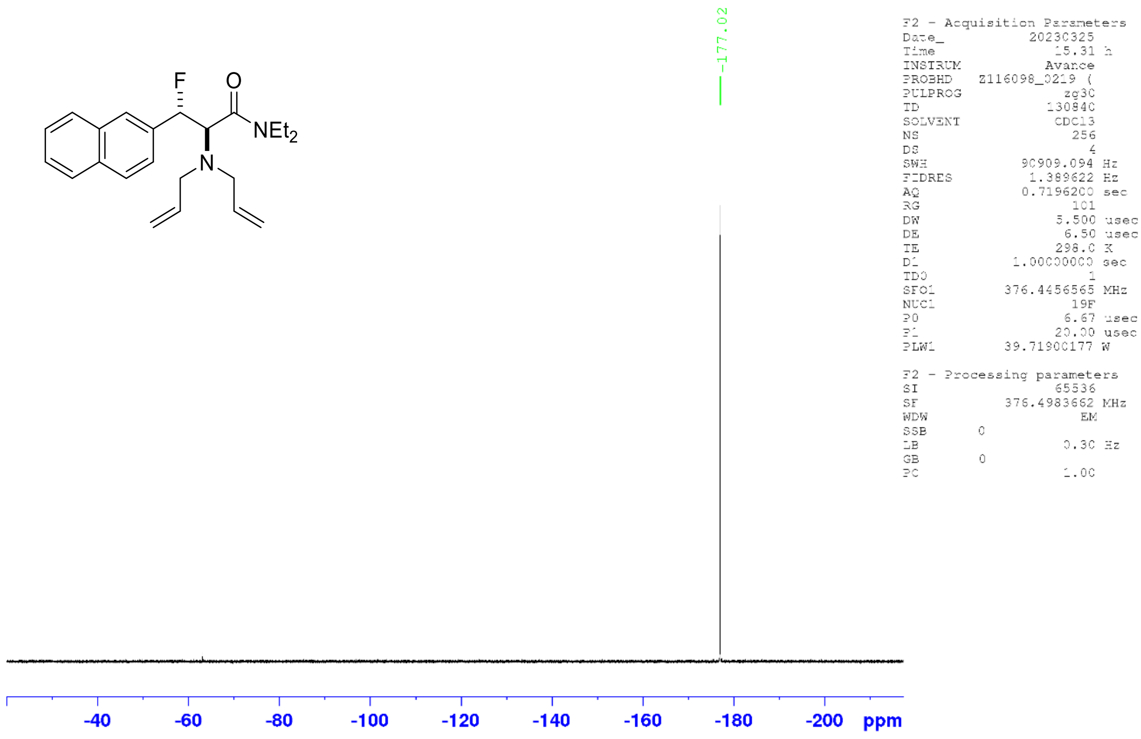

References

1. Pupo, G.; Ibba, F.; Ascough, D. M. H.; Vicini, A. C.; Ricci, P.; Christensen, K. E.; Pfeifer, L.; Morphy, J. R.; Brown, J. M.; Paton, R. S.; Gouverneur, V. "Asymmetric Nucleophilic Fluorination Under Hydrogen Bonding Phase-transfer Catalysis", *Science*, **2018**, *360*, 638−642.
2. Pupo, G.; Vicini, A. C.; Ascough, D. M. H.; Ibba, F.; Christensen, K. E.; Thompson, A. L.; Brown, J. M.; Paton, R. S.; Gouverneur, V. "Hydrogen Bonding Phase-transfer Catalysis with Potassium Fluoride: Enantioselective Synthesis of β-Fluoroamines", *J. Am. Chem. Soc.* **2019**, *141*, 2878−2883.
3. Liedtke, T.; Spannring, P; Riccardi, L.; Gansäuer, A. "Mechanism-based Condition Screening for Sustainable Catalysis in Single-Electron Steps by Cyclic Voltammetry", *Angew. Chem. Int. Ed.*, **2018**, *57*, 5006−5010.
4. Srivastava, R. P.; Zjawiony, J. K.; Peterson, J. R.; McChesney, J. D. "*N*-benzoyl-(2*R*,3*S*)-3-phenylisoserine Methyl Ester; A Facile and Convenient Synthesis and Resolution by Entrainment", *Tetrahedron: Asymmetry*, **1994**, *5*, 1683−1688.
5. Oh, K.; Knabe, W. E. "Lewis Acid-promoted Electron Transfer Deoxygenation of Epoxides, Sulfoxides, and Amine *N*-oxides: The Role of Low-valent Niobium Complexes From $NbCl_5$ and Zn", *Tetrahedron*, **2009**, *65*, 2966−2974.
6. Waser, M.; Herchl, R.; Müller, N. "Ammonium Ylides for the Diastereoselective Synthesis of Glycidic Amides", *Chem. Commun.* **2011**, *47*, 2170−2172.
7. Wilcke, D.; Bach, T. "$Sc(OTf)_3$-catalyzed Diastereoselective Friedel-Crafts Reactions of Arenes and Hetarenes with 3-Phenylglycidates", *Org. Biomol. Chem.* **2012**, *10*, 6498−6503.
8. Legters, J.; Thijs, L.; Zwanenburg, B. "A Convenient Synthesis of Aziridine-2-carboxylic esters" *Recl. Trav. Chim. Pays-Bas* **1992**, *111*, 1−15.
9. Darzens procedure from Reference 3 was used; this compound has previously been isolated by another method: Imashiro, R.; Seki, M. "A Catalytic Asymmetric Synthesis of Chiral Glycidic Acid Derivatives through Chiral Dioxirane-mediated Catalytic Asymmetric Epoxidation of Cinnamic Acid Derivatives", *J. Org. Chem.* **2004**, *69*, 4216−4226.
10. Darzens procedure from Reference 3 was used; this compound has previously been isolated by another method: Tak, R.; Kumar, M.; Menapara, T.; Choudhary, M. K.; Kureshy, R. I.; Khan, N.-u. H. "Asymmetric Catalytic Syntheses of Pharmaceutically Important β-Amino-α-hydroxyl Esters by Enantioselective Aminolysis of Methyl Phenylglycidate", *ChemCatChem* **2017**, *9*, 322−328.
11. Zhao, W.; Kotik, M.; Iacazio, G.; Archelas, A. "Enantioselective Bio-hydrolysis of Various Racemic and *Meso* Aromatic Epoxides Using the Recombinant Epoxide Hydrolase Kau2" *Adv. Synth. Catal.* **2015**, *357*, 1895−1908.
12. Azizi, N.; Saidi, M. R. "Highly Chemoselective Addition of Amines to Epoxides in Water", *Org. Lett.* **2005**, *7*, 3649−3651.
13. Guanti, G.; Banfi, L.; Narisano, E.; Scolastico, C. "Dibenzylaminoacetates as Useful Synthetic Equivalents of Glycine in the Synthesis of α–amino-β-hydroxyacids", *Tetrahedron* **1988**, *44*, 3671−3684.
14. Gani, D.; Hitchcock, P. B.; Young, D. W. "Stereochemistry of Catabolism of the DNA Base Thymine and of the Anti-cancer Drug 5-Fluorouracil", *J. Chem. Soc., Perkin Trans. 1* **1985**, 1363−1372.

15. Kim, Y.; Chang, S. "Borane-Catalyzed Reductive α-Silylation of Conjugated Esters and Amides Leaving Carbonyl Groups Intact", *Angew. Chem. Int. Ed.* **2016**, *55*, 218−222.
16. Nemoto, T.; Kakei, H.; Gnanadesikan, V.; Tosaki, S.-y.; Ohshima, T.; Shibasaki, M. "Catalytic Asymmetric Epoxidation of α,β-Unsaturated Amides: Efficient Synthesis of β-Aryl α-Hydroxy Amides Using a One-pot Tandem Catalytic Asymmetric Epoxidation-Pd-catalyzed Epoxide Opening Process", *J. Am. Chem. Soc.* **2002**, *124*, 14544−14545.
17. Frisch, M. J; Trucks, G. W.; Schlegel, H. B.; Scuseria, G. E.; Robb, M.A.; Cheeseman, J.R.; Scalmani, G.; Barone, V.; Mennucci, B; Nakatsuji, G. A.; Caricato, M.;Li, X.; Hratchian, H. P.; Izmaylow, A. F.; Bloino, J.; Zheng, G.; Sonnenberg, J. L.; Hada, M.; Ehara, M.; Toyota, K.; Fukuda, R.; Hasegawa, J.; Ishida, M.; Nakajima, T.; Honda, Y.; Kitao, O.; Nakai, H.; Vreven, T.; Montgomery, J. A. Jr.; Peralta, J. E.; Ogliaro, F.; Bearpark, M.; Heyd, J. J.; Brothers, E.; Kudin, K. N.; Staroverov, V. N.; Kobayashi, R.; Normand, J.; Raghavachari, K.; Rendell, A.; Burant, J. C.; Iyengar, S. S.; Tomasi, J.; Cossi, M.; Rega, N.; Millam, J. M.; Klene, M.; Knox, J. E.; Cross, J. B.; Bakken, V.; Adamo, C.; Jaramillo, J.;Gomperts, R.; Stratmann, R. E.; Yazyev, O.; Austin, A. J.; Cammi, R.; Pomelli, C.; Ochterski, J. W.; Martin, R. L.; Morokuma, K.; Zakrzewski, V. G.; Voth, G. A.; Salvador, P.; Dannenberg, J. J.; Dapprich, S.; Daniels, A. D.; Farkas, Ö.; Foresman, J. B.; Ortiz, J. V.; Cioslowski, J.; Fox D. J., *Gaussian 09, Revision D.01*, Gaussian, Inc, Wallingford CT, **2009**.
18. Zhao, Y.; Truhlar, D. G. "The M06 suite of density functionals for main group thermochemistry, thermochemical kinetics, noncovalent interactions, excited states, and transition elements: two new functionals and systematic testing of four M06-class functionals and 12 other functionals", *Theor. Chem. Acc.* **2008**, *120*, 215–241.
19. Weigend, F.; Ahlrichs, R. *Phys. Chem. Chem. Phys.* **2005**, *7*, 3297.
20. Hellweg, A.; Rappoport, D. "Balanced basis sets of split valence, triple zeta valence and quadruple zeta valence quality for H to Rn: Design and assessment of accuracy", *Phys. Chem. Chem. Phys.* **2015**, *17*, 1010–1017.
21. Cossi, M.; Rega, N.; Scalmani, G.; Barone, V. " Energies, structures, and electronic properties of molecules in solution with the C-PCM solvation model", *J. Comput. Chem.* **2003**, *24*, 669–681.
22. Barone, V.; Cossi, M. "Quantum Calculation of Molecular Energies and Energy Gradients in Solution by a Conductor Solvent Model", *J. Phys. Chem. A* **1998**, *102*, 1995–2001.
23. Neese, F.; Wennmohs, F.; Becker, U.; Riplinger, C. "The ORCA quantum chemistry program package", *J. Chem. Phys.* **2020**, *152*, 224108.
24. Chai, J.-D.; Head-Gordon, M. "Long-range corrected hybrid density functionals with damped atom–atom dispersion corrections", *Phys. Chem. Chem. Phys.* **2008**, *10*, 6615.
25. Grimme, S.; Antony, J.; Ehrlich, S. H. Krieg, "A consistent and accurate ab initio parametrization of density functional dispersion correction (DFT-D) for the 94 elements H-Pu", *J. Chem. Phys.* **2010**, *132*, 154104.
26. Zheng, J.; Xu, X.; Truhlar, D. G. "Minimally augmented Karlsruhe basis sets", *Theor. Chem. Acc.* **2011**, *128*, 295–305.
27. Paton, R. S.; Funes-Ardois, I. *GoodVibes.Py*, **n.d.**
28. Grimme, S. "Supramolecular Binding Thermodynamics by Dispersion-Corrected Density Functional Theory", *Chem. - Eur. J.* **2012**, *18*, 9955–9964.
29. (a) Chen, P.-P.; Ma, P.; He, X.; Svatunek, D.; Liu, F.; Houk, K. N., "Computational Exploration of Ambiphilic Reactivity of Azides and Sustmann's Paradigmatic Parabola", *J. Org. Chem.* **2021,** *86* (8), 5792–5804. (b) Bickelhaupt, F. M.; Houk, K. N., "Analyzing Reaction Rates with the Distortion/Interaction-Activation Strain Model", *Angew. Chem. Int. Ed.* **2017**, *56* (34), 10070–10086. (c) van Zeist, W.-J.; Bickelhaupt, F. M., "The activation strain model of chemical reactivity", *Org. Biomol. Chem.* **2010**, *8* (14), 3118–3127.
30. Shao, Y.; Gan, Z.; Epifanovsky, E.; Gilbert, A. T. B.; Wormit, M.; Kussmann, J.; Lange, A. W.; Behn, A.; Deng, J.; Feng, X.; Ghosh, D.; Goldey, M.; Horn, P. R.; Jacobson, L. D.; Kaliman,

I.; Khaliullin, R. Z.; Kuś, T.; Landau, A.; Liu, J.; Proynov, E. I.; Rhee, Y. M.; Richard, R. M.; Rohrdanz, M. A.; Steele, R. P.; Sundstrom, E. J.; Woodcock, H. L.; Zimmerman, P. M.; Zuev, D.; Albrecht, B.; Alguire, E.; Austin, B.; Beran, G. J. O.; Bernard, Y. A.; Berquist, E.; Brandhorst, K.; Bravaya, K. B.; Brown, S. T.; Casanova, D.; Chang, C.-M.; Chen, Y.; Chien, S. H.; Closser, K. D.; Crittenden, D. L.; Diedenhofen, M.; DiStasio, R. A.; Do, H.; Dutoi, A. D.; Edgar, R. G.; Fatehi, S.; Fusti-Molnar, L.; Ghysels, A.; Golubeva-Zadorozhnaya, A.; Gomes, J.; Hanson-Heine, M. W. D.; Harbach, P. H. P.; Hauser, A. W.; Hohenstein, E. G.; Holden, Z. C.; Jagau, T.-C.; Ji, H.; Kaduk, B.; Khistyaev, K.; Kim, J.; Kim, J.; King, R. A.; Klunzinger, P.; Kosenkov, D.; Kowalczyk, T.; Krauter, C. M.; Lao, K. U.; Laurent, A. D.; Lawler, K. V.; Levchenko, S. V.; Lin, C. Y.; Liu, F.; Livshits, E.; Lochan, R. C.; Luenser, A.; Manohar, P.; Manzer, S. F.; Mao, S.-P.; Mardirossian, N.; Marenich, A. V.; Maurer, S. A.; Mayhall, N. J.; Neuscamman, E.; Oana, C. M.; Olivares-Amaya, R.; O'Neill, D. P.; Parkhill, J. A.; Perrine, T. M.; Peverati, R.; Prociuk, A.; Rehn, D. R.; Rosta, E.; Russ, N. J.; Sharada, S. M.; Sharma, S.; Small, D. W.; Sodt, A.; Stein, T.; Stück, D.; Su, Y.-C.; Thom, A. J. W.; Tsuchimochi, T.; Vanovschi, V.; Vogt, L.; Vydrov, O.; Wang, T.; Watson, M. A.; Wenzel, J.; White, A.; Williams, C. F.; Yang, J.; Yeganeh, S.; Yost, S. R.; You, Z.-Q.; Zhang, I. Y.; Zhang, X.; Zhao, Y.; Brooks, B. R.; Chan, G. K. L.; Chipman, D. M.; Cramer, C. J.; Goddard, W. A.; Gordon, M. S.; Hehre, W. J.; Klamt, A.; Schaefer, H. F.; Schmidt, M. W.; Sherrill, C. D.; Truhlar, D. G.; Warshel, A.; Xu, X.; Aspuru-Guzik, A.; Baer, R.; Bell, A. T.; Besley, N. A.; Chai, J.-D.; Dreuw, A.; Dunietz, B. D.; Furlani, T. R.; Gwaltney, S. R.; Hsu, C.-P.; Jung, Y.; Kong, J.; Lambrecht, D. S.; Liang, W.; Ochsenfeld, C.; Rassolov, V. A.; Slipchenko, L. V.; Subotnik, J. E.; Van Voorhis, T.; Herbert, J. M.; Krylov, A. I.; Gill, P. M. W.; Head-Gordon, M., "Advances in molecular quantum chemistry contained in the Q-Chem 4 program package", *Mol. Phys.* **2015,** *113* (2), 184–215.

31. Liakos, D. G.; Neese, F. "Is It Possible To Obtain Coupled Cluster Quality Energies at near Density Functional Theory Cost? Domain-Based Local Pair Natural Orbital Coupled Cluster vs Modern Density Functional Theory", *J. Chem. Theory Comput.* **2015**, *11*, 4054–4063.
32. Kendall, R. A.; Dunning, T. H.; Harrison, R.J. "Electron affinities of the first-row atoms revisited. Systematic basis sets and wave functions", *J. Chem. Phys.* **1992**, *96*, 6796–6806.
33. Woon, D. E.; Dunning, T. H. "Gaussian basis sets for use in correlated molecular calculations. III. The atoms aluminum through argon", *J. Chem. Phys.* **1993**, *98*, 1358–1371.
34. Izsák, R.; Hansen, A.; Neese, F. "The resolution of identity and chain of spheres approximations for the LPNO-CCSD singles Fock term", *Mol. Phys.* **2012**, *110*, 2413–2417.
35. Weigend, F. "Accurate Coulomb-fitting basis sets for H to Rn", *Phys. Chem. Chem. Phys.* **2006**, *8*, 1057–1065.
36. Wang, J.; Horwitz, M. A.; Dürr, A. B.; Ibba, F.; Pupo, G.; Gao, Y.; Ricci, P.; Christensen, K. E.; Pathak, T. P.; Claridge, T. D. W.; Lloyd-Jones, G. C.; Paton, R. S.; Gouverneur, V. "Asymmetric Azidation under Hydrogen Bonding Phase-Transfer Catalysis: A Combined Experimental and Computational Study. ", *J. Am. Chem. Soc.* **2022,** *144*, 4572–4584.
37. S. Grimme, J. Chem. *Theory Comput.* **2019**, *15*, 2847–2862.
38. P. Pracht, F. Bohle, S. Grimme, *Phys. Chem. Chem. Phys.* **2020**, *22*, 7169–7192.
39. Palatinus, L.; Chapuis, G. "SUPERFLIP– a computer program for the solution of crystal structures by charge flipping in arbitrary dimensions", *J. Appl. Cryst.* **2007**, *40*, 786−790.
40. (a) Parois, P.; Cooper, R.I.; Thompson A.L. "Crystal structures of increasingly large molecules: meeting the challenges with CRYSTALS software", *Chem. Cent. J.* **2015**, *9*,30. (b) Cooper, R.I.; Thompson, A.L.; Watkin, D.J. "CRYSTALS Enhancements: Dealing with Hydrogen Atoms in Refinement", *J. Appl. Cryst.* **2010**, *43*, 1100−1107.
